# VanillaNet-YOLOv8 segment: detection of nano-iron oxide regulation on rice seedling growth vitality under salt stress

**DOI:** 10.3389/fpls.2025.1631279

**Published:** 2025-09-17

**Authors:** Weihang Jiang, Hongyu Li, Xuanhao Zhao, Renhong Wang, Meng Li, Xiuqing Fu, Zhibo Zhong, Ruxiao Bai, Yang Peng, Feng Pan

**Affiliations:** ^1^ College of Engineering, Nanjing Agricultural University, Nanjing, China; ^2^ Institute of Farmland Water Conservancy and Soil-Fertilizer, Xinjiang Academy of Agricultural Reclamation Science, Shihezi, Xinjiang, China; ^3^ Institute of Mechanical Equipment, Xinjiang Academy of Agricultural Reclamation Science, Shihezi, Xinjiang, China

**Keywords:** nano-iron oxide, salt stress, rice seedling stage, phenotypic detection, YOLOv8, true value calculation, vitality quantification

## Abstract

Rice, a pivotal global food crop, faces a substantial threat from soil salinization during its growth cycle. The present study focuses on the regulatory effects of nano-iron oxide on the growth vitality of rice seedlings under salt stress, and constructs a technical system of “phenotype acquisition-model detection-vitality quantification”. The present study utilised an independently developed high-throughput crop seedling phenotype detection system to obtain 3,888 full-time series growth images of rice seedlings over a period of 90 hours. The image quality was enhanced through preprocessing with a super-resolution algorithm (SSN). In order to address the challenges associated with detecting rice seedlings, which are characterised by their diminutive size and dense growth patterns, the YOLOv8-seg model has been enhanced. In this regard, the VanillaNet-YOLOv8 Segment model has been proposed. The VanillaNet concise backbone network was utilised to reduce computational complexity. The DualVit dual visual attention mechanism was introduced to decouple global semantics and local features to solve instance adhesion. A small object detection module was added to improve the recognition ability of weak seedlings, and the Real Value module was used to correct lens distortion and phototropic tilt to achieve accurate quantification of the true seedling length. The experimental findings demonstrate that the enhanced model attains a target detection accuracy (mAP50) of 98.4% and a segmentation accuracy (mAPmask50) of 96.4%, representing an improvement of 3.2% and 16.6%, respectively, over the original YOLOv8n-seg, while preserving its lightweight advantages. The “static vitality (average seedling length)-dynamic vitality (growth rate)” dual-index evaluation system was utilised to ascertain the most significant promoting effect on rice growth under salt stress (0-150 mmol/L NaCl). It was found that 300 mg/L nano-iron oxide had the most significant promoting effect on rice growth under salt stress, especially in terms of alleviating the inhibitory effect under severe salt stress. The present study provides an efficient and accurate technical framework for the evaluation of nanomaterial agricultural applications and the screening of salt-tolerant crops.

## 1 Introduction

Rice (*Oryza sativa L.*) is one of the most widely distributed food crops in the world and occupies a pivotal position in the global food security system. Rice is cultivated and consumed by nearly half of the global population, making it a staple food of significant importance to the global food supply. In China, rice is referred to as “the head of the five grains” and plays a significant role in the nation’s food production. Statistical analysis indicates that approximately 65% of the Chinese population consumes rice as their primary source of carbohydrates (Zhang et al., 2021), thereby establishing rice as a predominant component of the national dietary landscape. Moreover, rice constitutes not only a pivotal food crop but also a crucial pillar crop of China’s agricultural economy, exerting a profound influence on national food security and economic development.

Rice (*Oryza sativa L.*) is a crop that is highly sensitive to salt stress, and its growth and yield are significantly affected by soil salinization. Soil salinization, a major environmental challenge confronting contemporary agriculture, poses a grave threat to the sustainable development of agriculture (Jia et al., 2022). It has been established that elevated concentrations of sodium ions (Na^+^) and chloride ions (Cl^-^) in saline soil have the capacity to compromise the water and nutrient absorption mechanisms of plant cells (Yang and Guo, 2018), thereby inducing impairment to crop physiological functions. Research has demonstrated that salt stress can also significantly reduce the photosynthetic efficiency of crops, hinder the synthesis of carbohydrates, and ultimately lead to a significant decline in crop yields (Liang et al., 2018; Li et al., 2022). Globally, salt damage affects more than 7.32% of the land area (Wicke et al., 2011; Liu and Wang, 2021), while in China, the area of saline-alkali land accounts for 3.5%-10.3% of the national land area, distributed in 17 provinces across the country (Liu and Wang, 2021), posing a major threat to agricultural production. Consequently, there is a need for in-depth research on the salt tolerance mechanism of rice and the development of salt-tolerant varieties. This research is of great significance for improving the productivity of rice in saline-alkali land and ensuring global food security.

In order to address the issue of soil salinization and its subsequent impact on crop growth, the utilisation of seed priming technology as a novel seed treatment method has garnered significant attention. The process of seed priming has been shown to enhance the salt stress resistance of plants. This is achieved by treating seeds with priming agents prior to germination, thereby regulating key information molecules (such as transcription factors) in plants (Goyal et al., 2021). In recent years, there has been a growing body of research highlighting the significant potential of nanomaterials in promoting plant growth and increasing crop yields. This is due to their unique physical and chemical properties. Research has demonstrated the efficacy of various nanomaterials, including zinc oxide nanoparticles (ZnO NPs), selenium nanoparticles (SeNPs), and cerium oxide nanoparticles (CeO2 NPs), in the field of seed priming (Rawashdeh et al., 2020; Prakash et al., 2021; Sardar et al., 2022). Specifically, nano-iron oxide has demonstrated remarkable efficacy in enhancing the germination and seedling growth of aromatic rice (Guha et al., 2018) and Dracocephalum moldavica (Sabaghnia et al., 2016) under conditions of drought stress. Furthermore, the findings indicate a substantial concentration dependence in the effects of nano-iron oxide concentrations and particle sizes on the growth of watermelon seedlings. Consequently, the potential of nano-iron oxide in enhancing crop stress resistance is worthy of further research.

The observation of the evolution of seedling stage characteristics during the process of rice germination is fundamental to the evaluation of its germination vitality. This constitutes the basis for the study of the effects of nano-iron oxide priming on rice seedling germination under salt stress. Presently, the detection methods for rice seedling morphology are principally divided into two categories: traditional manual detection and automatic detection based on machine learning. Conventional methods depend on manual observation, measurement, and calculation. However, due to the diminutive size of rice seedlings and the high planting density characteristic of field production, these methods are encumbered by significant disadvantages, including protracted time consumption, elevated cost, and substantial error (Joosen et al., 2010). Conversely, machine learning-based (Jiang et al., 2023) technologies offer a more efficient and accurate solution for seedling feature detection. For instance, Adams Begue et al. utilised machine learning technology to achieve automatic recognition of medicinal plants (Begue et al., 2017), while Lavika et al. applied it to the classification and identification of plant diseases (Goel and Nagpal, 2022), and Meshach Ojo Aderele’s team combined machine learning with agroecosystem modelling (Begue et al., 2017). Nevertheless, the establishment of existing machine learning models still faces many challenges, including the reliance on manual experience to adjust model parameters and the tendency to overfit during training. The present study proposes a rapid, cost-effective, precise and automated method for the detection of characteristics associated with the initial stages of rice seedling development. The objective of this study is to facilitate a comprehensive exploration of the impact of nano-iron oxide priming on the process of rice seedling germination. This method has the potential to enhance experimental efficiency and provide scientific substantiation for rice breeding under salt stress.

Deep learning, a significant component of the machine learning domain, employs multi-layer artificial neural networks (ANNs) to emulate brain functions, thereby facilitating automatic data analysis and efficient feature extraction. Within the deep learning framework, convolutional neural networks (CNN) and recurrent neural networks (RNN) are two common neural network models (Ye et al., 2023). Of these, CNN has been found to be particularly effective for the detection of phenotypic traits in rice seedlings, due to its proven ability to process spatial structure data, such as images and videos (Krizhevsky et al., 2017). In the field of deep convolutional neural network-based object detection models, the YOLO (You Only Look Once) algorithm has garnered significant attention due to its high accuracy and rapid detection capabilities. Since the advent of YOLOv1 in 2015, the YOLO series has undergone continuous iteration and has been extensively applied in fields such as transportation, security, industry, and agriculture (Terven et al., 2023). In the domain of agriculture, the implementation of the YOLO algorithm has been instrumental in propelling the advancement of contemporary agricultural practices. For instance, Cheng et al. employed YOLOv9-seg to segment the ROI of asparagus, subsequently deriving the average diameter and morphological bone length of asparagus from the segmented images. The mean absolute error in length measurement was found to be 0.9 cm, with a mean relative error of 3.5%. These findings demonstrate the viability of the proposed method (Chen et al., 2025). As posited by Wu et al., the Segment module was enhanced through the integration of the SegNext-Attention mechanism and the CAL module, thereby facilitating the effective calculation of the morphological evolution of radicle contour features with growth (Wu et al., 2024). Hed et al. developed an algorithm known as ALSS-YOLO-Seg, which applied the precise segmentation of UAV-captured images to yield estimation and plant health assessment in banana plantations (He et al., 2024). As posited by Wu et al., the incorporation of the MSDA-CBAM and DR-Neck feature fusion network into the YOLOv8-seg model has been shown to enhance the segmentation and processing efficiency of tea garden roads in hilly areas. This integration has been demonstrated to result in an improvement of 0.6% in accuracy, 1.6% in AP@0.5, and a 17.1% reduction in inference time (Paul et al., 2024). Furthermore, Ayan et al. successfully achieved precise detection of chili pedicels using the YOLOv8s-seg model, thereby providing technical support for the development of intelligent agricultural robot harvesting systems (Paul et al., 2024).

YOLOv8 is an open-source object detection model developed by Ultralytics based on YOLOv5. It exhibits a number of advantages over its predecessor, including faster detection speed, higher accuracy, and a unified training framework. It is capable of performing multiple tasks such as object detection, instance segmentation, image classification, and human pose estimation (Terven et al., 2023). It is evident that instance segmentation technology has the capacity to identify the category and location of targets. Furthermore, it has been demonstrated to provide pixel-level object instance information. The technology has been widely applied in fields such as security monitoring, autonomous driving, and medical imaging. In the context of smart agriculture, instance segmentation technology, with its pixel-level fine analysis capability, is of great significance for key links in agricultural production such as crop phenotypic analysis and growth monitoring.

However, in the context of agricultural applications, such as the analysis of rice seedling stage phenotypic (Qiao et al., 2022), the implementation of instance segmentation technology encounters two significant challenges. Firstly, the small size of rice seedlings, their dense growth, and the instance adhesion caused by phototropism (Scheres et al., 1995) result in a substantial decline in the mask average accuracy of the traditional YOLOv8s-seg model. Secondly, lens radial distortion and geometric deformation of phototropic growth from an overhead perspective lead to a deviation between the pixel-level perimeter measurement value and the true seedling length. Despite the fact that earlier studies have enhanced the speed of detection through the implementation of lightweight modifications, these studies continue to encounter difficulties in fulfilling the dual requirements of accuracy and real-time performance for the assessment of rice seedling growth vitality under conditions that are both complex and characterised by dense planting. In order to address these issues, this paper improves the YOLOv8n-seg model and proposes the VanillaNet-YOLOv8 segment model. This achieves performance breakthroughs through multi-module collaborative optimisation and the addition of functional modules for post-segmentation visualisation design and real-value conversion calculation of seedling stage features. The purpose of this is to achieve efficient and accurate assessment of rice seedling growth vitality. The subsequent discussion will address the following tasks:

1. Relying on an independently developed seed germination phenotyping detection system, high-throughput acquisition of 90-hour seedling stage images was achieved in a multi-factor experiment with salt stress and nano-iron oxide priming, constructing a rice seedling stage dataset containing 3,888 images.
2. Replacing the backbone network with the VanillaNet concise backbone network, reducing the number of convolutional layers while maintaining feature extraction capabilities; introducing the DualVit dual visual attention mechanism to fuse global seedling shape semantics and local color features to solve instance overlap problems caused by phototropism and dense growth; adding a small target module to improve detection accuracy for the small size of rice seedlings.
3. Designing a visualization design module and a real value module to convert segment output values into readable pixel values and achieve precise conversion from pixel-level perimeter to actual seedling length through lens distortion correction and phototropism correction.
4. Combining full-time series monitoring data, quantifying the regulatory effects of different concentrations of nano-iron oxide (0-300 mg/L) on rice seedling length and growth rate under salt stress (0-150 mmol/L NaCl), and constructing a “static viability (average seedling length) - dynamic viability (growth rate)” dual-index evaluation system.

## 2 Materials and methods

### 2.1 Full-time series monitoring system for crop seedling stage

**Figure 1:**
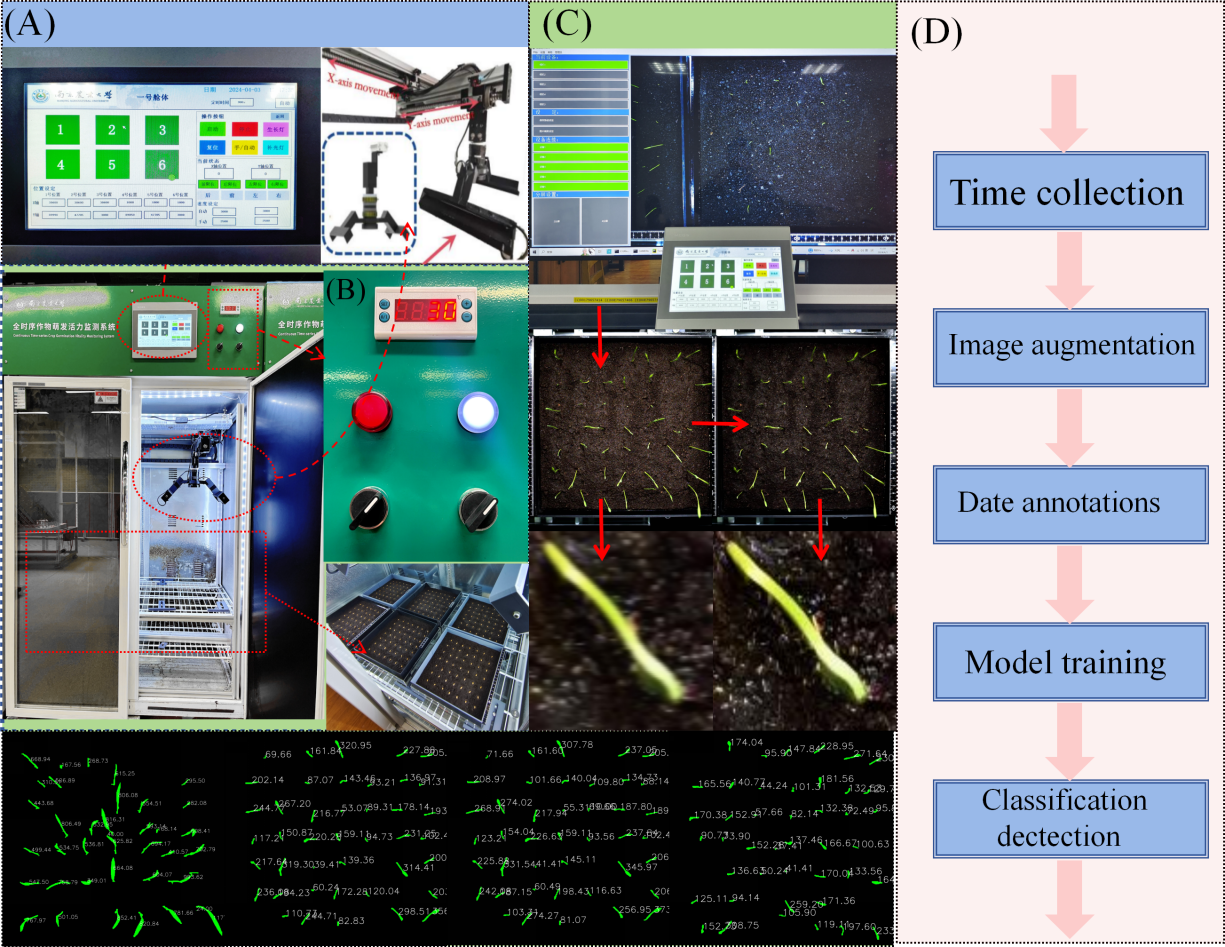
Full-time series detection system for crop seedling stage **(A)** Human-machine interaction module. **(B)** Environmental control module and seed germination module. **(C)** Image processing module. **(D)** Schematic diagram of model training process.

An independent full-time series, high-throughput crop seedling phenotype detection system was developed (see **Figure 1**), which is used to cultivate and continuously monitor the growth process of rice seeds. The system is composed of two core modules: an environmental control module and a seed germination module. These modules are capable of providing precise temperature, humidity, and soil conditions for seeds, thereby creating a controllable environment for experiments studying the effects of different growth conditions on seed germination. At the stage of the experiment at which the seeds have reached the requisite phase of development, the system utilises an orbital high-throughput germination image acquisition module. This module is designed to collect high-resolution images of rice growth. The image acquisition module utilises a programmable logic controller (PLC) program to regulate a stepper motor, systematically actuating an RGB imaging sensor (resolution 5472×3648, 20 million pixels) to translate in the X and Y axes with high precision. The camera communicates with the host via a GigE Ethernet interface, and users can adjust the camera focus, set the shooting interval, and configure image preprocessing parameters through a human-machine interface. The collected image data is finally transmitted to the host, where users can complete data preprocessing and model training through the image processing module.

### 2.2 Data collection and processing

**Figure 2:**
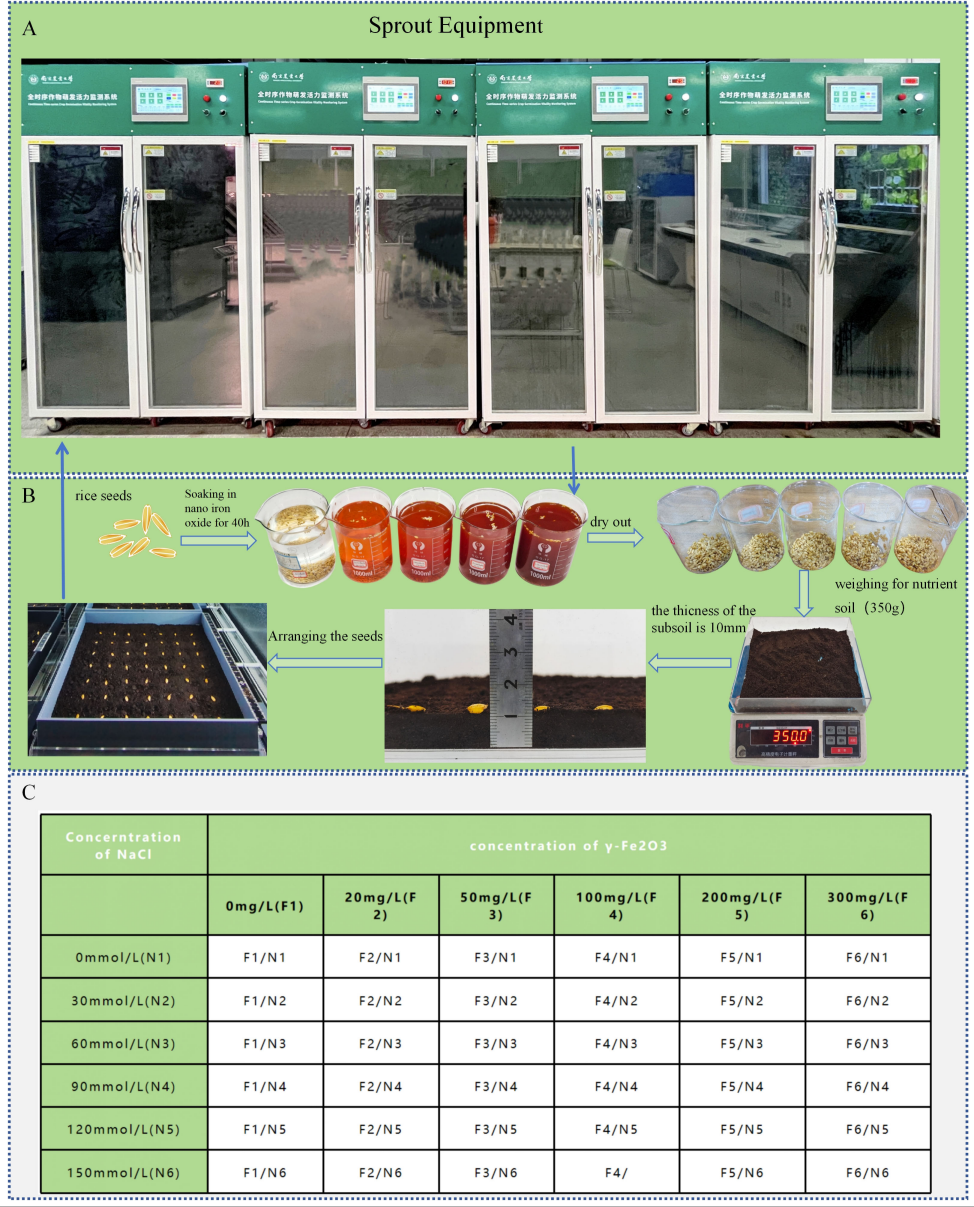
Pre-experiment preparation **(A)** Sprout equipment. **(B)** Seed treatment process. **(C)** Parameter table for salt solution concentrations and nano-iron oxide dispersion concentrations.

The medium-maturing medium-japonica rice variety Nanjing 58 (Nan Geng 58), which was bred by the Food Crop Research Institute of Jiangsu Academy of Agricultural Sciences, was selected for this experiment. The experiment (see **Figure 2**) employed soil culture techniques, utilising six distinct concentrations of salt solutions to emulate salt stress environments. A total of 500 millilitres of each concentration was allocated within the chassis of the soil culture apparatus. Concurrently, six concentration gradients of nano-iron oxide (III) dispersion were utilised as seed pretreatment agents, and the seeds were immersed in a constant temperature environment of 28°C for 40 hours for priming treatment. Following this, the seeds were extracted and dried to remove excess moisture, thereby yielding the pretreated seeds. The soil culture box was prepared by means of 3D printing, with specifications of 250mm × 250mm, and the thickness of the bottom soil was approximately 10mm (350 ± 2g). The pretreated seeds were then arranged in a 7 × 7 grid configuration within the designated culture box. The precise parameters for salt solution and nano-iron oxide concentrations are delineated in Table C.

**Figure 3:**
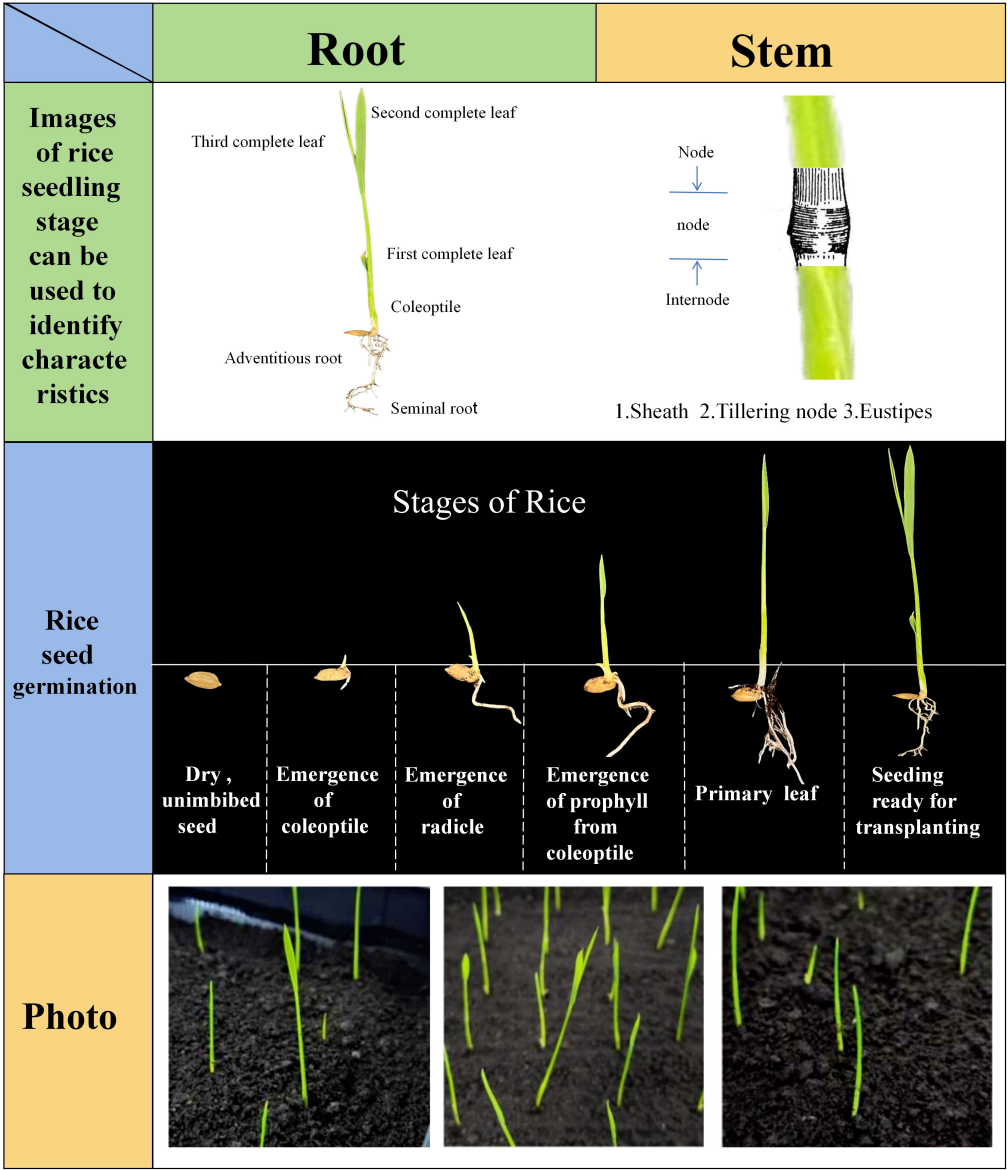
Observable characteristics of rice seedlings.

The prepared culture boxes were placed in a crop seedling phenotype detection incubator, with the temperature set to a constant 28°C through the environmental control module and normal light conditions provided to promote seed germination and a suitable temperature for root growth. When the chest-breaking rate (white exposure rate) of the control group (CK) seeds reached 80%, the incubator temperature was adjusted to 25°C for the purpose of promoting seedling growth (Chen et al., 2024). During the experiment, 1-2 millilitres of the original soaking concentration of nano-iron oxide (III) dispersion was added to each group of rice seeds at 12-hour intervals. Preliminary experimentation yielded results indicating that the germination and seedling growth periods of rice seeds each lasted 90 hours. The present study focused on the 90 hours of seedling growth. During the cultivation process, the rice seedlings exhibited six discernible growth stages, characterised by quantifiable attributes (see **Figure 3**) such as seedling length, internode length, and leaf area. Of these, the seedling length exhibited the most substantial alterations and was readily discernible; consequently, the seedling length growth rate was utilised as a pivotal metric to assess the growth vitality of rice seedlings. In order to circumvent the complications that seedling lodging can pose during image analysis, the soil covering treatment was implemented in the early stages of seedling growth in the formal experiment. This approach effectively mitigates the issue of seedling lodging and overlap as they become taller. The experiment spanned a duration of 180 hours, with a 90-hour period dedicated to the observation of seedling growth. During this phase, images were collected at 50-minute intervals, yielding a total of 3,888 full-time series images depicting the growth of rice seedlings. A total of 640 high-quality images were meticulously selected for subsequent data analysis.

The data processing stage entailed the utilisation of Labelme software for the manual execution of semantic segmentation labelling on the radicles of rice seeds. These radicles functioned as the training dataset for the deep learning process of the neural network. The folder containing the dataset was imported into the Labelme software, and the Create Polygons tool was used to annotate the boundaries of the rice radicles, marking them as ROOT and saving them as.json files. In order to translate the image data into a format comprehensible by the computer, it was necessary to convert the.json files into.txt files. In accordance with the requirements of the model training, 640 images of seedling growth were divided into a training set, a test set, and a validation set at a ratio of 8:1:1.

During the preliminary trial training, it was found that overfitting occurred during the training process. Therefore, it was necessary to further improve the diversity and richness of the dataset to enhance the model’s robustness. The augmentation of the original training set was achieved by employing data augmentation techniques, including image sharpening, image rotation, and brightness adjustment. This expansion resulted in the augmentation of the original training set to 800 images, while the test and validation sets remained unaltered.

During the process of dataset organisation, it was discovered that the inherent defects of the RGB camera resulted in blurry radicle edges, thereby affecting the accuracy of both manual annotation and computer vision recognition. To address this, a super-resolution (SR) algorithm was introduced for image processing to optimize data quality. In the domain of computer vision (CV), super-resolution technology addresses the issue of image detail loss by restoring high-resolution (HR) images from low-resolution (LR) images. The fundamental principle underlying this approach is the utilisation of information and prior knowledge contained within images to infer and restore details that have been lost due to technical defects. This process serves to enhance image clarity and detail performance (Lim et al., 2017). Conventional super-resolution algorithms principally depend on interpolation techniques to enhance image resolution by augmenting the number of pixels; however, they are unable to restore lost high-frequency information. The advent of deep learning technology has precipitated a series of breakthroughs in the domain of super-resolution algorithms based on convolutional neural networks (CNNs). These advancements have led to substantial progress in the field of image mapping relationship construction, thereby enabling the reconstruction of high-quality images and the enhancement of the performance of computer vision tasks.

**Figure 4:**
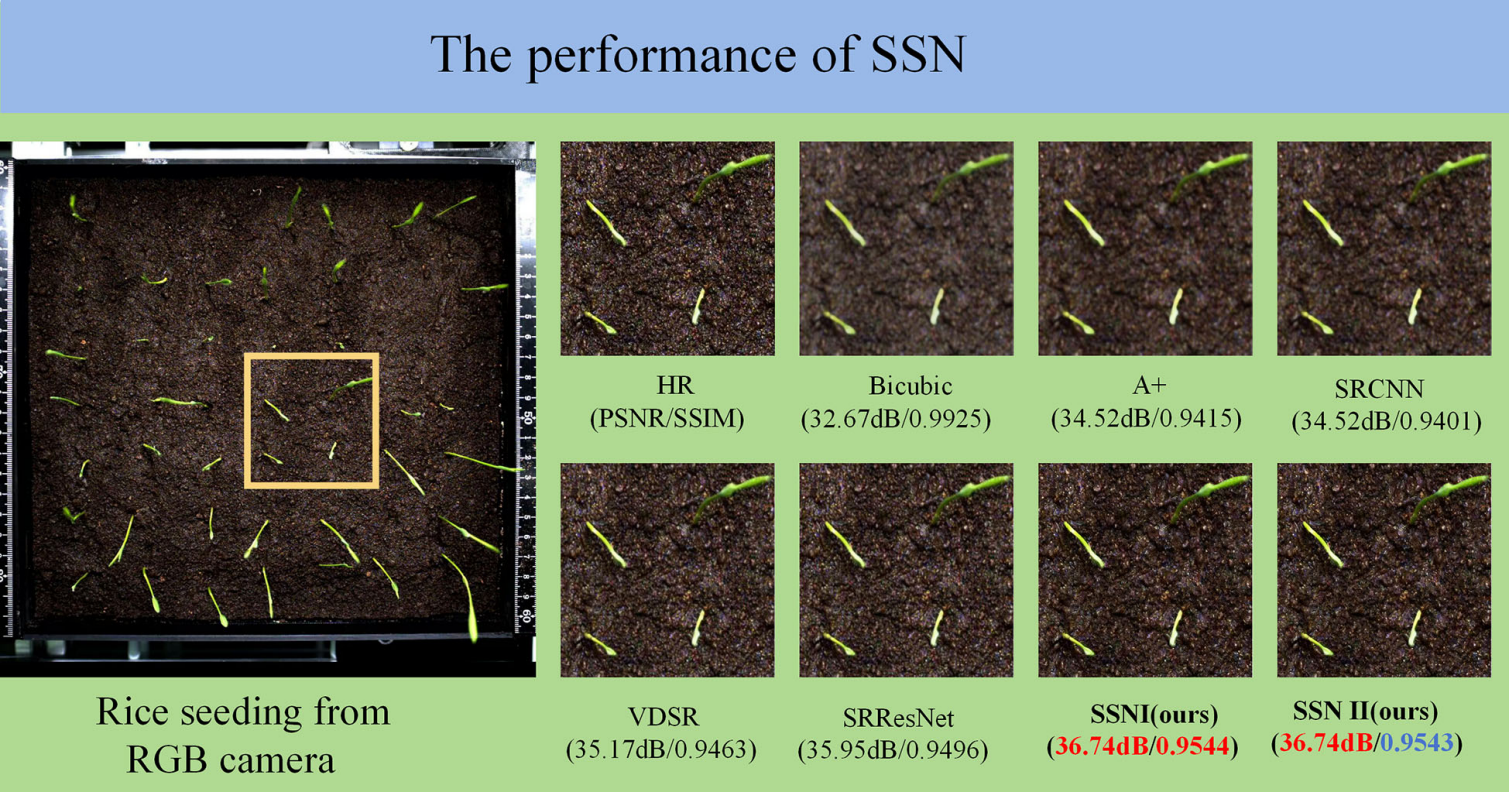
Performance comparison diagram of SSN and other super-resolution algorithms.

In this experiment, SRGAN (Super-Resolution Generative Adversarial Network) technology was utilised to achieve super-resolution. SRGAN employs a generative adversarial network (GAN) to address super-resolution tasks, achieving a substantial enhancement in the realism of reconstructed images through the integration of perceptual loss and adversarial loss (Lim et al., 2017). In order to enhance the performance of the model, the network structure of SRGAN was improved. This involved the removal of batch normalization (BN) layers, with the saved computational resources being utilised to augment convolutional neural network (CNN) sub-modules. This process served to enhance the model’s expressiveness. The enhanced model was designated Single-Scale Net (SSN). Furthermore, a more efficient loss function was selected, thus resulting in SSN exhibiting superior convergence characteristics in comparison to the original SRGAN. The experimental results demonstrated that SSN exhibited superior performance in terms of image reconstruction quality when compared to SRGAN. The SSN super-resolution algorithm was applied to the preprocessing of rice seedling stage images, with a consequent enhancement of clarity and detail performance in unlabeled datasets. The performance comparison between SSN and other super-resolution algorithms is demonstrated in **Figure 4**.

### 2.3 Design of VanillaNet-YOLOv8 segment rice seedling length semantic segmentation based on YOLOv8n-segment

**Figure 5:**
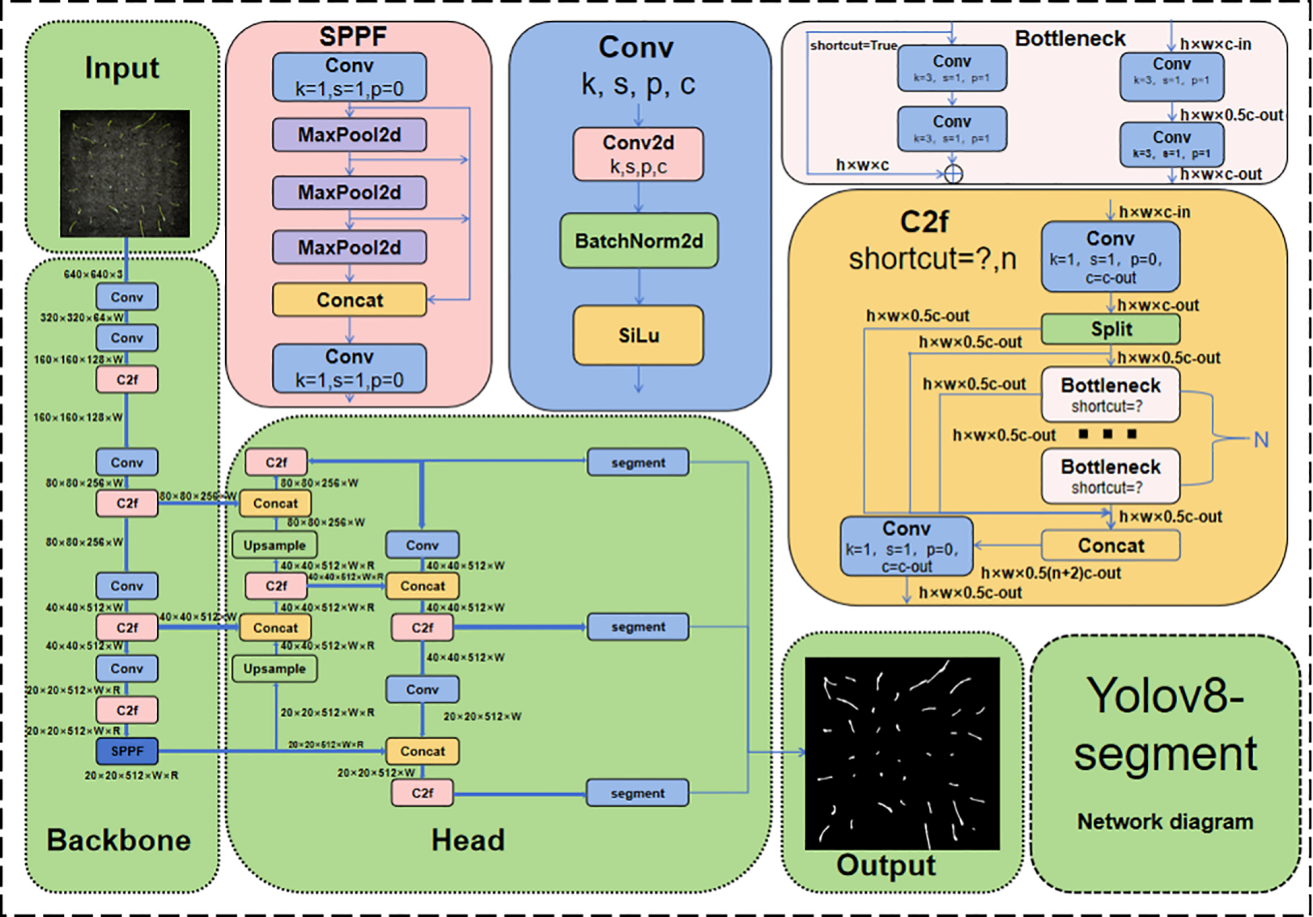
Neural network structure diagram of YOLOv8n-segment.

The YOLOv8n-segment network structure is an object detection and semantic segmentation network based on the YOLO (You Only Look Once) framework. The backbone network utilises a deep residual network (ResNet) architecture to enhance the extraction of features (Li and He, 2018). In order to further enhance performance, YOLOv8n-segment integrates multiple technical modules, including the C2f module, Spatial Pyramid Pooling-Fast module (SPPF), and Convolution (Conv) module (Wang et al., 2023). The C2f module has been shown to enhance the lightweight performance of the model by introducing rich gradient flow information, the SPPF module enhances adaptability to target sizes through multi-scale feature extraction, and the Conv module is used to extract image features layer by layer (Wang et al., 2023). The synergy of these technologies has been demonstrated to enhance the accuracy and efficiency of YOLOv8n-segment in object detection and semantic segmentation tasks. The network structure diagram of YOLOv8n-Segment is shown in **Figure 5**.

In order to enhance the precision of rice seedling stage detection, augment the model’s capacity to discern diminutive targets, and imbue the model with the aptitude to semantically segment rice seedling phenotypes and generate ground truths, the following enhancements were made to the model:

1. Backbone network replacement: A segment of the VanillaNet backbone network was transferred to the YOLOv8n-seg backbone network. The elimination of residual connections and a proportion of the attention modules resulted in a simplified network structure, thus circumventing complex operations such as high depth, shortcuts and self-attention. Consequently, this approach enhanced the model’s speed and accuracy.
2. Introduction of dual visual transformer structure: A MergeBlock self-attention mechanism was added after the C2f module located in the Head position to use global semantics for self-attention learning, significantly improving model accuracy and reducing training complexity.
3. Addition of micro target detection module: A micro-target segmentation head was newly introduced in the Head section to strengthen the model’s segmentation ability for small targets. Additionally, SPD-Conv was used to replace the stride operations in all convolutional and pooling layers, enabling the model to perform better in complex tasks involving low-resolution images and small objects.

**Figure 6:**
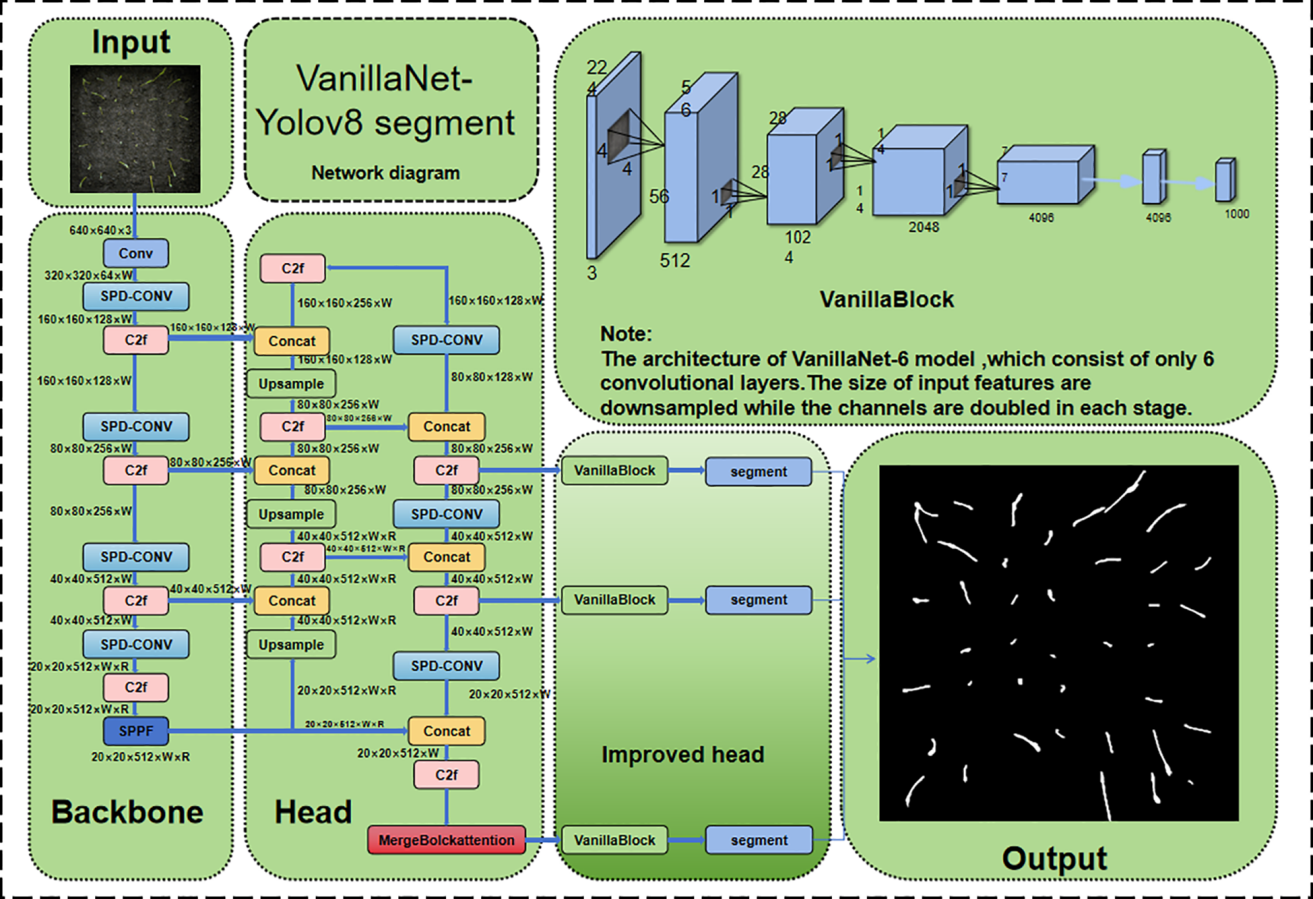
Improved neural network structure diagram of VanillaNet-YOLOv8 segment.

**Figure 6** shows the improved VanillaNet-YOLOv8 Segment network structure. The following sections will elaborate on the VanillaNet backbone replacement, DualViT self-attention mechanism, and micro-target detection module in detail.

#### 2.3.1 Design of concise segmentation model based on VanillaNet

Traditional segmentation networks commonly use the Residual Network (ResNet) architecture, which offers excellent segmentation performance (He et al., 2016). However, in rice seedling segmentation, high computational complexity and deployment difficulties have become bottlenecks. To address this, this study proposes a simple segmentation model based on VanillaNet, which achieves efficient segmentation while maintaining high performance by simplifying the network structure.

VanillaNet-Segment inherits the concise design philosophy of VanillaNet, avoiding excessive depth and complex operations, significantly reducing computational overhead, and making it suitable for large-scale data sets. It adopts the activation functions of VanillaNet (He et al., 2016) (including multiple learnable affine transformations), eliminating the need for non-linear layers while balancing inference speed and merging convenience. The architecture retains the main trunk, body, and fully connected layers of the segment network, but each stage uses only one layer. Taking a 6-layer convolutional network as an example: the Stem part uses 4×4 convolutions for feature transformation, the Body part uses MaxPool downsampling and 1×1 convolutional kernels to minimise computational cost, and the Head part introduces two non-linear layers and performs batch normalisation to optimise training.

**Table 1** compares the performance of different models. The experimental results show that VanillaNet-Segment achieves a mIOU of approximately 89% while significantly reducing inference latency compared to models based on ResNet, DenseNet, UNet, and YOLOv8-Segment (59.2% reduction compared to UNet, 79.4% reduction compared to ResNet, 91.5% reduction compared to DenseNet, YOLOv8-Segment by 79.3%); it reduces the number of convolutional layers by 36.4% and achieves a 56.6% reduction in the number of parameters; in a small-sample subset (160 images) experiment, the initial accuracy is 15.2% higher than that of YOLOv8-Segment, demonstrating strong generalisation ability.

**Table 1:** Performance comparison of VanillaNet and other deep networks.

Model	mIOU (%)	GPU lantency	Parameter
UNet-22	89.1	4.56	26.3
ResNet-40	88.9	9.02	34.2
DenseNet-129	89.3	21.86	41.8
YOLOv8-Segment	**89.7**	6.86	28.6
VanillaNet-Segment	**89.7**	**1.86**	**12.4**

The bolded words indicate the best performance.

Compared with deep networks such as ResNet, DenseNet, and UNet, VanillaNet reduces redundant modules and complex connections to lower computational and memory overhead. It also optimises the design of convolution layers and activation functions to ensure efficient feature extraction. By adopting improved optimisation algorithms and learning rate scheduling, it improves training speed and stability, demonstrating excellent performance in image classification and object detection tasks with good generalisation ability.

In summary, VanillaNet-Segment offers an efficient solution for agricultural applications such as rice seedling segmentation with its simple design and efficient feature extraction, reducing computational costs while meeting the efficiency and accuracy requirements of smart agriculture.

#### 2.3.2 DualVit attention

To solve the problem of instance adhesion (instance adhesion) caused by phototropism and dense growth - the pixel-level boundary blur of adjacent radicles, this study introduces the DualVit attention mechanism, which decouples global semantics and local details through a dual-path feature fusion strategy to achieve precise segmentation in complex scenarios and improve segmentation accuracy.

**Figure 7:**
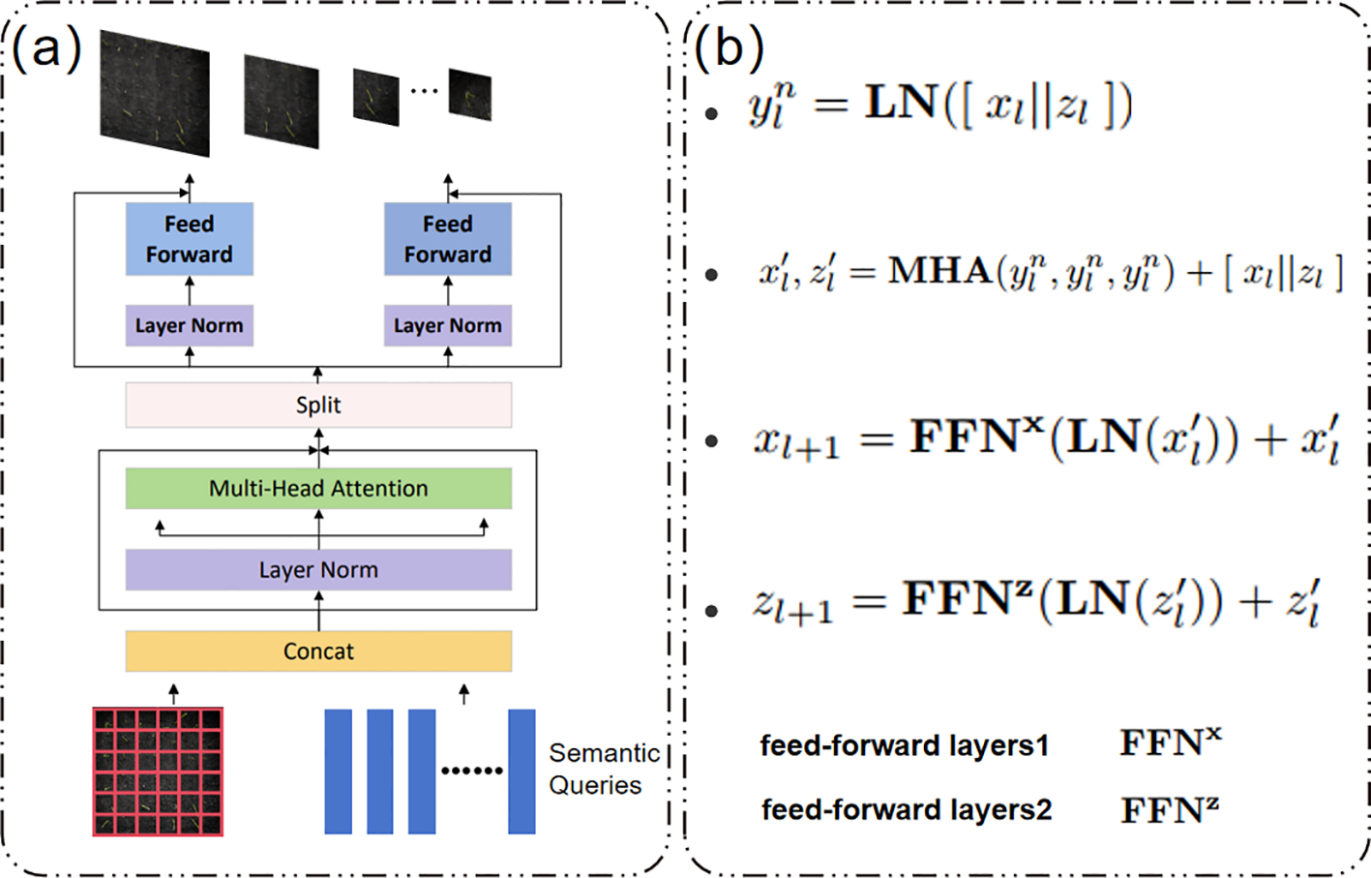
**(a)** Merge block; **(b)** Operation formula of merge block.

The Transformer architecture (Wang et al., 2020; Dosovitskiy et al., 2021; He et al., 2016) has revolutionised computer vision tasks (Chen et al., 2020; Liu et al., 2021), but its reliance on intensive self-attention computations limits its training efficiency for high-resolution complex images, thereby constraining its development. DualVit adopts a dual ViT architecture, decomposing training into global semantic and internal feature attention, comprising two paths. One path is used to extract a more comprehensive global view of input semantic features, employing a deep Transformer encoder to capture the overall morphological features of seedlings (e.g., bending angles, stem orientation) through a global self-attention mechanism, generating global feature maps containing spatial layout information. This path models long-range dependencies through eight layers of multi-head attention (Head=8), effectively distinguishing structural differences between overlapping seedlings. The other path focuses on learning internal local features through a pixel-level pathway, using lightweight convolutional blocks (3×3 depth-separable convolutions) to extract local colour and texture features (such as leaf sheath green saturation and root tip brightness), generating pixel-level detail feature maps.Five layers of cascaded convolutions (channel numbers 64, 128, and 256) retain high-frequency edge information to address the blurred boundaries of micro-level seedlings. As shown in **Figure 7a**, these two paths are referred to as the ‘semantic path’ and ‘pixel path’ (Yao et al., 2023). Dualvit considers the dependency between global semantic and local features on the two paths to reduce token size and attention, thereby simplifying training.

As shown in **Figure 7b**, the Merge block operation formula involves the two paths being processed through pre-integration layer 1 and pre-integration layer 2 in the Merge block, and finally generating classification information through global average pooling. This mechanism effectively addresses instance adhesion caused by phototropism, providing a critical technical foundation for the precise assessment of rice seedling growth vitality under salt stress.

As shown in **Figure 7b**, the Merge block operation formula involves the two paths being processed through pre-integration layer 1 and pre-integration layer 2 in the Merge block, and finally generating classification information through global average pooling. This mechanism effectively addresses instance adhesion caused by phototropism, providing a critical technical foundation for the precise assessment of rice seedling growth vitality under salt stress.

#### 2.3.3 Small target detection design

For the YOLOv8 model, rice seedling detection falls under the category of small object segmentation. Small object segmentation poses significant challenges: the objects themselves have low resolution, limiting the amount of global information available for model learning; when large and small objects coexist in an image, the model is prone to being dominated by the features of large objects, leading to failed segmentation of small objects. An analysis of the YOLOv8 model mechanism reveals that cross-convolution layers and pooling operations filter redundant pixel information, while its three-target segmentation head design is more suitable for extracting information from medium-sized targets. In complex scenarios with blurred images or extremely small targets, due to insufficient target information and the inability of the three-segmentation head to extract information that meets the redundancy assumption conditions, the model suffers from fine-grained information loss and weakened feature learning capabilities.

To address these challenges, this study proposes a two-stage optimisation architecture: ‘backbone enhancement - specialised detection head.’ The backbone is replaced with SPD-Conv layers (Sunkara and Luo, 2022), which consist of deep layers and non-cross-row convolutional layers, replacing traditional cross-row convolutions and pooling. This preserves the original channel information of feature maps while downsampling. The detection head is augmented with a dedicated segmentation head for small objects.

**Figure 8:**
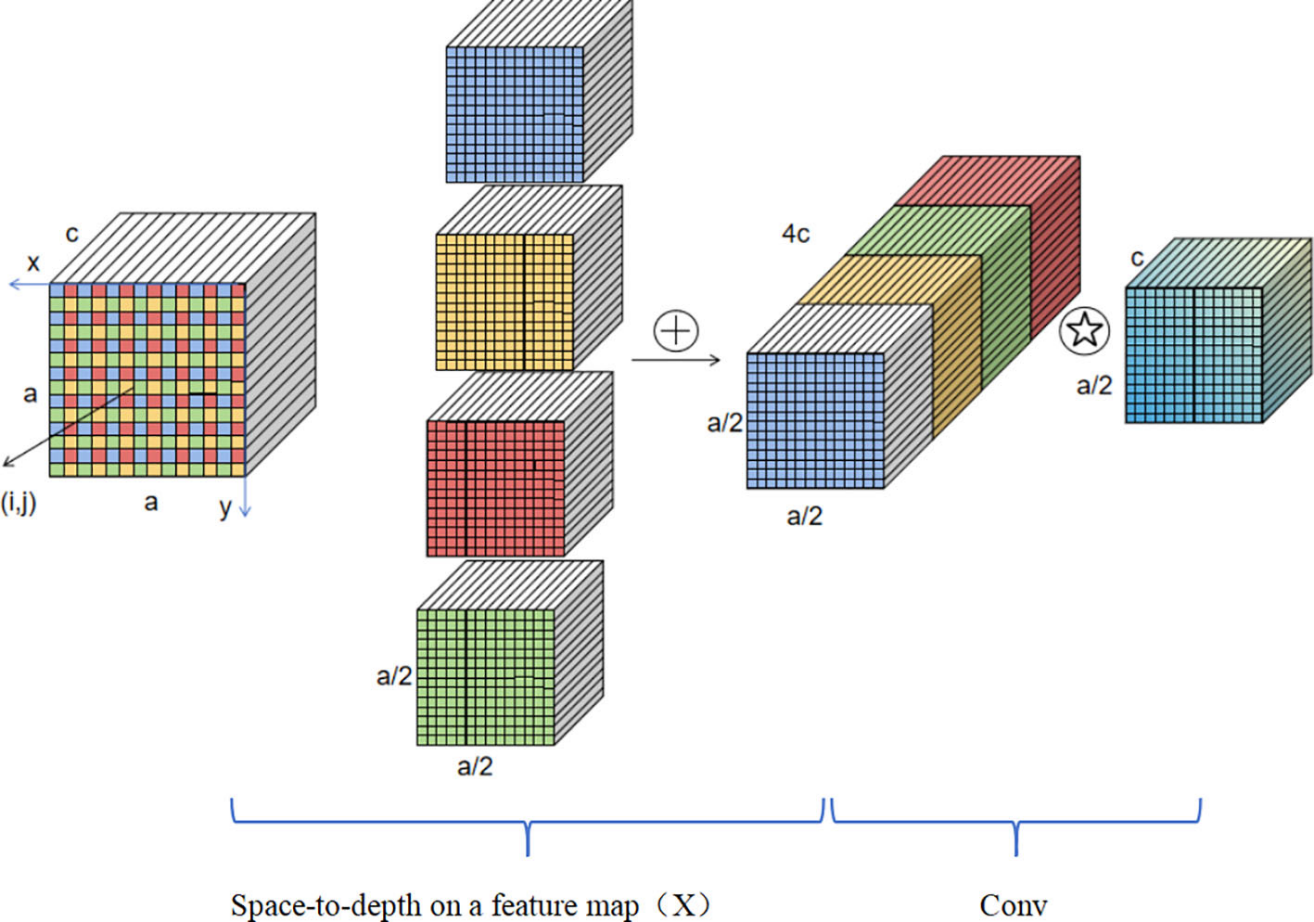
Schematic diagram of small target detection network structure.

SPD-Conv (see **Figure 8**) and the specialised segmentation head complement each other: the former performs lossless downsampling to preserve edge details of small targets, while the latter densely extracts features to enhance the semantic representation of low-resolution targets. This overcomes the feature dilution issue of small targets in traditional YOLOv8, enabling precise segmentation of weak rice seedlings during the seedling stage and meeting the practical standards for agricultural phenotyping detection.

#### 2.3.4 Visualization design of the segment module

**Figure 9:**
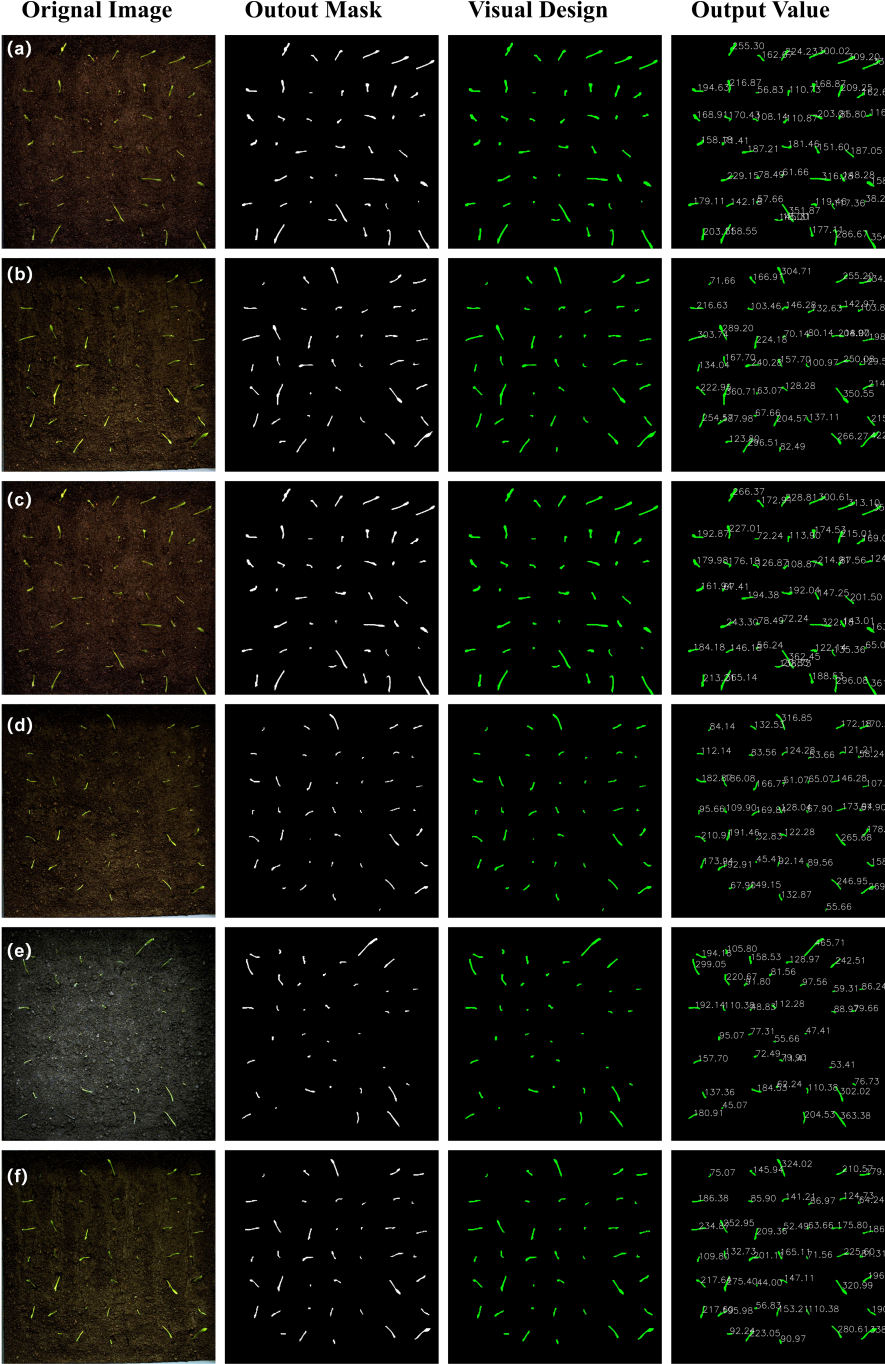
**(a–f)** Schematic diagram of visualization design output. Unit: cm.

Following the completion of the radicle detection process, the subsequent segmentation task (see **Figure 9**) is implemented in accordance with the following procedure. Initially, the mask data generated by the segmentation model is read in order to identify potential radicle regions within the image. Thereafter, an empty mask of the same dimensions as the original image is created to store the subsequent merged radicle region information. Subsequently, each mask is traversed, converted to the Uint8 type, resized, and then merged by addition. Given the potential for multiple radicle regions to overlap, the pixel values of the merged mask may exceed 255. Consequently, threshold processing is necessary to ensure that the pixel values are within the [0, 255] range. It is noteworthy that VanillaNet-YOLOv8 Segment facilitates direct output of binary maps comprising solely 0 and 1, thereby markedly enhancing the standardisation and usability of the model’s output values.

It is important to note that the number of pixel points in the mask perimeter cannot be obtained by only outputting binary maps. Consequently, a visualization design was incorporated into the original model’s output module. Firstly, it is necessary to import the requisite libraries, namely Torch, CV2 and Numpy. The best.pt is loaded using the torch.load() function, and the image is read using the cv2.imread() function. The image size is then adjusted to correspond with the dimensions specified by the model, subsequently converted into a tensor, and finally normalised. Subsequently, inference is performed, and the model is propagated forward in order to obtain the output mask. The cv2.Canny function is utilised to extract the edges of the binary mask, thus obtaining the contour coordinate list. The cv2.arcLength function is then employed to calculate the pixel perimeter of the contour. Finally, the matplotlib.pyplot function is used to draw text at the corresponding position in the original image. During the process of mask merging, the precision of data type conversion is strictly controlled. The original floating-point mask (0-1 probability values) is converted to a binary matrix through the process of (mask > 0.5).astype(np.uint8) in order to avoid edge errors caused by threshold blurring. Furthermore, np.clip is utilised to suppress pixel value overflow during multi-instance accumulation, thereby ensuring the accuracy of subsequent Canny edge detection.

This visualization design successfully implements full-process automation, encompassing instance segmentation and perimeter pixel quantification, thereby providing high-precision raw data for the Real Value Module. The experimental results demonstrate that the processing time for a single image is ≤120 milliseconds, thereby satisfying the real-time requirements of high-throughput phenotype detection.

#### 2.3.5 Real value module

**Figure 10:**
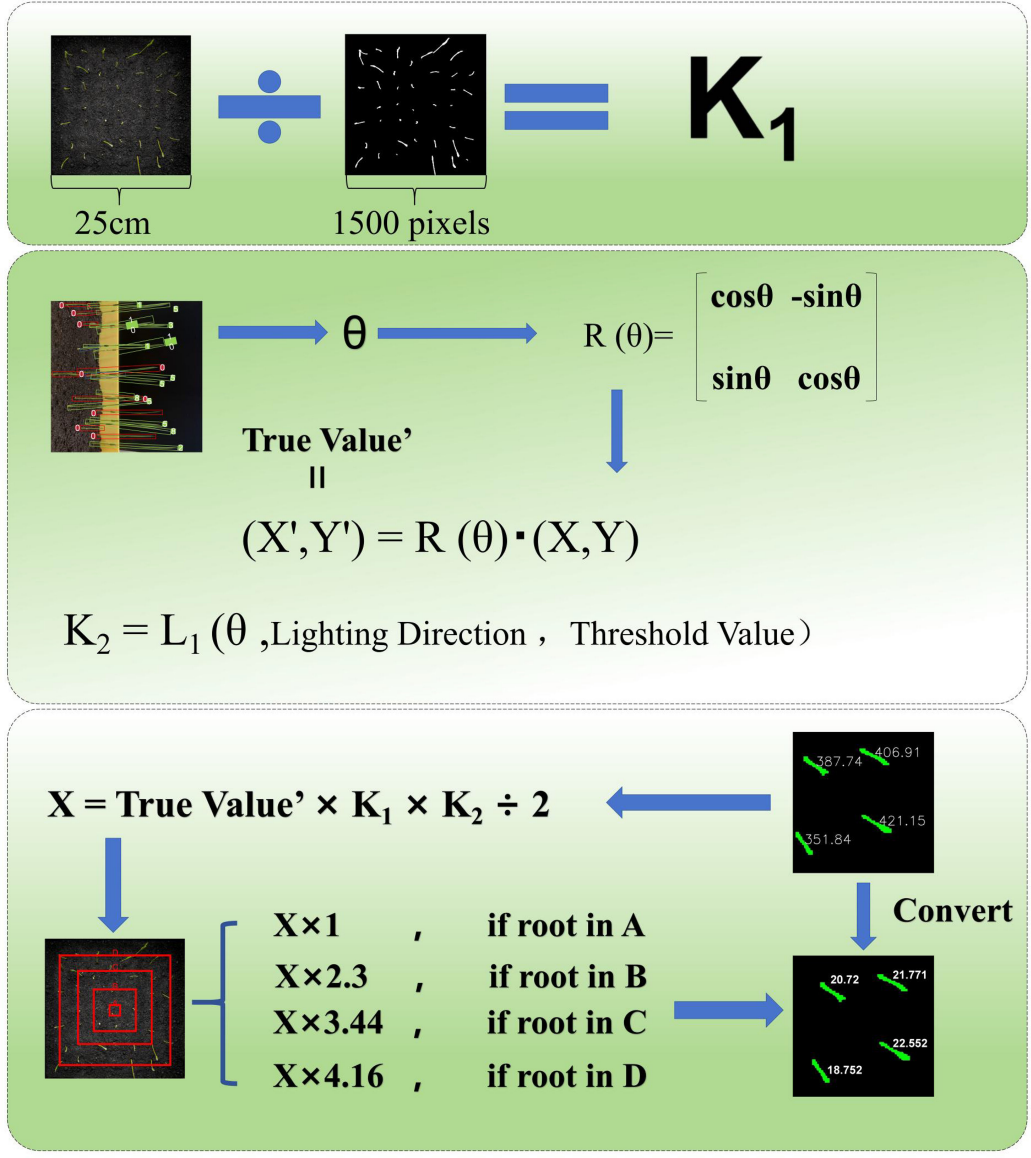
True value output formula.

The study’s objective is to address the measurement deviation caused by lens distortion from top-down shooting of the RGB camera and the phototropic growth of rice seedlings. To this end, the Real Value Module has been designed, which achieves high-precision quantification of rice seedling length through lens distortion correction, phototropic posture alignment, and multi-source constraint fusion (see **Figure 10**).

In the crop seedling full-time series detection system, the RGB camera is positioned at the top of the incubator, enabling the acquisition of top-down images of rice seedlings. The fixed-focus RGB camera utilised in the experimental setup exhibited radial pincushion distortion, characterised by the outward spreading of edge pixels. This distortion resulted in an overestimation of the measured perimeter of seedlings located farther from the image’s centre. This phototropic growth phenomenon results in a discrepancy between the output value of the VanillaNet-YOLOv8 Segment model and the true value. The Real Value Module has been developed to address the issues of lens distortion during RGB camera shooting and the phototropic tilting growth of rice seedlings at the seedling stage. The objective of the Real Value Module is to obtain the true value of rice seedling length for more accurate experimental results.

**Figure 11:**
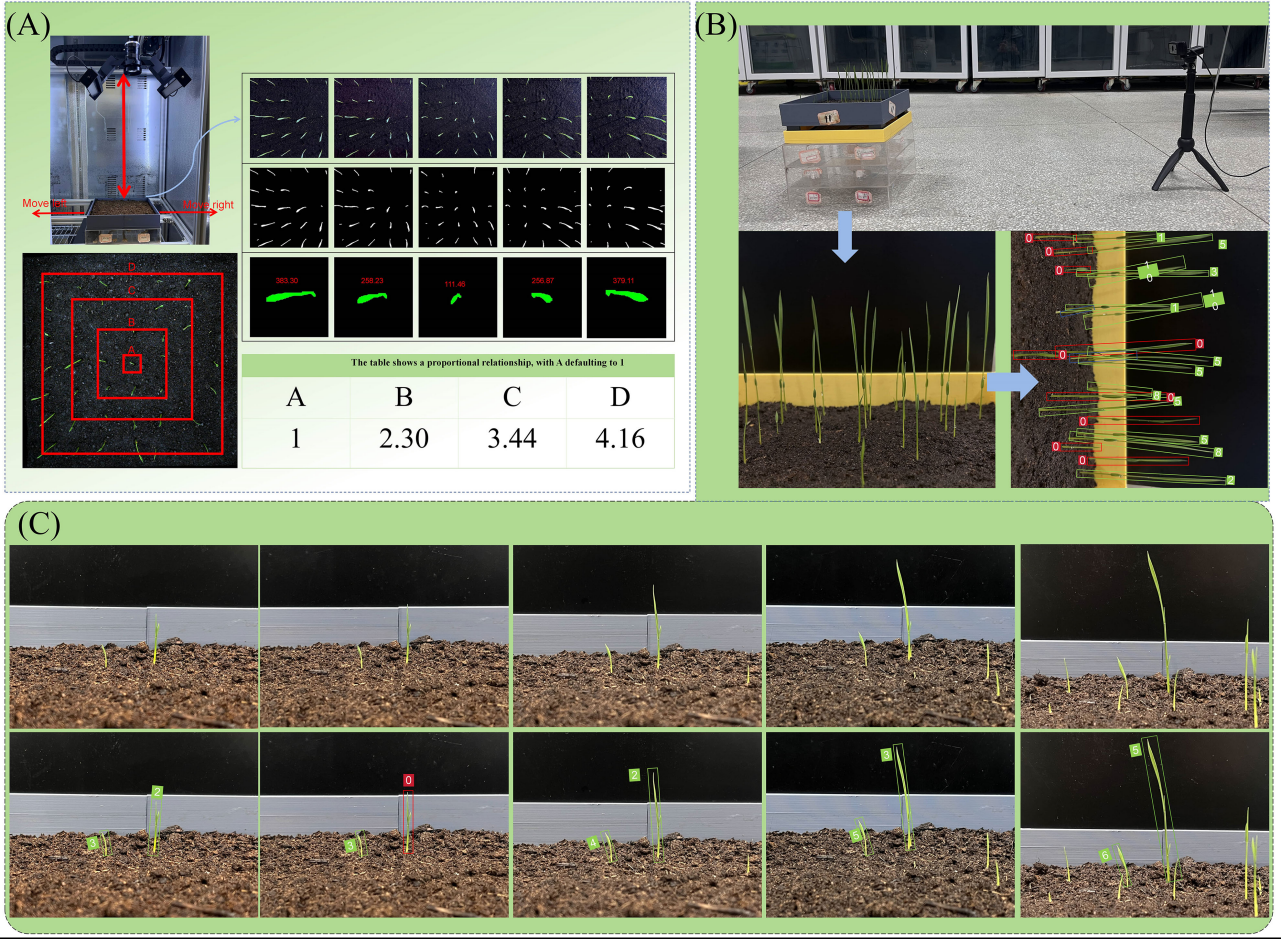
**(A)** Schematic diagram of lens distortion correction; **(B)** Schematic diagram of phototropism correction; **(C)** Schematic diagram of individual pose detection by YOLO-OBB.

RGB lens distortion correction is a crucial task in computer vision and image processing, primarily used to rectify the geometric distortion in images caused by optical defects in the lens. Lens distortions are mainly classified into two categories: radial distortion and tangential distortion. Through investigation, we determined that the lens distortion in this experiment belongs to pincushion distortion, a type of radial distortion, where edge pixels spread outward. We captured 20 groups (a total of 140 images) of the seedling box at different angles using the central seedling as the calibration point, with partial results shown in **Figure 11**. Using the model file output by VanillaNet-YOLOv8 Segment, we re-predicted these 20 groups of images and obtained the mask perimeters. We then stratified the seedlings based on their distance from the calibration point and found a stable proportional relationship between the outer and inner layers. By incorporating this proportional relationship into the correction process, we achieved the goal of correcting lens distortion.

During the growth phase of rice, the stems and leaves exhibit a bending tendency towards the direction of light, thereby demonstrating a fixed-direction tilt. In order to address the tilting problem of rice seedlings caused by phototropism, correction was performed in combination with YOLO-OBB. The utilisation of the seedling full-time series detection system facilitated the cultivation of rice seeds to the seedling stage. A mobile camera was employed to capture images of the rice front, with a black cardboard background board serving as the backdrop. The dual-vit attention mechanism was integrated into YOLO-OBB, a process that effectively mitigated the impact of rice overlap on dataset construction. A total of 245 images were captured, and following the processes of annotation, processing, and data augmentation, a rice phototropism dataset was constructed. Following the training of YOLO-OBB using this dataset, the main growth direction (θ) of each seedling was output. The seedlings were then aligned in the vertical direction through the implementation of differentiable rotation operations, with the objective of eliminating coordinate overlap caused by tilting. This was achieved specifically through the formula (X’, Y’) = R(θ)?(X, Y), where R is the rotation matrix. Concurrently, a phototropic angle smoothness constraint was introduced, denoted by [L1(θ), lighting direction], with the objective of compelling the model to discern the mapping relationship between light and angle. The measured light angles of the crop seedling full-time series detection system are 45° ± 15° northeast, 45° ± 15° northwest, 45° ± 15° southeast, and 45° ± 15° southwest. The detection threshold is dynamically adjusted according to the age of the seedling: a soft threshold of 0.4 is used during the tillering stage to retain weak seedlings, and it is increased to 0.6 during the jointing stage to filter out noise, balancing detection accuracy at different growth stages.

In the VanillaNet-YOLOv8 Segment model, the number of pixels in the characteristic border of rice seedlings has been output. The dimensions of the training image pixels are 1600×1600, and the actual length of the seedling box employed is 25cm×25cm. Given that the output pixel count is equivalent to the mask perimeter, it can be deduced that the pixel count of the rice seedling length should be equivalent to half of the irregular polygon shape formed after segmentation calculation. The true value of the rice seedling length is obtained by combining lens distortion correction and phototropism correction.

**Figure 12:**
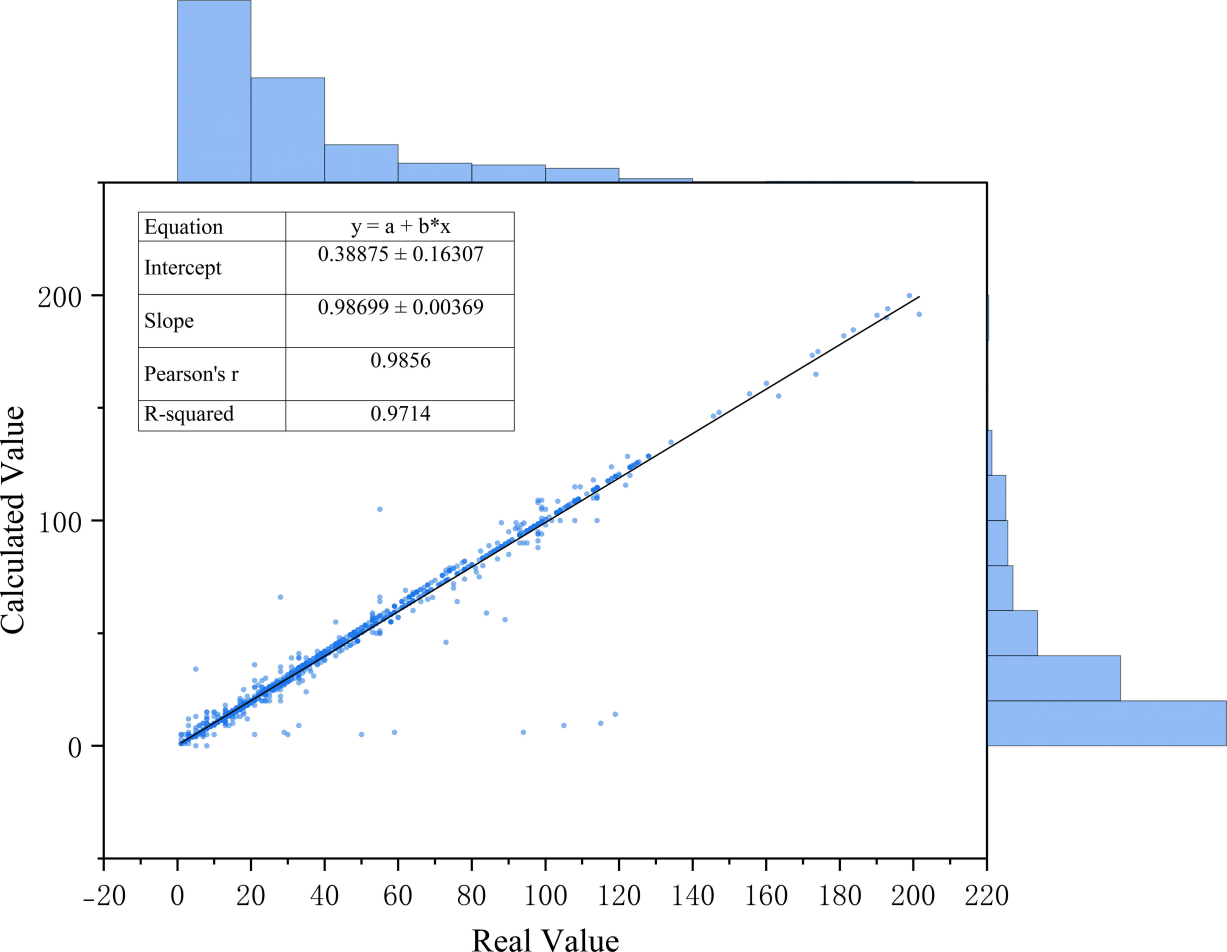
Relationship diagram between manually measured rice seedling length and output values of the true value module.

The manually measured length of the rice seedlings was represented as a scatter plot, with the true value output by the True Value module represented by a fitted line, as illustrated in **Figure 12**. The gradient of the fitting line is 0.98699, indicating a strong correlation between the manual measurement value and the module output value. The distribution of the manual measurement value and the module output value is also found to be essentially consistent, thus providing evidence of the success of the True Value module design and offering reliable phenotypic data support for the quantitative evaluation of the regulatory effect of nano-iron oxide on rice seedling growth vitality under salt stress.

### 2.4 Evaluation indicators

In the model performance evaluation system, superiority analysis is conducted from two perspectives: model lightweight performance and reliability performance. The lightweight degree of the model is quantitatively evaluated by the total number of parameters (Params) and the amount of floating-point operations (FLOPS), which are used as measures of model complexity to determine the efficiency of network design. For the rice radicle detection and segmentation tasks, average precision indicators (mAP0.5 and mAP0.5-0.95) are utilised for reliability evaluation: mAP0.5 represents the detection accuracy of the model for radicle detection boxes (box) and masks (mask) when the intersection-over-union (IOU) threshold is 0.5; mAP0.5-0.95 measures the comprehensive detection accuracy when the IOU threshold is in the range of 0.5 to 0.95. A higher mAP value is indicative of a higher precision and reliability of model detection and segmentation.


$$mAP_{0.5}=\frac{1}{n_{c}}\int_{0}^{1}p(R)dR$$

$$mAP_{0.5-0.95}=avg\;(mAP_{i}),\;i=0.5:0.05:0.95$$

$$FLOPS=2\times H\times W(C_{in}K^{2}+1)C_{out}$$

$$Params=C_{in}\times K^{2}\times C_{out}$$

Furthermore, precision (P) is defined as the proportion of true positive samples among predicted positive samples, and recall (R) is the proportion of correctly predicted true positive samples among real positive samples (Smith, 2019). In this context, H×W denotes the height×width of the input feature map, k² signifies the magnitude of the convolution kernel, and Cin and Cout represent the number of input and output channels, respectively.

## 3 Results and discussion

### 3.1 Environment and parameters

The model operates on the Windows 10 system with a processor configuration of NVIDIA A100 80GB. The model operates within a Python-3.8 framework, utilising PyTorch-2.21 and a CUDA version of 11.8. Furthermore, in accordance with the stipulated criteria for rice radicle small-scale detection and the attributes inherent in the dataset, the following hyperparameters were selected: (see **Table 2**).

**Table 2:** Parameter settings for the system and its accompanying software.

Parameters	Setup
Epoch	300
Batch size	16
NMS IoU	0.7
Image Size	640
Initial Learning Rate	1×10^-2^
Final Learning Rate	1×10^-4^
Momentum	0.937
Weight-Decay	5×10^-4^

The utilisation of the containerisation technology of the OpenBays platform facilitates the creation of a computational instance that supports CUDA acceleration. Concurrently, a Python 3.8 environment is configured, in conjunction with the PyTorch 2.21 framework. The YOLOv8 open-source code repository is then cloned into the computational container via the Git protocol, ensuring version consistency. The pre-annotated compressed package of the rice seedling dataset is mounted into the computational container using the S3 protocol. Subsequent to the process of decompression, the data is stored in the mydata directory in conjunction with the VOC.yaml file, thereby establishing a conventional YOLO dataset structure.

### 3.2 Ablation experiments

In order to verify the impact of each improved module on model performance, ablation experiments were carried out based on YOLOv8n-seg, whilst ensuring that other configurations and hyperparameters remained consistent. The findings of the aforementioned experiments are displayed in **Table 3**.

**Table 3:** Results of ablation experiments.

Model	Params (M)	FLOPs (G)	mAP^box^_50_ (%)	mAP^box^_50-95_ (%)	mAP^mask^_50_ (%)	mAP^mask^_50-95_ (%)
YOLOv8n-seg	**3.2**	**12**	95.2	70.2	79.8	40.0
YOLOv8-seg -VanillaNet	3.01	8.2	94.8	70.1	78.8	40.2
YOLOv8-small target	3.64	65.2	97.7	75.7	87.3	47.5
Dualvit-yolov8-seg	3.48	10.2	95.6	70.7	80.0	41.6
Small target and Dualvit	3.71	67.6	98.2	78.0	96.1	59.8
VanillaNet-YOLOv8 Segment	4.5	13.1	**98.4**	**78.2**	**96.4**	**60.0**

The bolded words indicate the best performance.

**Figure 13:**
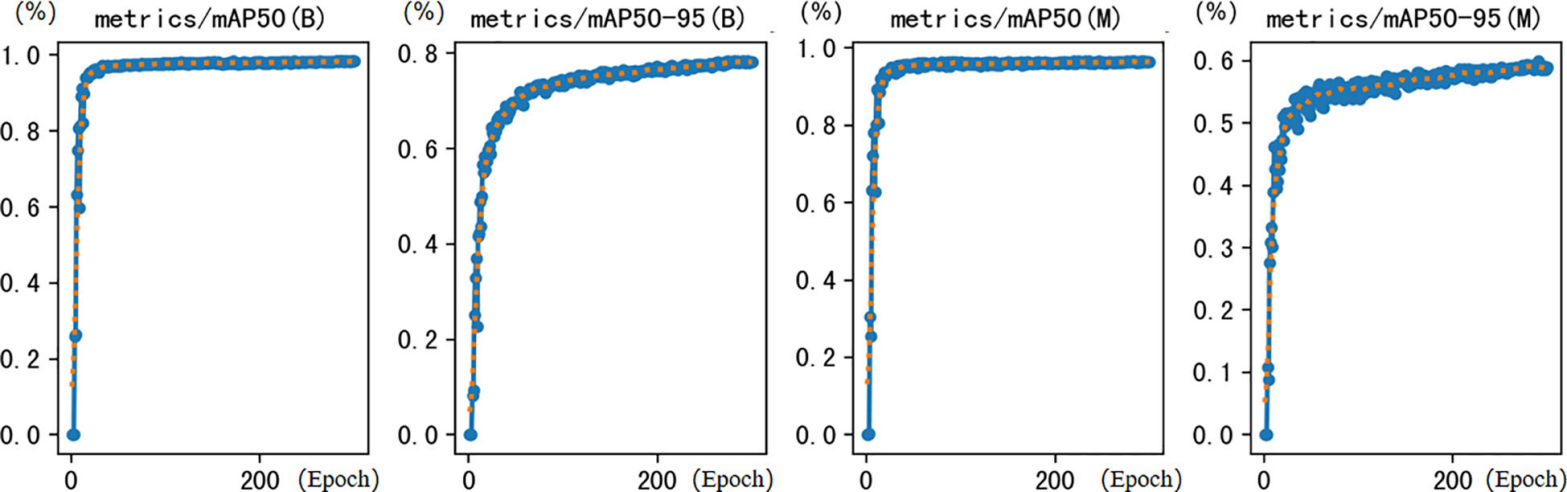
Performance diagram of VanillaNet-YOLOv8 segment.

As shown in the table, when replacing the original backbone network with VanillaNet (YOLOv8-seg-VanillaNet), the model parameters (Params) decreased from 3.2M to 3.01M, a decrease of 13.5%; the computational amount (FLOPs) decreased from 12G to 8.2G, a decrease of 49.38%, achieving significant lightweighting. Although the target detection and segmentation accuracy only slightly decreased, it indicates that the concise structure of VanillaNet effectively reduces model complexity while maintaining high performance. By introducing the SPD-Conv layer and adding a dedicated detection head, the small target design increased the mAP50 of target detection accuracy by 2.5% and mAP50-100 by 5.5%; the mAP50 of segmentation accuracy increased by 7.5%, and mAP50-100 increased by 7.5%. It can be seen that the small target design has a very significant improvement in small target detection accuracy. However, the FLOPs increased sharply by 443.4% (from 12G to 65.2G), reflecting the high demand of this module for computational resources. The change in model complexity was minimal, and the detection and segmentation accuracy only slightly increased. Its core value lies in solving the problem of seedling overlap caused by phototropism and improving the robustness of instance segmentation in complex scenarios. When the small target detection module and DualVit were used together, the detection accuracy (mAPbox50/mAPbox50-95) further increased to 98.2% and 78.0%, and the segmentation accuracy (mAPmask50/mAPmask50-95) reached 96.1% and 59.8%, which were close to the performance of the final model VanillaNet-YOLOv8 Segment (mAPbox50 = 98.4%, mAPmask50 = 96.4%), verifying the collaborative optimization effect of DualVit on small target detection. After integrating these modules, VanillaNet-YOLOv8 Segment can achieve a significant improvement in accuracy performance while maintaining the original level of model complexity, reflecting its effectiveness for small target detection at the rice seedling stage. **Figure 13** shows the performance diagram of VanillaNet-YOLOv8.

Segment.

### 3.3 Comparative experiments of different detection models

In order to demonstrate the superiority of the VanillaNet-YOLOv8 Segment model, comparative experiments were conducted. The experiments involved selecting YOLOv5-seg, YOLOv8-seg, YOLOv9e-seg, YOLOv11-seg, and VanillaNet-YOLOv8 Segment, and the configuration environment and hyperparameter settings were kept the same for all models. The results of the study are presented in **Table 4**. As demonstrated in **Figure 1**, the YOLOv5-seg model is the most lightweight, yet its rice segmentation accuracy is significantly inferior to that of competing models. A comparative analysis of the VanillaNet-YOLOv8 Segment with the YOLOv5-seg, YOLOv8-seg, and YOLOv11-seg reveals that they are equivalent in terms of parameters and FLOPs. However, the target detection mAP50 of the VanillaNet-YOLOv8 Segment achieves 98.4%, which is 3.4% higher than that of the YOLOv5-seg. The mAP50-95 metric was found to be 78.2%, representing an 8% increase over YOLOv5-seg, a 4.7% increase over YOLOv8-seg, and an 8.3% increase over YOLOv11-seg. In terms of segmentation accuracy, the mAP50 of VanillaNet-YOLOv8 is 35.1% higher than that of YOLOv5-seg, 16.6% higher than that of YOLOv8-seg, and 15.8% higher than that of YOLOv11-seg. Furthermore, mAP50-95 reaches 60%, which is 44.9% higher than that of YOLOv5-seg, 20% higher than that of YOLOv8-seg, and 18.3% higher than that of YOLOv11-seg. This finding suggests that the model demonstrates a high degree of robustness when it comes to multi-scale target detection and pixel-level segmentation of small rice seedling images. The parameters and FLOPs of VanillaNet-YOLOv8 Segment are only 15% and 8% of those of YOLOv9e-Seg, respectively. However, the former is significantly more accurate, which is indicative of an efficient model design. This has resulted in a substantial enhancement in detection and segmentation performance, whilst only exhibiting a minor increase in computational resource consumption. This provides feasibility for the embedded deployment of agricultural phenotype detection.

**Table 4:** Results of comparison experiments.

Model	Params (M)	FLOPs (G)	mAP^box^_50_ (%)	mAP^box^_50-95_ (%)	mAP^mask^_50_ (%)	mAP^mask^_50-95_ (%)
YOLOv5-seg	**2.5**	**7.2**	95.0	70.2	61.3	15.1
YOLOv8n-seg	3.2	12	95.2	70.2	79.8	40.0
YOLOv9e-seg	27.6	157.6	95.1	73.5	80.0	42.6
YOLOv11-seg	2.83	10.2	94.7	69.9	80.6	41.7
VanillaNet-YOLOv8 Segment	4.5	13.1	**98.4**	**78.2**	**96.4**	**60.0**

The bolded words indicate the best performance.

### 3.4 Monitoring the impact of nano-iron oxide on rice growth vitality under salt stress

The experimental design comprised 36 groups of control experiments, encompassing six nano-iron oxide concentrations (0, 20, 50, 100, 200, 300 mg/L) and six salt stress levels (0, 30, 60, 90, 120, 150 mmol/L). The high-throughput phenotype detection system was utilised to obtain a series of 90-hour growth images of 640 rice seedlings. Following semantic segmentation and correction by the True Value module, a dataset of true seedling length values containing 28,224 time series was established. The investigation revealed divergent growth trends of rice seedlings under varied conditions. In order to further explore the impact of the combined action of nano-iron oxide and salt stress on rice growth vitality, the relationship between growth height and time was obtained, and the growth vitality of rice was evaluated using quantitative standards of average value and change trend. In addition, a “static vitality-dynamic vitality” dual-index evaluation system was constructed in order to reveal the interaction effect of nano-iron oxide and salt stress during rice growth.

The figure presents histograms illustrating the change in rice seedling length over time under various salt stress conditions. The control group (CK) is compared with five concentrations of nano-iron oxide. Each colour in the spectrum corresponds to a specific nano-iron oxide concentration. In order to more intuitively characterize the growth vitality of rice, we performed linear fitting on the full-time series growth lengths of rice seedlings under each nano-iron oxide condition. The growth vitality can be comprehensively evaluated by combining the slope of the fitting line and the average seedling length.

**Figure 14:**
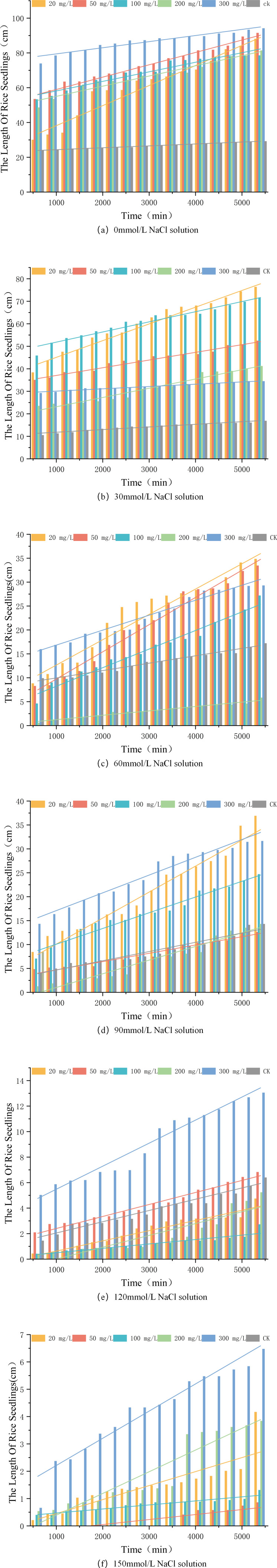
**(a–f)** Relationship diagram of rice seedling length changes over time under different conditions.

As demonstrated in **Figure 14**, the rice seedling length typically exhibits an upward trend over time. In comparison with the CK group, it was determined that only the 300 mg/L nano-iron oxide concentration exhibited a promoting effect on rice growth under all salt stress conditions. The 300 mg/L nano-iron oxide solution demonstrated a marked effect on seedling length, as evidenced by the significant increase in histogram height and linear fitting slope (with the exception of 30 mmol/L NaCl stress). This finding suggests that the 300 mg/L nano-iron oxide solution exerts the optimal stimulatory effect on rice growth. The 50 mg/L nano-iron oxide exhibited only a marginal inhibitory effect on rice growth in a 90 mmol/L NaCl solution, yet the growth rate of rice seedlings was notably elevated under its influence, surpassing that of the 300 mg/L nano-iron oxide solution under certain salt stress conditions. The 20 mg/L and 100 mg/L nano-iron oxide solutions both inhibited rice growth under 120 mmol/L salt stress, yet exhibited a promoting effect under 30 mmol/L, 60 mmol/L, 90 mmol/L, and 150 mmol/L salt stress. The growth rate of rice under the 20 mg/L nano-iron oxide solution was the highest in the CK group, at 30 mmol/L and 90 mmol/L. The 200 mg/L nano-iron oxide solution exhibited an inhibitory effect under 60 mmol/L and 90 mmol/L salt stress but still had a promoting effect at other concentrations, indicating that this concentration may have a stress response threshold. It is evident that under conditions of severe salt stress (150 mmol/L NaCl solution), rice seedlings in the CK group exhibited no growth, indicative of a substantial inhibitory effect of salt stress on rice seedling growth. However, rice seedlings in the other five groups treated with nano-iron oxide solutions demonstrated varying degrees of growth, suggesting that nano-iron oxide solutions can promote rice growth under severe salt stress conditions.

**Figure 15:**
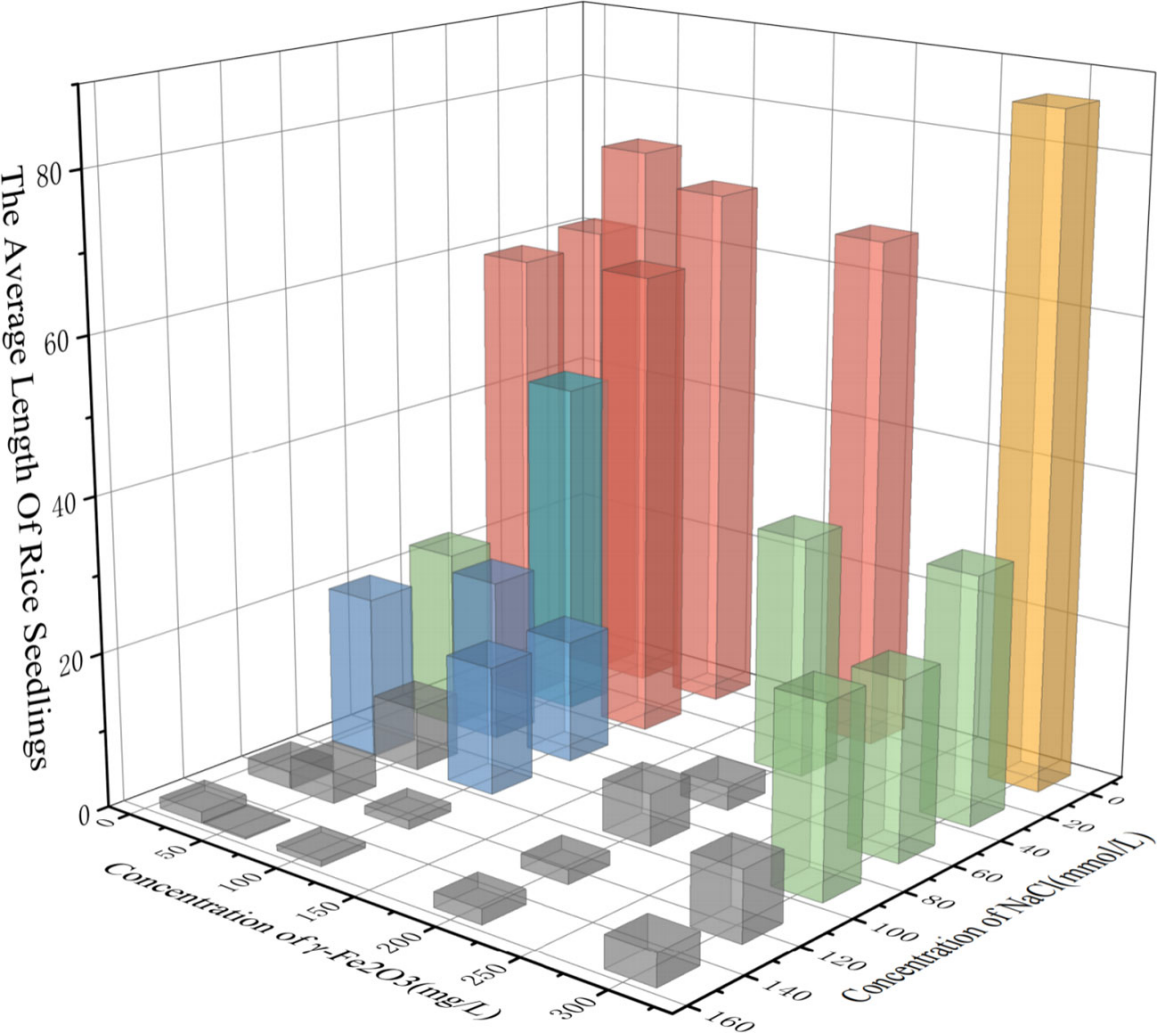
Histogram of average rice seedling length under various conditions.

As illustrated in **Figure 15**, the histogram depicts the mean length of rice seedlings across 36 distinct groups. As demonstrated in the accompanying figure, the plant growth exhibited a direct correlation with the reduced sodium chloride solution content. The most significant growth was observed in the rice growth group, in which 300 mg/L of nano-iron oxide was administered under conditions of no salt stress. In the context of the same salt stress condition, varying concentrations of nano-iron oxide have been observed to generally demonstrate a rice growth trend of low in the middle and high on both sides. In order to comprehensively evaluate the growth vitality of rice based on the average seedling length and growth trend of rice, the height of the rectangular body in the figure is employed to represent the average seedling length of rice under different conditions. This reflects the uniformity of current population growth and basic robustness. This is an intuitive manifestation of the photosynthetic area and material accumulation, and is called static vitality. In a similar manner, the fitting slope depicted in the figure is employed to symbolise the growth rate of rice under varying conditions. This slope reflects the growth increment per unit time and the activity degree of cell division and elongation. The activity degree is a pivotal indicator for evaluating growth potential, stress resistance, and the progression of the growth period. This activity degree is termed dynamic vitality. The seedling stage is characterised by the rapid differentiation of roots and stems and leaves. The growth speed of the seedling is directly related to its rooting ability, tillering potential, and subsequent yield formation. It is more significantly affected by salt stress. The average seedling length, as a basic growth index, supplements the growth speed to avoid misjudgement of “leggy seedlings” (such as thin and weak seedlings) caused by a single-minded focus on speed.

In order to comprehensively evaluate the growth vitality of rice, a quantitative evaluation system was established. This incorporated static vitality, characterised by average seedling length to reflect population growth uniformity, and dynamic vitality, characterised by the slope of the growth rate to reflect growth increment per unit time. The comprehensive evaluation value was calculated through the application of a standardised procedure involving the normalisation processing (the division of growth rate and average seedling length by their respective maximum values under equivalent conditions) and equal weight allocation (0.5 for each indicator).The results obtained demonstrated that 300 mg/L nano-iron oxide exhibited the most significant promoting effect on rice growth under salt stress, with its comprehensive evaluation value accounting for 23.8% of the total. The 20 mg/L treatment group followed closely with 22.7%, while the 50 mg/L and 100 mg/L groups accounted for 16.2% and 15.6% respectively. Despite the 200 mg/L treatment group demonstrating the least comprehensive performance among all concentrations (13.6%), it nevertheless exhibited a significant enhancement in comparison to the CK group devoid of nanomaterials (8.1%). The findings indicate that nanomaterials generally alleviate salt stress, with significant differences in promotion effects across concentrations: the synergistic effect was most prominent at 300 mg/L, followed by 20 mg/L, while the promotion effect at 200 mg/L was relatively weak but still better than the control. This quantitative result provides data support for the precise application of nano-iron oxide in salt-resistant rice cultivation.

## 4 Conclusion, limitations and future work

The present study proposes a technical system of “nanoparticles priming-full-time series phenotype acquisition-improved YOLOv8 segmentation model-vitality quantification analysis” for the purpose of evaluating the impact of nano-iron oxide on the growth and vitality of rice seedlings under salt stress. An independently developed high-throughput crop seedling phenotype detection system was utilised to achieve continuous monitoring of rice seedlings over a period of 90 hours, resulting in the accumulation of 3,888 full-time series growth images. This approach effectively addressed the technical challenges associated with traditional phenotype analysis, such as prolonged analysis time, low efficiency, and subjectivity. A high-precision annotated dataset was constructed through a combination of manual fine annotation of 640 sample images and the preprocessing of motion-blurred images with a super-resolution algorithm (SSN). The VanillaNet-YOLOv8 Segment improved model was proposed as a solution to the problem of small target detection of rice seedlings. This model employs a number of techniques to enhance the efficiency of the convolutional layers, including parameter compression and the reduction of inference delay. It achieves these objectives by replacing the original backbone network with the concise structure of VanillaNet, thereby improving the accuracy of recognition. Concurrently, DualVit is introduced to fuse global semantics (seedling shape) and local features (seedling colour), thereby enhancing precision and resolving the issue of seedling overlap caused by phototropism. The integration of a small target detection module represents a significant advancement, as it overcomes the recognition limitations of YOLOv8 Segment for diminutive targets. This is achieved by incorporating a dedicated detection head for small objects and aligning it with SPD-Conv. The model has been developed to output the number of pixel points in the mask perimeter and to obtain the true value of rice seedling length through lens distortion correction and phototropism correction. This has been achieved through segment visualization design and the Real Value Module.

The VanillaNet-YOLOv8 Segment model demonstrates a target detection accuracy of mAP50 reaching 98.4% and mAP50-95 reaching 78.2%; the segmentation accuracy mAP50 reaches 96.4%, and mAP50-95 reaches 60.0%. In comparison with the conventional YOLOv8n-seg model, the target detection accuracy mAP50 and mAP50-95 exhibited an enhancement of 3.2% and 8.0%, respectively, while the segmentation accuracy mAP50 and mAP50-95 demonstrated an augmentation of 16.6% and 20%, respectively. Notwithstanding the attainment of such a substantial precision breakthrough, the model’s complexity remains commensurate with that of the unimproved YOLOv8n-seg. The True Value module facilitates the entire model’s capacity to generate the true values necessary for subsequent observation of the impact of nano-iron oxide on rice growth vitality under salt stress conditions. The correlation coefficient between manual measurement and the machine learning values output by the True Value module is 0.98699, indicating a high degree of reliability in the true seedling length values output by the True Value module.

In this study, the effects of nano-iron oxide (0–300 mg/L) under salt stress (0–150 mmol/L) on seedling length and growth rate were systematically analysed. The experimental results demonstrated that an increase in NaCl concentration resulted in a decline in the growth vitality of rice seedlings, thereby substantiating the assertion that saline environments significantly inhibit seedling development. However, nano-iron oxide has been shown to mitigate the deleterious effects of salt stress on rice, thereby promoting its growth and vitality under salt-free conditions. It is noteworthy that varying concentrations of nano-iron oxide exhibited disparate effects on the growth vitality of rice plants under diverse salt stress conditions.In general, the 300 mg/L nano-iron oxide solution demonstrated superiority in enhancing the growth rate of rice and increasing the average length of seedlings. The 20 mg/L solution was closely followed, demonstrating strong growth-promoting effects under low salt concentrations (≤60 mmol/L) but exhibiting slight inhibition of growth vitality at the high salt concentration of 120 mmol/L. The 100 mg/L concentration demonstrated marginally inferior performance in comparison to the 50 mg/L concentration, while the 200 mg/L solution exhibited the least efficacious properties in promoting growth vitality. Notably, this solution even exerted an inhibitory effect on rice growth at salt concentrations of 60 and 90 mmol/L. It is noteworthy that all five gradient concentrations of nano-iron oxide promoted rice growth under the severe salt stress of 150 mmol/L, thus underscoring the potential application of nanomaterials in highly saline environments.

The present study is encumbered by two technical impediments. Firstly, the collection of phenotype data is constrained by the limitation of the two-dimensional top-down shooting perspective, which results in the loss of three-dimensional morphological information regarding upright seedlings. This, in turn, has a deleterious effect on the reliability of the deep learning model’s semantic segmentation of seedling spatial structures. Secondly, the presence of complex background interference is a further hindrance. The similar grey-scale features of seedlings and small soil blocks in the soil environment can lead to the misdetection of early seedlings.

In the future, we will continue to optimise our model, with a view to further improving its accuracy and reducing its complexity. Concurrently, we will introduce 3D point cloud and multispectral imaging technologies to construct a three-dimensional phenotype dataset containing parameters such as height and curvature, thus enhancing the recognition accuracy of seedlings in complex environments. It is further proposed that the applicability of VanillaNet-YOLOv8 Segment in the analysis of multi-phenotype parameters, such as leaf area measurement and disease spot detection, will be explored. In addition, the development of cross-crop universal morphological detection modules will be undertaken, and technical support will be provided for intelligent breeding and precision agriculture. The phenotype detection and model analysis system constructed in this study provides a reusable technical framework for the evaluation of nanomaterial agricultural applications and the screening of salt-tolerant crops. The relevant results are expected to promote the automation and precision of plant phenomics research.

## Data Availability

The datasets presented in this study can be found in online repositories. The names of the repository/repositories and accession number(s) can be found in the article/**Supplementary Material**.
