# Supplementary figures and images for: VanillaNet-YOLOv8 segment: detection of nano-iron oxide regulation on rice seedling growth vitality under salt stress (part 1 of 5)
Source: Front Plant Sci. 2025 Sep 17;16:1631279. doi: 10.3389/fpls.2025.1631279 (PMC12484053; doi:10.3389/fpls.2025.1631279)

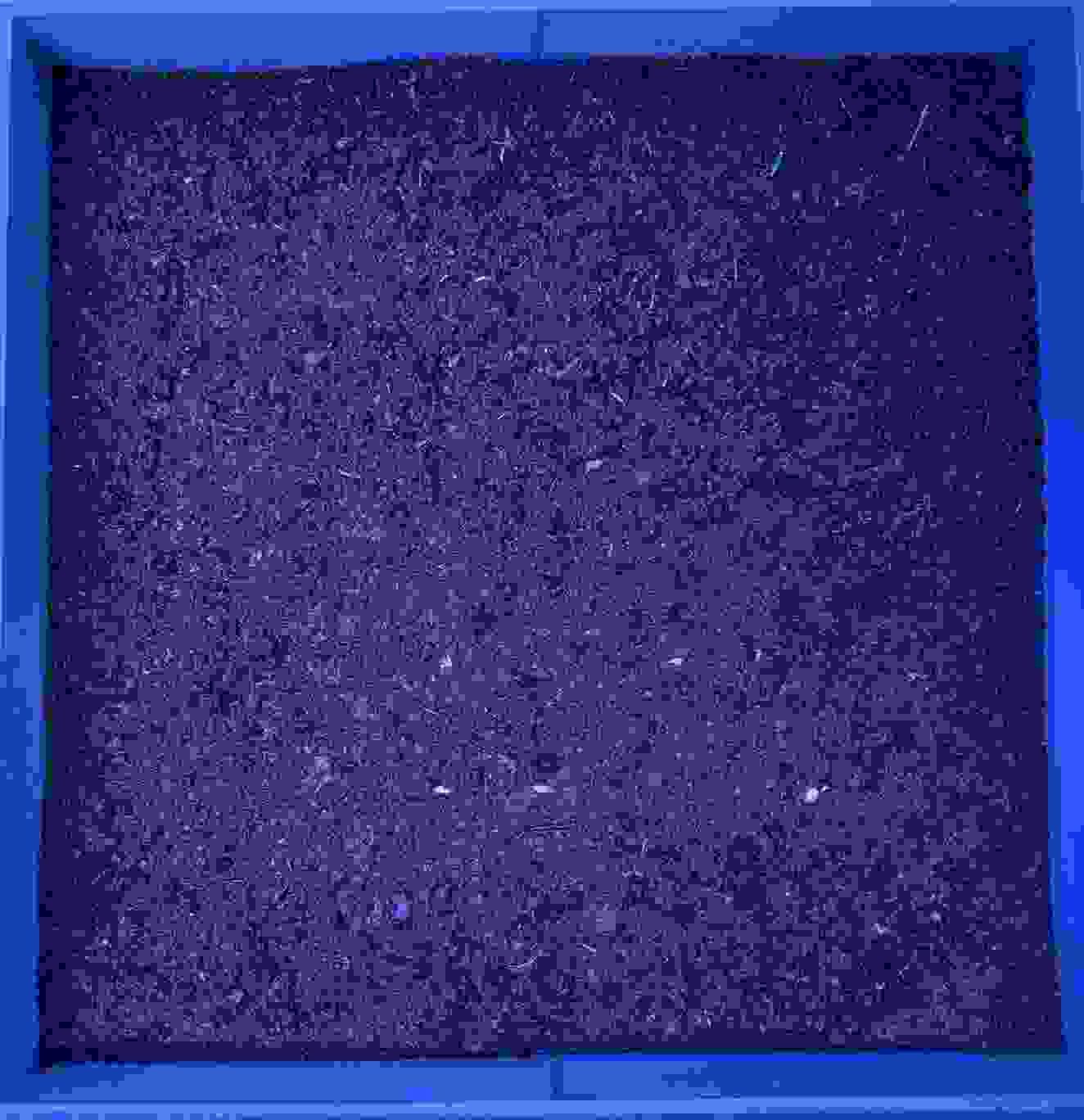

Supplement: Supplementary file 1 [file DataSheet1.zip › val/1-5.JPG]

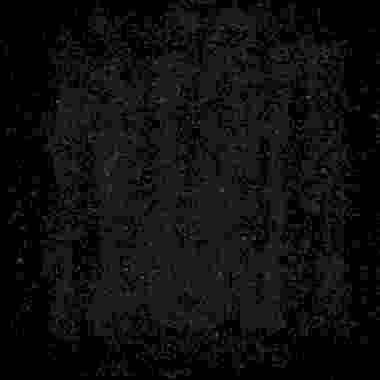

Supplement: Supplementary file 1 [file DataSheet1.zip › val/100150-2024-3-19-12-40-5.JPG]

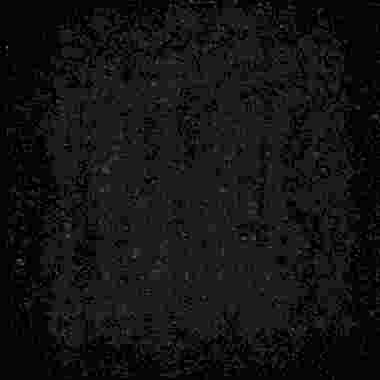

Supplement: Supplementary file 1 [file DataSheet1.zip › val/100150-2024-3-20-3-56-31.JPG]

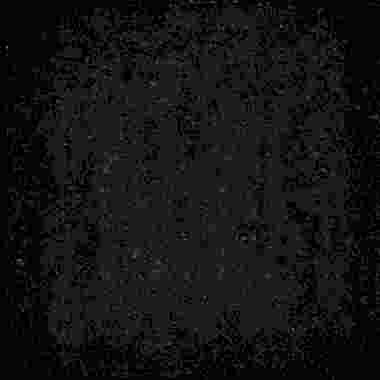

Supplement: Supplementary file 1 [file DataSheet1.zip › val/100150-2024-3-20-6-28-52.JPG]

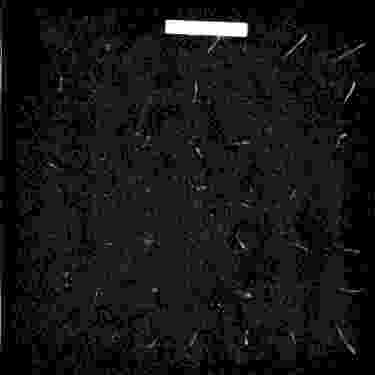

Supplement: Supplementary file 1 [file DataSheet1.zip › val/10030-2024-3-19-17-23-47.JPG]

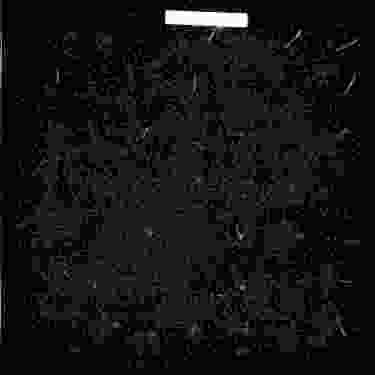

Supplement: Supplementary file 1 [file DataSheet1.zip › val/10030-2024-3-19-2-6-14.JPG]

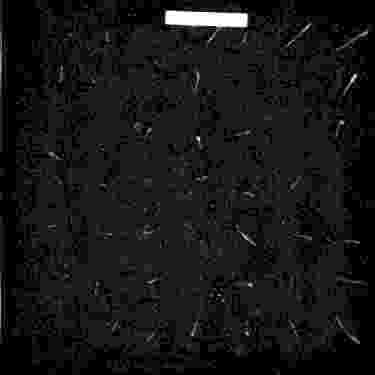

Supplement: Supplementary file 1 [file DataSheet1.zip › val/10030-2024-3-20-3-35-13.JPG]

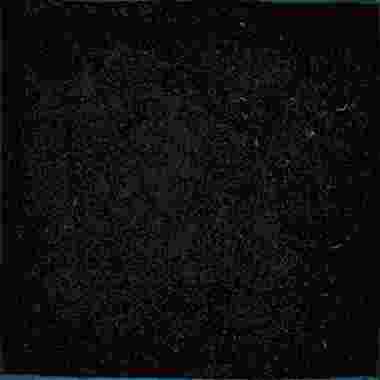

Supplement: Supplementary file 1 [file DataSheet1.zip › val/10090-2024-3-18-20-59-54.JPG]

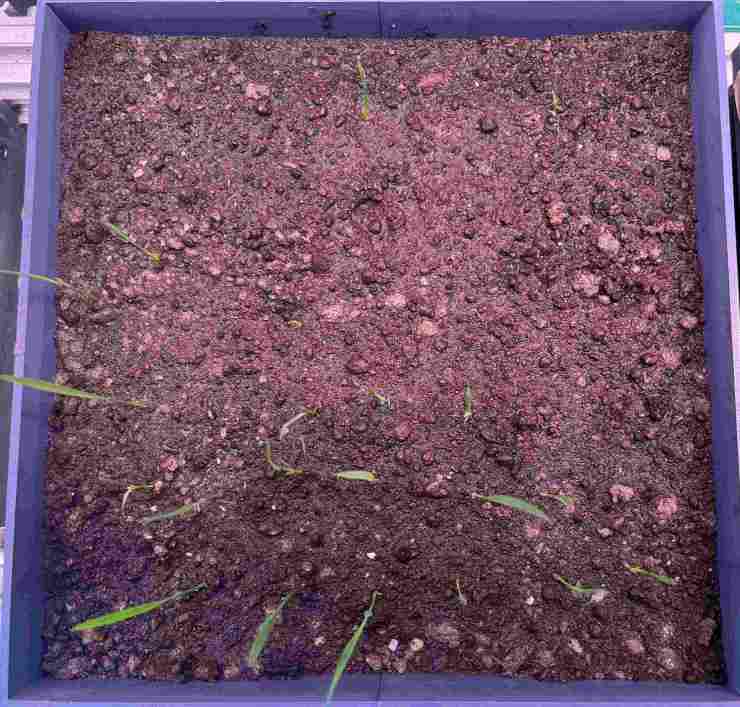

Supplement: Supplementary file 1 [file DataSheet1.zip › val/12-2.JPG]

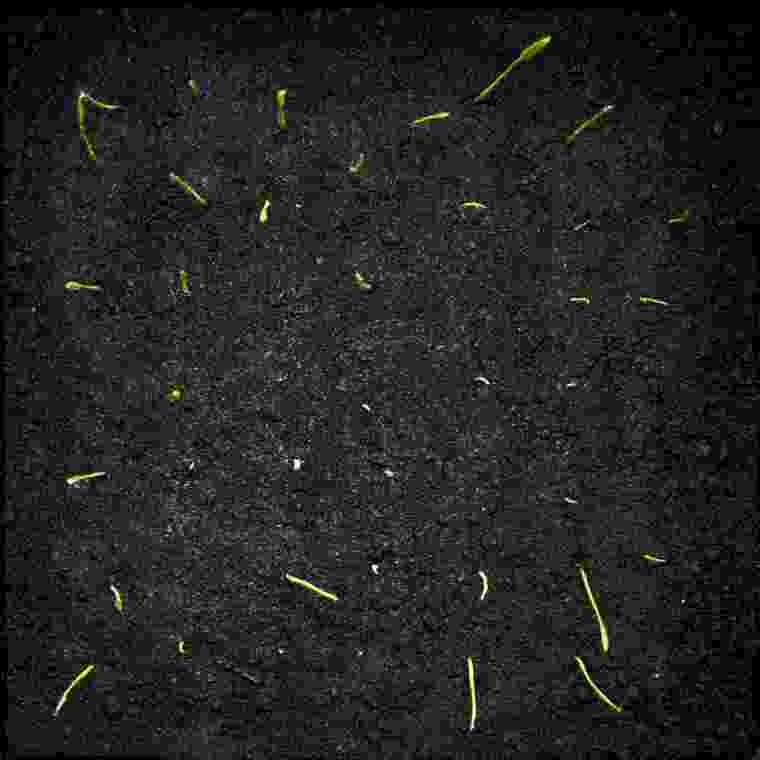

Supplement: Supplementary file 1 [file DataSheet1.zip › val/200-2024-3-19-11-56-12.JPG]

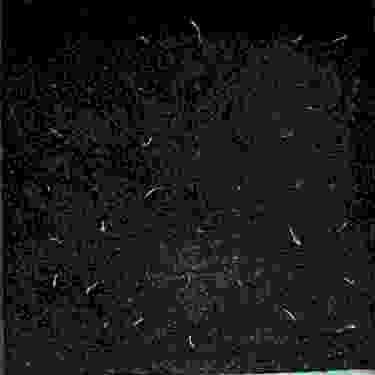

Supplement: Supplementary file 1 [file DataSheet1.zip › val/2000-2024-3-18-15-47-54.JPG]

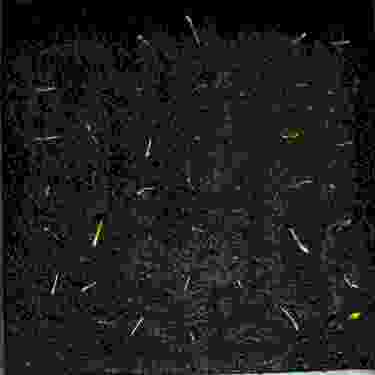

Supplement: Supplementary file 1 [file DataSheet1.zip › val/2000-2024-3-19-14-40-28.JPG]

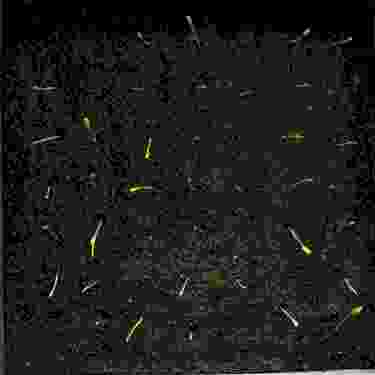

Supplement: Supplementary file 1 [file DataSheet1.zip › val/2000-2024-3-20-5-53-34.JPG]

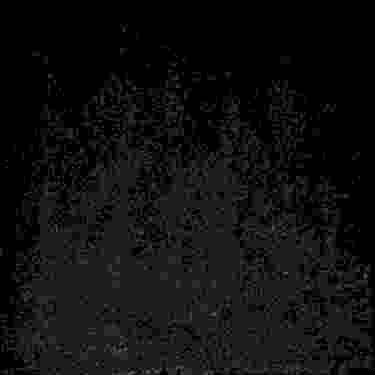

Supplement: Supplementary file 1 [file DataSheet1.zip › val/200120-2024-3-19-4-34-4.JPG]

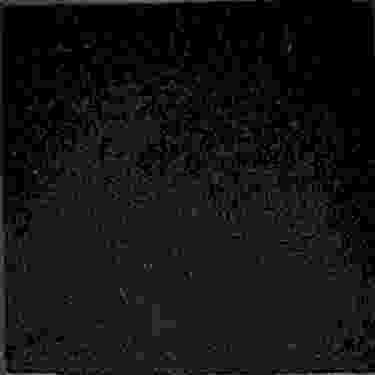

Supplement: Supplementary file 1 [file DataSheet1.zip › val/200150-2024-3-18-20-57-34.JPG]

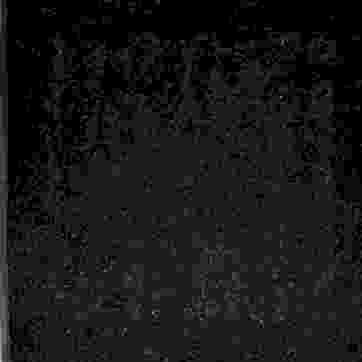

Supplement: Supplementary file 1 [file DataSheet1.zip › val/20060-2024-3-19-4-13-47.JPG]

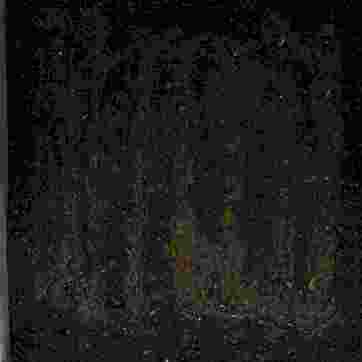

Supplement: Supplementary file 1 [file DataSheet1.zip › val/20060-2024-3-19-9-17-50.JPG]

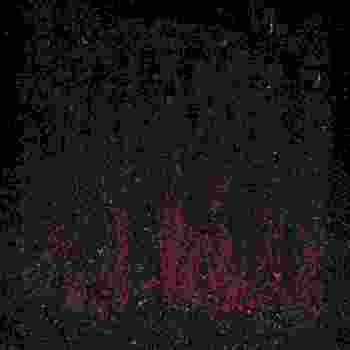

Supplement: Supplementary file 1 [file DataSheet1.zip › val/20060-2024-3-20-0-30-36.JPG]

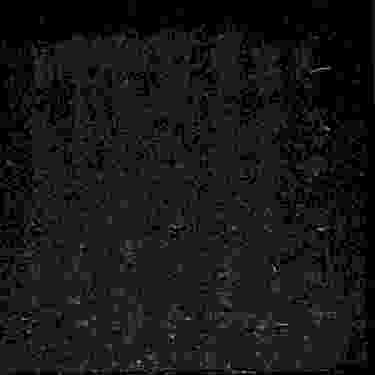

Supplement: Supplementary file 1 [file DataSheet1.zip › val/20090-2024-3-19-1-42-57.JPG]

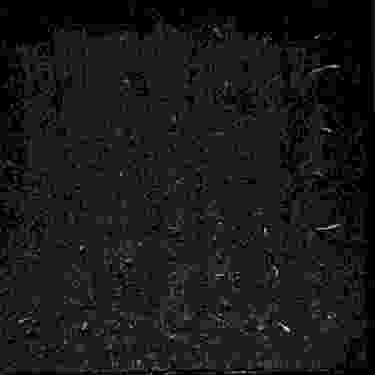

Supplement: Supplementary file 1 [file DataSheet1.zip › val/20090-2024-3-20-3-3-34.JPG]

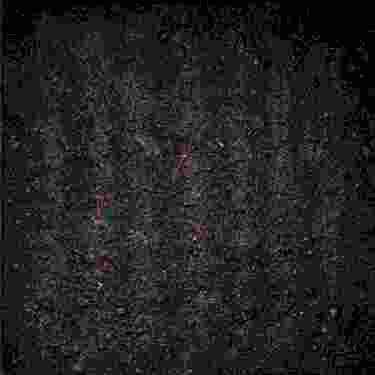

Supplement: Supplementary file 1 [file DataSheet1.zip › val/20120-2024-3-19-11-58-28.JPG]

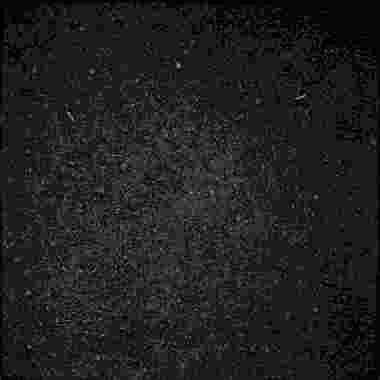

Supplement: Supplementary file 1 [file DataSheet1.zip › val/20150-2024-3-18-15-14-34.JPG]

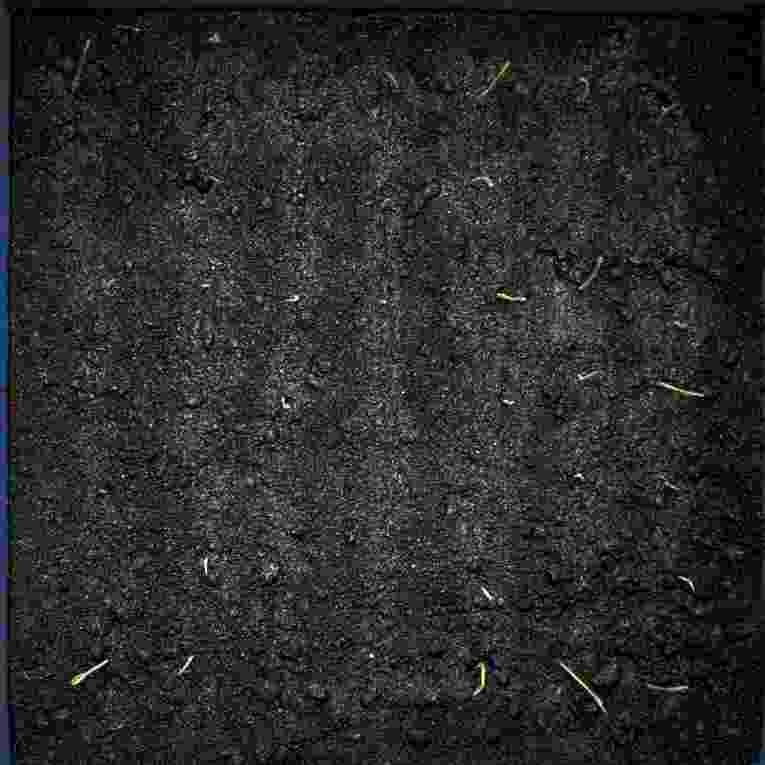

Supplement: Supplementary file 1 [file DataSheet1.zip › val/2030-2024-3-19-11-56-42.JPG]

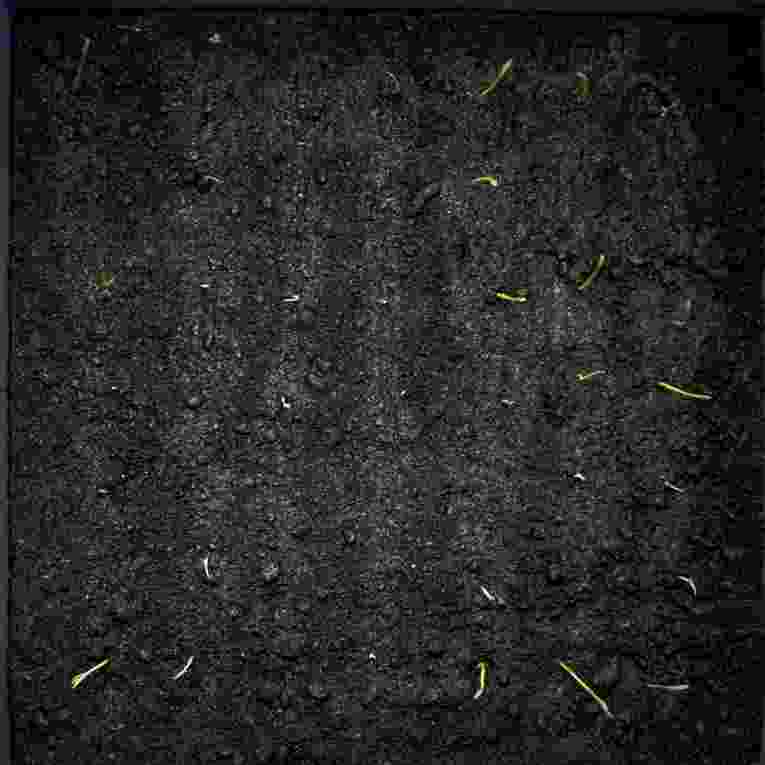

Supplement: Supplementary file 1 [file DataSheet1.zip › val/2030-2024-3-19-17-38-38.JPG]

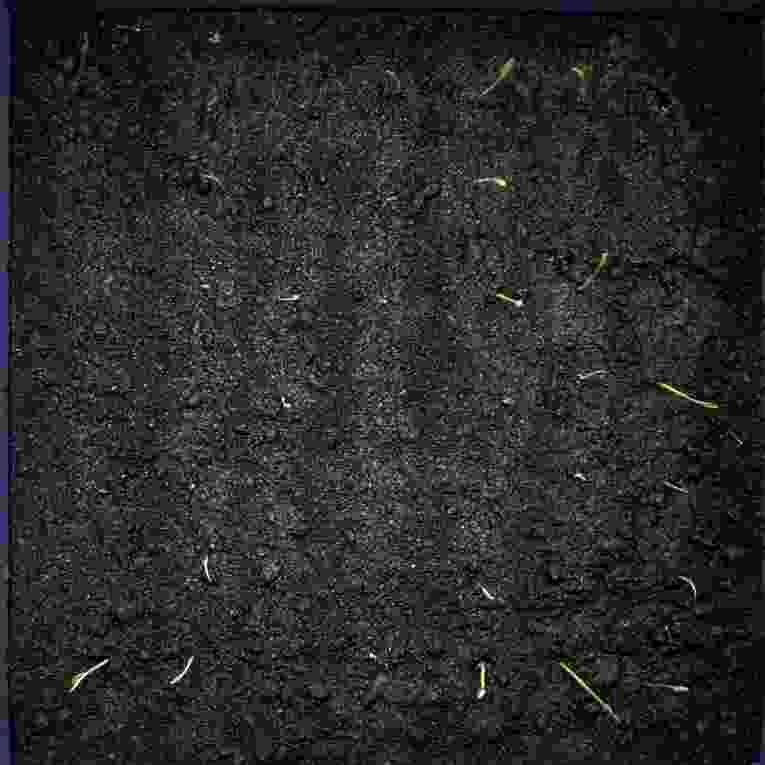

Supplement: Supplementary file 1 [file DataSheet1.zip › val/2030-2024-3-19-23-20-55.JPG]

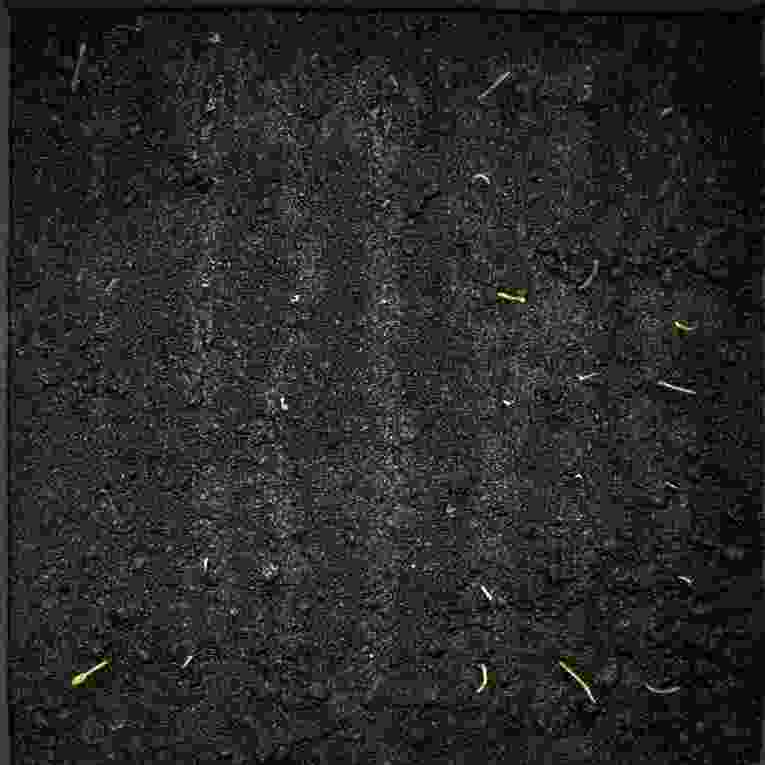

Supplement: Supplementary file 1 [file DataSheet1.zip › val/2030-2024-3-19-3-21-22.JPG]

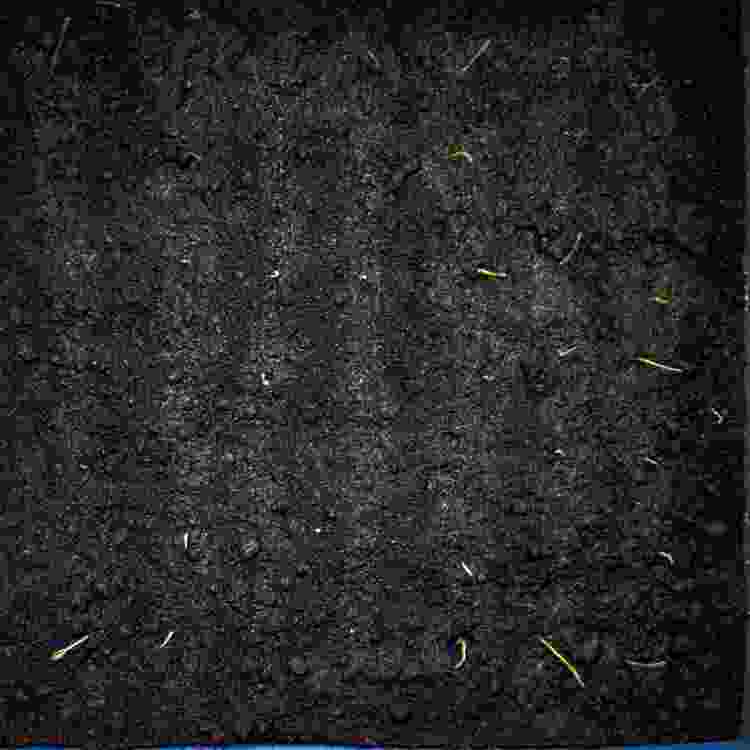

Supplement: Supplementary file 1 [file DataSheet1.zip › val/2030-2024-3-19-9-4-48.JPG]

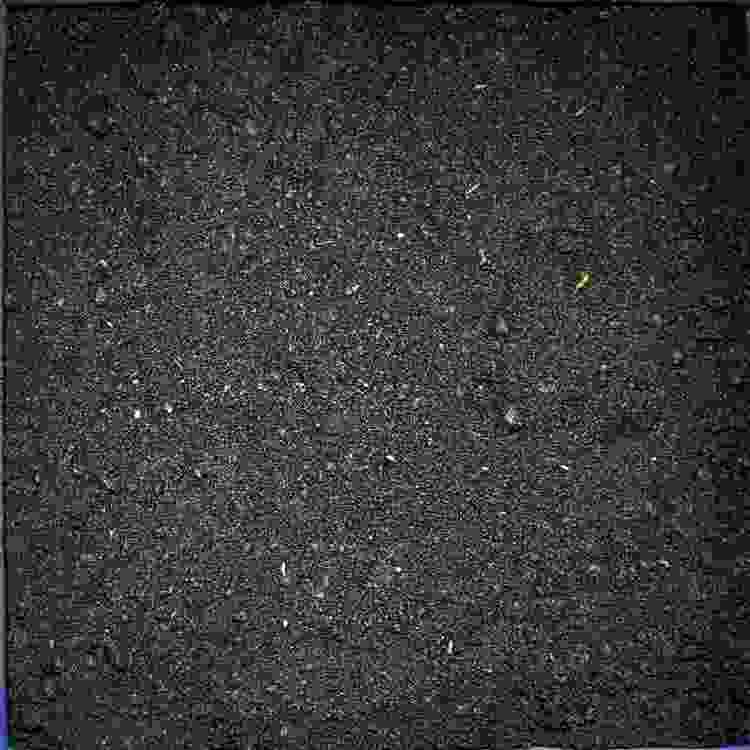

Supplement: Supplementary file 1 [file DataSheet1.zip › val/2060-2024-3-18-20-20-36.JPG]

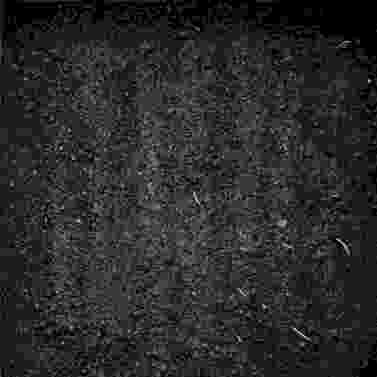

Supplement: Supplementary file 1 [file DataSheet1.zip › val/2090-2024-3-19-17-2-3.JPG]

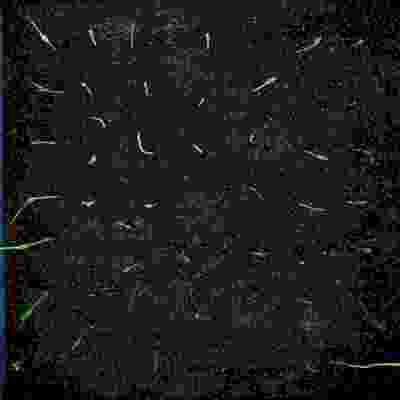

Supplement: Supplementary file 1 [file DataSheet1.zip › val/3000-2024-4-2-11-25-25.JPG]

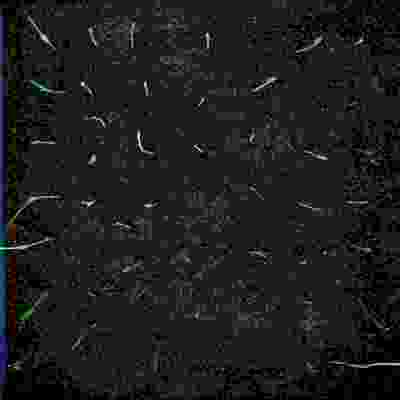

Supplement: Supplementary file 1 [file DataSheet1.zip › val/3000-2024-4-2-13-50-9.JPG]

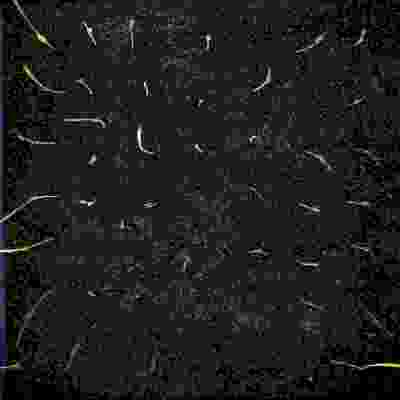

Supplement: Supplementary file 1 [file DataSheet1.zip › val/3000-2024-4-3-4-15-55.JPG]

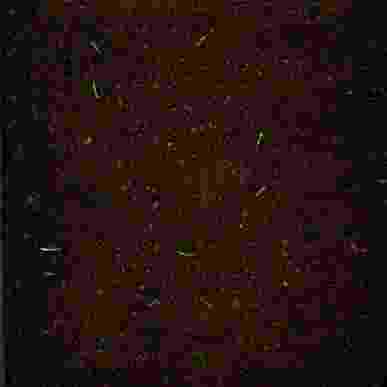

Supplement: Supplementary file 1 [file DataSheet1.zip › val/300120-2024-4-3-7-35-38.JPG]

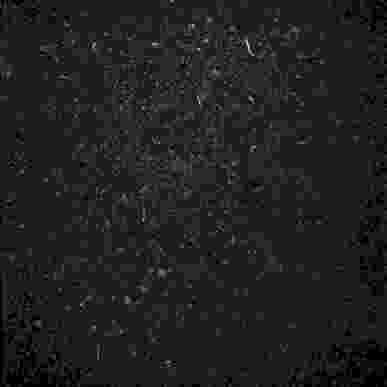

Supplement: Supplementary file 1 [file DataSheet1.zip › val/300150-2024-4-3-10-18-2.JPG]

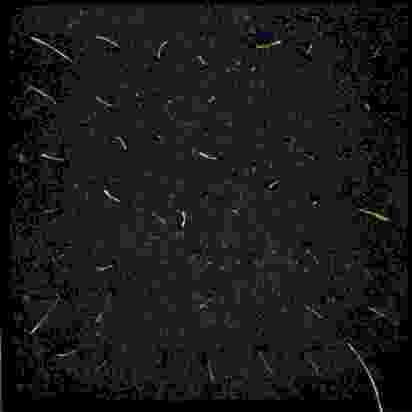

Supplement: Supplementary file 1 [file DataSheet1.zip › val/30030-2024-4-2-12-1-49.JPG]

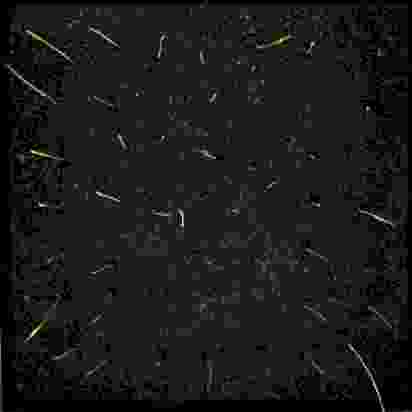

Supplement: Supplementary file 1 [file DataSheet1.zip › val/30030-2024-4-3-2-27-39.JPG]

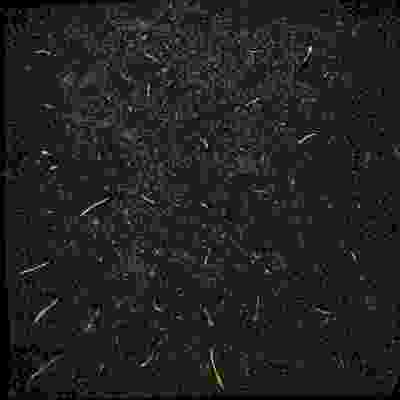

Supplement: Supplementary file 1 [file DataSheet1.zip › val/30060-2024-4-2-19-15-30.JPG]

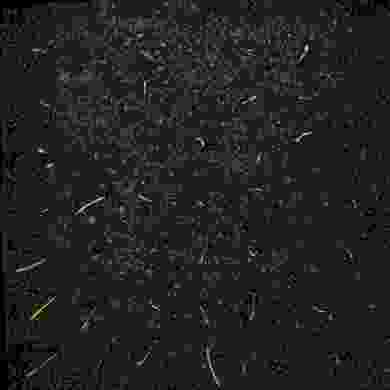

Supplement: Supplementary file 1 [file DataSheet1.zip › val/30060-2024-4-2-21-39-22.JPG]

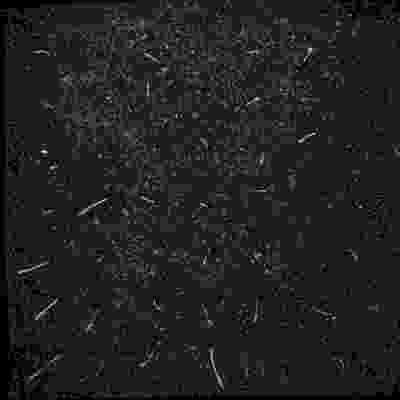

Supplement: Supplementary file 1 [file DataSheet1.zip › val/30060-2024-4-3-0-4-15.JPG]

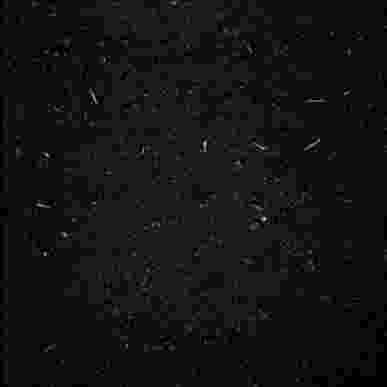

Supplement: Supplementary file 1 [file DataSheet1.zip › val/30090-2024-4-2-2-44-17.JPG]

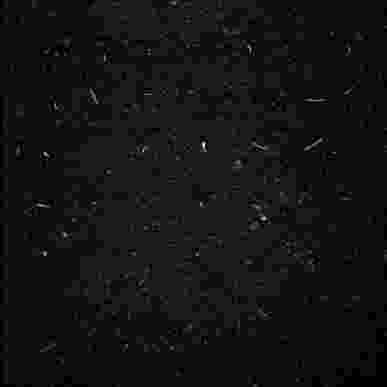

Supplement: Supplementary file 1 [file DataSheet1.zip › val/30090-2024-4-2-7-32-50.JPG]

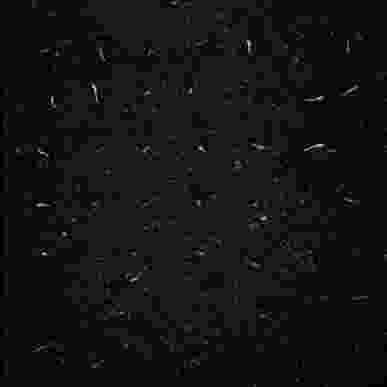

Supplement: Supplementary file 1 [file DataSheet1.zip › val/30090-2024-4-3-0-22-46.JPG]

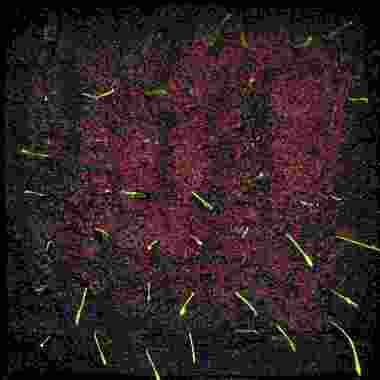

Supplement: Supplementary file 1 [file DataSheet1.zip › val/500-2024-3-20-3-22-51.JPG]

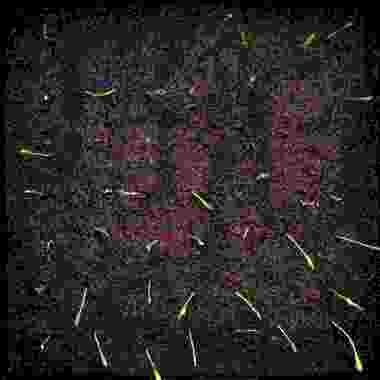

Supplement: Supplementary file 1 [file DataSheet1.zip › val/500-2024-3-20-8-58-43.JPG]

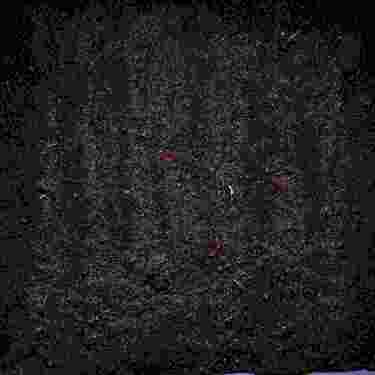

Supplement: Supplementary file 1 [file DataSheet1.zip › val/50120-2024-3-20-0-0-38.JPG]

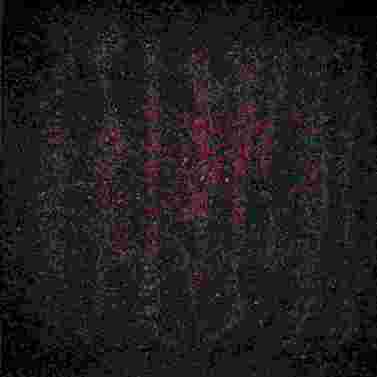

Supplement: Supplementary file 1 [file DataSheet1.zip › val/50150-2024-3-19-5-59-53.JPG]

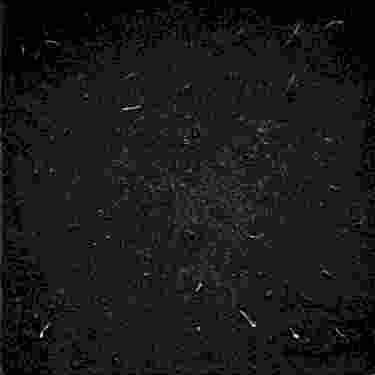

Supplement: Supplementary file 1 [file DataSheet1.zip › val/5030-2024-3-18-17-33-17.JPG]

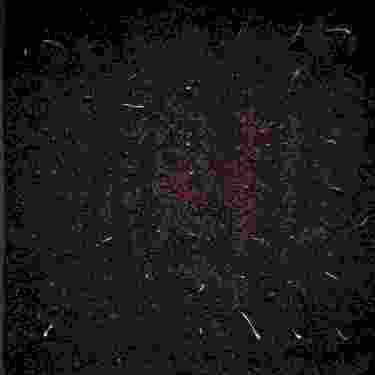

Supplement: Supplementary file 1 [file DataSheet1.zip › val/5030-2024-3-19-4-43-43.JPG]

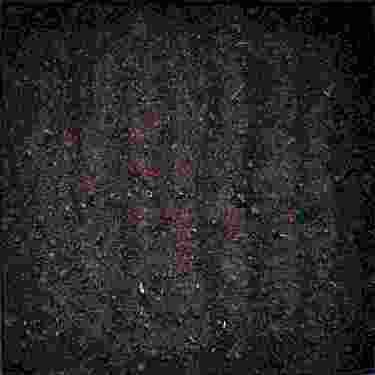

Supplement: Supplementary file 1 [file DataSheet1.zip › val/5060-2024-3-19-19-13-58.JPG]

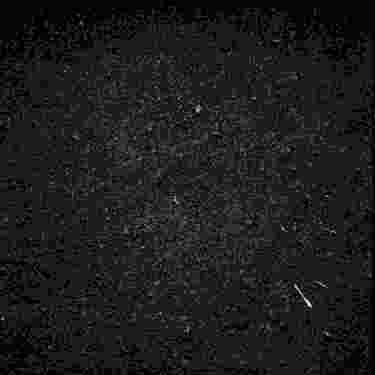

Supplement: Supplementary file 1 [file DataSheet1.zip › val/5090-2024-3-18-17-15-47.JPG]

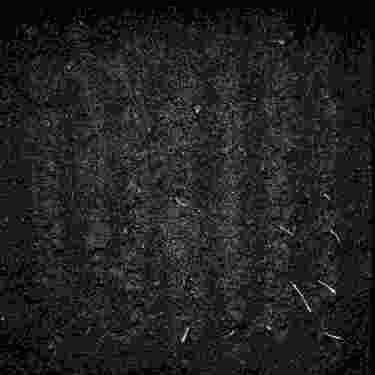

Supplement: Supplementary file 1 [file DataSheet1.zip › val/5090-2024-3-19-15-37-34.JPG]

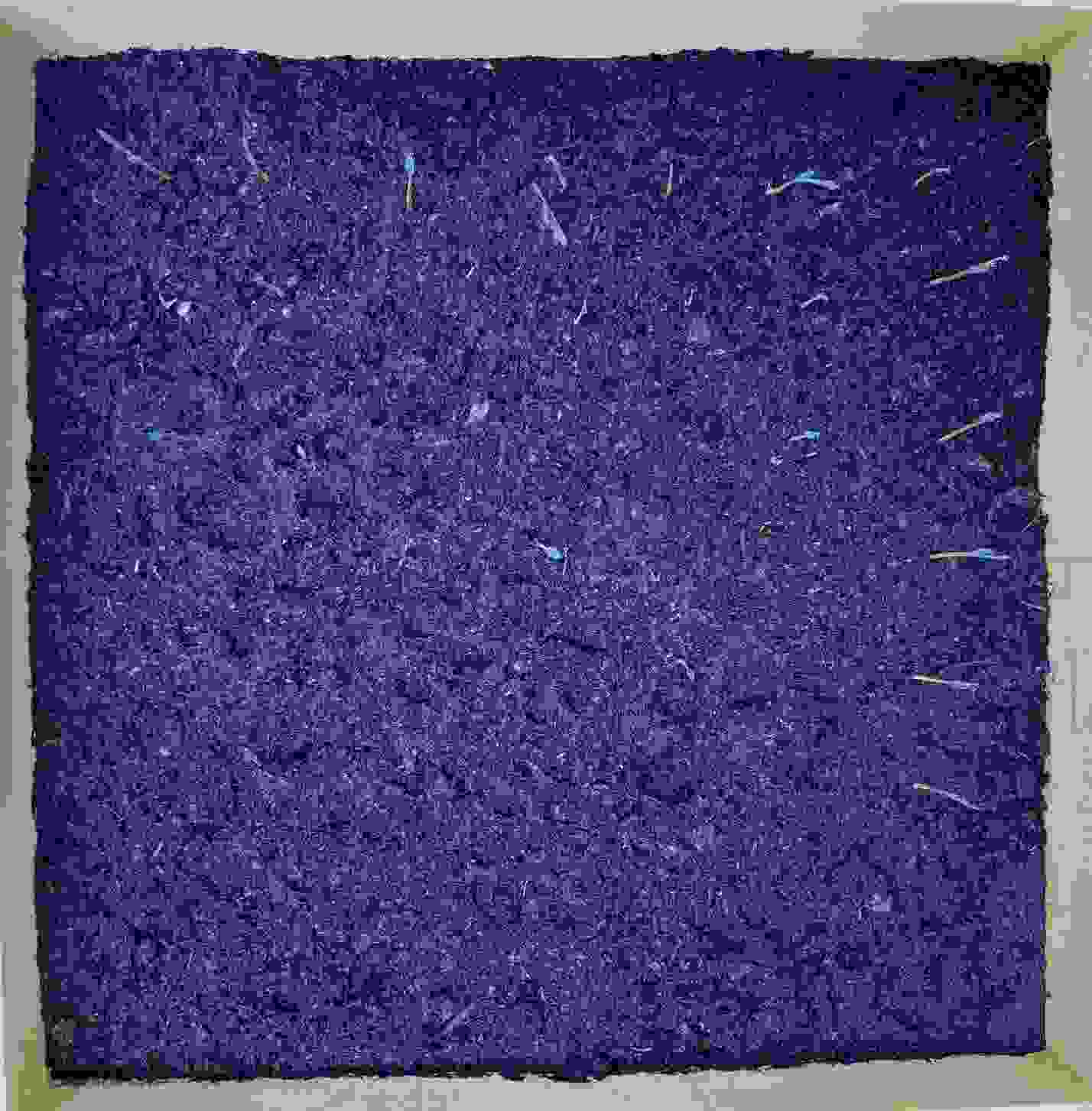

Supplement: Supplementary file 1 [file DataSheet1.zip › val/9-4.JPG]

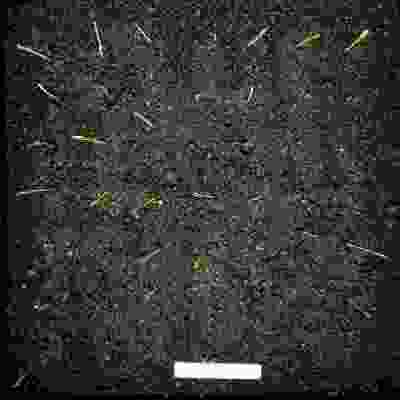

Supplement: Supplementary file 1 [file DataSheet1.zip › val/ck-2024-3-19-11-16-24.JPG]

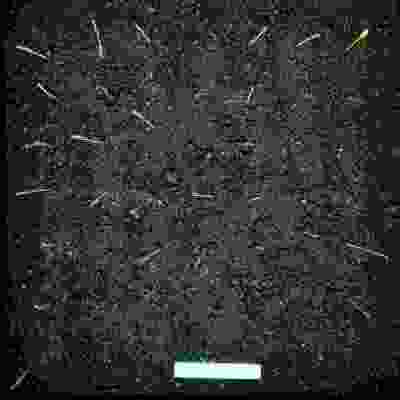

Supplement: Supplementary file 1 [file DataSheet1.zip › val/ck-2024-3-19-13-40-59.JPG]

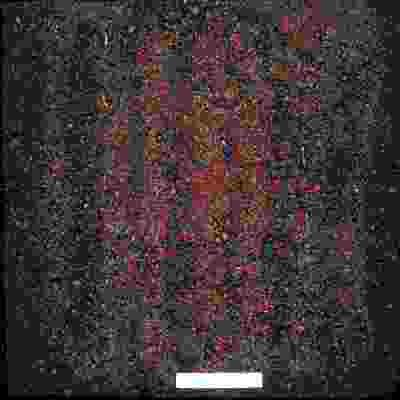

Supplement: Supplementary file 1 [file DataSheet1.zip › val/ck120-2024-3-20-7-44-21.JPG]

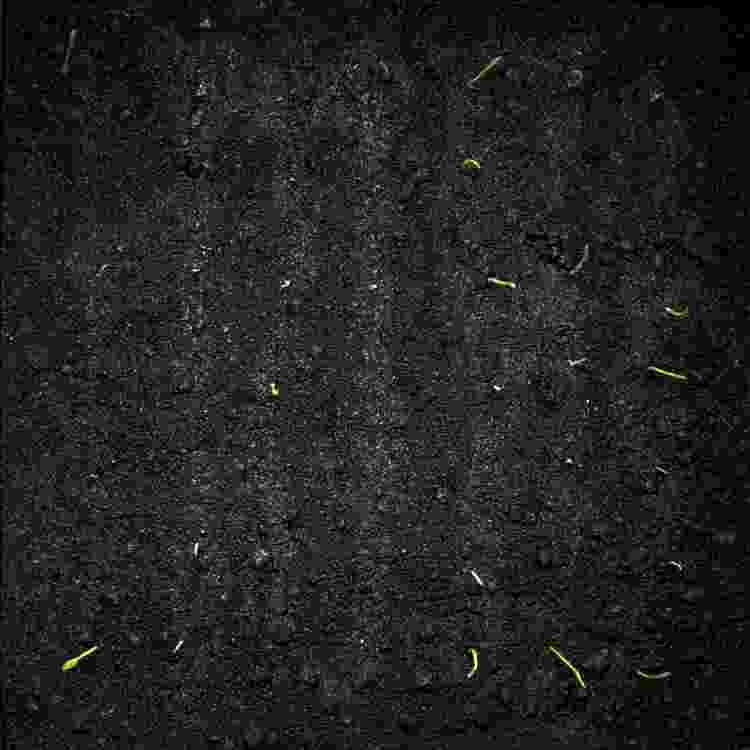

Supplement: Supplementary file 1 [file DataSheet1.zip › val/ck30-2024-3-19-3-2-10.JPG]

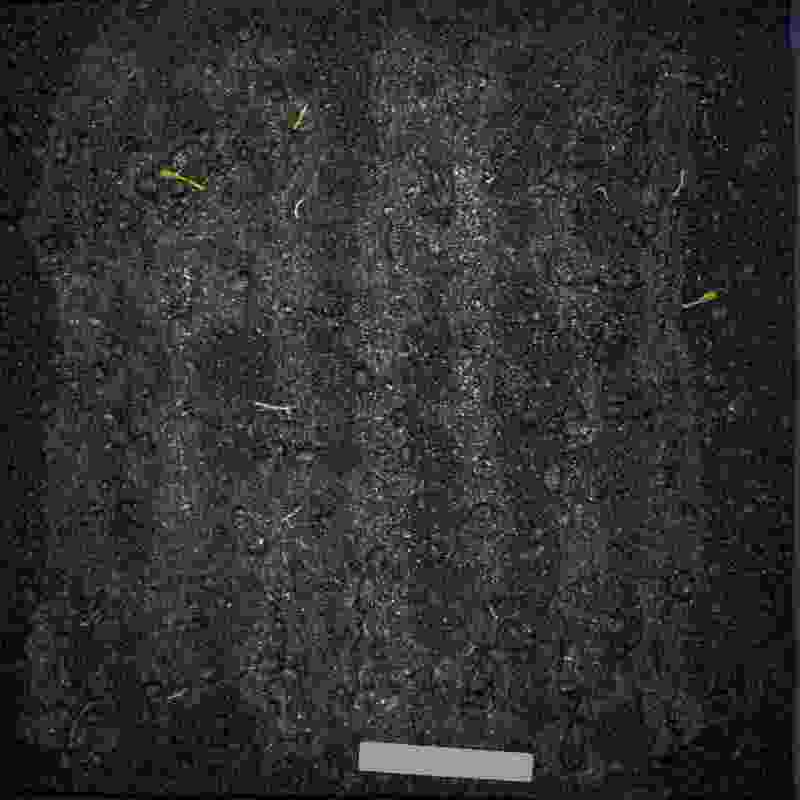

Supplement: Supplementary file 1 [file DataSheet1.zip › val/ck60-2024-3-19-10-40-57.JPG]

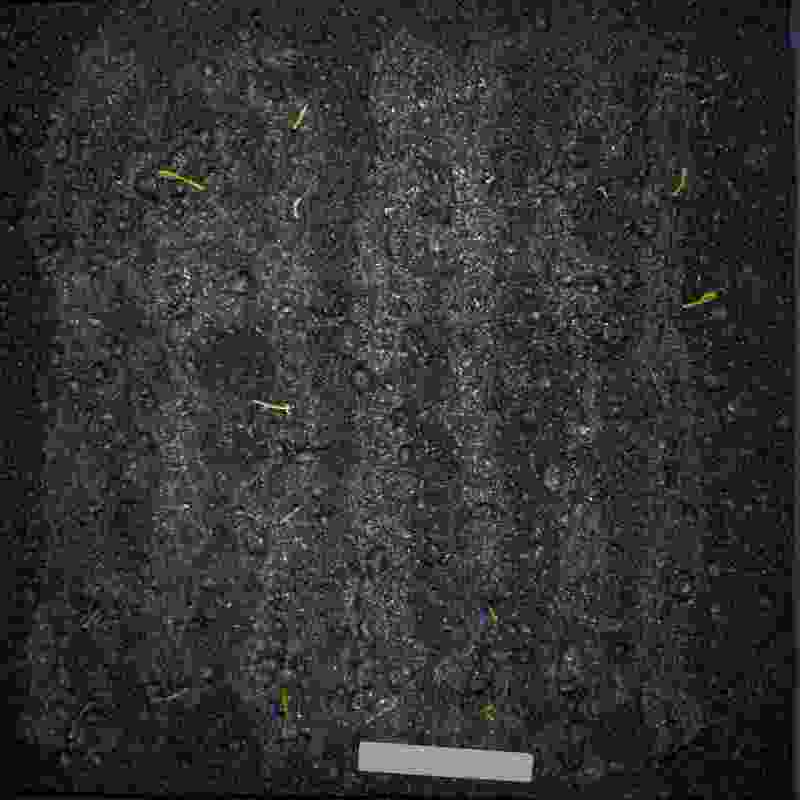

Supplement: Supplementary file 1 [file DataSheet1.zip › val/ck60-2024-3-19-13-41-42.JPG]

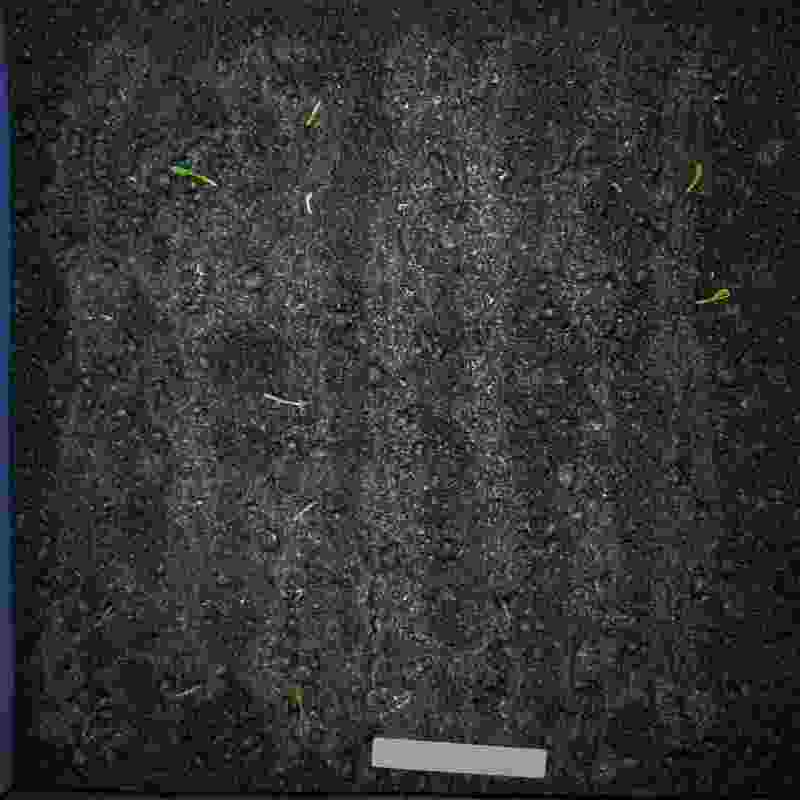

Supplement: Supplementary file 1 [file DataSheet1.zip › val/ck60-2024-3-19-16-41-41.JPG]

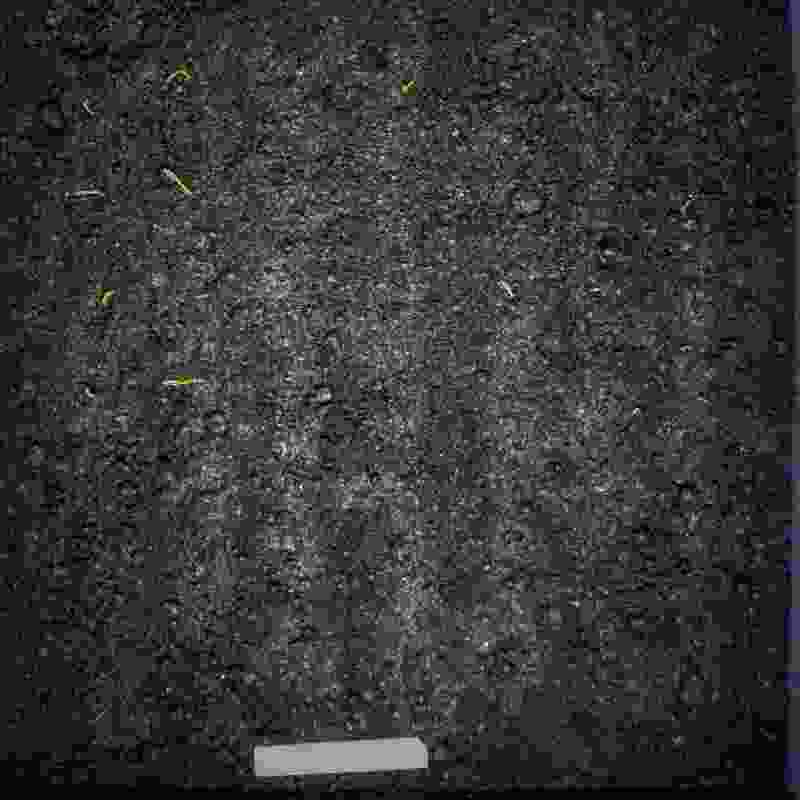

Supplement: Supplementary file 1 [file DataSheet1.zip › val/ck90-2024-3-19-13-42-18.JPG]

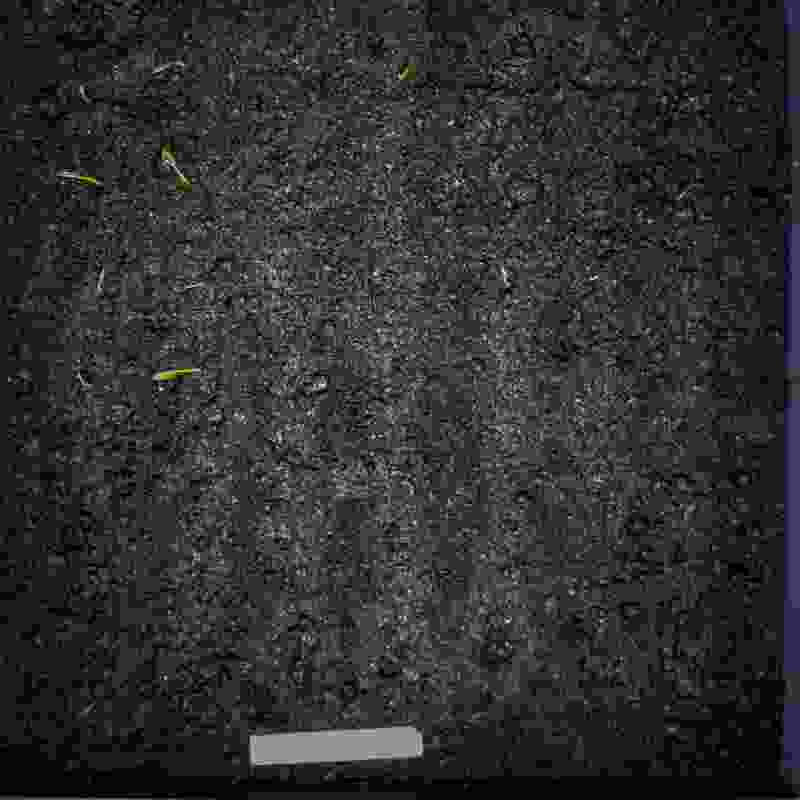

Supplement: Supplementary file 1 [file DataSheet1.zip › val/ck90-2024-3-20-1-43-16.JPG]

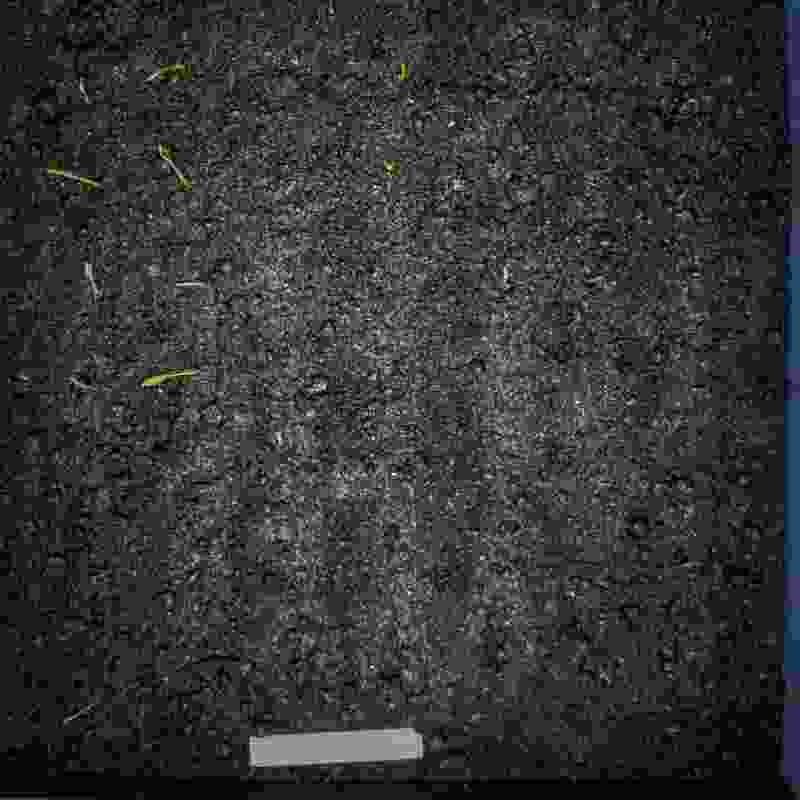

Supplement: Supplementary file 1 [file DataSheet1.zip › val/ck90-2024-3-20-13-44-23.JPG]

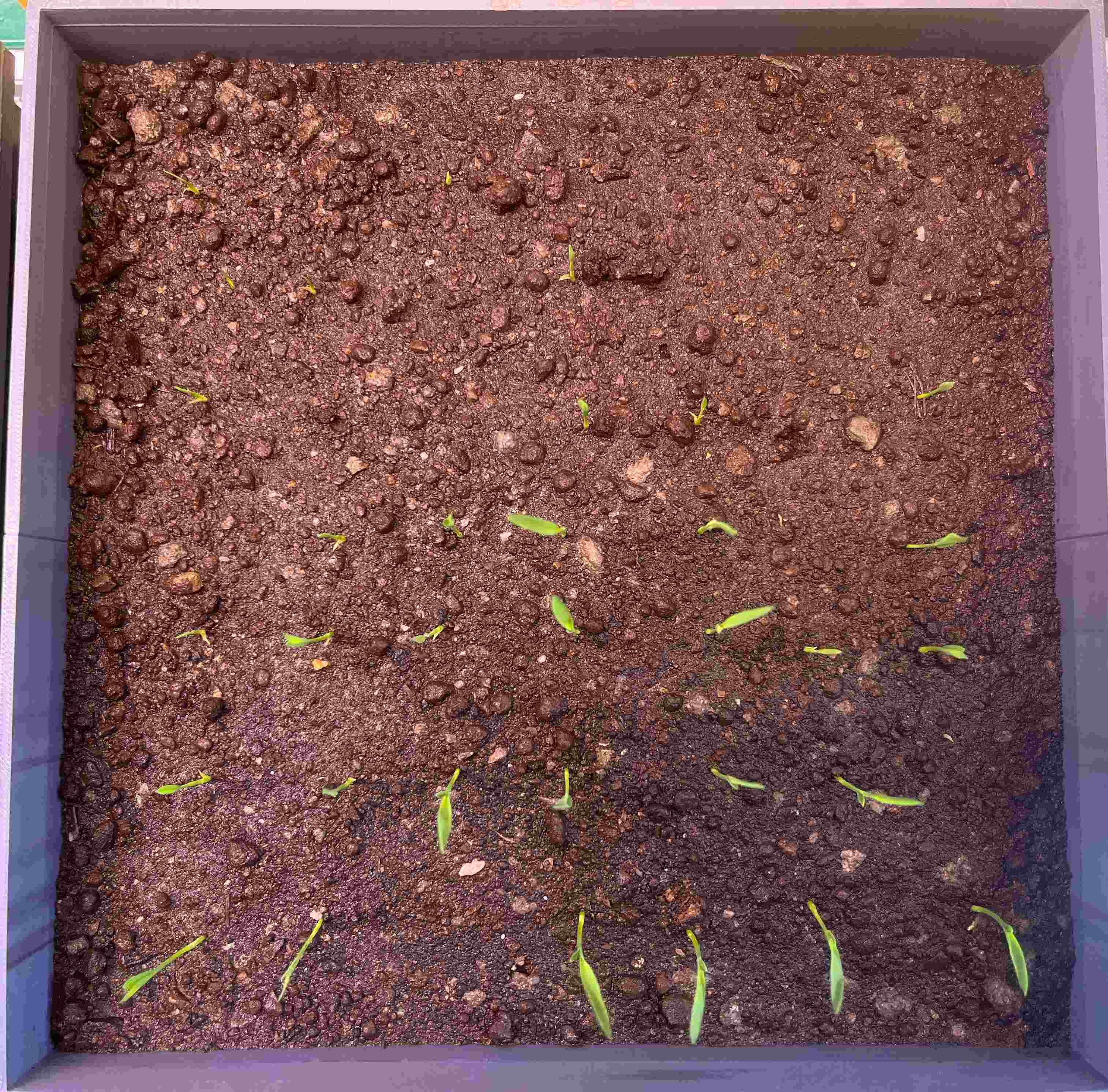

Supplement: Supplementary file 2 [file DataSheet2.zip › test/10-1.JPG]

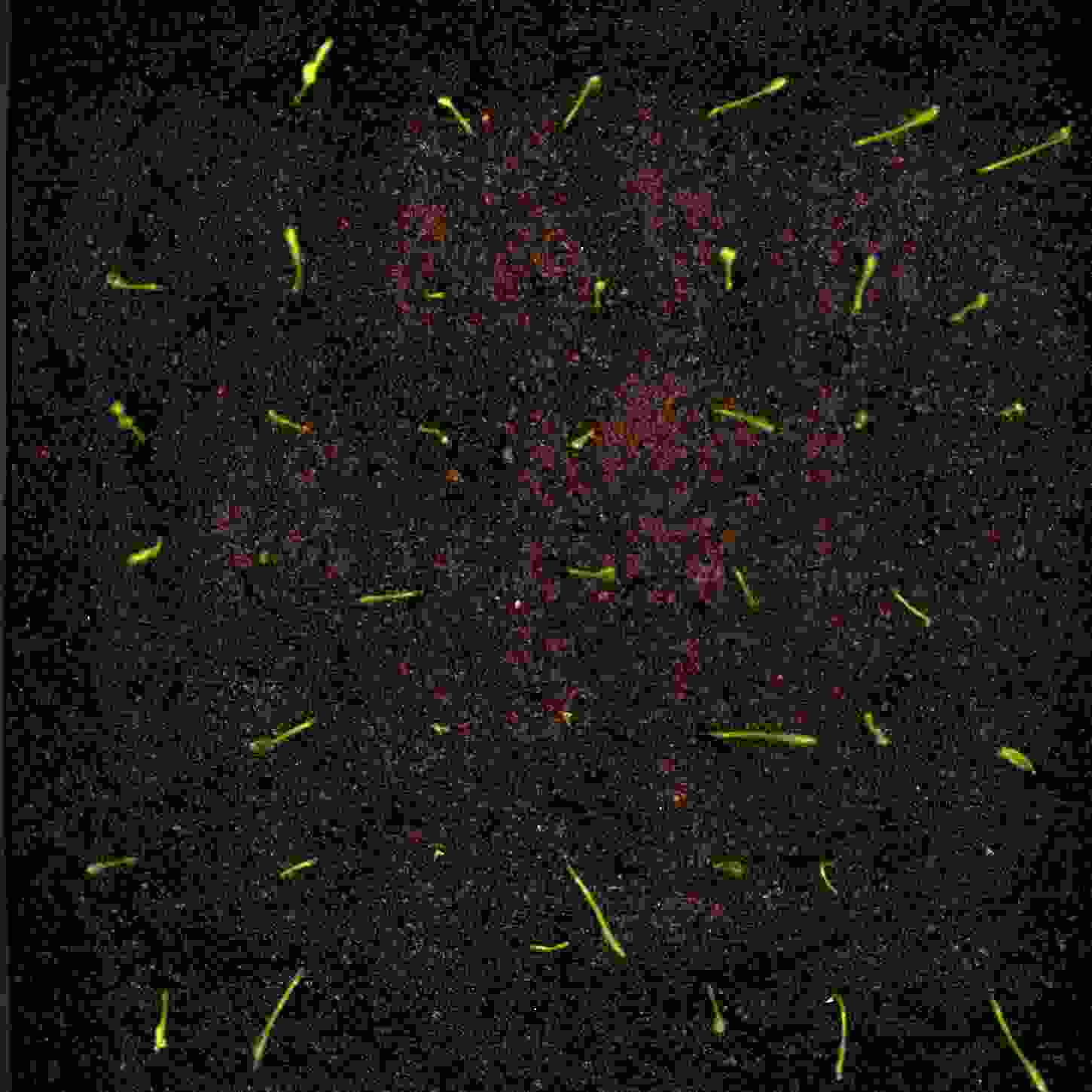

Supplement: Supplementary file 2 [file DataSheet2.zip › test/1000-2024-3-20-3-34-42.JPG]

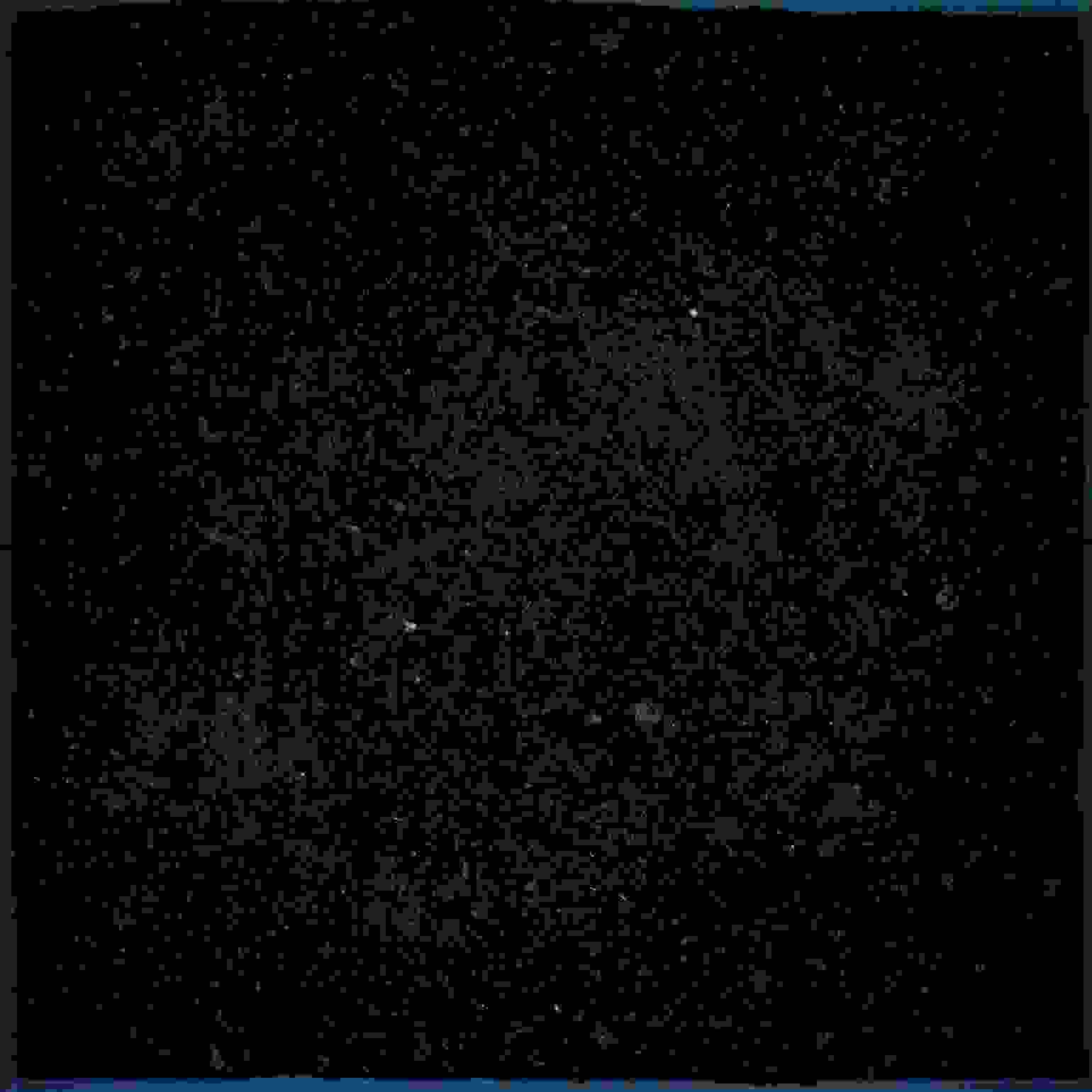

Supplement: Supplementary file 2 [file DataSheet2.zip › test/100120-100120-2024-3-18-15-51-3.JPG]

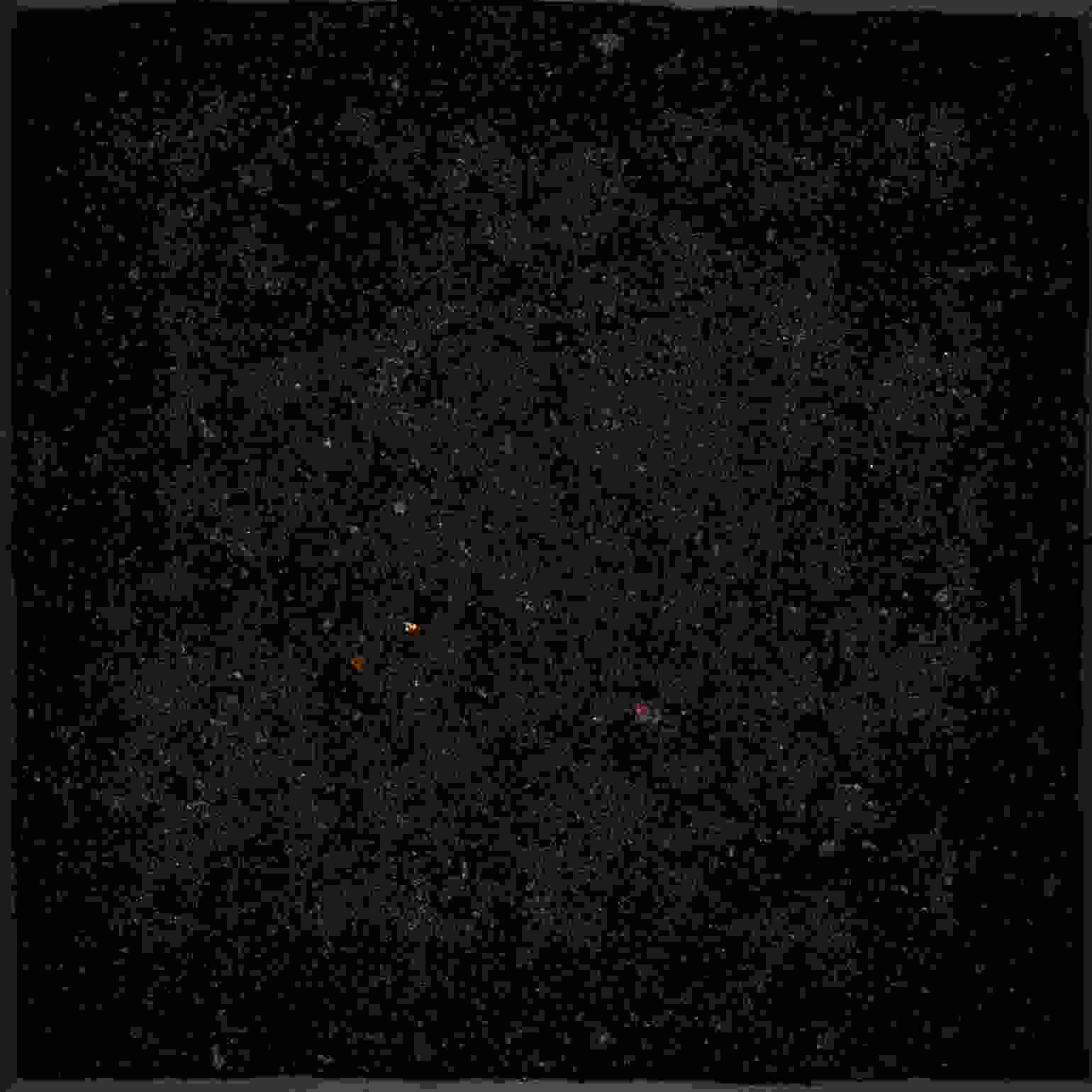

Supplement: Supplementary file 2 [file DataSheet2.zip › test/100120-2024-3-18-23-34-36.JPG]

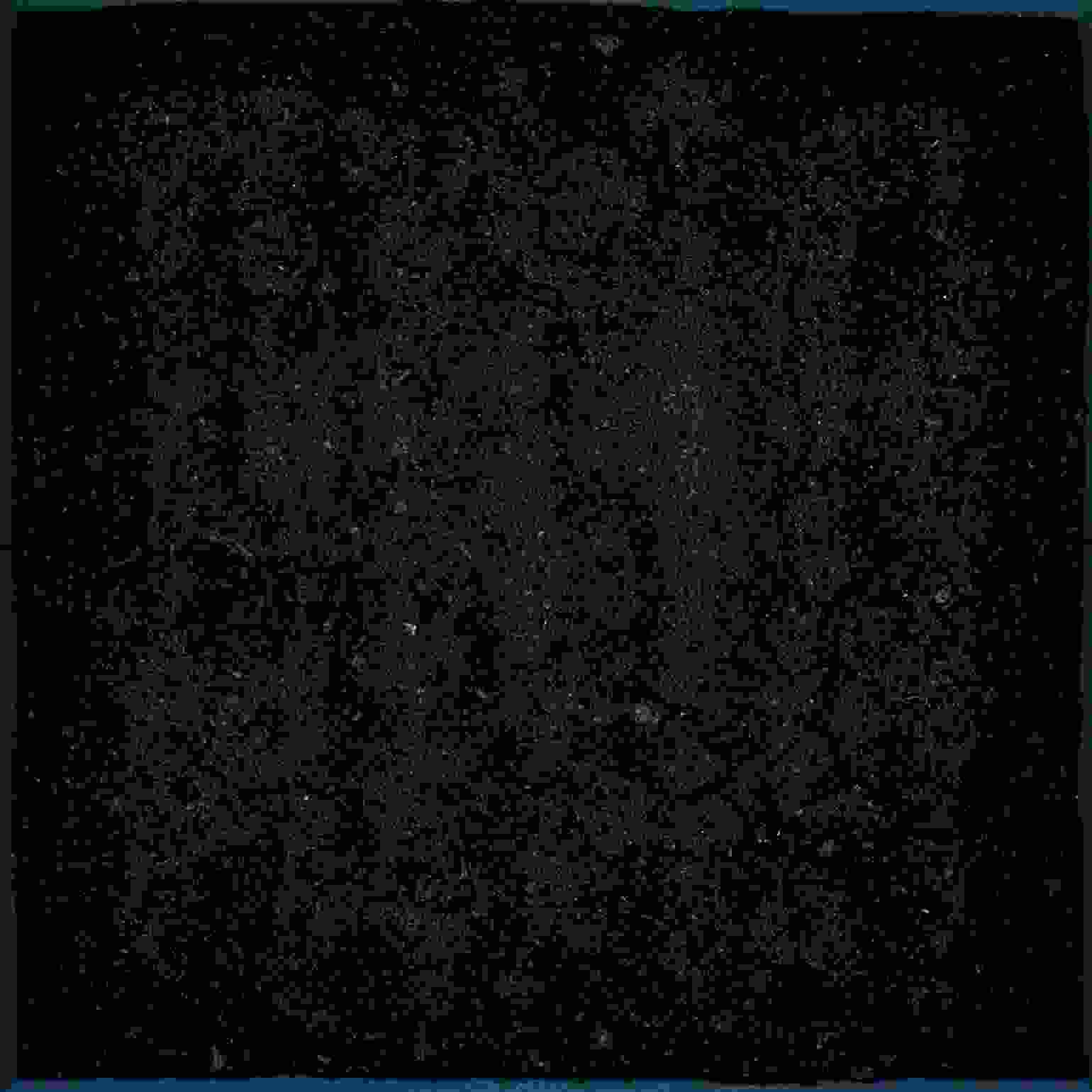

Supplement: Supplementary file 2 [file DataSheet2.zip › test/100120-2024-3-19-14-52-51.JPG]

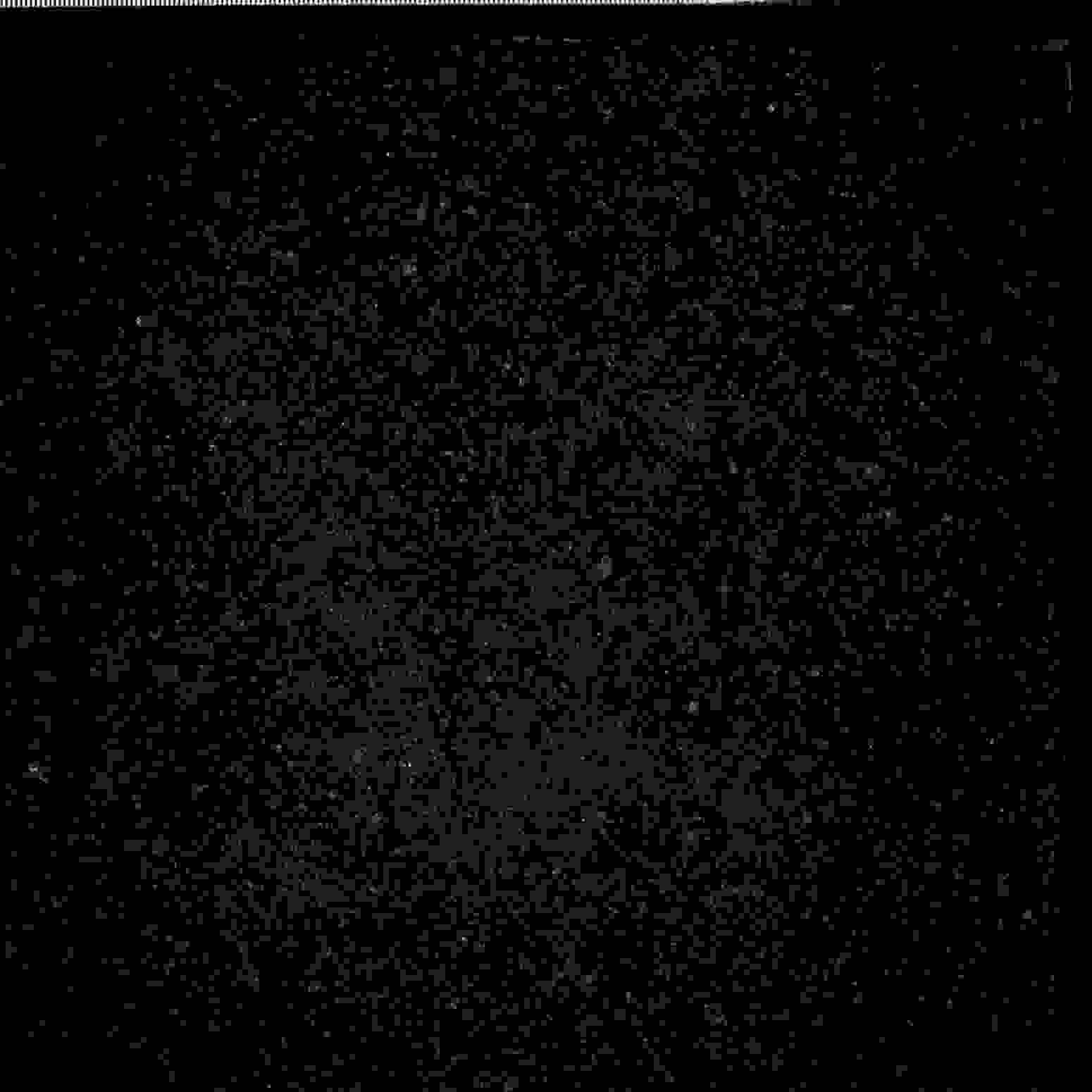

Supplement: Supplementary file 2 [file DataSheet2.zip › test/100150-2024-3-18-18-45-6.JPG]

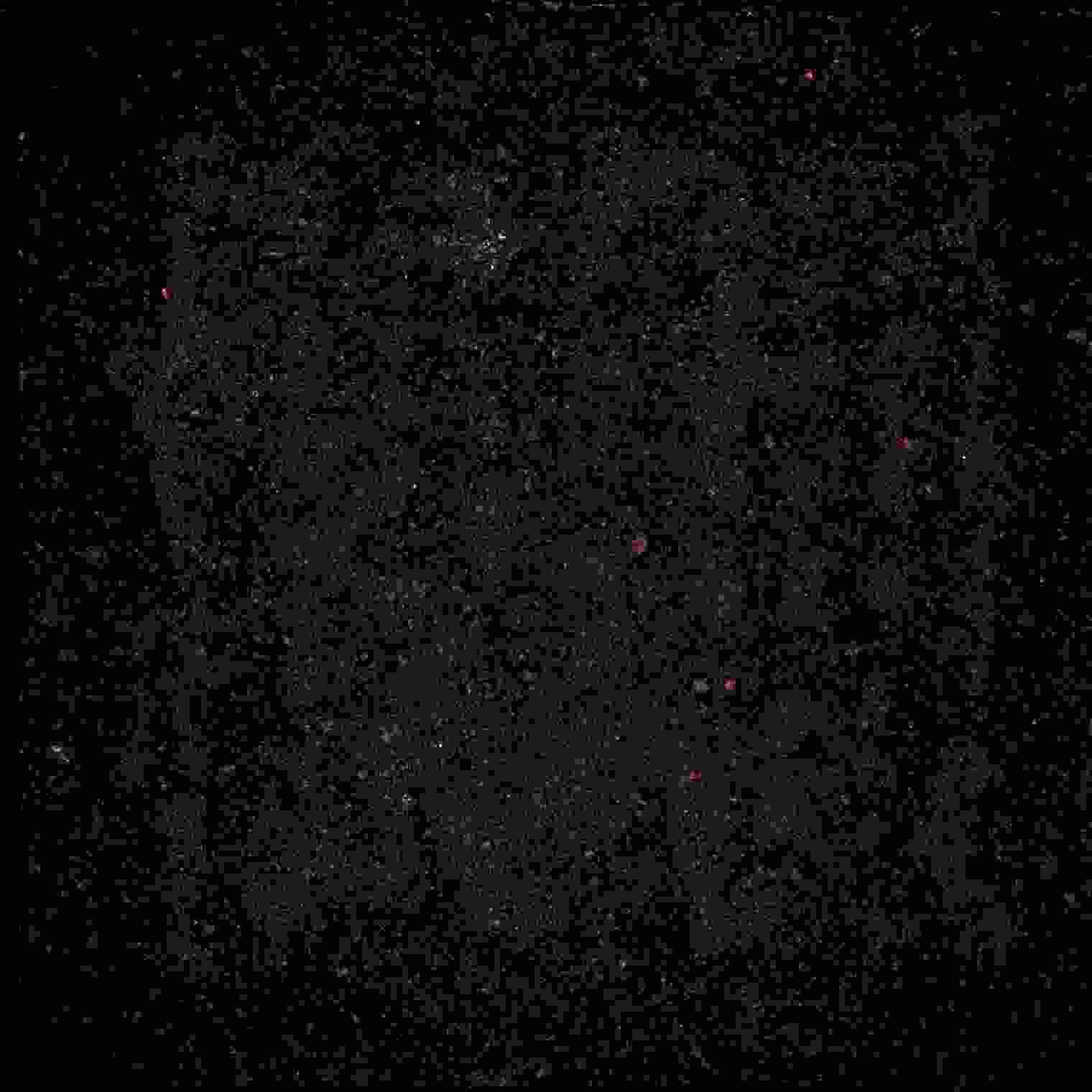

Supplement: Supplementary file 2 [file DataSheet2.zip › test/100150-2024-3-18-23-54-28.JPG]

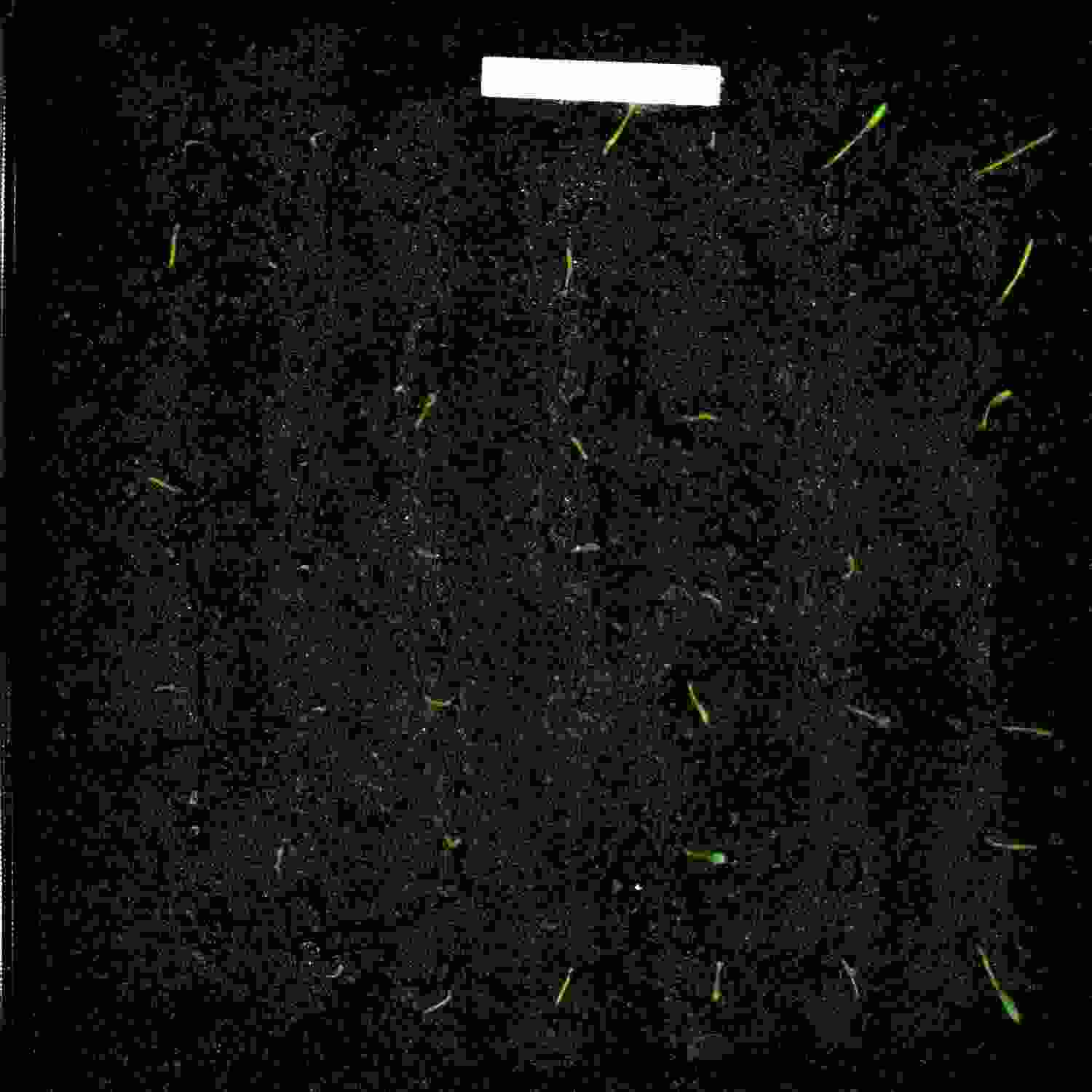

Supplement: Supplementary file 2 [file DataSheet2.zip › test/10030-2024-3-19-9-46-8.JPG]

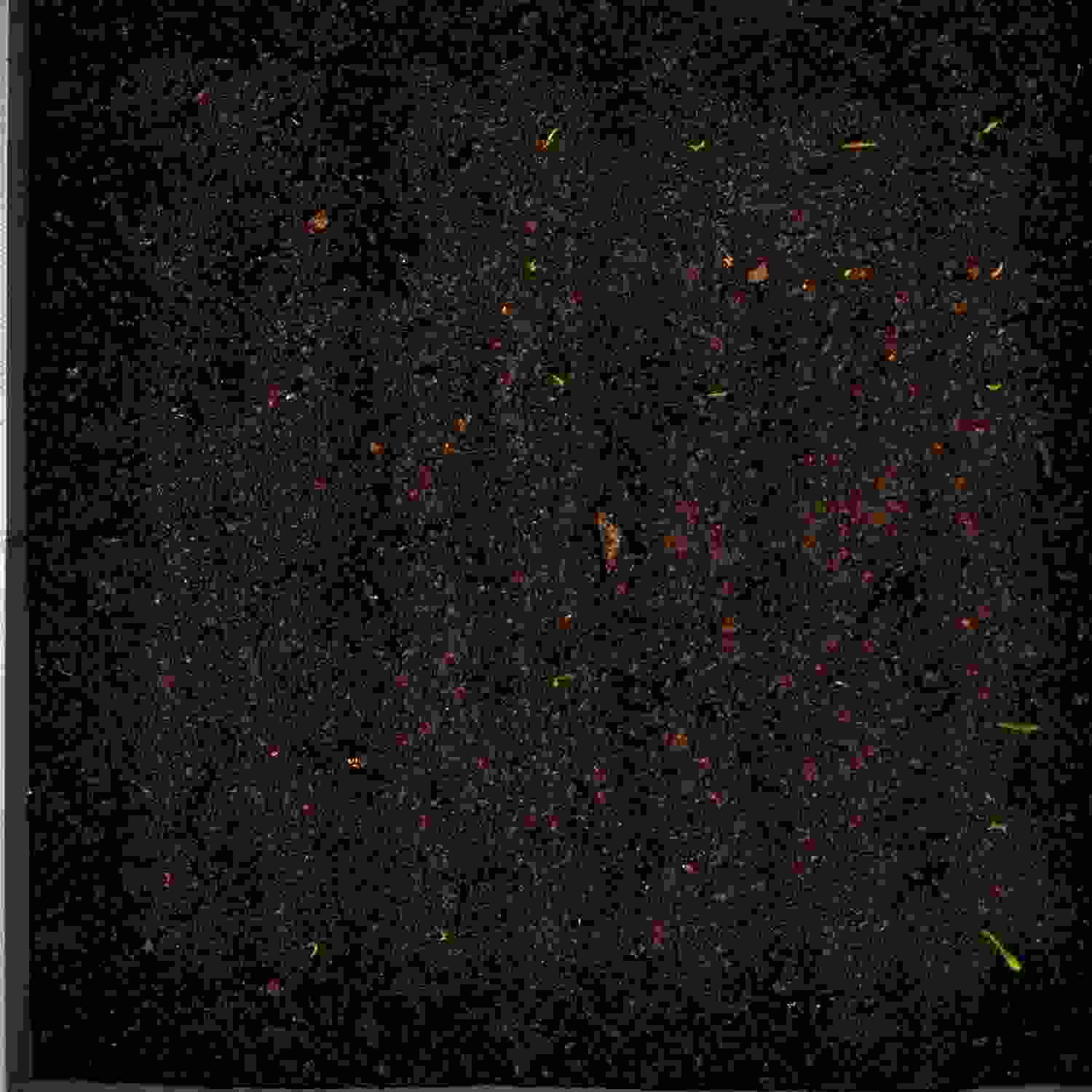

Supplement: Supplementary file 2 [file DataSheet2.zip › test/10060-2024-3-19-12-38-16.JPG]

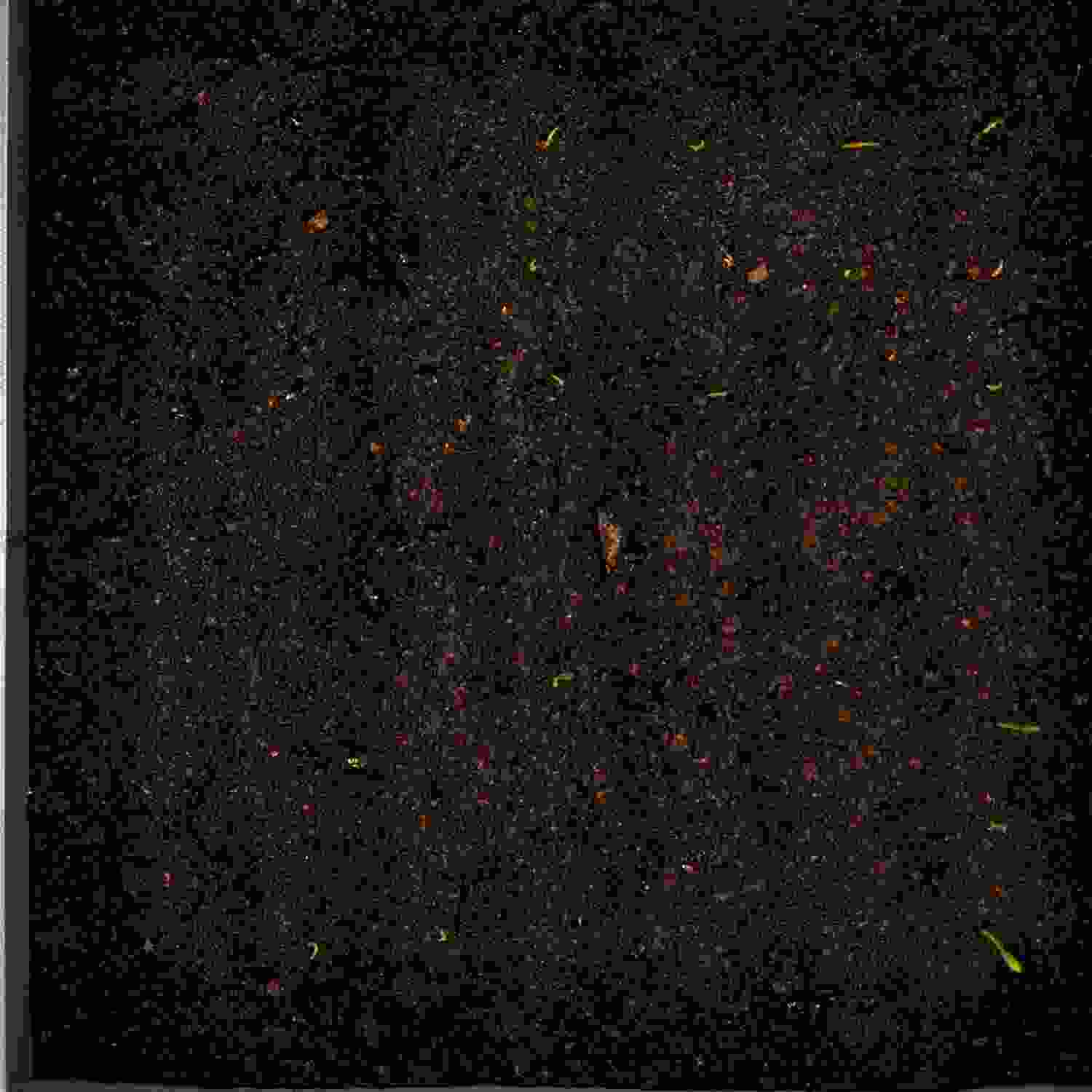

Supplement: Supplementary file 2 [file DataSheet2.zip › test/10060-2024-3-19-15-10-37.JPG]

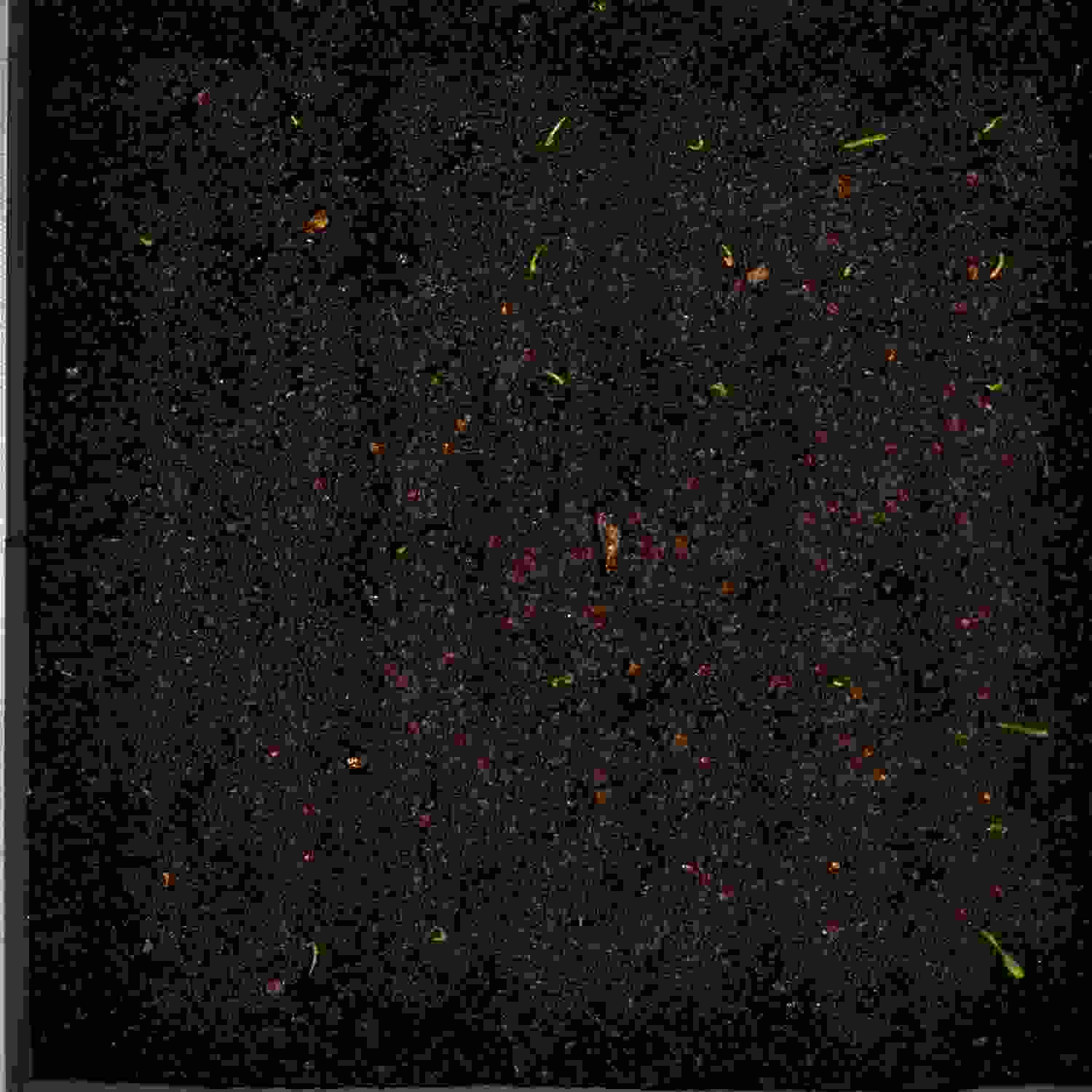

Supplement: Supplementary file 2 [file DataSheet2.zip › test/10060-2024-3-20-3-54-43.JPG]

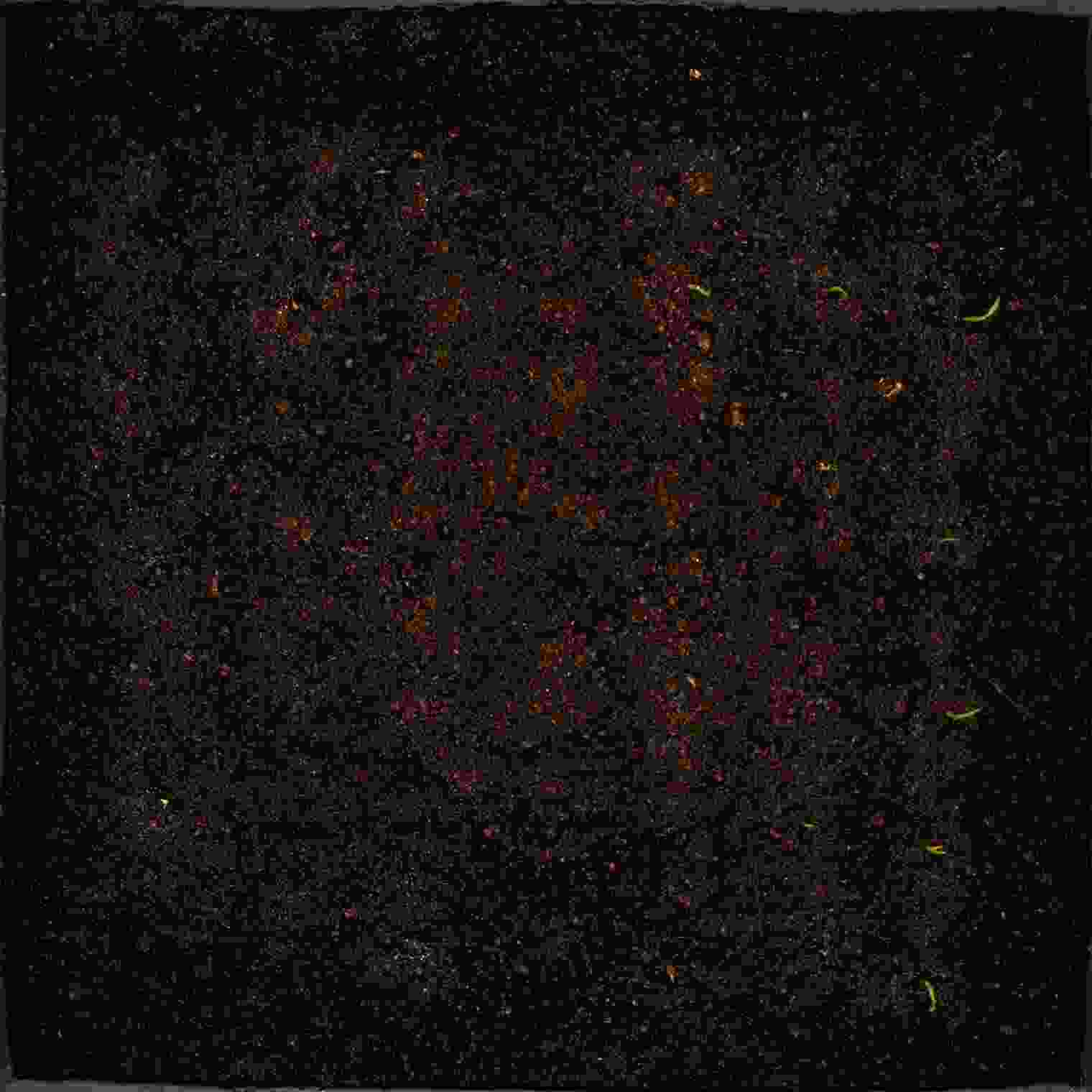

Supplement: Supplementary file 2 [file DataSheet2.zip › test/10090-2024-3-18-23-34-5.JPG]

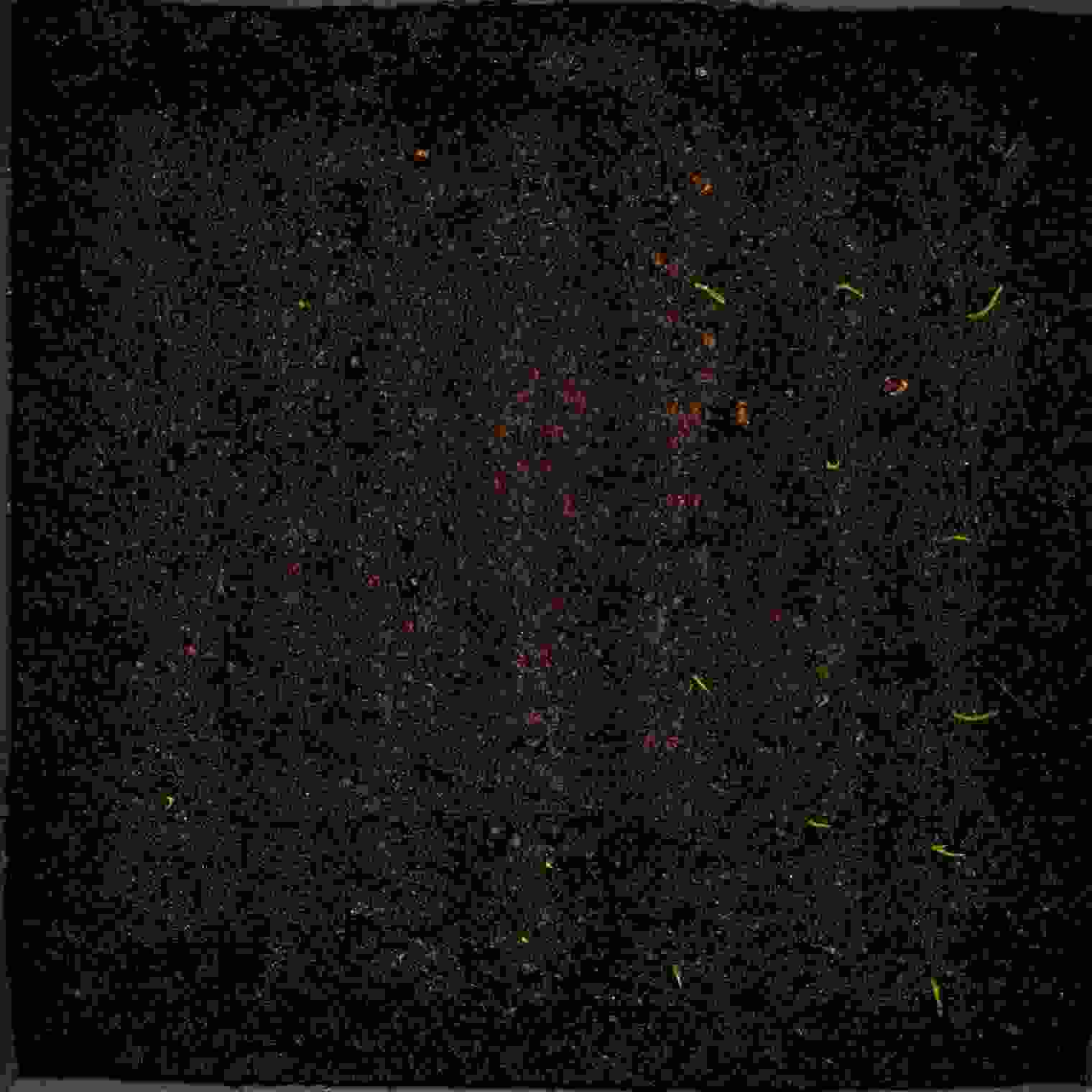

Supplement: Supplementary file 2 [file DataSheet2.zip › test/10090-2024-3-19-12-20-5.JPG]

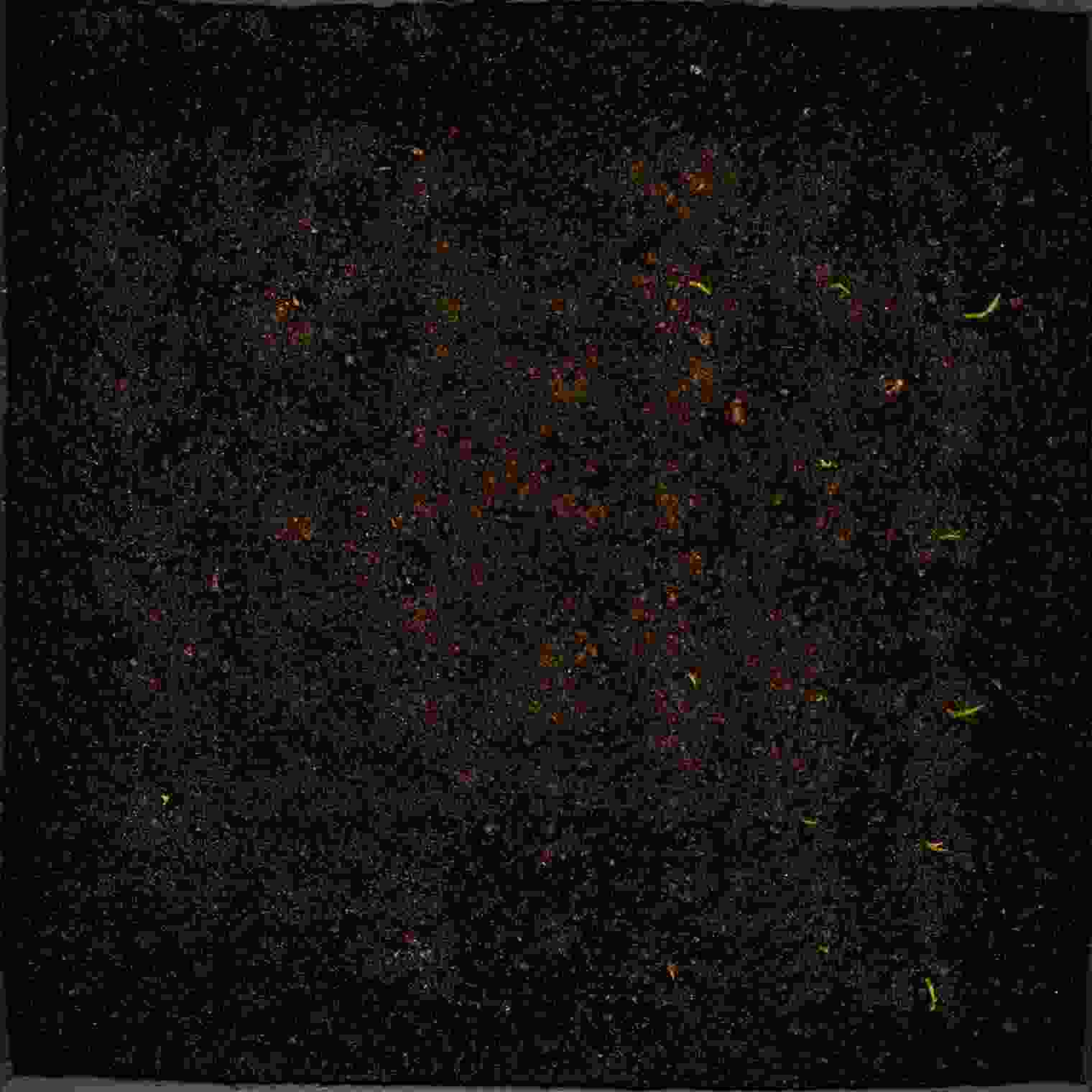

Supplement: Supplementary file 2 [file DataSheet2.zip › test/10090-2024-3-19-2-7-36.JPG]

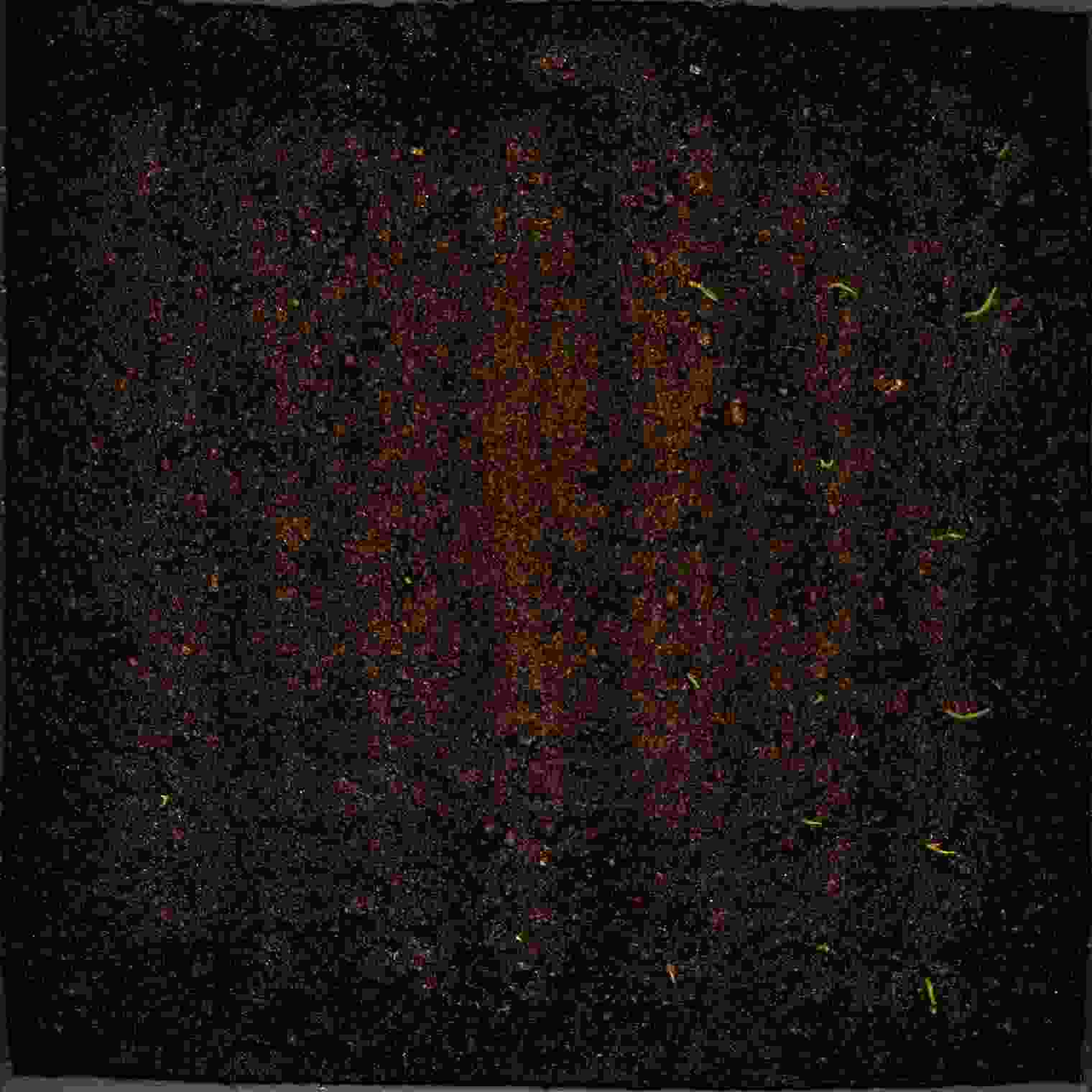

Supplement: Supplementary file 2 [file DataSheet2.zip › test/10090-2024-3-19-9-47-34.JPG]

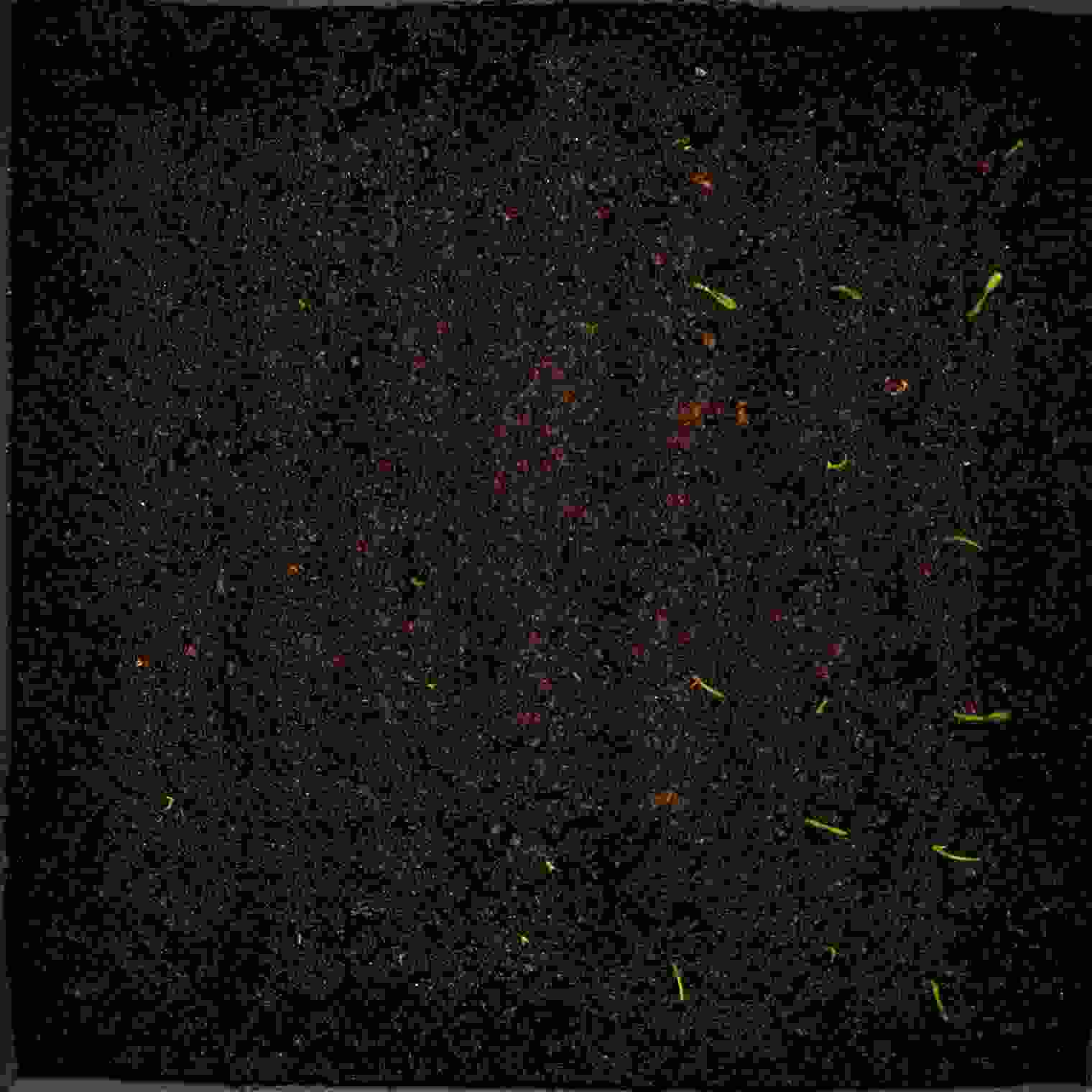

Supplement: Supplementary file 2 [file DataSheet2.zip › test/10090-2024-3-20-1-3-47.JPG]

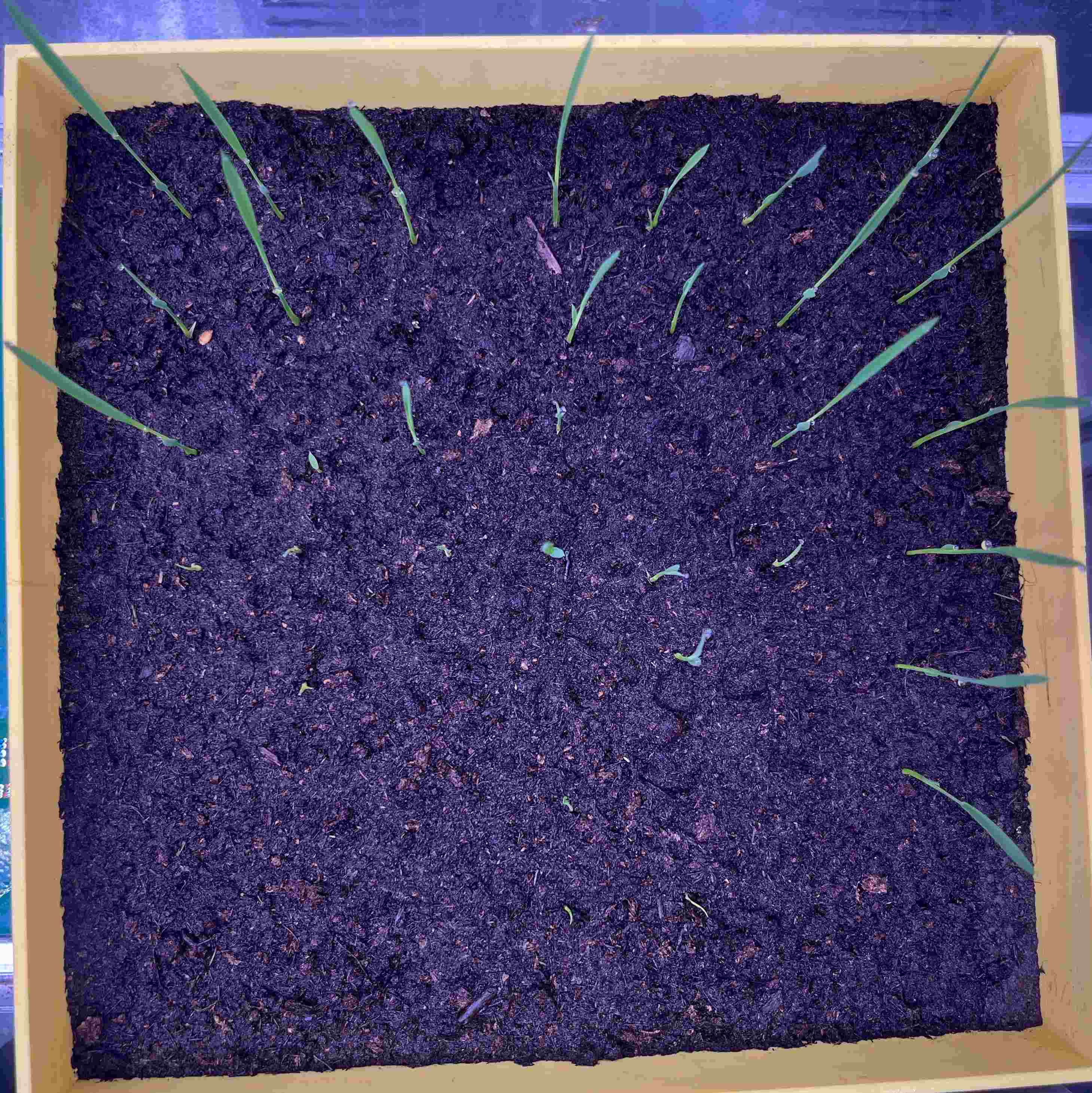

Supplement: Supplementary file 2 [file DataSheet2.zip › test/12-5.JPG]

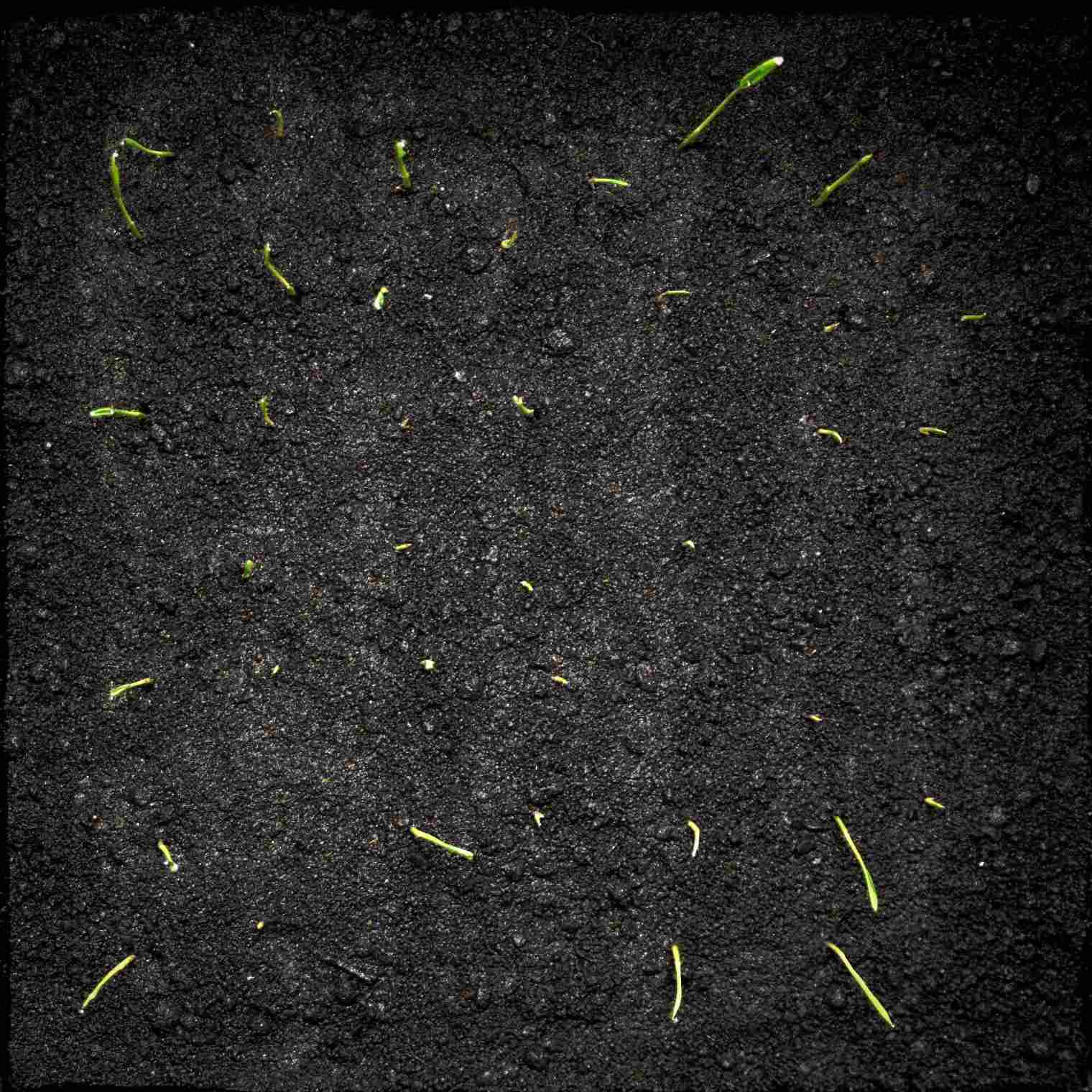

Supplement: Supplementary file 2 [file DataSheet2.zip › test/200-2024-3-19-0-29-2.JPG]

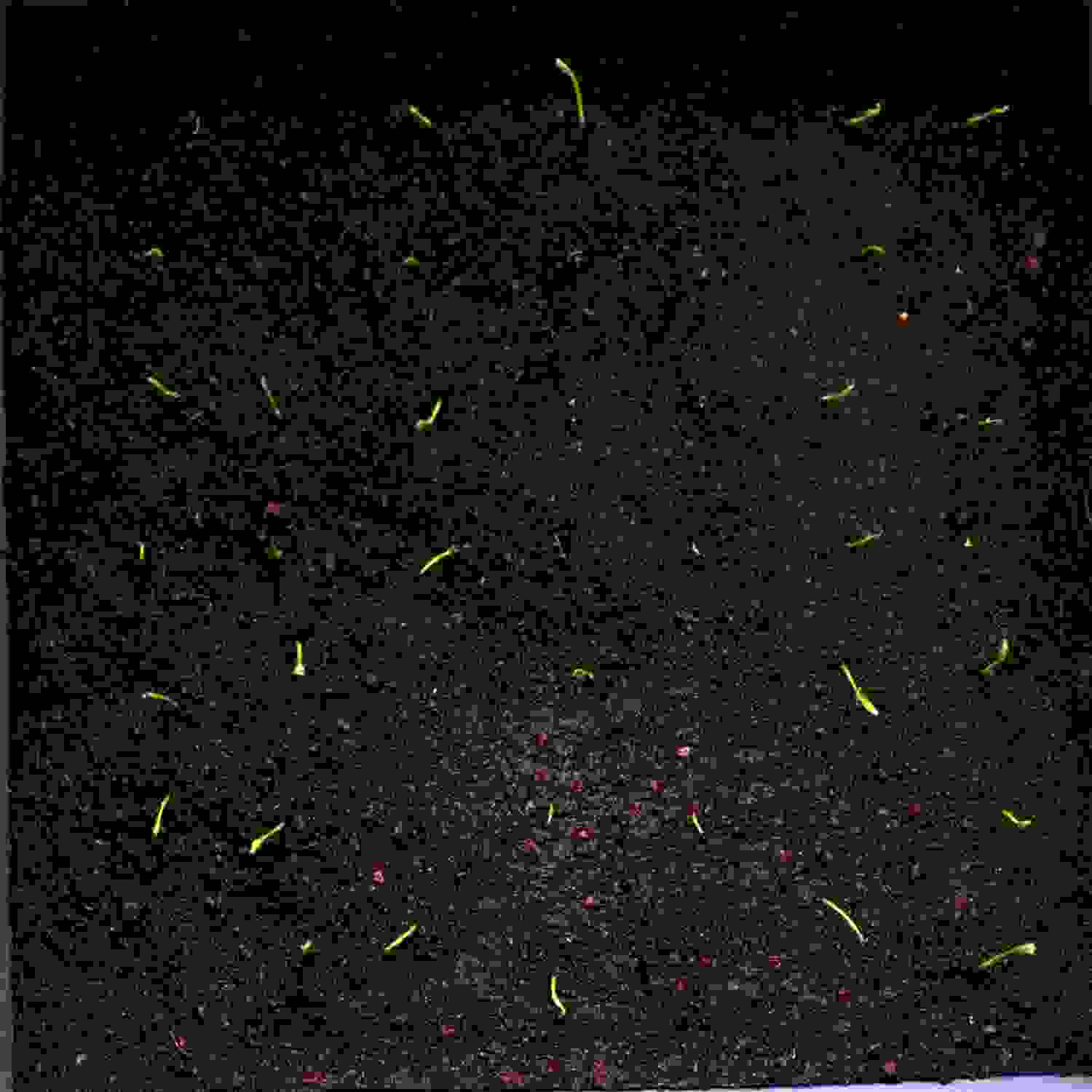

Supplement: Supplementary file 2 [file DataSheet2.zip › test/2000-2024-3-18-18-21-49.JPG]

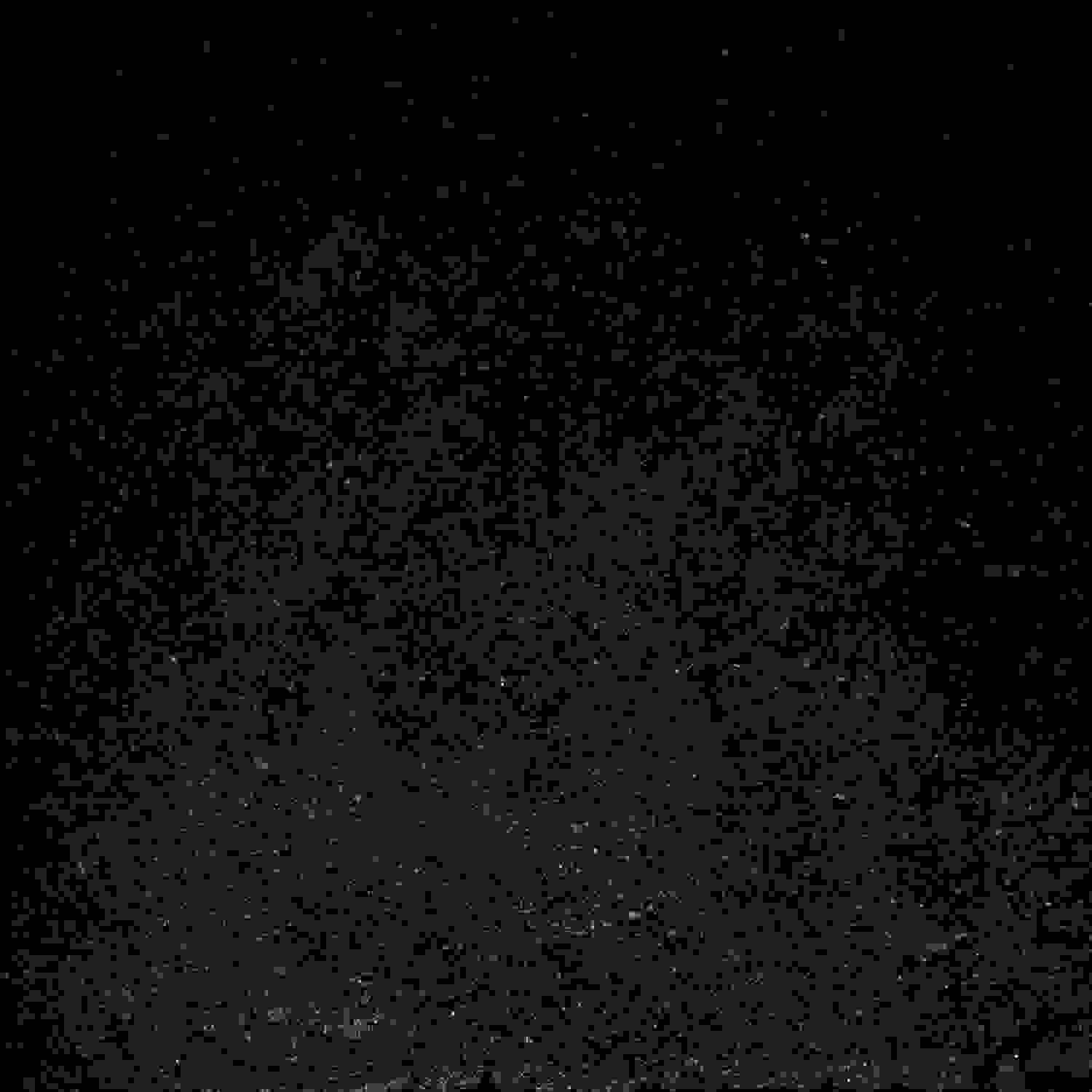

Supplement: Supplementary file 2 [file DataSheet2.zip › test/200120-2024-3-18-20-57-5.JPG]

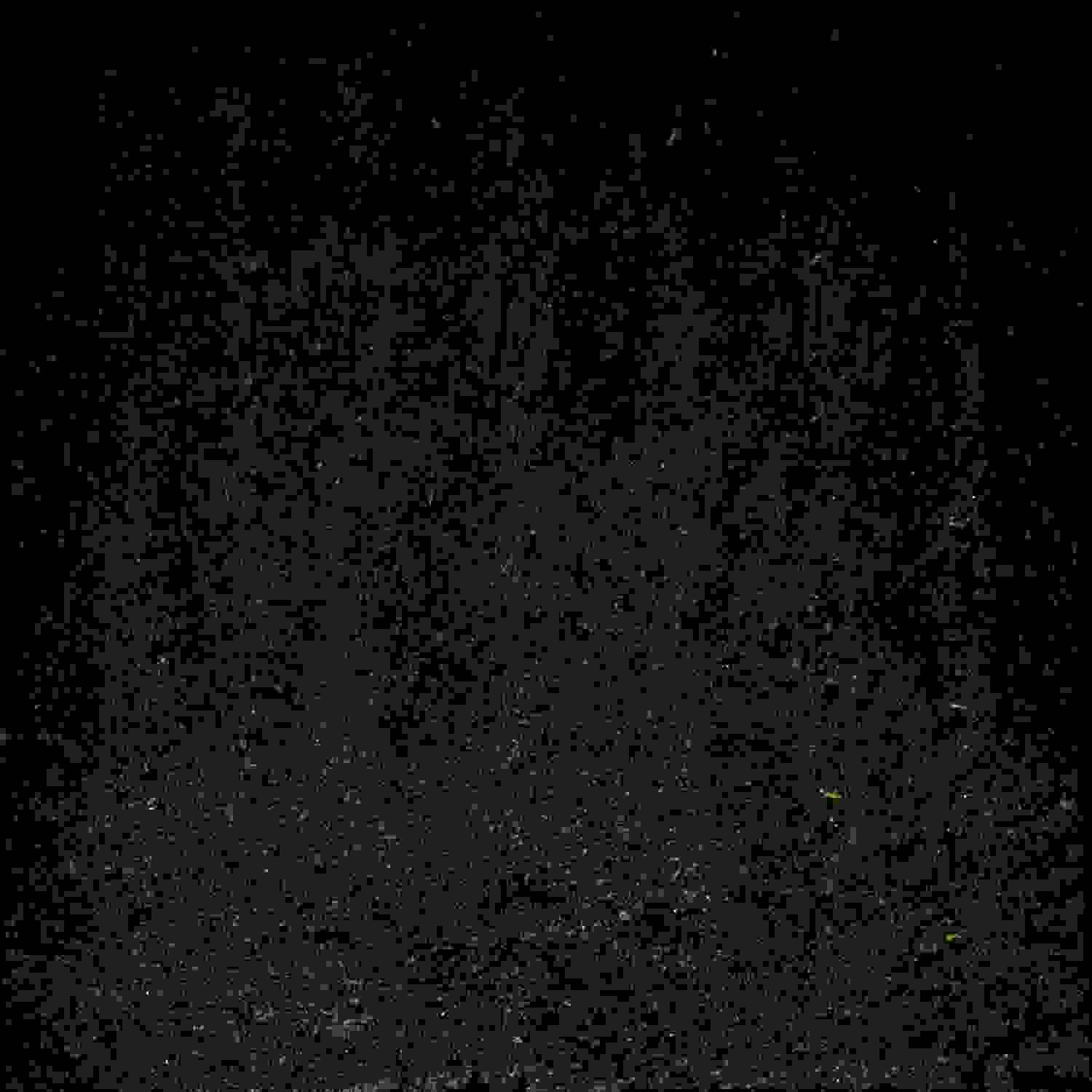

Supplement: Supplementary file 2 [file DataSheet2.zip › test/200120-2024-3-19-17-14-56.JPG]

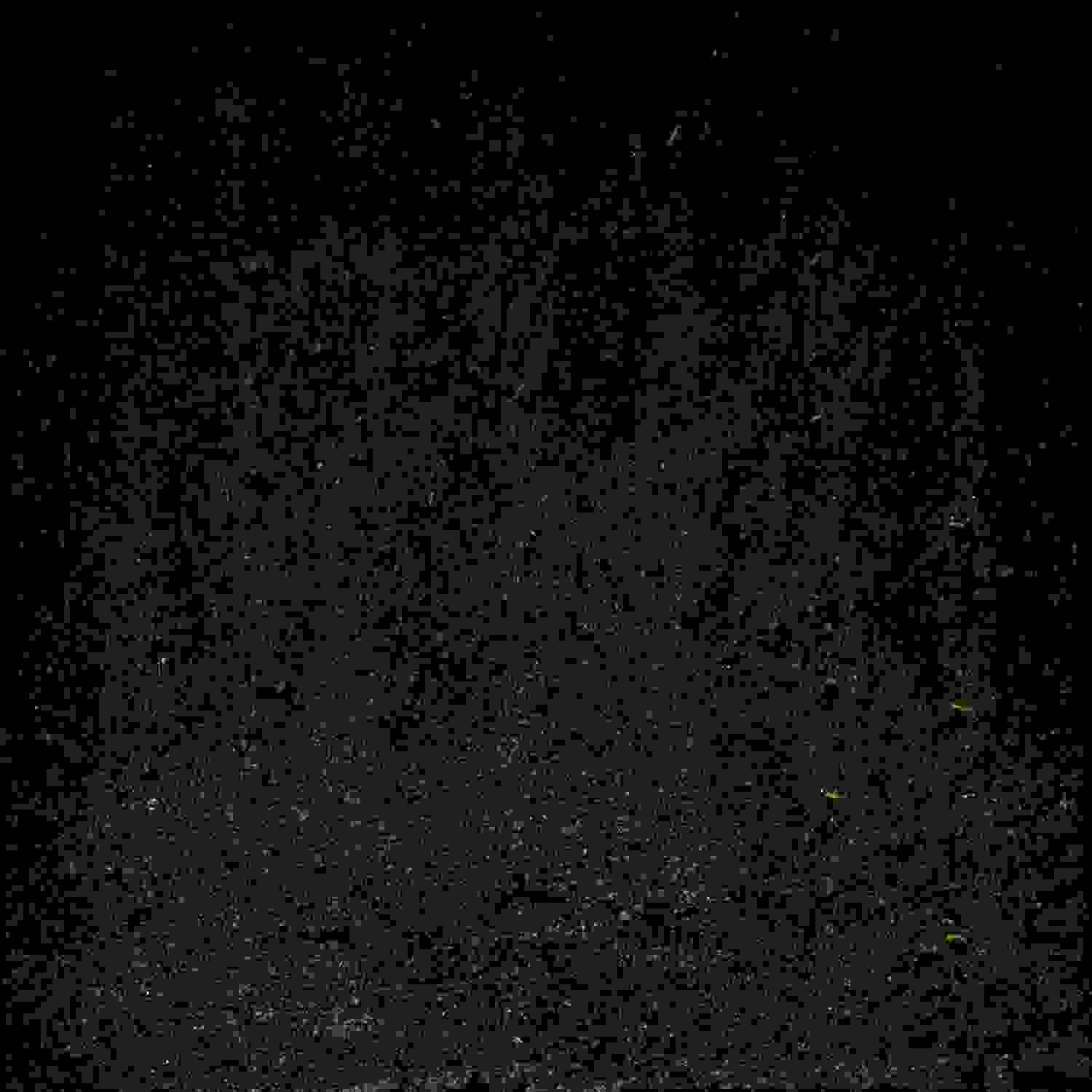

Supplement: Supplementary file 2 [file DataSheet2.zip › test/200120-2024-3-19-22-18-53.JPG]

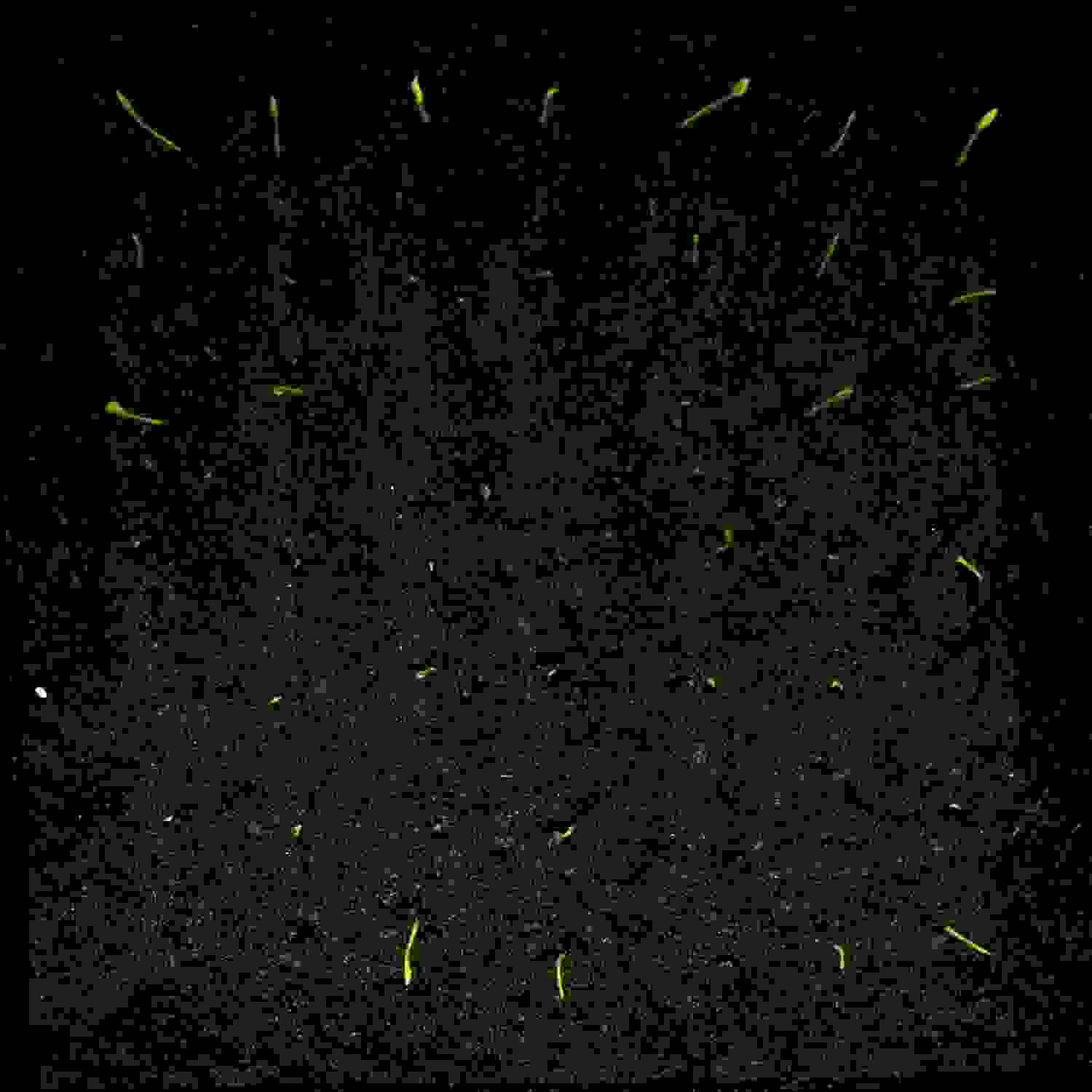

Supplement: Supplementary file 2 [file DataSheet2.zip › test/20030-2024-3-19-19-45-21.JPG]

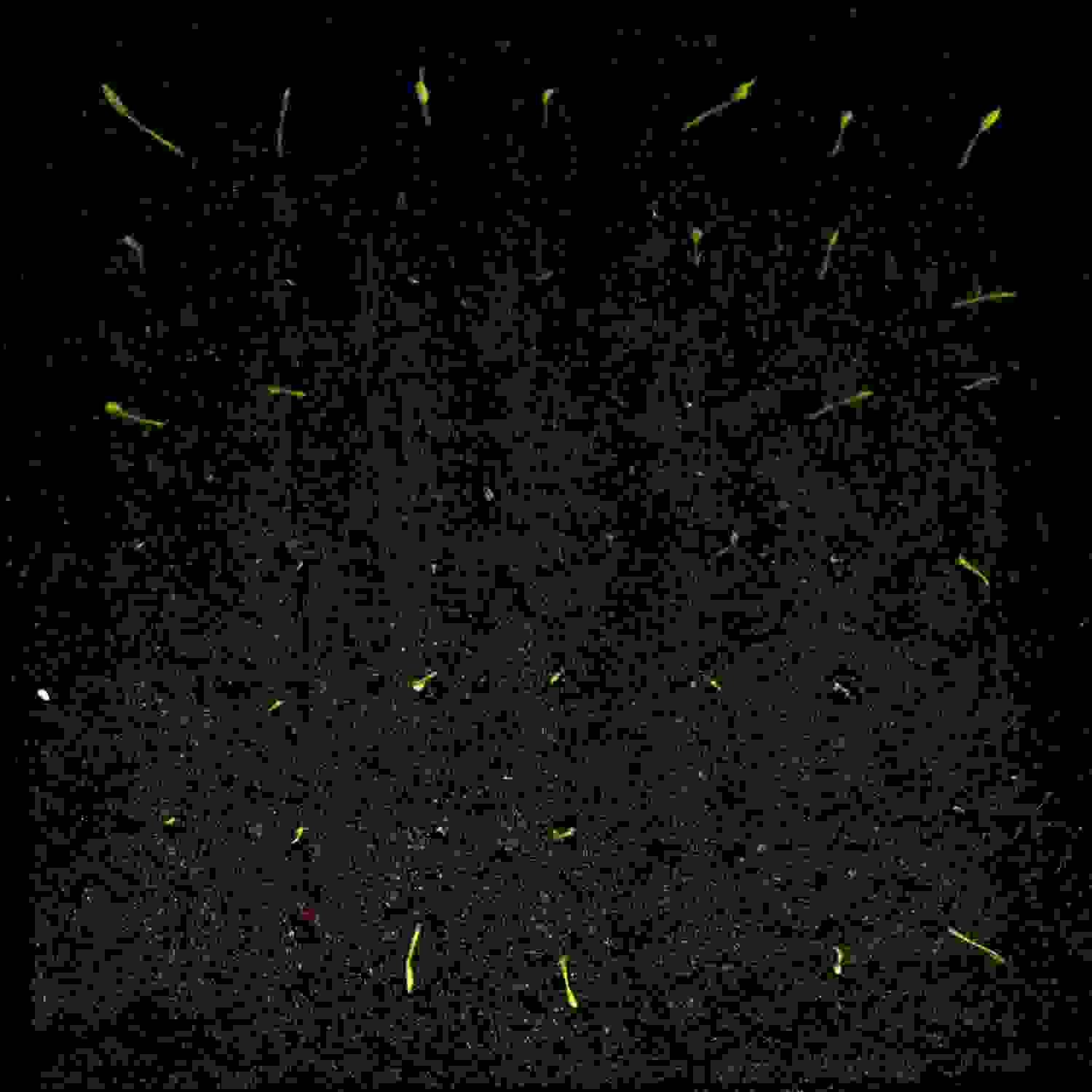

Supplement: Supplementary file 2 [file DataSheet2.zip › test/20030-2024-3-20-5-54-9.JPG]

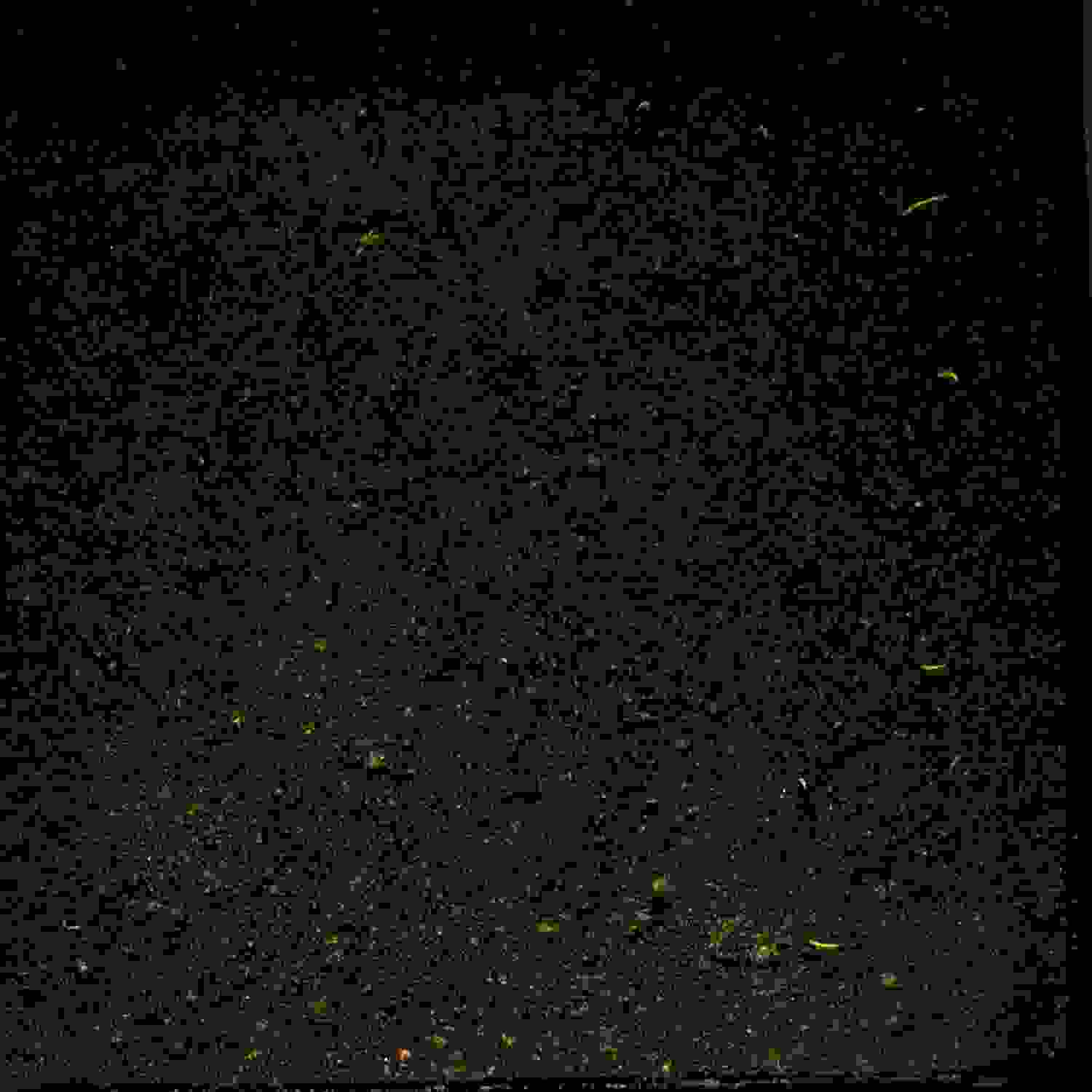

Supplement: Supplementary file 2 [file DataSheet2.zip › test/20090-2024-3-18-15-30-39.JPG]

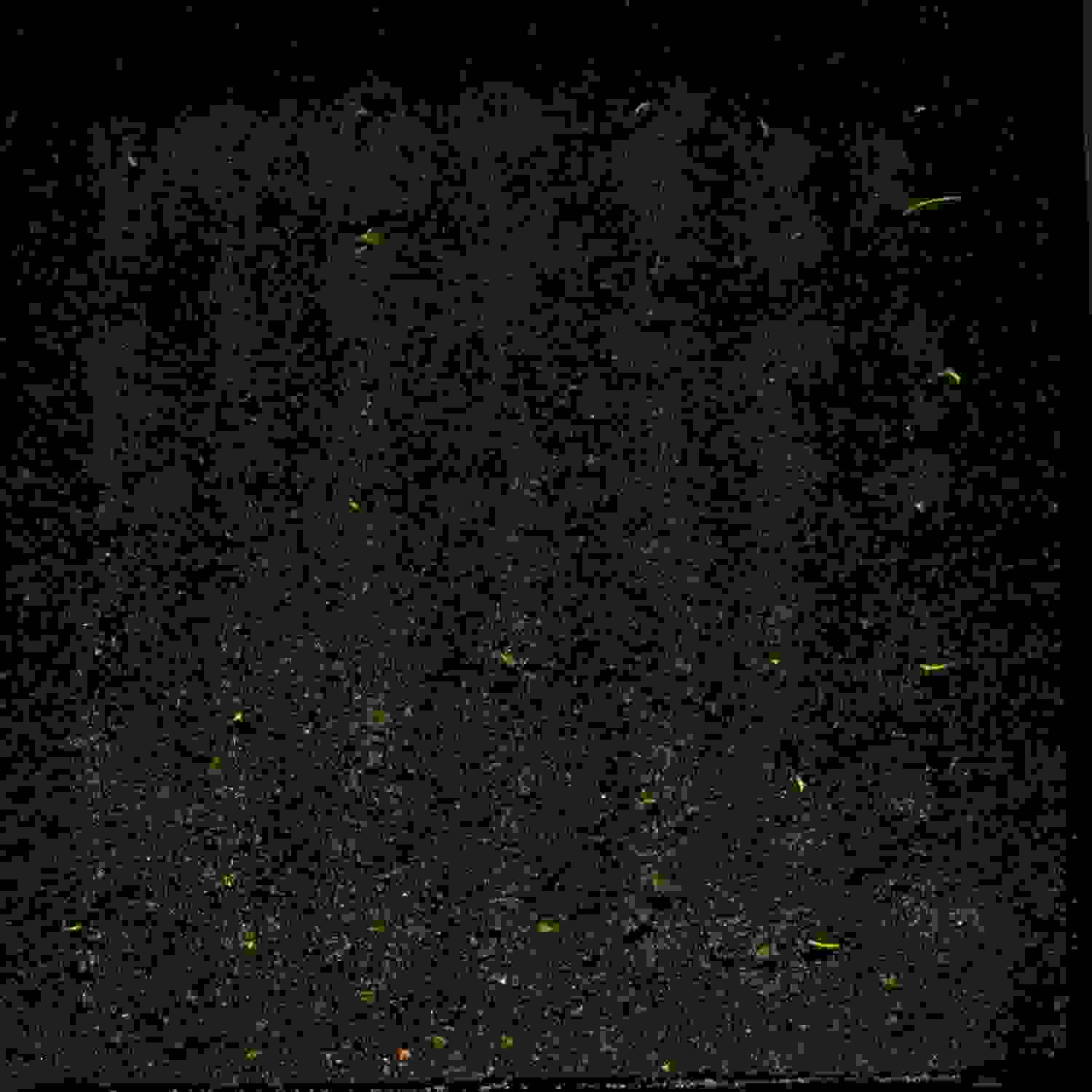

Supplement: Supplementary file 2 [file DataSheet2.zip › test/20090-2024-3-18-23-10-4.JPG]

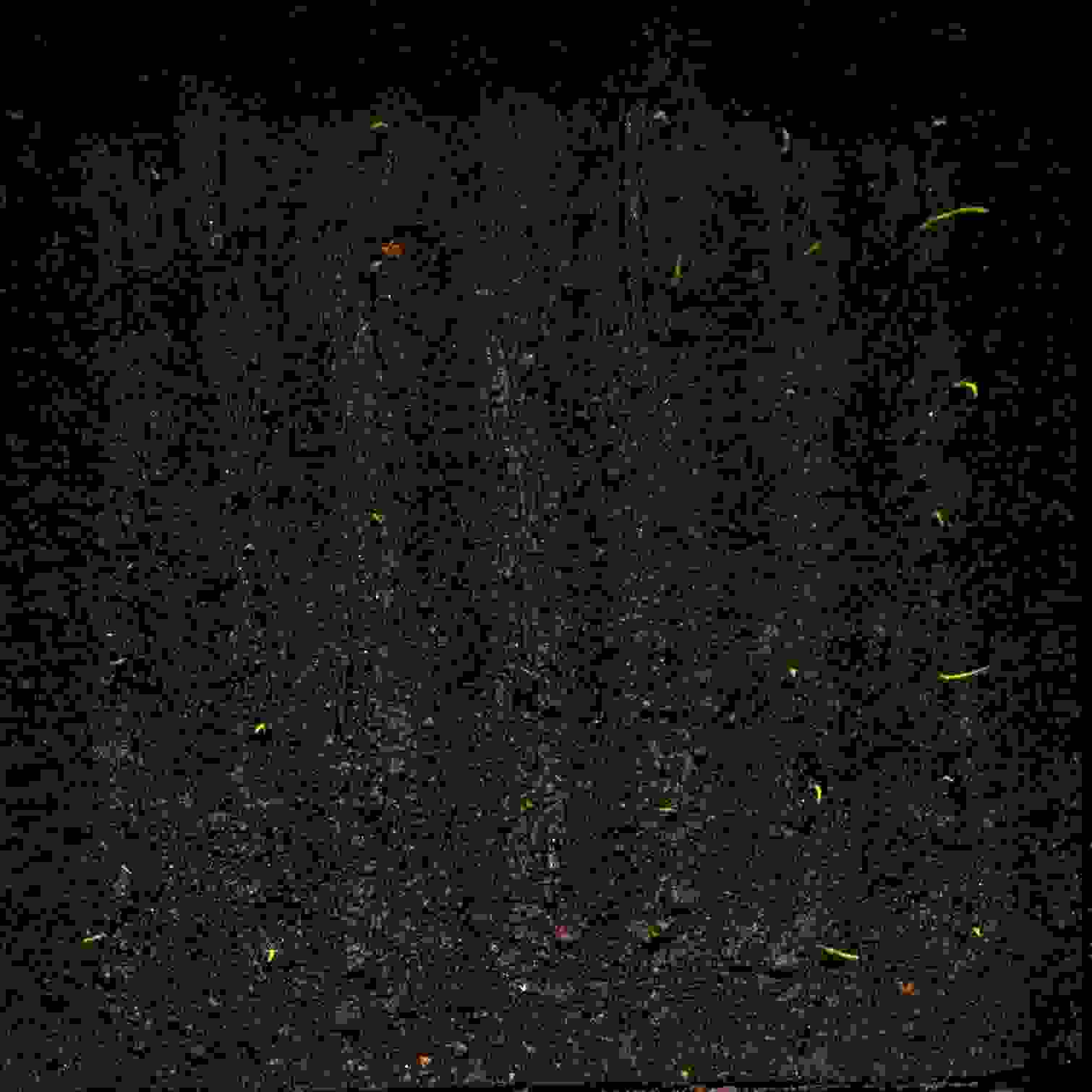

Supplement: Supplementary file 2 [file DataSheet2.zip › test/20090-2024-3-19-11-50-25.JPG]

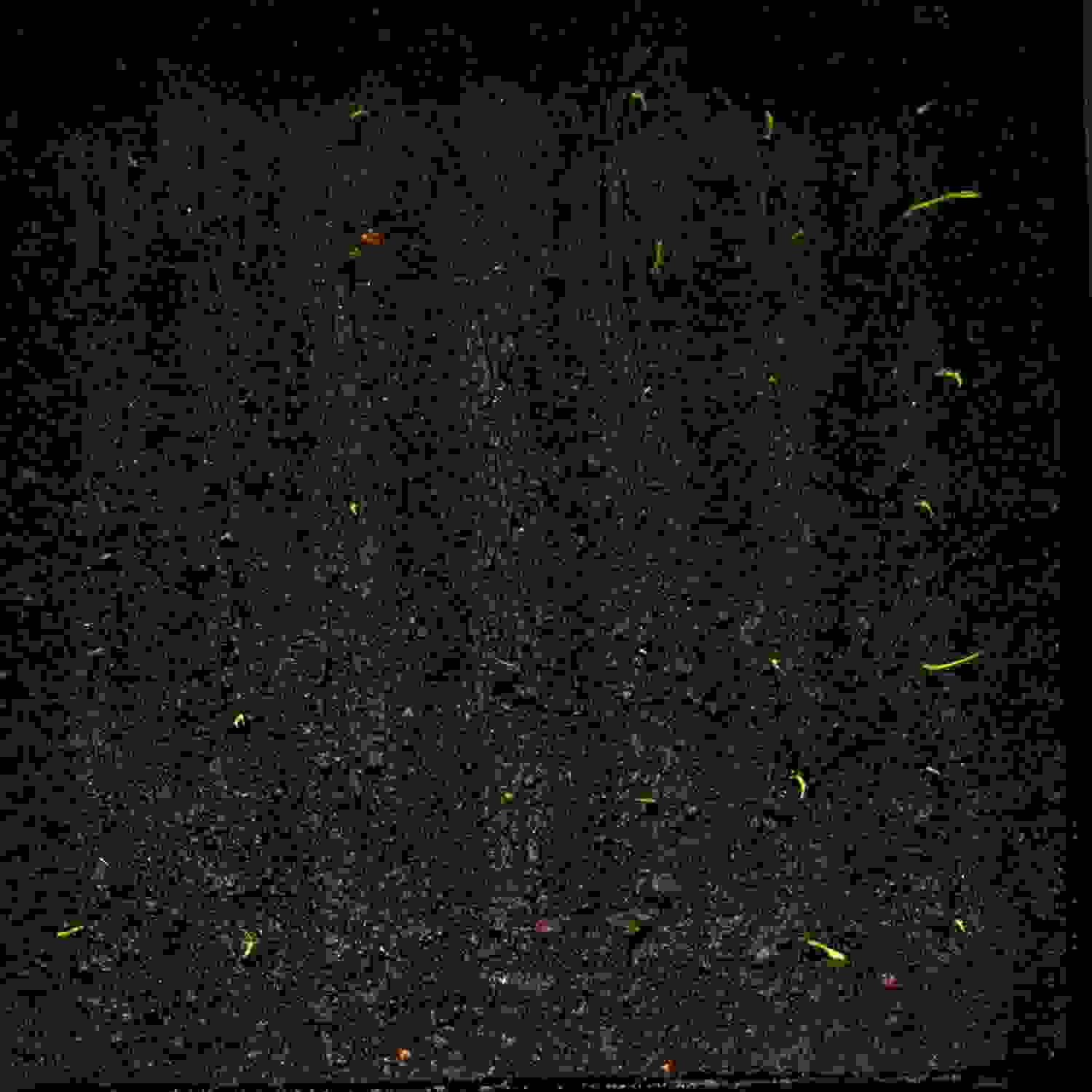

Supplement: Supplementary file 2 [file DataSheet2.zip › test/20090-2024-3-19-19-27-43.JPG]

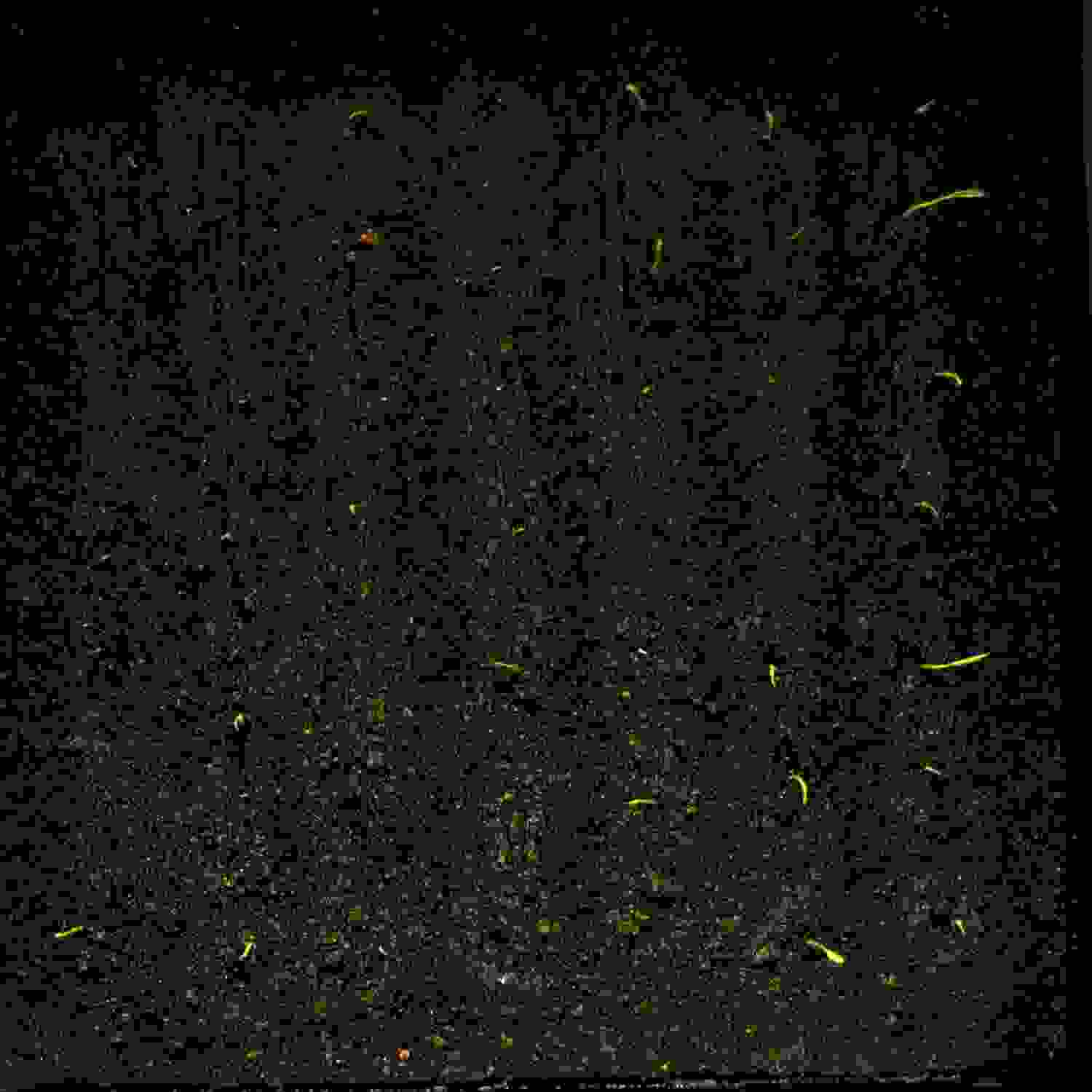

Supplement: Supplementary file 2 [file DataSheet2.zip › test/20090-2024-3-20-0-31-28.JPG]

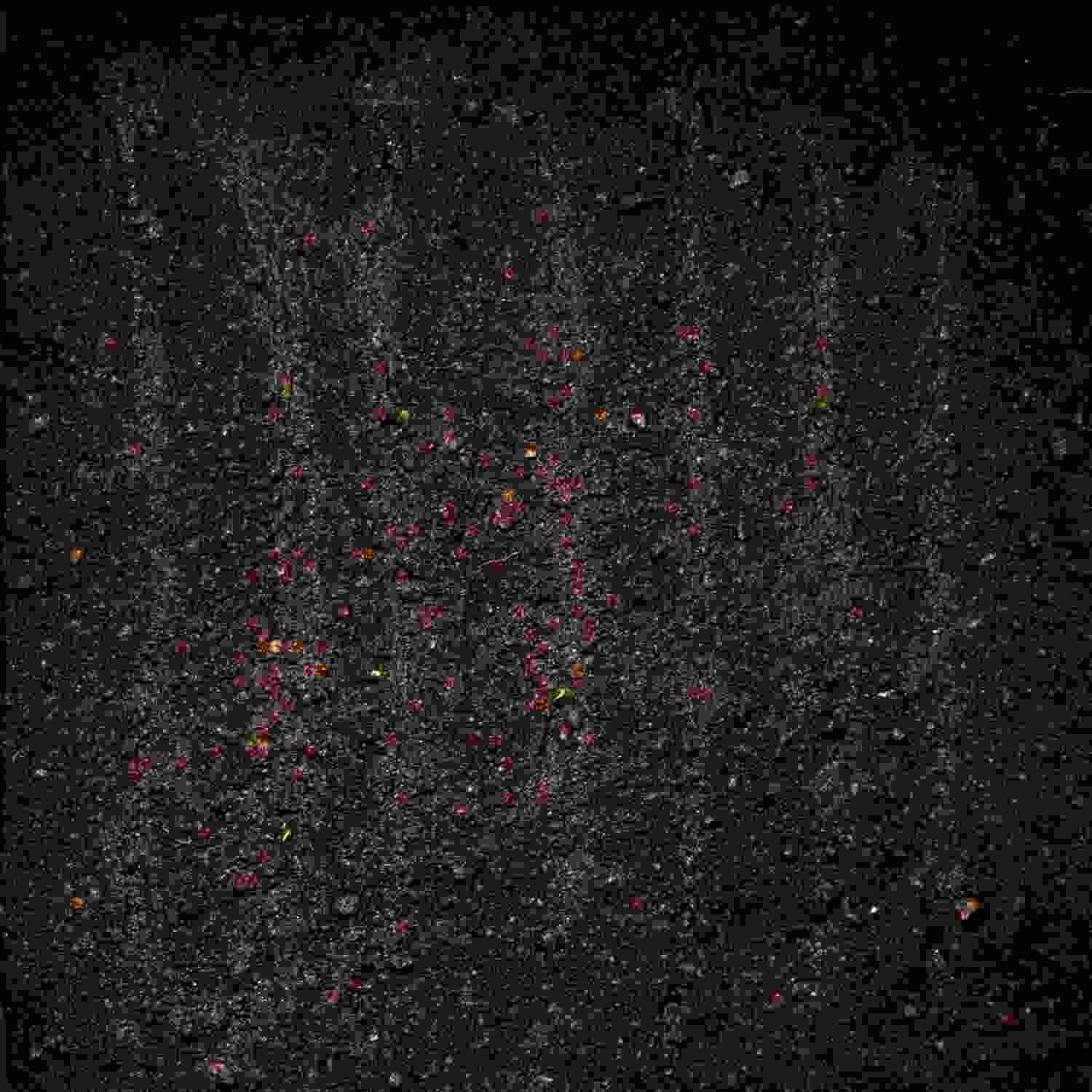

Supplement: Supplementary file 2 [file DataSheet2.zip › test/20120-2024-3-19-0-31-29.JPG]

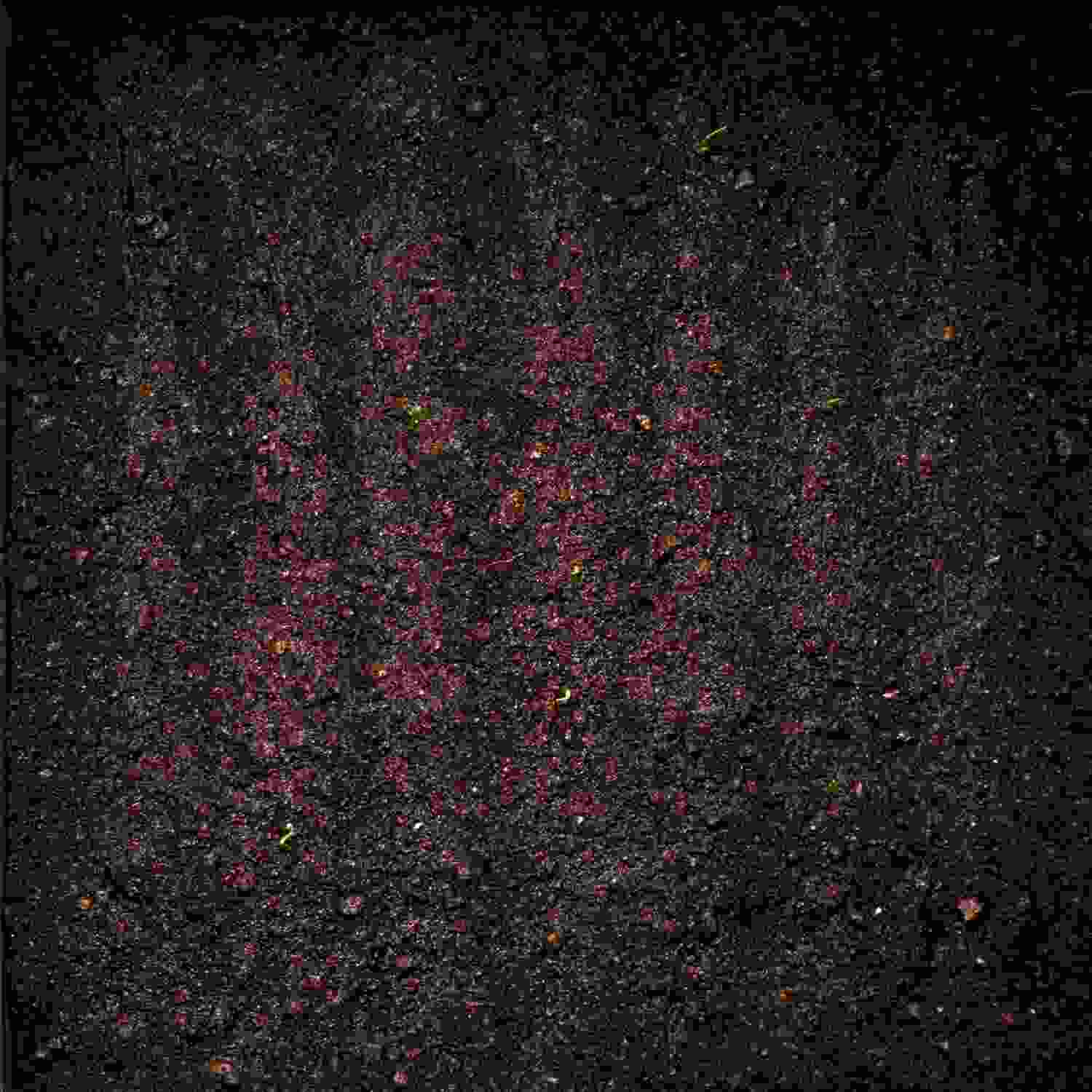

Supplement: Supplementary file 2 [file DataSheet2.zip › test/20120-2024-3-20-5-5-36.JPG]

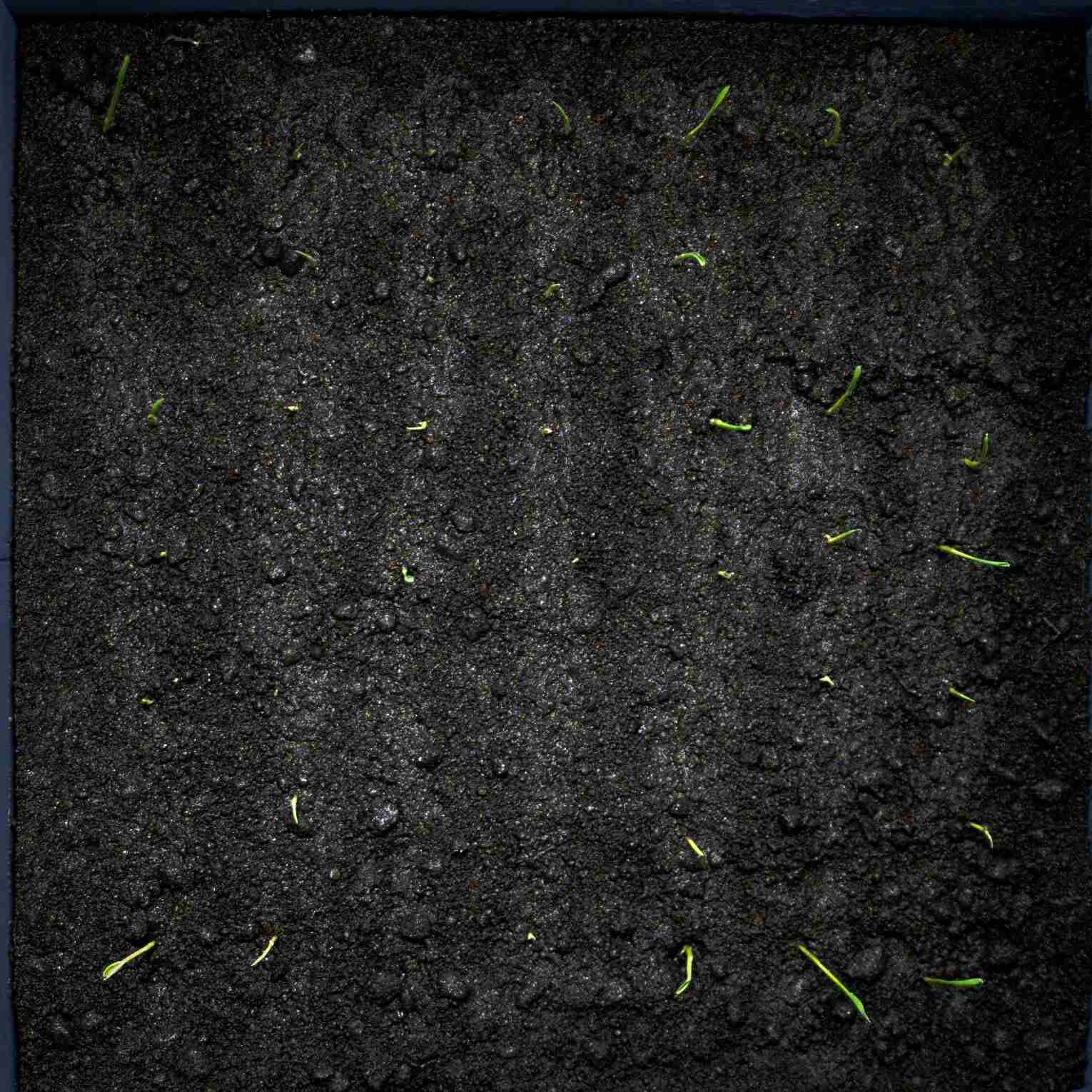

Supplement: Supplementary file 2 [file DataSheet2.zip › test/2030-2024-3-19-14-47-22.JPG]

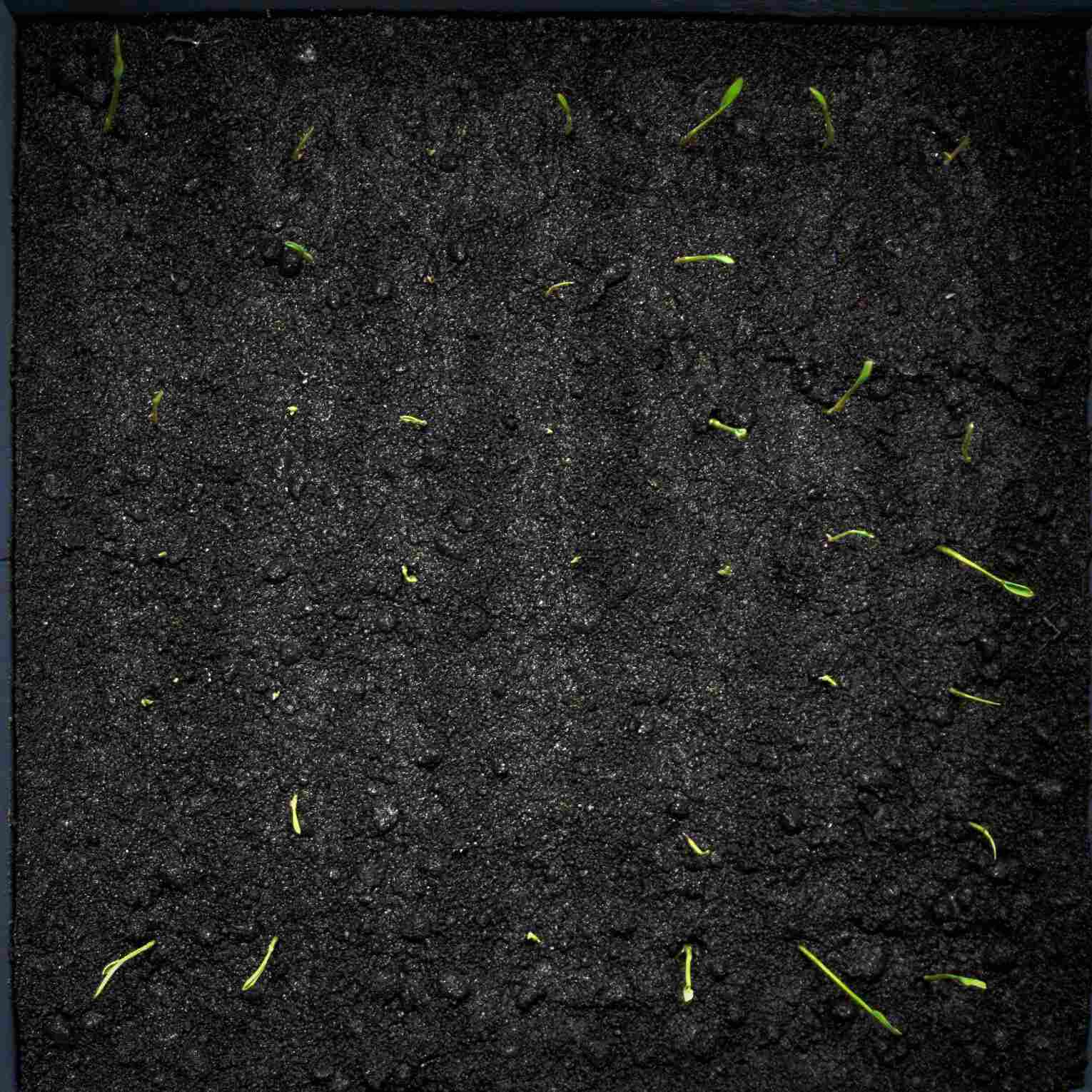

Supplement: Supplementary file 2 [file DataSheet2.zip › test/2030-2024-3-20-7-54-28.JPG]

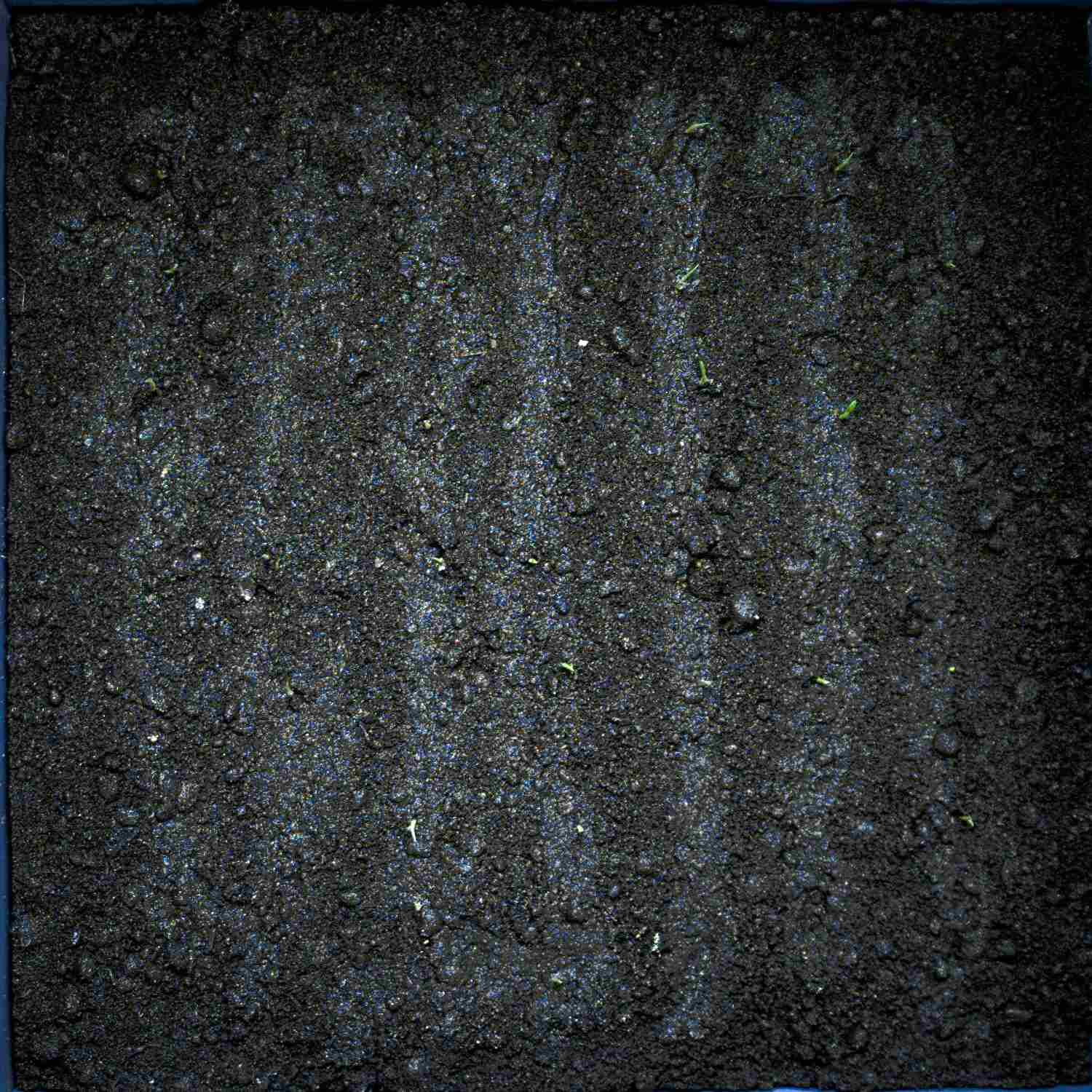

Supplement: Supplementary file 2 [file DataSheet2.zip › test/2060-2024-3-18-22-54-16.JPG]

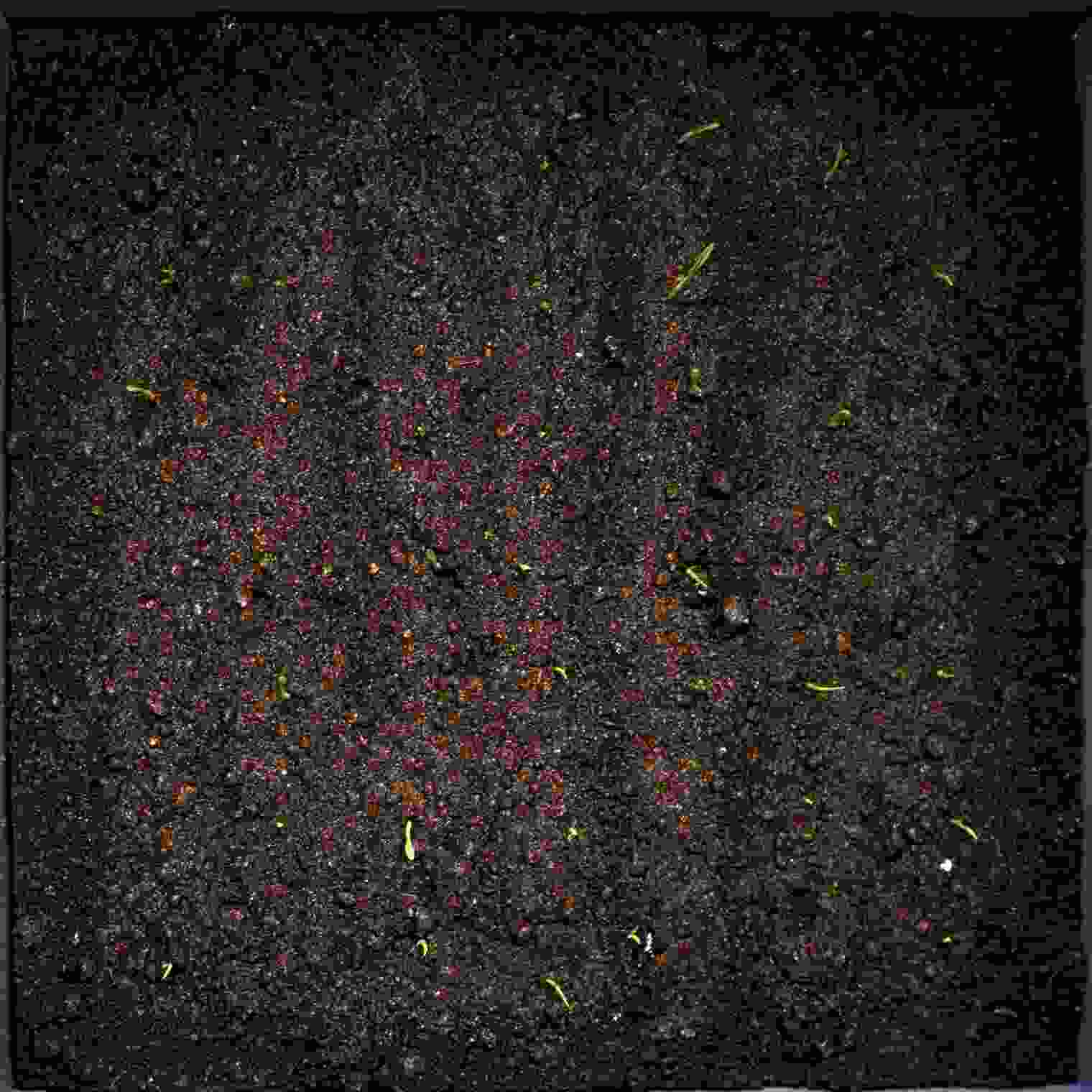

Supplement: Supplementary file 2 [file DataSheet2.zip › test/2060-2024-3-20-5-23-15.JPG]

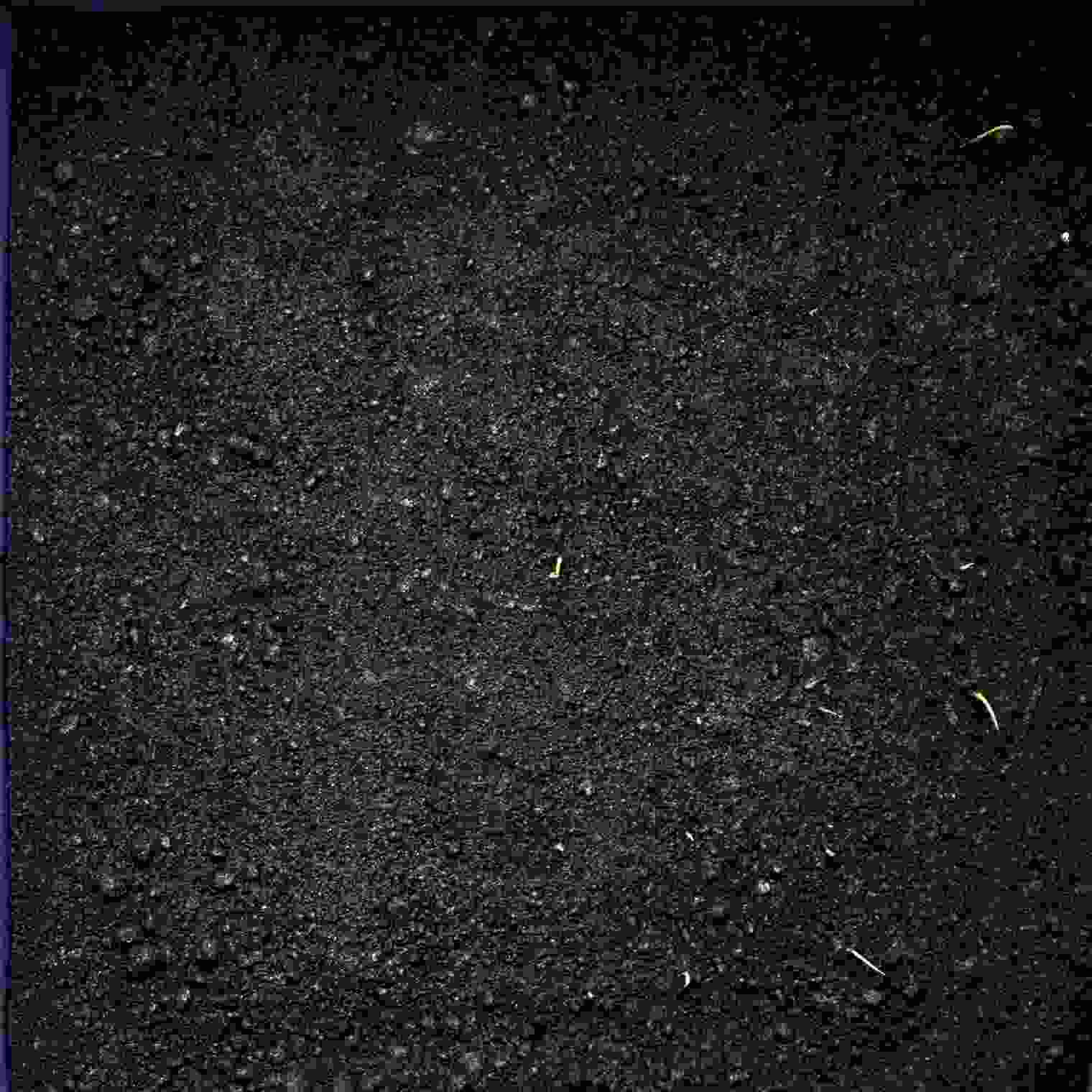

Supplement: Supplementary file 2 [file DataSheet2.zip › test/2090-2024-3-18-20-40-38.JPG]

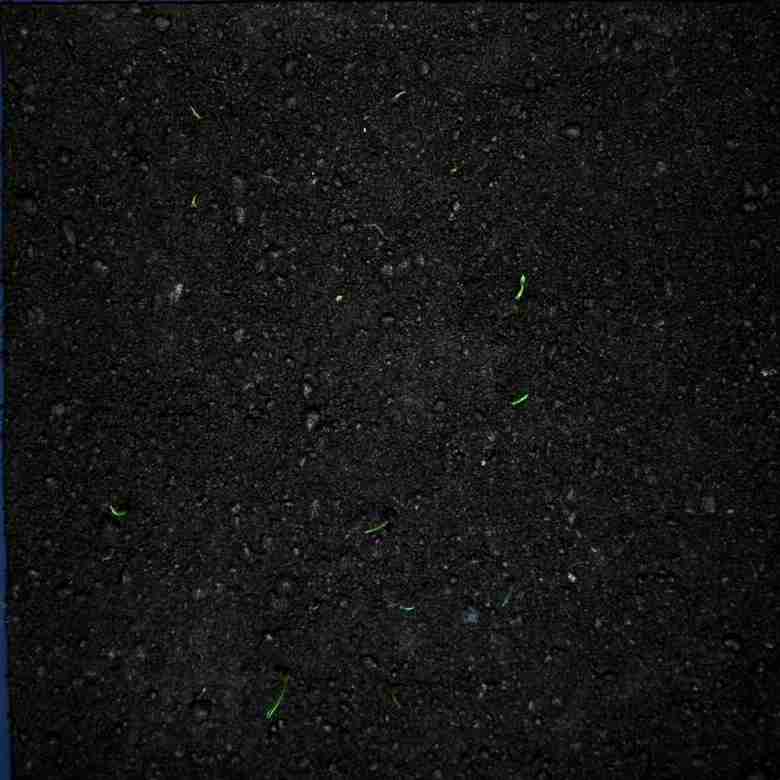

Supplement: Supplementary file 2 [file DataSheet2.zip › test/300120-2024-4-2-7-33-11.JPG]

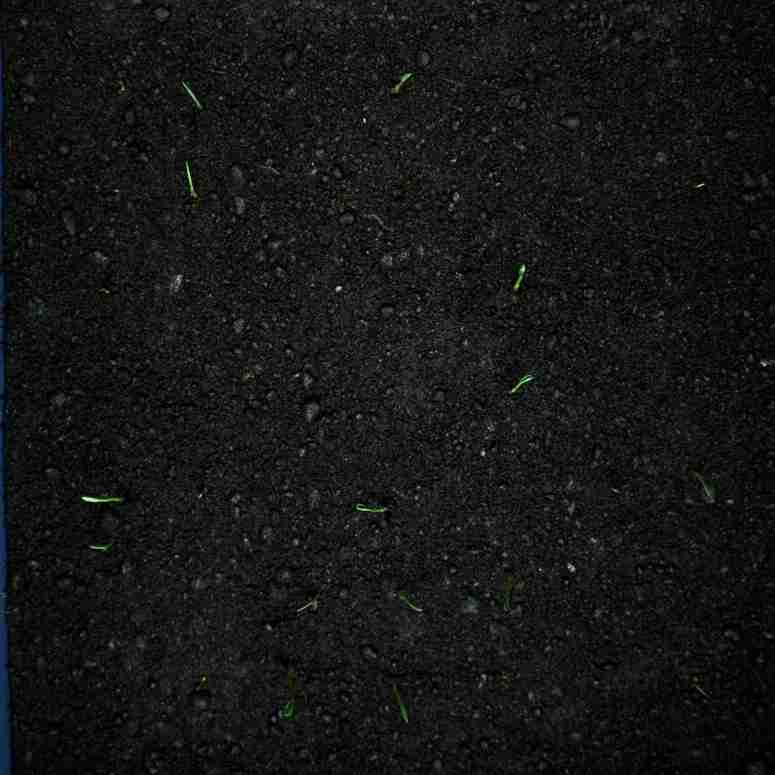

Supplement: Supplementary file 2 [file DataSheet2.zip › test/300120-2024-4-3-5-11-35.JPG]
