# Supplementary figures and images for: VanillaNet-YOLOv8 segment: detection of nano-iron oxide regulation on rice seedling growth vitality under salt stress (part 2 of 5)
Source: Front Plant Sci. 2025 Sep 17;16:1631279. doi: 10.3389/fpls.2025.1631279 (PMC12484053; doi:10.3389/fpls.2025.1631279)

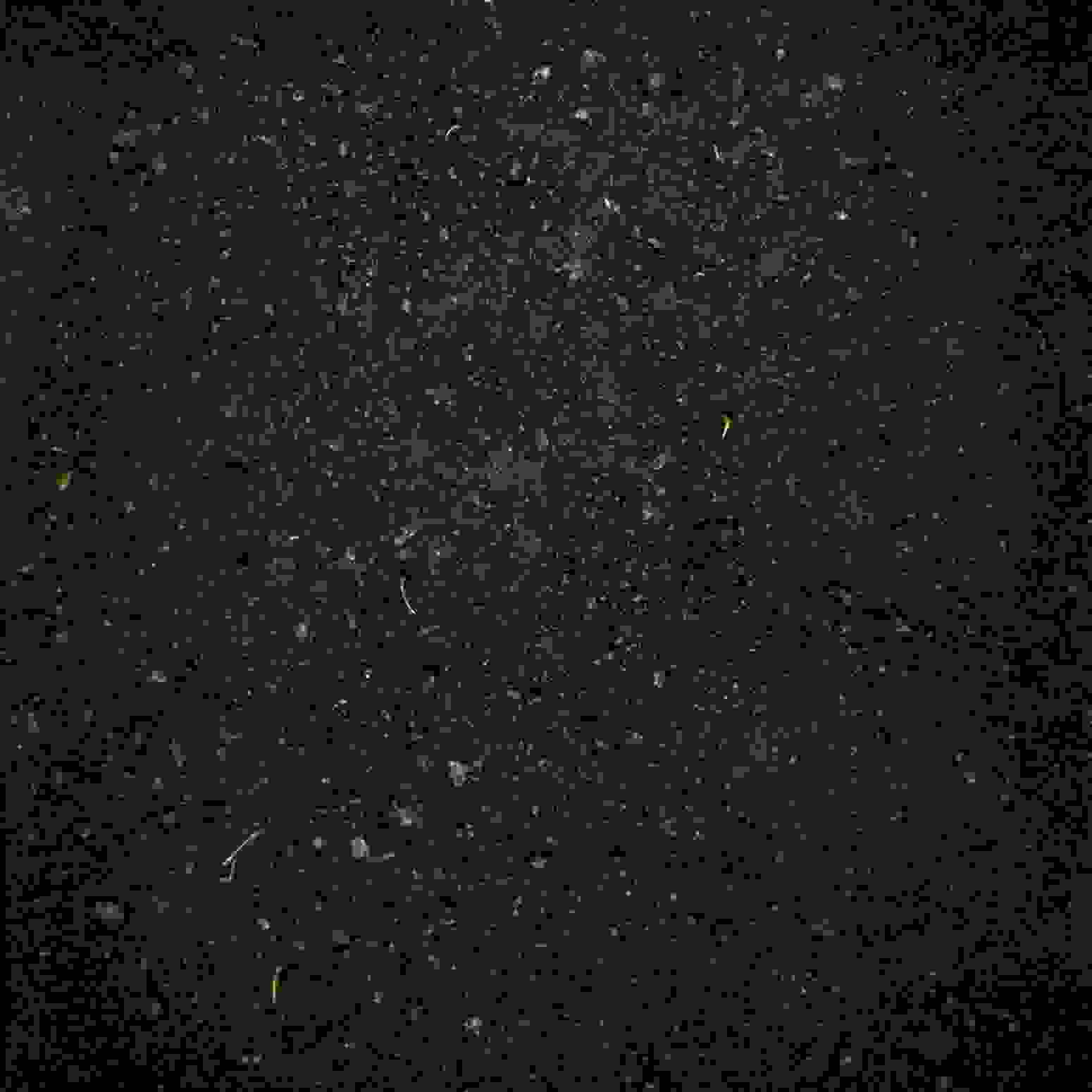

Supplement: Supplementary file 2 [file DataSheet2.zip › test/300150-2024-4-2-0-38-57.JPG]

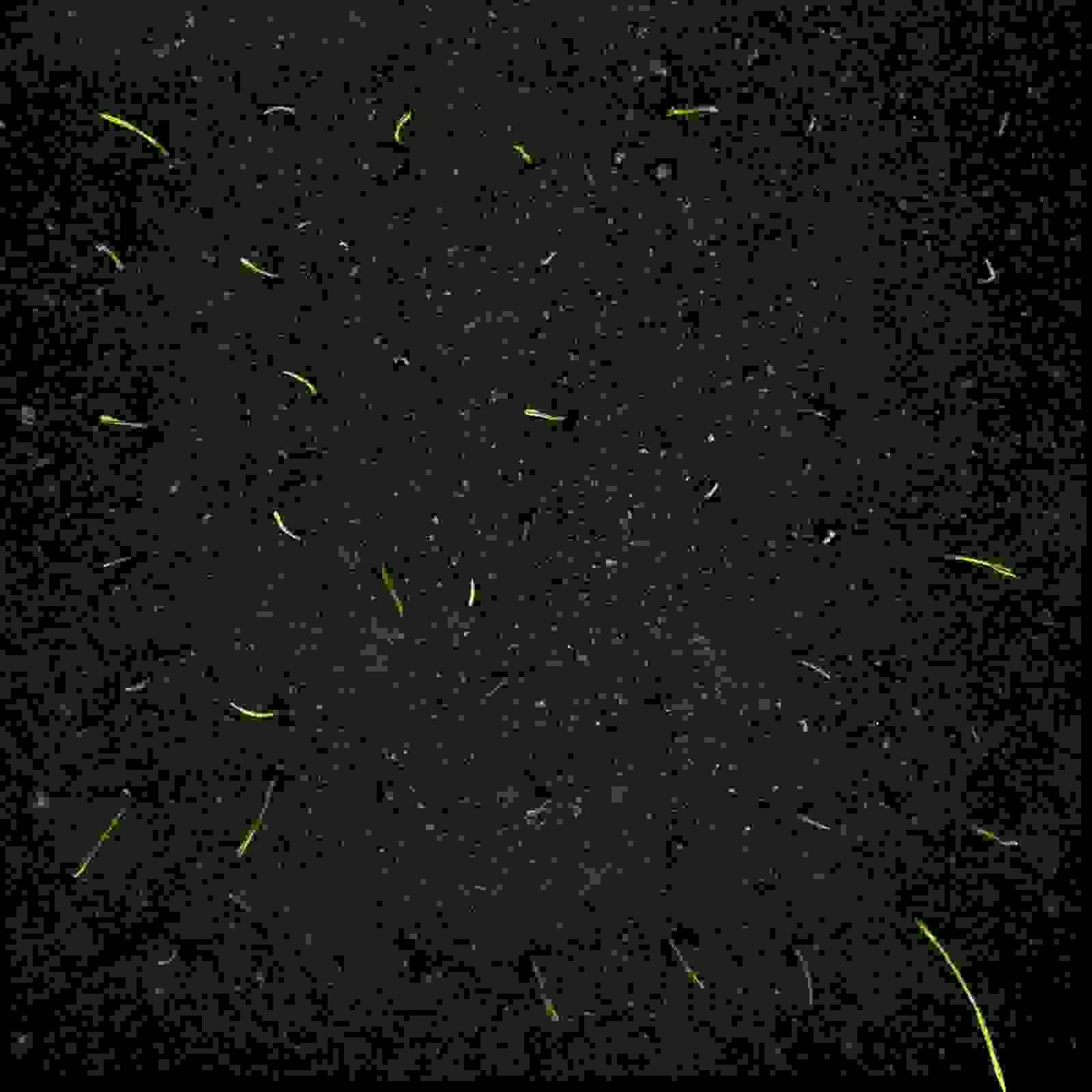

Supplement: Supplementary file 2 [file DataSheet2.zip › test/30030-2024-4-1-21-36-59.JPG]

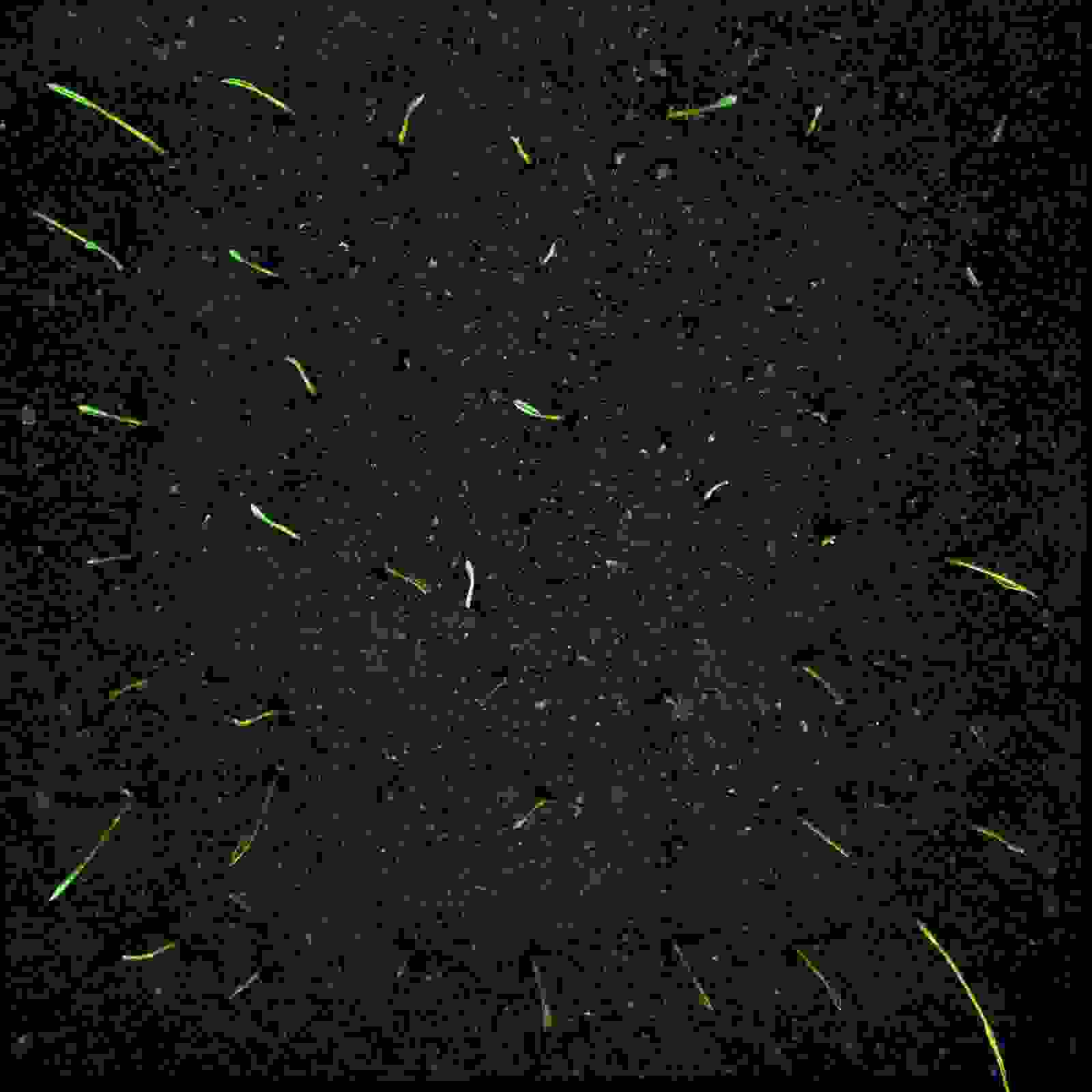

Supplement: Supplementary file 2 [file DataSheet2.zip › test/30030-2024-4-2-14-26-26.JPG]

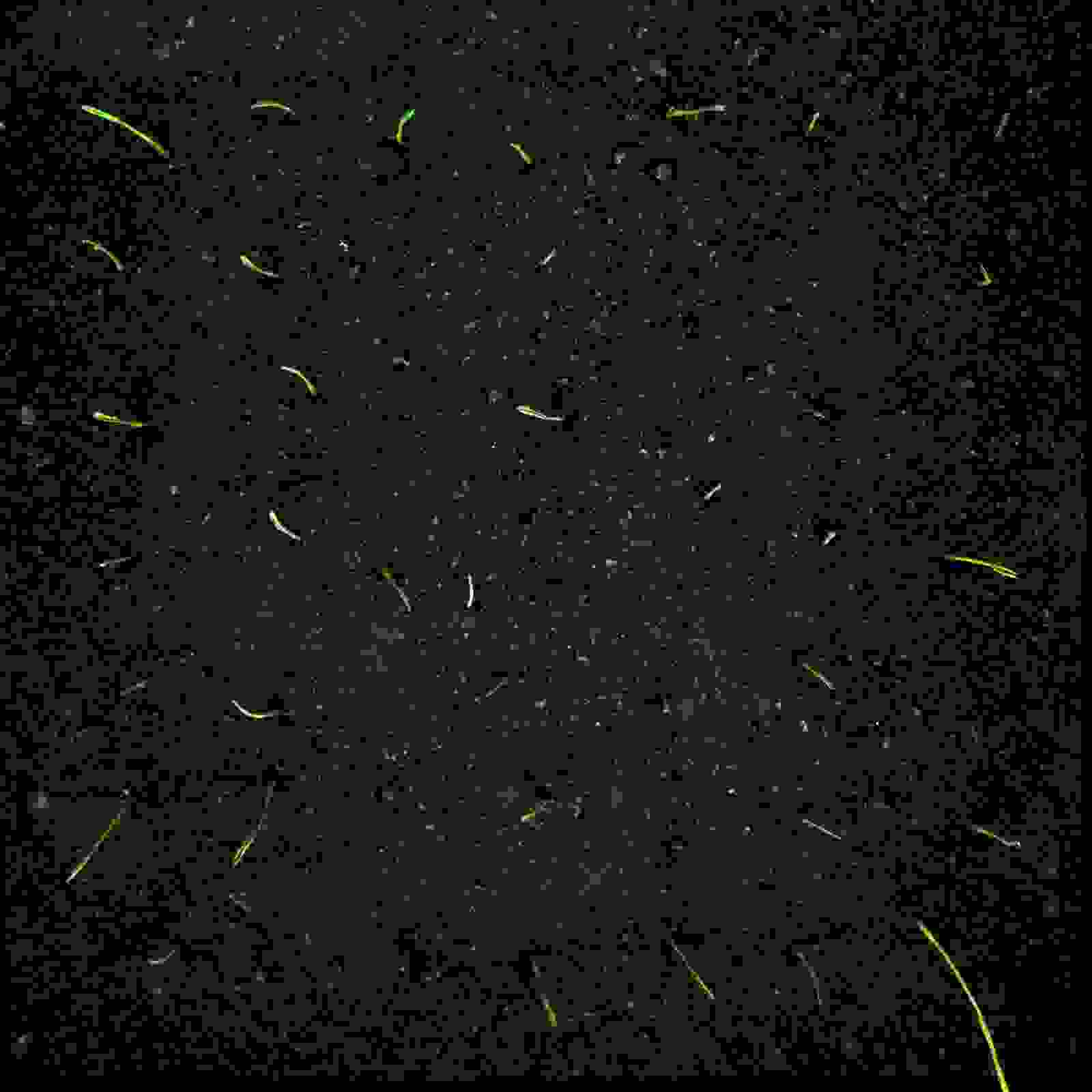

Supplement: Supplementary file 2 [file DataSheet2.zip › test/30030-2024-4-2-2-25-18.JPG]

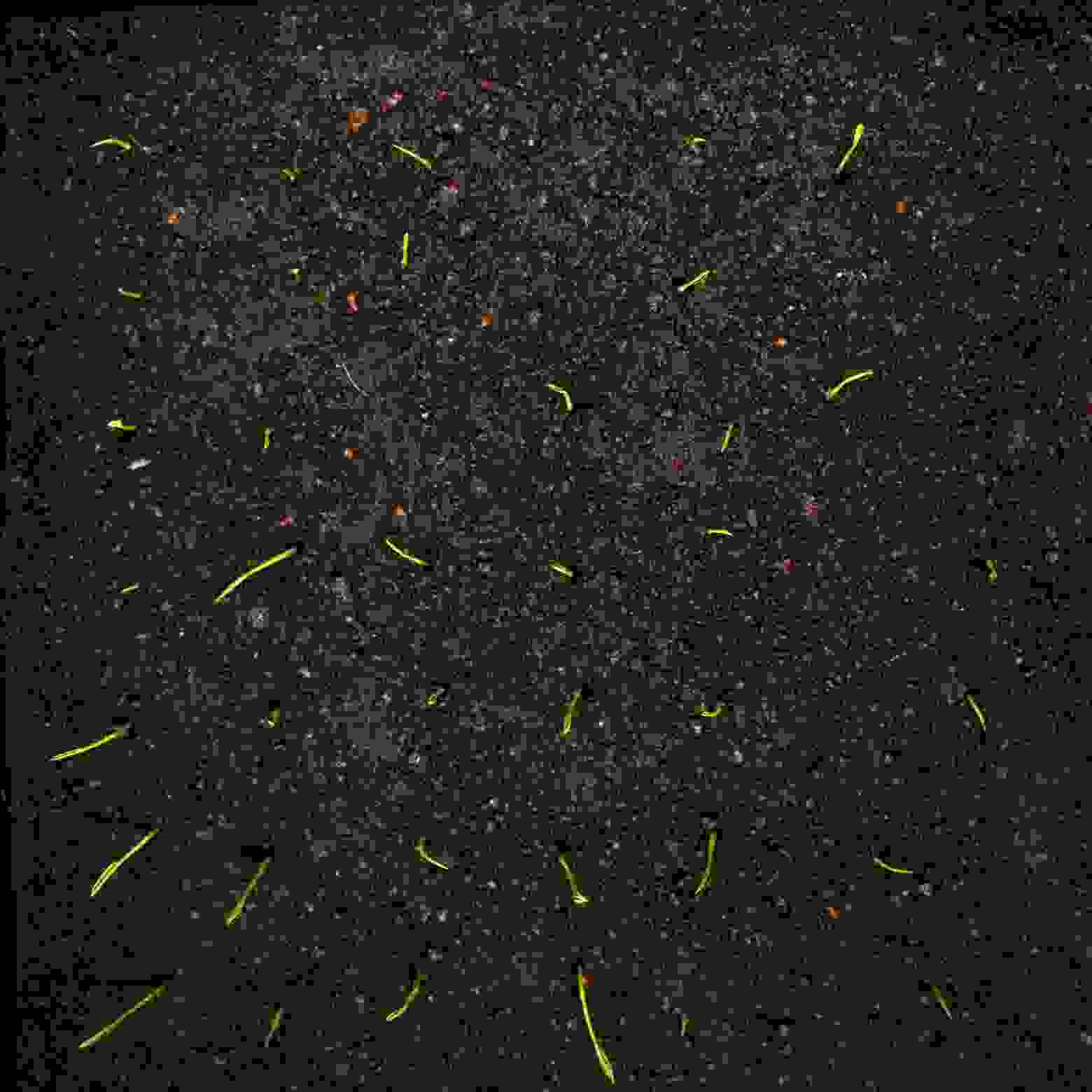

Supplement: Supplementary file 2 [file DataSheet2.zip › test/30060-2024-4-2-16-51-7.JPG]

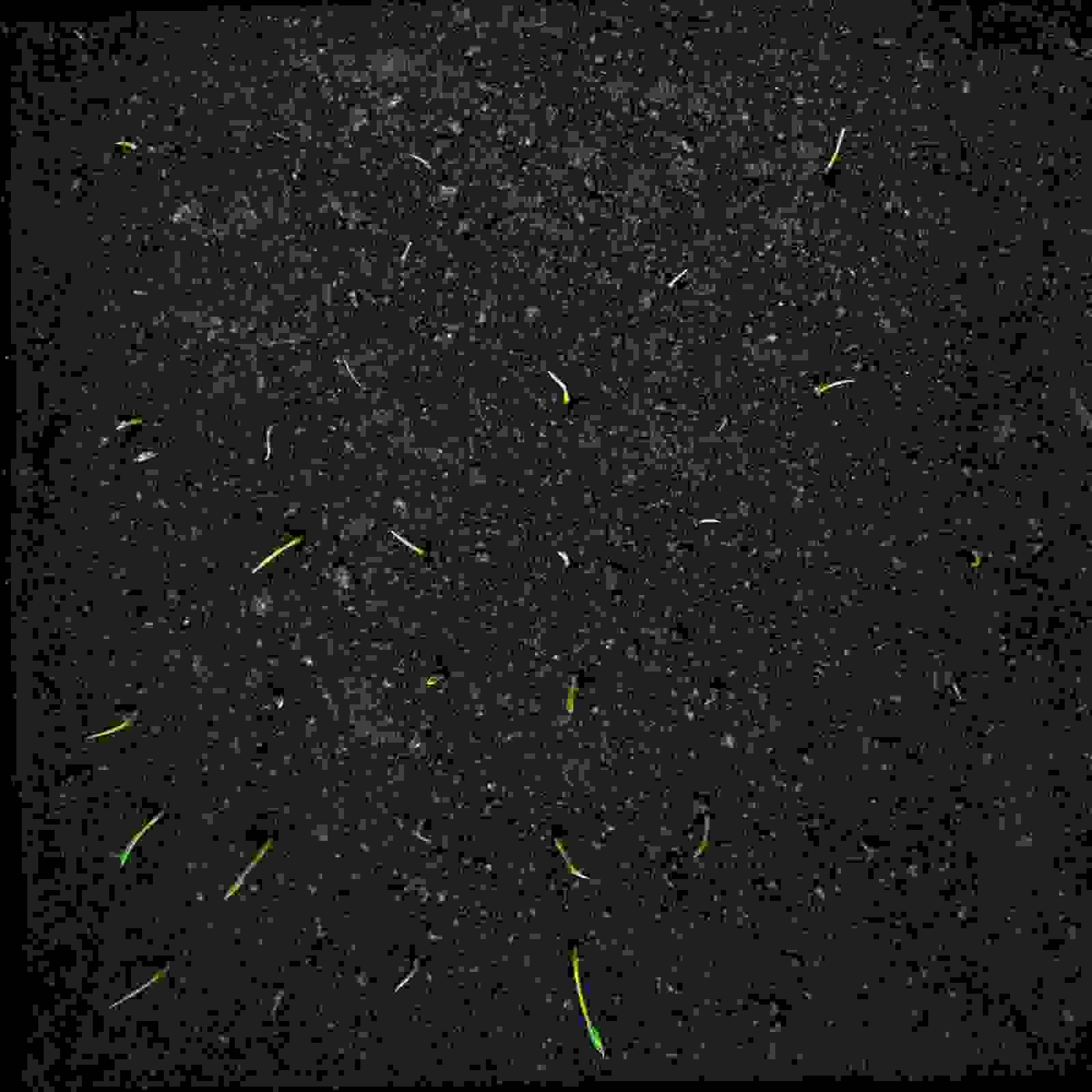

Supplement: Supplementary file 2 [file DataSheet2.zip › test/30060-2024-4-2-2-25-40.JPG]

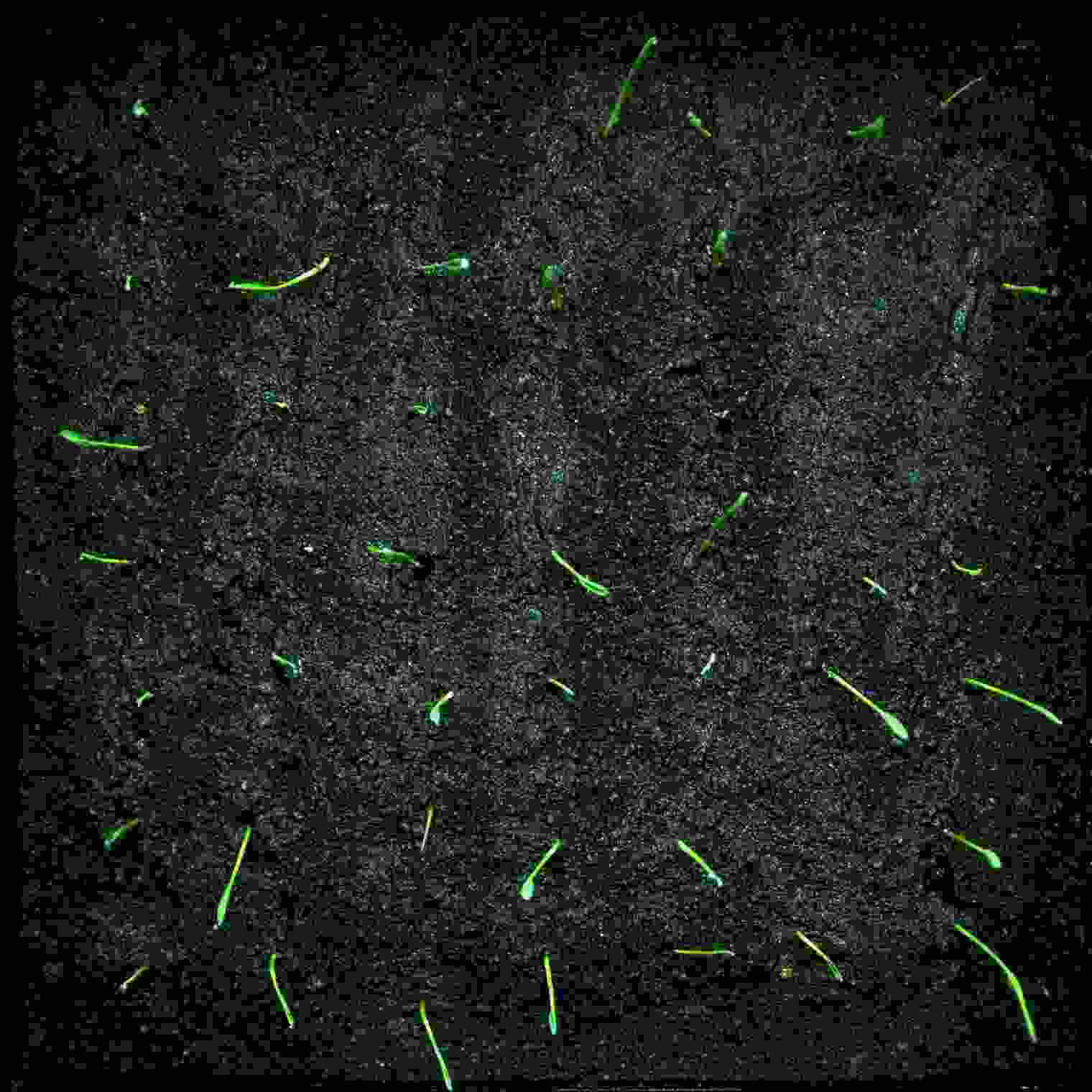

Supplement: Supplementary file 2 [file DataSheet2.zip › test/500-2024-3-19-13-25-18.JPG]

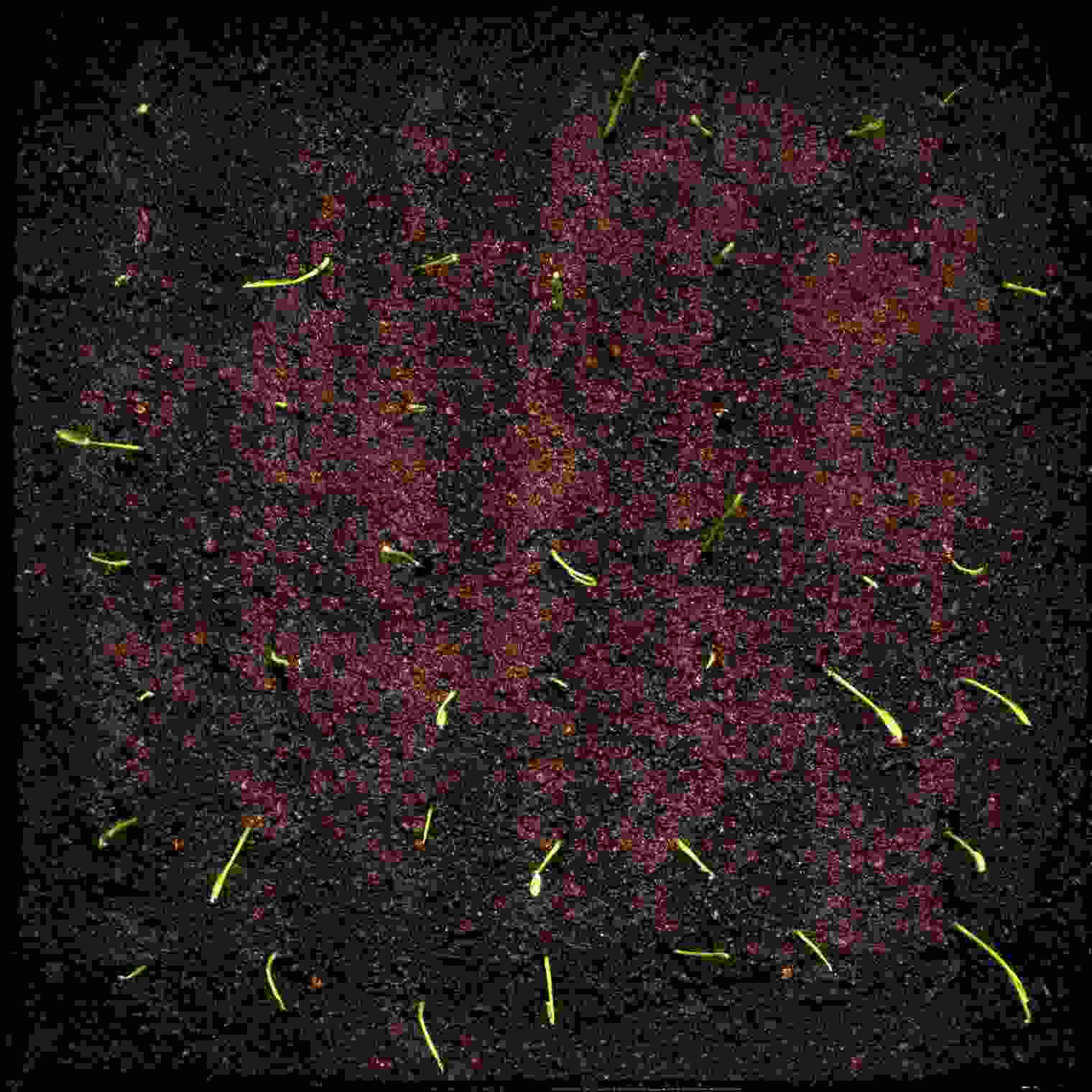

Supplement: Supplementary file 2 [file DataSheet2.zip › test/500-2024-3-19-2-14-20.JPG]

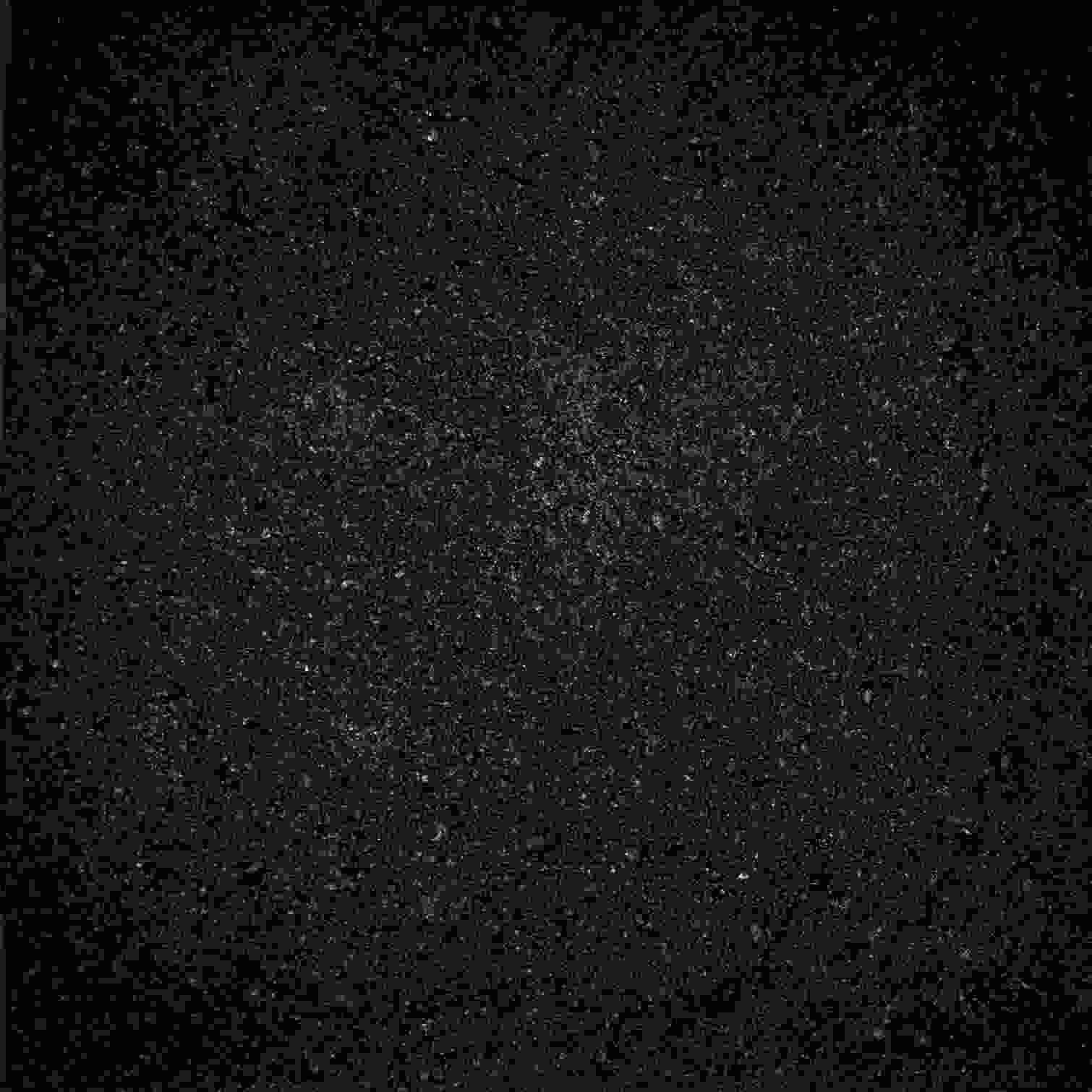

Supplement: Supplementary file 2 [file DataSheet2.zip › test/50150-2024-3-18-20-4-9.JPG]

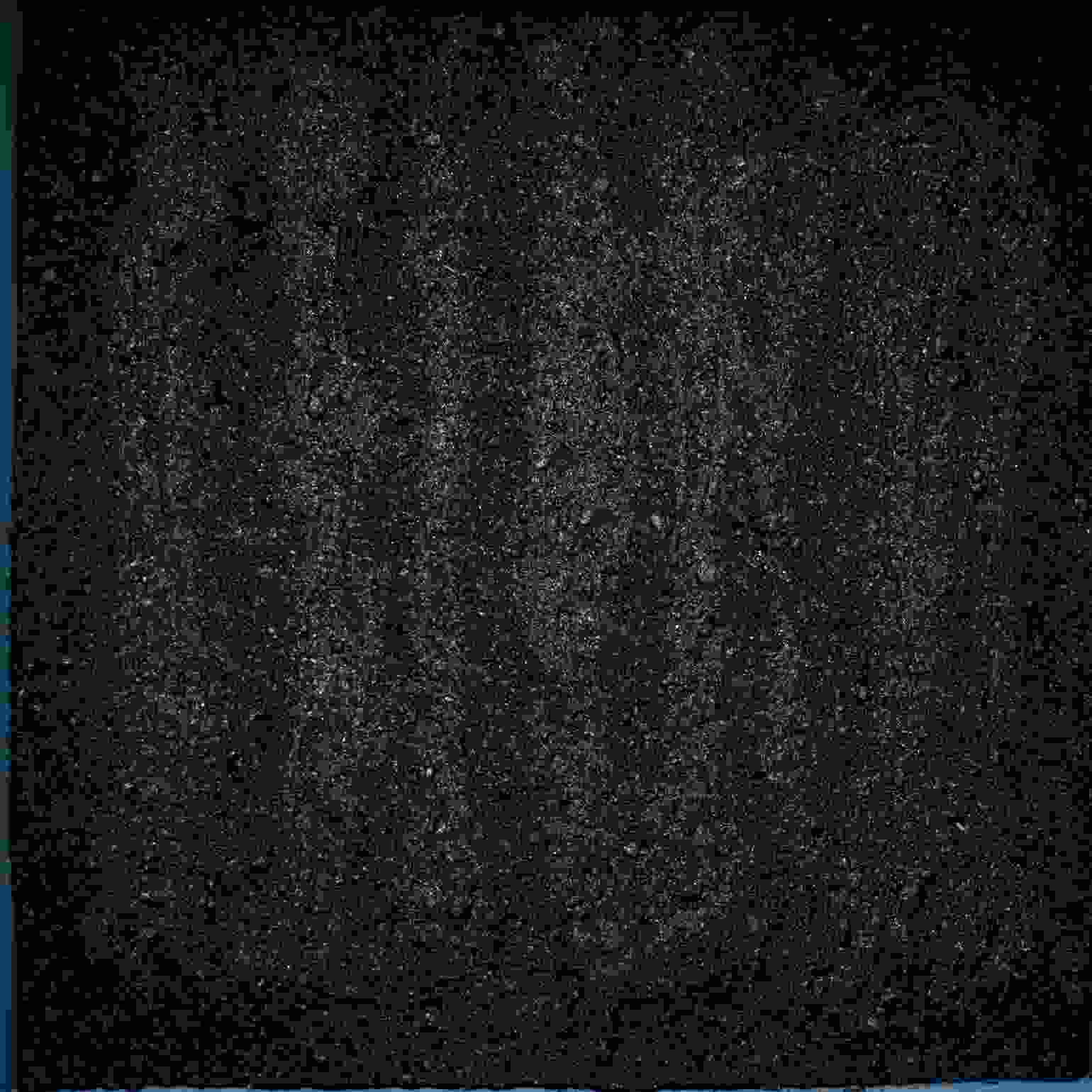

Supplement: Supplementary file 2 [file DataSheet2.zip › test/50150-2024-3-19-15-56-57.JPG]

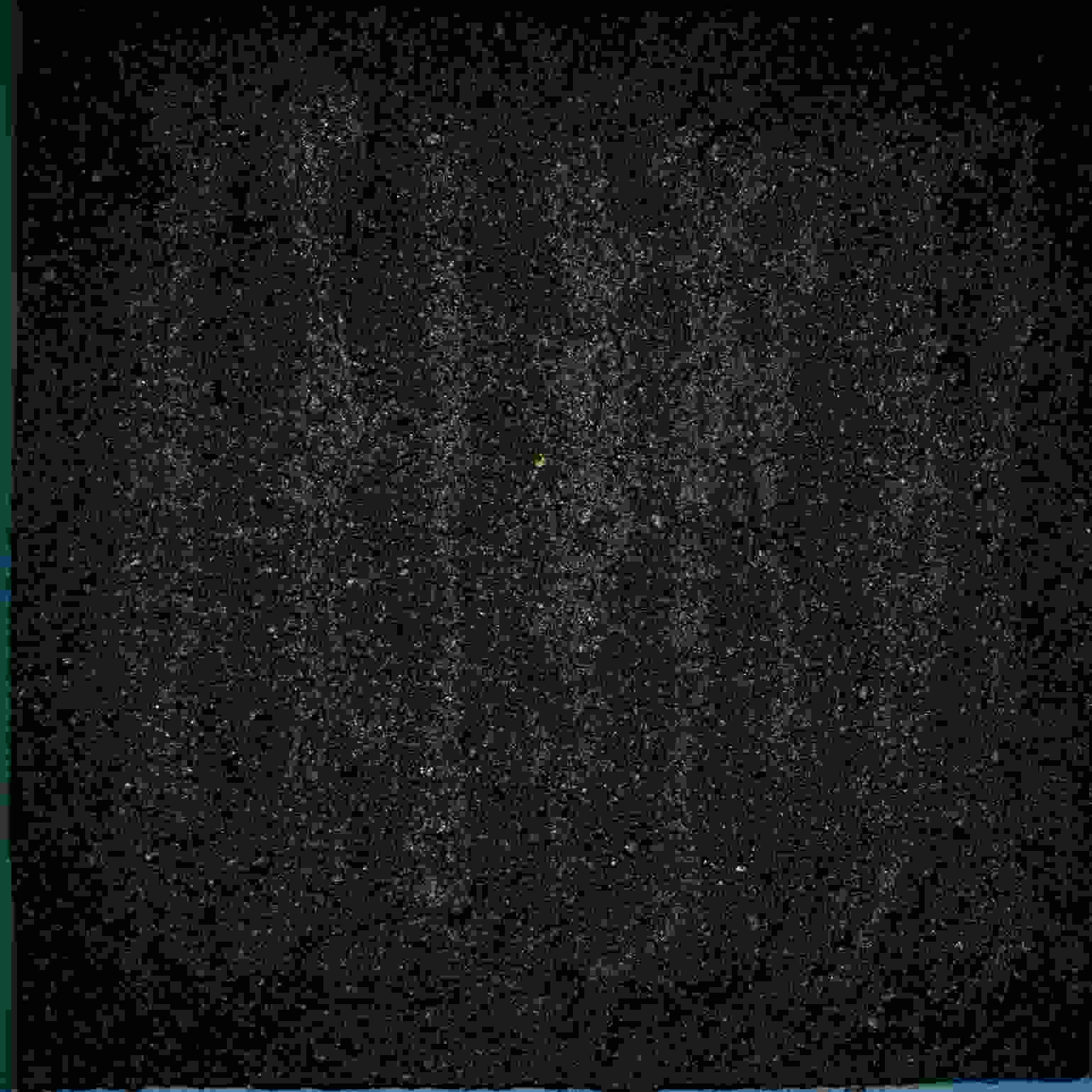

Supplement: Supplementary file 2 [file DataSheet2.zip › test/50150-2024-3-19-8-29-37.JPG]

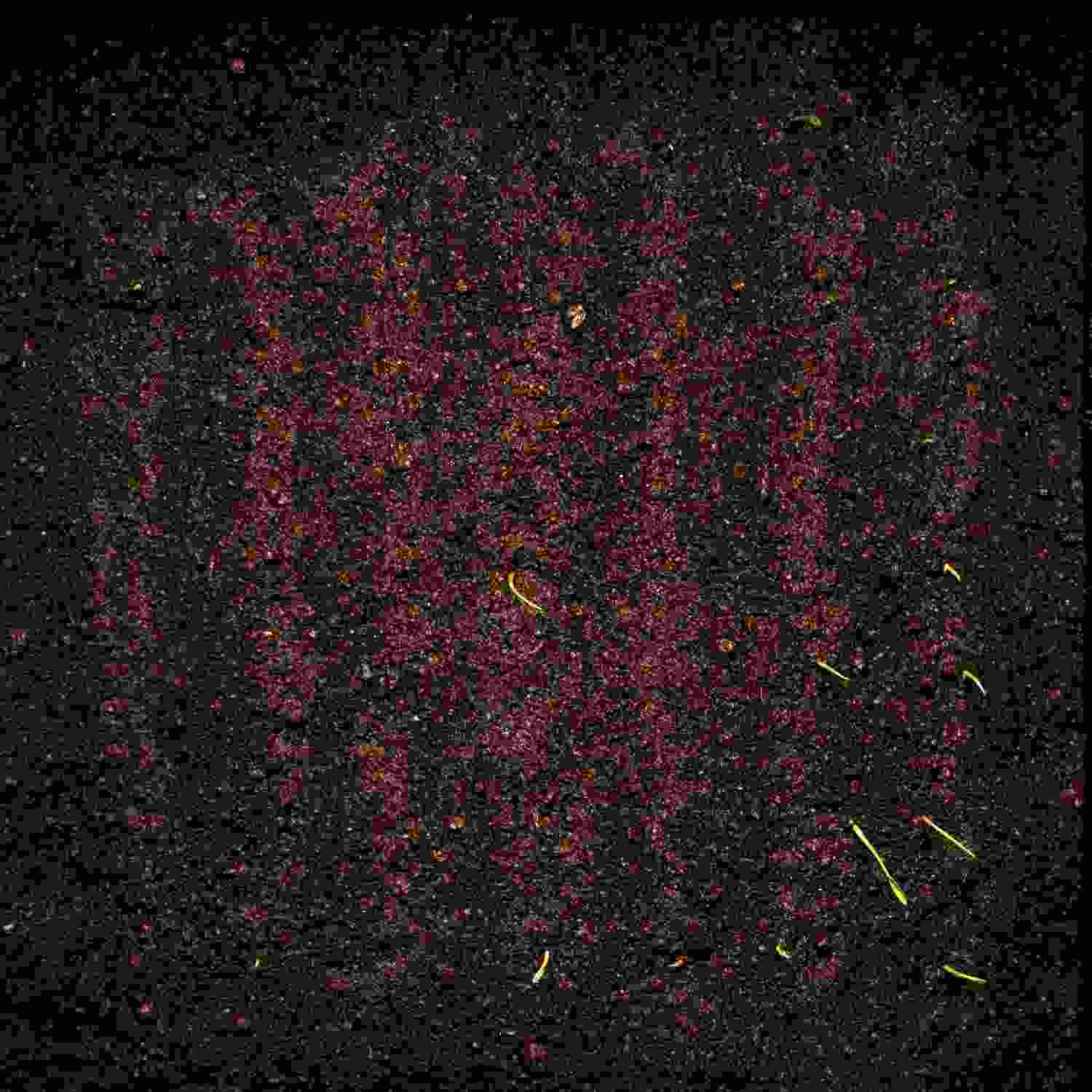

Supplement: Supplementary file 2 [file DataSheet2.zip › test/5090-2024-3-19-8-9-54.JPG]

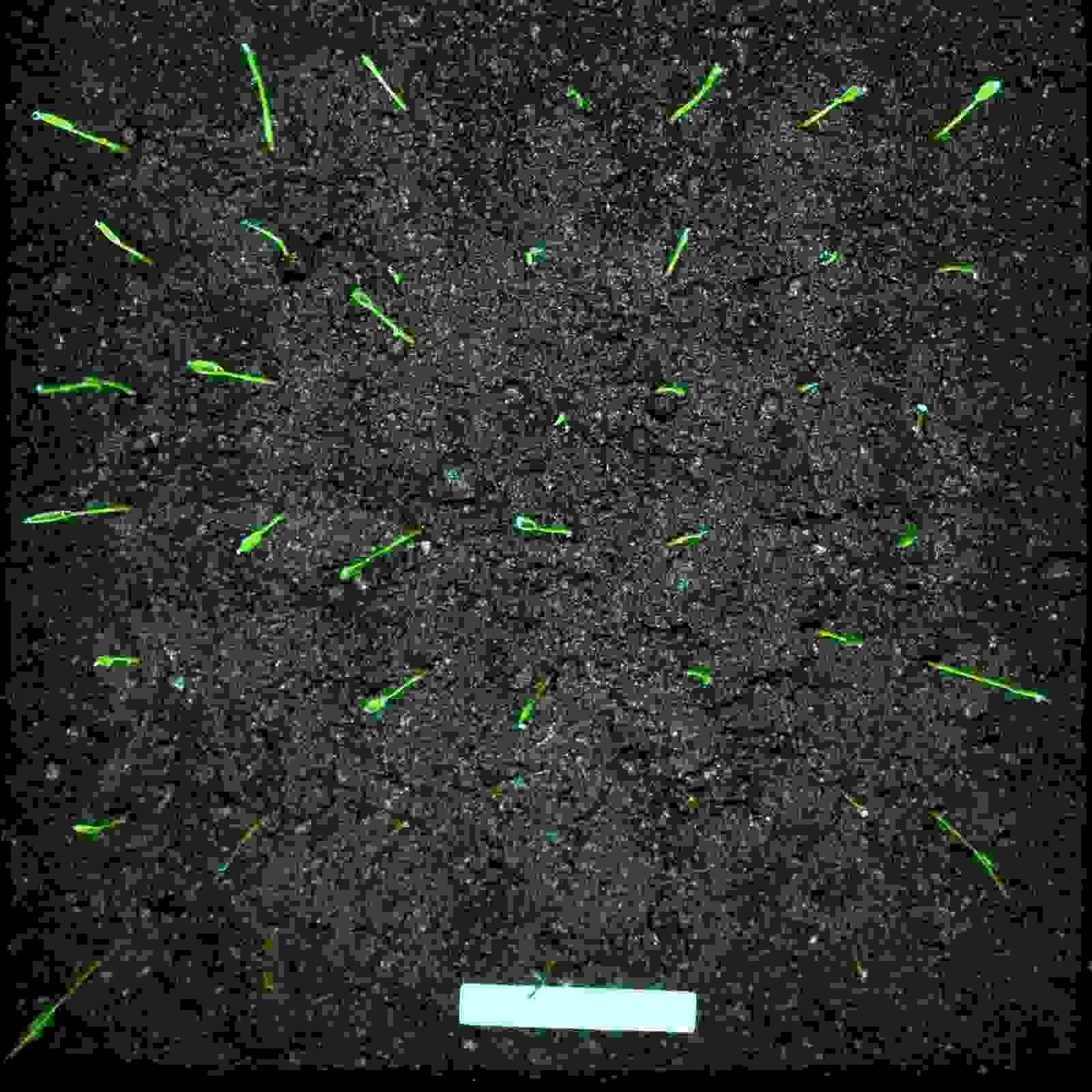

Supplement: Supplementary file 2 [file DataSheet2.zip › test/ck-2024-3-19-20-53-8.JPG]

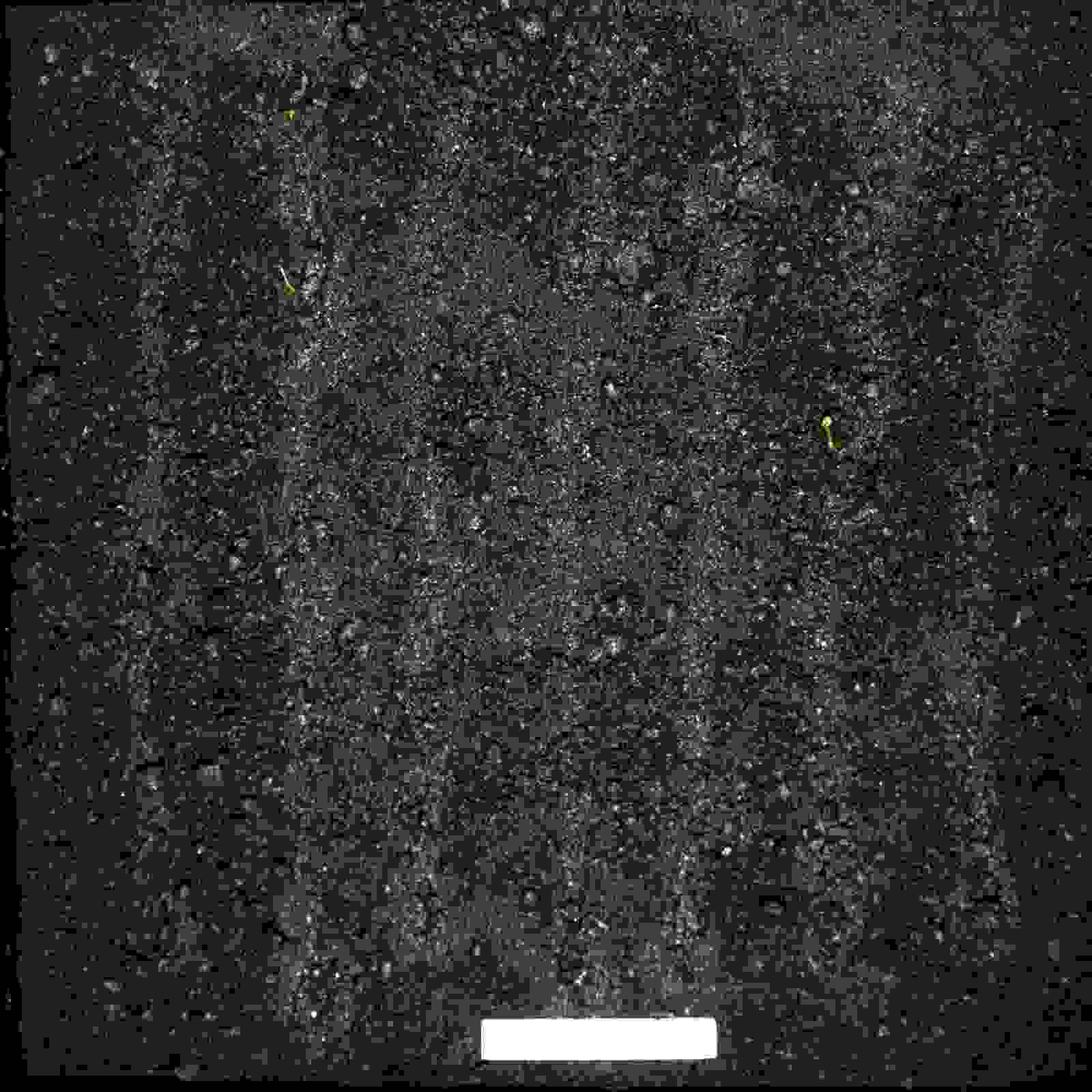

Supplement: Supplementary file 2 [file DataSheet2.zip › test/ck120-2024-3-19-1-41-16.JPG]

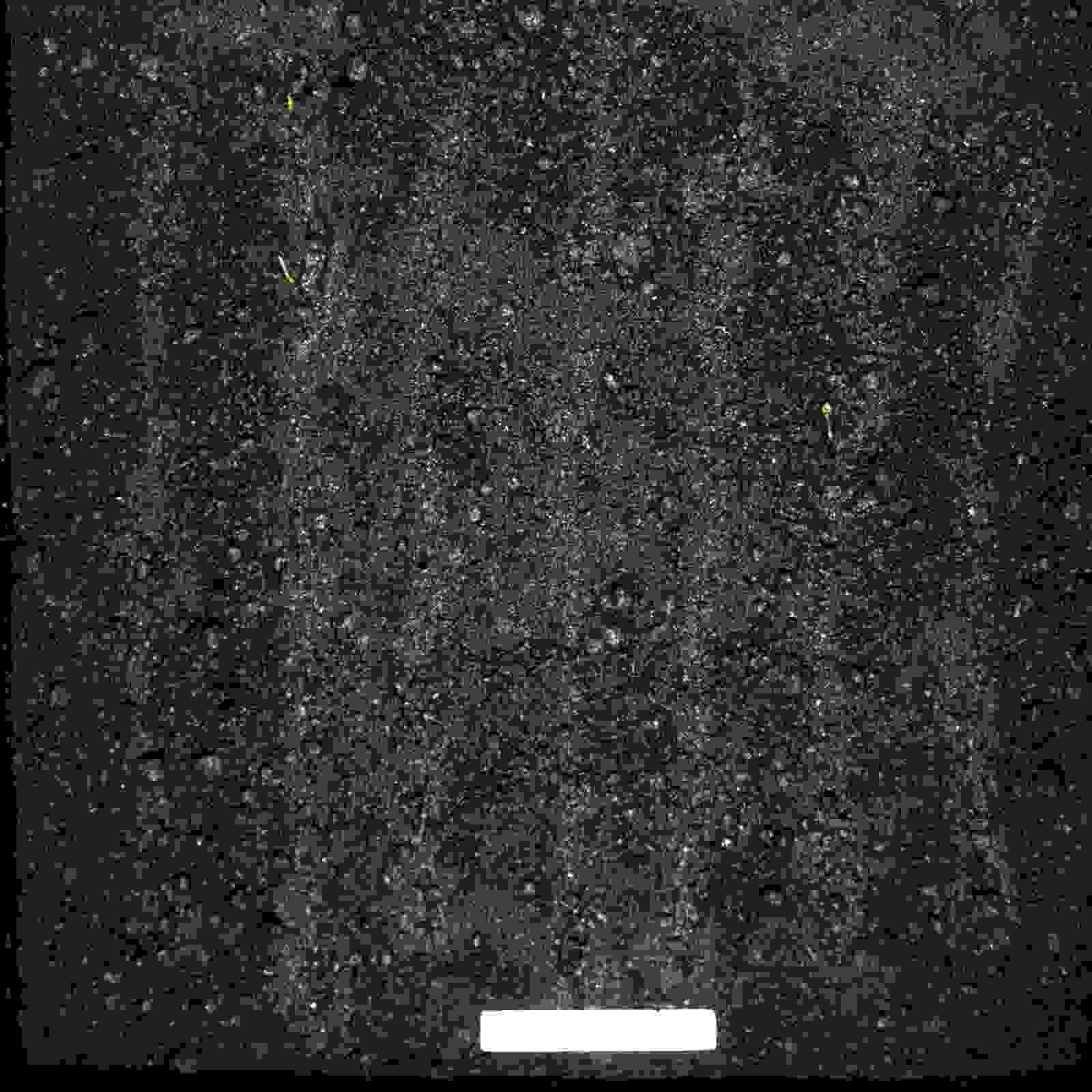

Supplement: Supplementary file 2 [file DataSheet2.zip › test/ck120-2024-3-19-7-41-54.JPG]

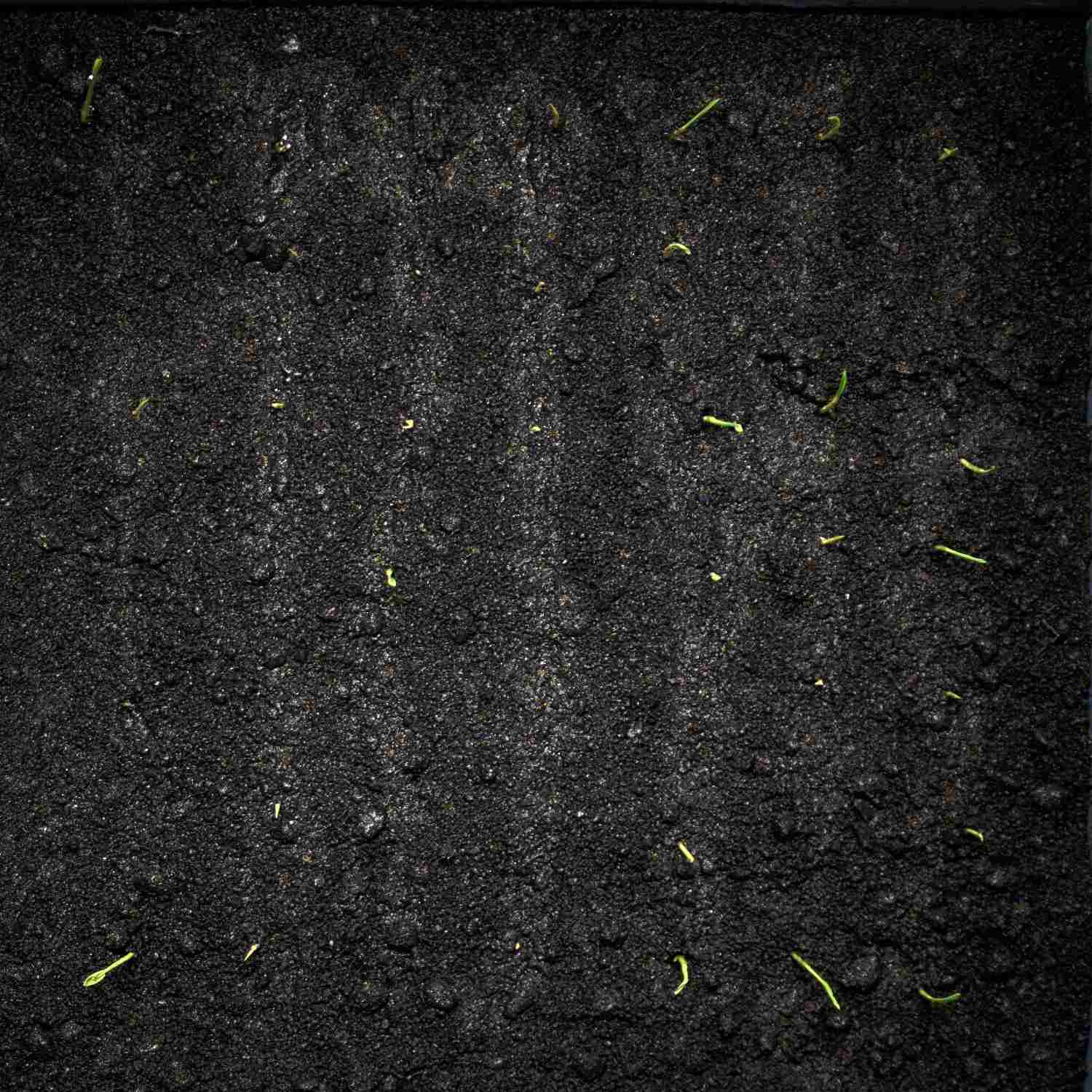

Supplement: Supplementary file 2 [file DataSheet2.zip › test/ck30-2024-3-19-0-10-19.JPG]

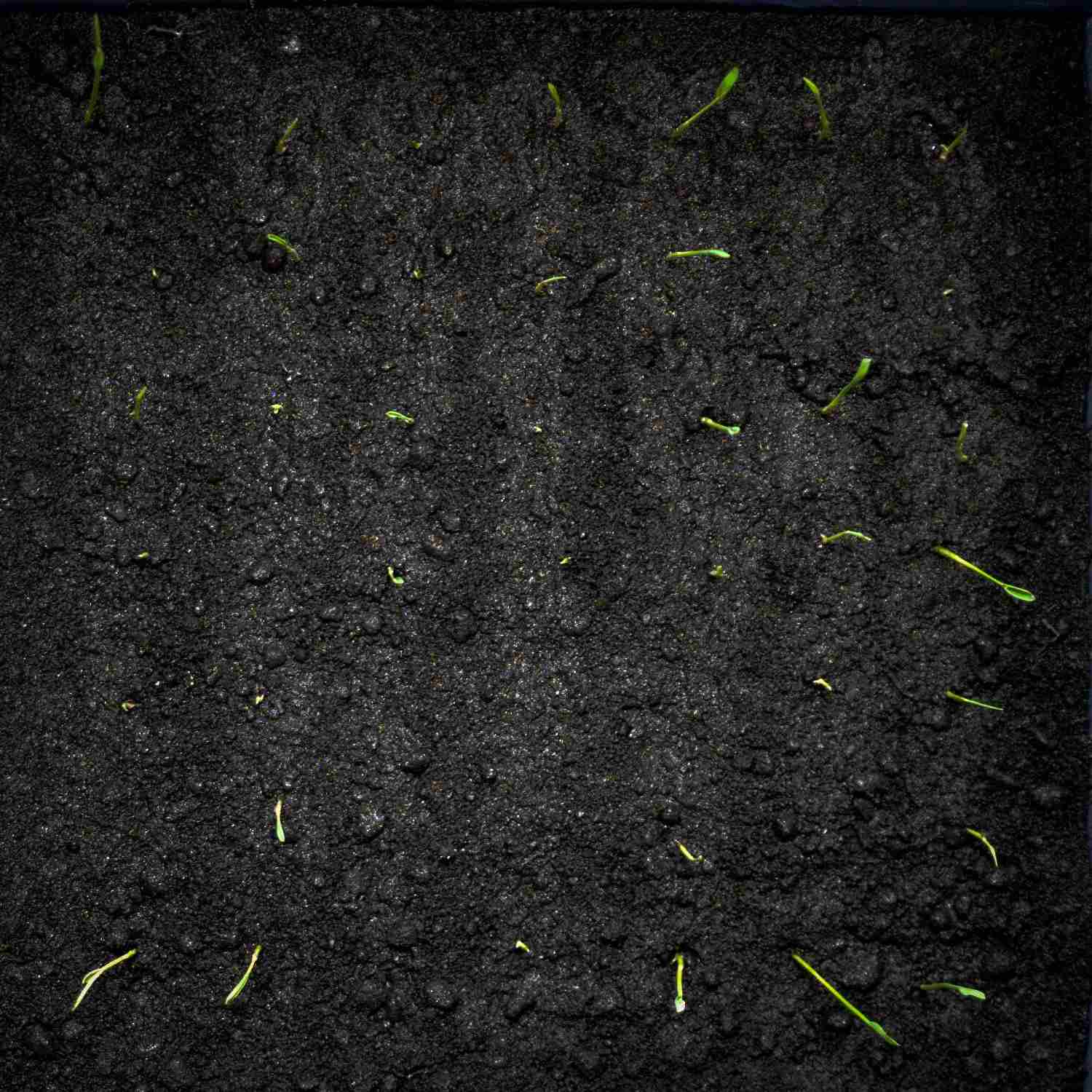

Supplement: Supplementary file 2 [file DataSheet2.zip › test/ck30-2024-3-20-10-26-41.JPG]

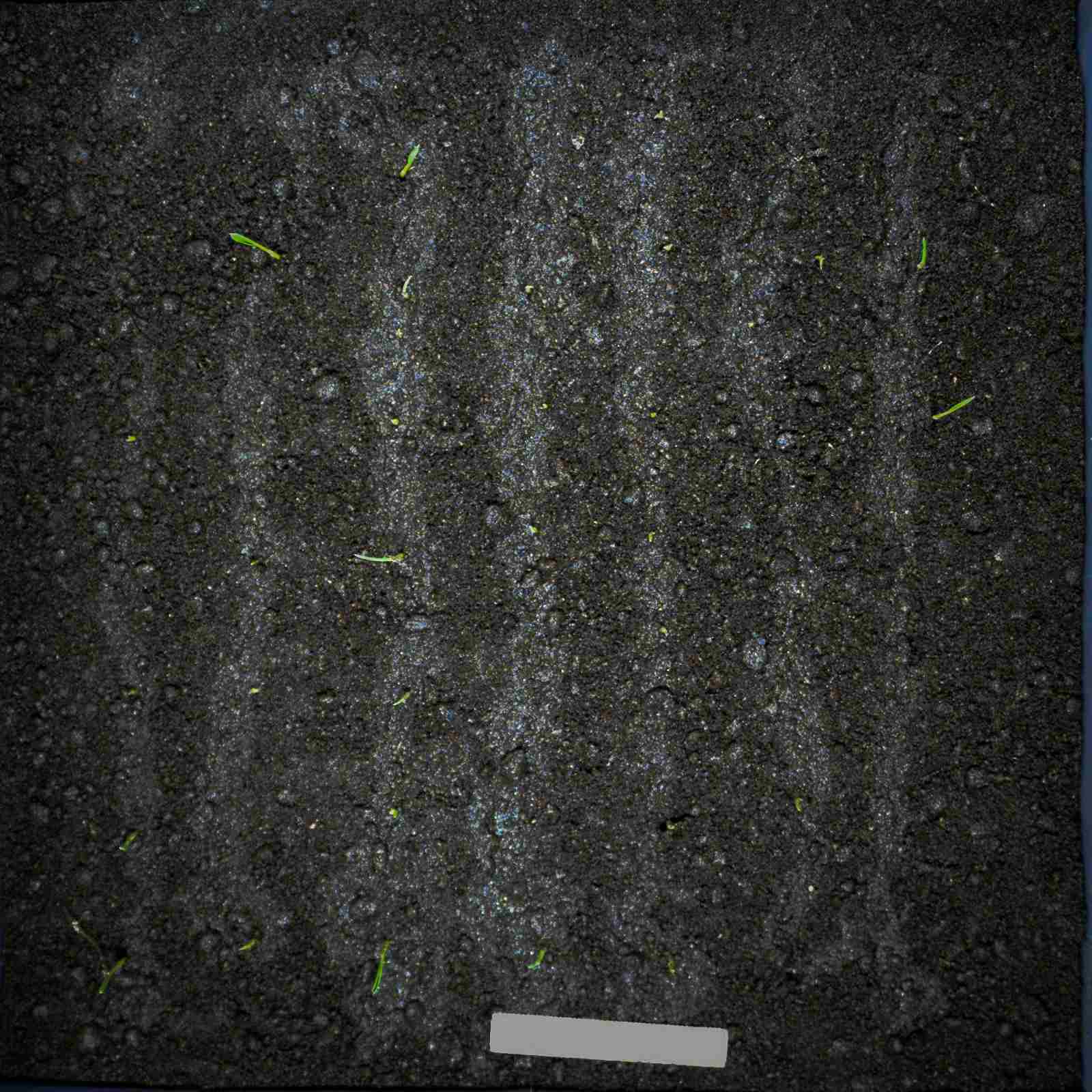

Supplement: Supplementary file 2 [file DataSheet2.zip › test/ck60-2024-3-19-1-40-21.JPG]

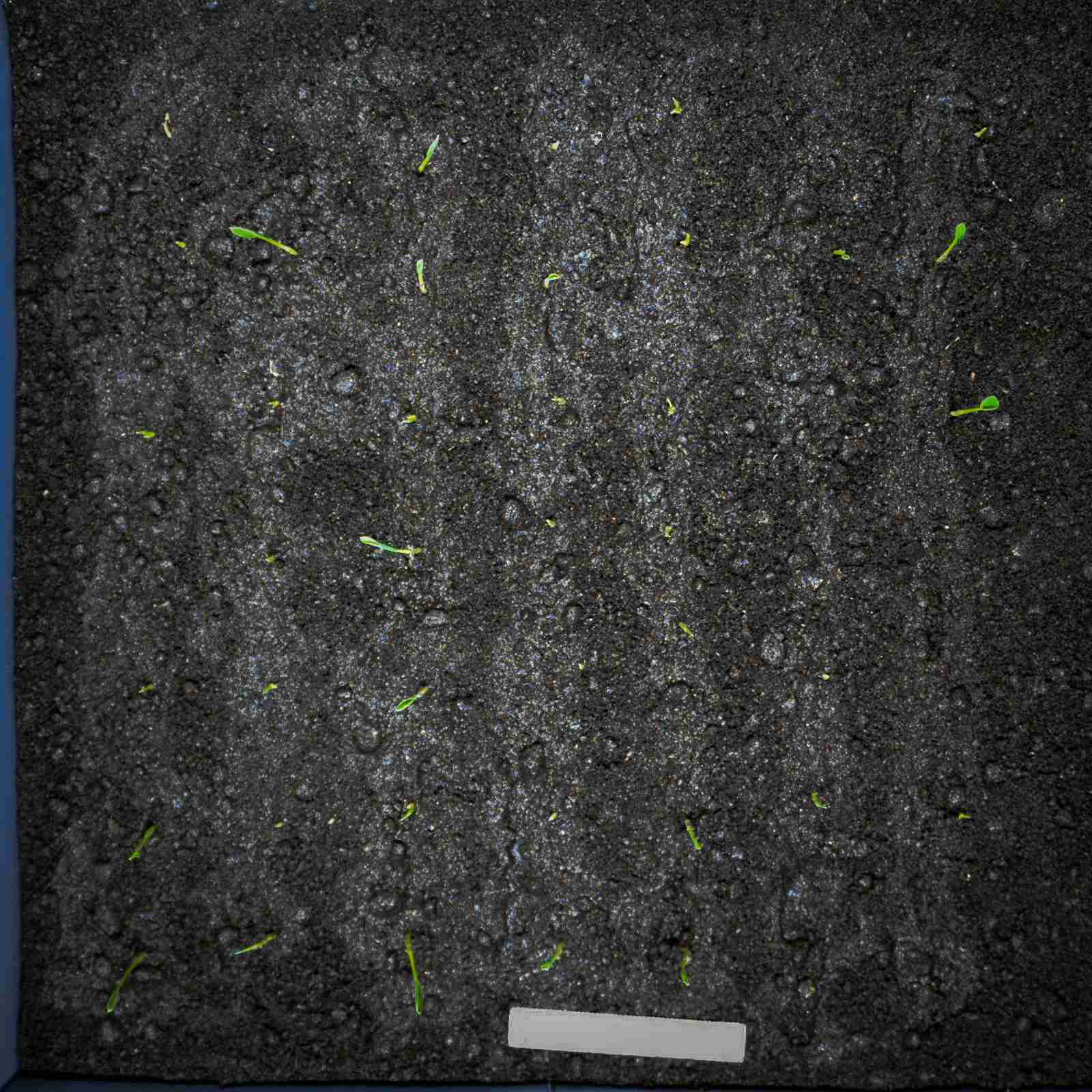

Supplement: Supplementary file 2 [file DataSheet2.zip › test/ck60-2024-3-19-22-42-34.JPG]

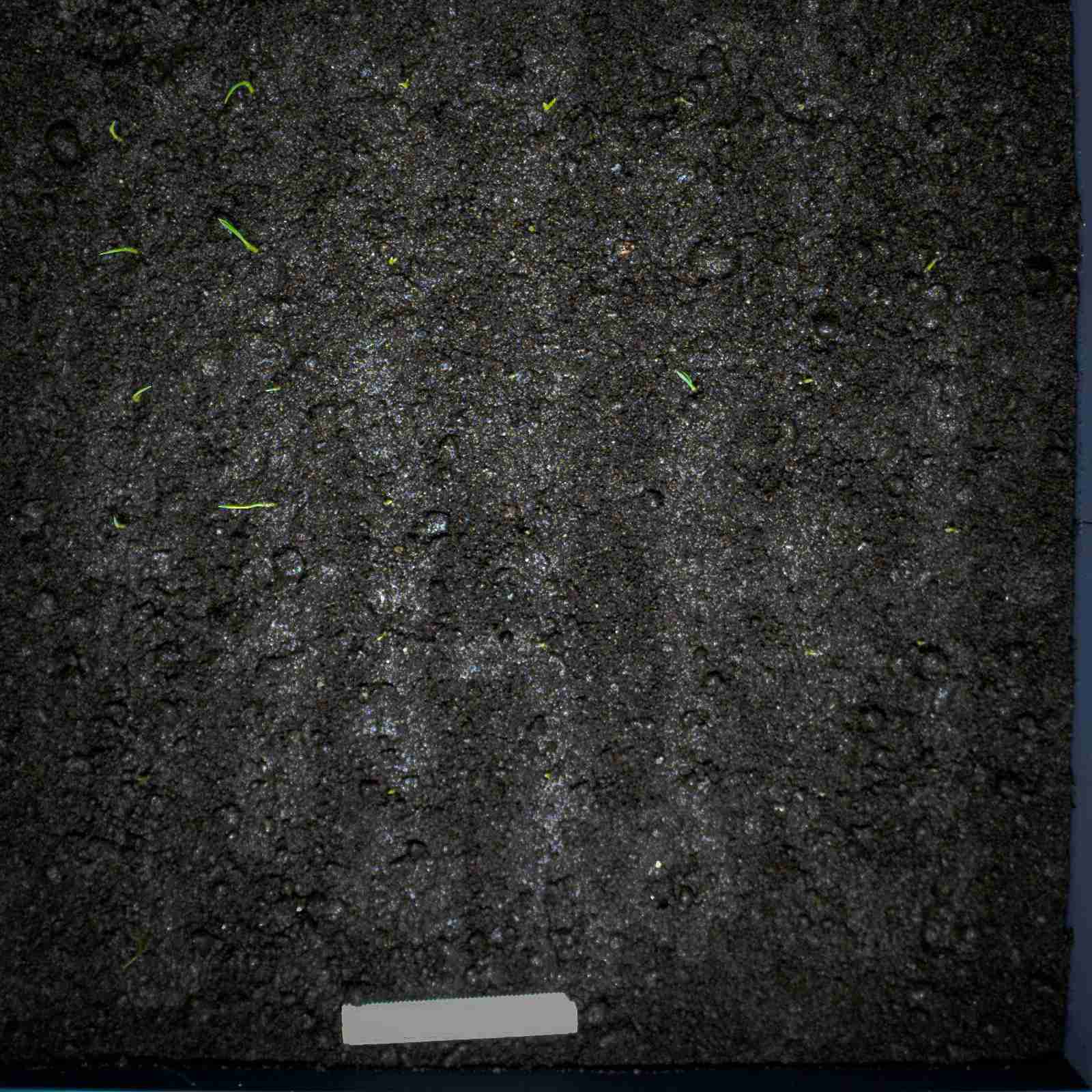

Supplement: Supplementary file 2 [file DataSheet2.zip › test/ck90-024-3-19-10-41-32.JPG]

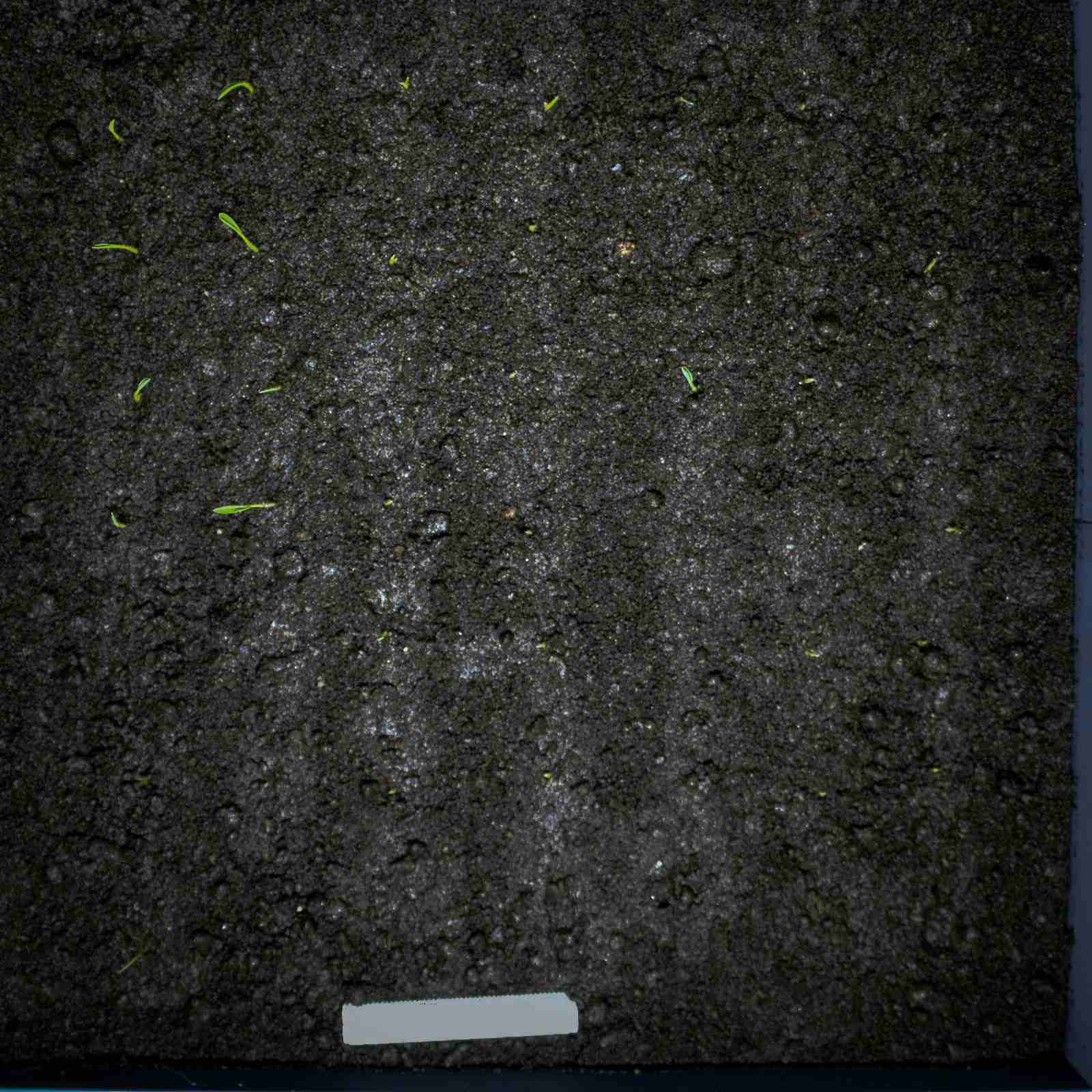

Supplement: Supplementary file 2 [file DataSheet2.zip › test/ck90-2024-3-19-16-42-17.JPG]

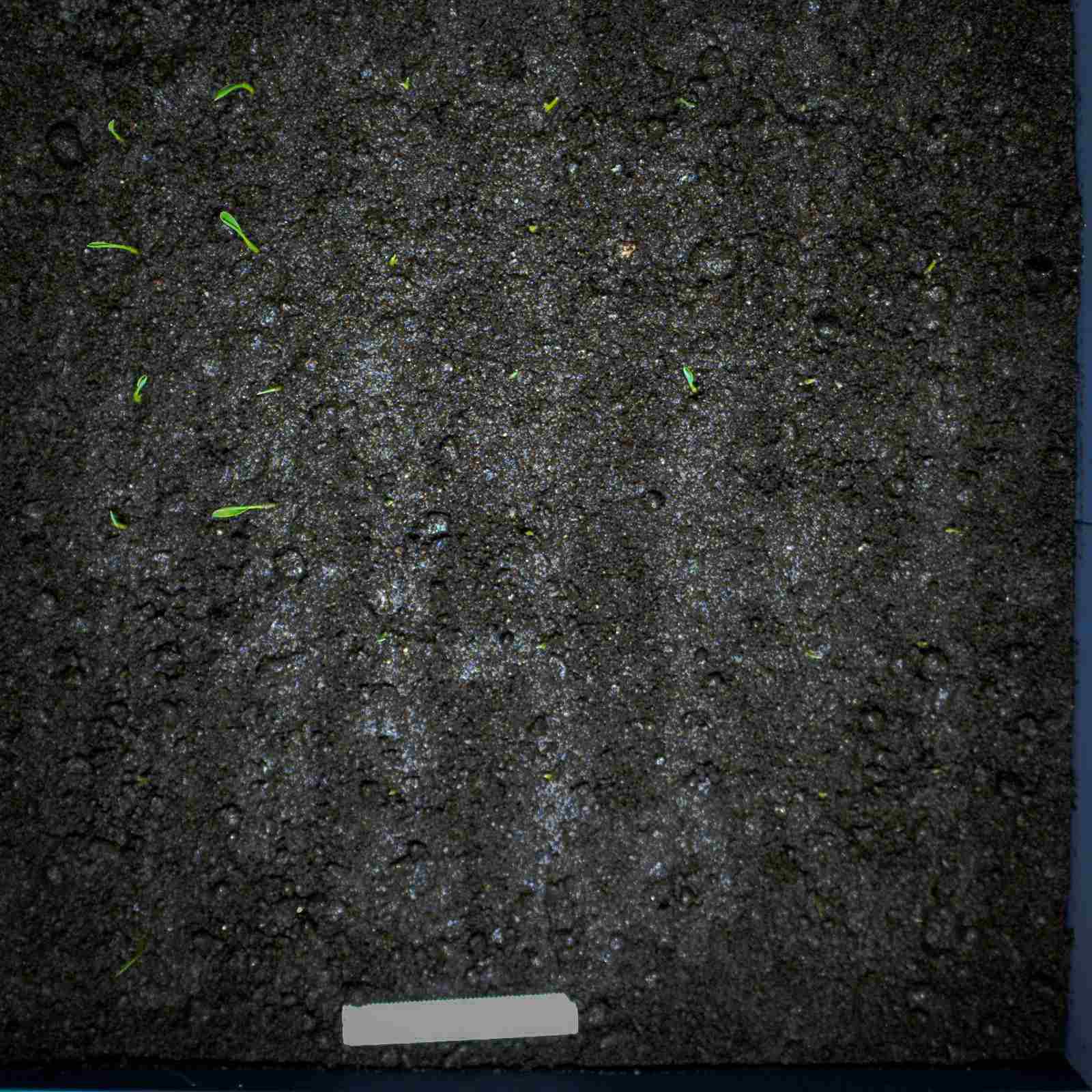

Supplement: Supplementary file 2 [file DataSheet2.zip › test/ck90-2024-3-19-19-42-15.JPG]

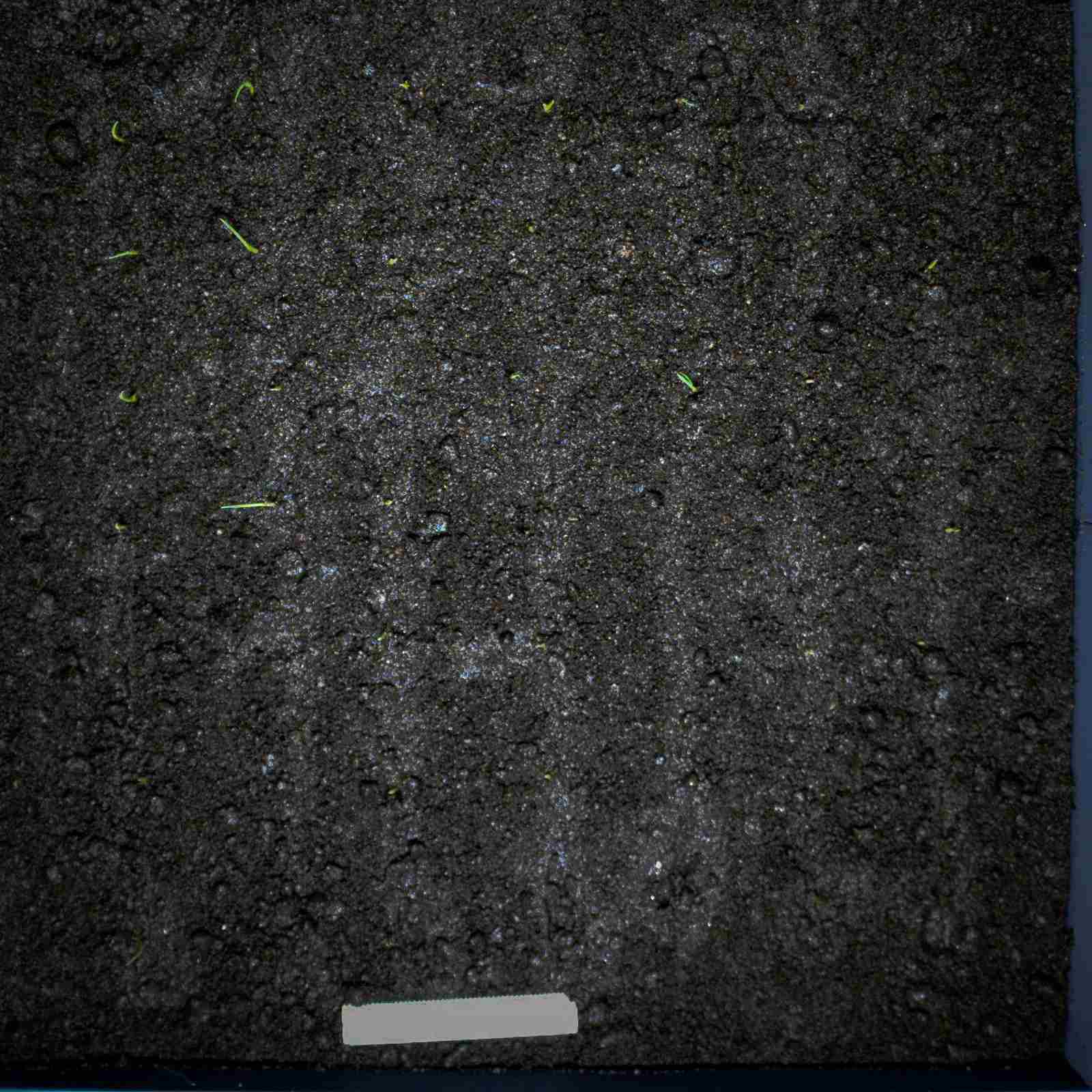

Supplement: Supplementary file 2 [file DataSheet2.zip › test/ck90-2024-3-19-4-41-7.JPG]

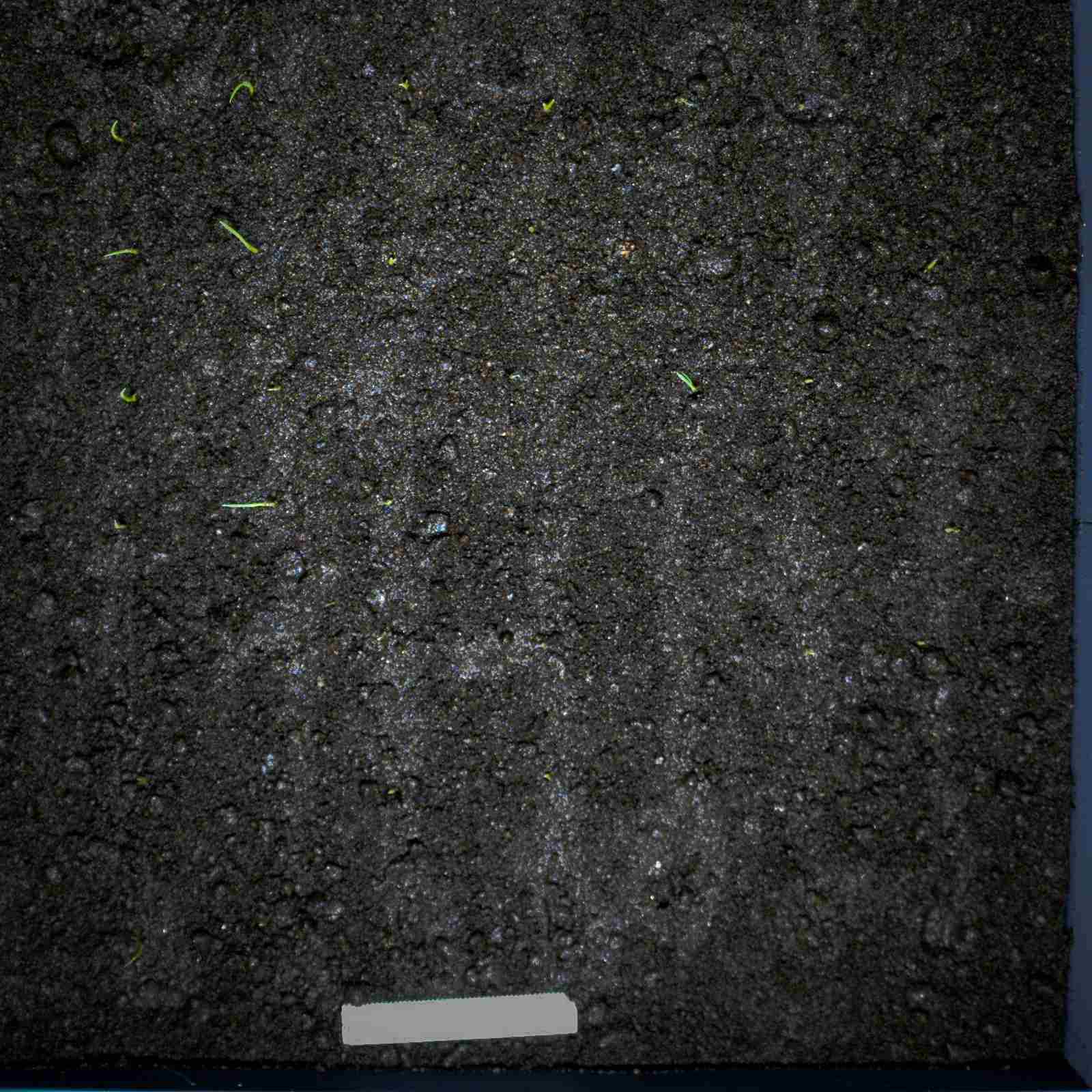

Supplement: Supplementary file 2 [file DataSheet2.zip › test/ck90-2024-3-19-7-41-34.JPG]

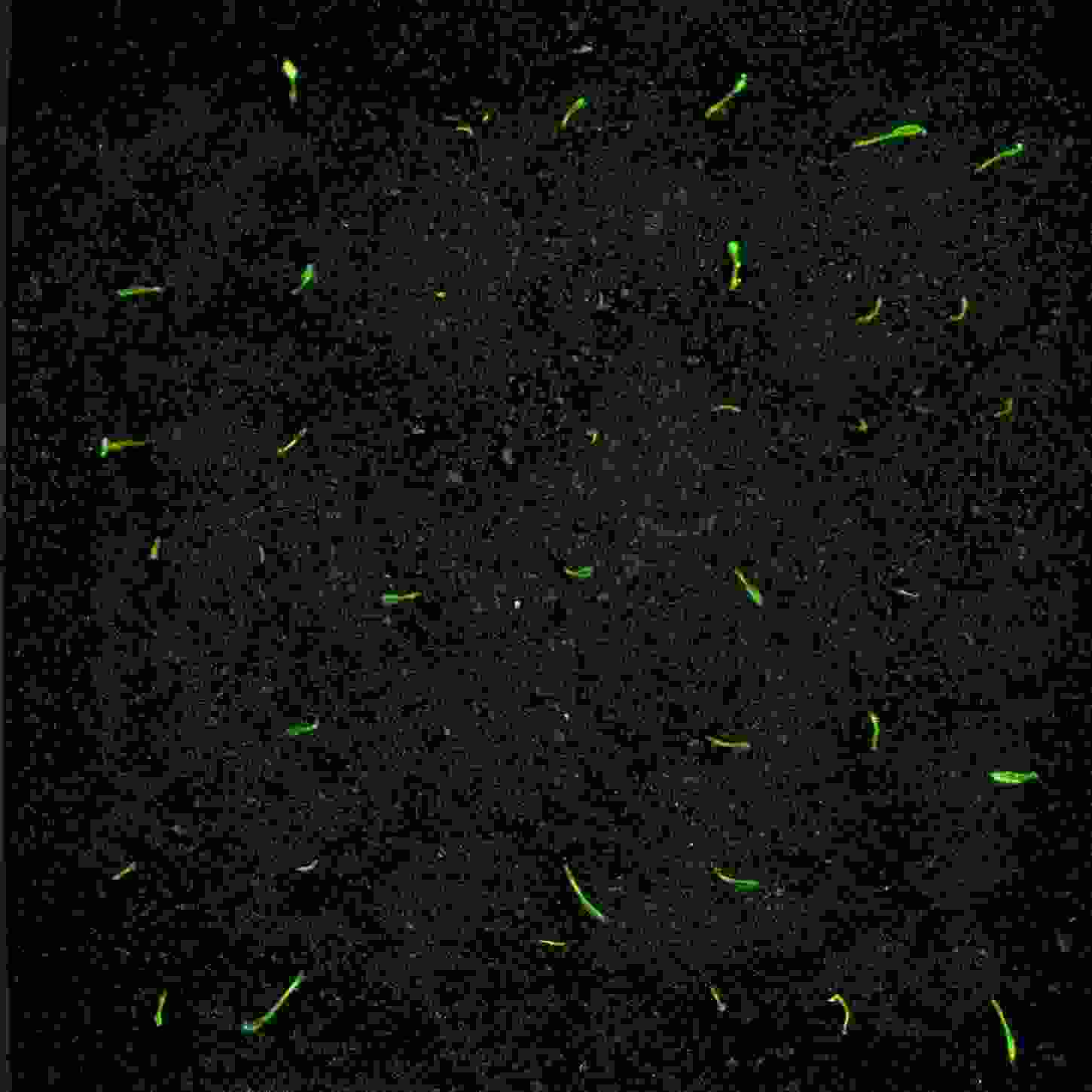

Supplement: Supplementary file 3 [file DataSheet3.zip › train1/1000-2024-3-18-15-48-40.JPG]

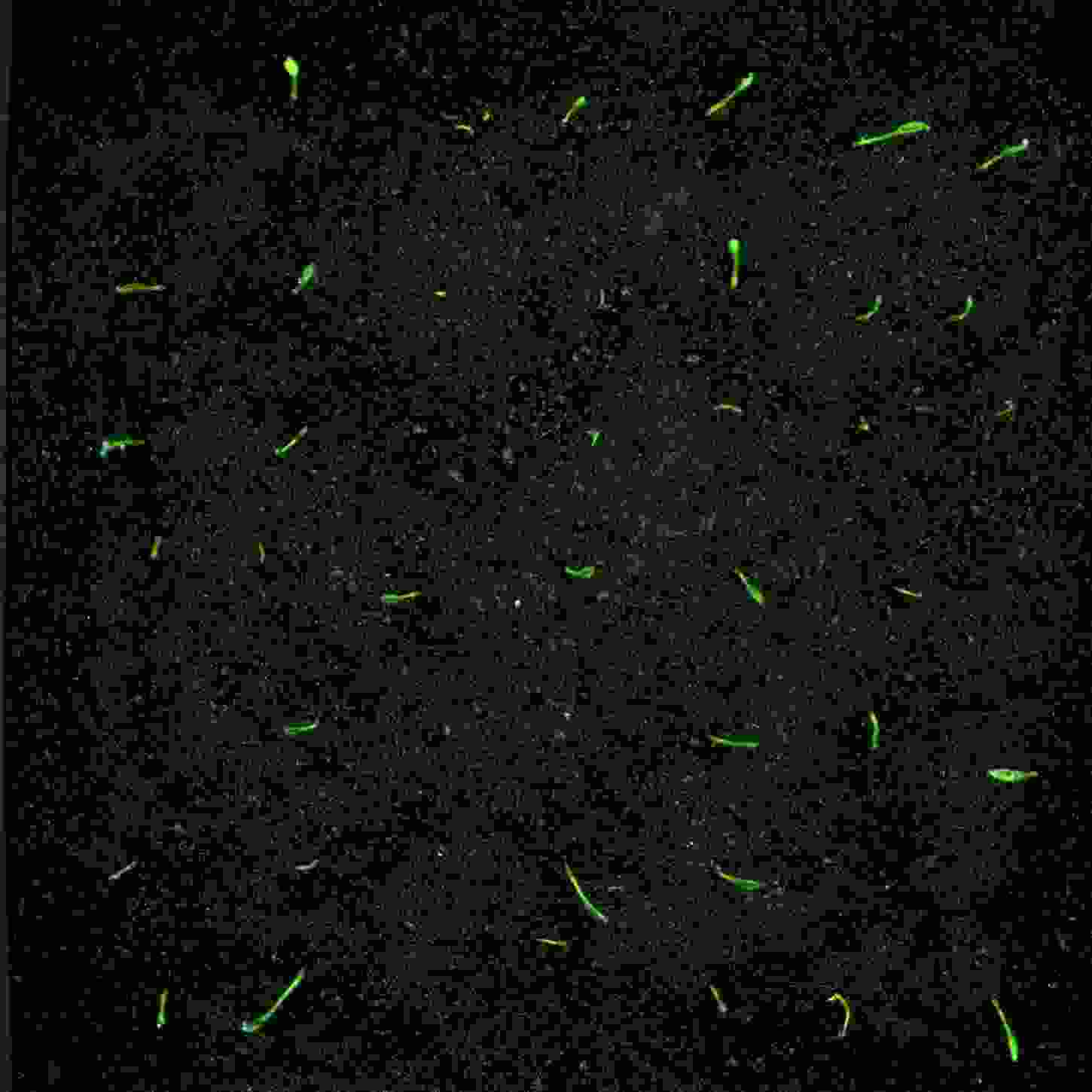

Supplement: Supplementary file 3 [file DataSheet3.zip › train1/1000-2024-3-18-18-22-50.JPG]

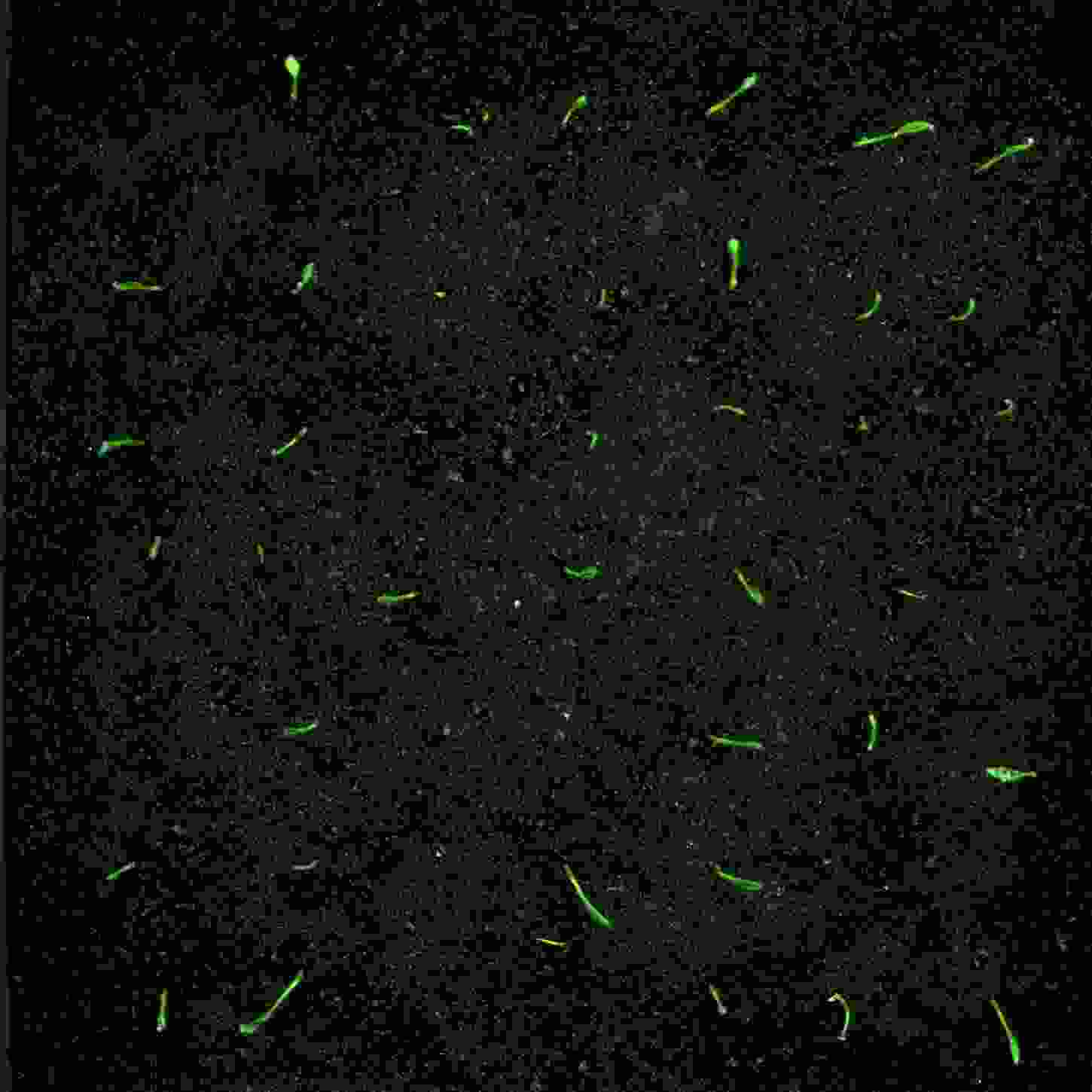

Supplement: Supplementary file 3 [file DataSheet3.zip › train1/1000-2024-3-18-20-57-48.JPG]

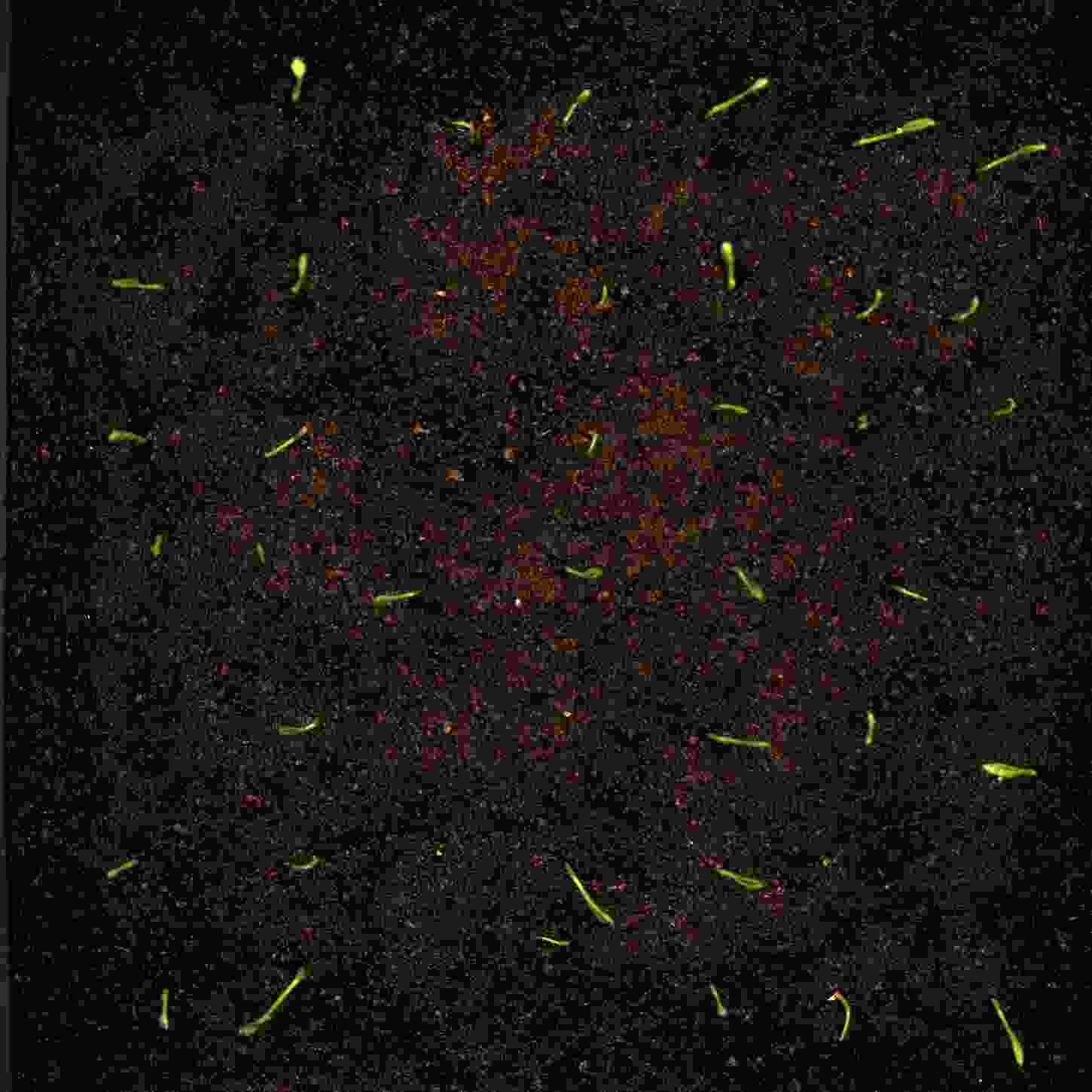

Supplement: Supplementary file 3 [file DataSheet3.zip › train1/1000-2024-3-18-23-32-7.JPG]

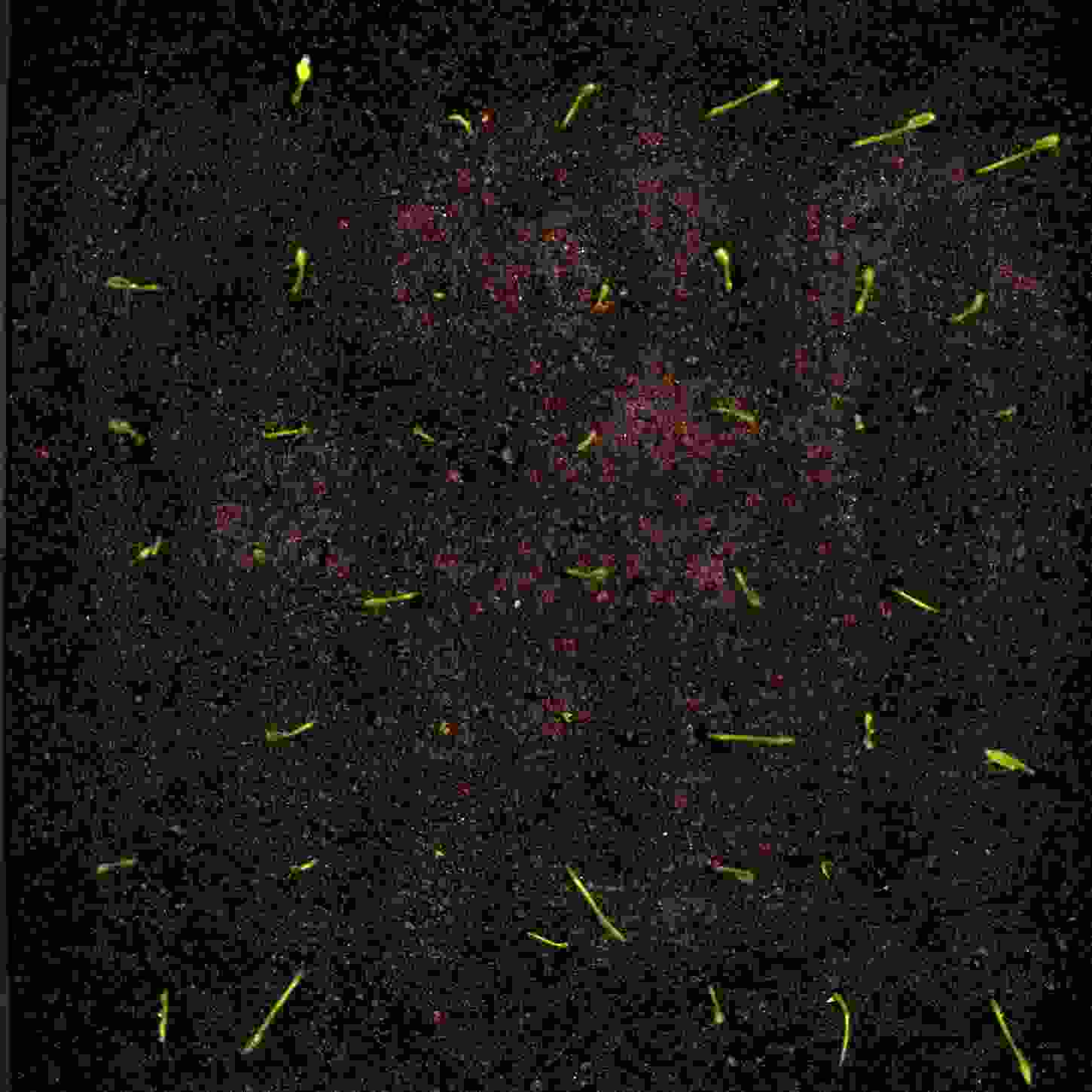

Supplement: Supplementary file 3 [file DataSheet3.zip › train1/1000-2024-3-19-12-18-14.JPG]

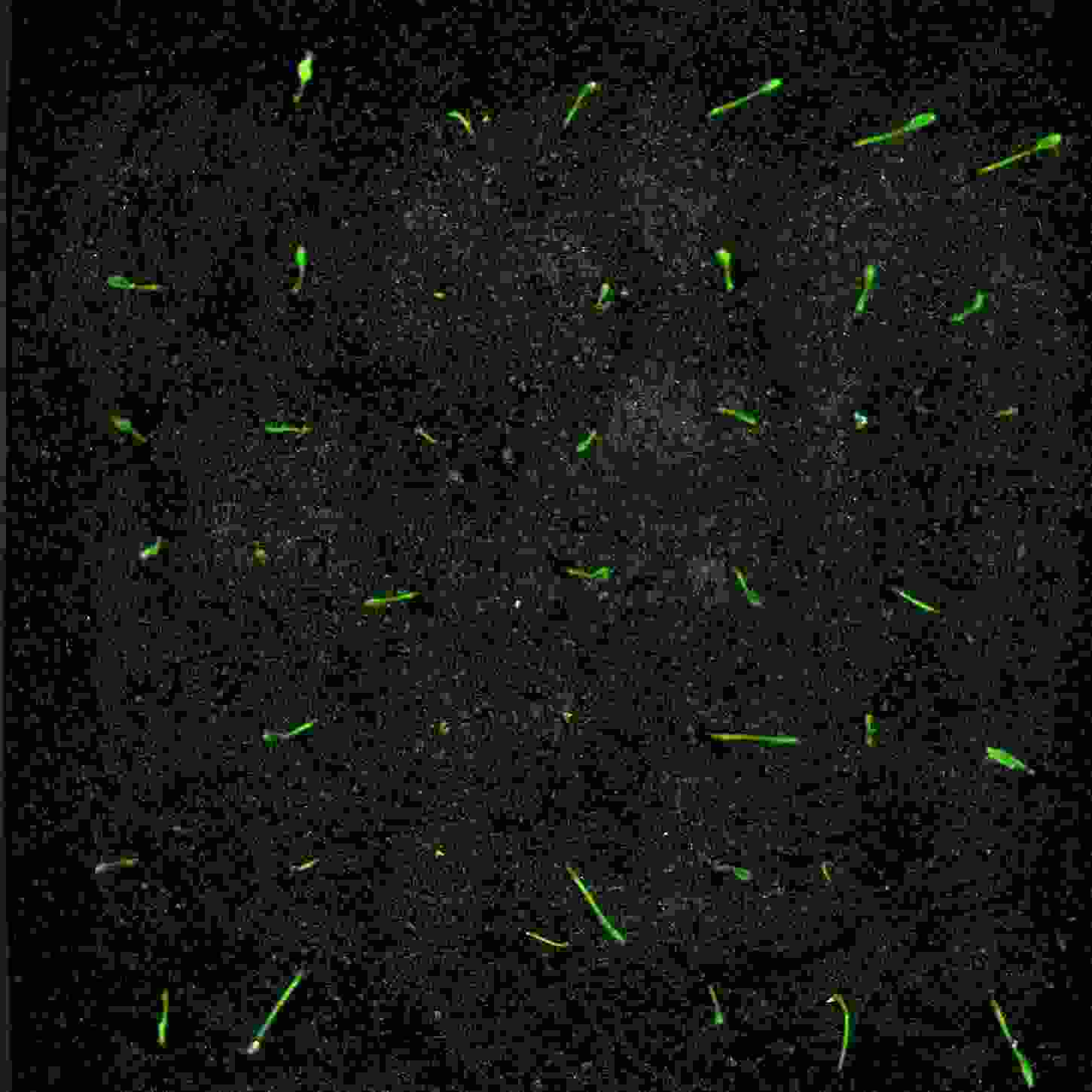

Supplement: Supplementary file 3 [file DataSheet3.zip › train1/1000-2024-3-19-14-50-33.JPG]

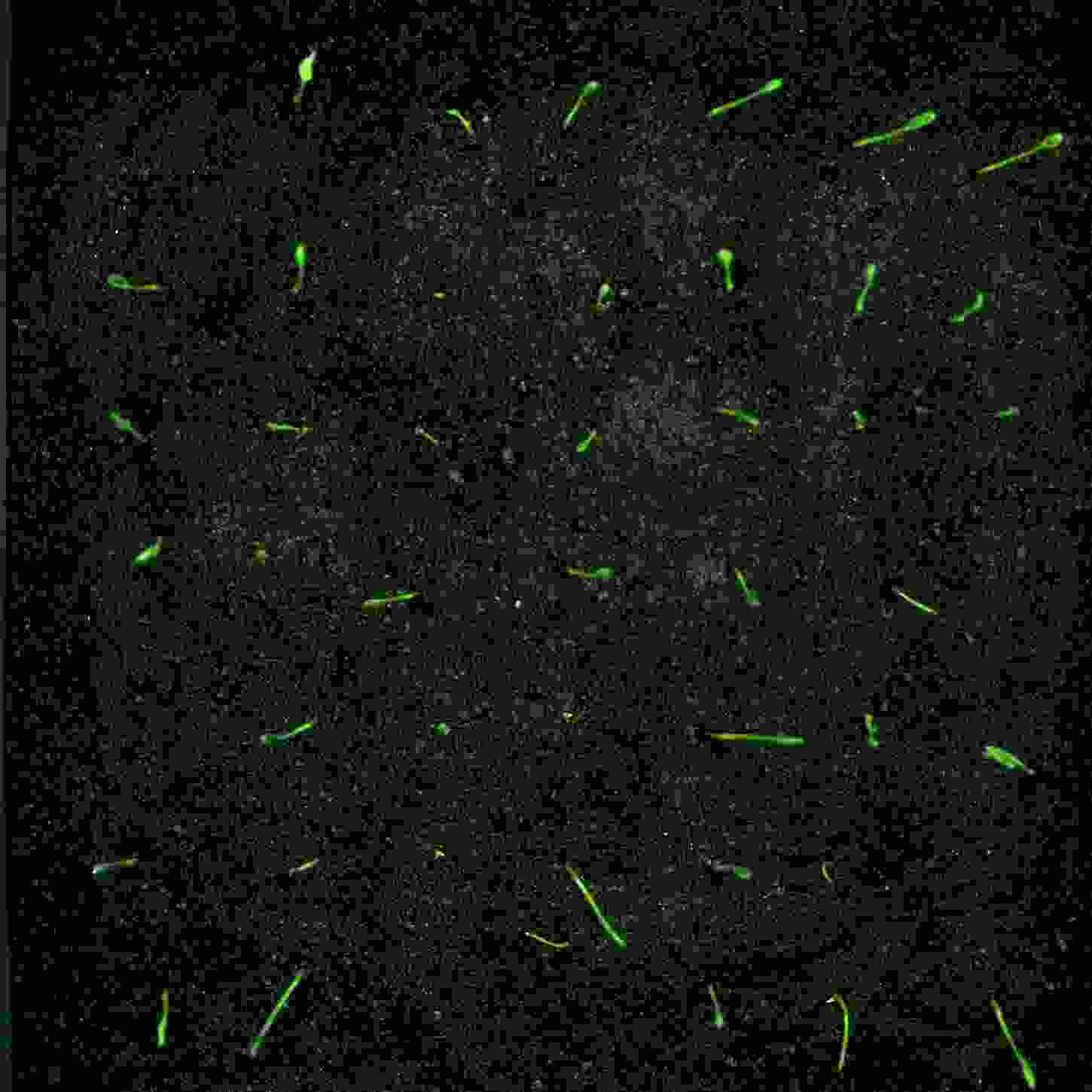

Supplement: Supplementary file 3 [file DataSheet3.zip › train1/1000-2024-3-19-17-23-15.JPG]

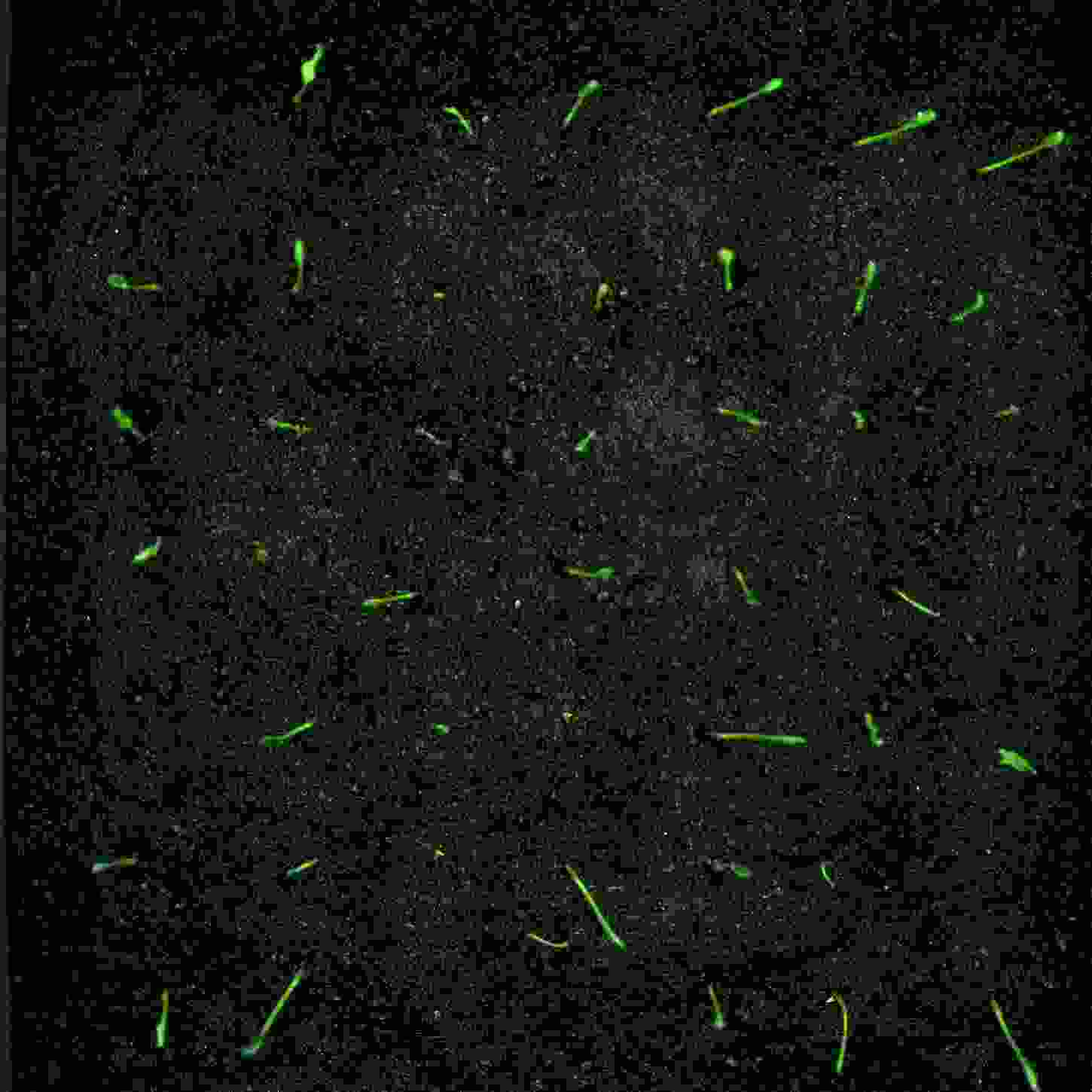

Supplement: Supplementary file 3 [file DataSheet3.zip › train1/1000-2024-3-19-19-56-29.JPG]

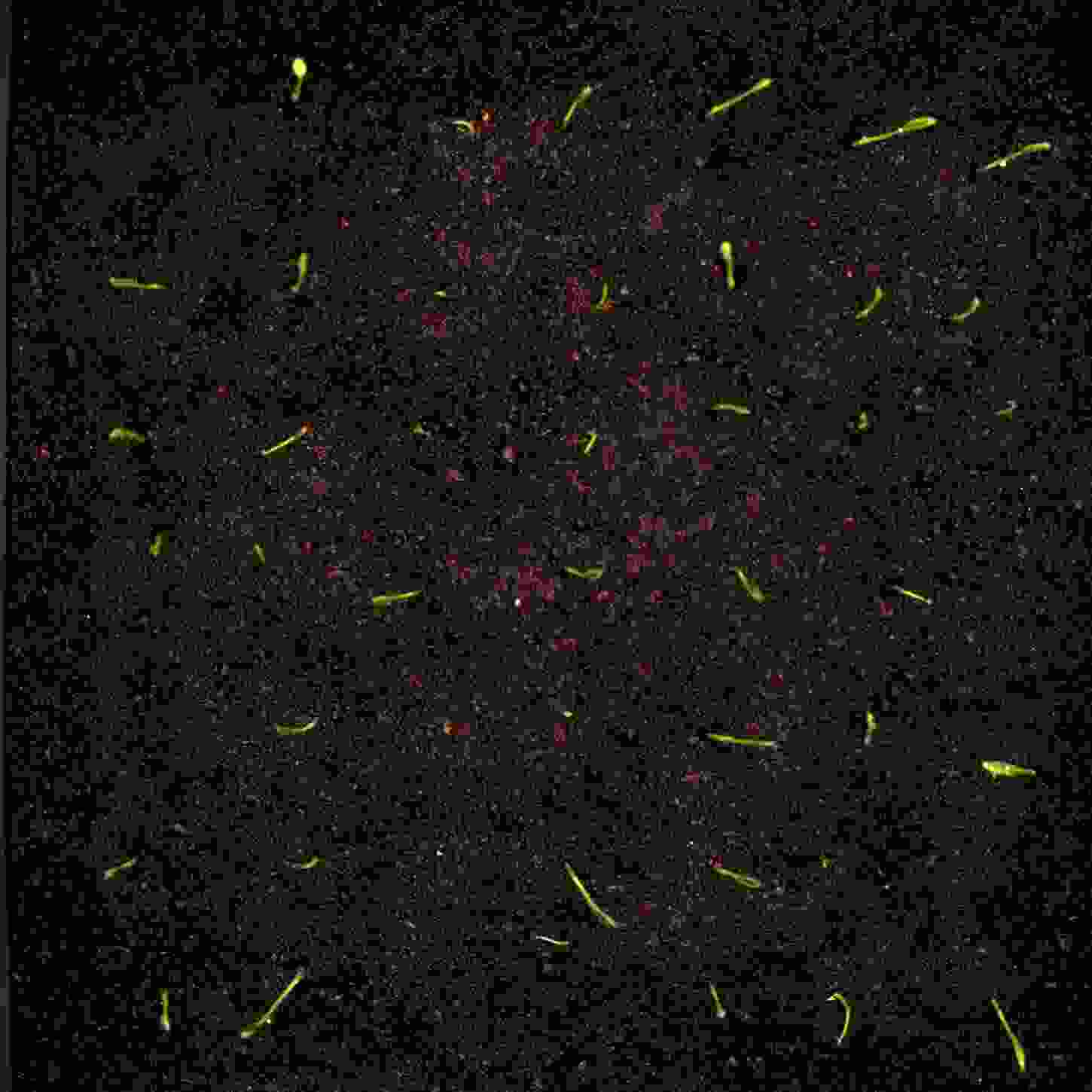

Supplement: Supplementary file 3 [file DataSheet3.zip › train1/1000-2024-3-19-2-5-43.JPG]

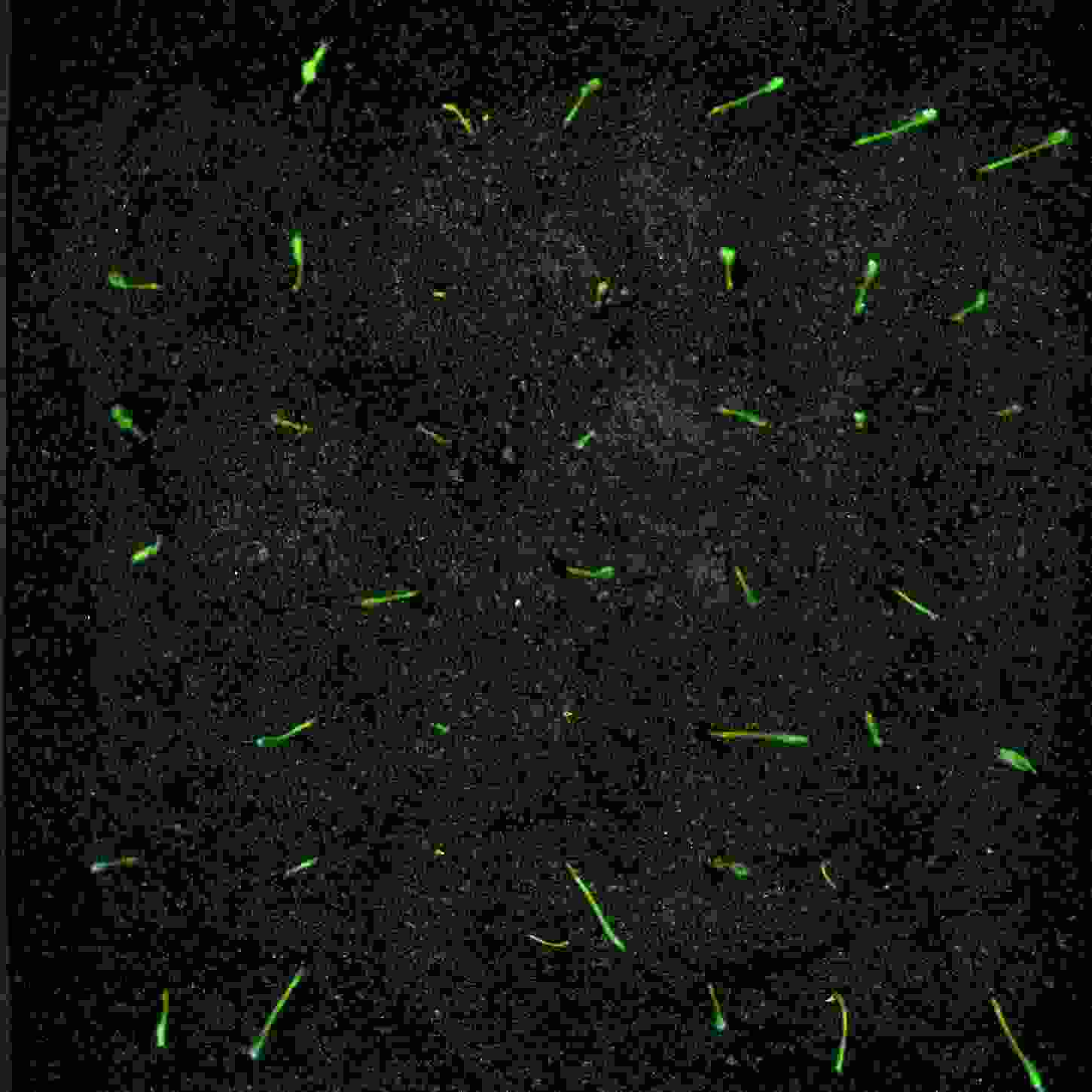

Supplement: Supplementary file 3 [file DataSheet3.zip › train1/1000-2024-3-19-22-29-34.JPG]

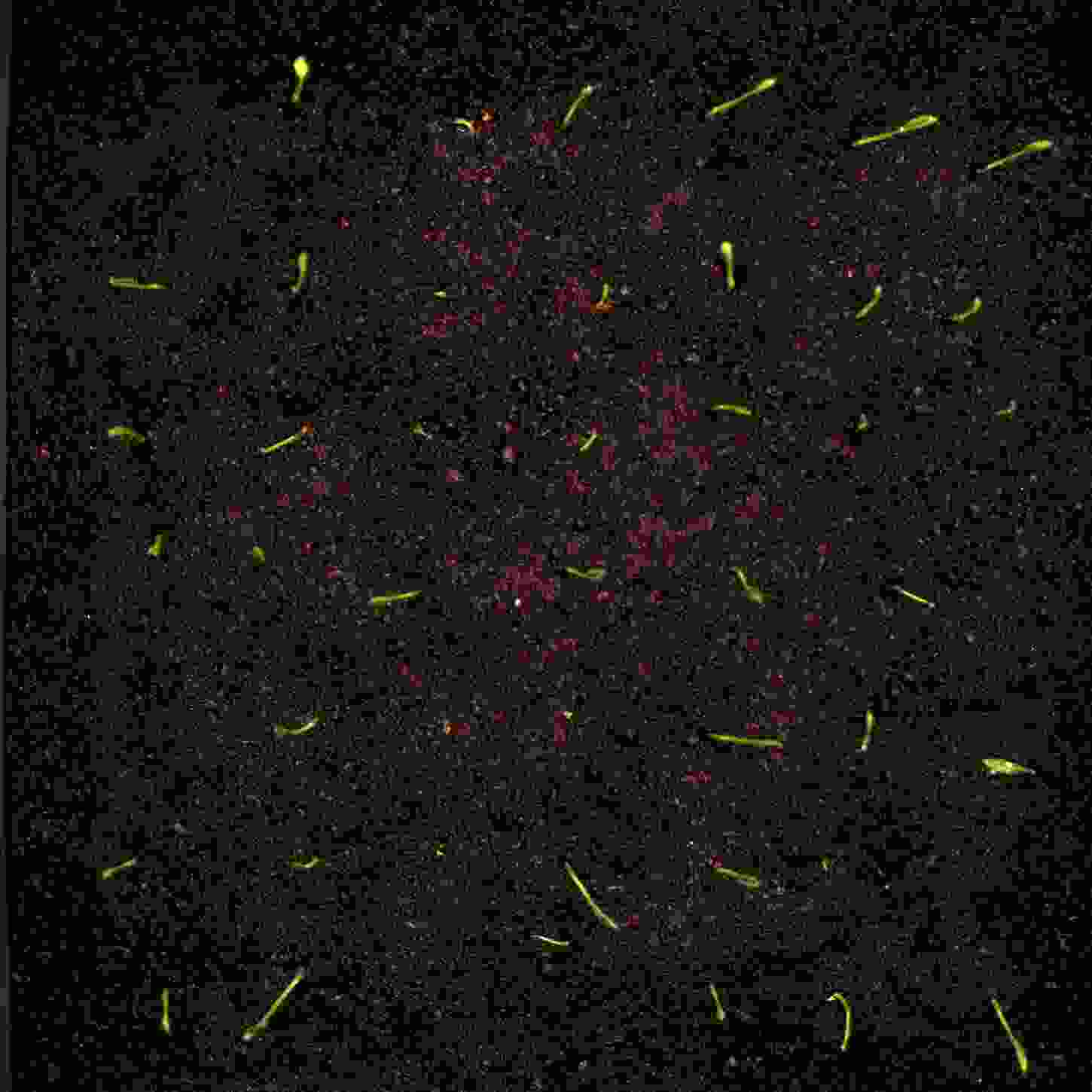

Supplement: Supplementary file 3 [file DataSheet3.zip › train1/1000-2024-3-19-4-38-55.JPG]

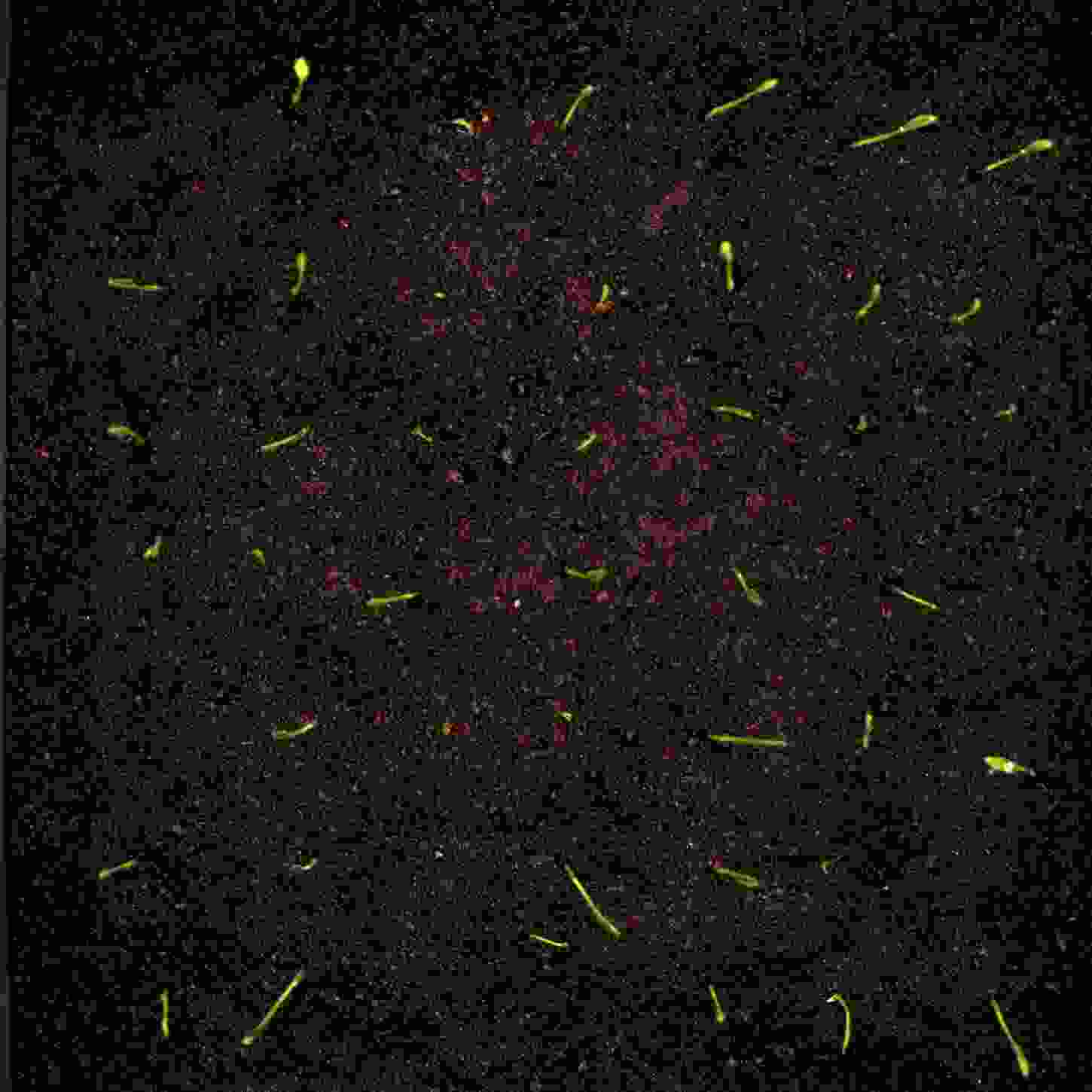

Supplement: Supplementary file 3 [file DataSheet3.zip › train1/1000-2024-3-19-7-12-49.JPG]

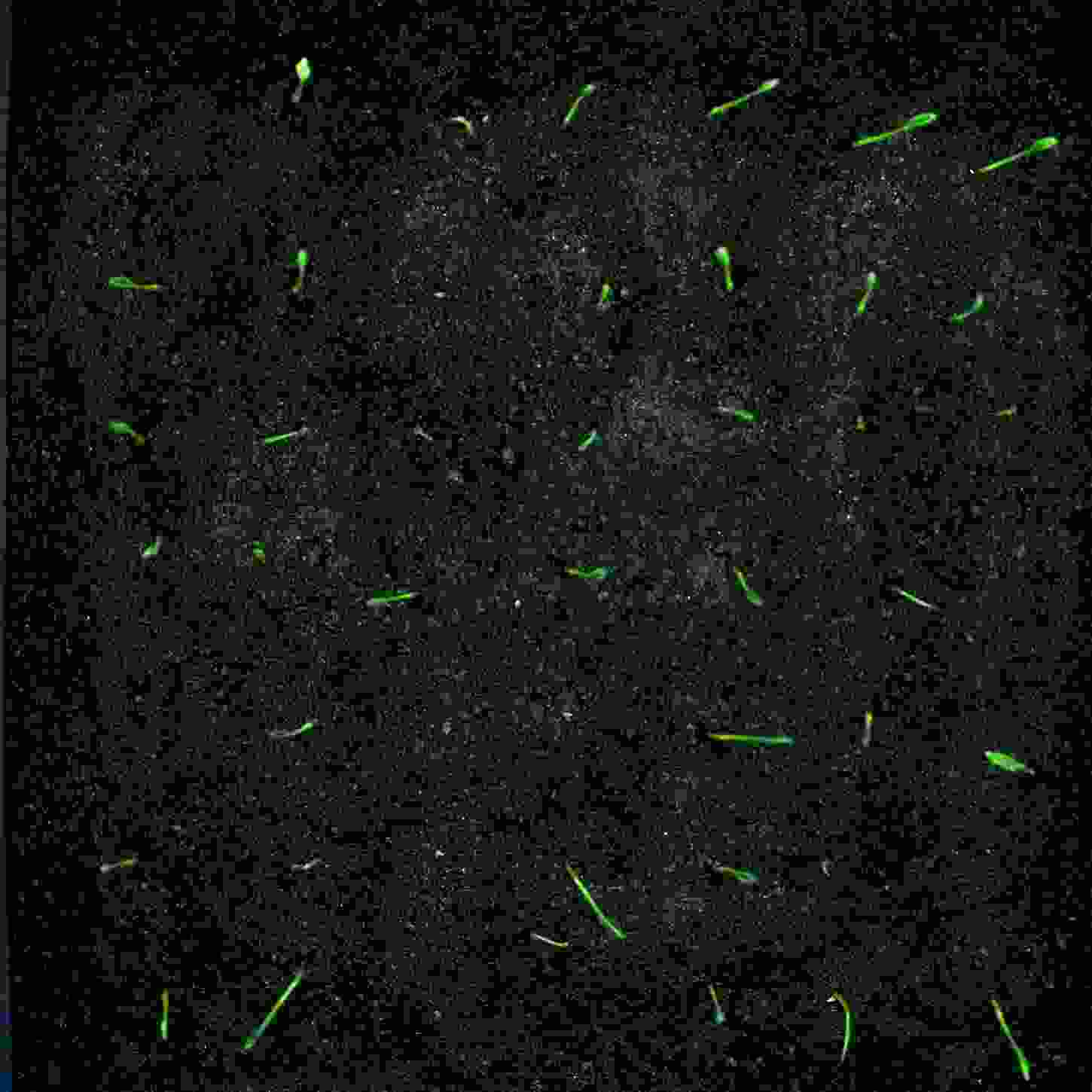

Supplement: Supplementary file 3 [file DataSheet3.zip › train1/1000-2024-3-19-9-45-36.JPG]

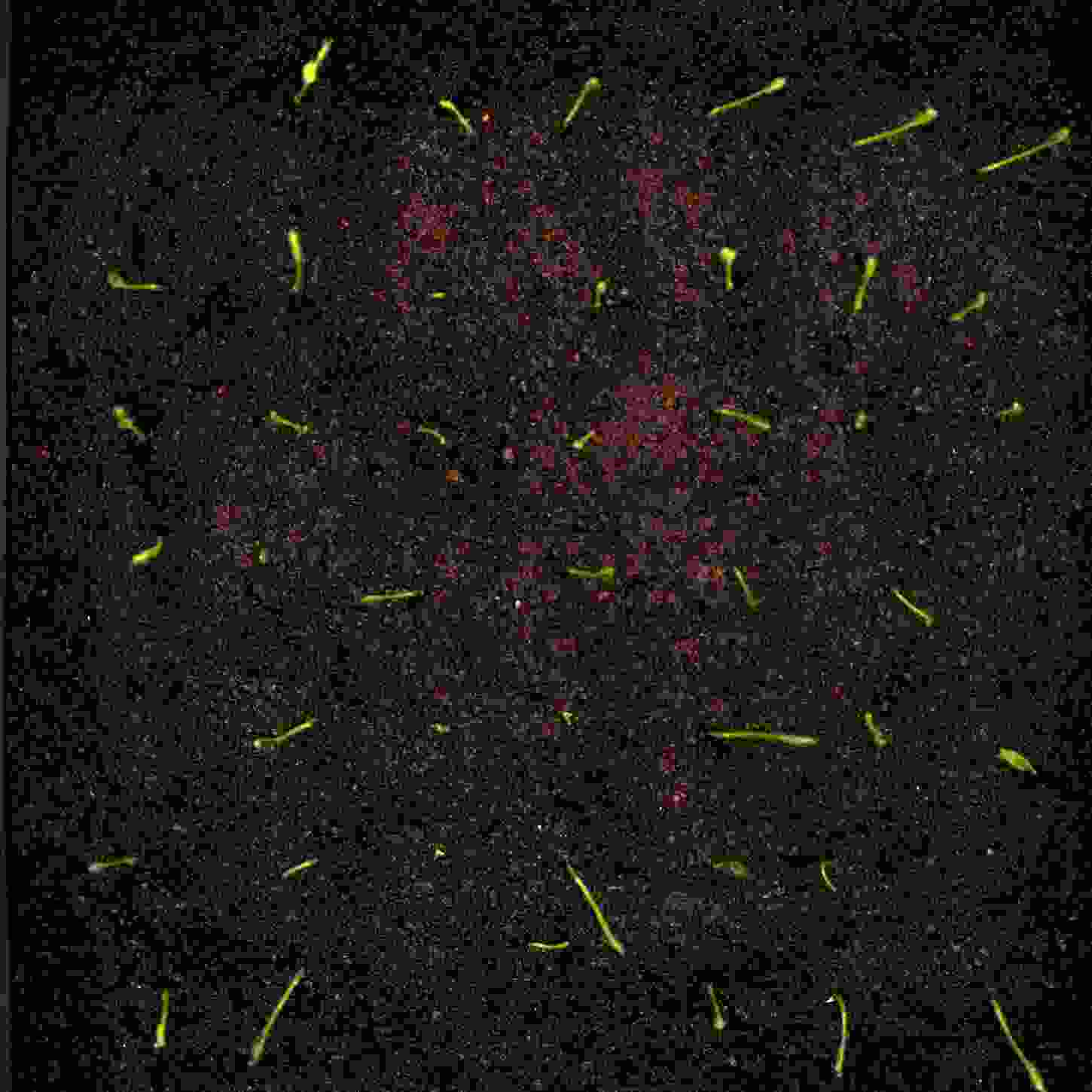

Supplement: Supplementary file 3 [file DataSheet3.zip › train1/1000-2024-3-20-1-1-54.JPG]

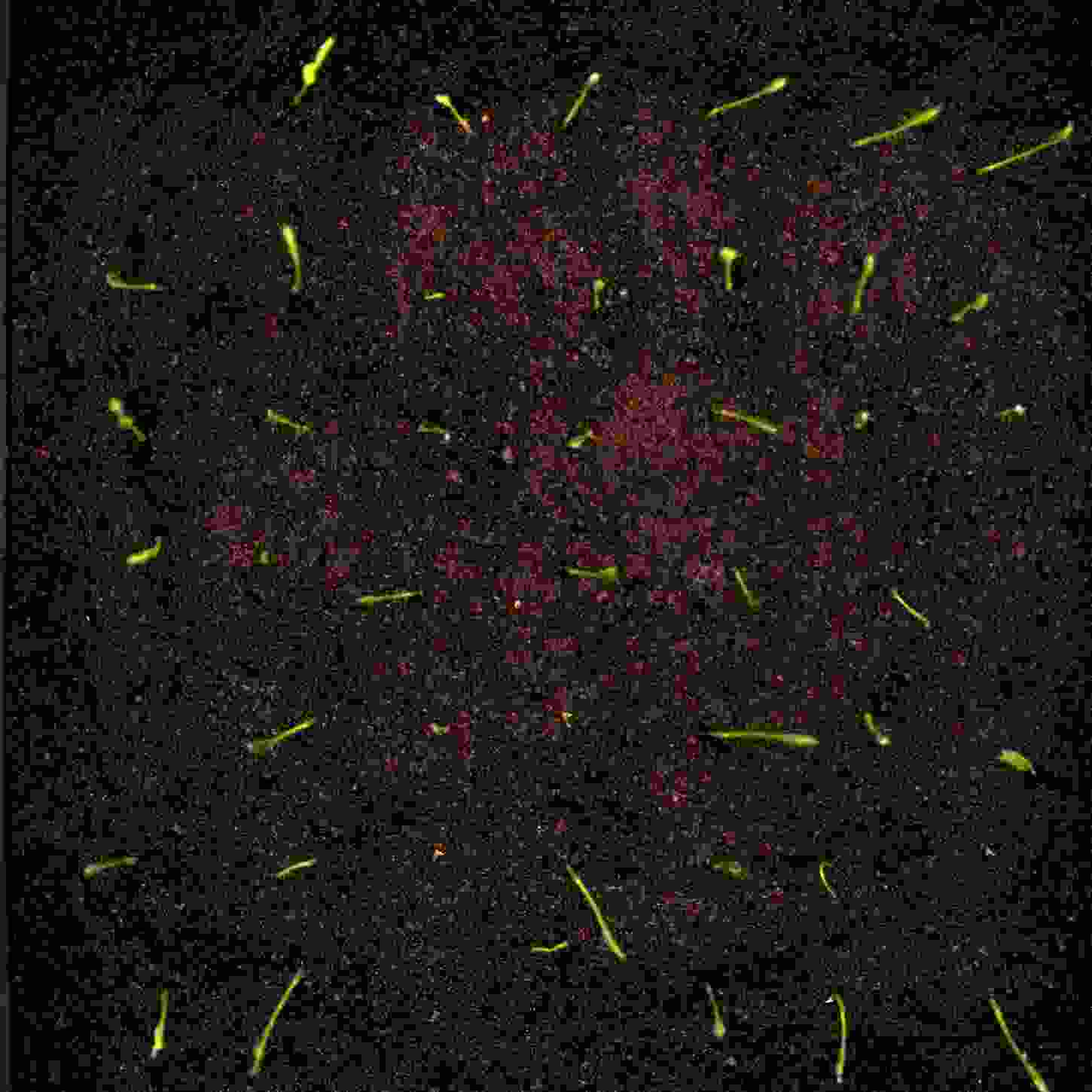

Supplement: Supplementary file 3 [file DataSheet3.zip › train1/1000-2024-3-20-6-7-1.JPG]

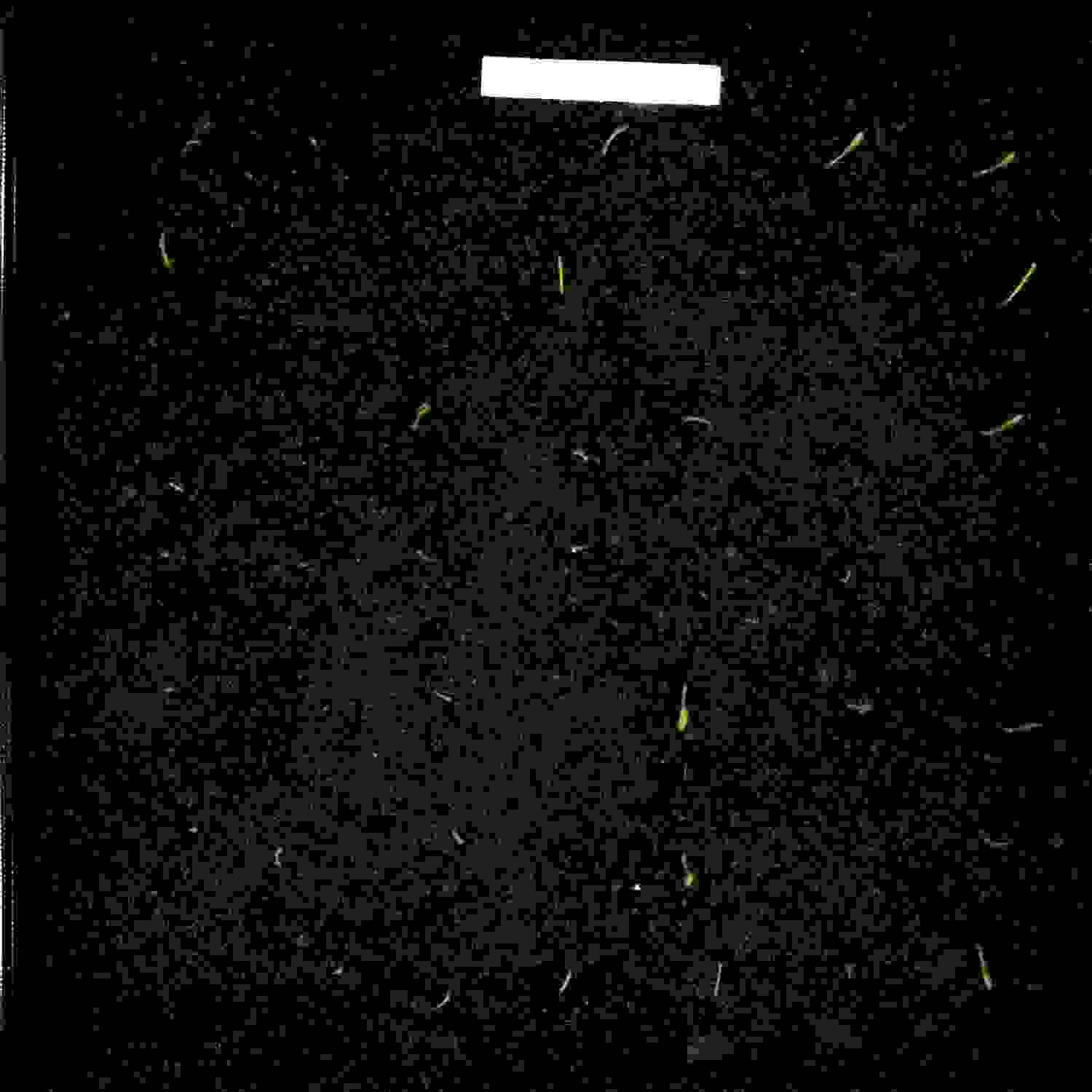

Supplement: Supplementary file 3 [file DataSheet3.zip › train1/10030-2024-3-18-15-49-11.JPG]

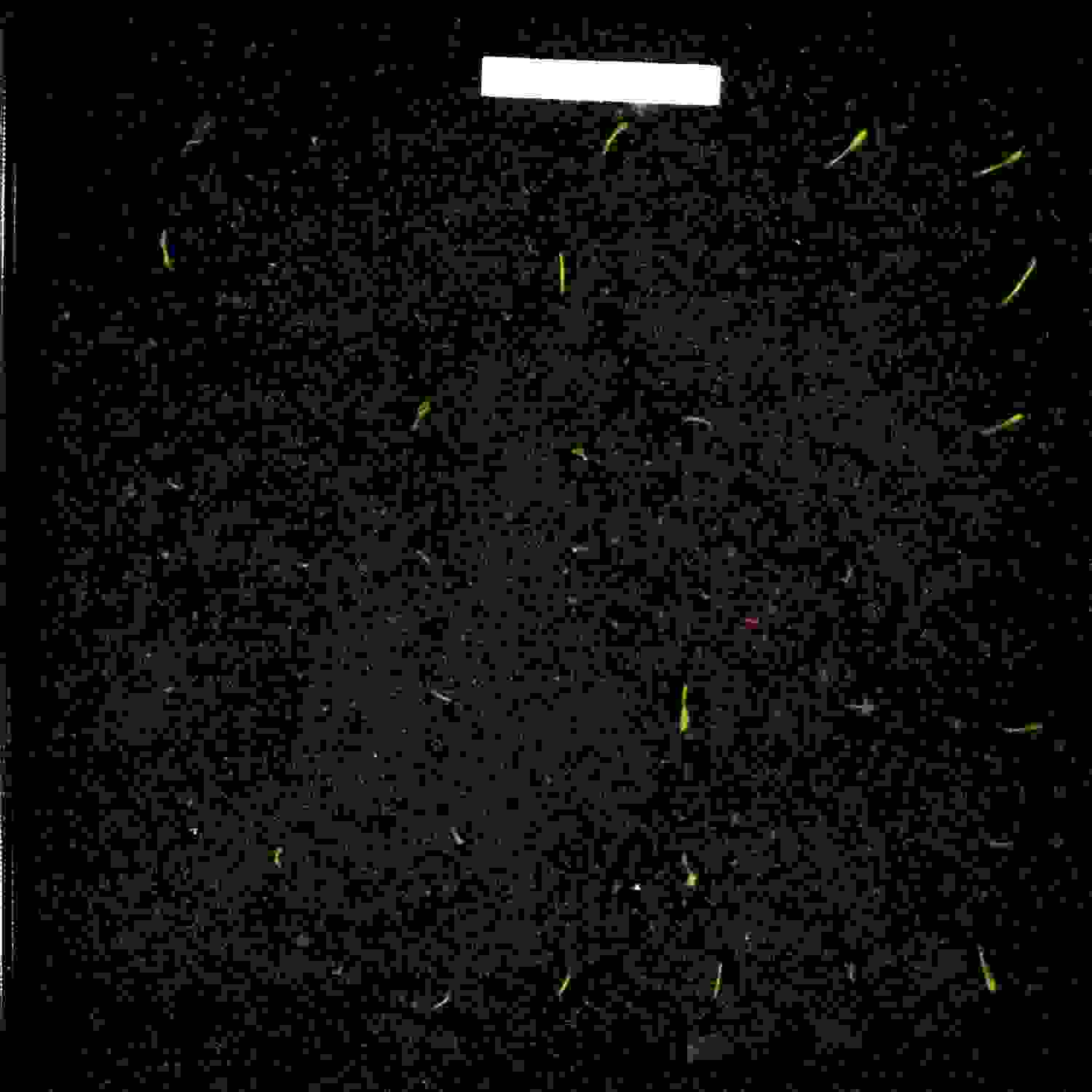

Supplement: Supplementary file 3 [file DataSheet3.zip › train1/10030-2024-3-18-18-23-22.JPG]

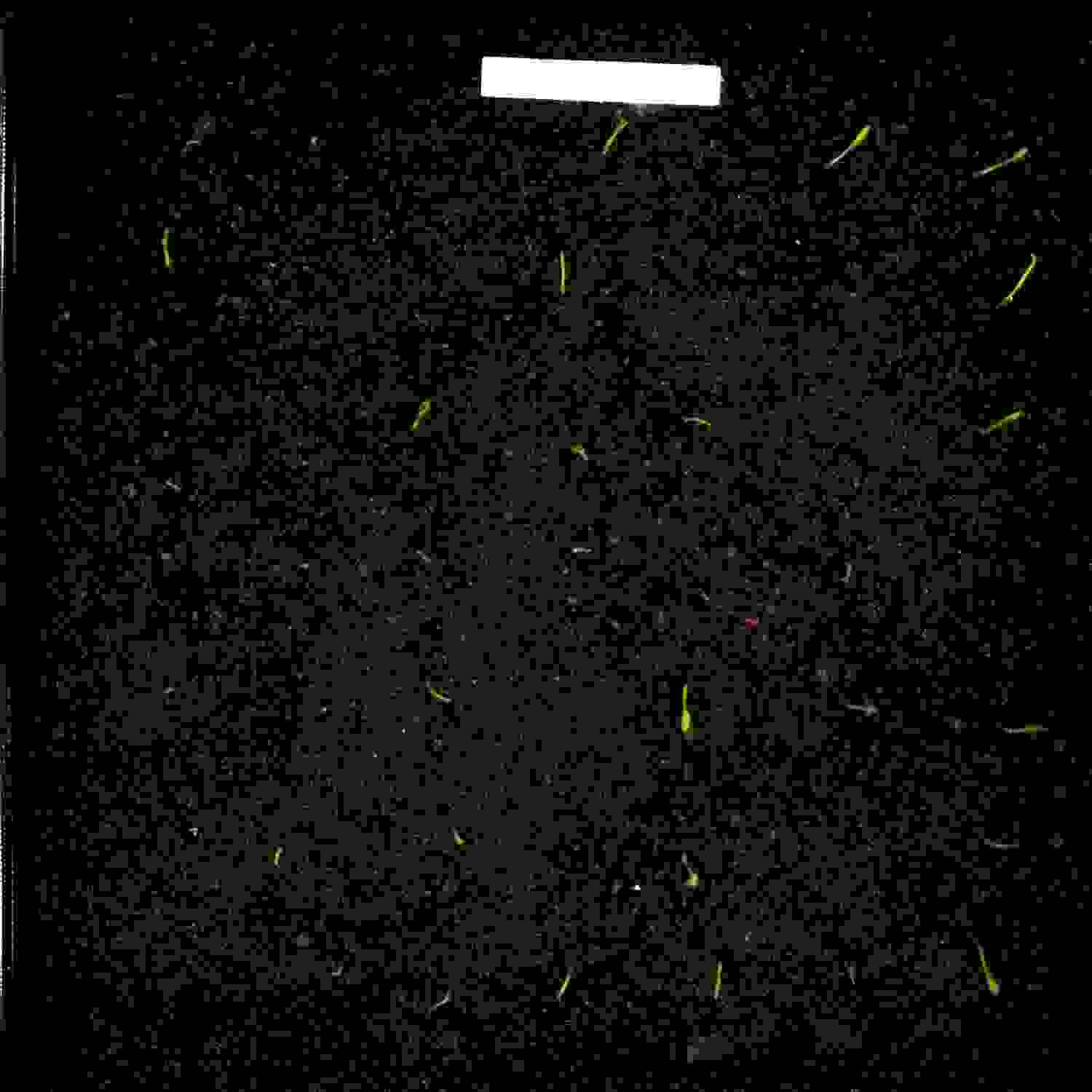

Supplement: Supplementary file 3 [file DataSheet3.zip › train1/10030-2024-3-18-20-58-23.JPG]

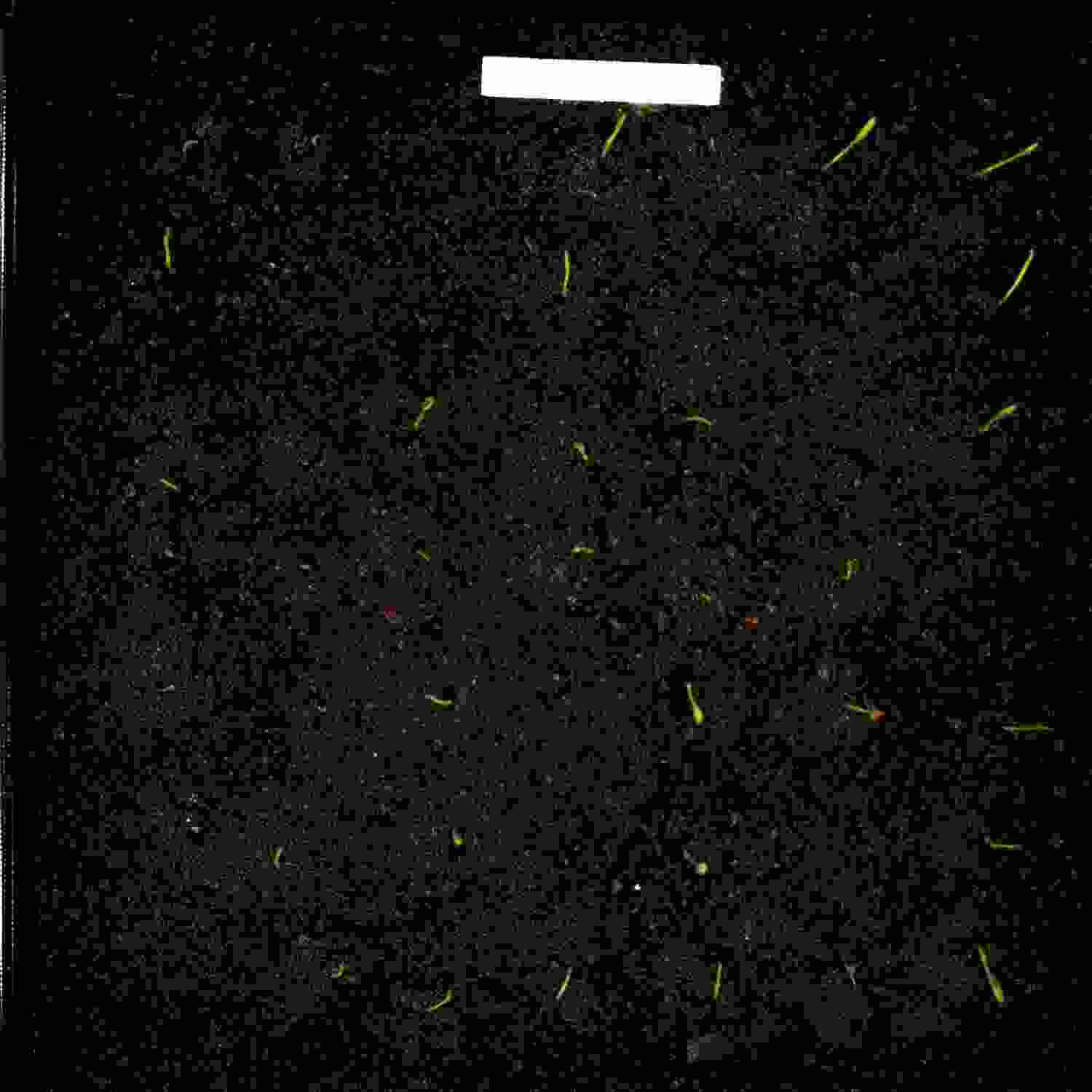

Supplement: Supplementary file 3 [file DataSheet3.zip › train1/10030-2024-3-18-23-32-38.JPG]

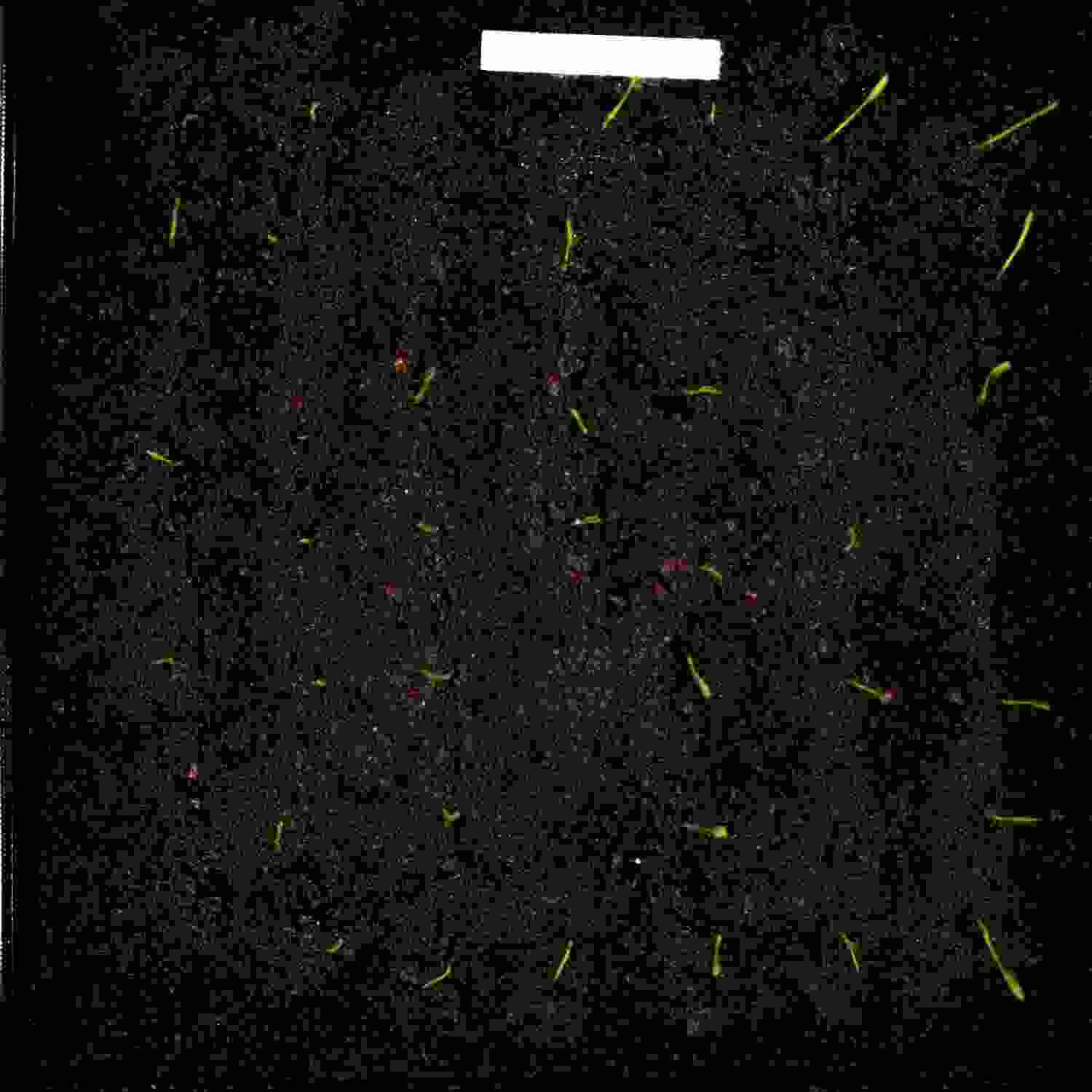

Supplement: Supplementary file 3 [file DataSheet3.zip › train1/10030-2024-3-19-12-18-46.JPG]

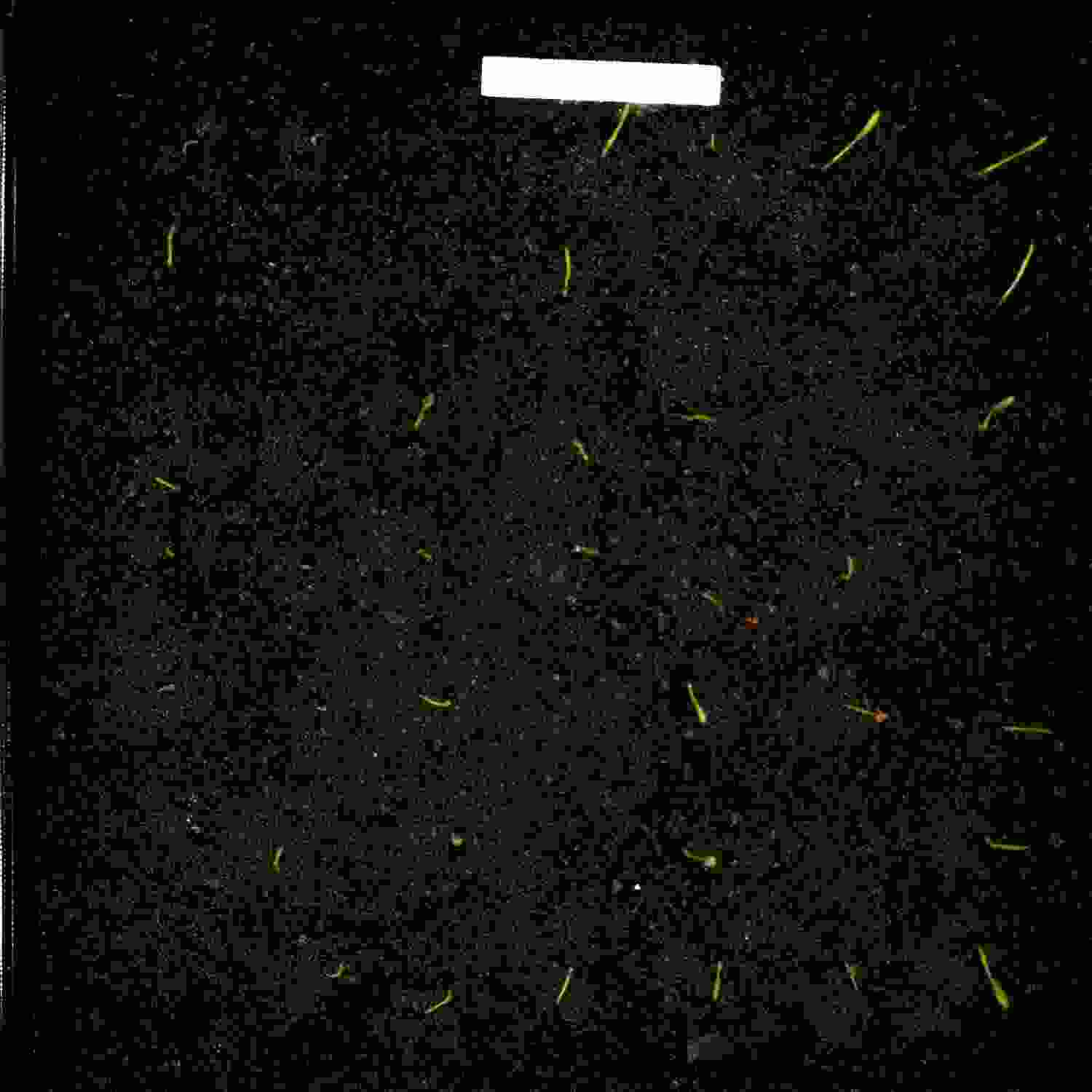

Supplement: Supplementary file 3 [file DataSheet3.zip › train1/10030-2024-3-19-4-39-30.JPG]

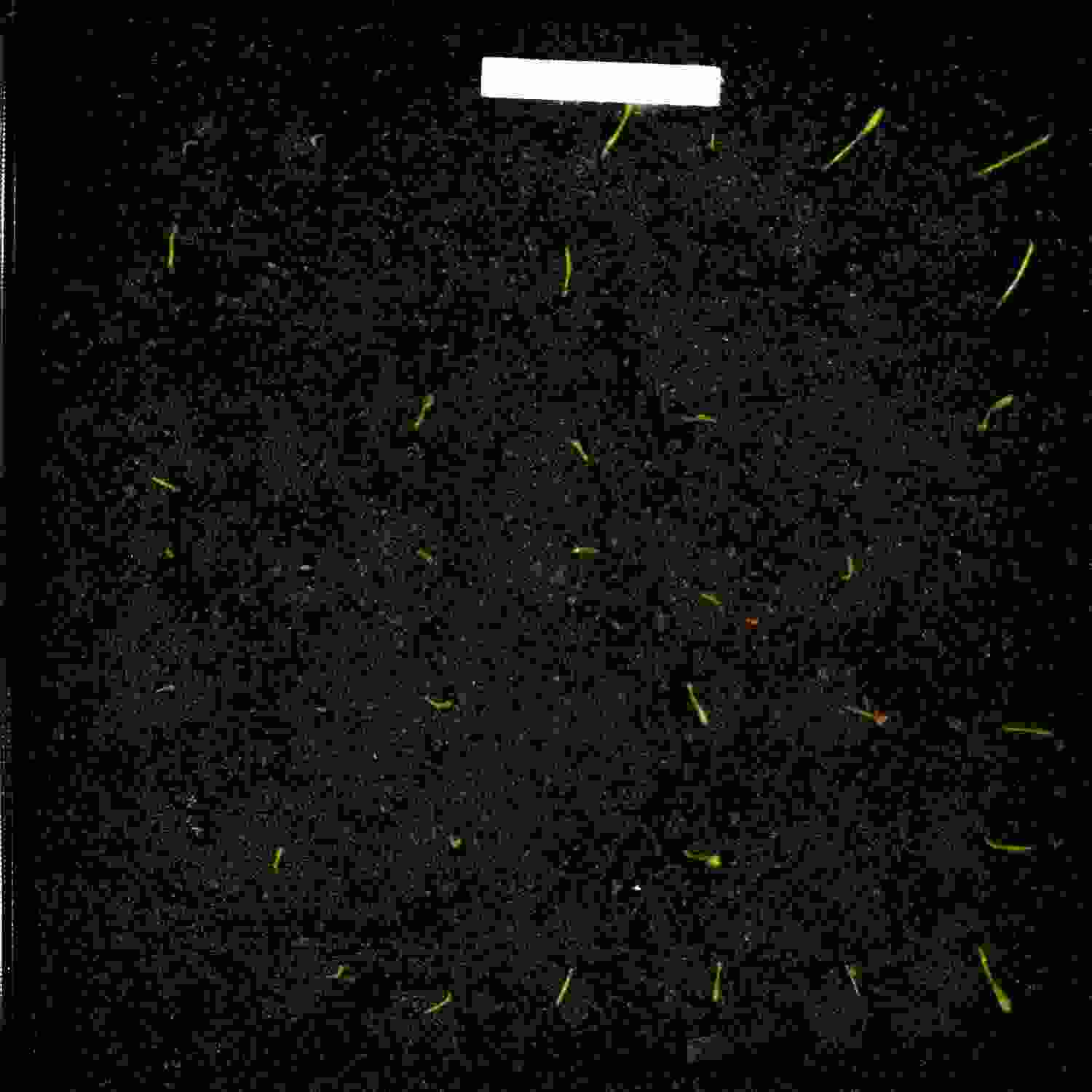

Supplement: Supplementary file 3 [file DataSheet3.zip › train1/10030-2024-3-19-7-13-20.JPG]

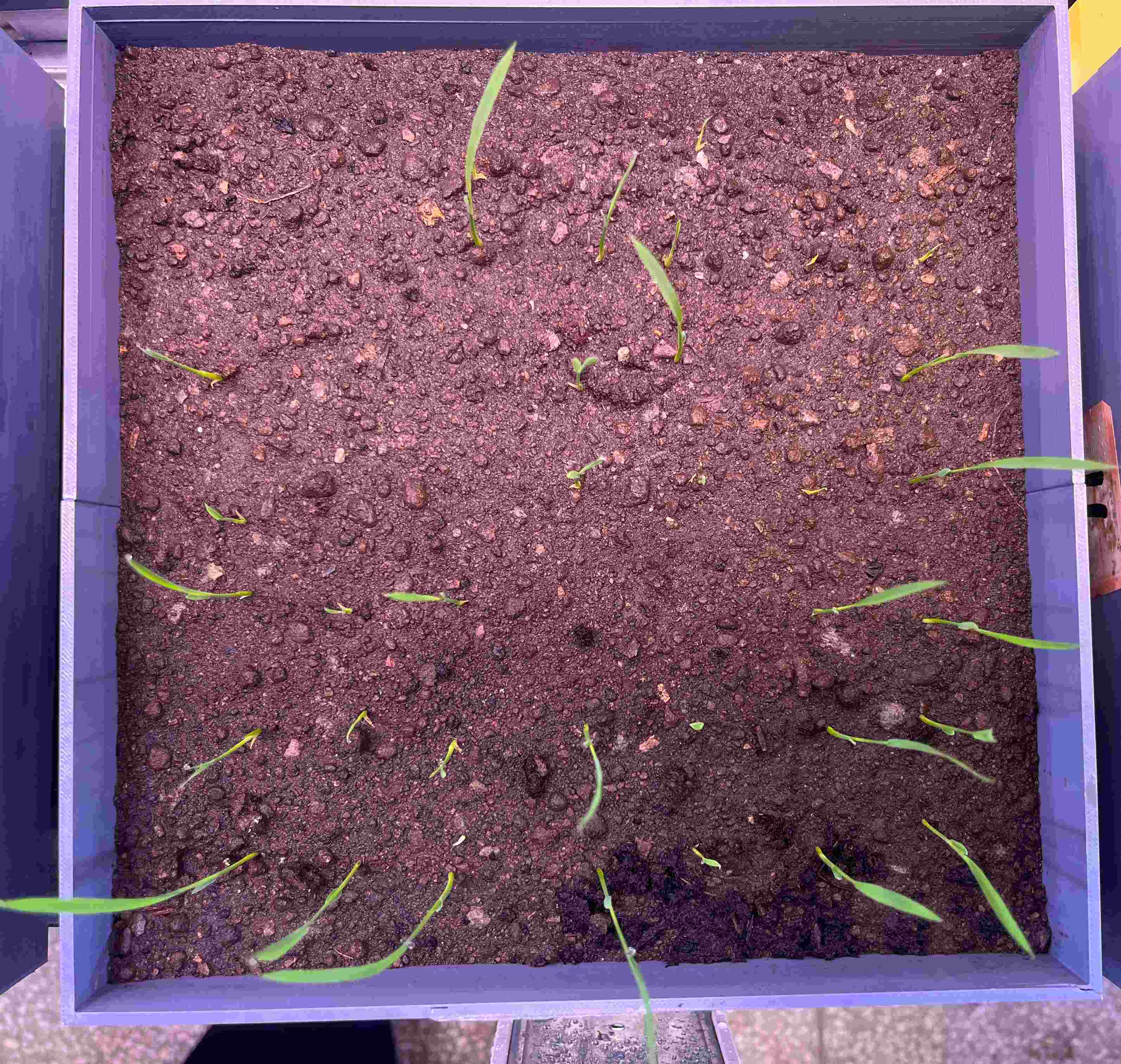

Supplement: Supplementary file 3 [file DataSheet3.zip › train1/12-1.JPG]

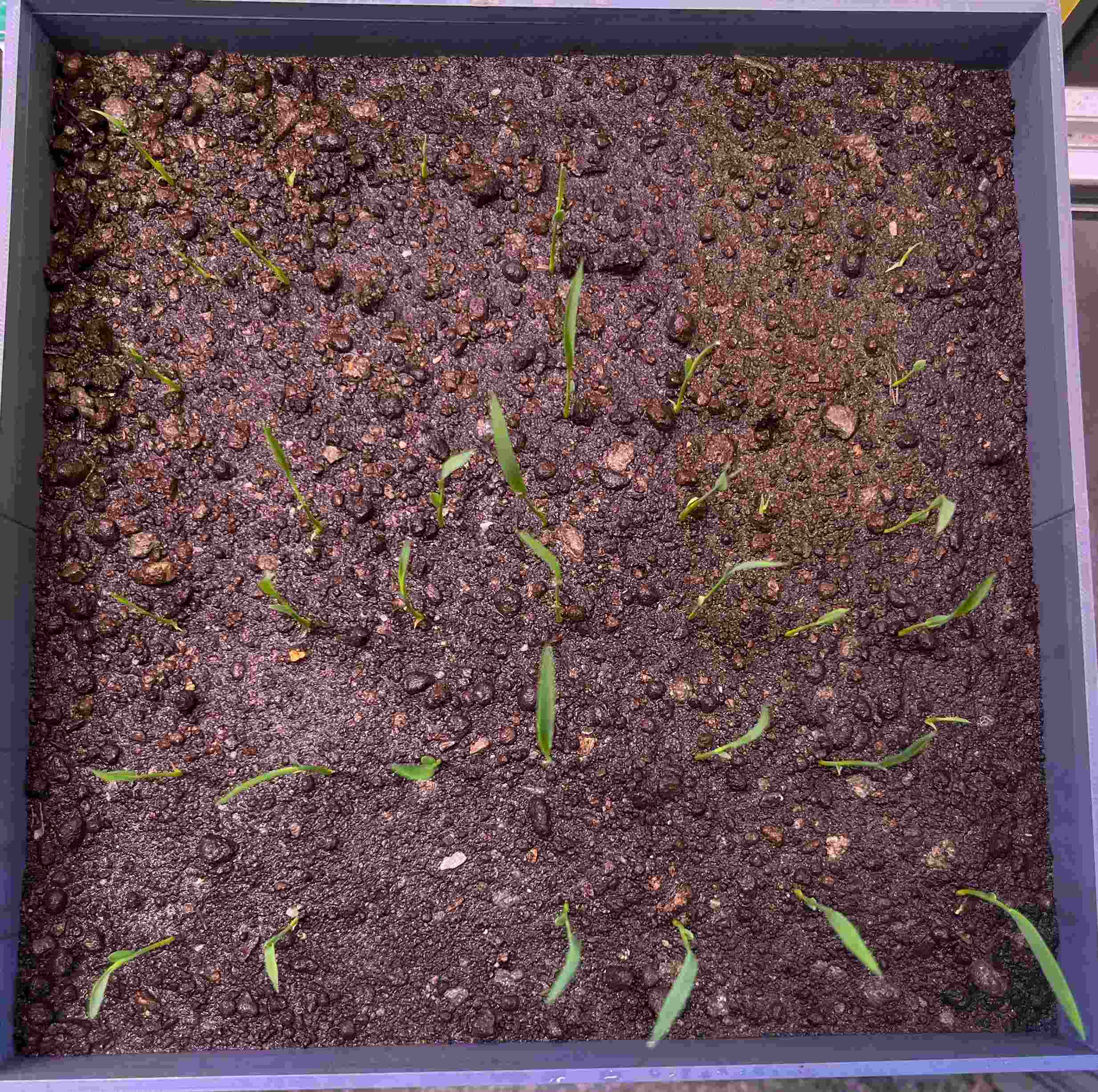

Supplement: Supplementary file 3 [file DataSheet3.zip › train1/12-3.JPG]

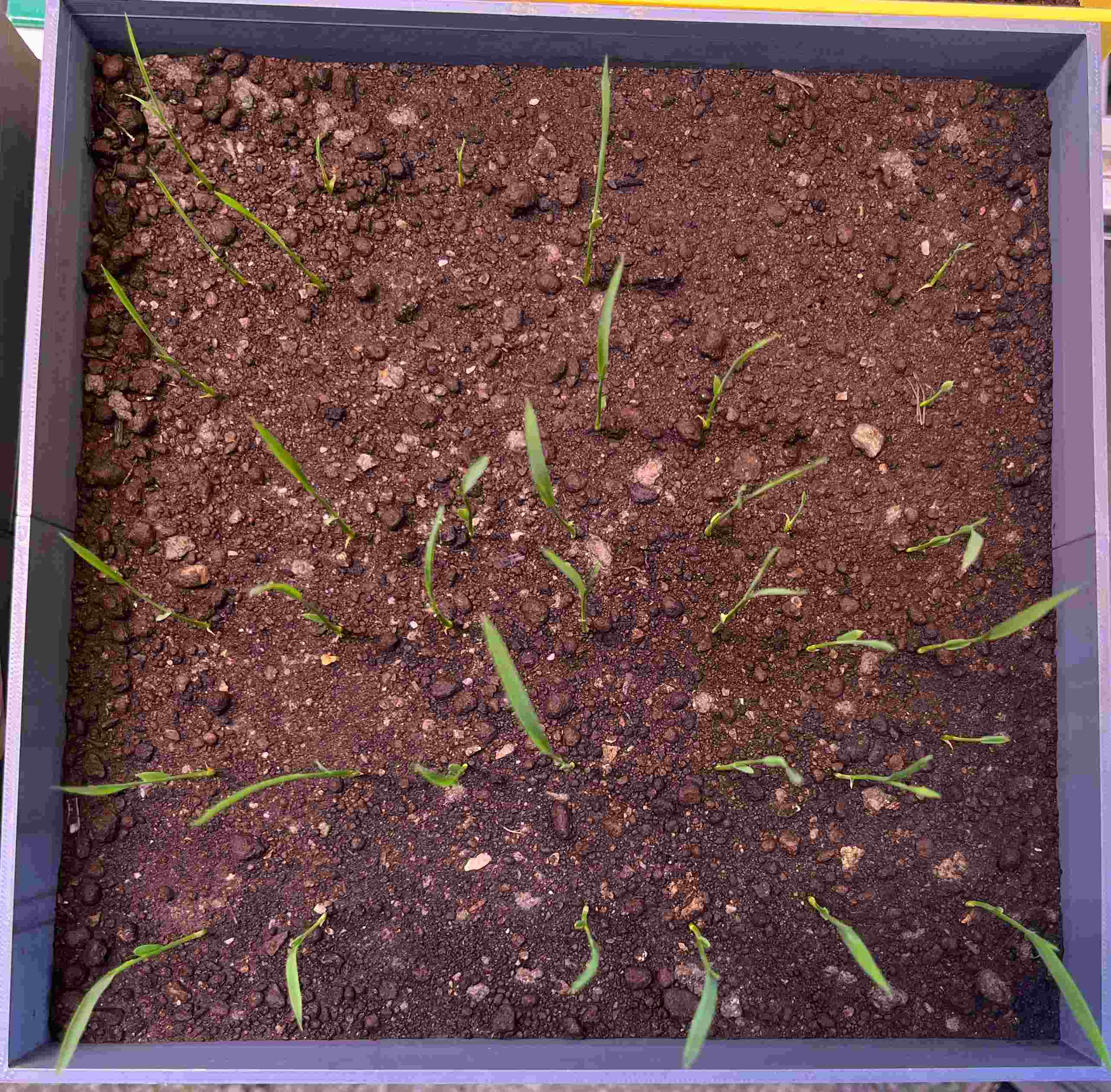

Supplement: Supplementary file 3 [file DataSheet3.zip › train1/13-1.JPG]

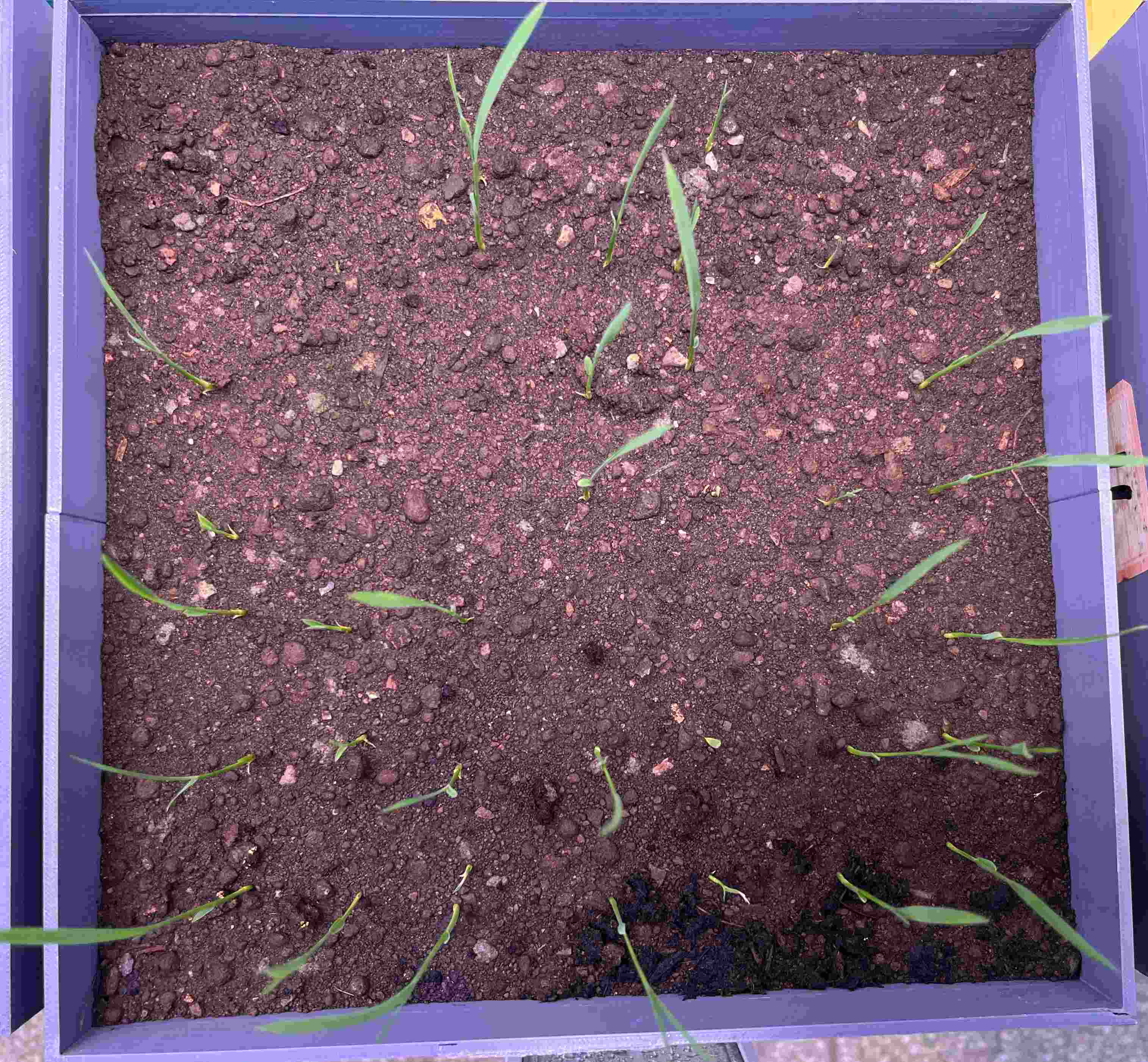

Supplement: Supplementary file 3 [file DataSheet3.zip › train1/13-2.JPG]

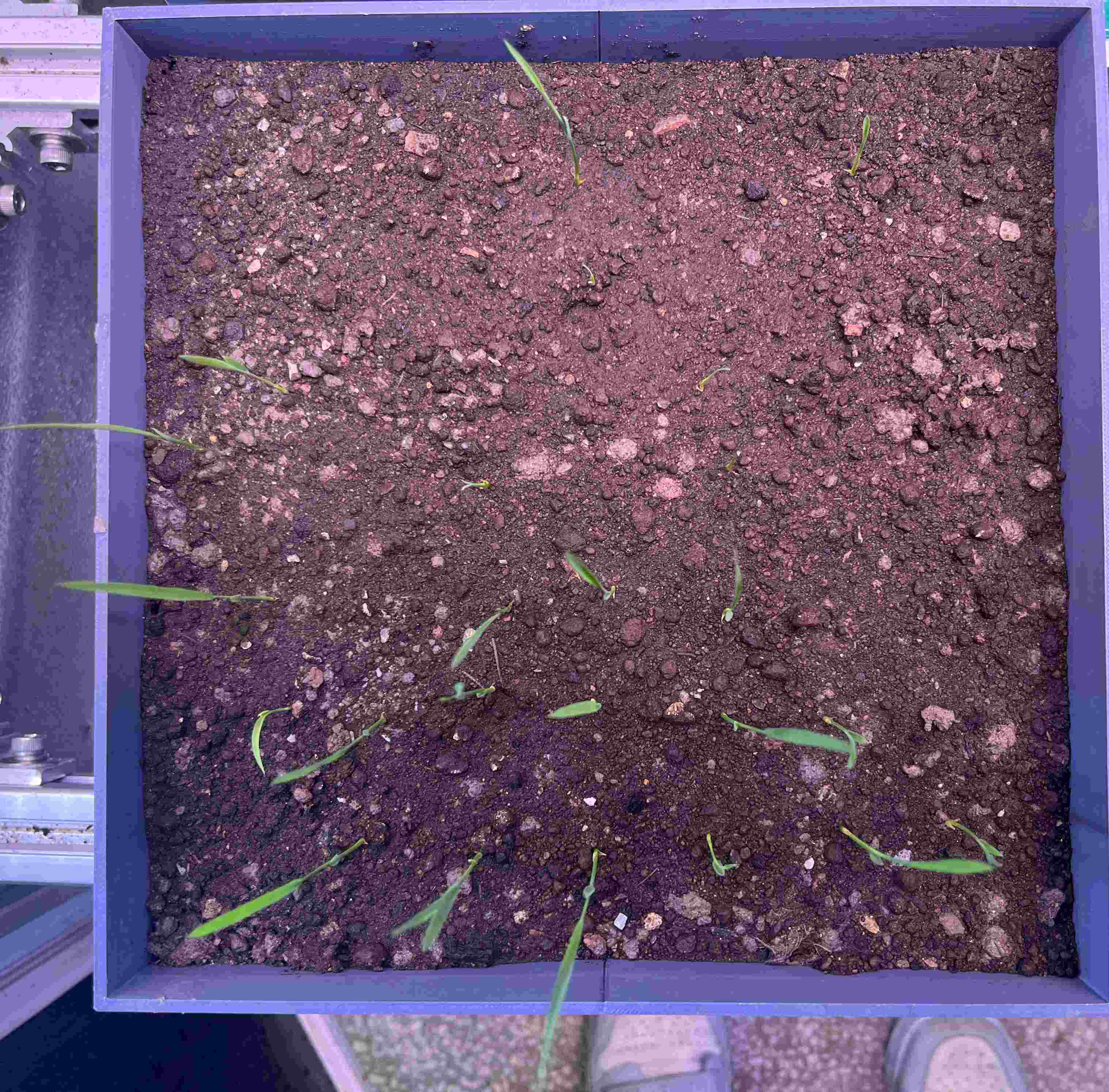

Supplement: Supplementary file 3 [file DataSheet3.zip › train1/13-4.JPG]

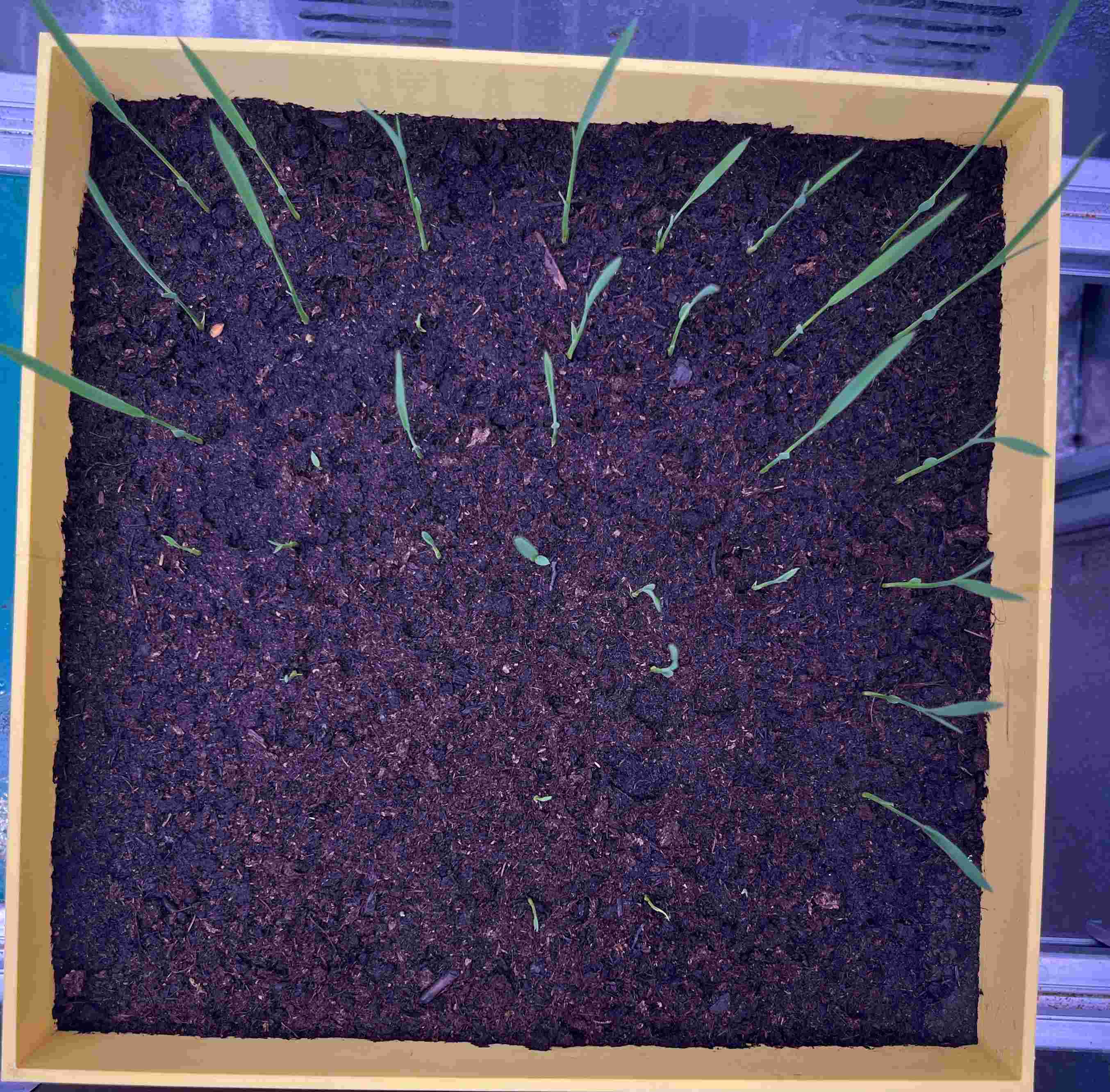

Supplement: Supplementary file 3 [file DataSheet3.zip › train1/13-5.JPG]

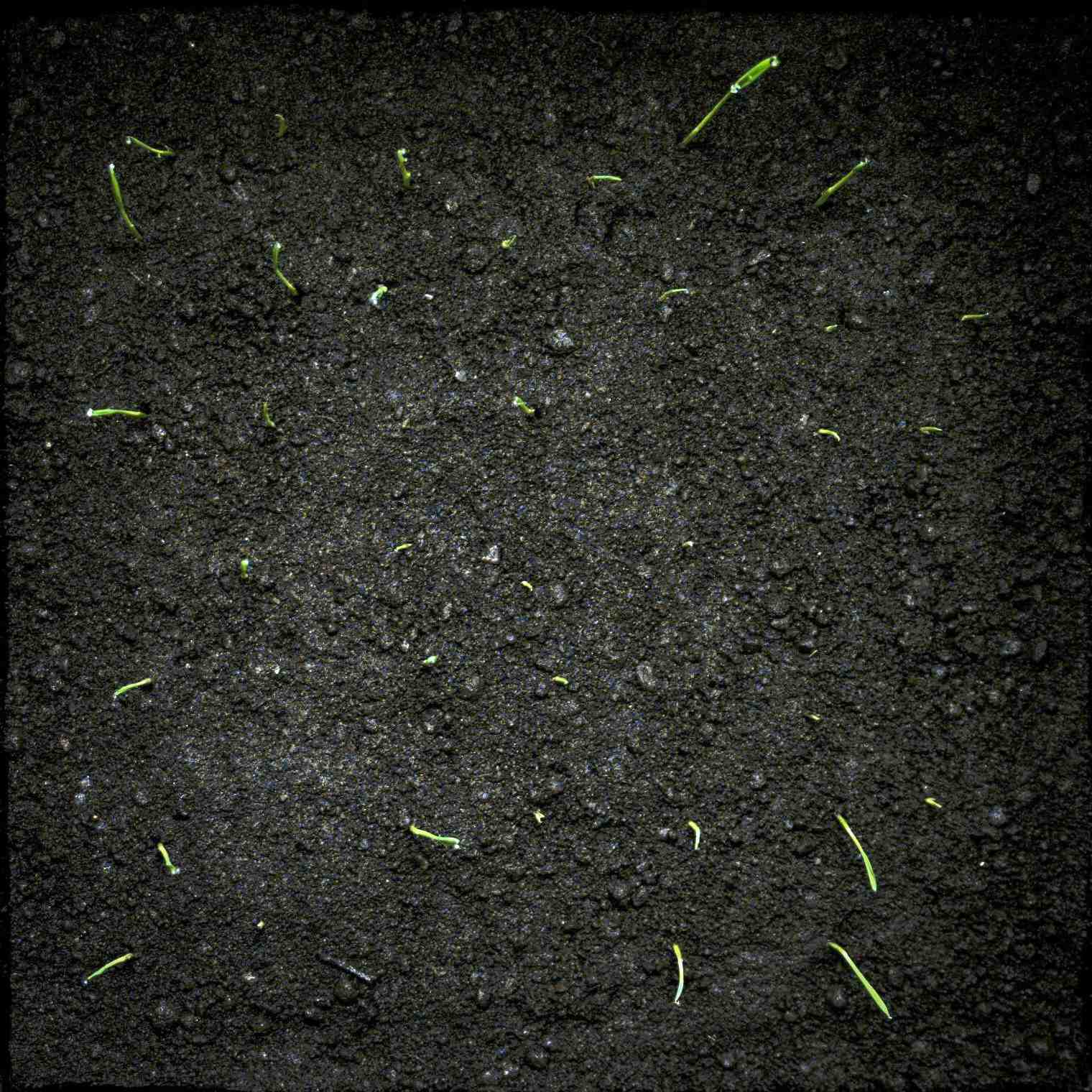

Supplement: Supplementary file 3 [file DataSheet3.zip › train1/200-2024-3-18-18-42-33.JPG]

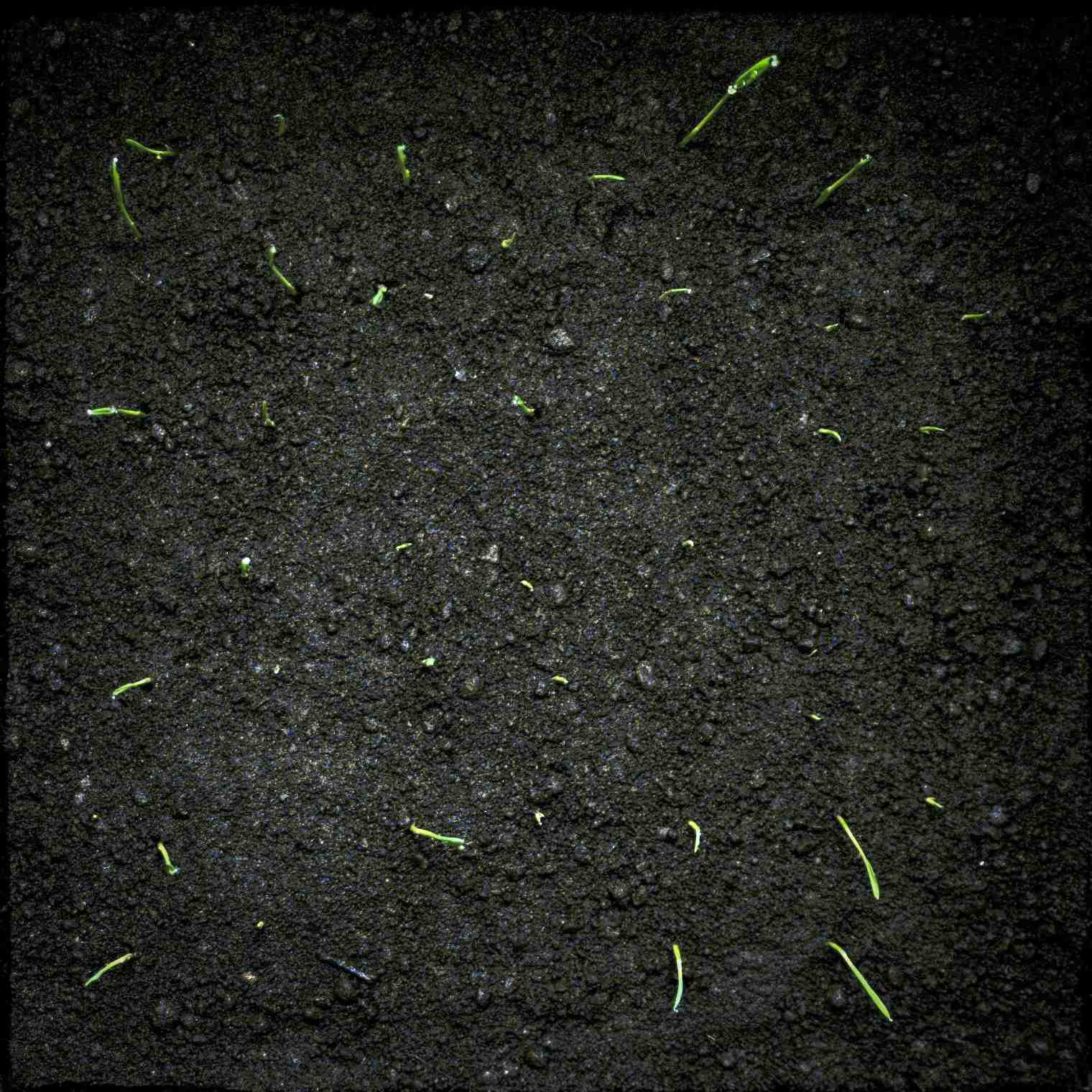

Supplement: Supplementary file 3 [file DataSheet3.zip › train1/200-2024-3-18-21-36-32.JPG]

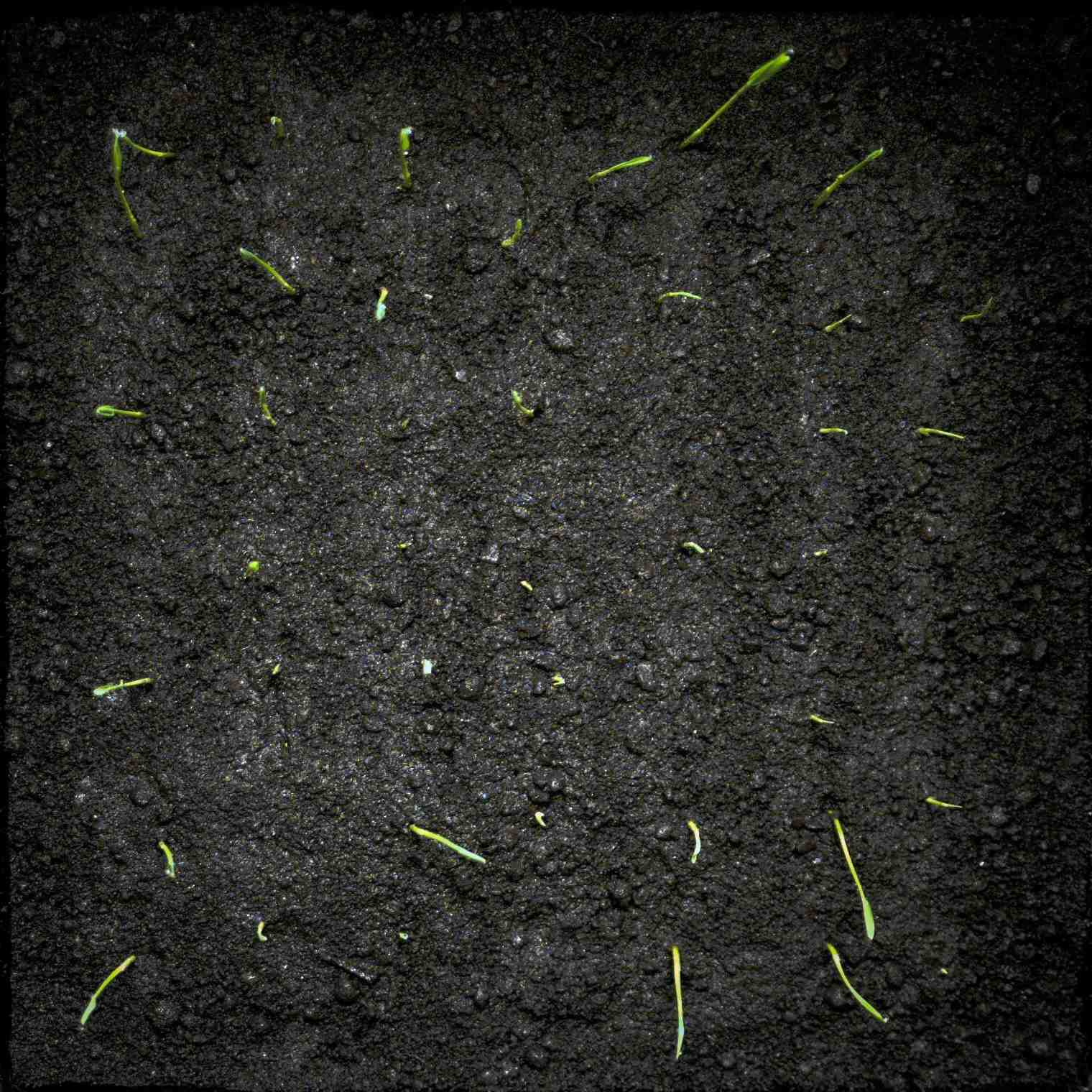

Supplement: Supplementary file 3 [file DataSheet3.zip › train1/200-2024-3-19-14-46-52.JPG]

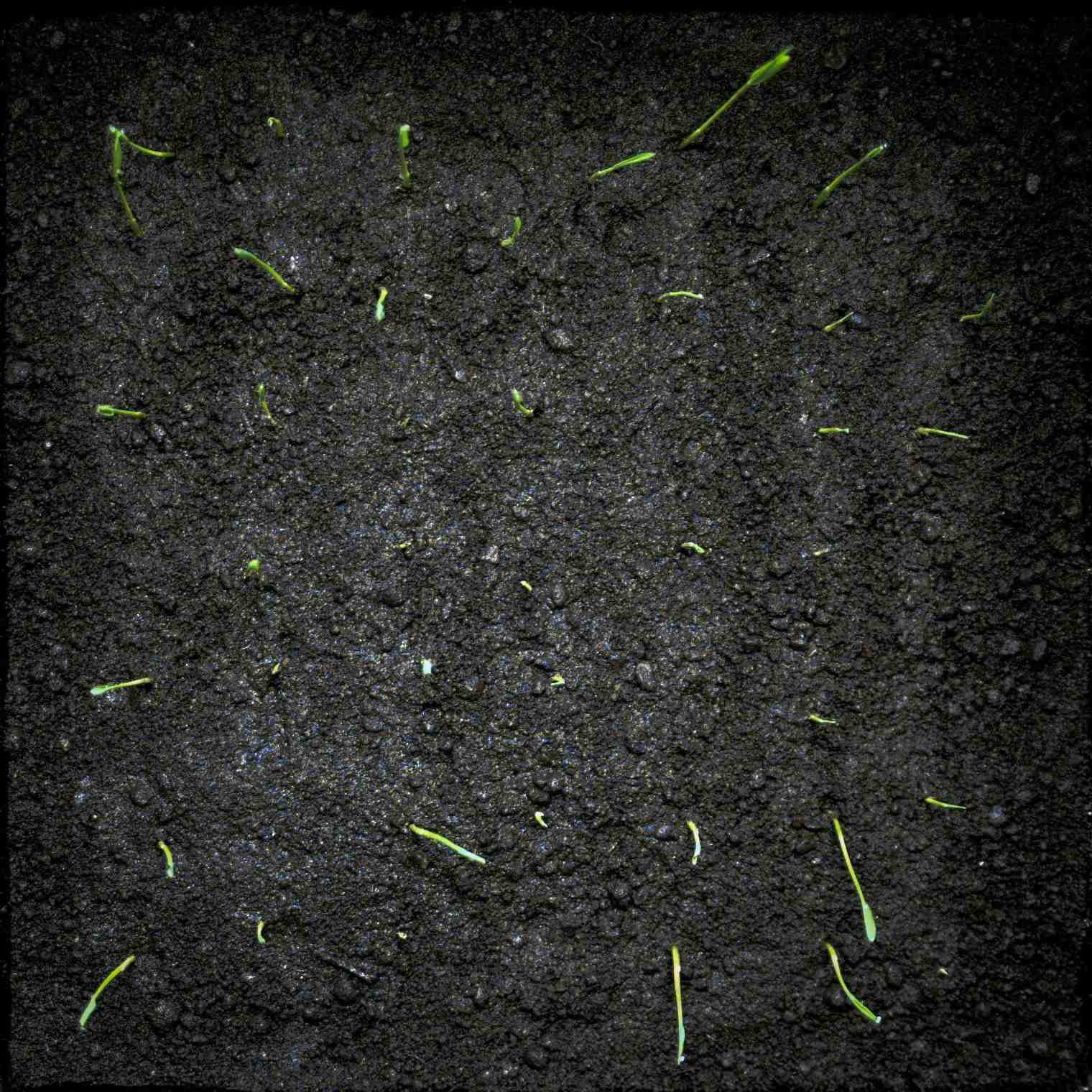

Supplement: Supplementary file 3 [file DataSheet3.zip › train1/200-2024-3-19-17-38-9.JPG]

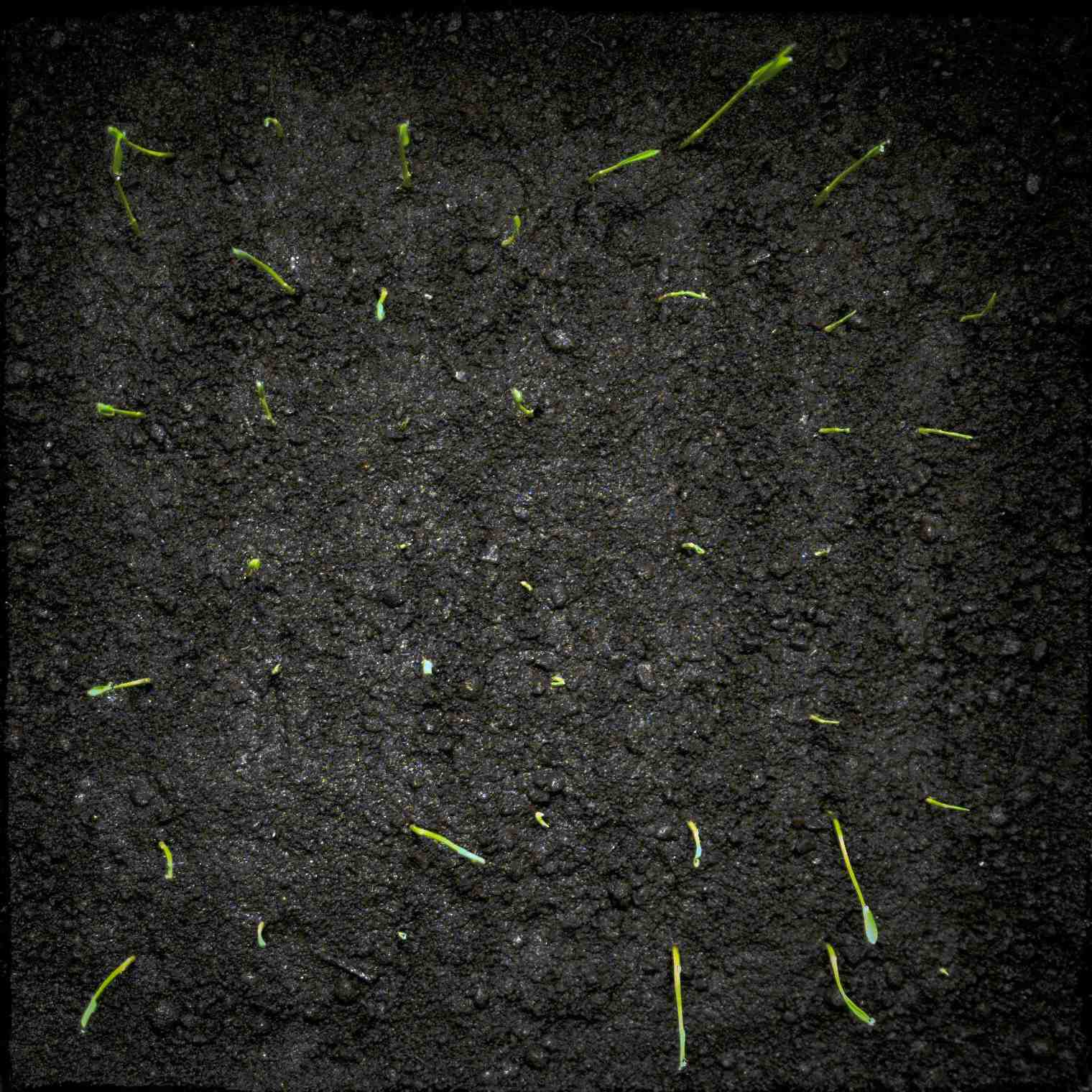

Supplement: Supplementary file 3 [file DataSheet3.zip › train1/200-2024-3-19-20-28-54.JPG]

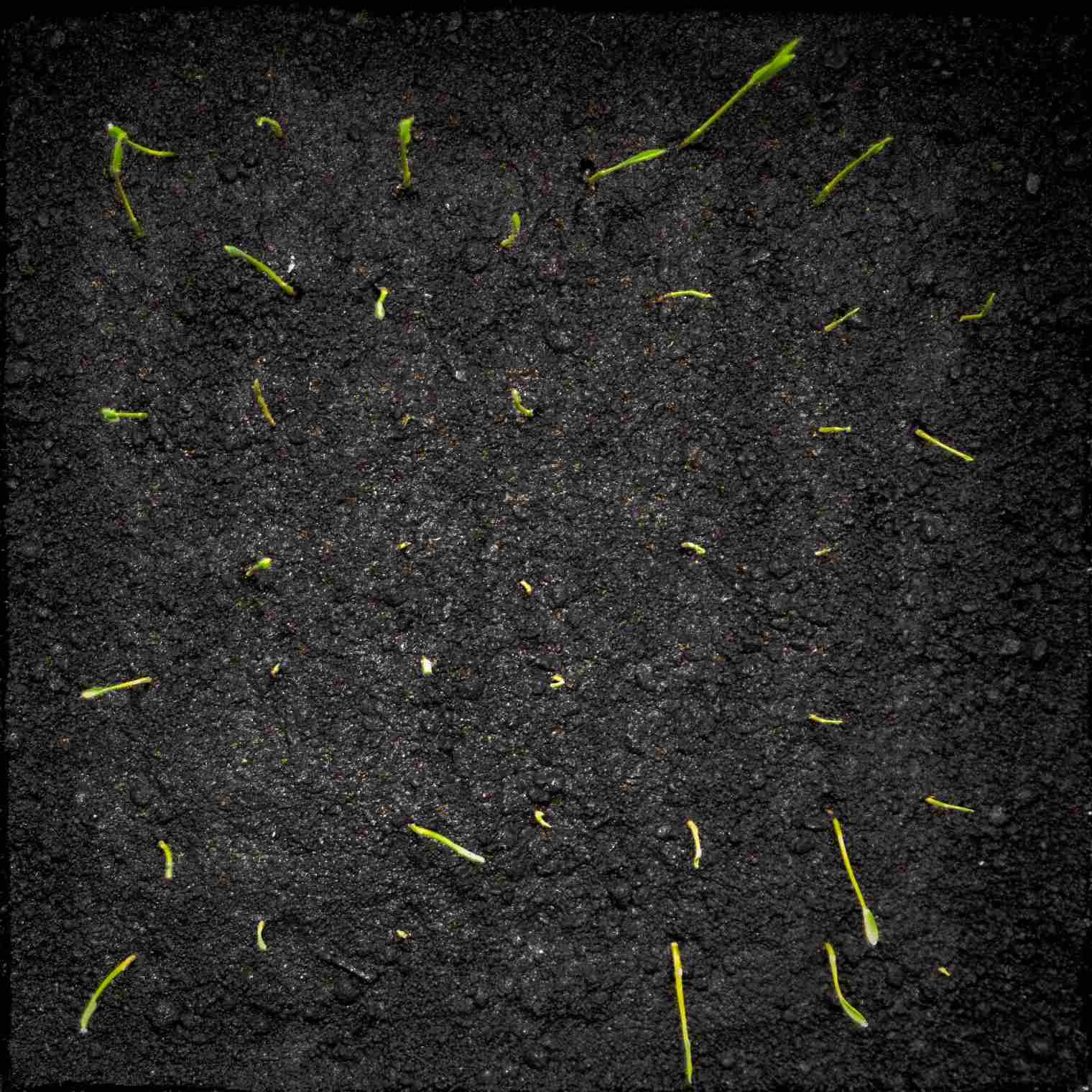

Supplement: Supplementary file 3 [file DataSheet3.zip › train1/200-2024-3-19-23-20-25.JPG]

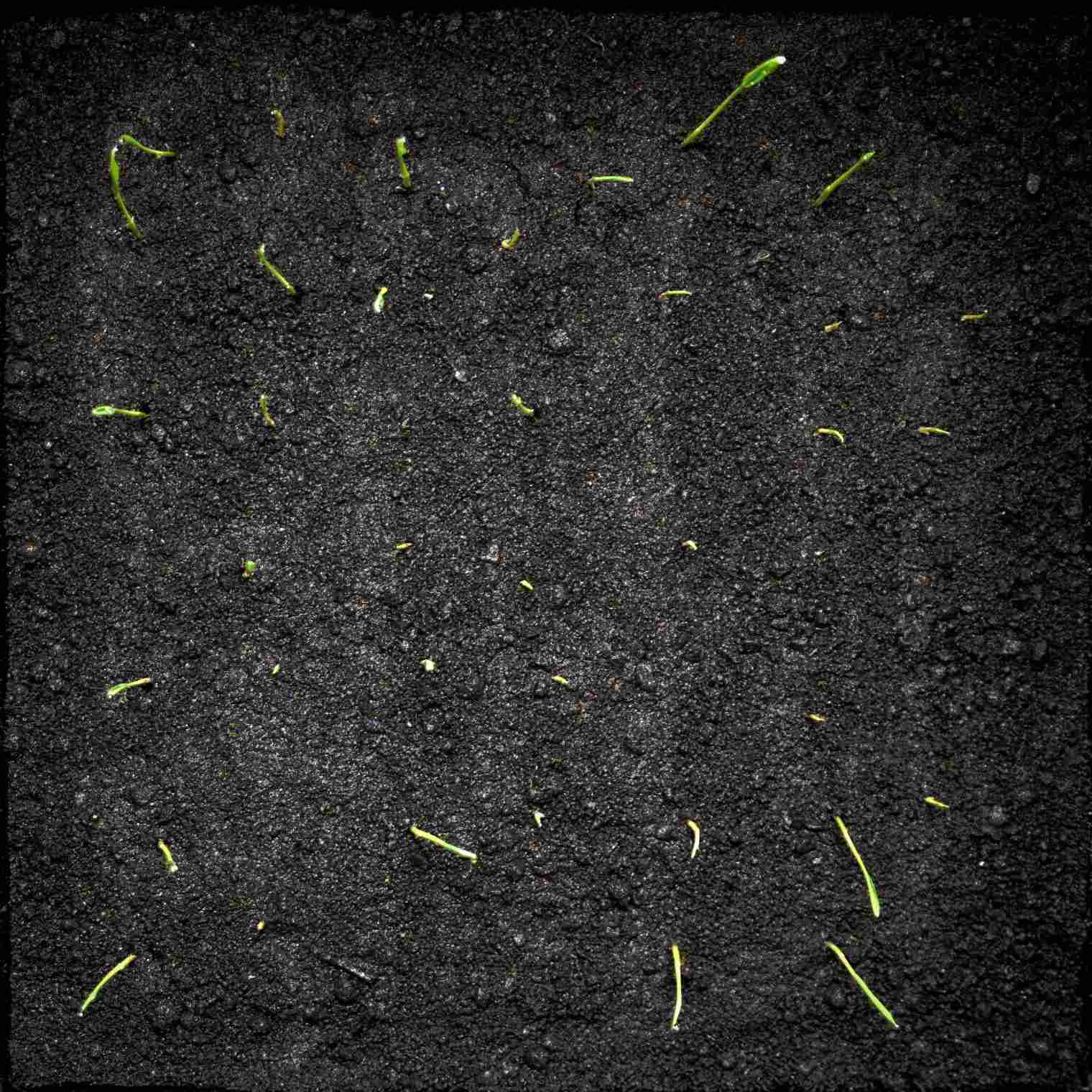

Supplement: Supplementary file 3 [file DataSheet3.zip › train1/200-2024-3-19-3-20-52.JPG]

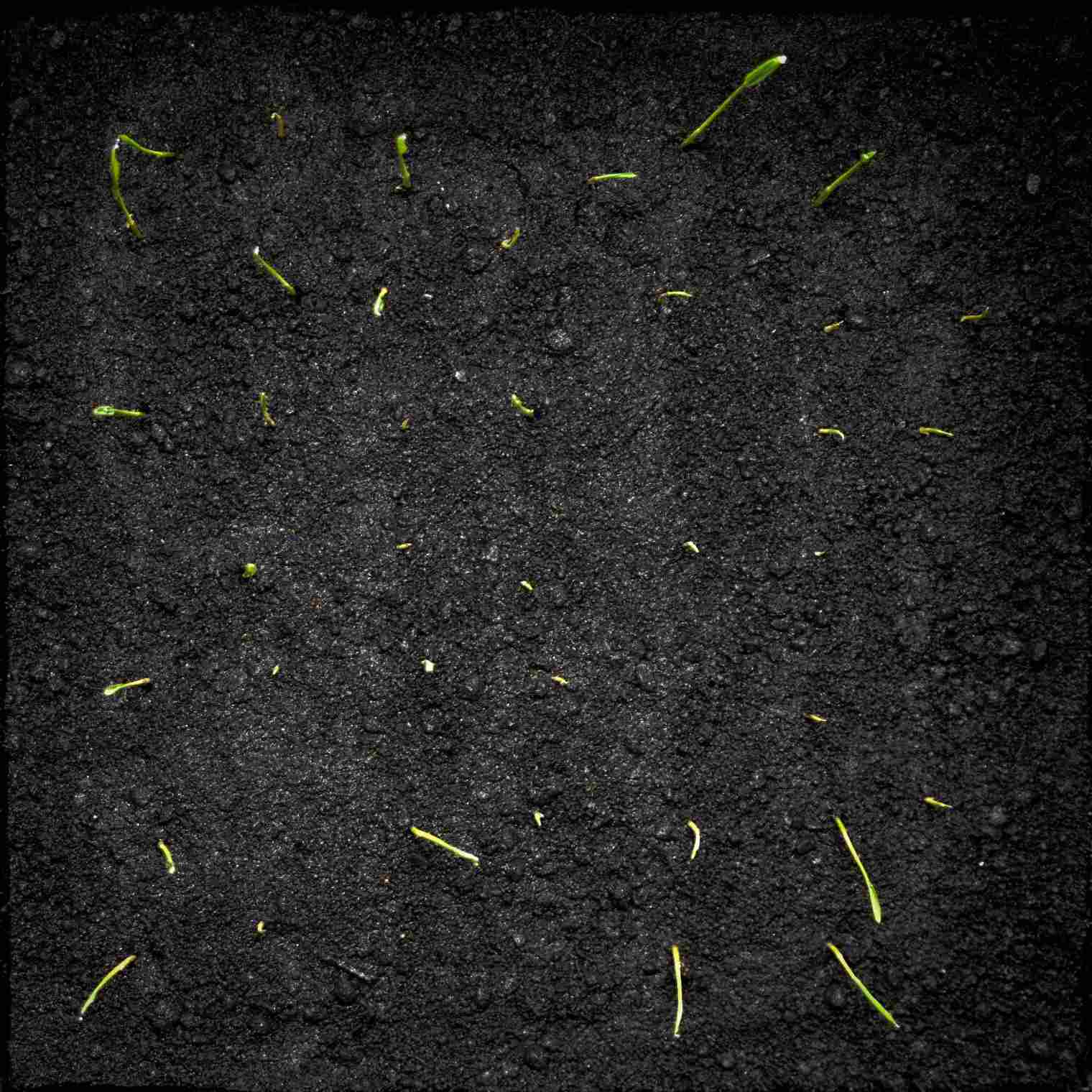

Supplement: Supplementary file 3 [file DataSheet3.zip › train1/200-2024-3-19-6-12-37.JPG]

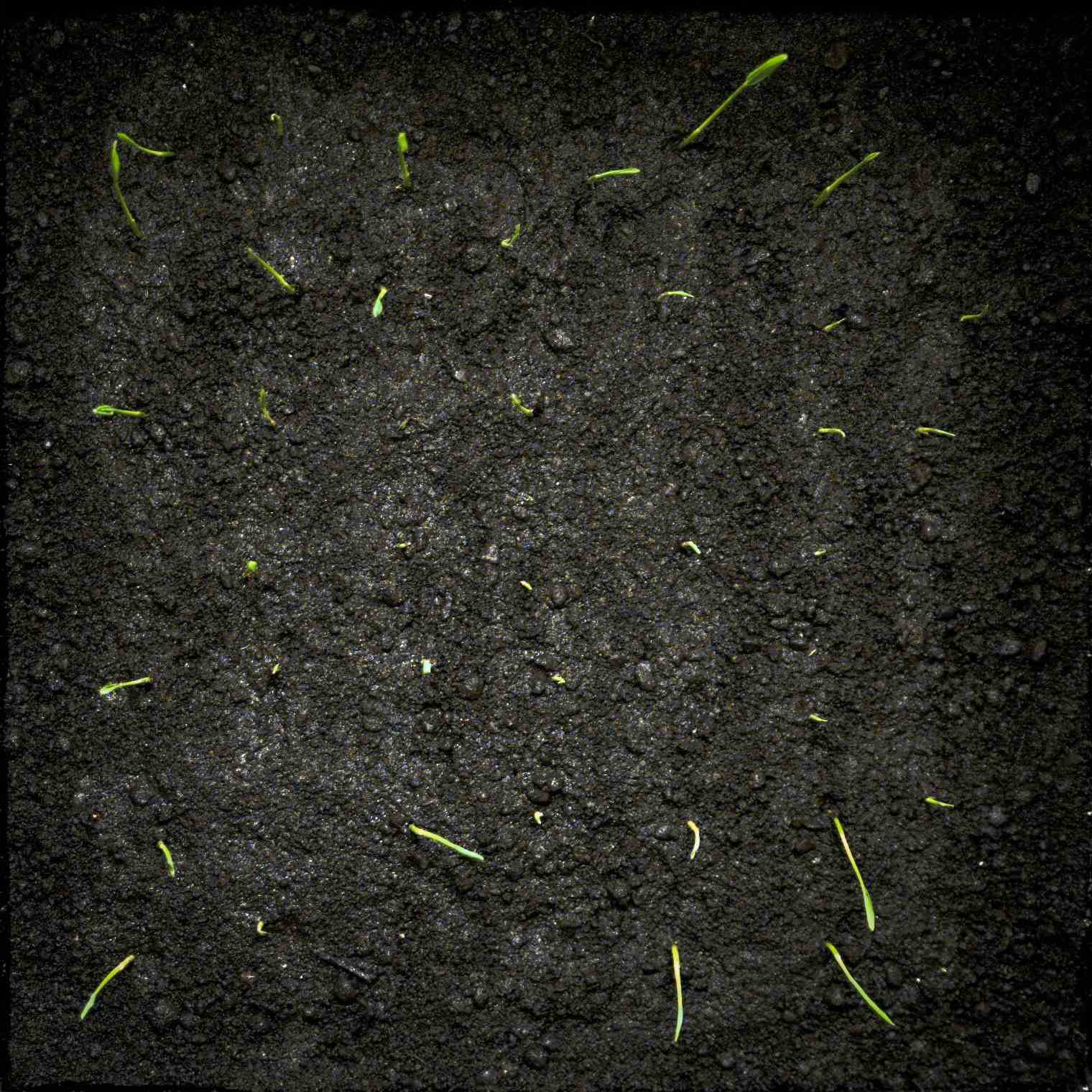

Supplement: Supplementary file 3 [file DataSheet3.zip › train1/200-2024-3-19-9-4-18.JPG]

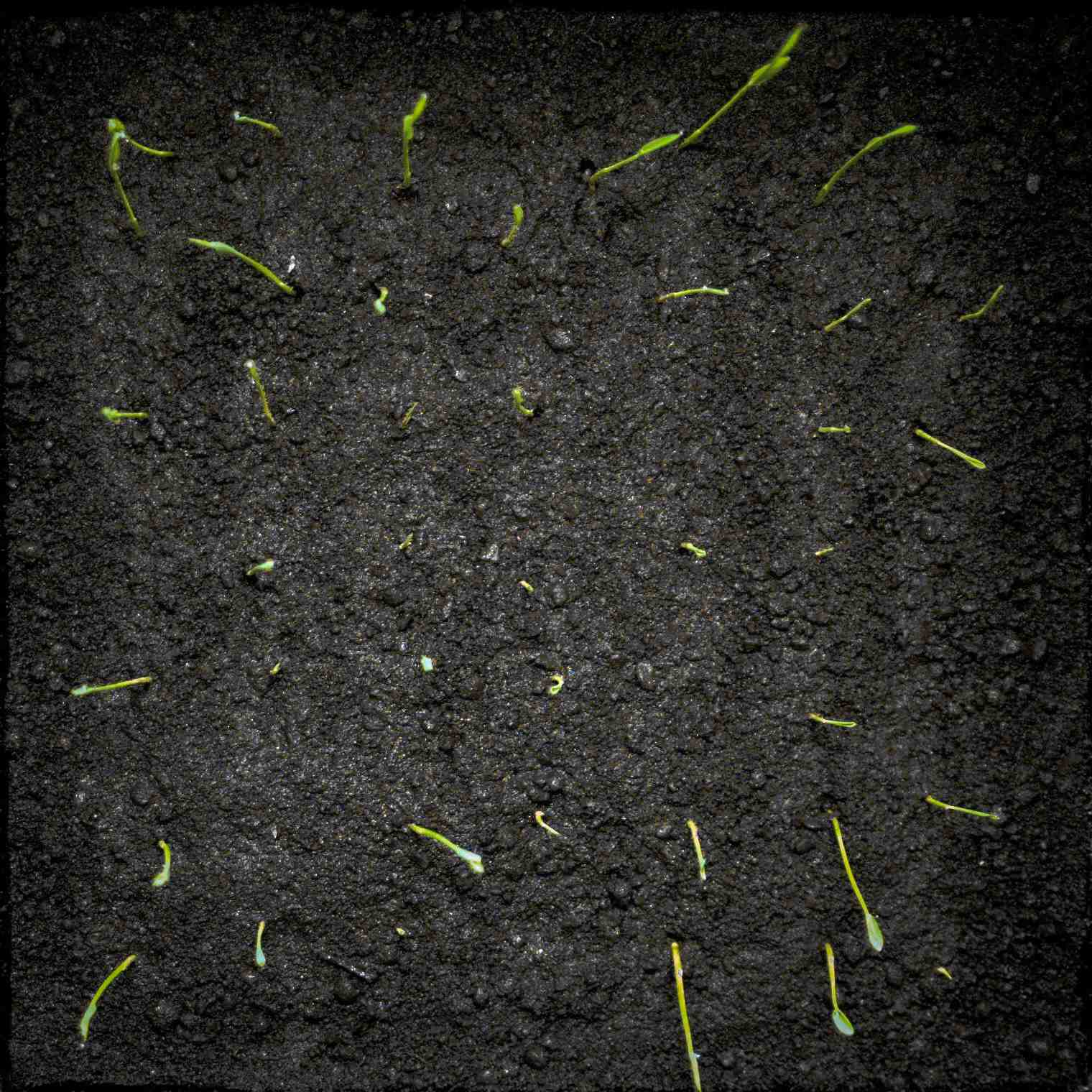

Supplement: Supplementary file 3 [file DataSheet3.zip › train1/200-2024-3-20-10-45-13.JPG]

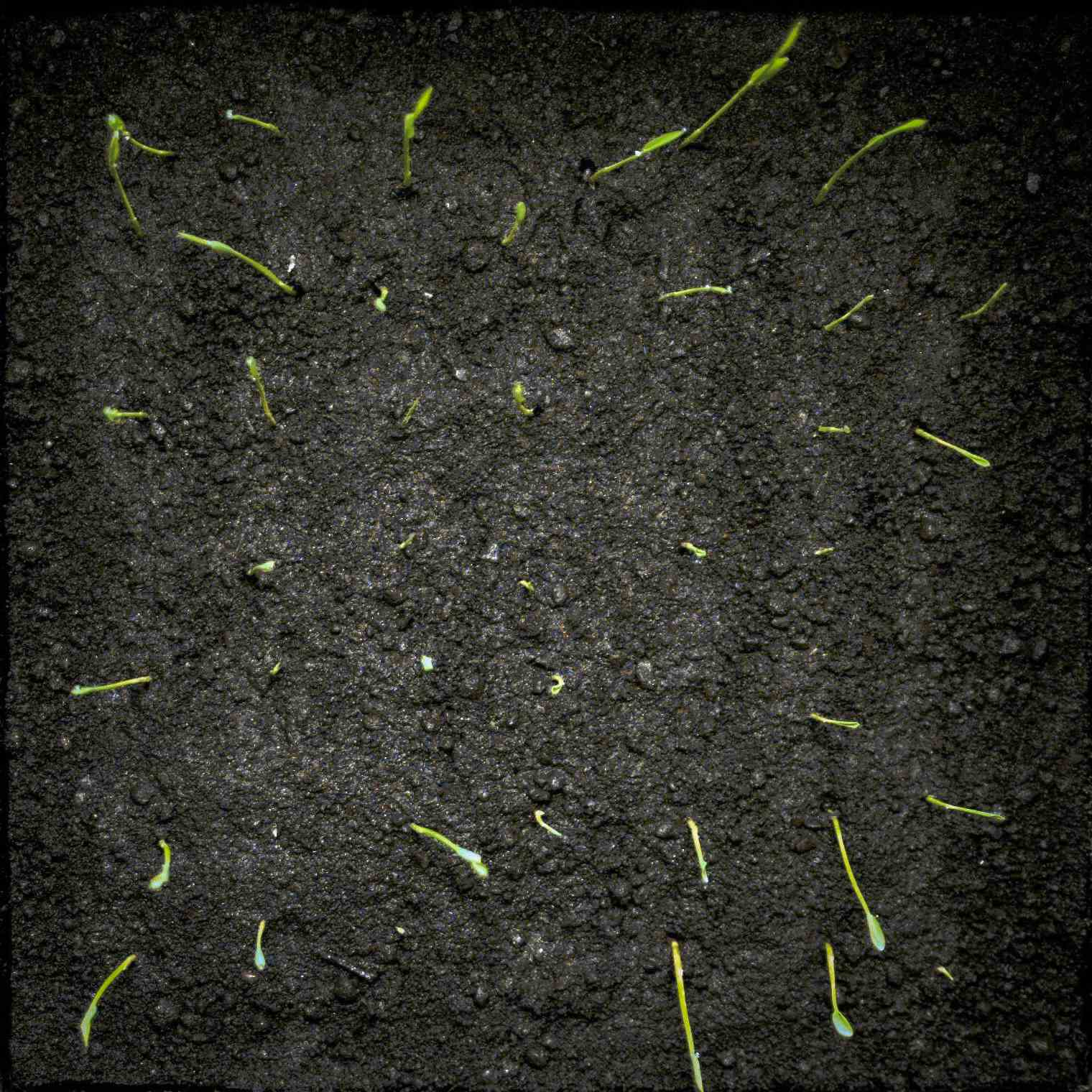

Supplement: Supplementary file 3 [file DataSheet3.zip › train1/200-2024-3-20-13-36-13.JPG]

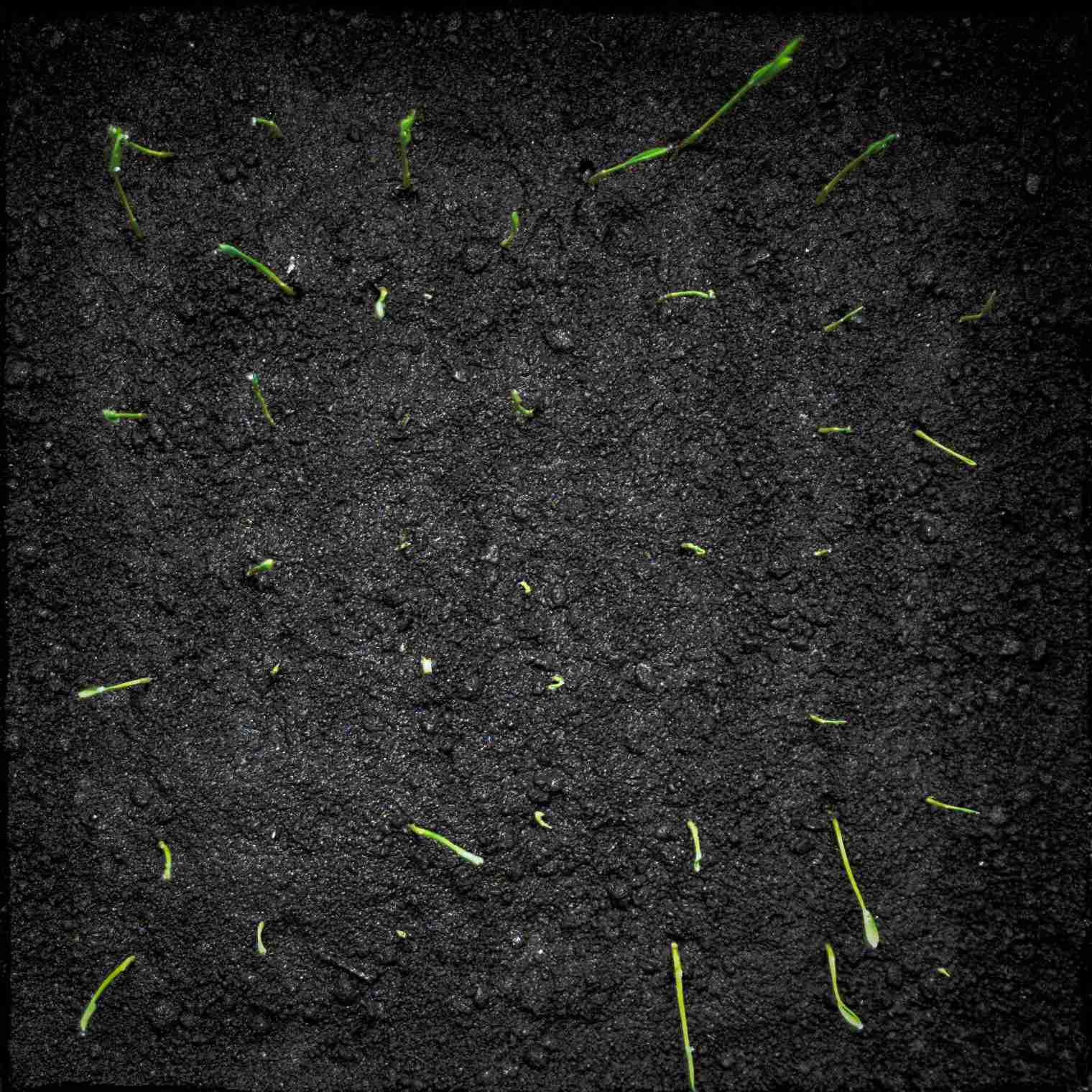

Supplement: Supplementary file 3 [file DataSheet3.zip › train1/200-2024-3-20-2-12-5.JPG]

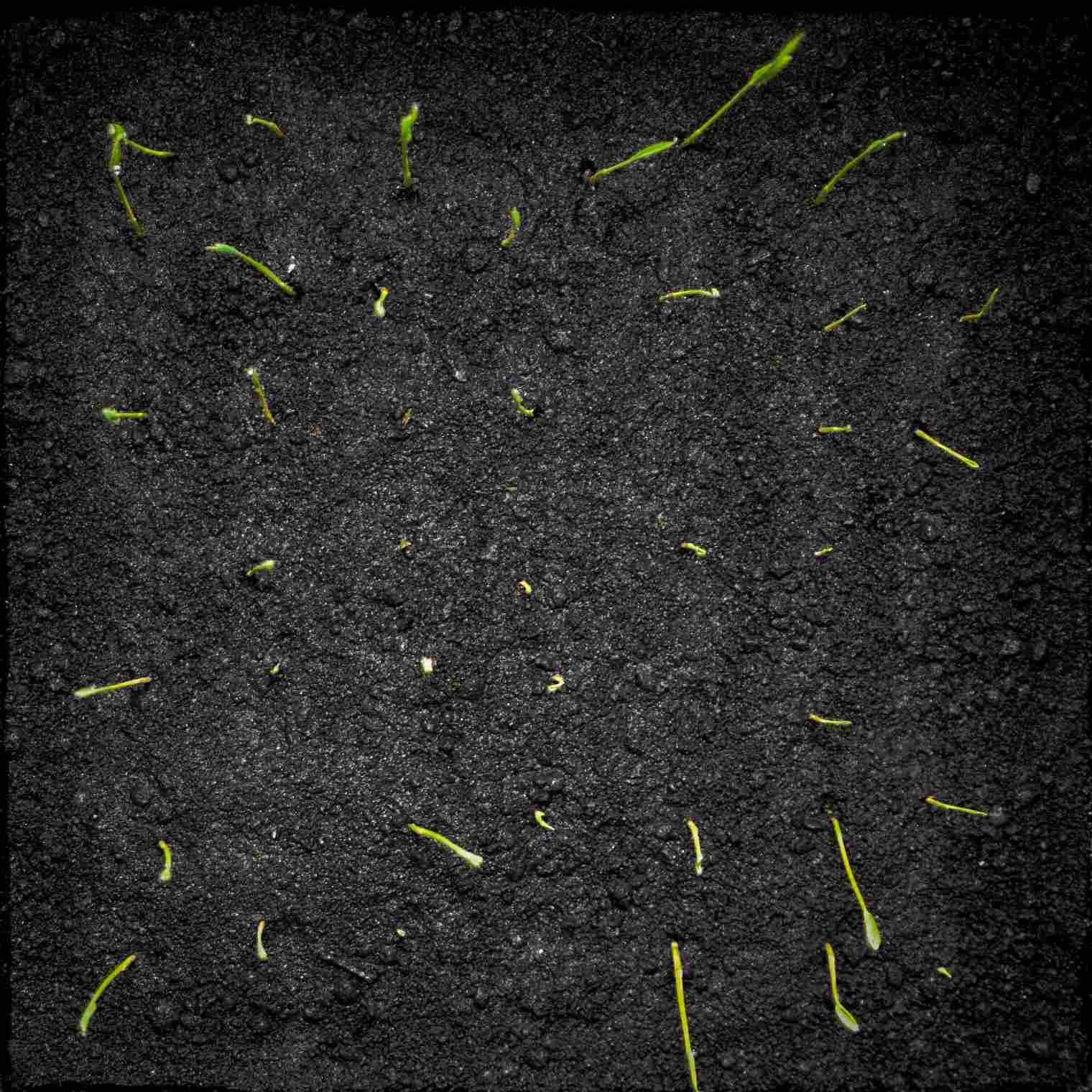

Supplement: Supplementary file 3 [file DataSheet3.zip › train1/200-2024-3-20-5-3-18.JPG]

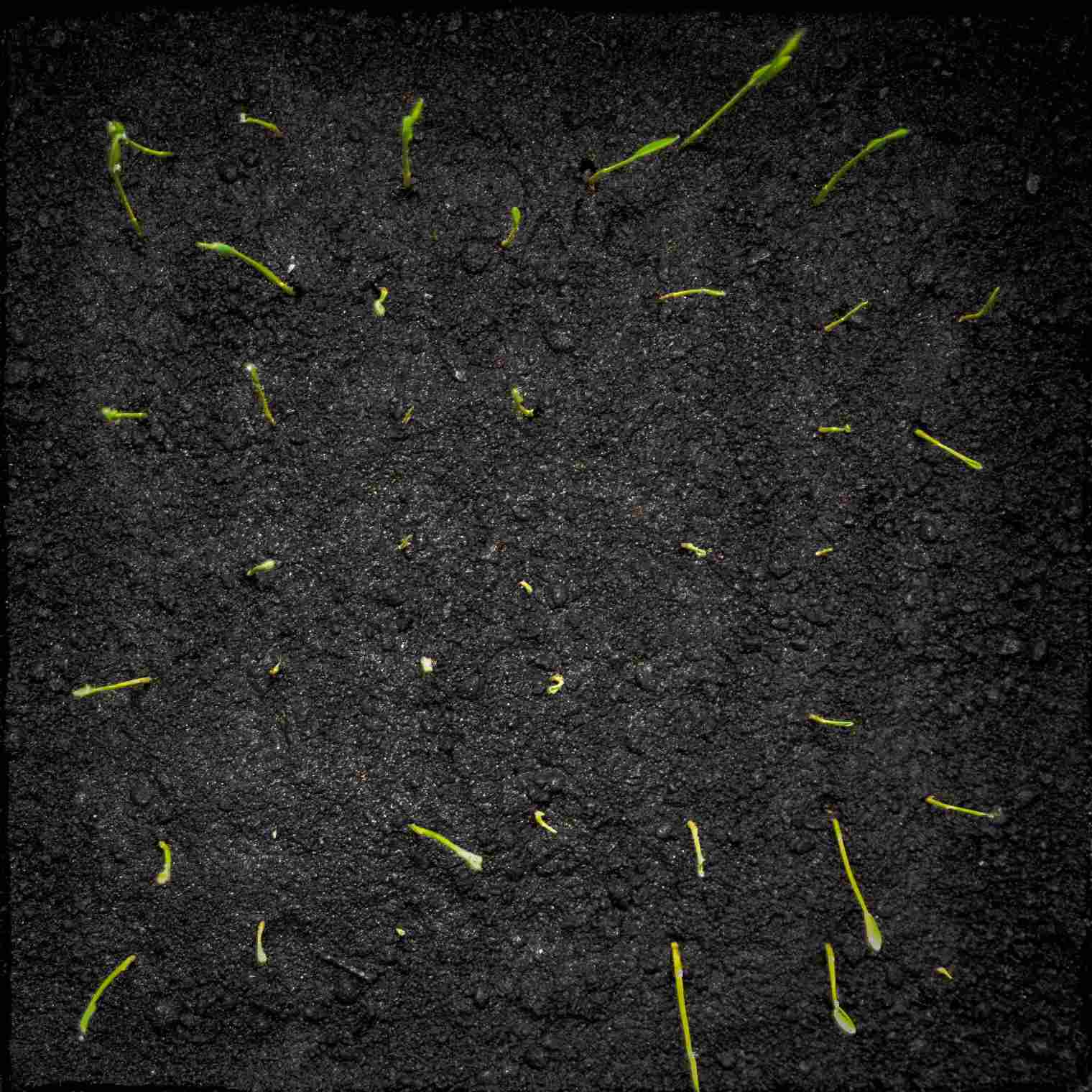

Supplement: Supplementary file 3 [file DataSheet3.zip › train1/200-2024-3-20-7-53-58.JPG]

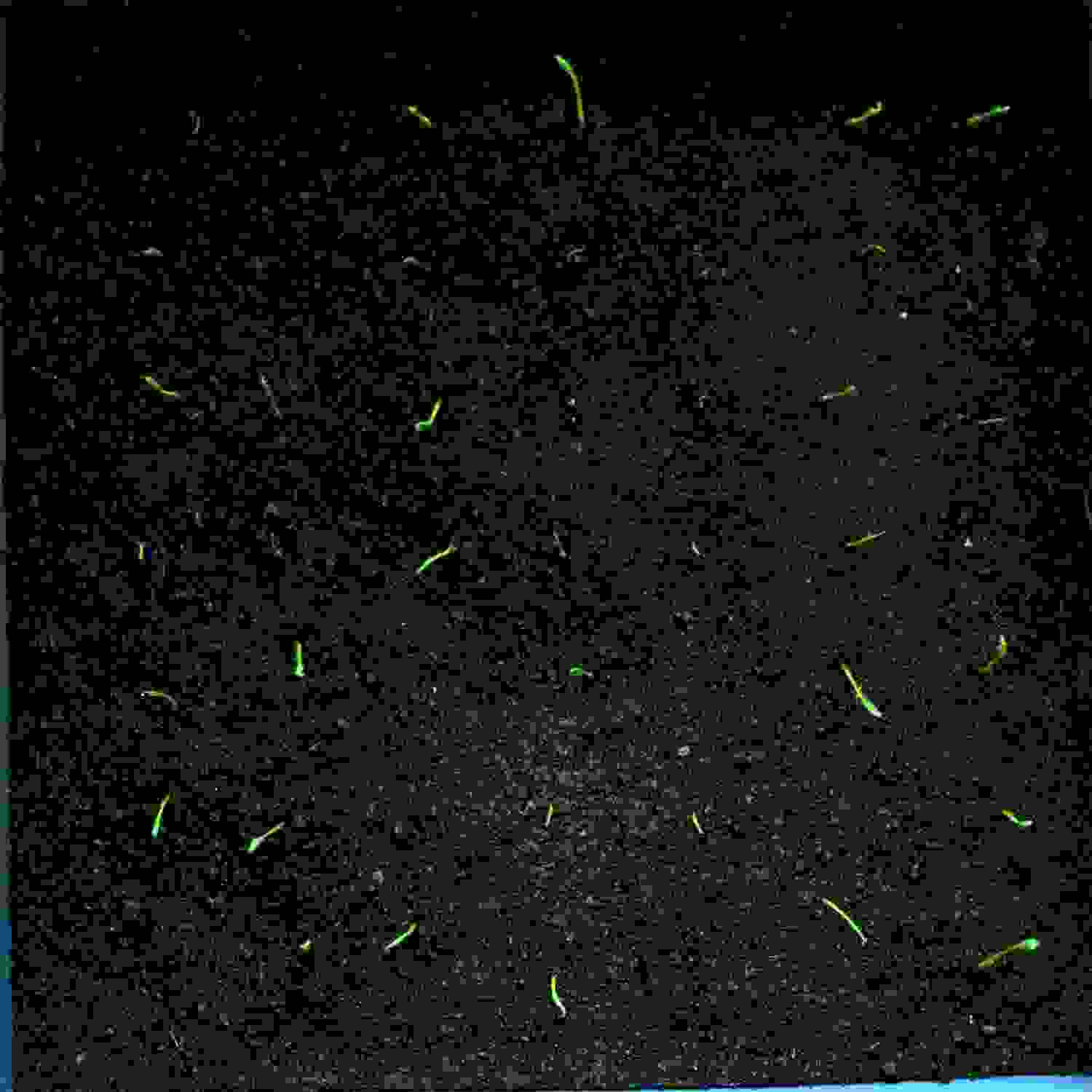

Supplement: Supplementary file 3 [file DataSheet3.zip › train1/2000-2024-3-18-20-54-48.JPG]

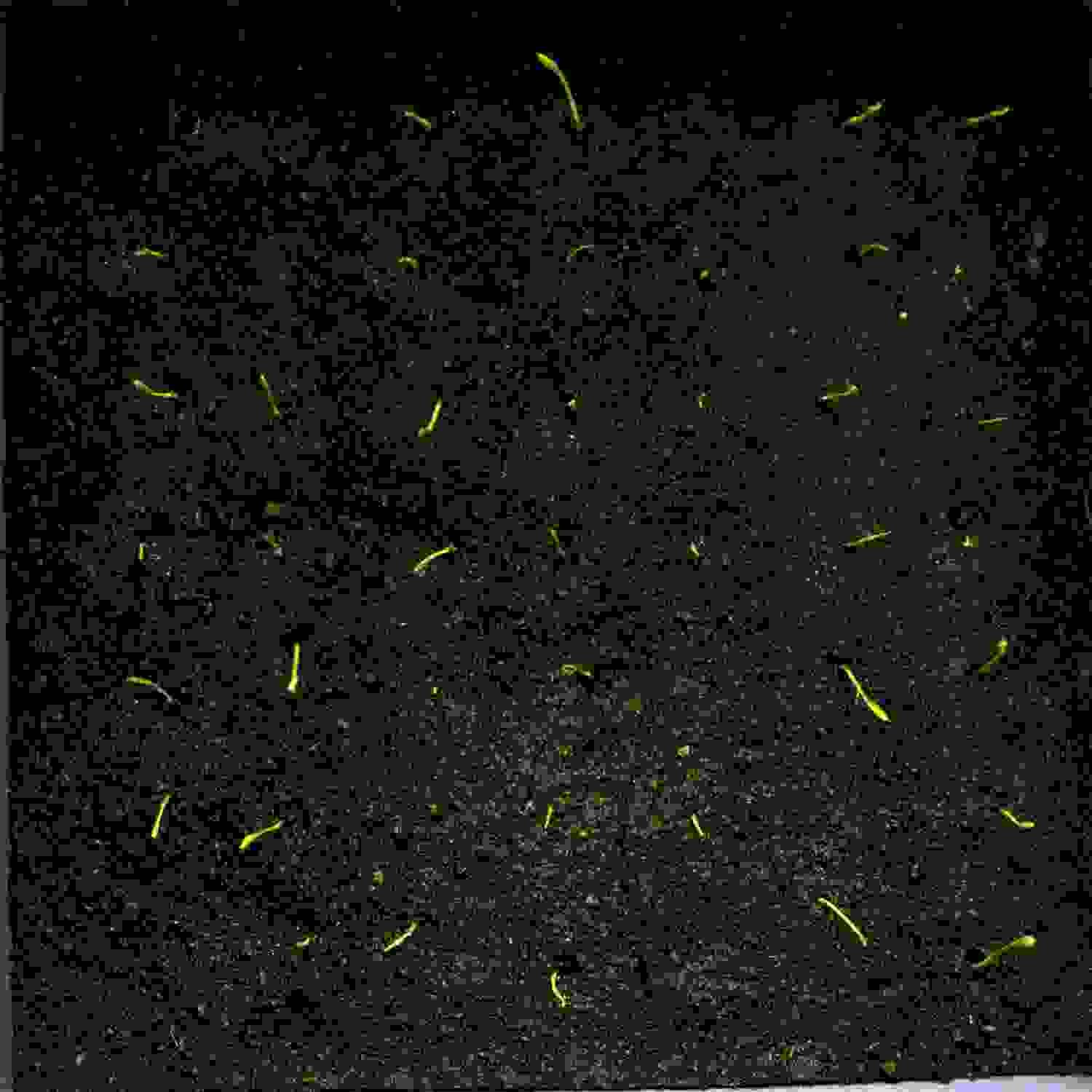

Supplement: Supplementary file 3 [file DataSheet3.zip › train1/2000-2024-3-18-23-27-23.JPG]

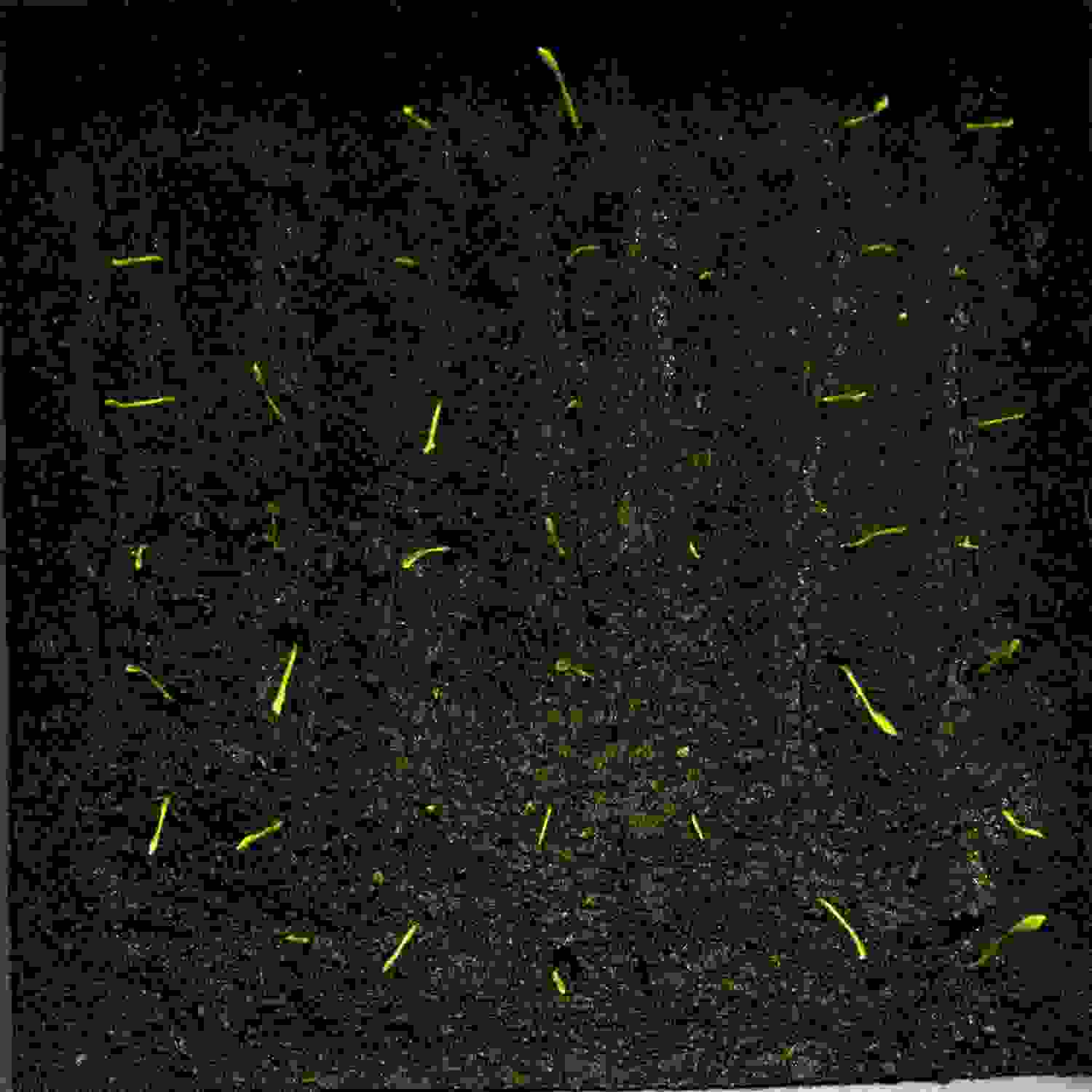

Supplement: Supplementary file 3 [file DataSheet3.zip › train1/2000-2024-3-19-12-7-33.JPG]

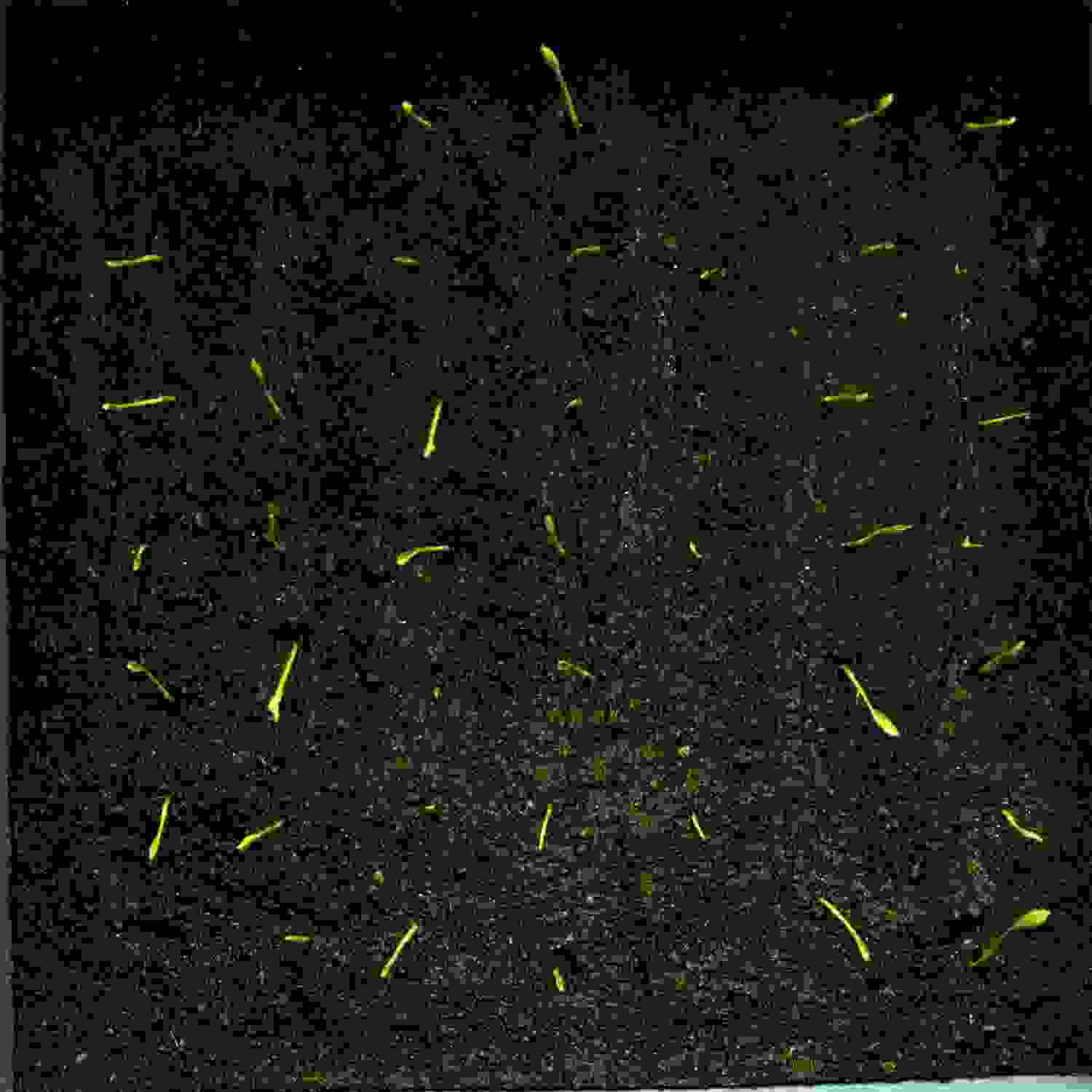

Supplement: Supplementary file 3 [file DataSheet3.zip › train1/2000-2024-3-19-17-12-39.JPG]

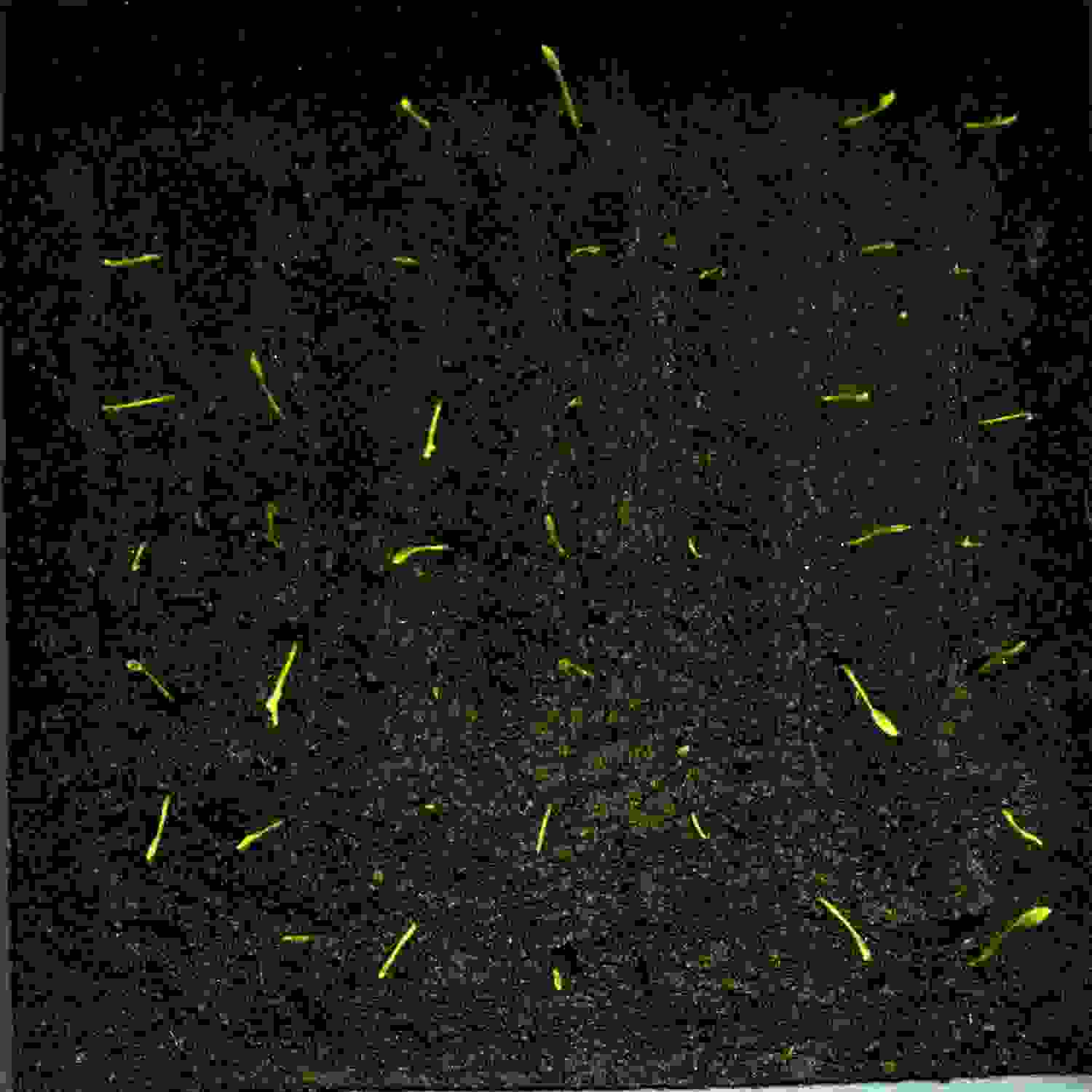

Supplement: Supplementary file 3 [file DataSheet3.zip › train1/2000-2024-3-19-19-44-52.JPG]

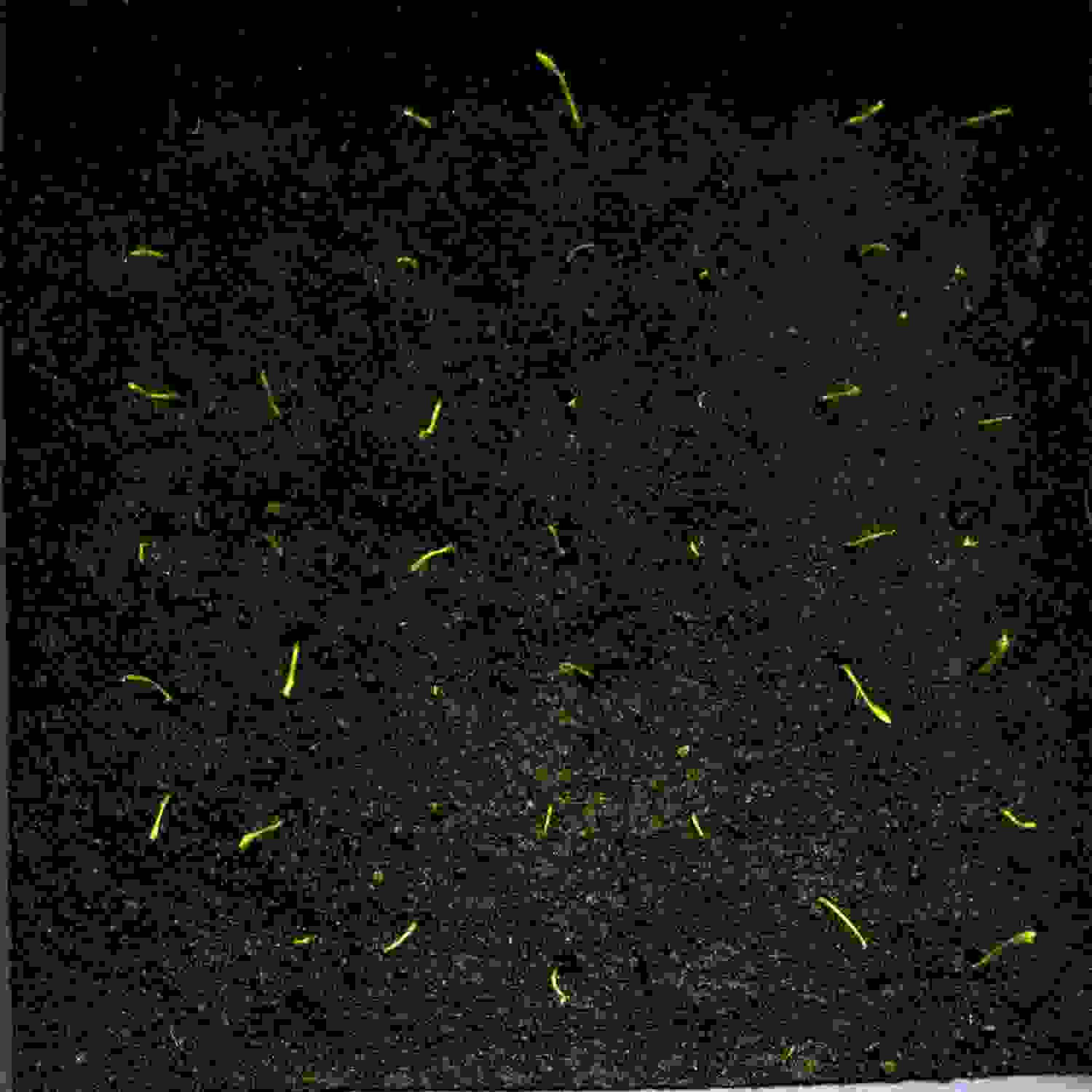

Supplement: Supplementary file 3 [file DataSheet3.zip › train1/2000-2024-3-19-2-0-5.JPG]

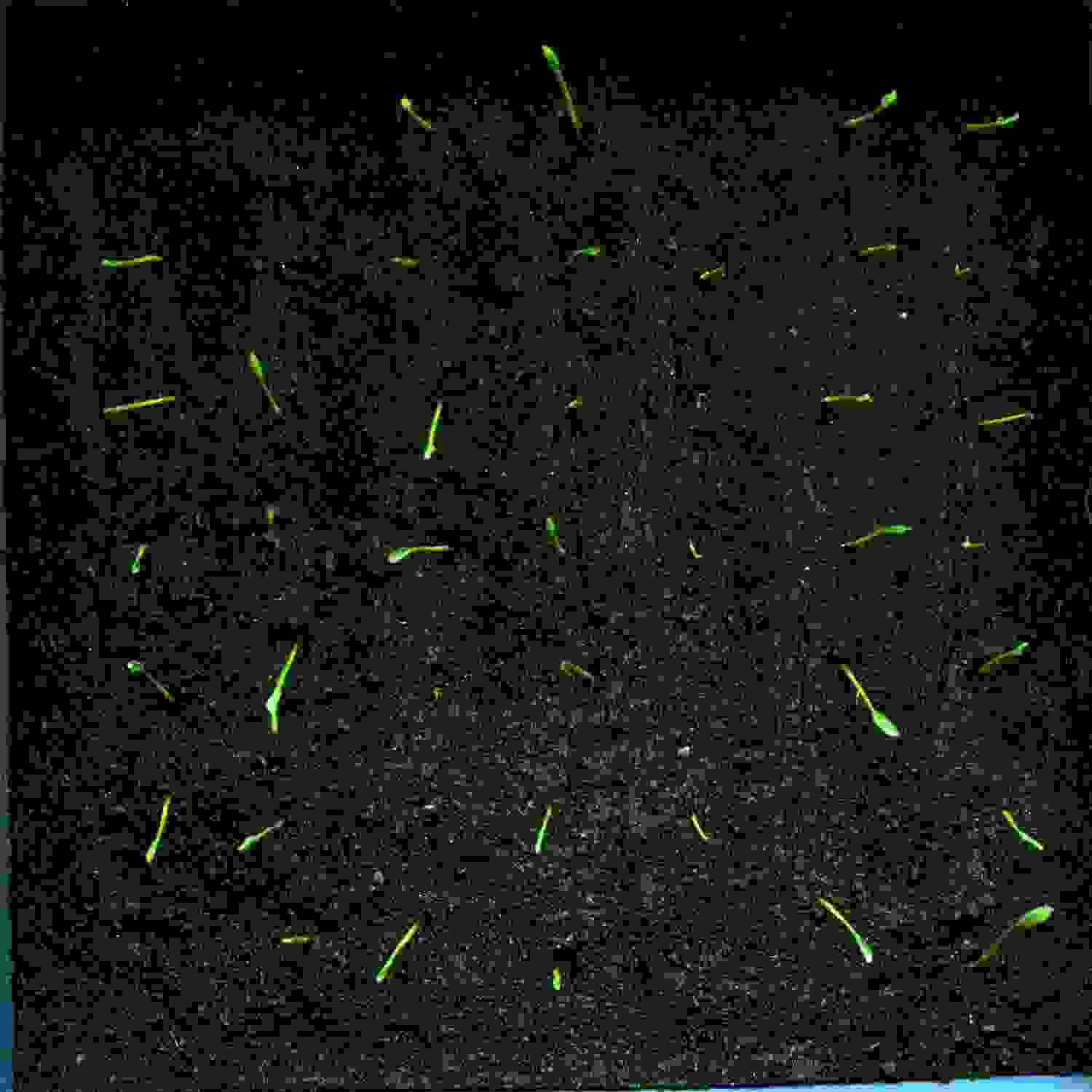

Supplement: Supplementary file 3 [file DataSheet3.zip › train1/2000-2024-3-19-22-16-36.JPG]

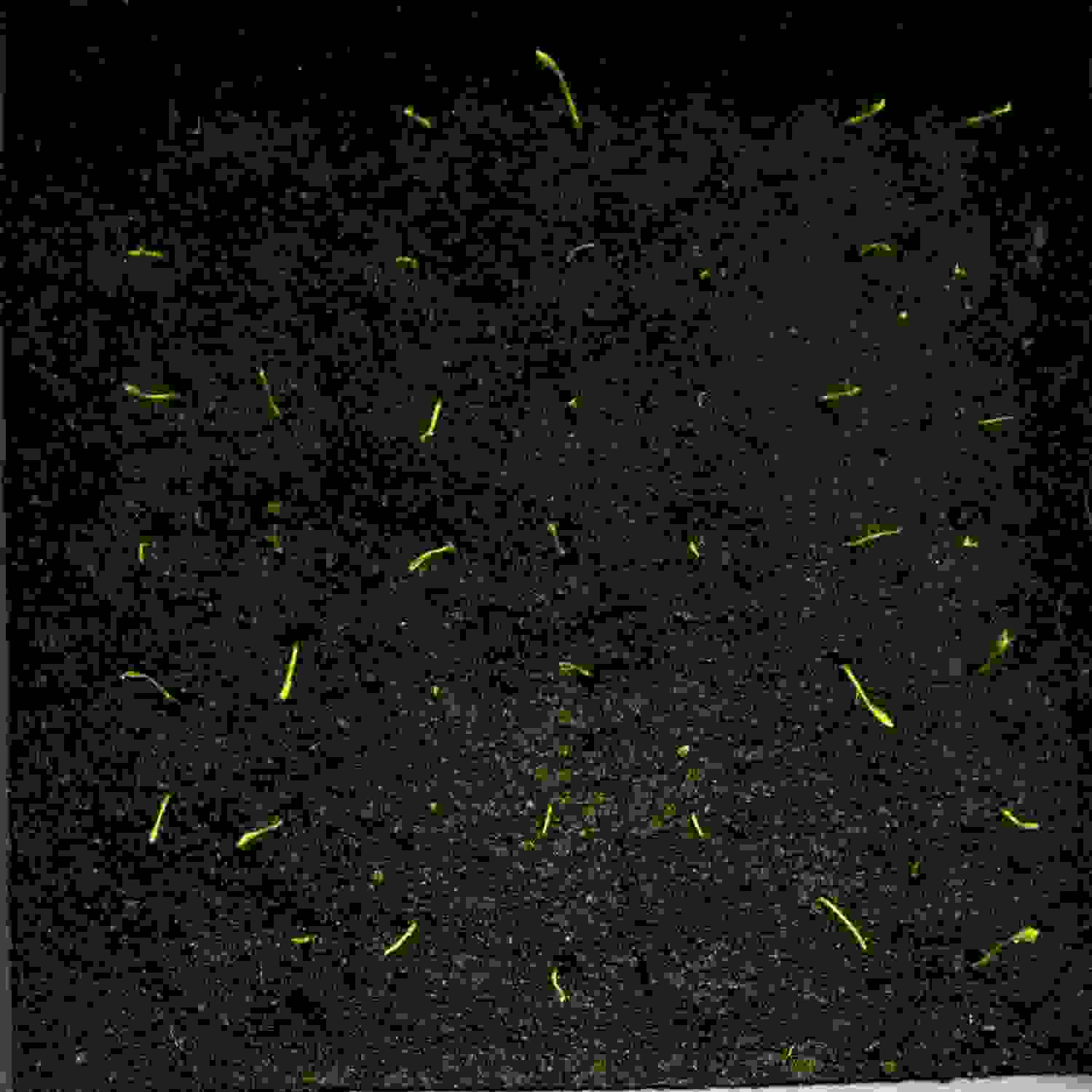

Supplement: Supplementary file 3 [file DataSheet3.zip › train1/2000-2024-3-19-4-31-48.JPG]

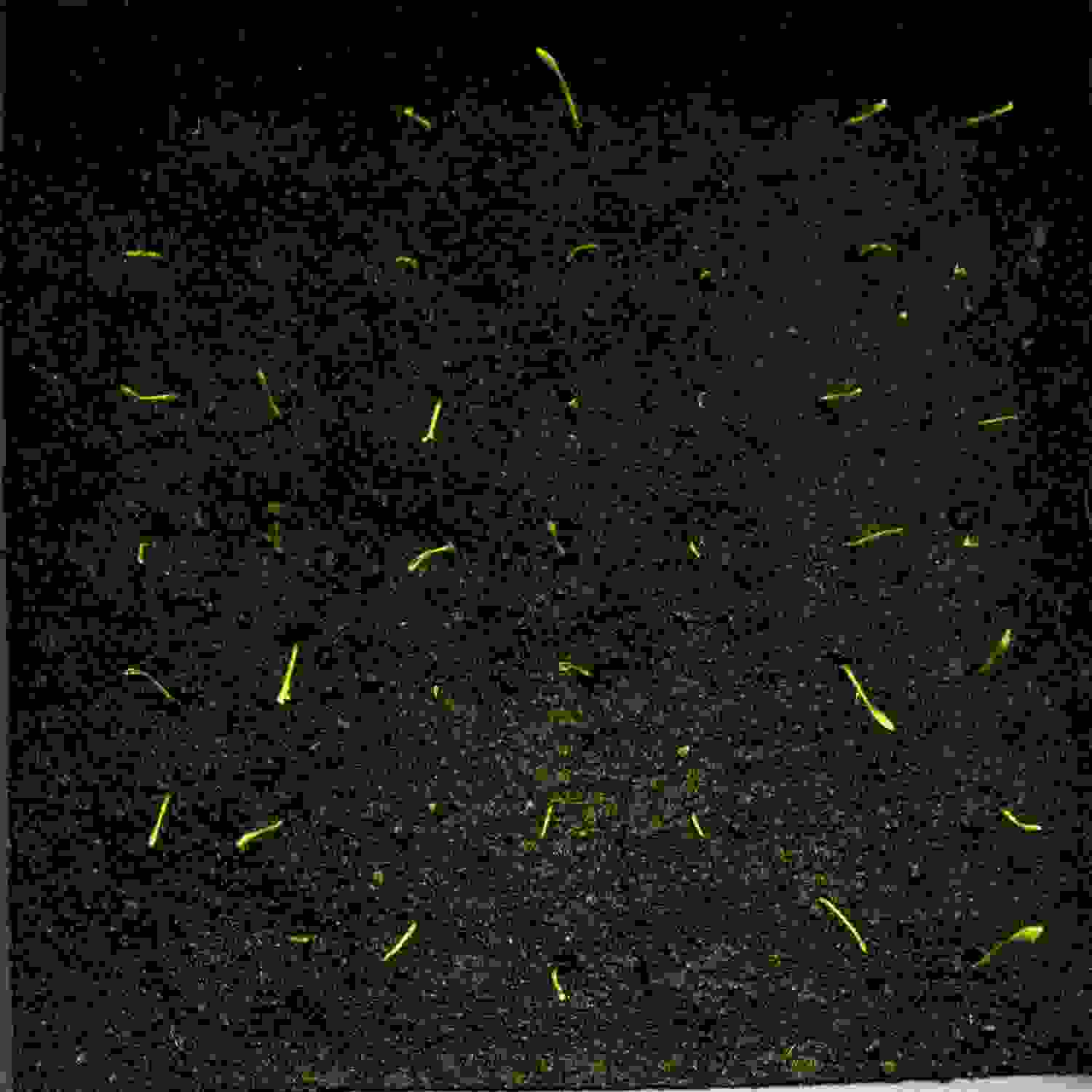

Supplement: Supplementary file 3 [file DataSheet3.zip › train1/2000-2024-3-19-7-3-44.JPG]

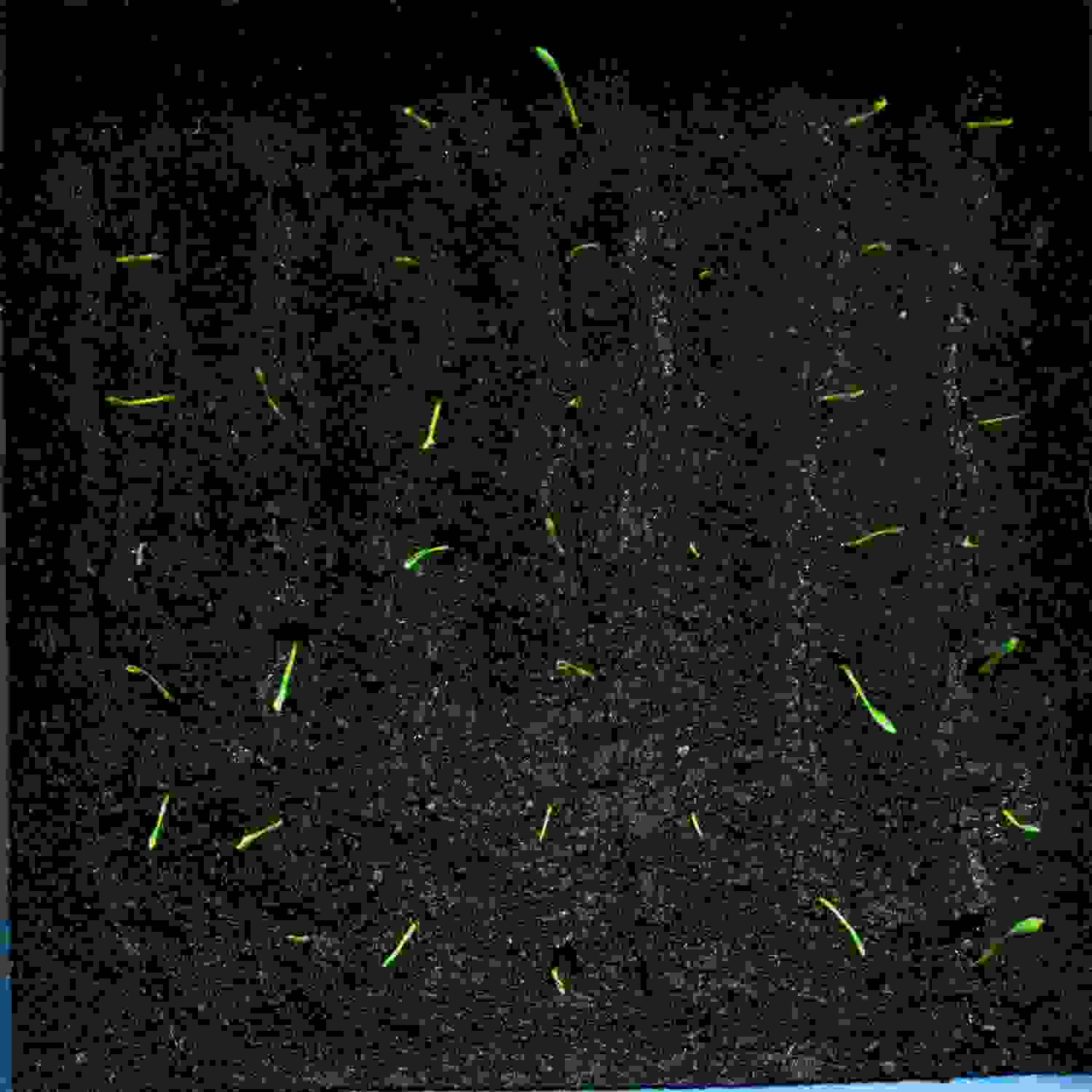

Supplement: Supplementary file 3 [file DataSheet3.zip › train1/2000-2024-3-19-9-35-50.JPG]

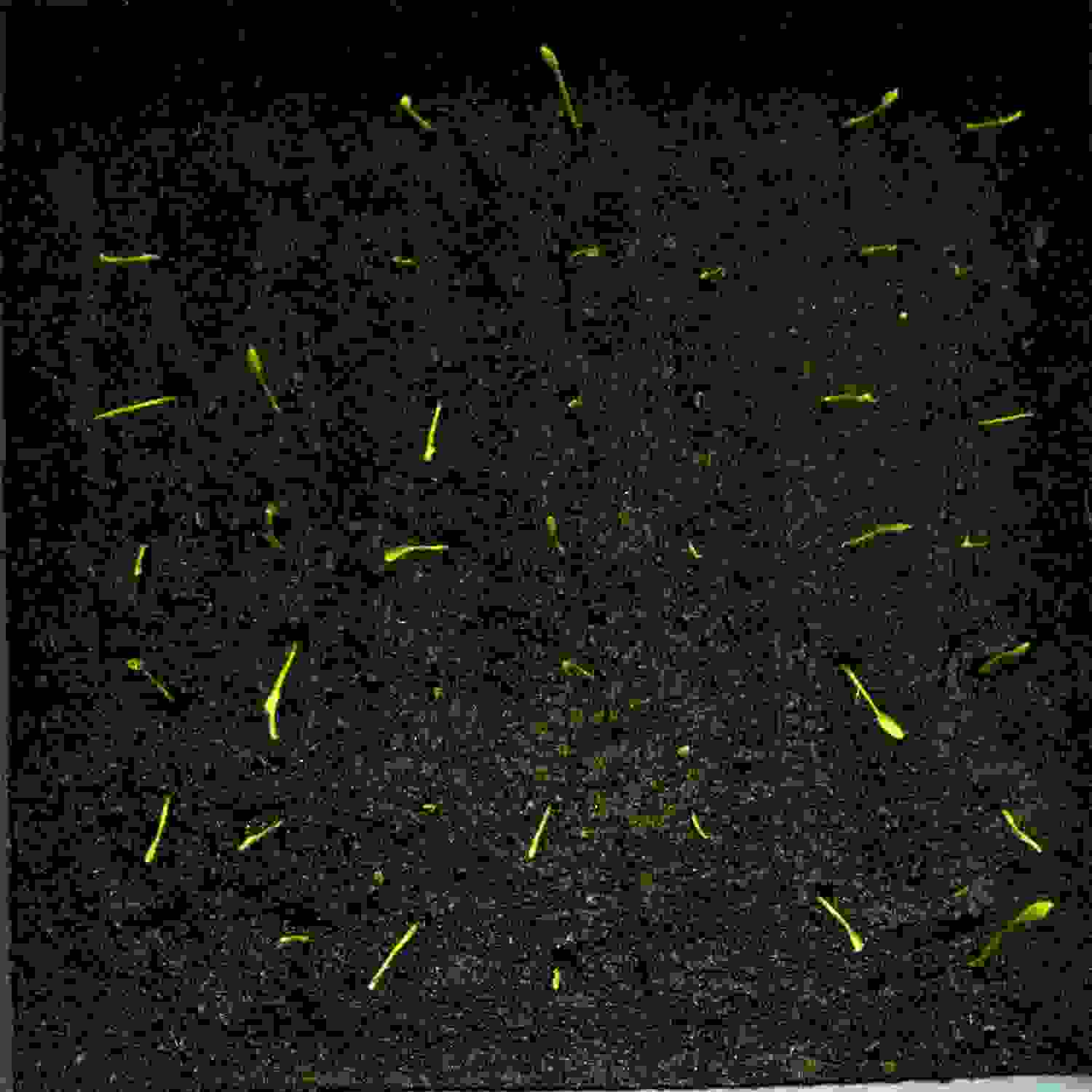

Supplement: Supplementary file 3 [file DataSheet3.zip › train1/2000-2024-3-20-0-48-45.JPG]

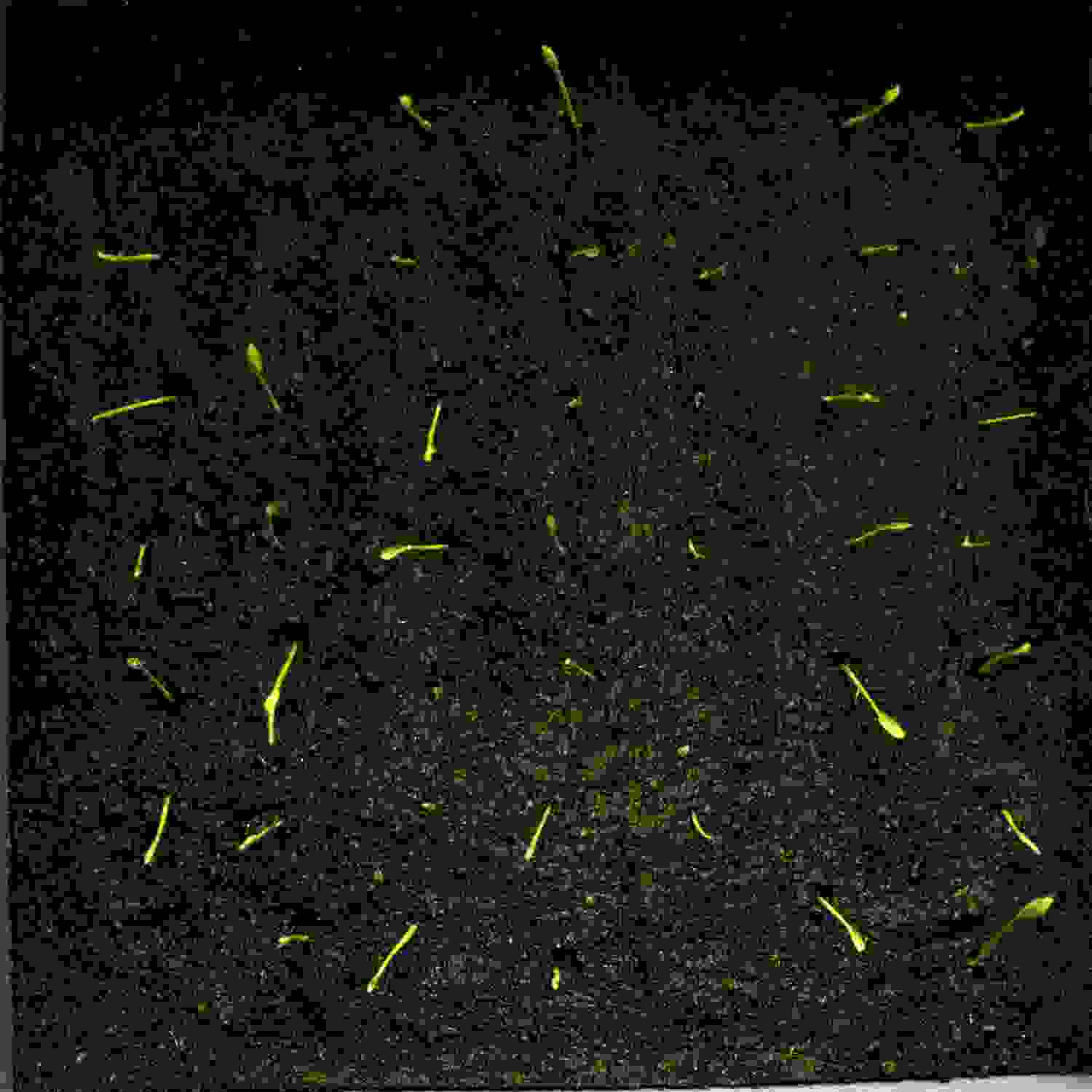

Supplement: Supplementary file 3 [file DataSheet3.zip › train1/2000-2024-3-20-3-20-44.JPG]

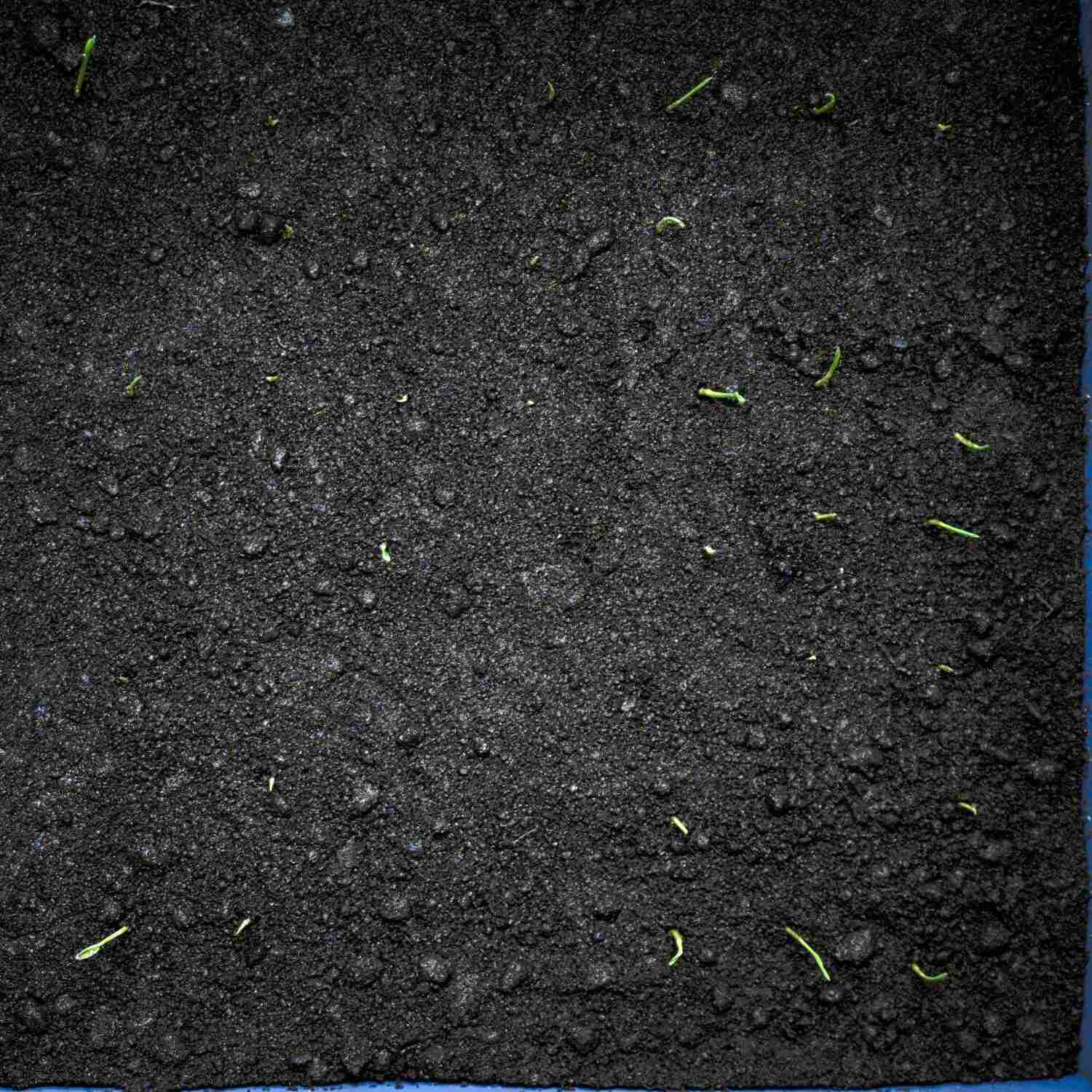

Supplement: Supplementary file 3 [file DataSheet3.zip › train1/2030-2024-3-18-21-37-2.JPG]

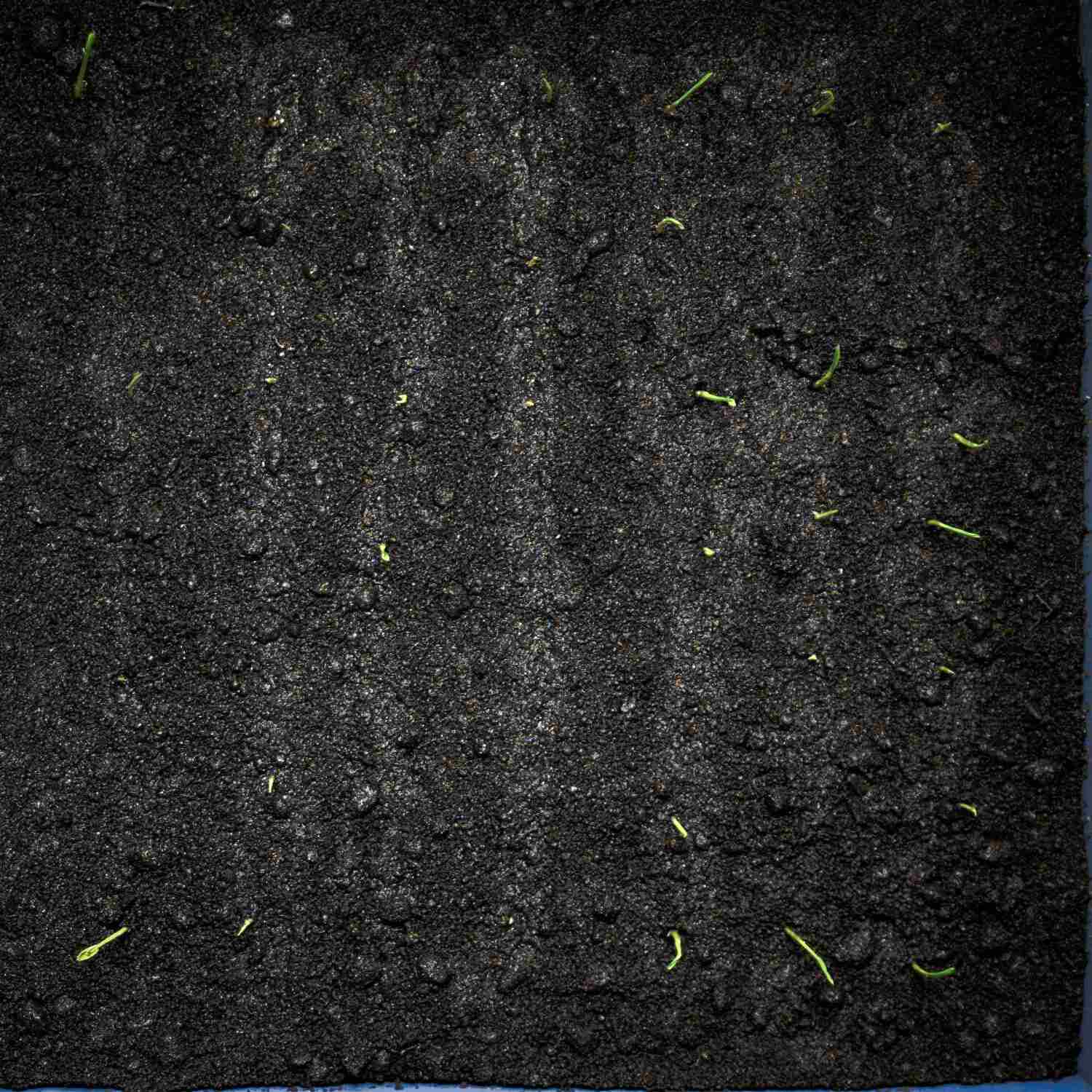

Supplement: Supplementary file 3 [file DataSheet3.zip › train1/2030-2024-3-19-0-29-33.JPG]

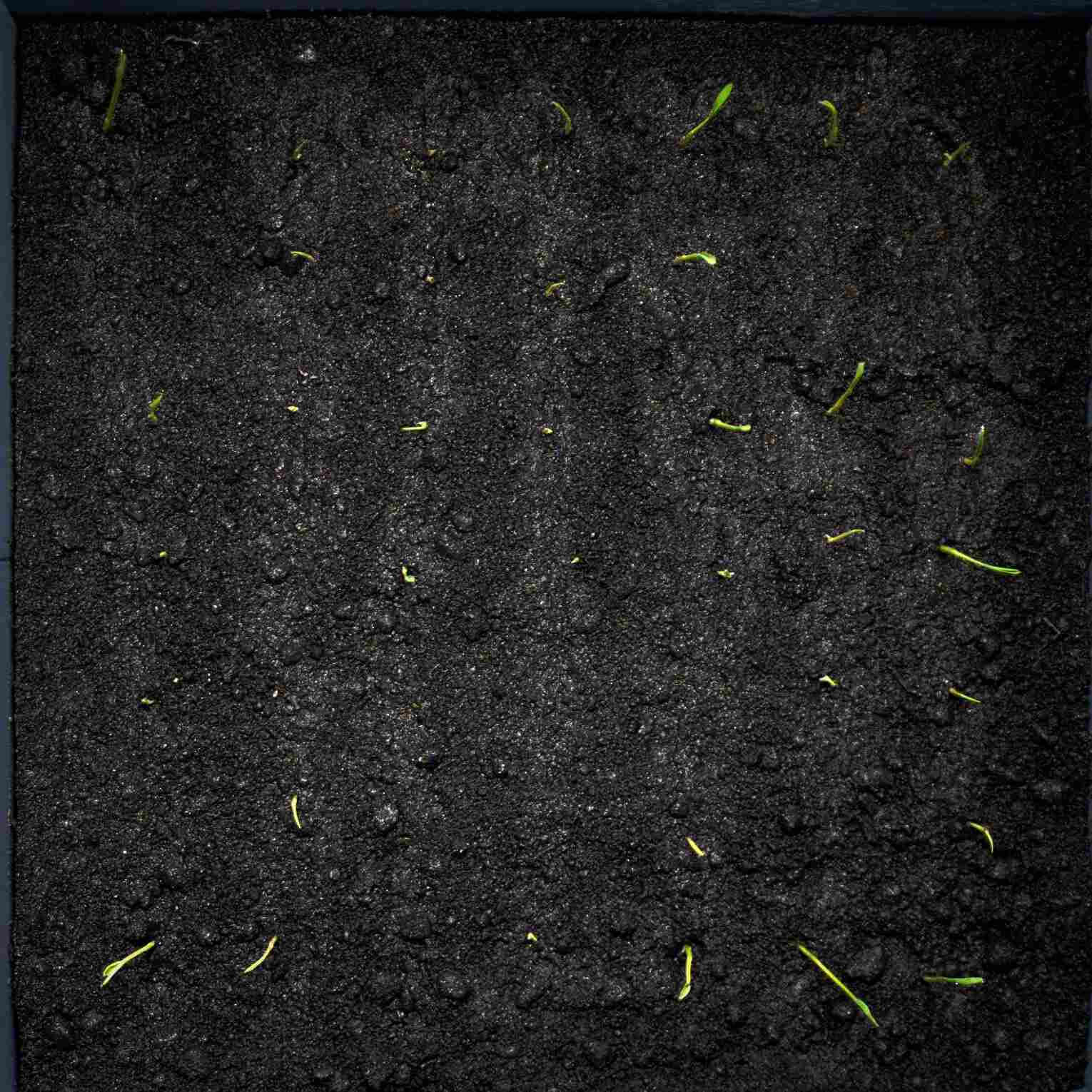

Supplement: Supplementary file 3 [file DataSheet3.zip › train1/2030-2024-3-19-20-29-24.JPG]

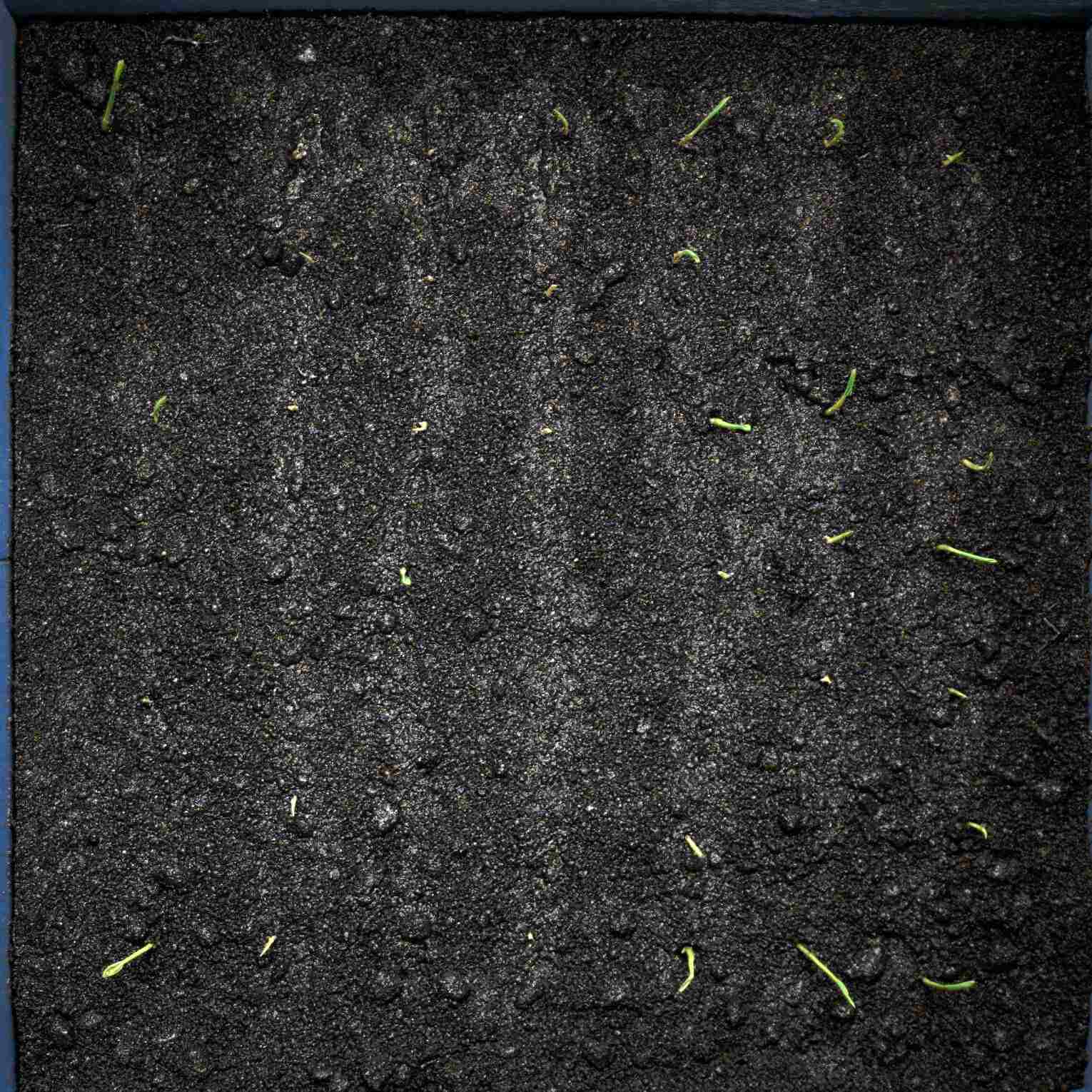

Supplement: Supplementary file 3 [file DataSheet3.zip › train1/2030-2024-3-19-6-13-8.JPG]

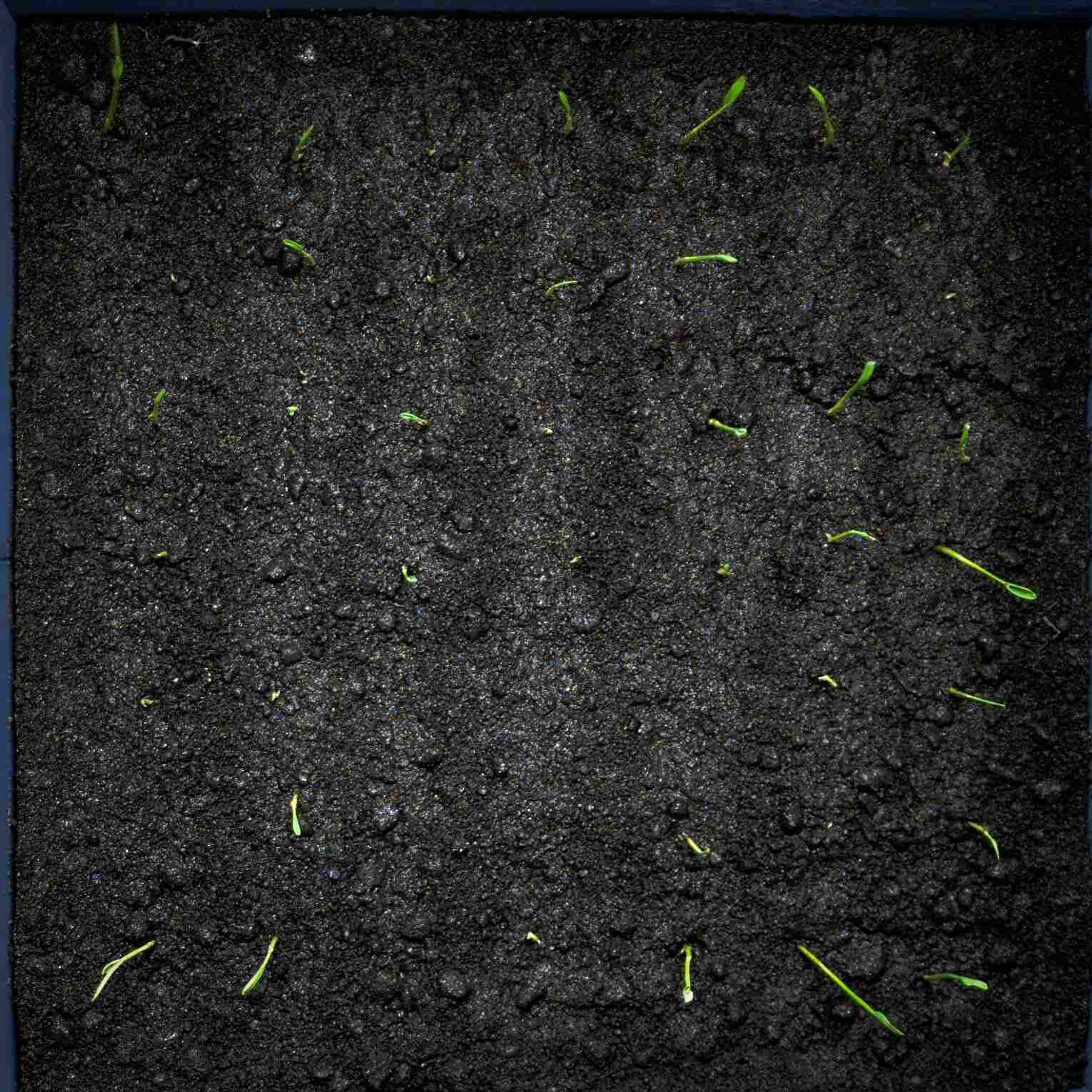

Supplement: Supplementary file 3 [file DataSheet3.zip › train1/2030-2024-3-20-10-45-43.JPG]

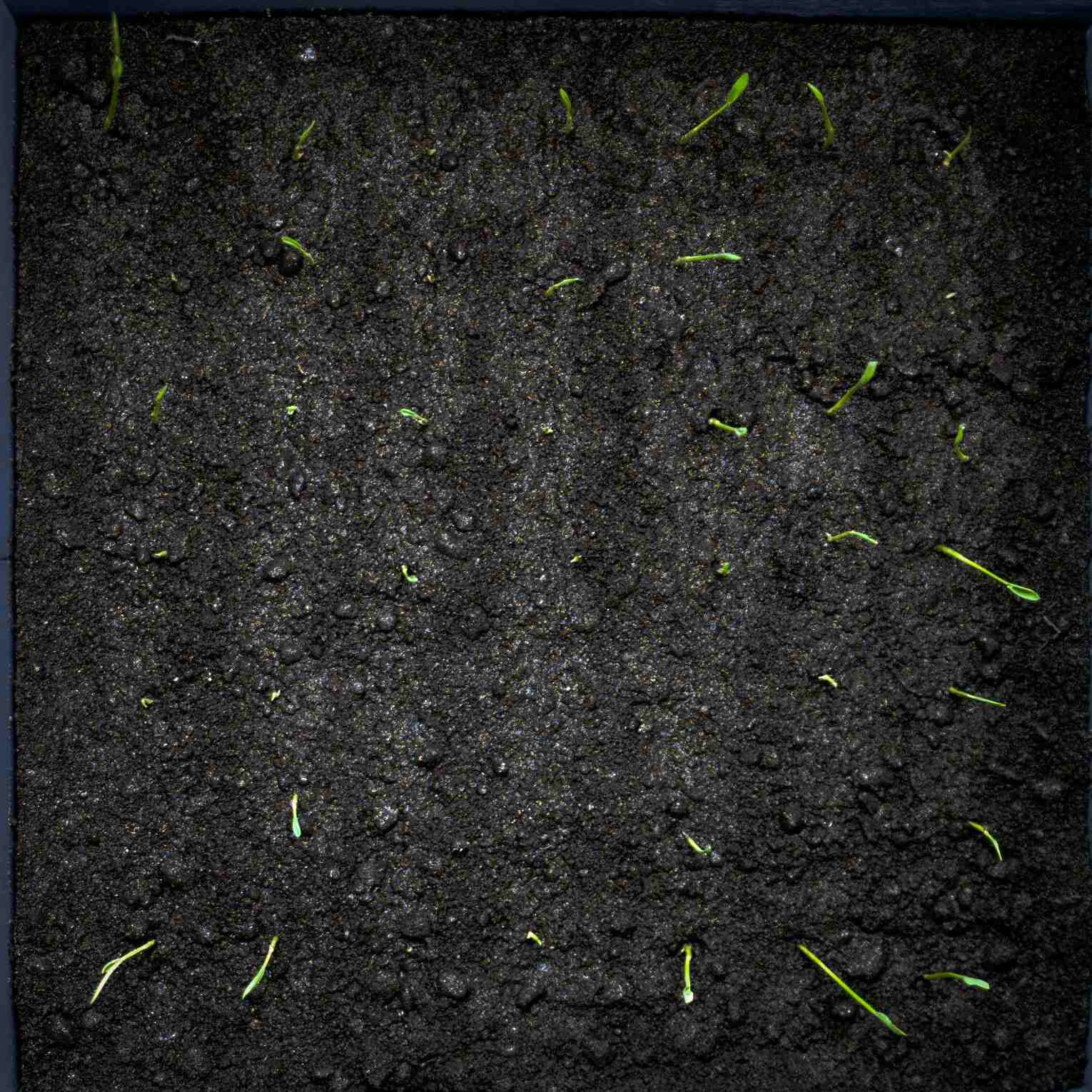

Supplement: Supplementary file 3 [file DataSheet3.zip › train1/2030-2024-3-20-13-36-43.JPG]

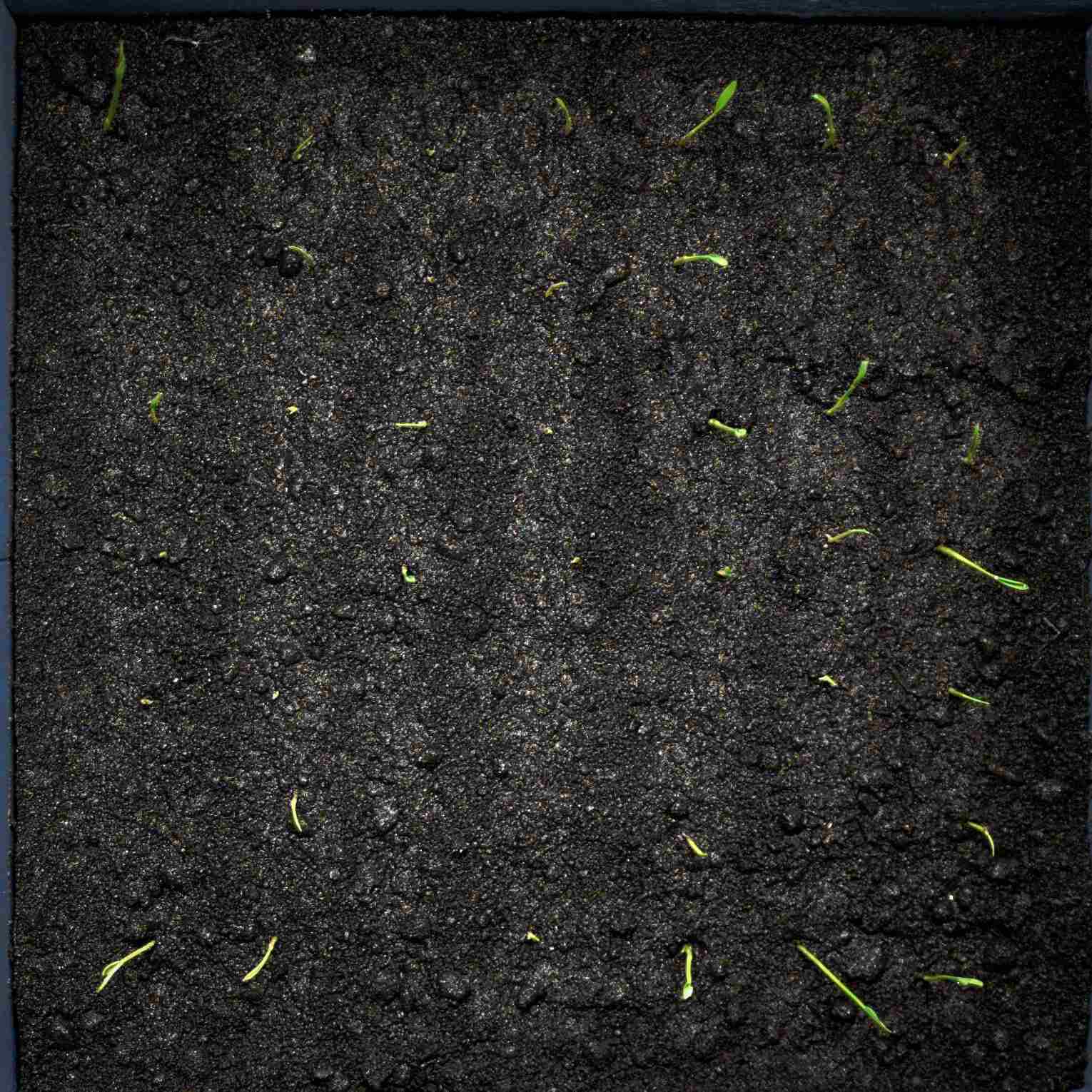

Supplement: Supplementary file 3 [file DataSheet3.zip › train1/2030-2024-3-20-2-12-35.JPG]

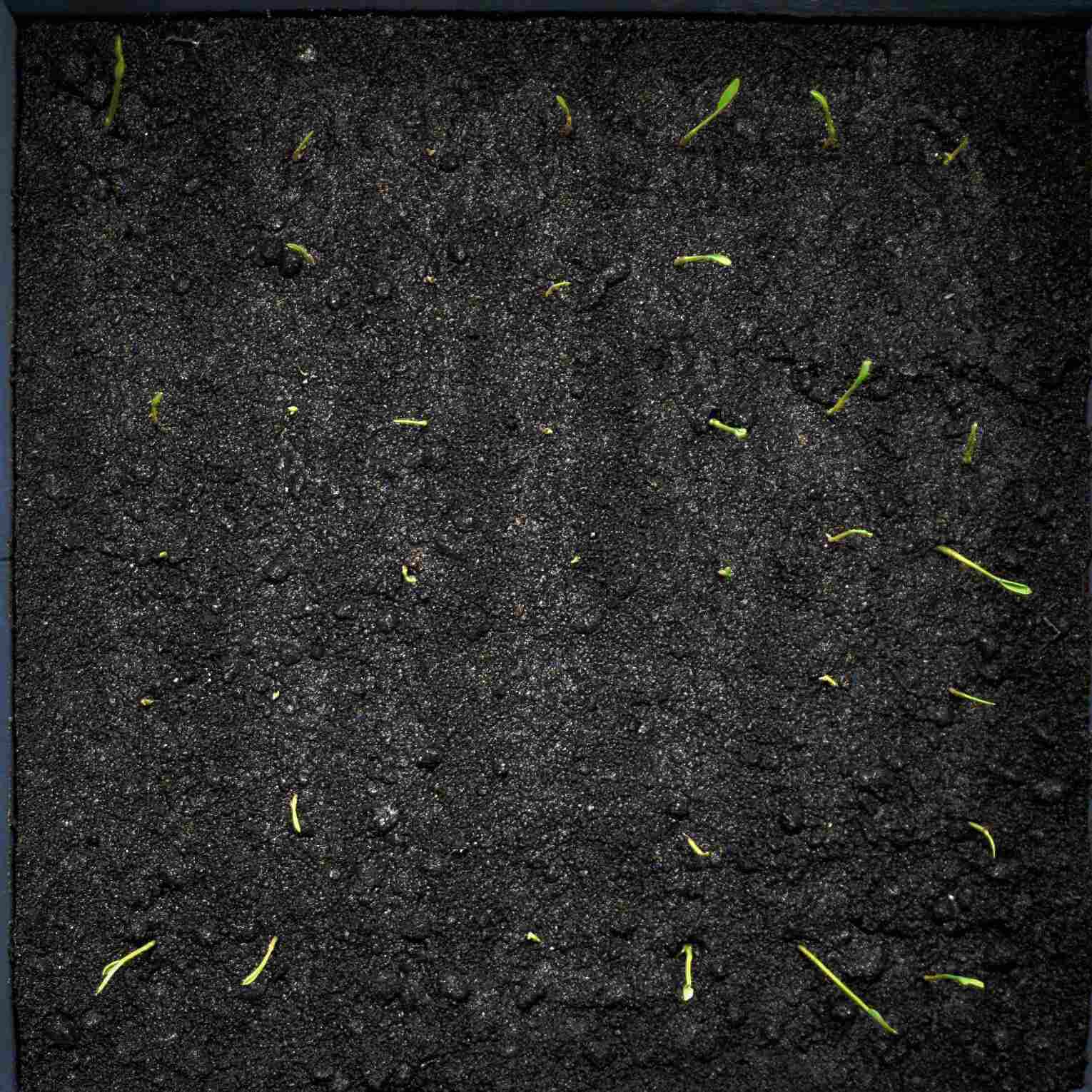

Supplement: Supplementary file 3 [file DataSheet3.zip › train1/2030-2024-3-20-5-3-48.JPG]

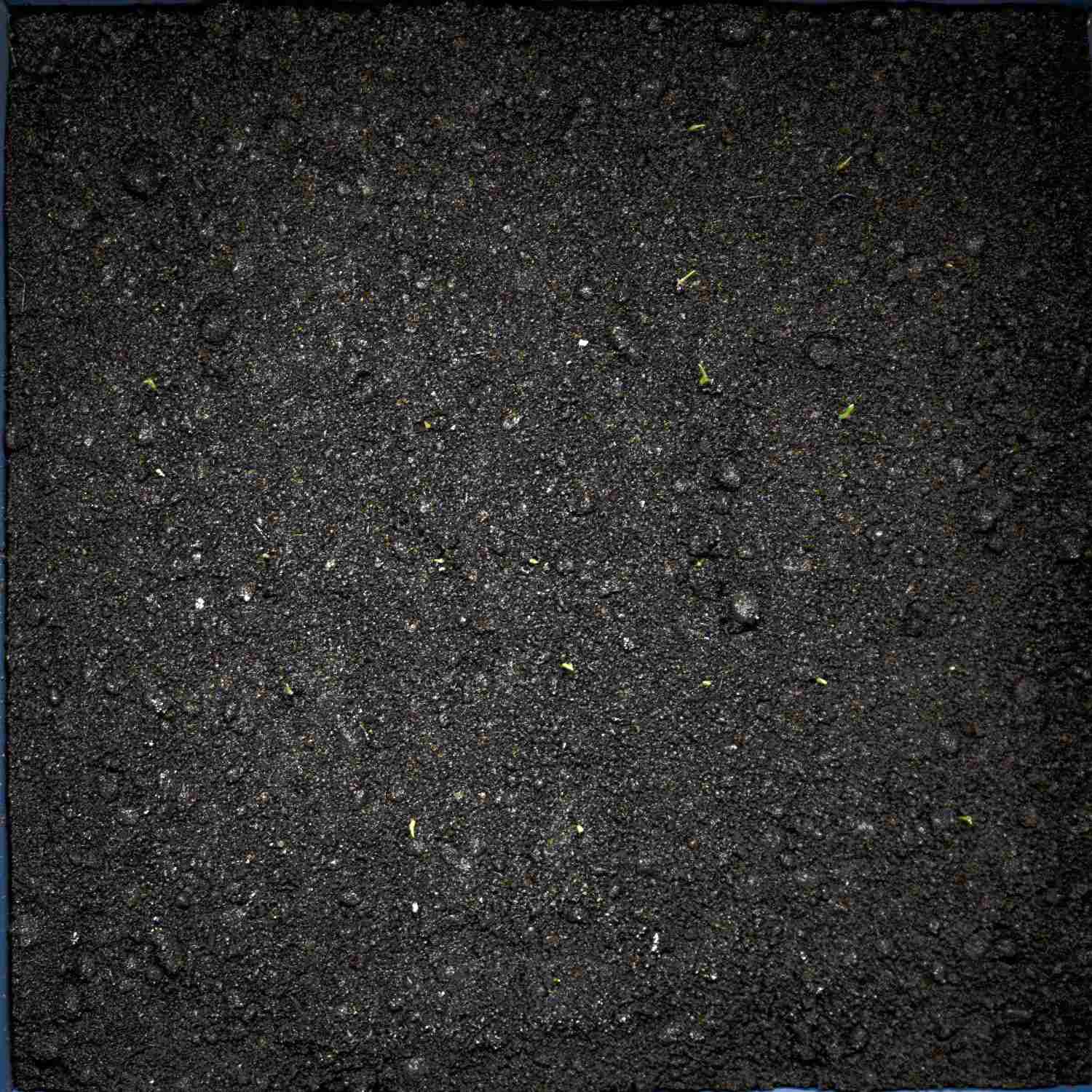

Supplement: Supplementary file 3 [file DataSheet3.zip › train1/2060-2024-3-18-17-46-12.JPG]

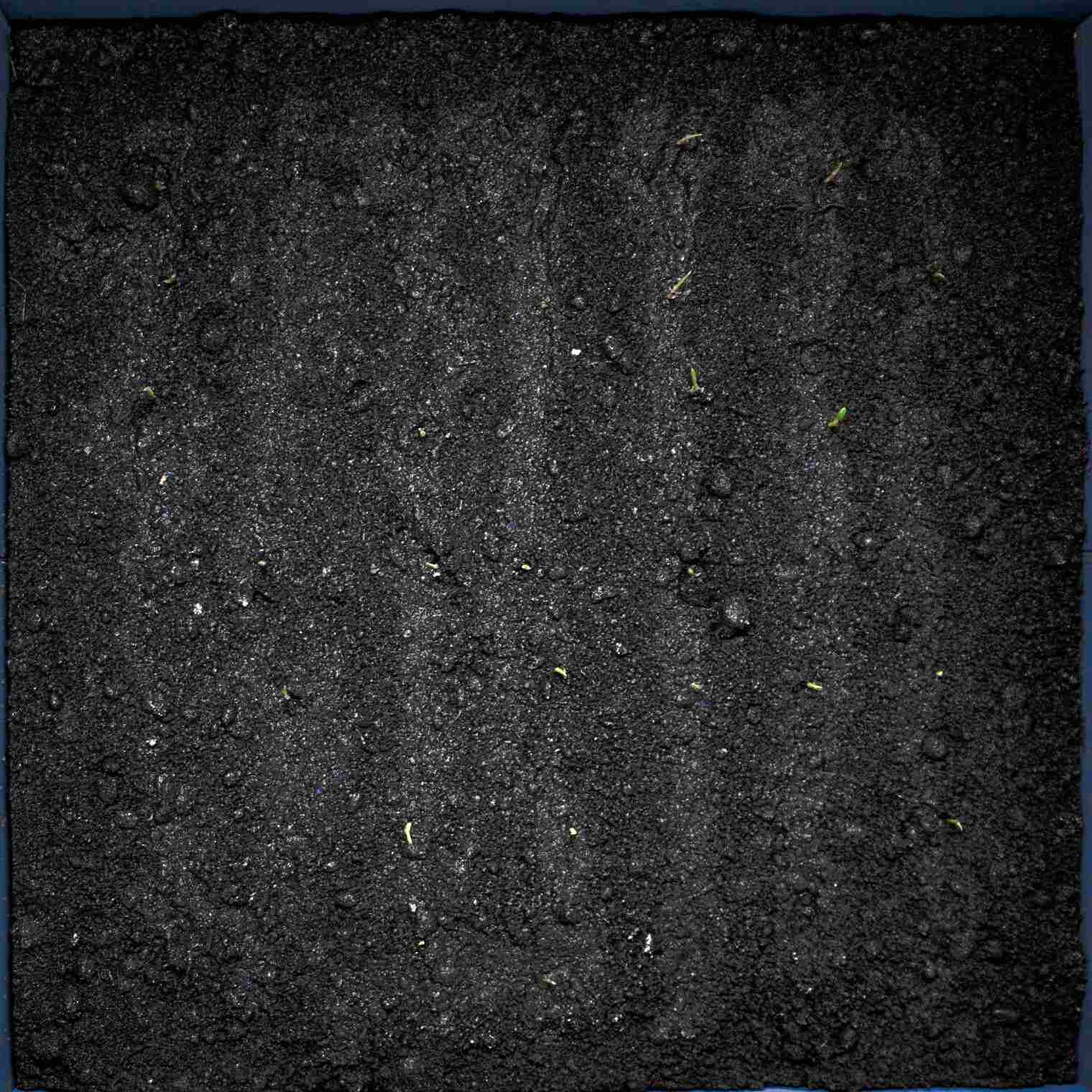

Supplement: Supplementary file 3 [file DataSheet3.zip › train1/2060-2024-3-19-1-27-29.JPG]

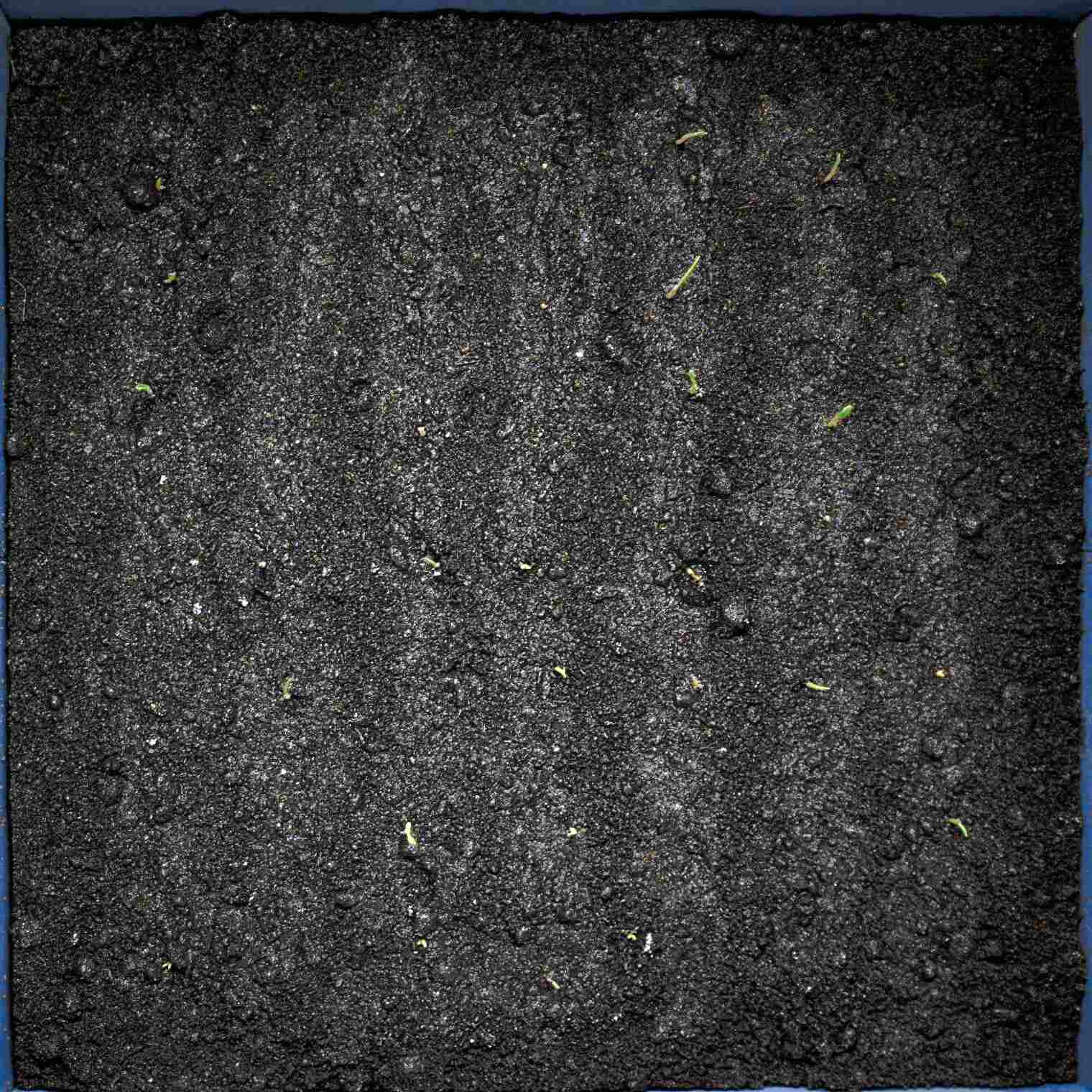

Supplement: Supplementary file 3 [file DataSheet3.zip › train1/2060-2024-3-19-11-38-12.JPG]

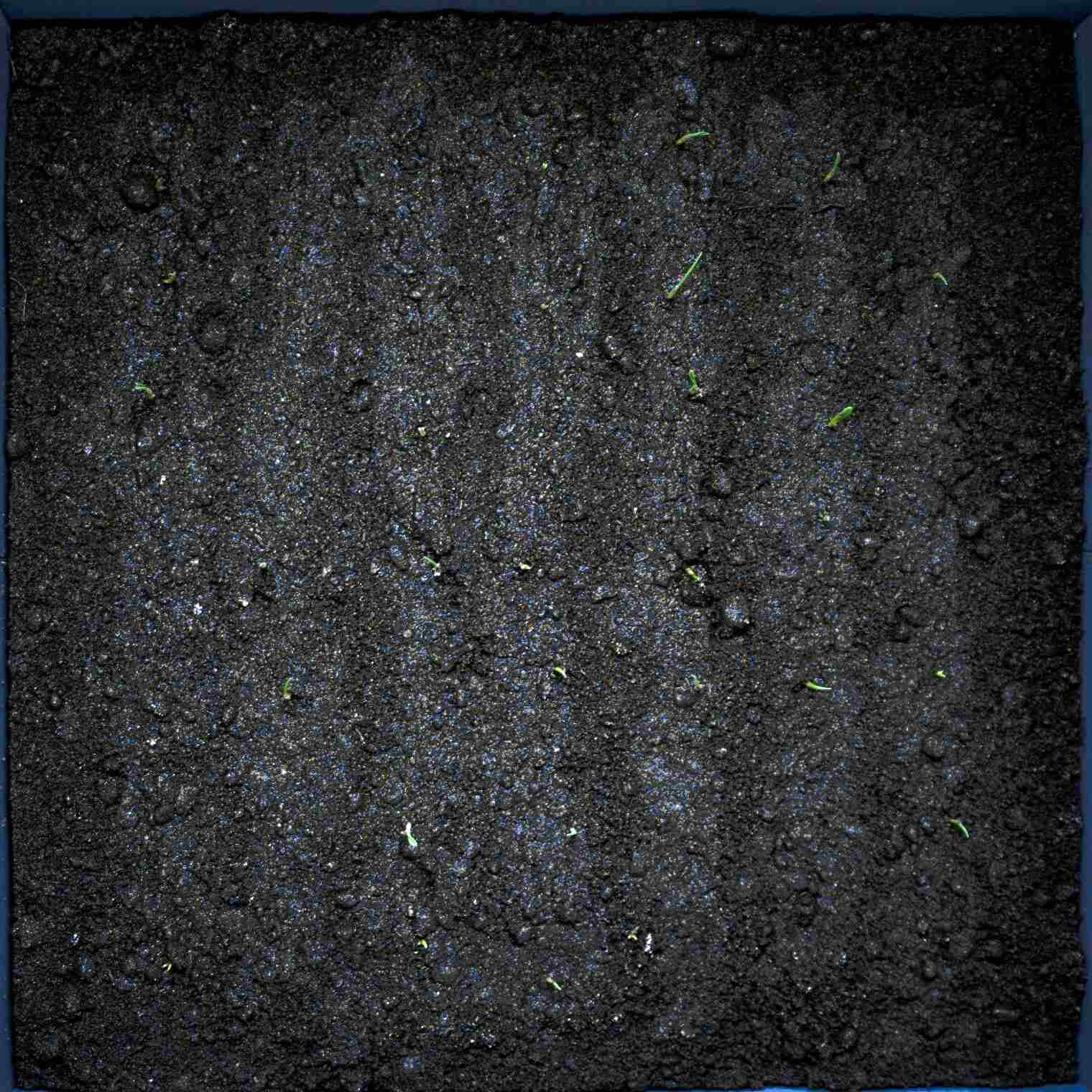

Supplement: Supplementary file 3 [file DataSheet3.zip › train1/2060-2024-3-19-14-9-54.JPG]

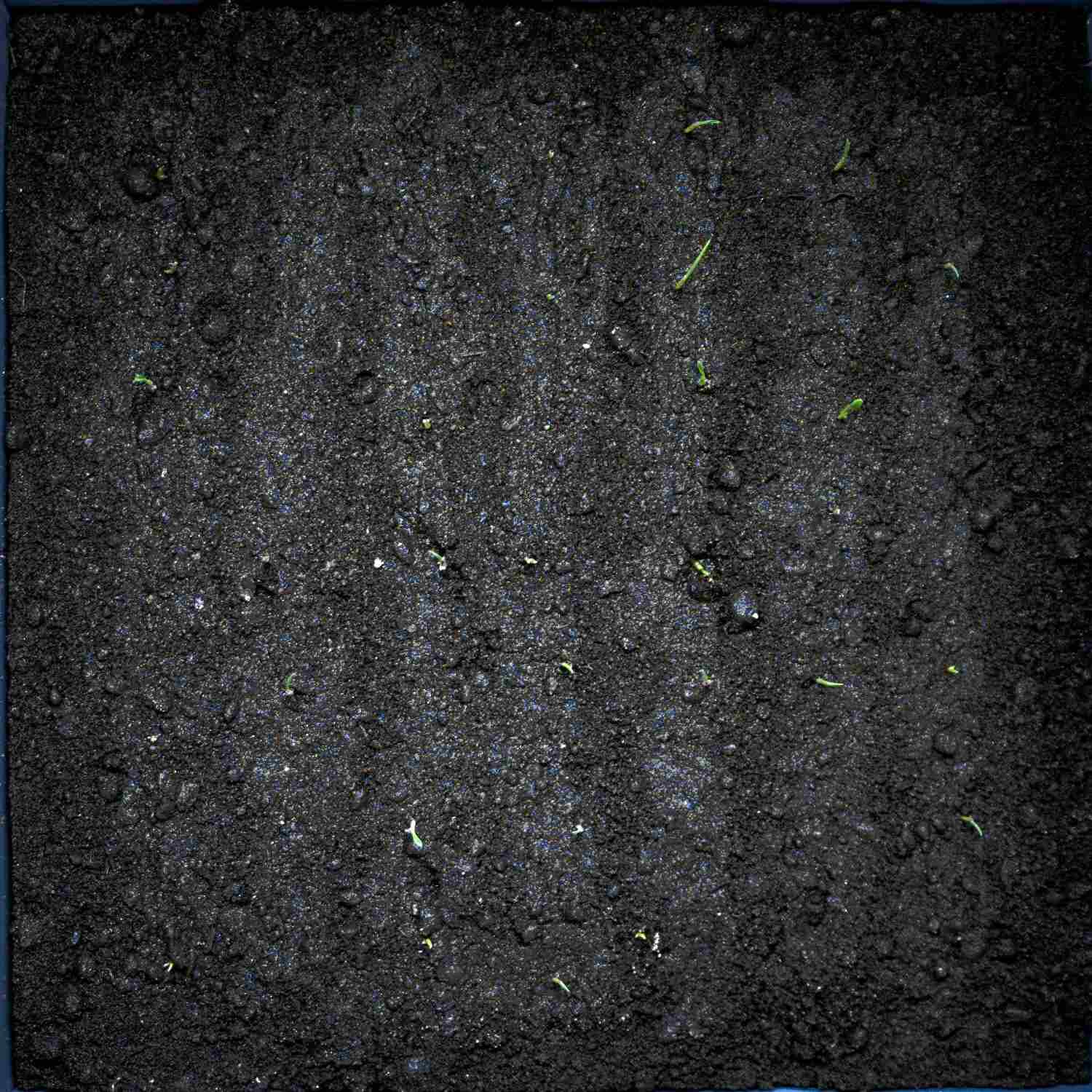

Supplement: Supplementary file 3 [file DataSheet3.zip › train1/2060-2024-3-19-16-42-2.JPG]

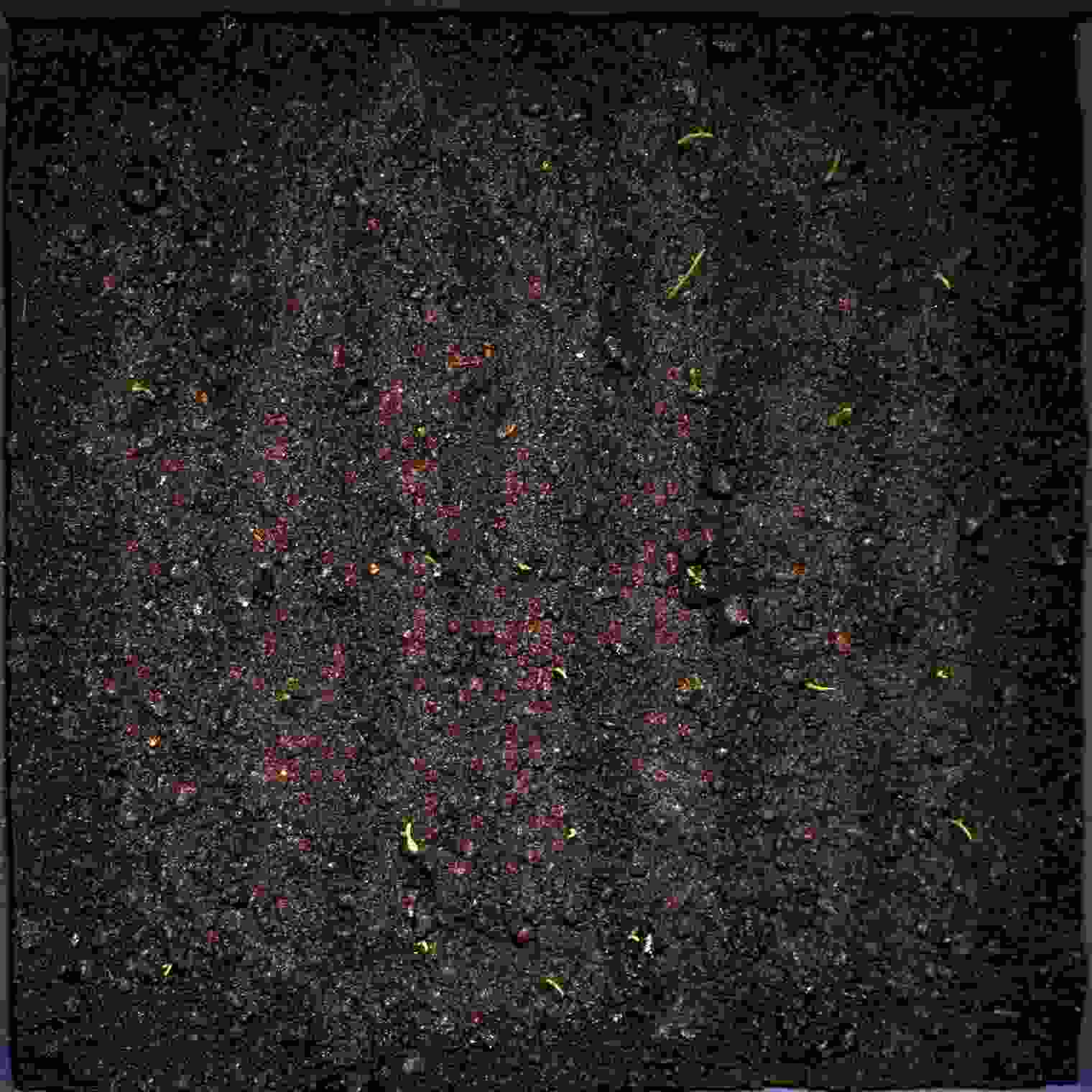

Supplement: Supplementary file 3 [file DataSheet3.zip › train1/2060-2024-3-19-19-13-58.JPG]

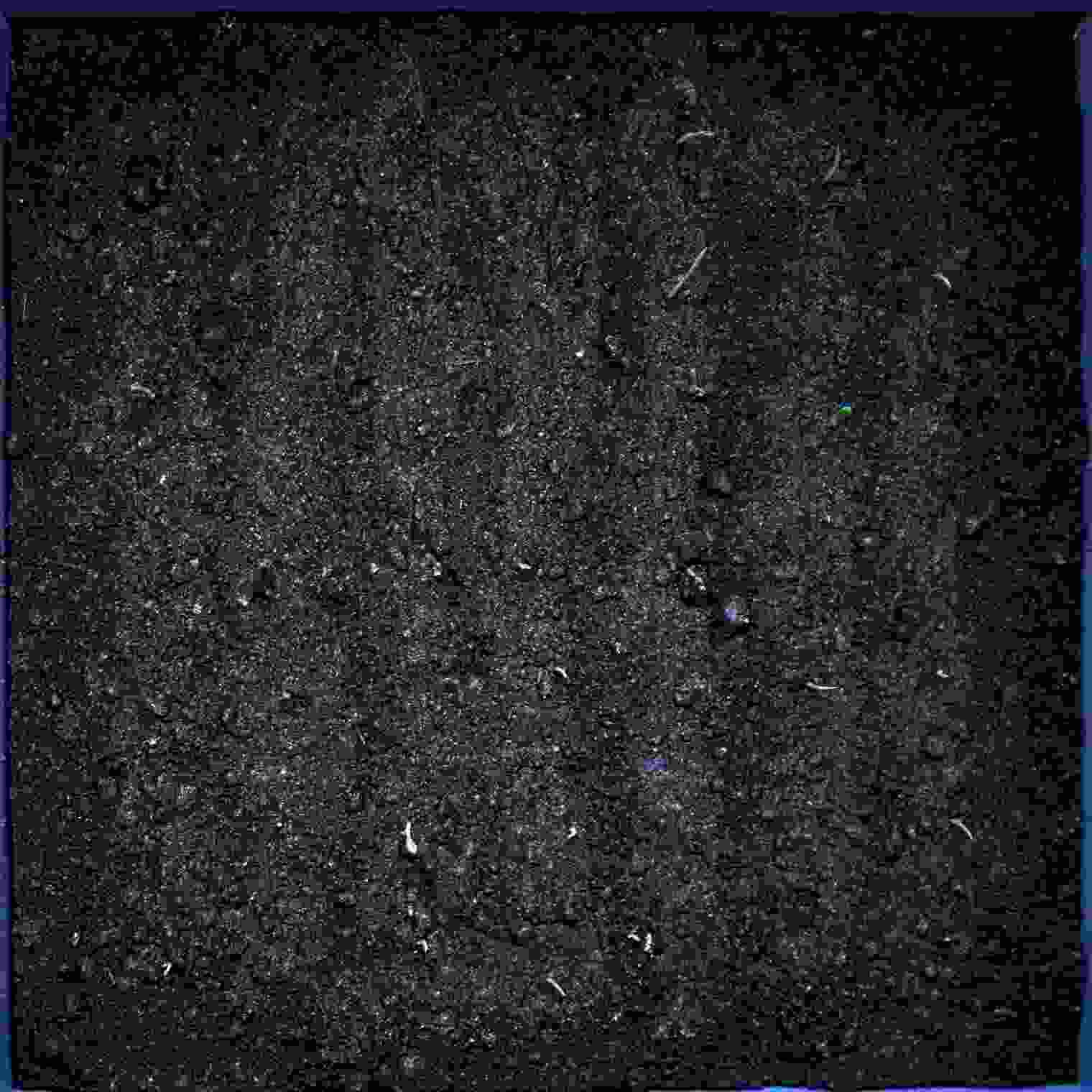

Supplement: Supplementary file 3 [file DataSheet3.zip › train1/2060-2024-3-19-21-45-47.JPG]

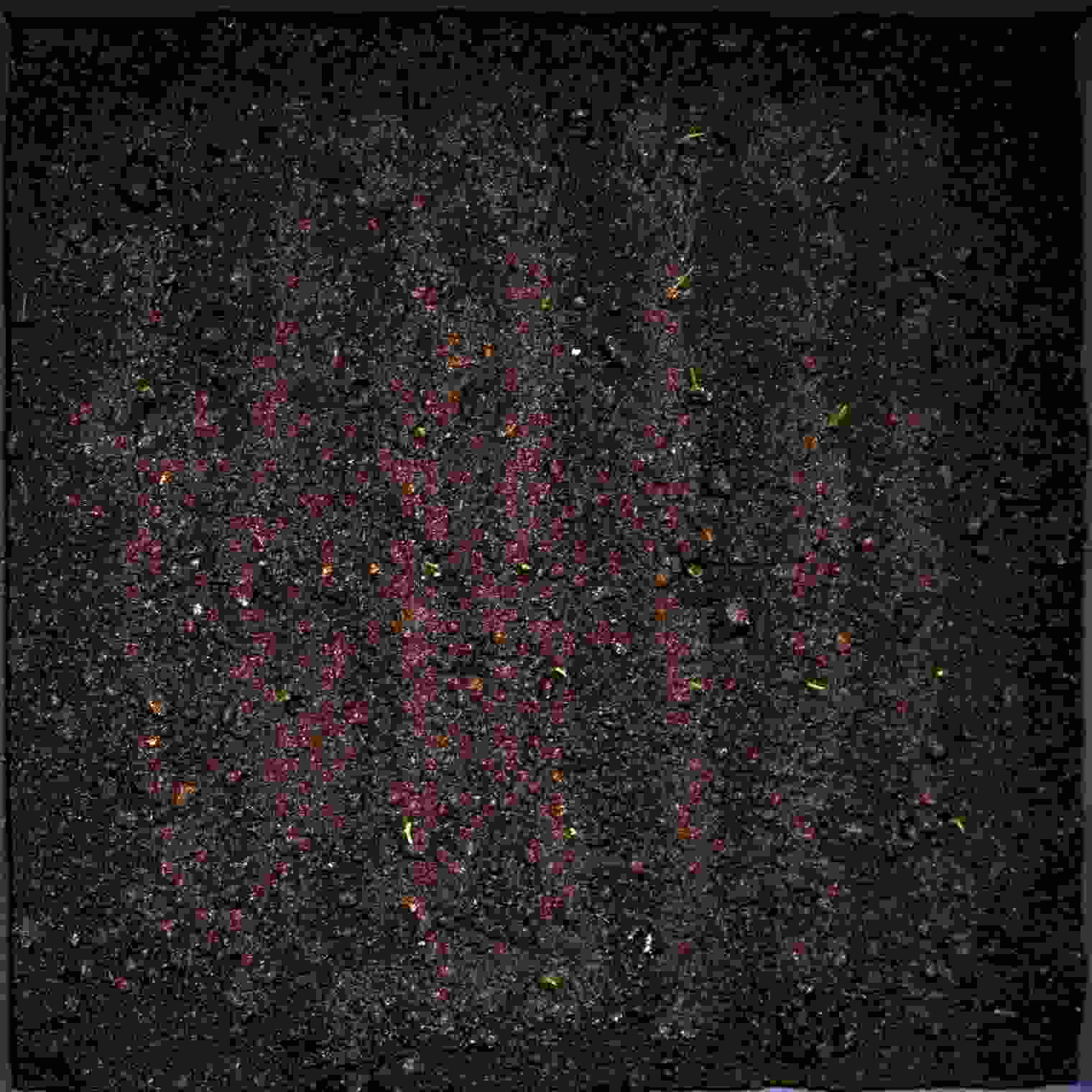

Supplement: Supplementary file 3 [file DataSheet3.zip › train1/2060-2024-3-19-3-59-57.JPG]

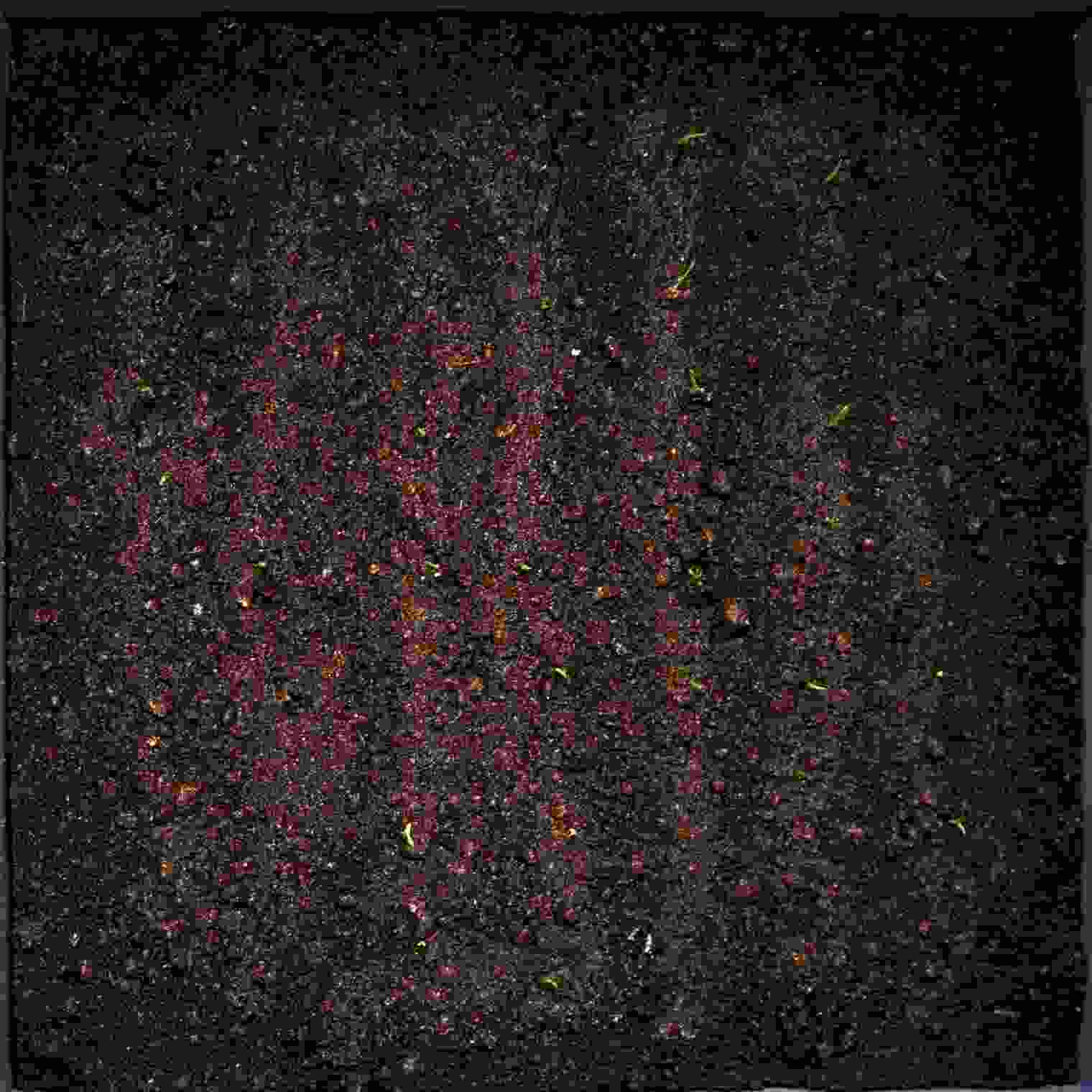

Supplement: Supplementary file 3 [file DataSheet3.zip › train1/2060-2024-3-19-6-32-44.JPG]

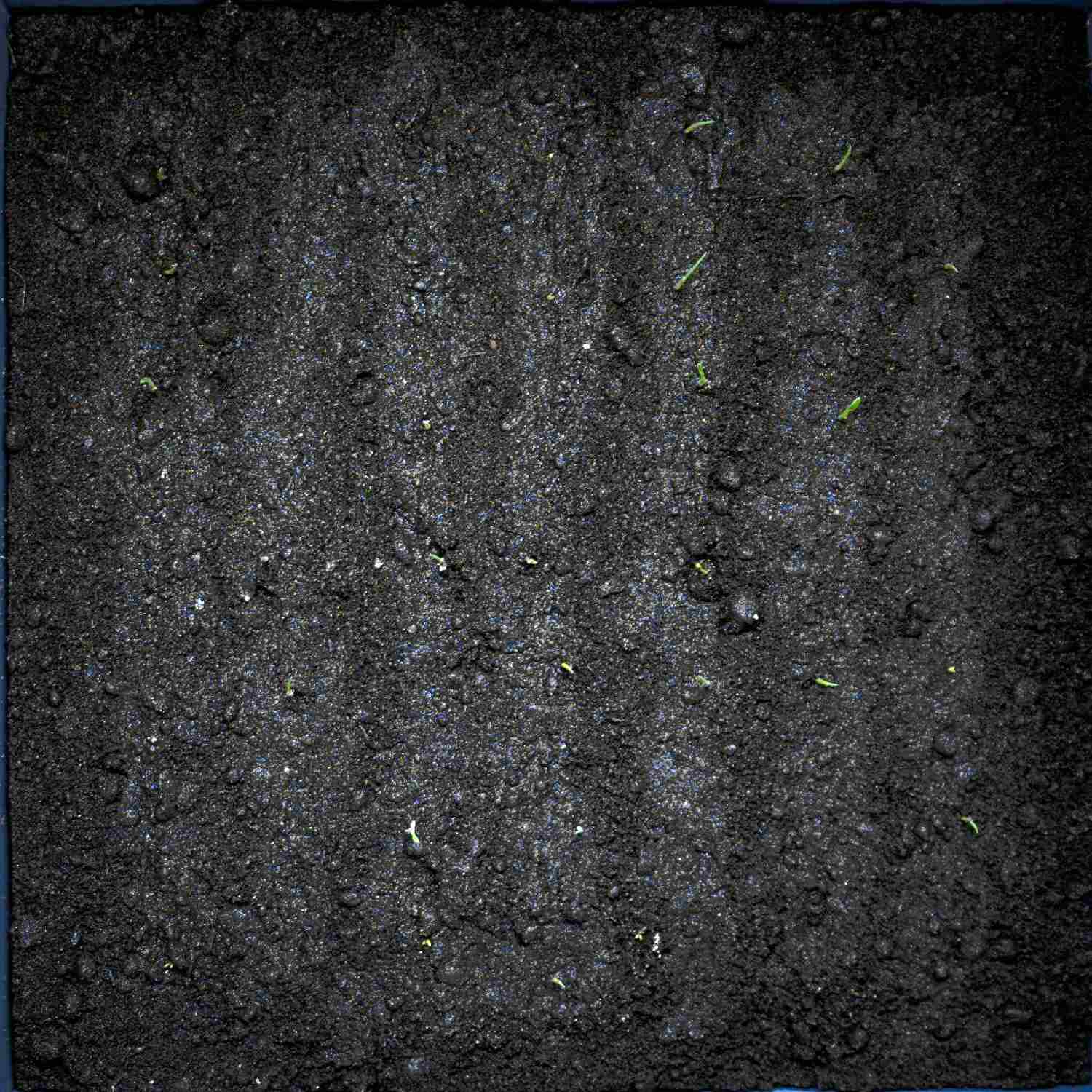

Supplement: Supplementary file 3 [file DataSheet3.zip › train1/2060-2024-3-19-9-5-22.JPG]

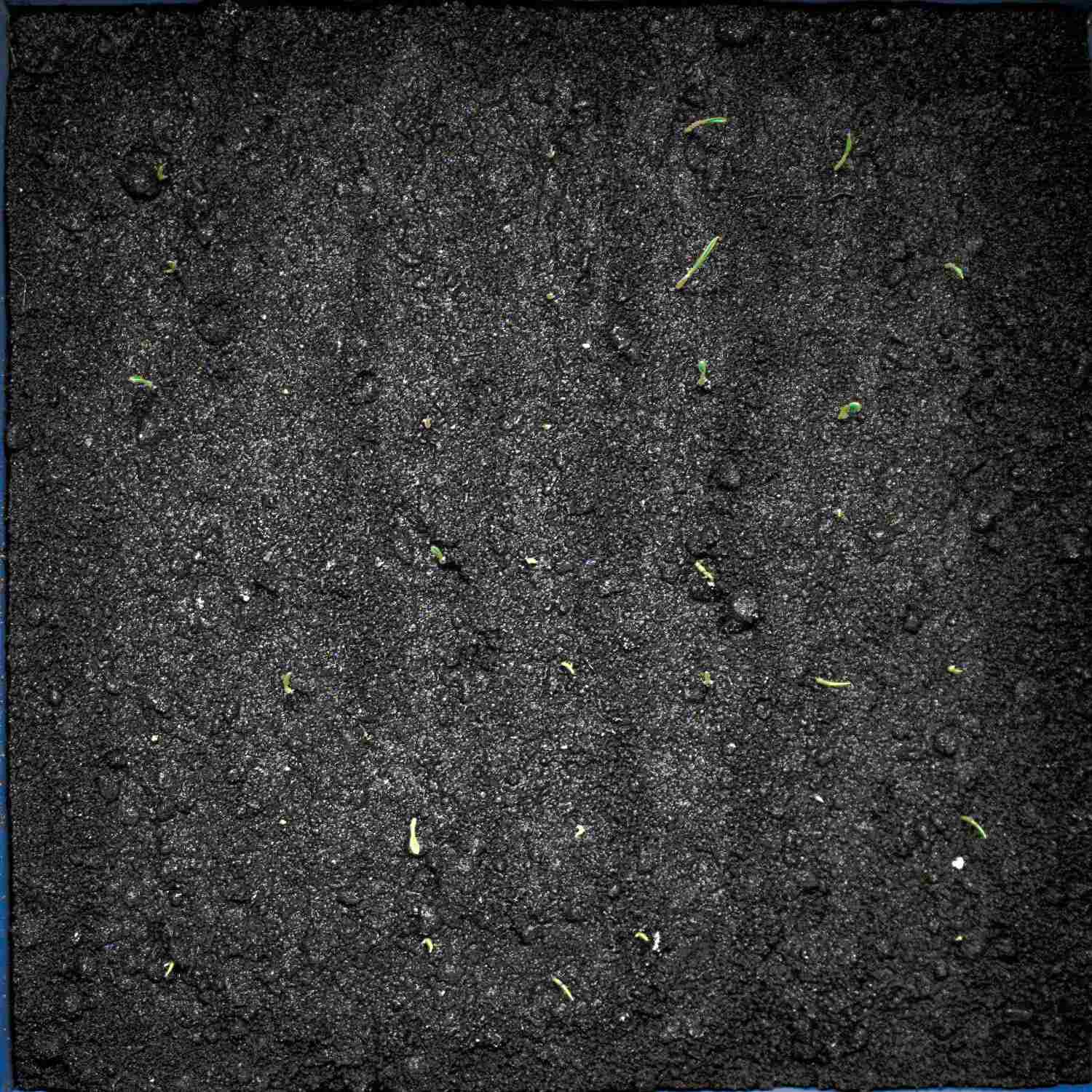

Supplement: Supplementary file 3 [file DataSheet3.zip › train1/2060-2024-3-20-0-18-53.JPG]

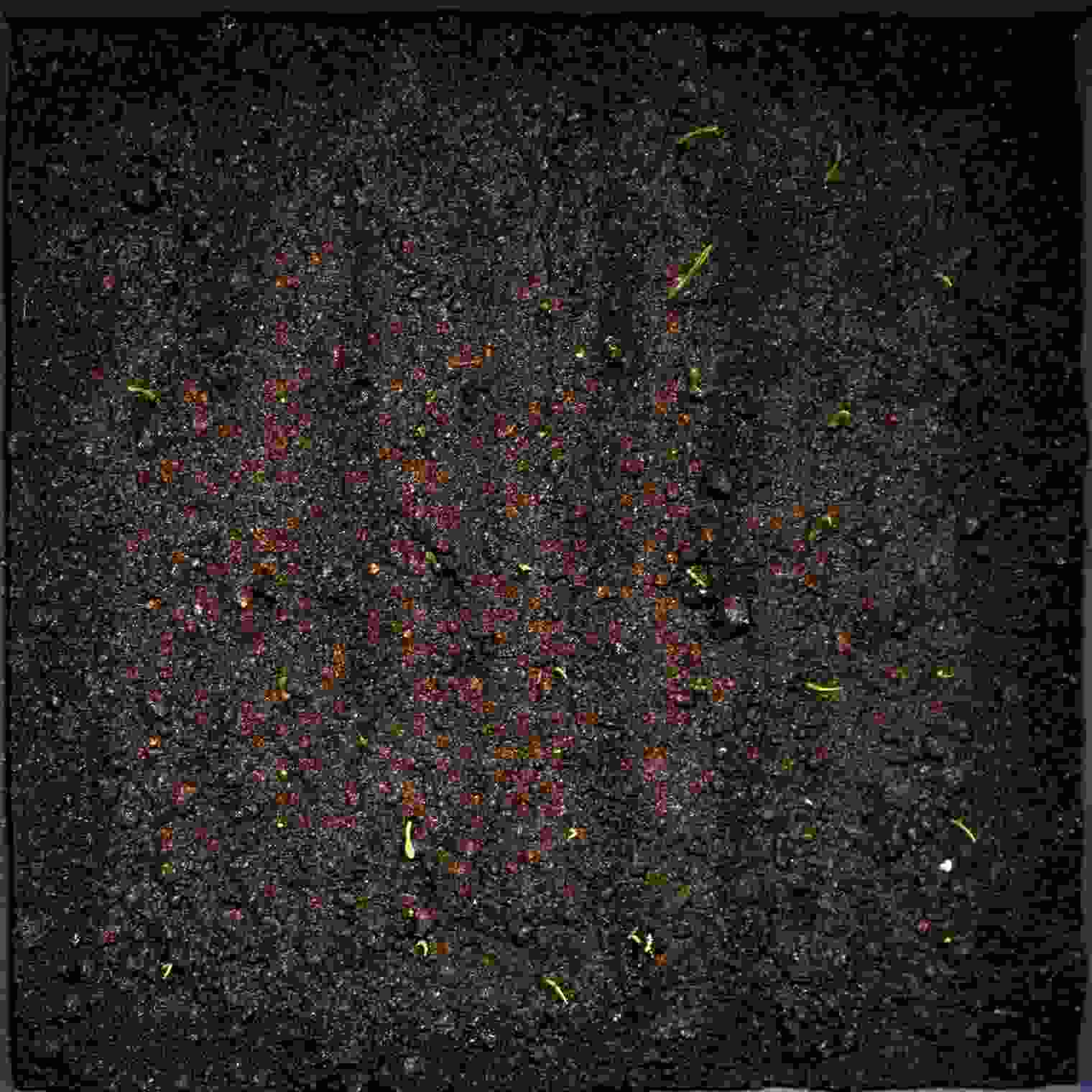

Supplement: Supplementary file 3 [file DataSheet3.zip › train1/2060-2024-3-20-2-51-0.JPG]

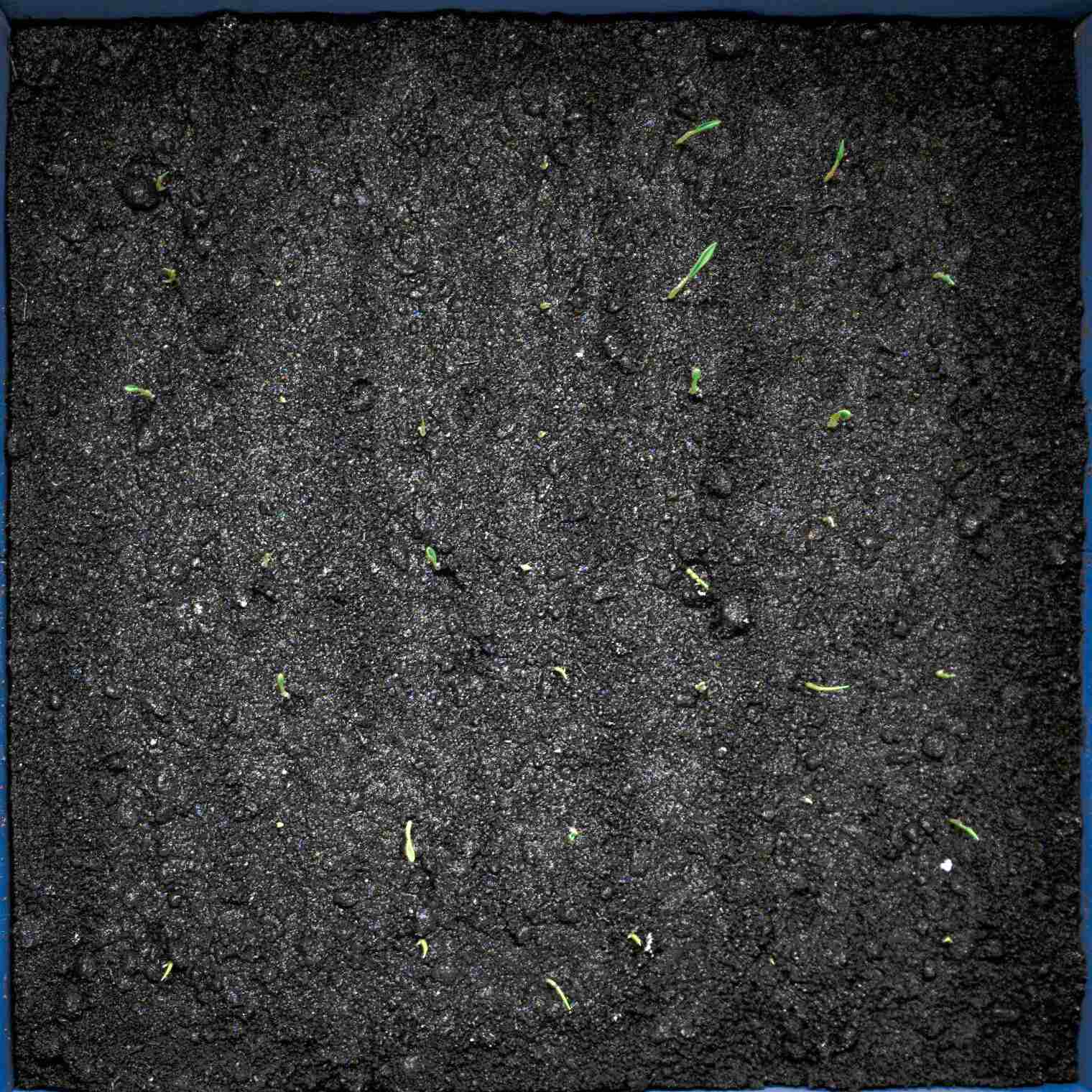

Supplement: Supplementary file 3 [file DataSheet3.zip › train1/2060-2024-3-20-7-54-57.JPG]

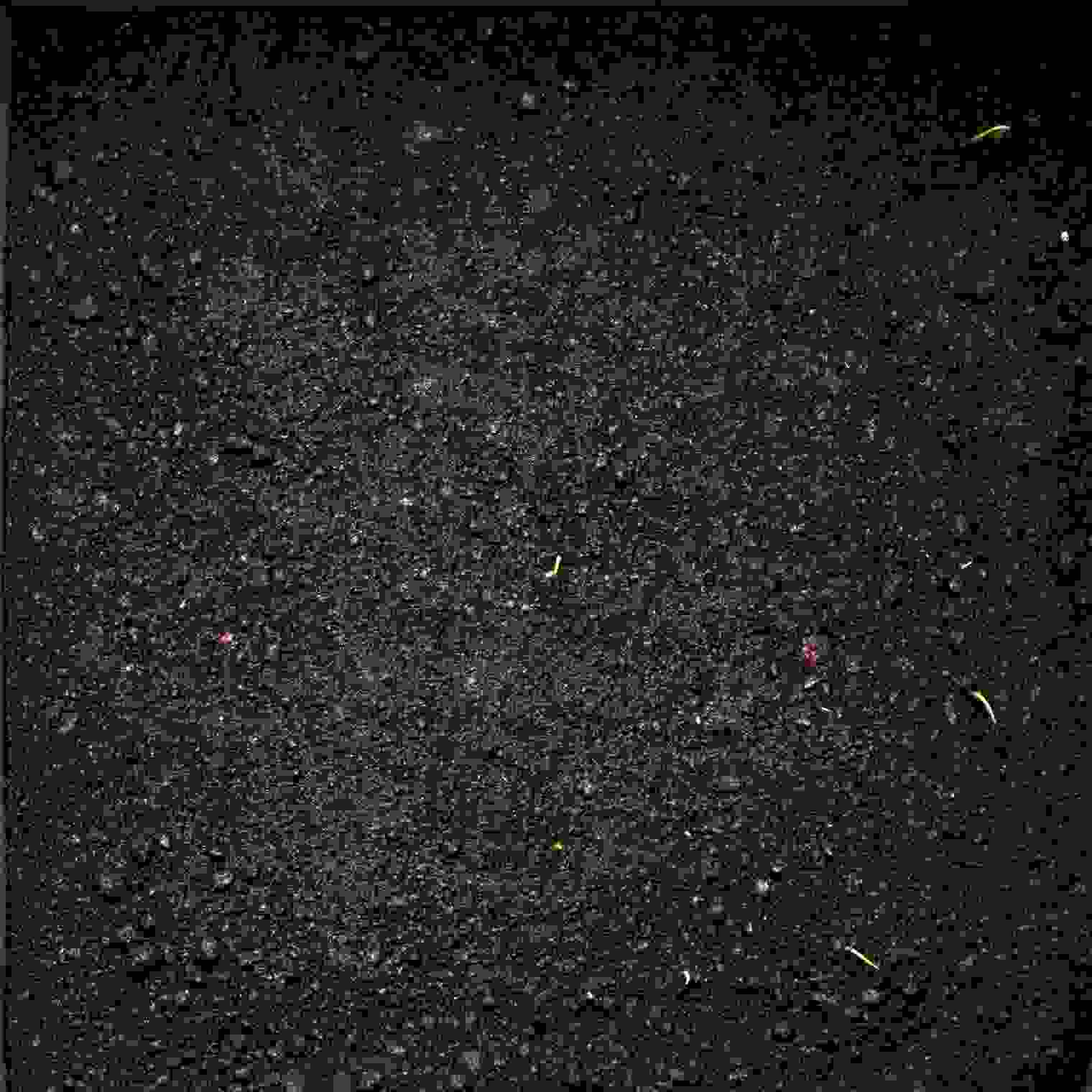

Supplement: Supplementary file 3 [file DataSheet3.zip › train1/2090-2024-3-18-15-32-40.JPG]
