# Supplementary figures and images for: VanillaNet-YOLOv8 segment: detection of nano-iron oxide regulation on rice seedling growth vitality under salt stress (part 3 of 5)
Source: Front Plant Sci. 2025 Sep 17;16:1631279. doi: 10.3389/fpls.2025.1631279 (PMC12484053; doi:10.3389/fpls.2025.1631279)

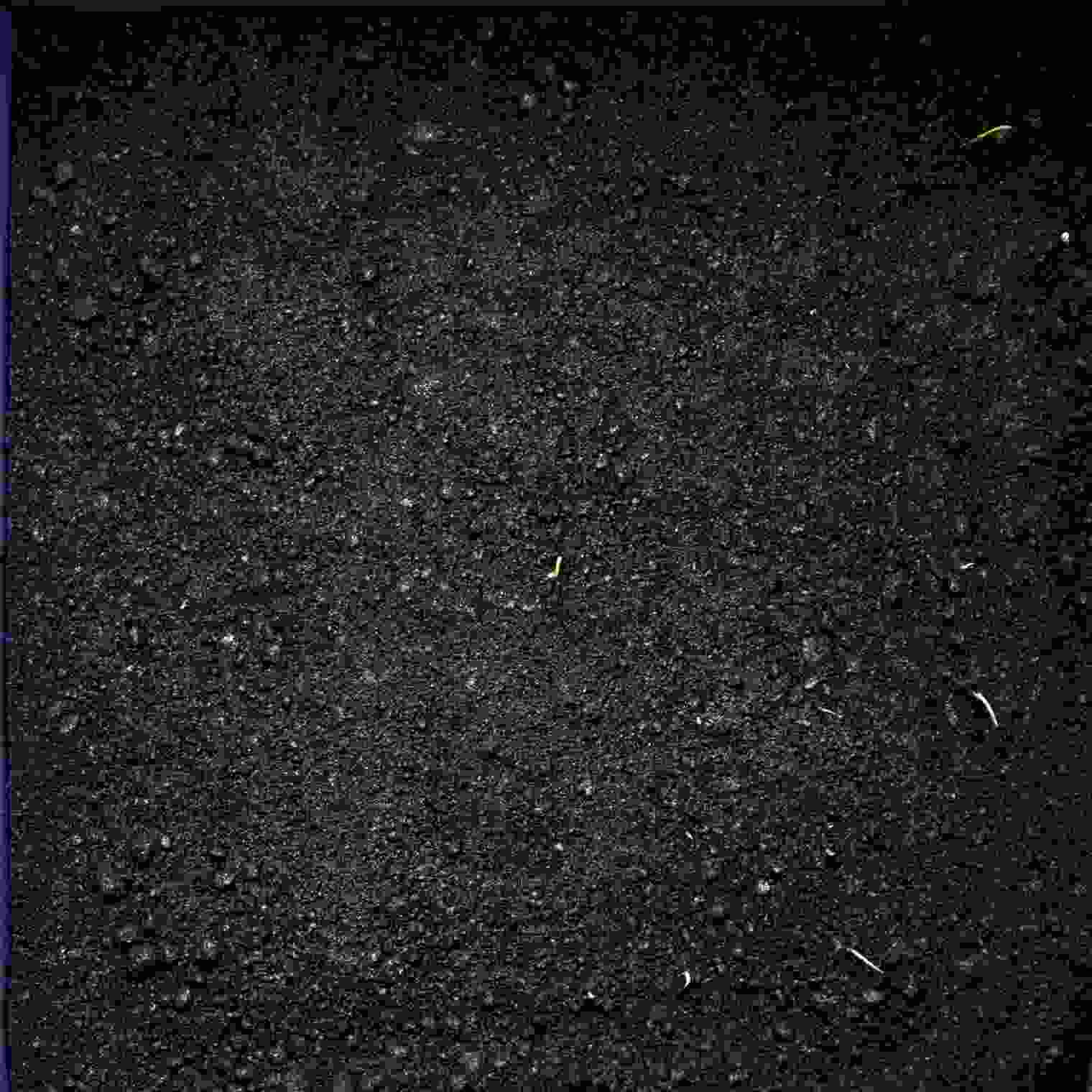

Supplement: Supplementary file 3 [file DataSheet3.zip › train1/2090-2024-3-18-18-6-15.JPG]

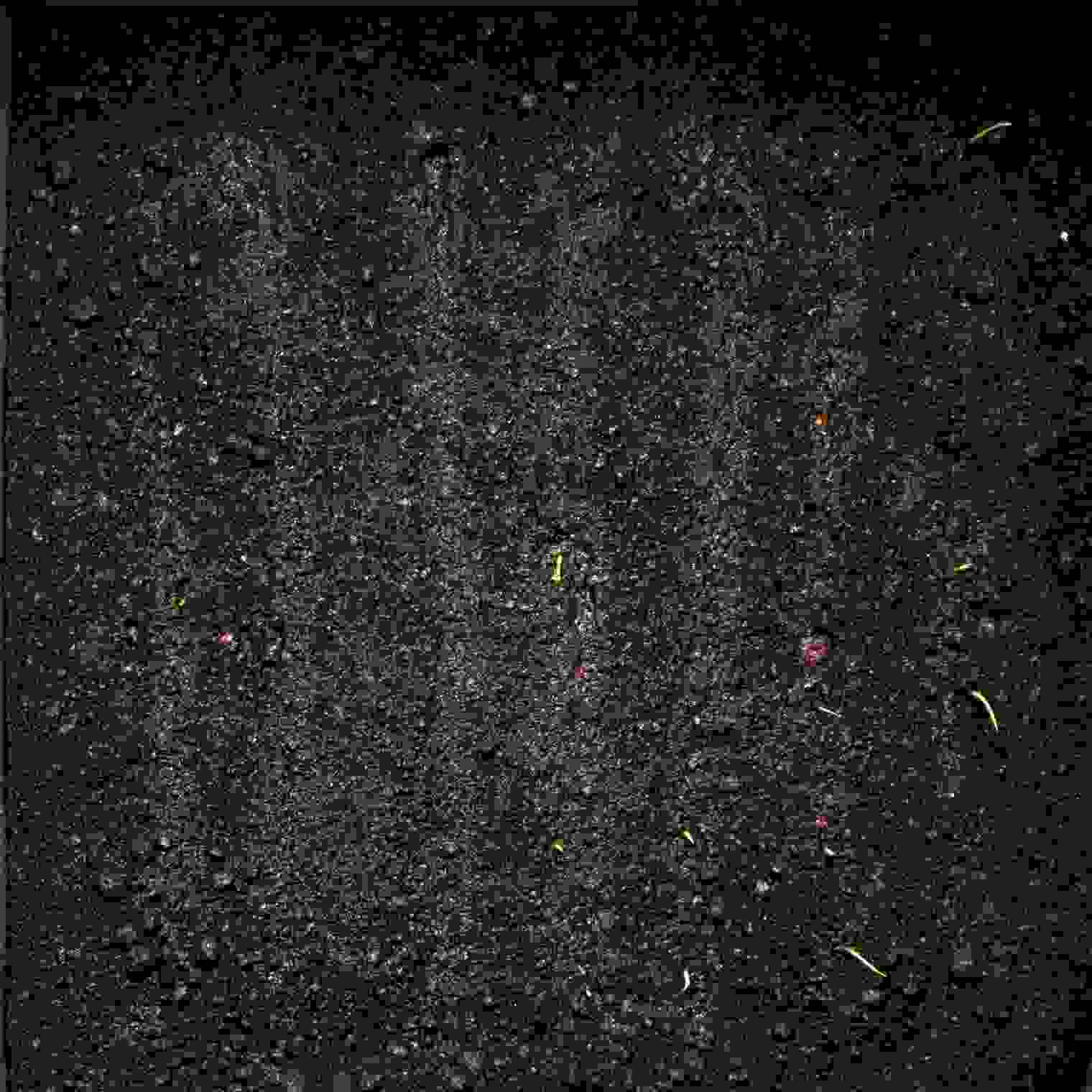

Supplement: Supplementary file 3 [file DataSheet3.zip › train1/2090-2024-3-18-23-14-13.JPG]

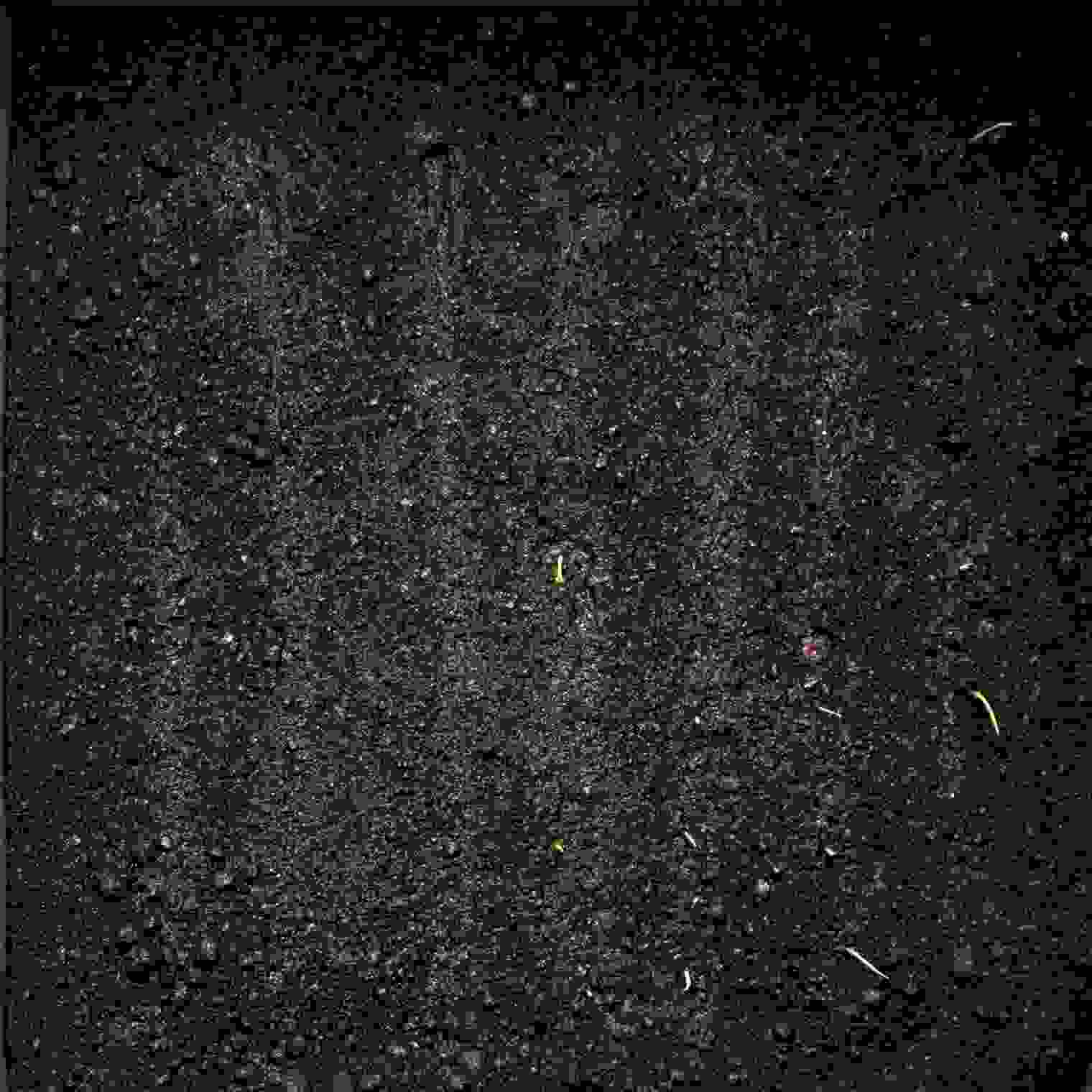

Supplement: Supplementary file 3 [file DataSheet3.zip › train1/2090-2024-3-19-1-47-24.JPG]

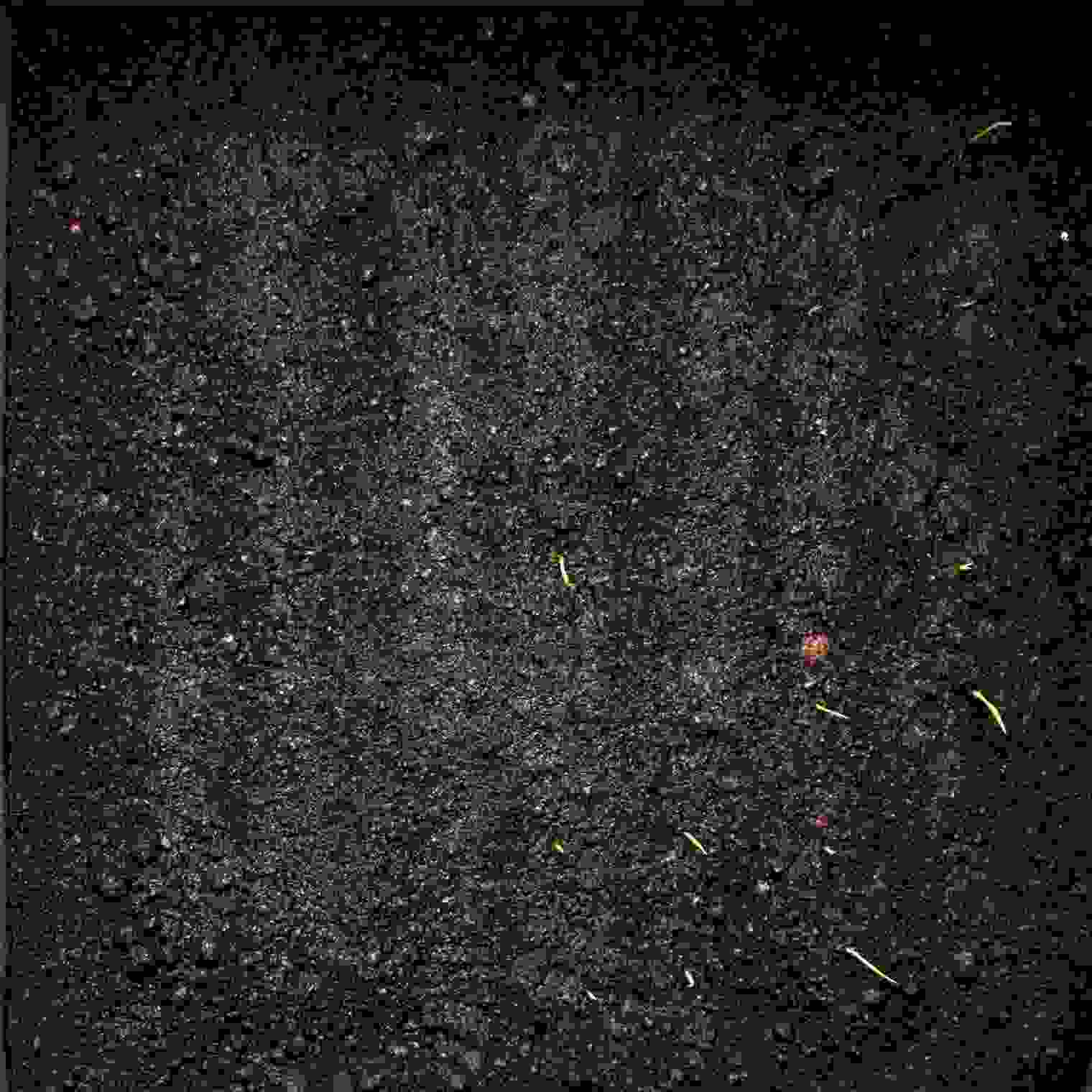

Supplement: Supplementary file 3 [file DataSheet3.zip › train1/2090-2024-3-19-11-58-0.JPG]

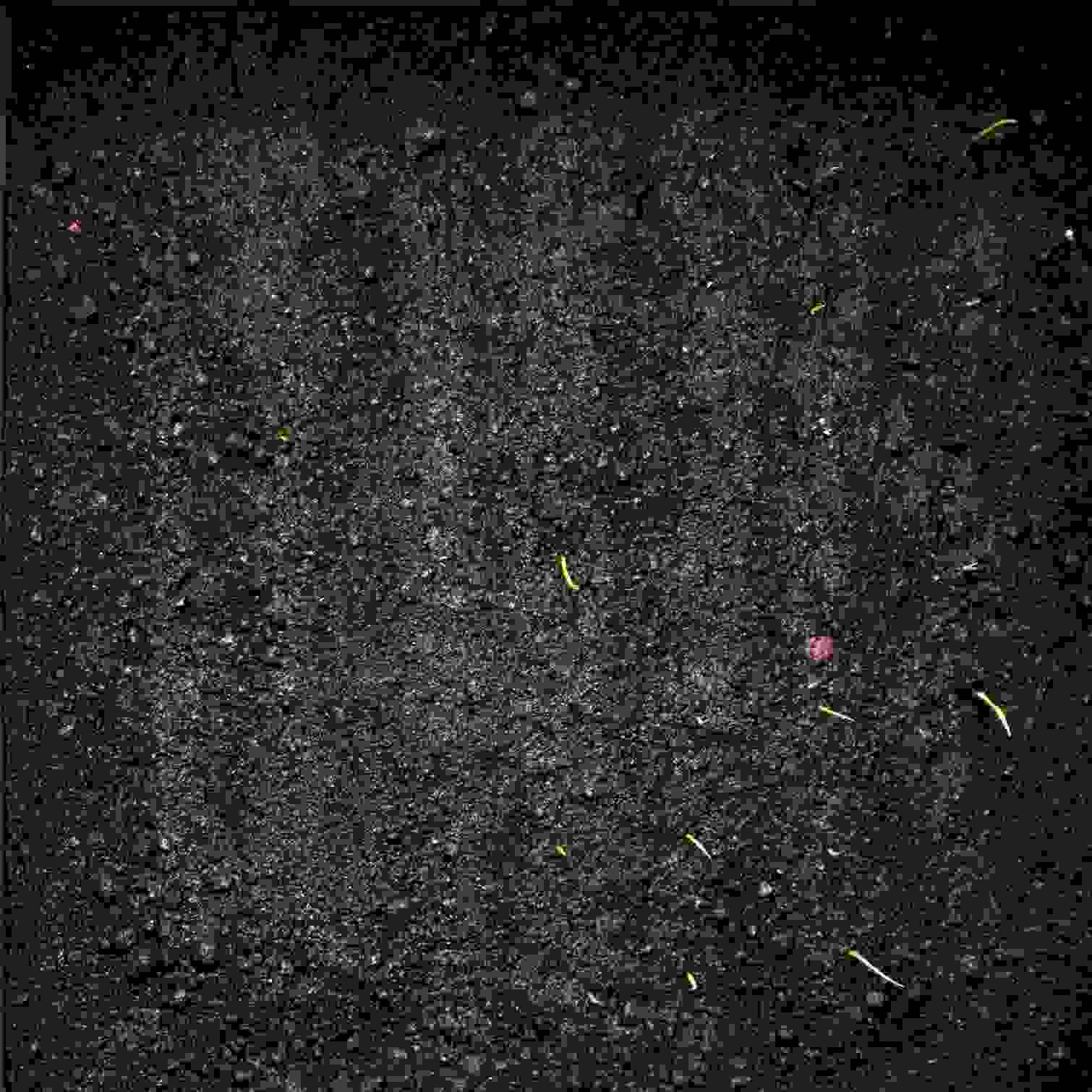

Supplement: Supplementary file 3 [file DataSheet3.zip › train1/2090-2024-3-19-14-29-44.JPG]

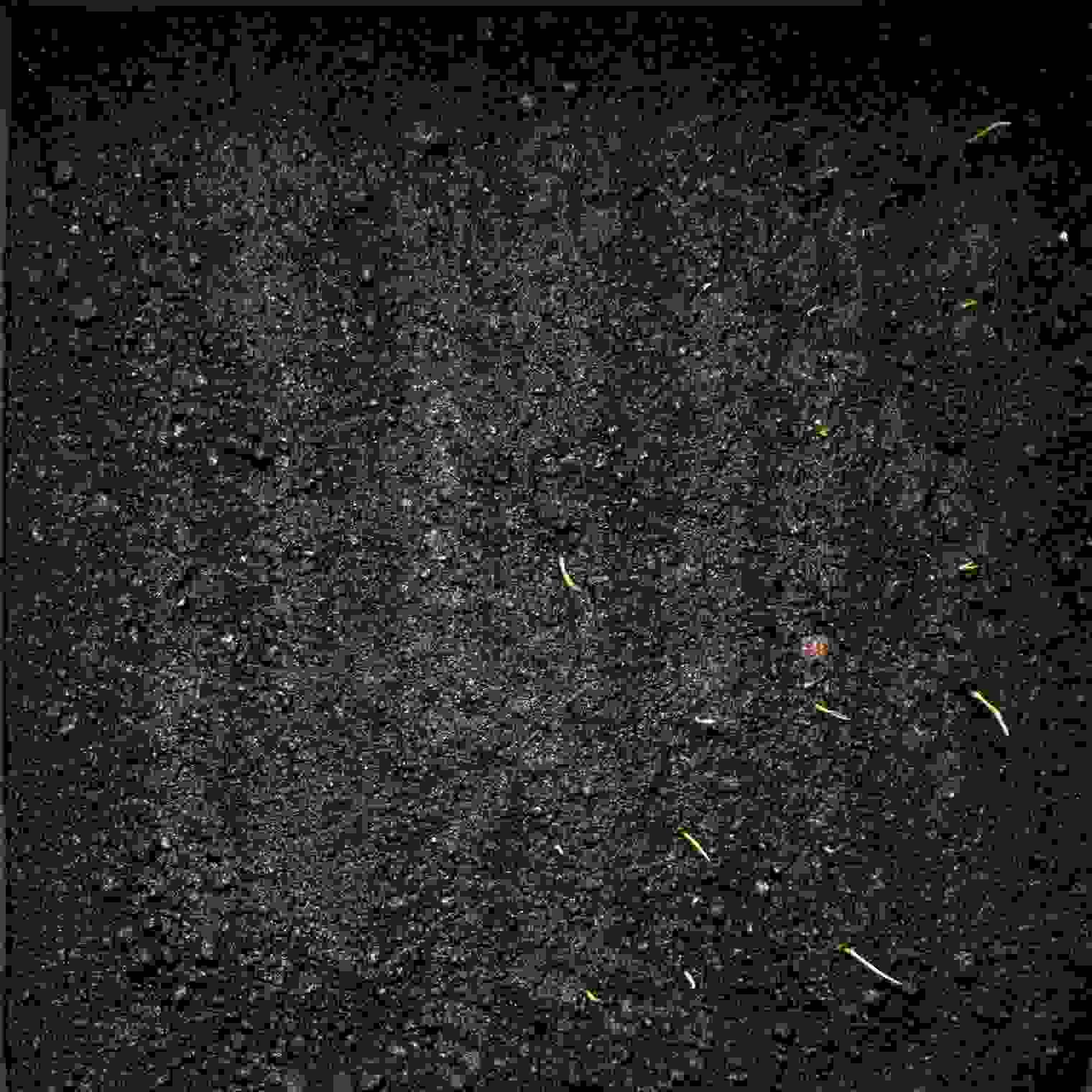

Supplement: Supplementary file 3 [file DataSheet3.zip › train1/2090-2024-3-19-19-33-48.JPG]

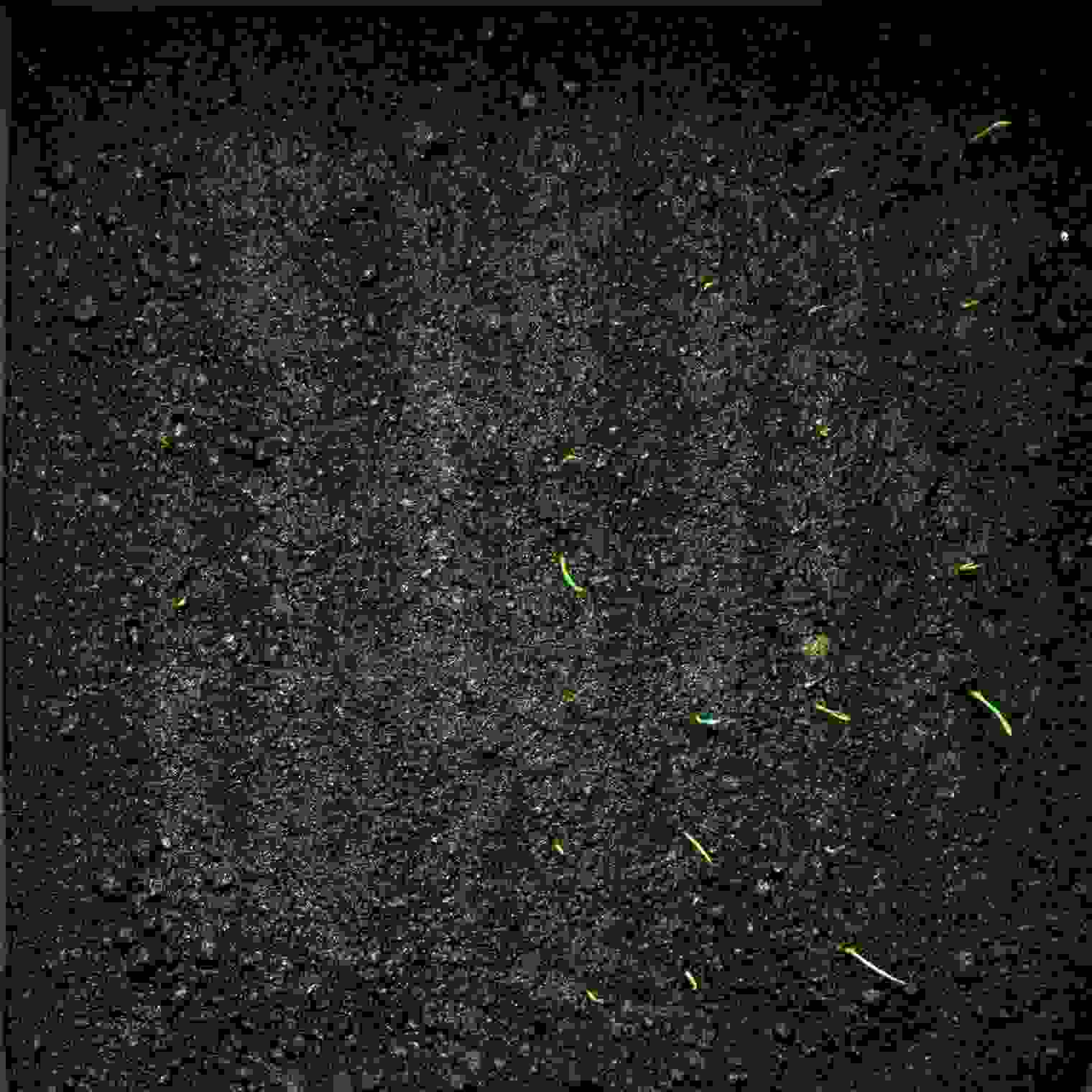

Supplement: Supplementary file 3 [file DataSheet3.zip › train1/2090-2024-3-19-22-5-39.JPG]

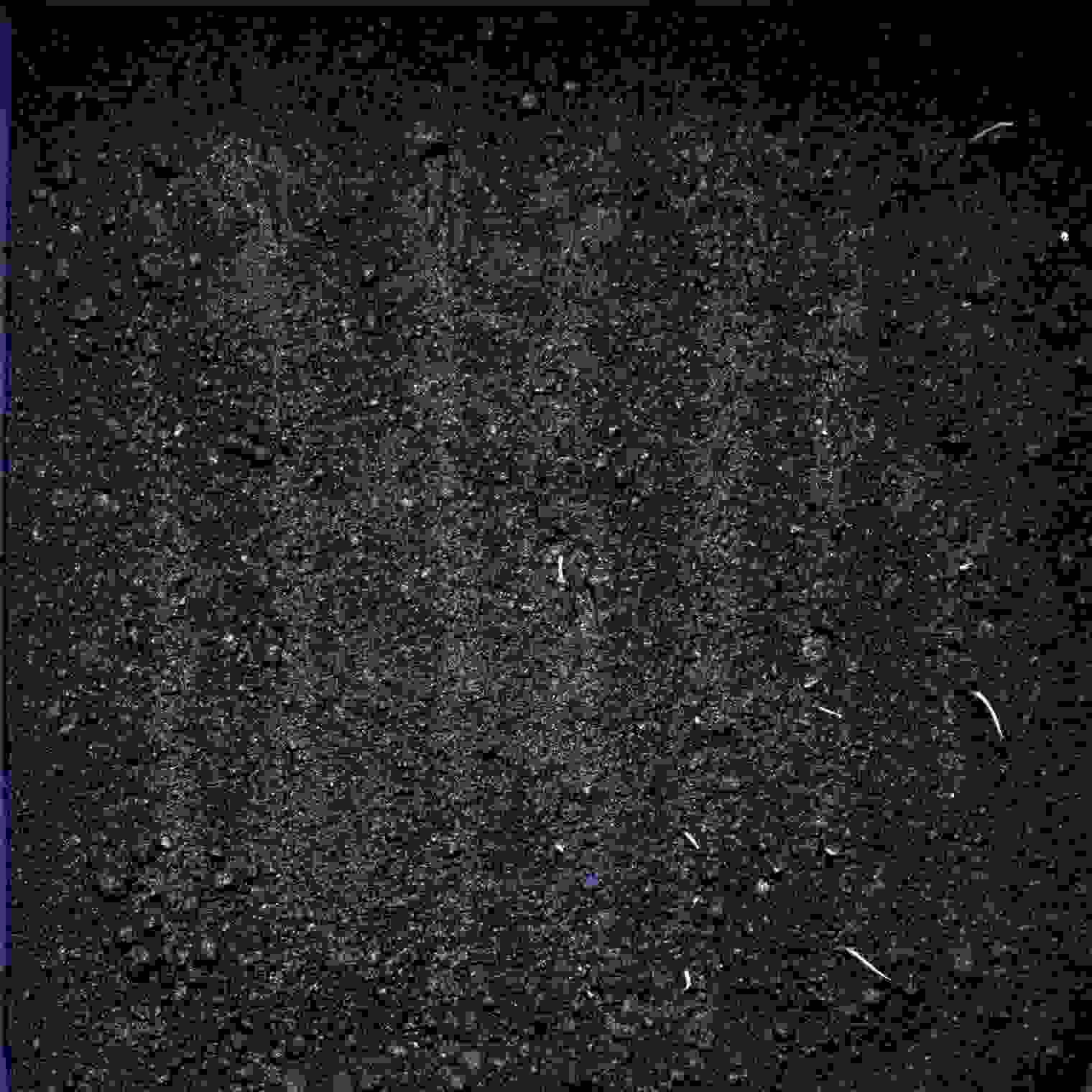

Supplement: Supplementary file 3 [file DataSheet3.zip › train1/2090-2024-3-19-4-19-53.JPG]

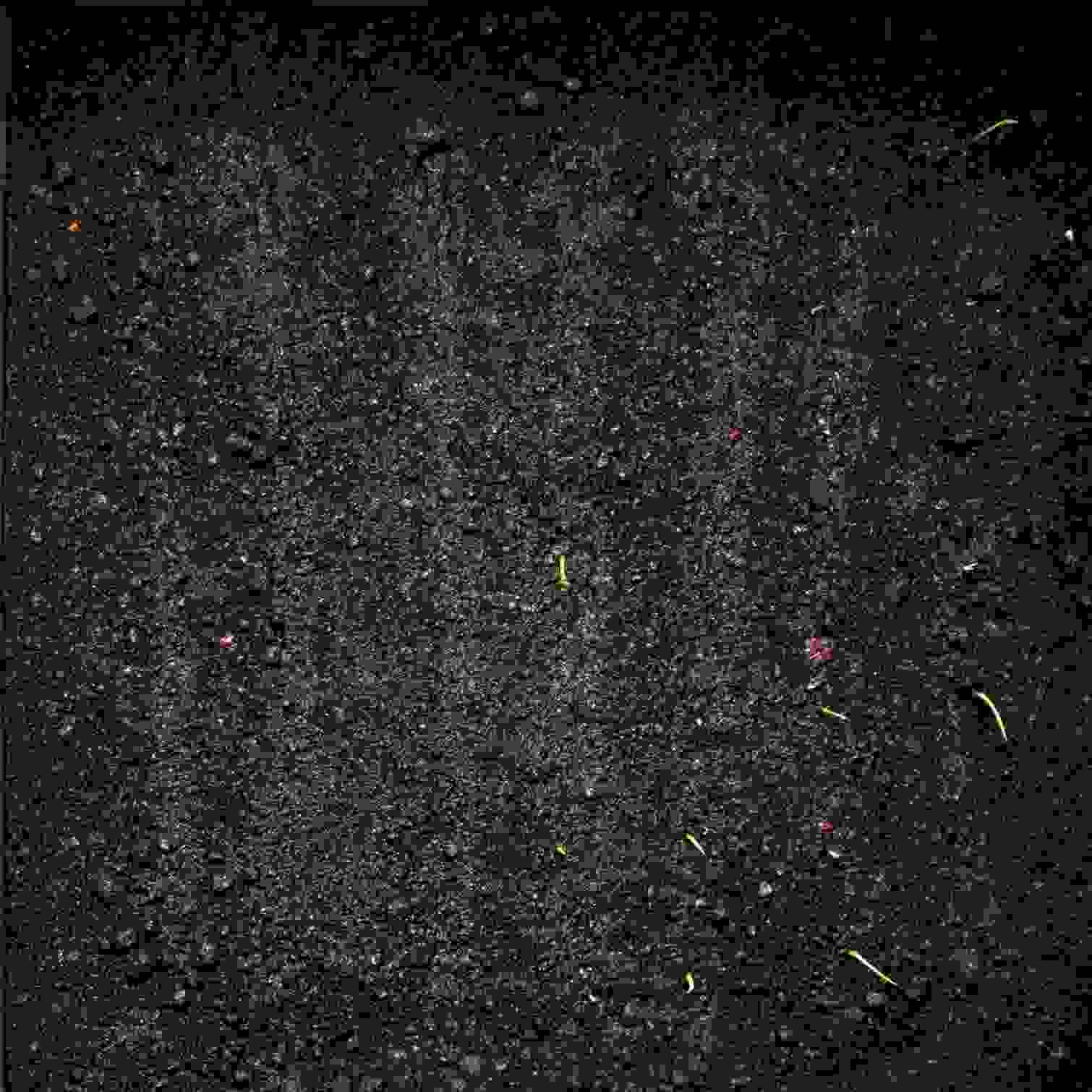

Supplement: Supplementary file 3 [file DataSheet3.zip › train1/2090-2024-3-19-6-52-36.JPG]

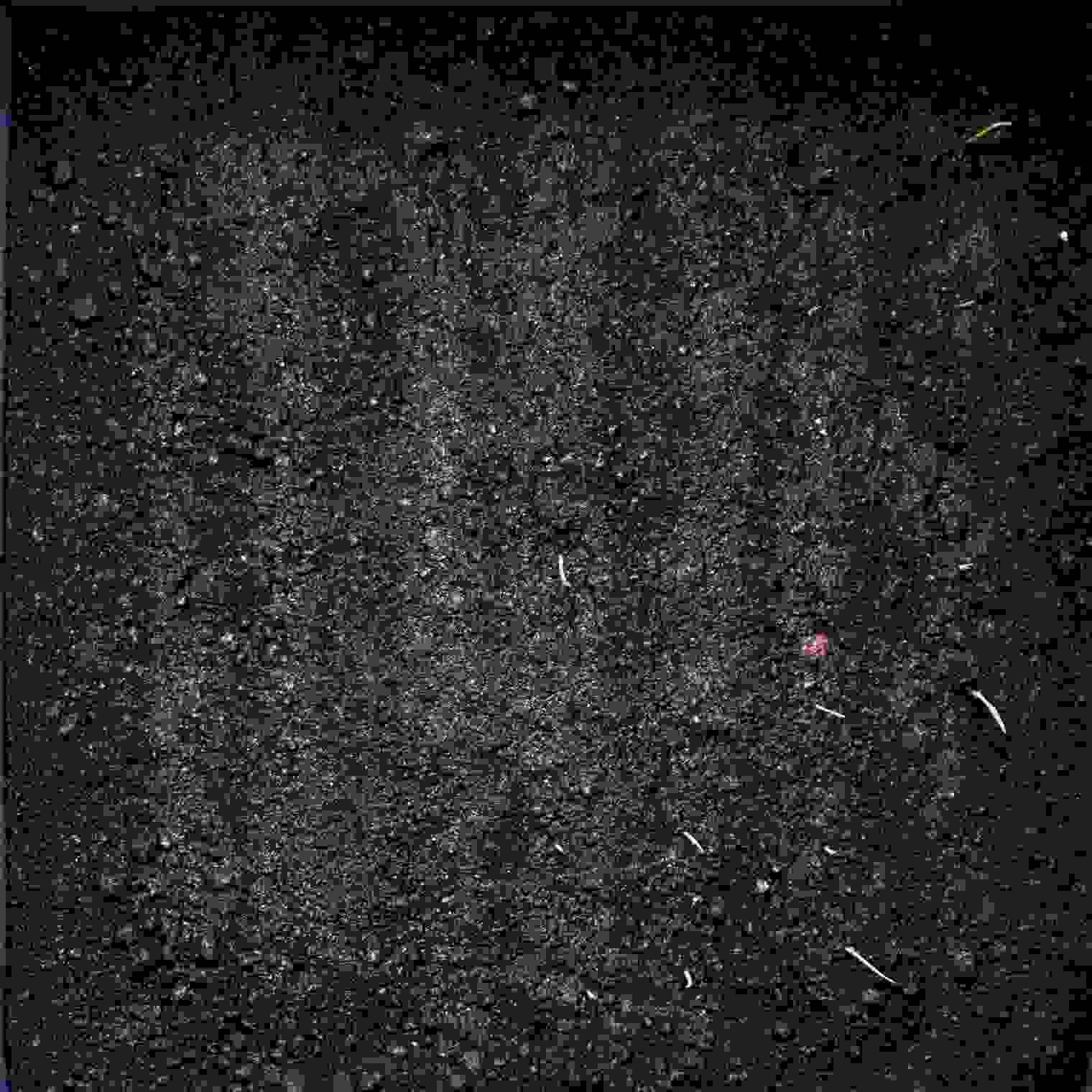

Supplement: Supplementary file 3 [file DataSheet3.zip › train1/2090-2024-3-19-9-25-25.JPG]

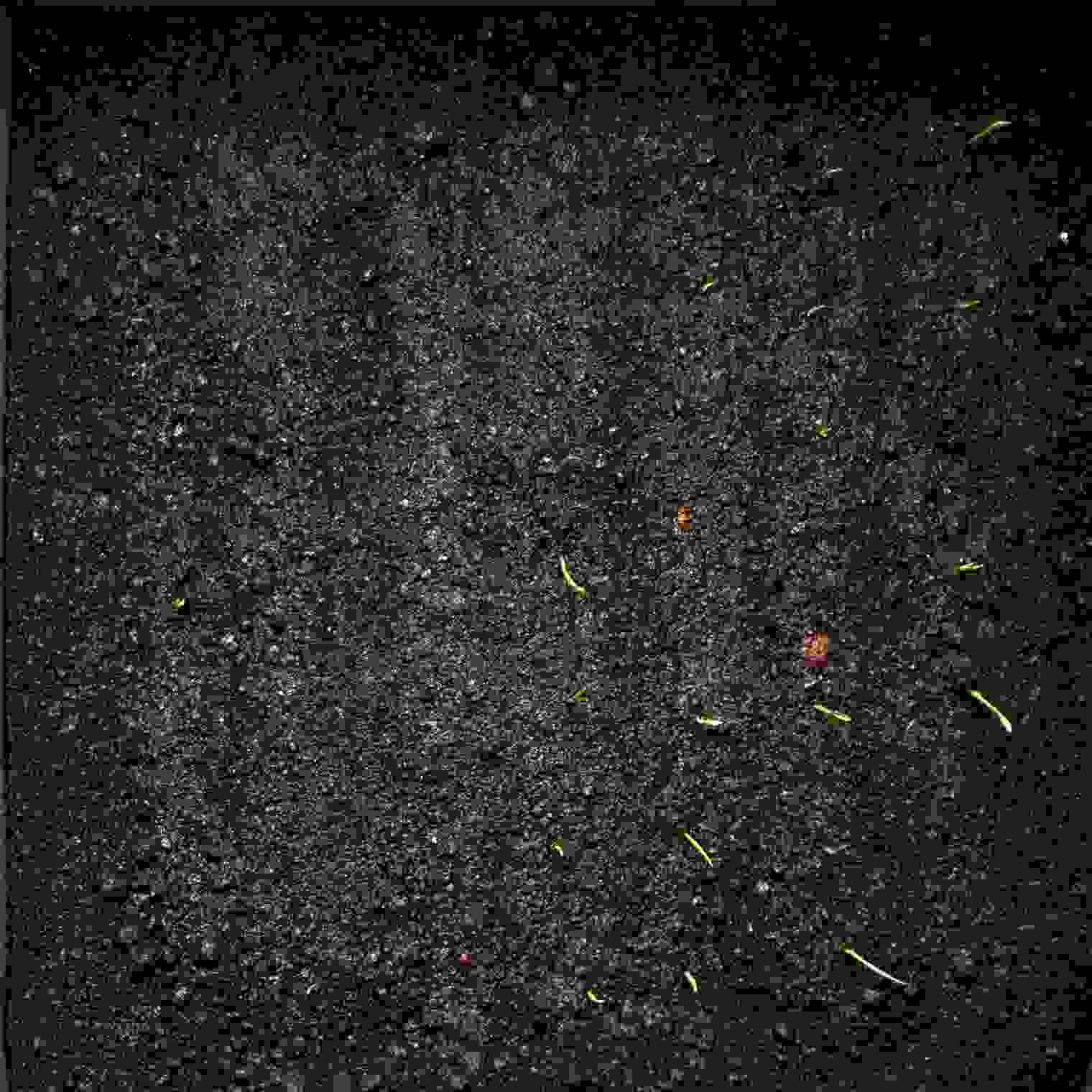

Supplement: Supplementary file 3 [file DataSheet3.zip › train1/2090-2024-3-20-0-38-51.JPG]

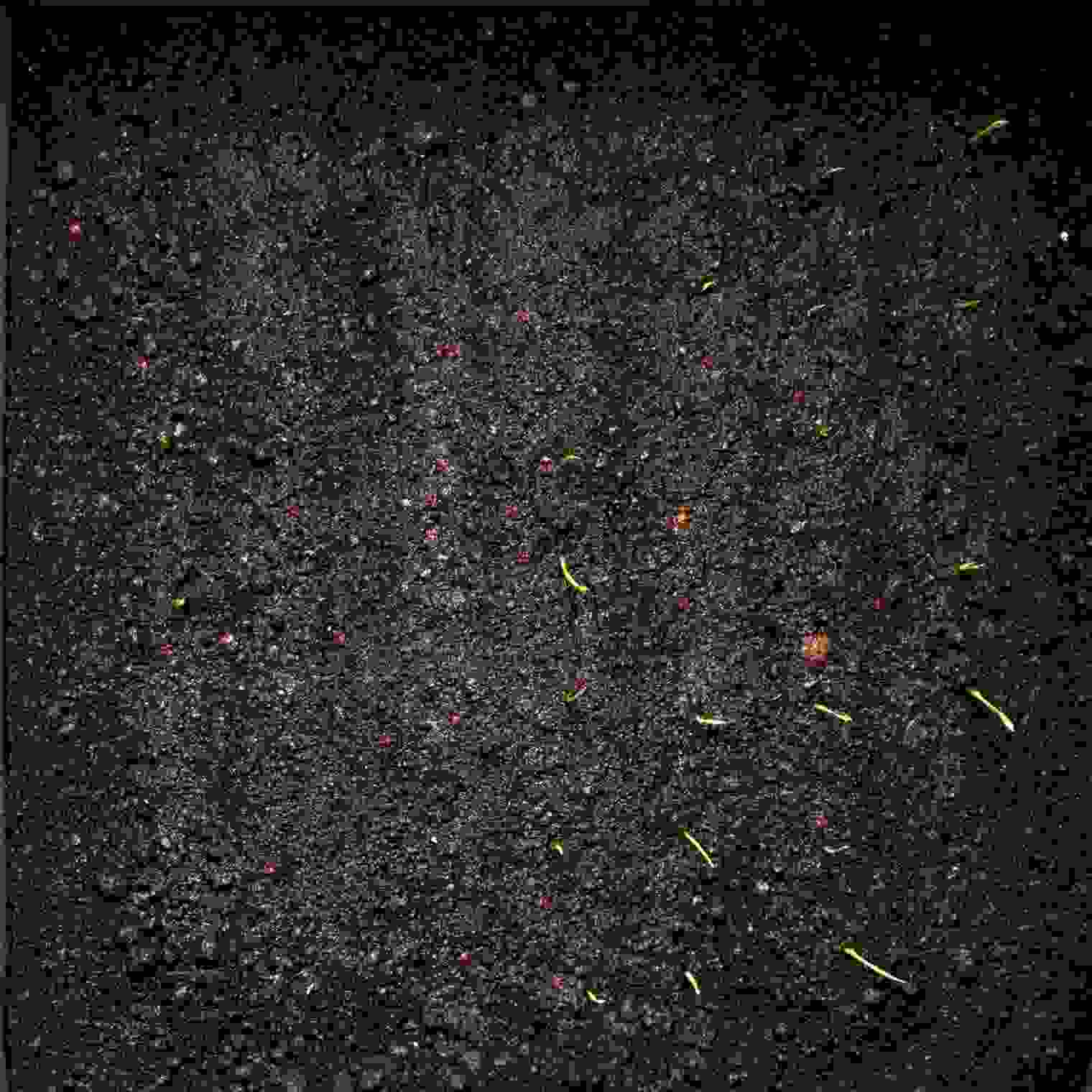

Supplement: Supplementary file 3 [file DataSheet3.zip › train1/2090-2024-3-20-3-10-47.JPG]

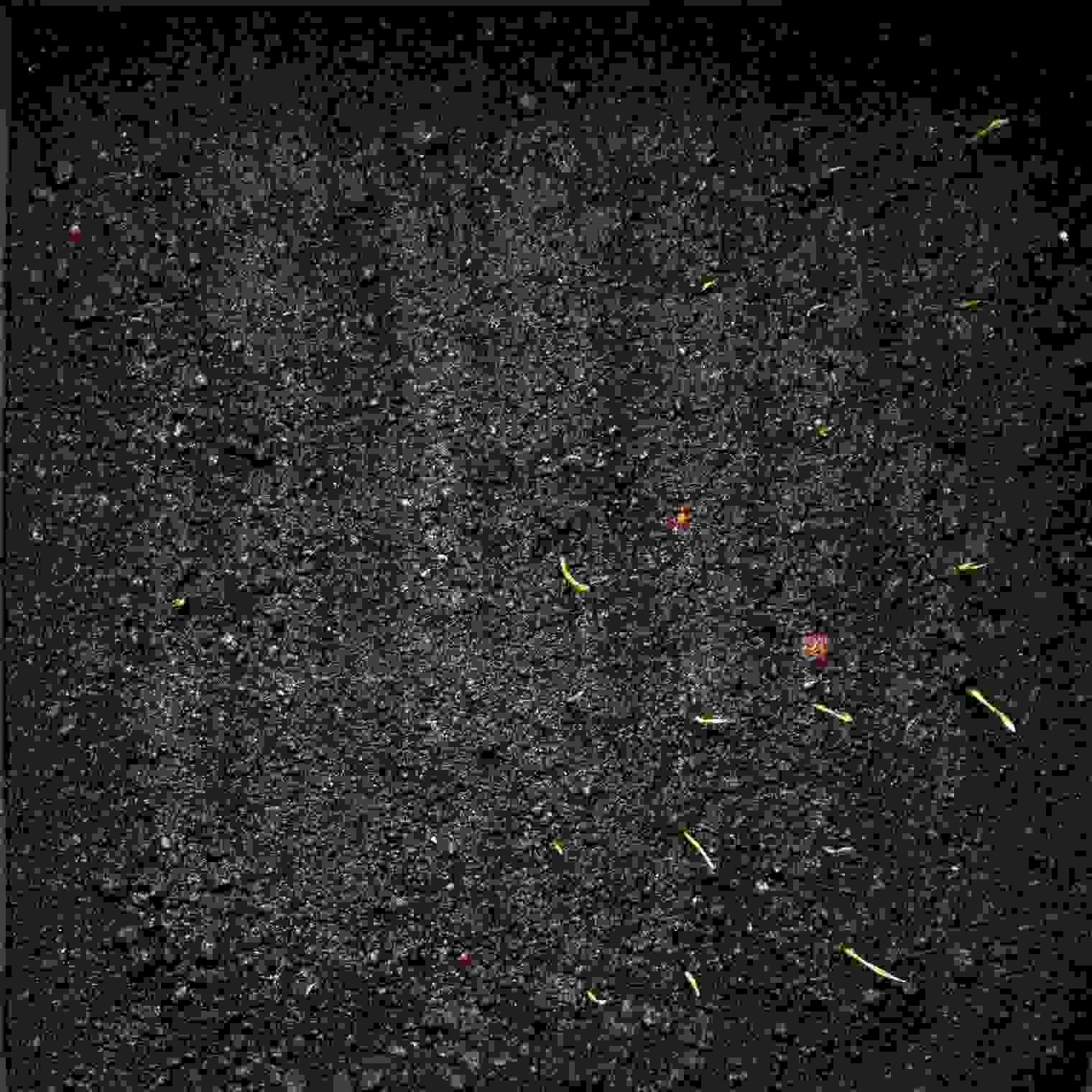

Supplement: Supplementary file 3 [file DataSheet3.zip › train1/2090-2024-3-20-5-43-5.JPG]

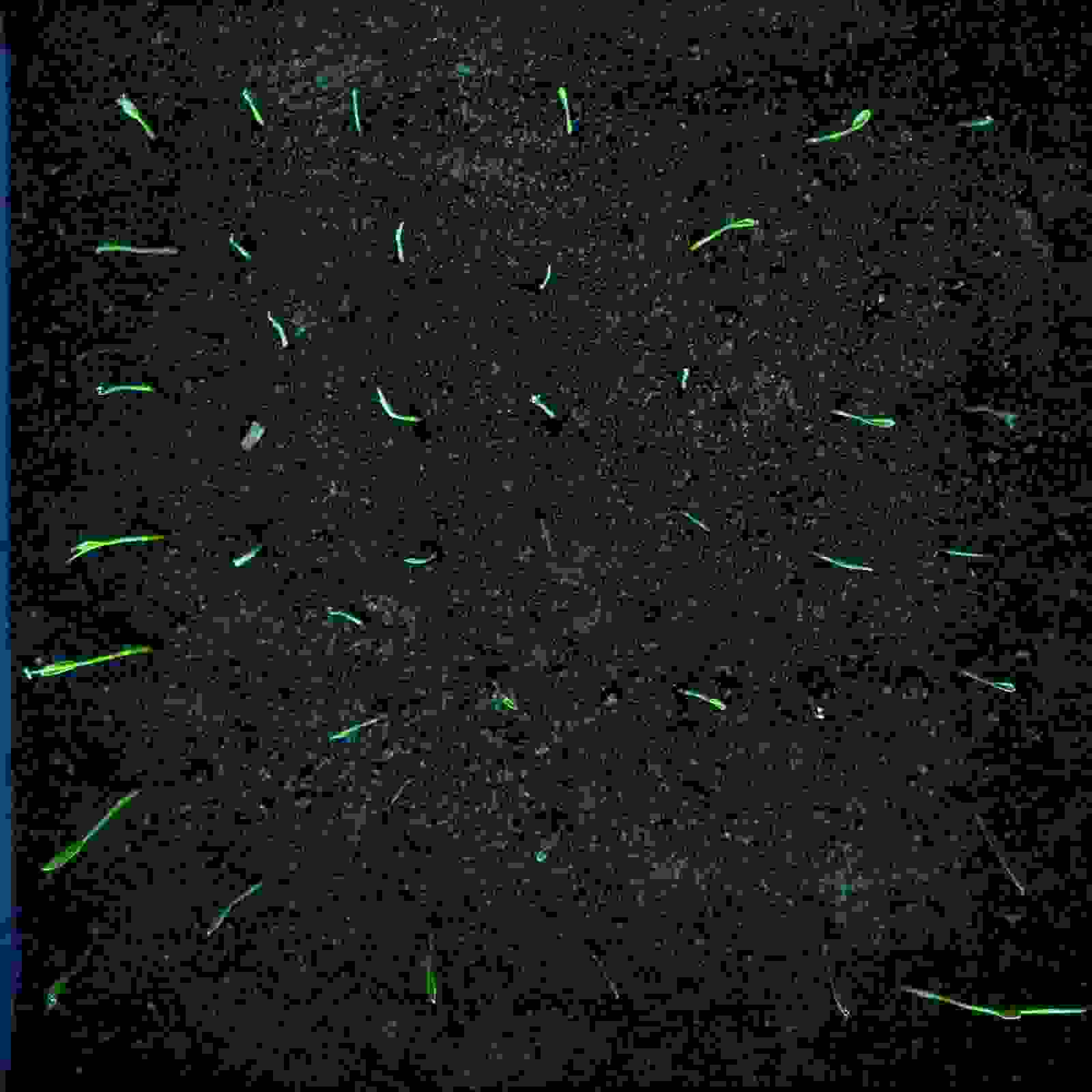

Supplement: Supplementary file 3 [file DataSheet3.zip › train1/3000-2024-4-1-21-0-33.JPG]

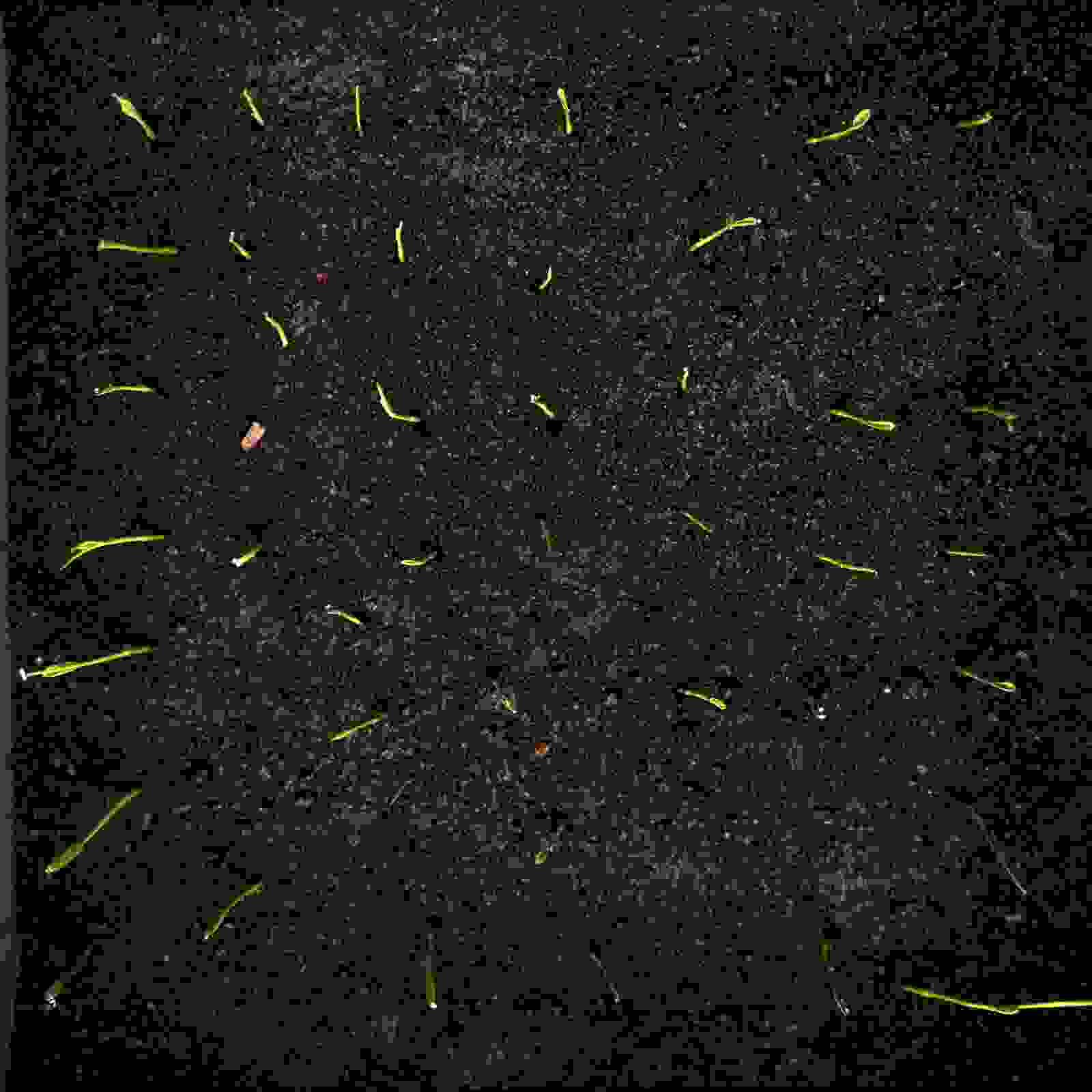

Supplement: Supplementary file 3 [file DataSheet3.zip › train1/3000-2024-4-1-23-24-49.JPG]

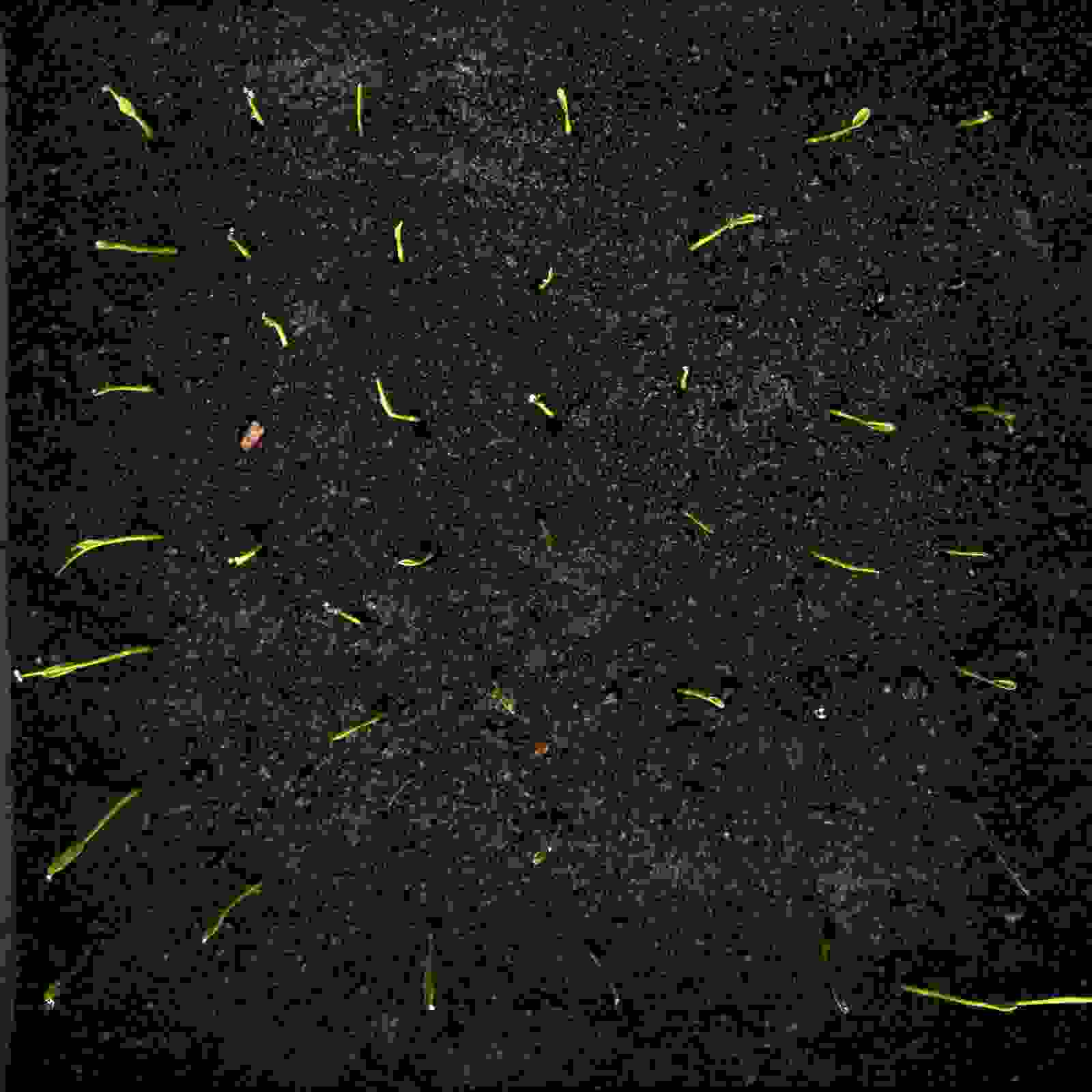

Supplement: Supplementary file 3 [file DataSheet3.zip › train1/3000-2024-4-2-1-48-50.JPG]

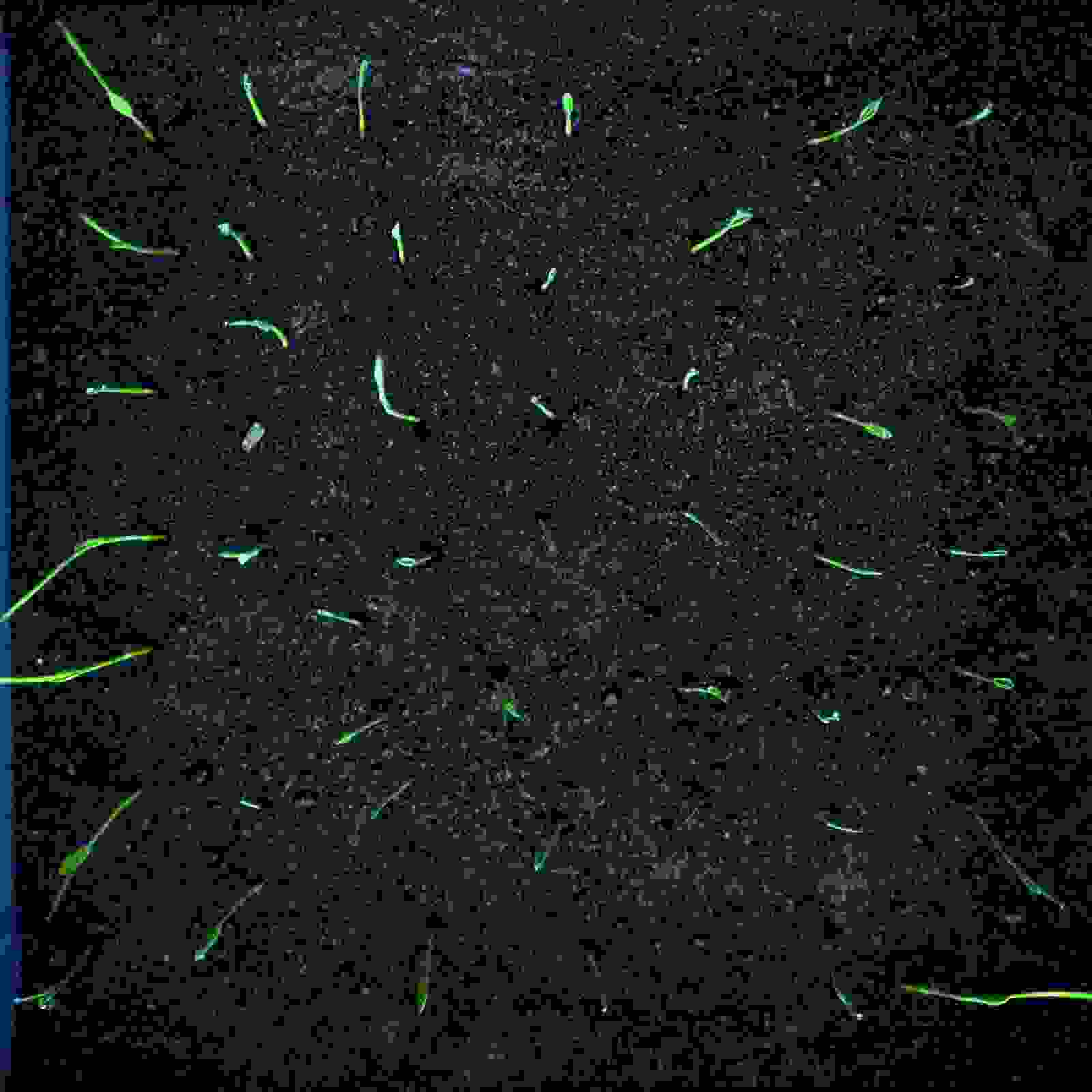

Supplement: Supplementary file 3 [file DataSheet3.zip › train1/3000-2024-4-2-16-14-6.JPG]

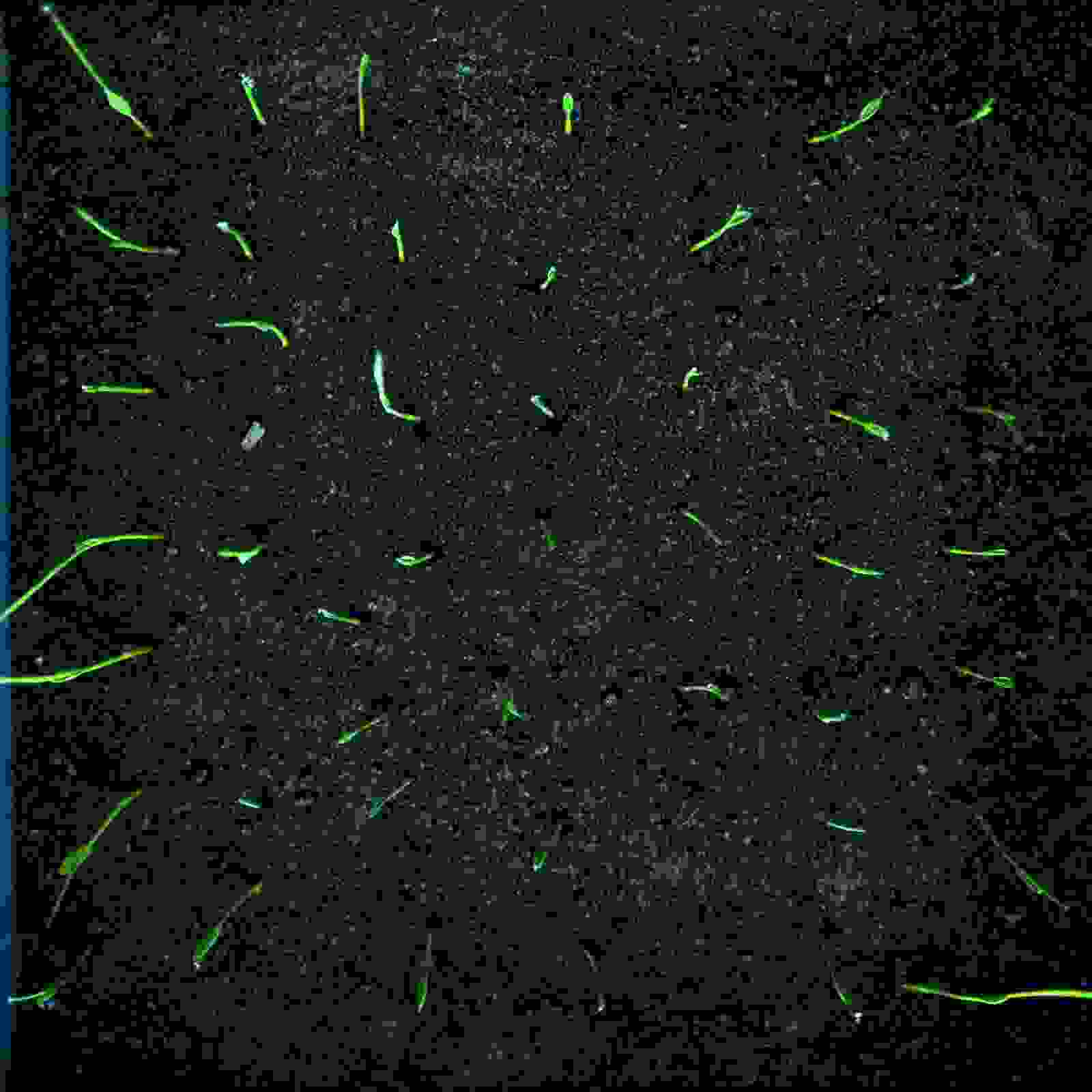

Supplement: Supplementary file 3 [file DataSheet3.zip › train1/3000-2024-4-2-18-38-54.JPG]

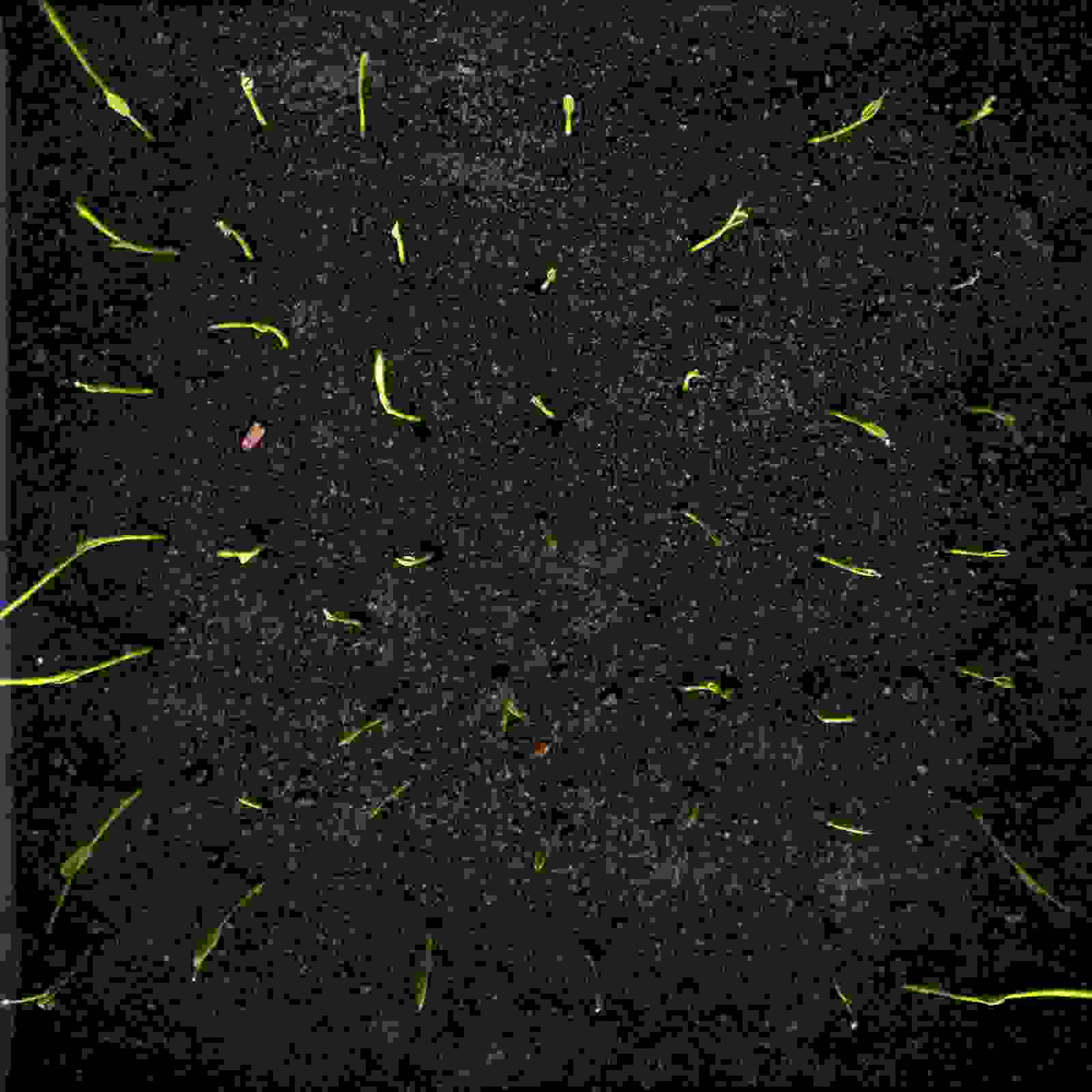

Supplement: Supplementary file 3 [file DataSheet3.zip › train1/3000-2024-4-2-21-2-40.JPG]

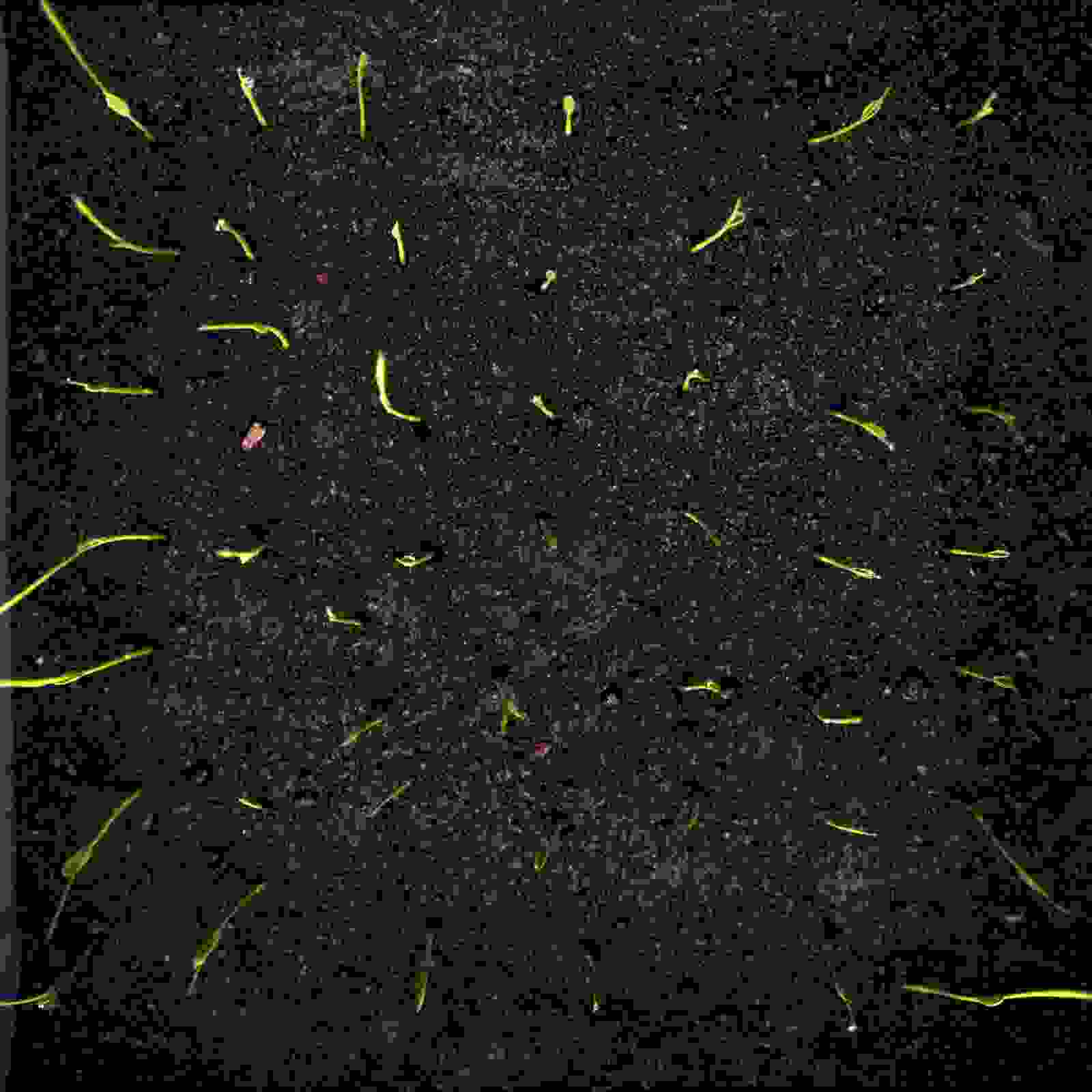

Supplement: Supplementary file 3 [file DataSheet3.zip › train1/3000-2024-4-2-23-27-40.JPG]

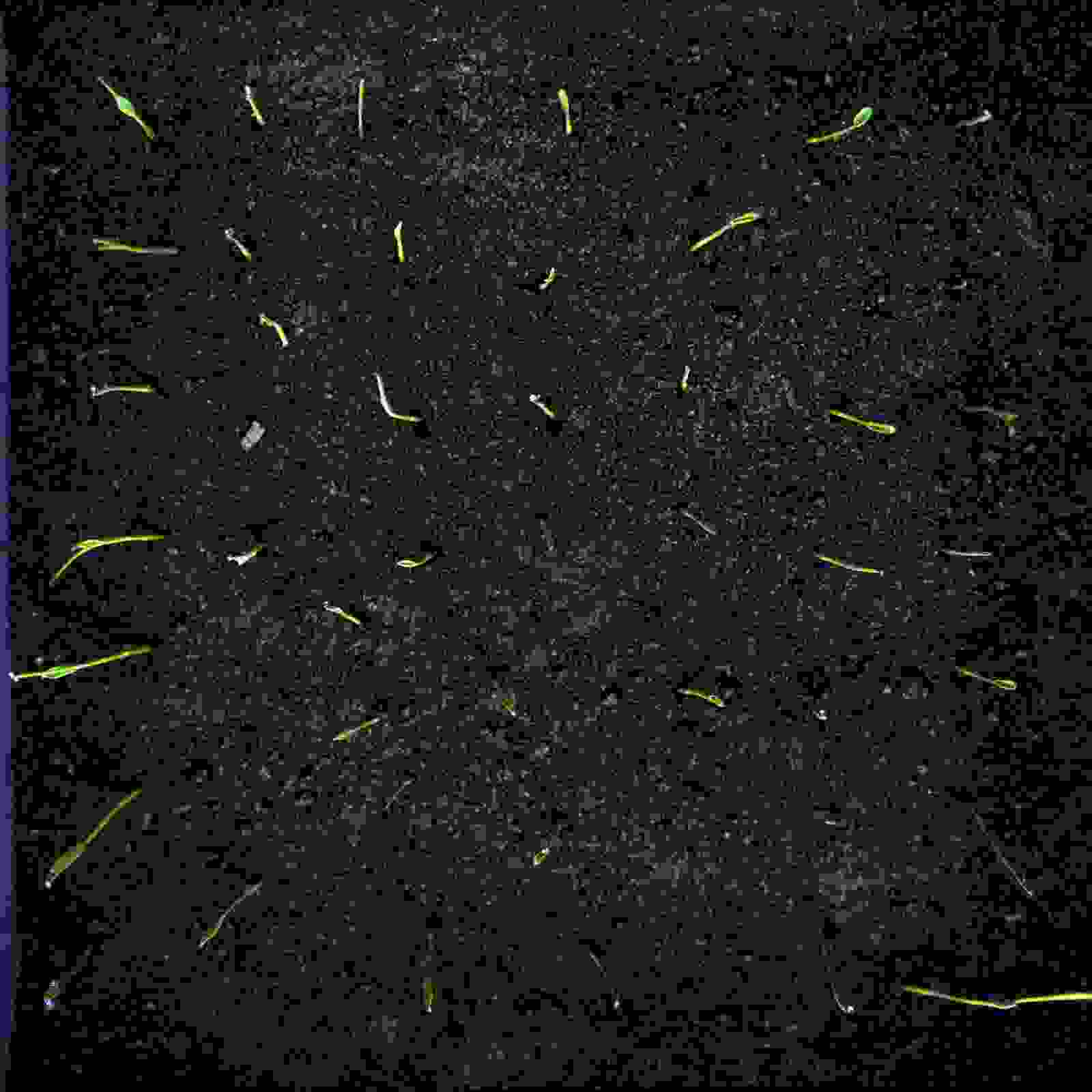

Supplement: Supplementary file 3 [file DataSheet3.zip › train1/3000-2024-4-2-4-13-0.JPG]

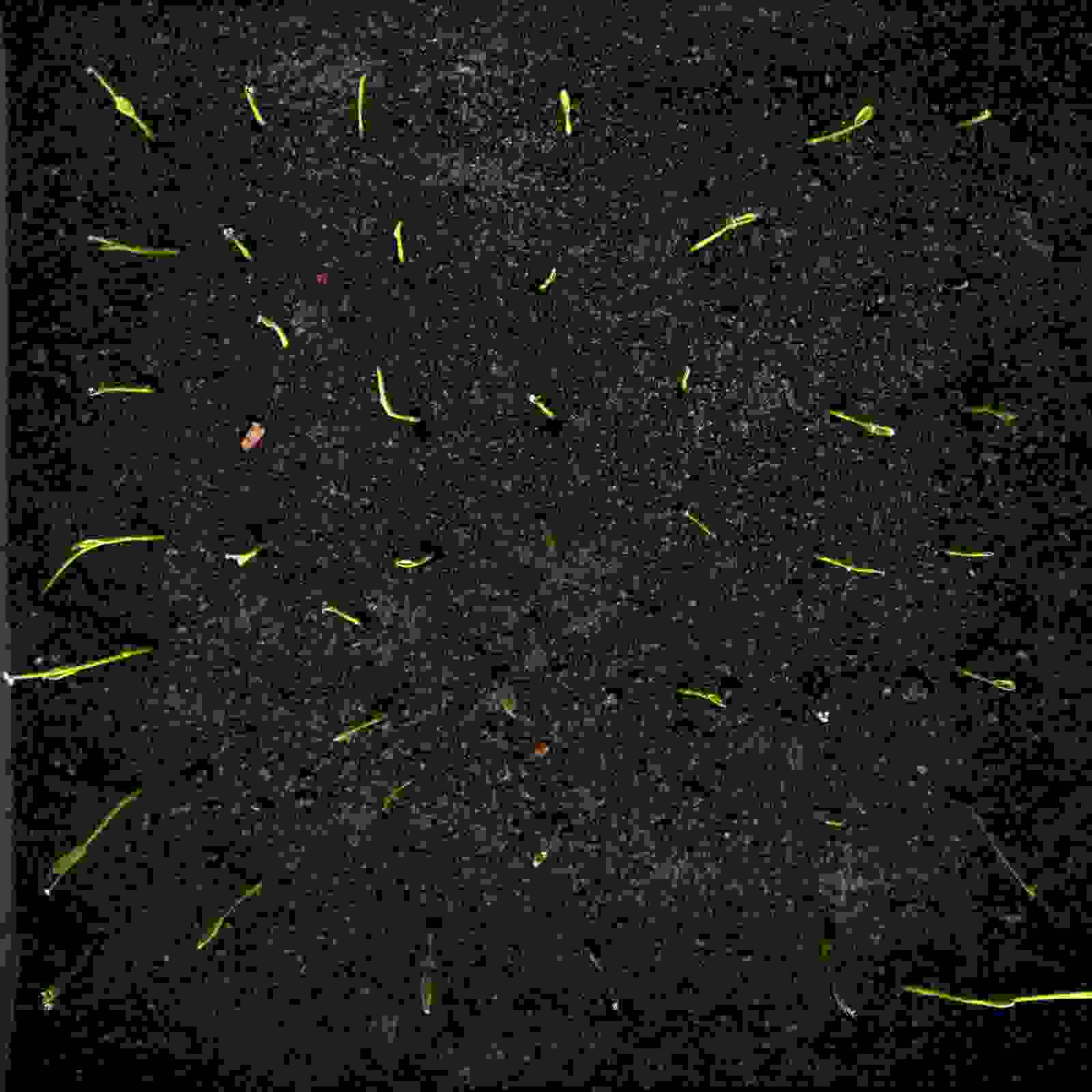

Supplement: Supplementary file 3 [file DataSheet3.zip › train1/3000-2024-4-2-6-37-4.JPG]

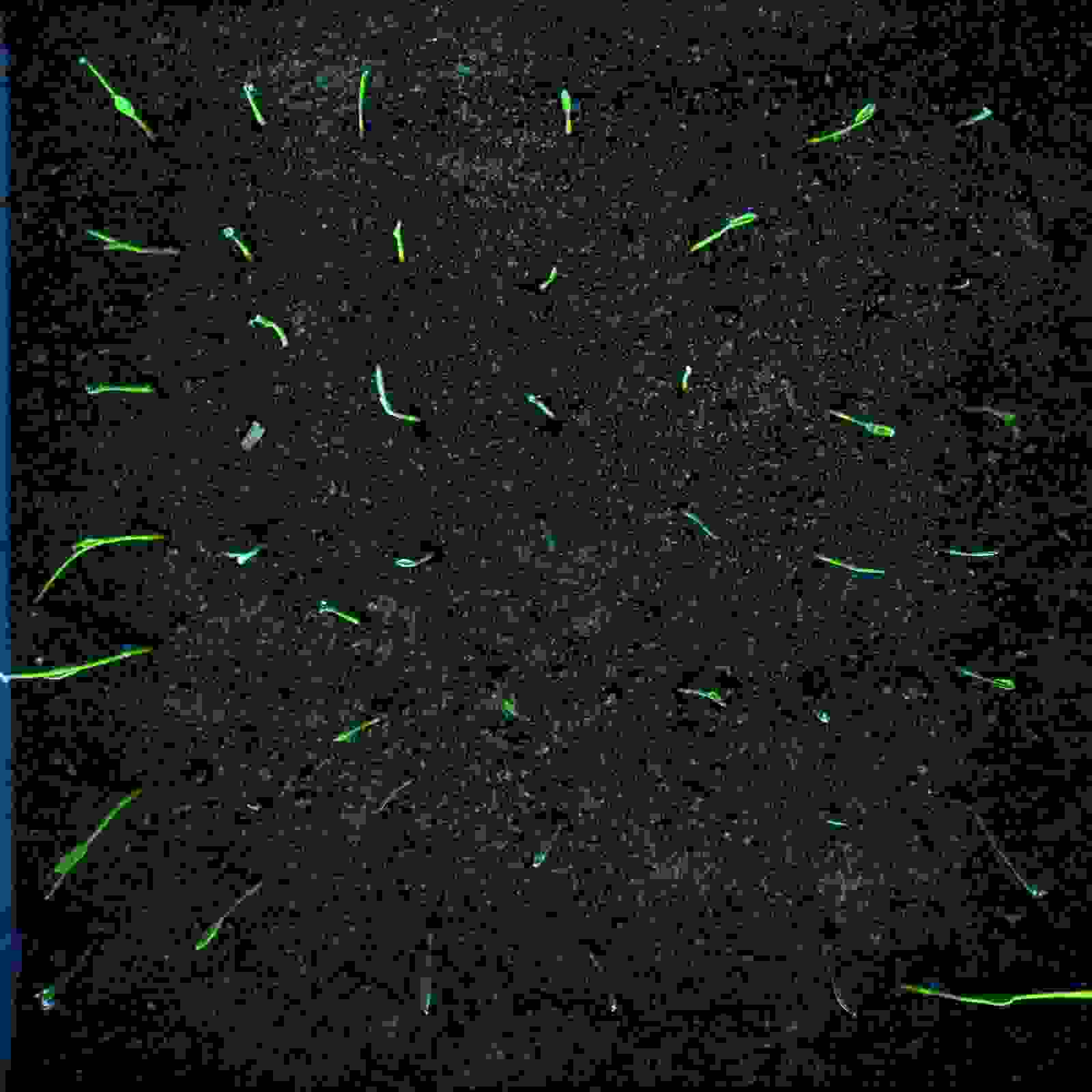

Supplement: Supplementary file 3 [file DataSheet3.zip › train1/3000-2024-4-2-9-1-16.JPG]

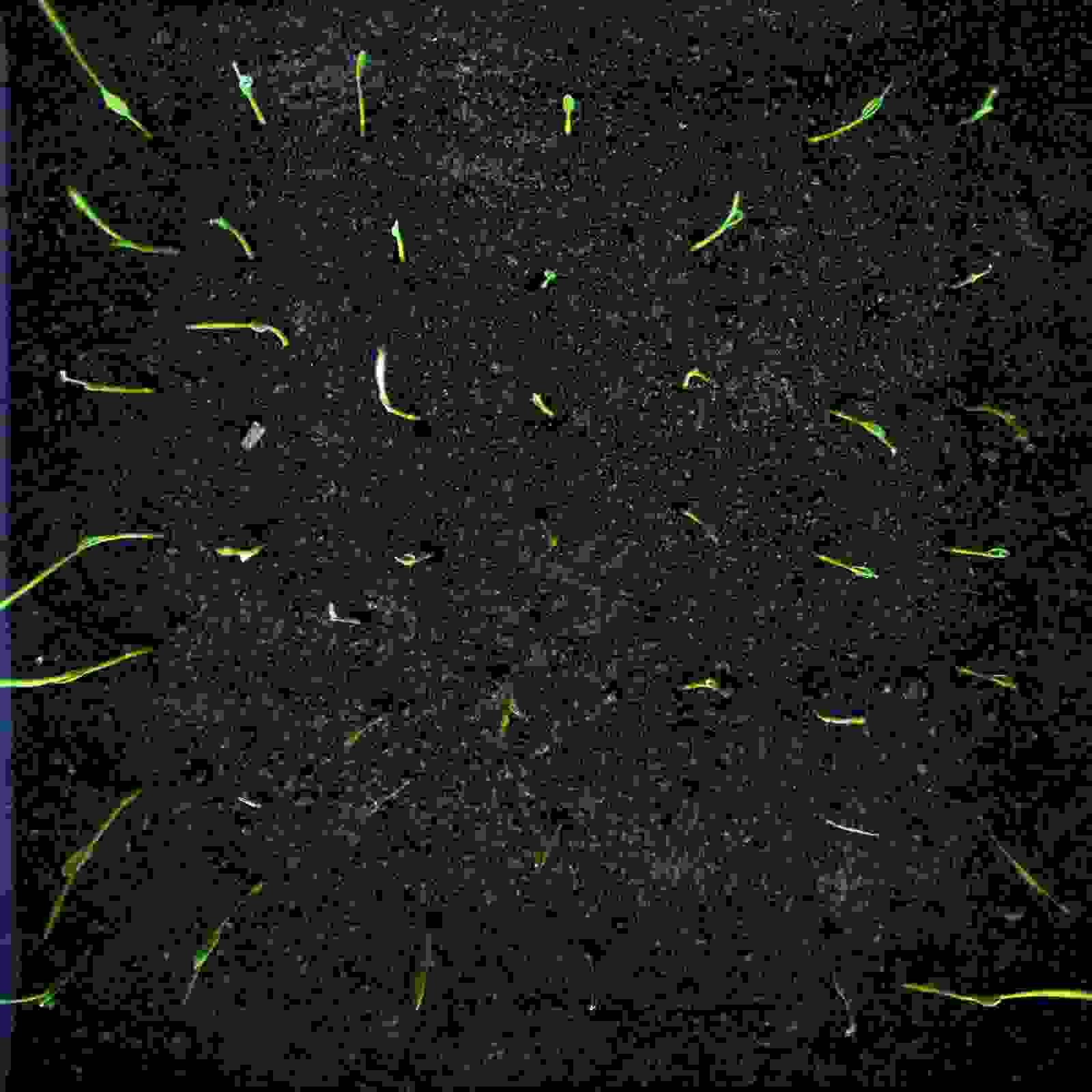

Supplement: Supplementary file 3 [file DataSheet3.zip › train1/3000-2024-4-3-1-51-18.JPG]

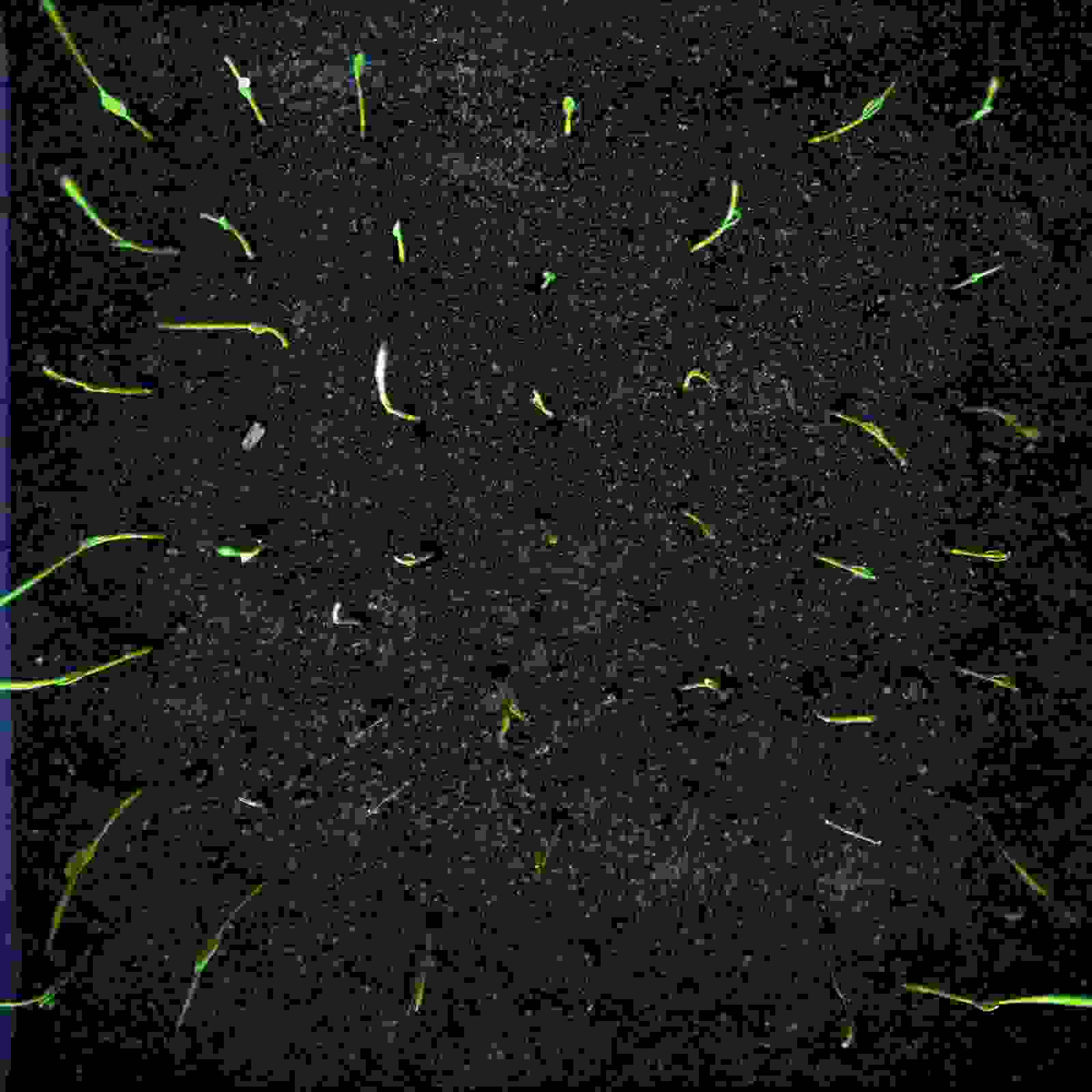

Supplement: Supplementary file 3 [file DataSheet3.zip › train1/3000-2024-4-3-6-39-41.JPG]

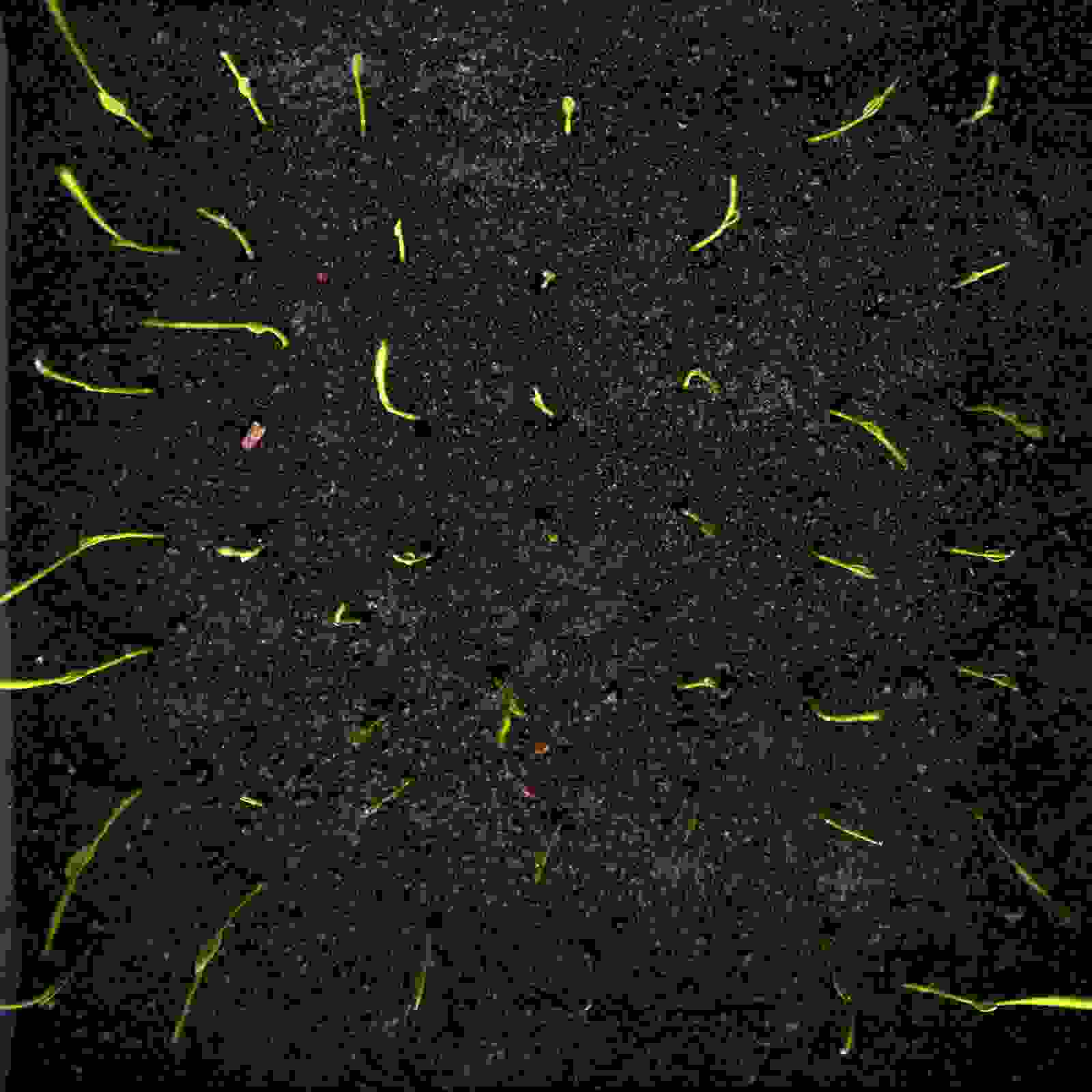

Supplement: Supplementary file 3 [file DataSheet3.zip › train1/3000-2024-4-3-9-4-5.JPG]

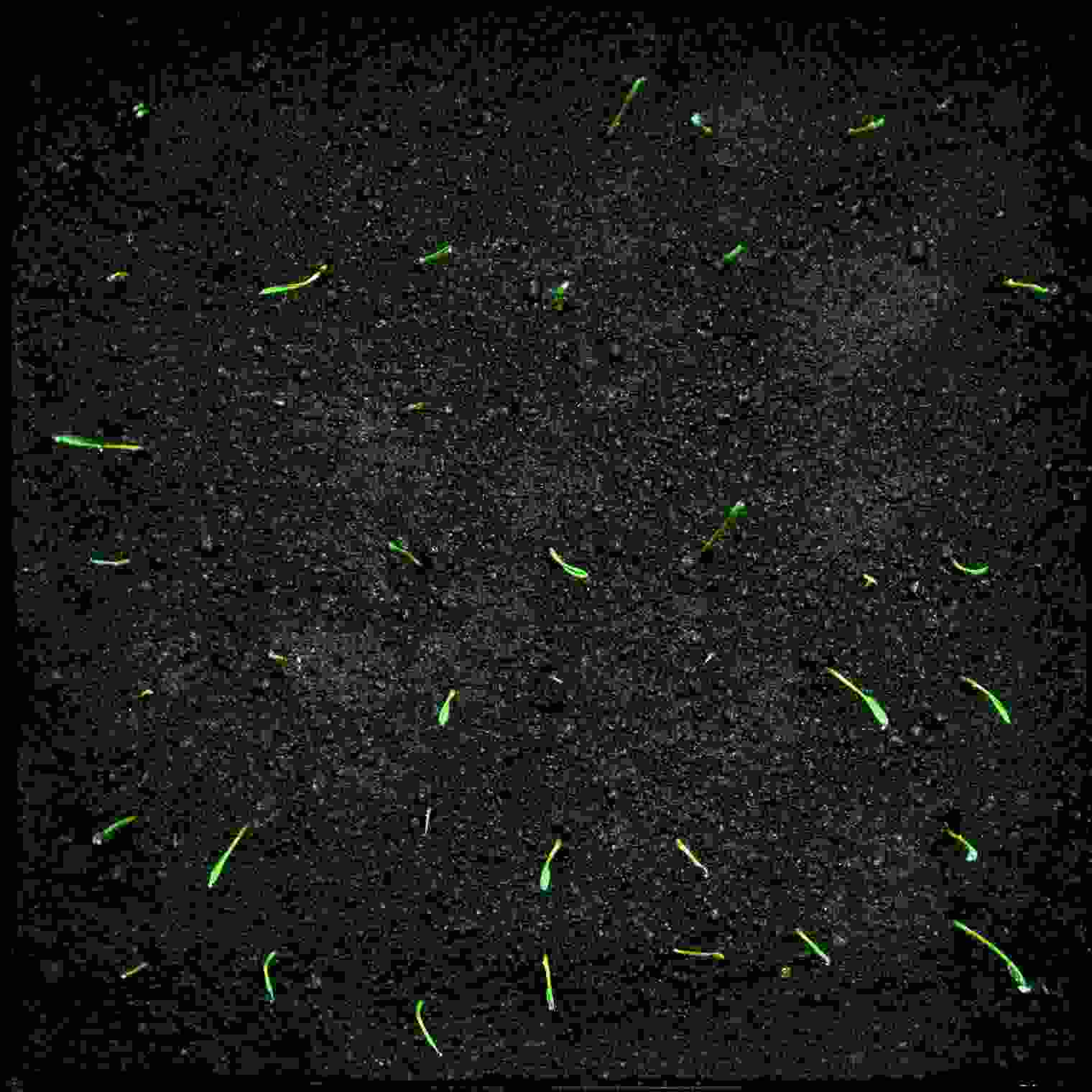

Supplement: Supplementary file 3 [file DataSheet3.zip › train1/500-2024-3-18-17-51-33.JPG]

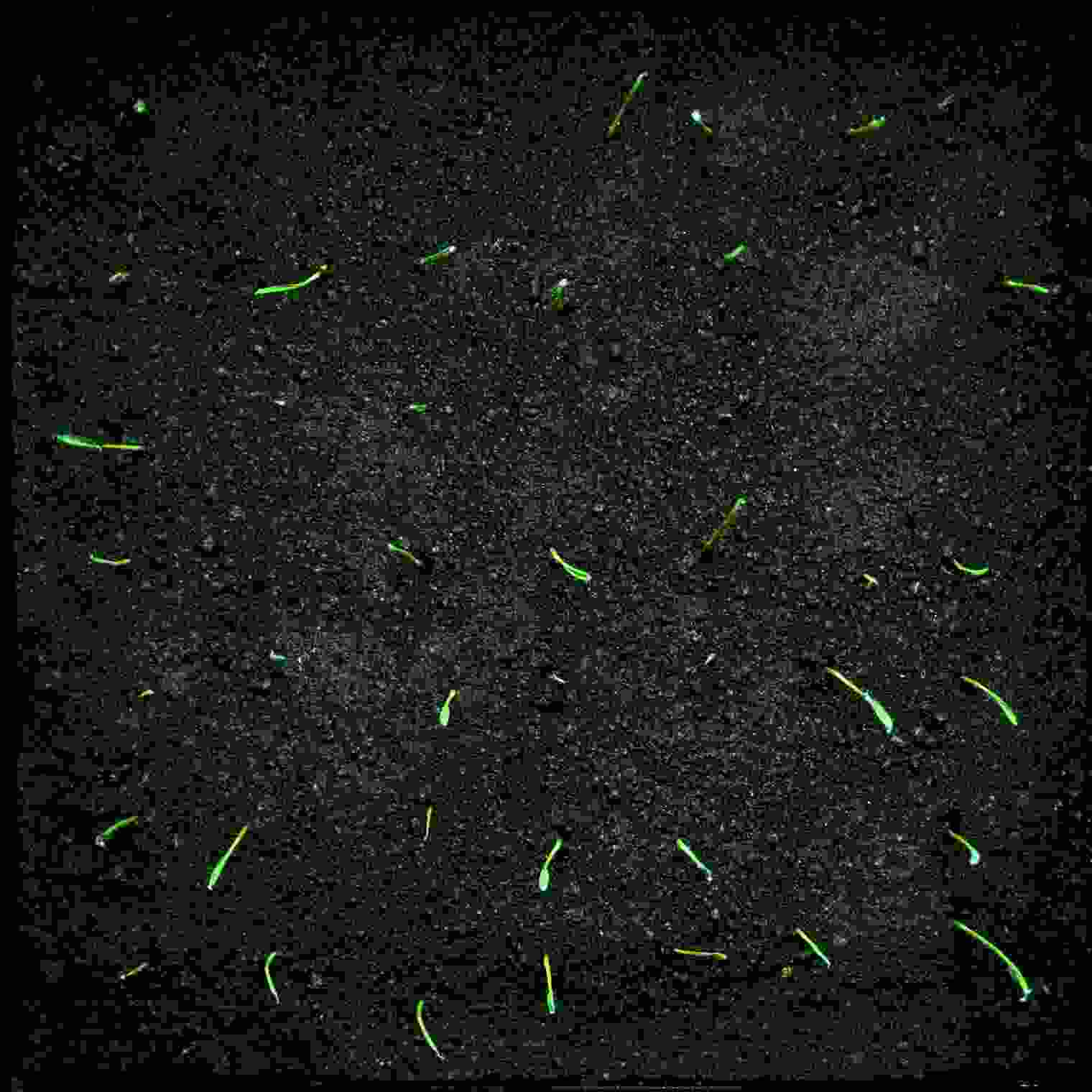

Supplement: Supplementary file 3 [file DataSheet3.zip › train1/500-2024-3-18-20-39-4.JPG]

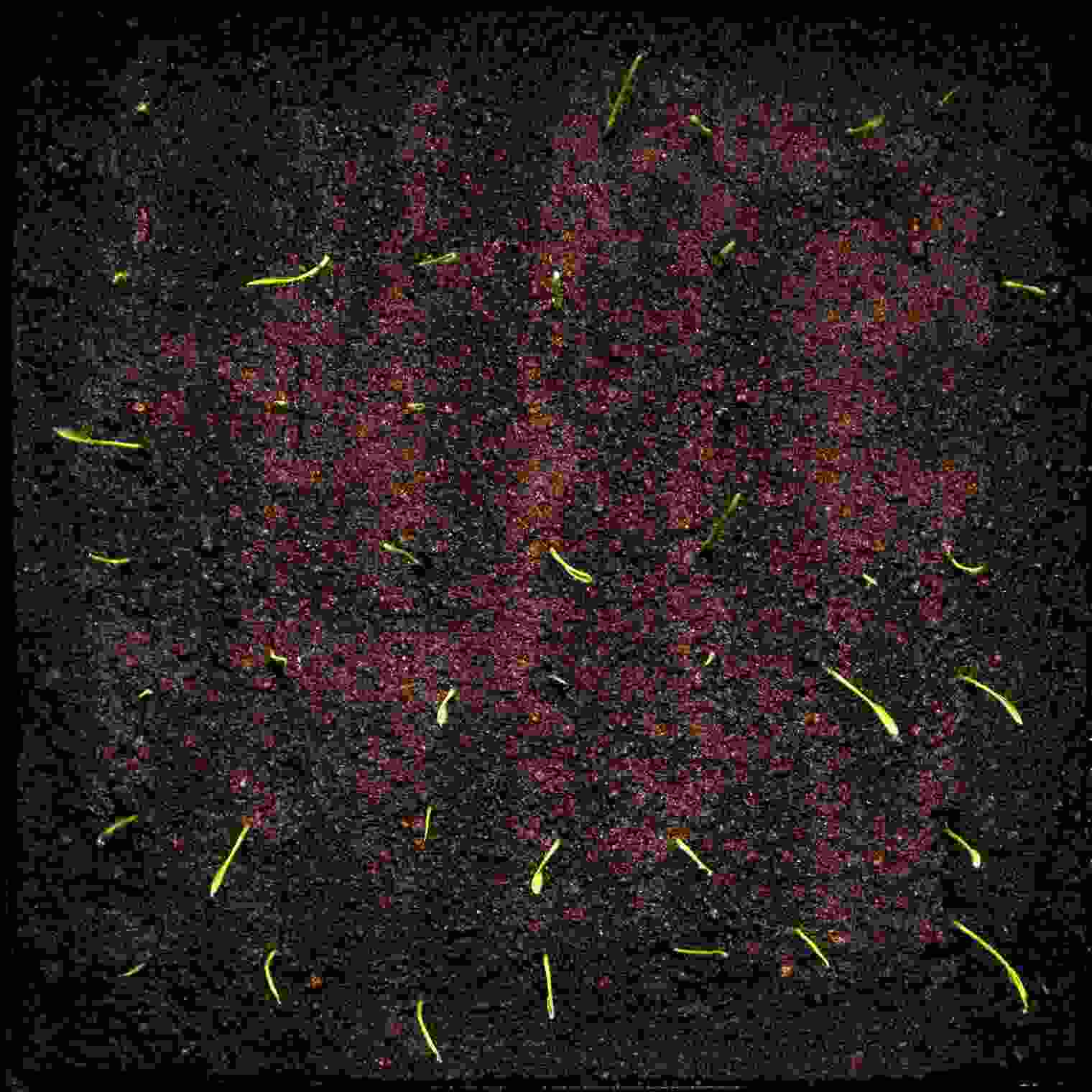

Supplement: Supplementary file 3 [file DataSheet3.zip › train1/500-2024-3-18-23-27-7.JPG]

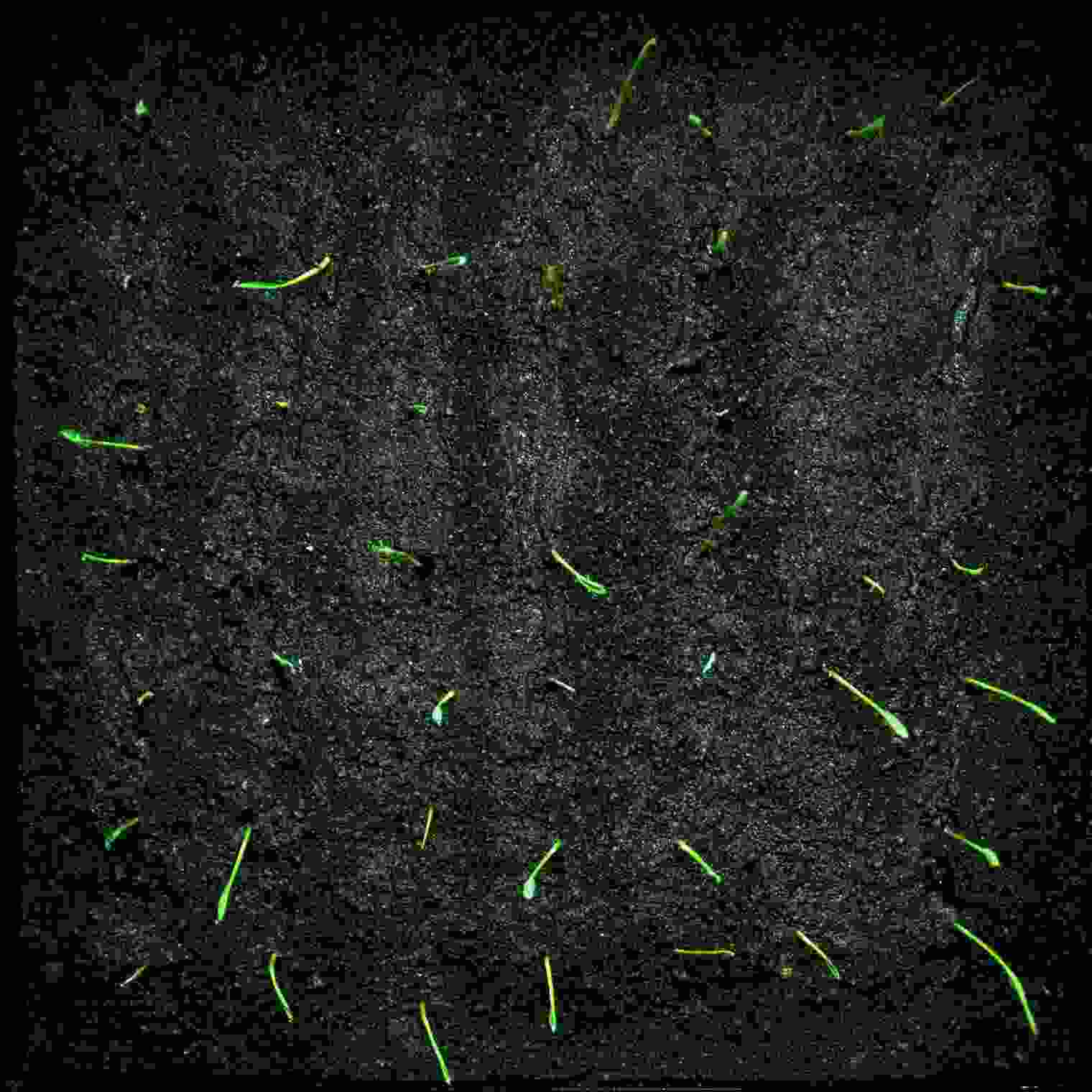

Supplement: Supplementary file 3 [file DataSheet3.zip › train1/500-2024-3-19-10-37-31.JPG]

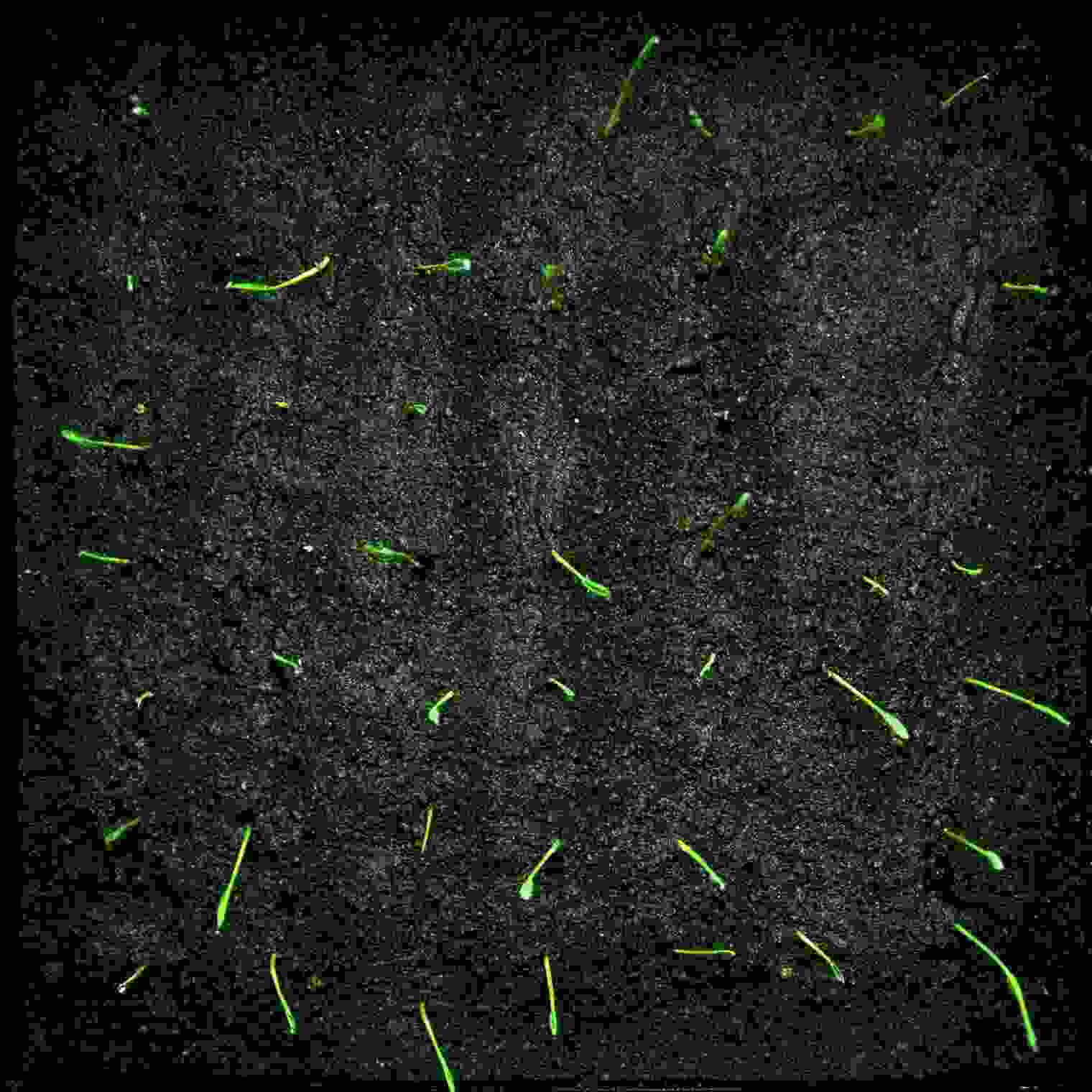

Supplement: Supplementary file 3 [file DataSheet3.zip › train1/500-2024-3-19-16-13-6.JPG]

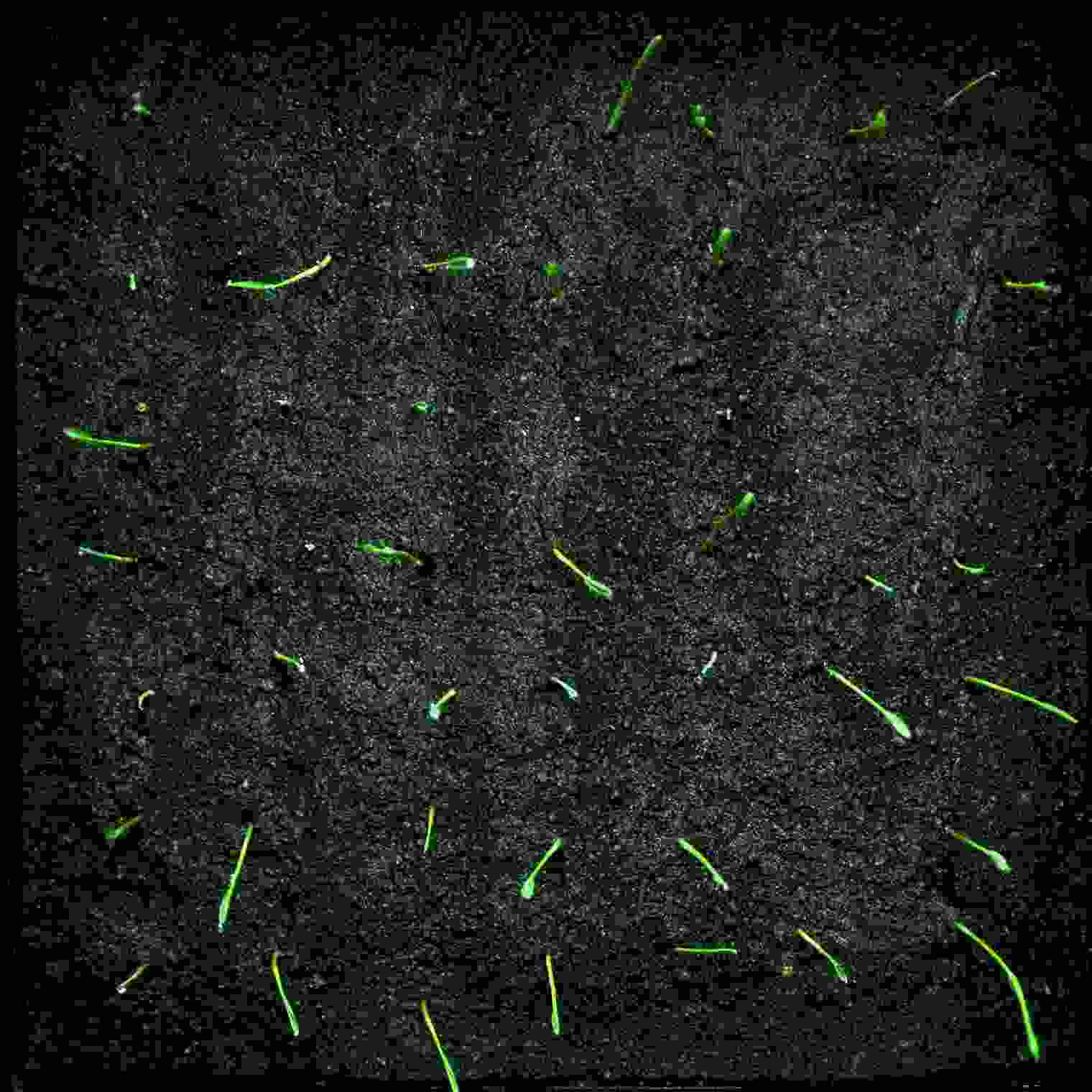

Supplement: Supplementary file 3 [file DataSheet3.zip › train1/500-2024-3-19-19-0-4.JPG]

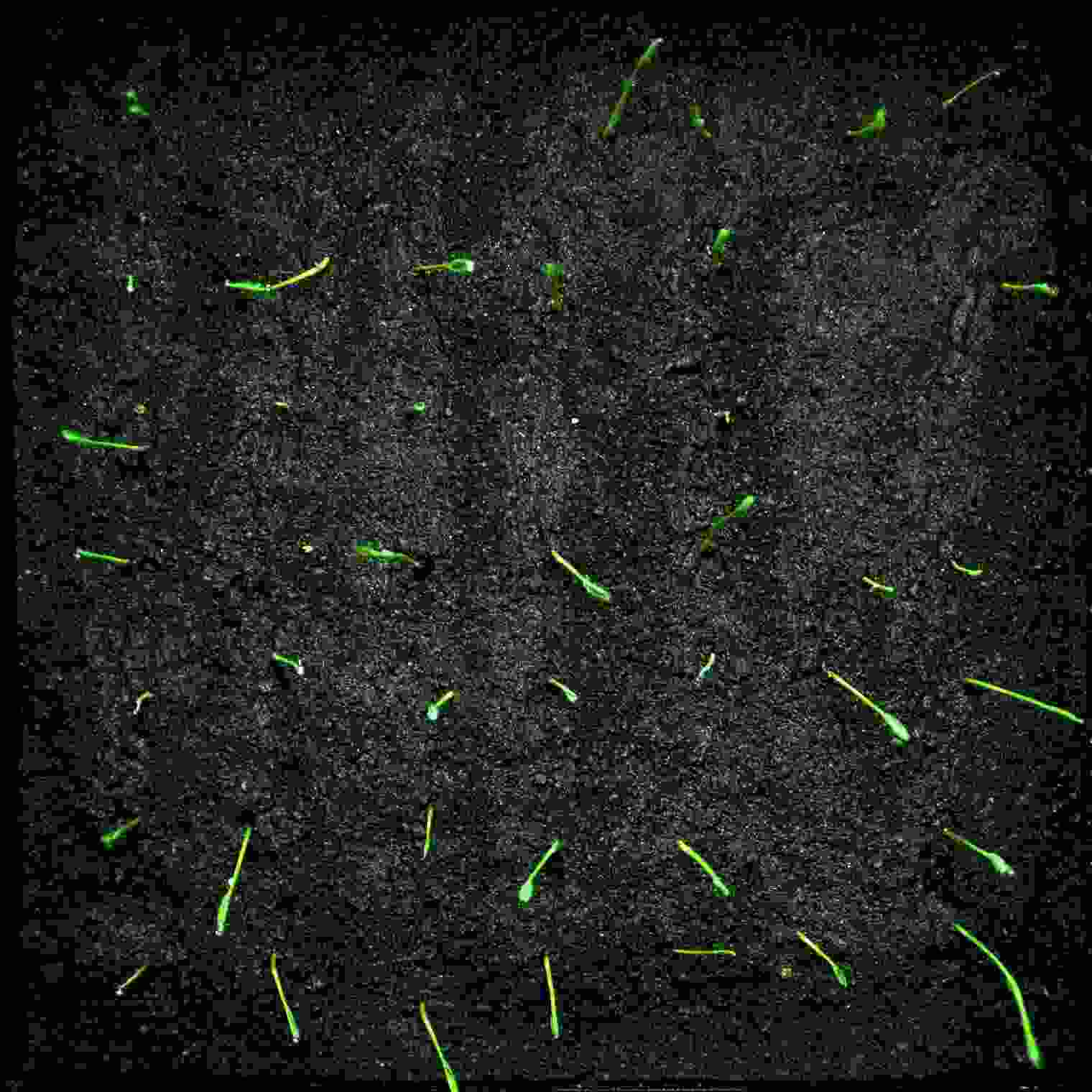

Supplement: Supplementary file 3 [file DataSheet3.zip › train1/500-2024-3-19-21-48-6.JPG]

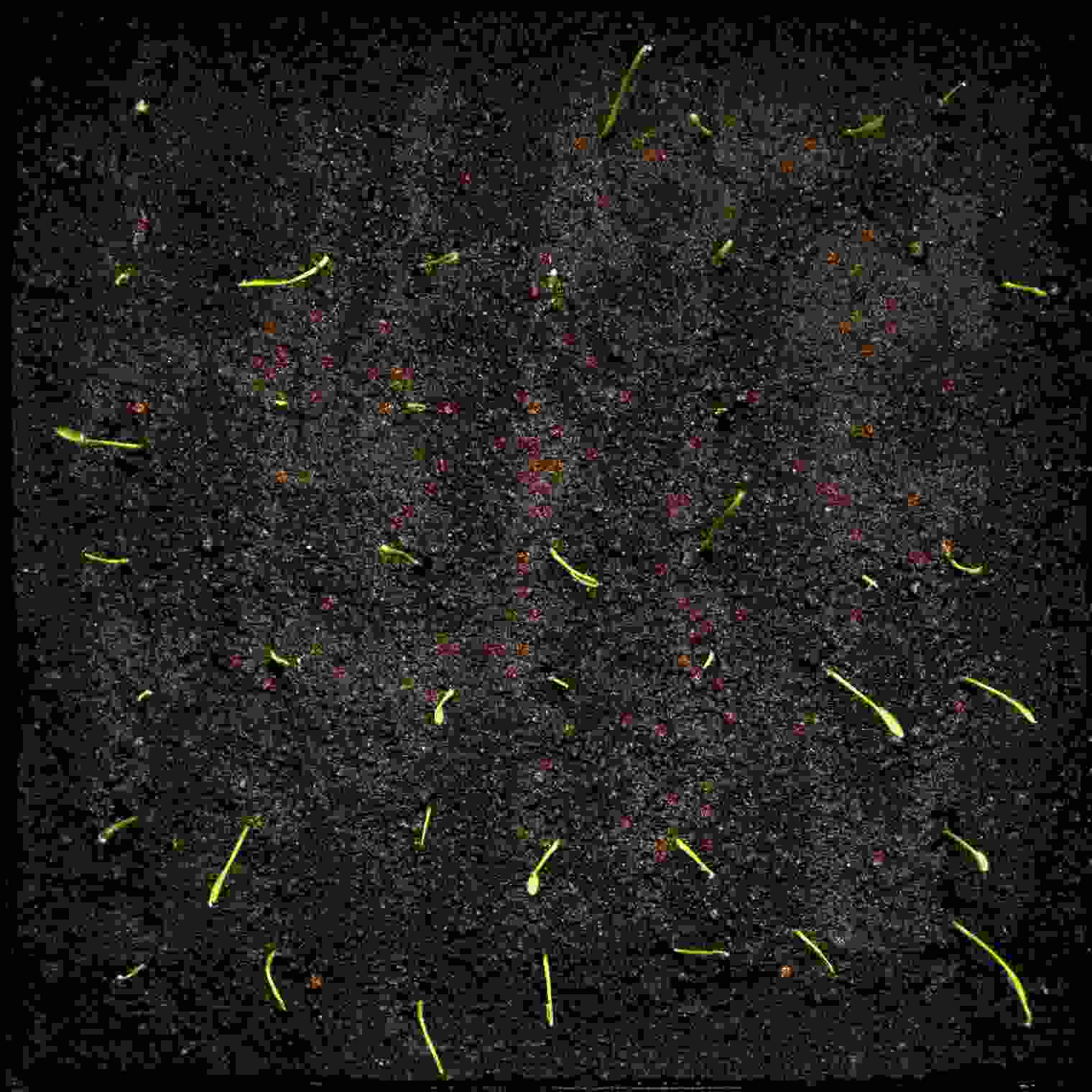

Supplement: Supplementary file 3 [file DataSheet3.zip › train1/500-2024-3-19-5-1-46.JPG]

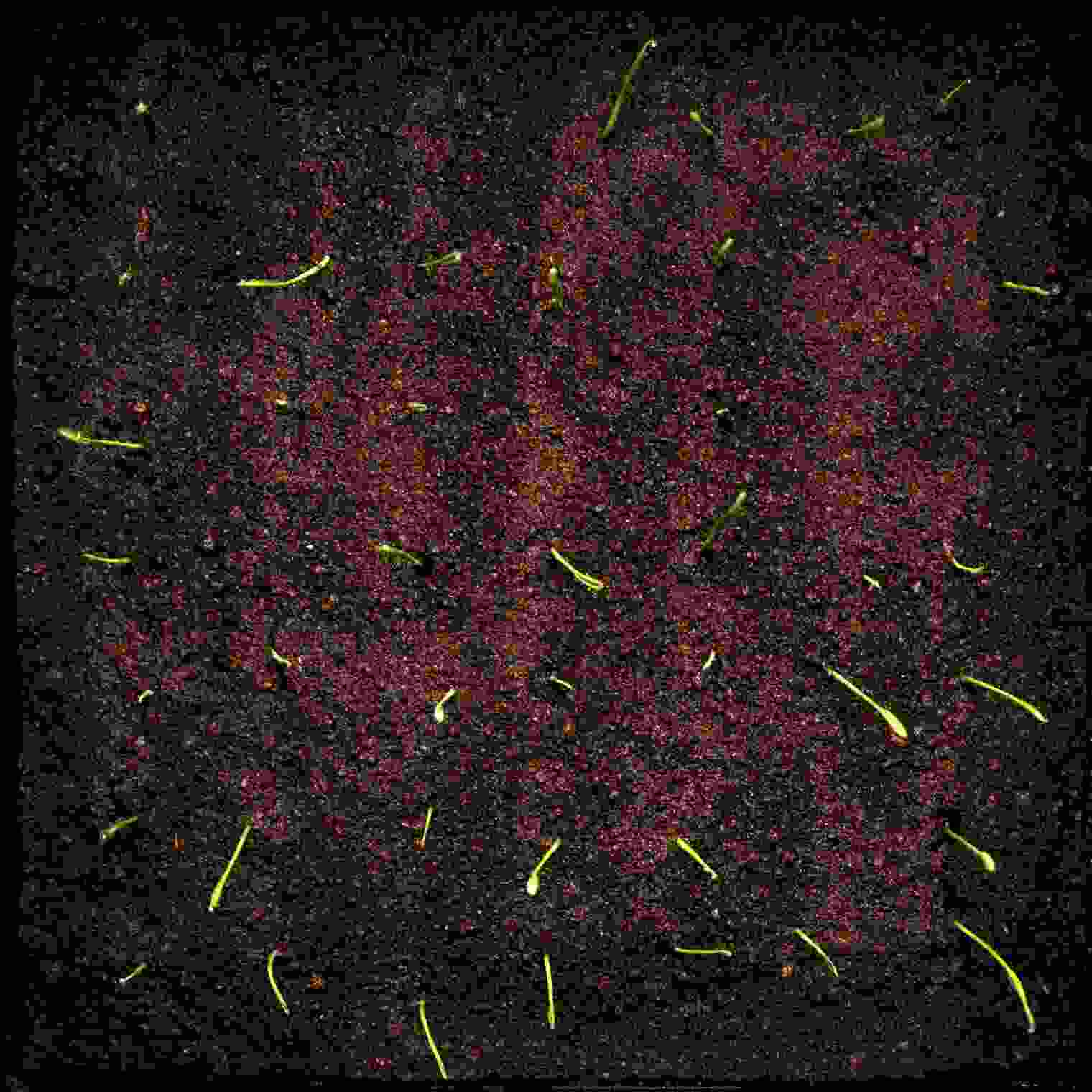

Supplement: Supplementary file 3 [file DataSheet3.zip › train1/500-2024-3-19-7-49-34.JPG]

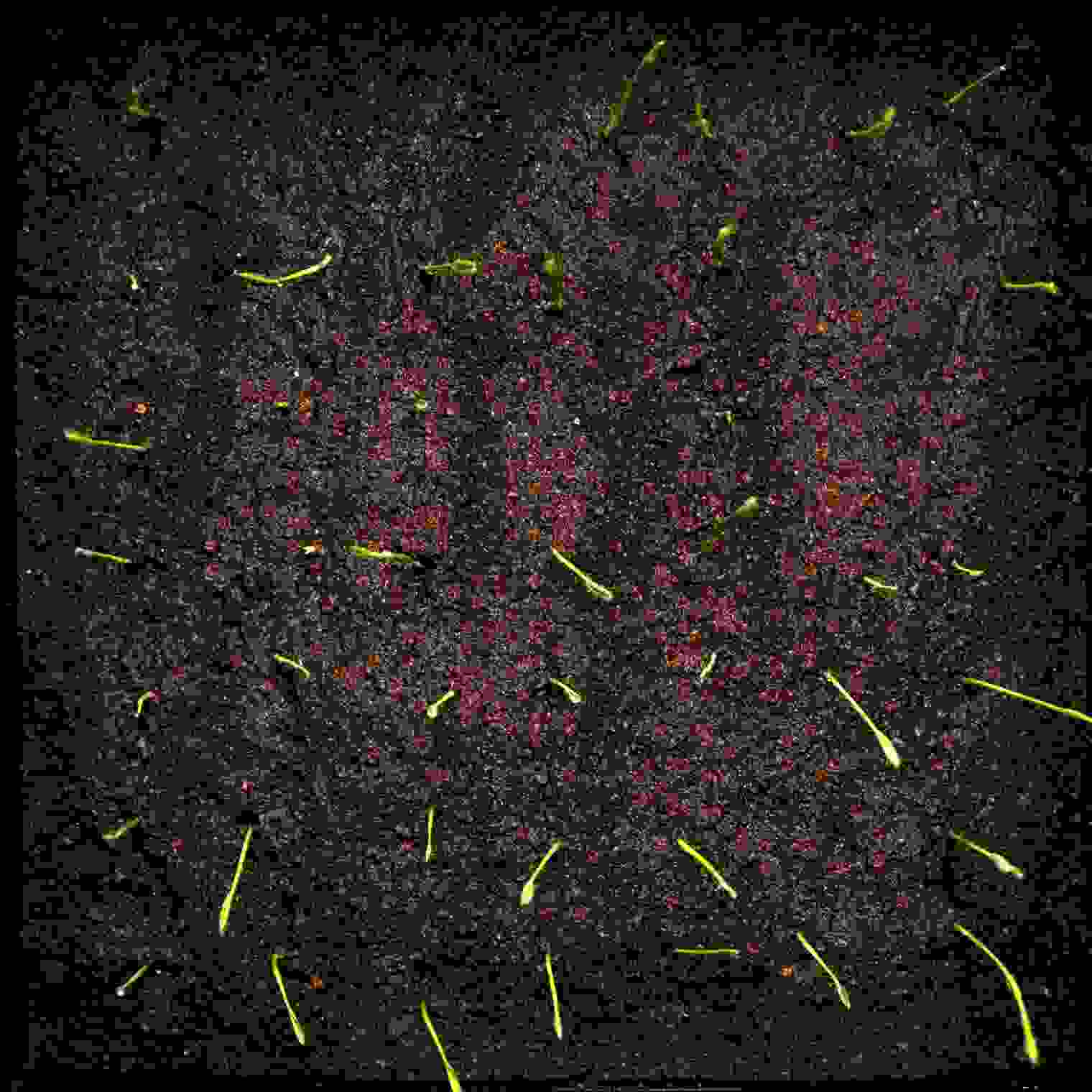

Supplement: Supplementary file 3 [file DataSheet3.zip › train1/500-2024-3-20-0-35-48.JPG]

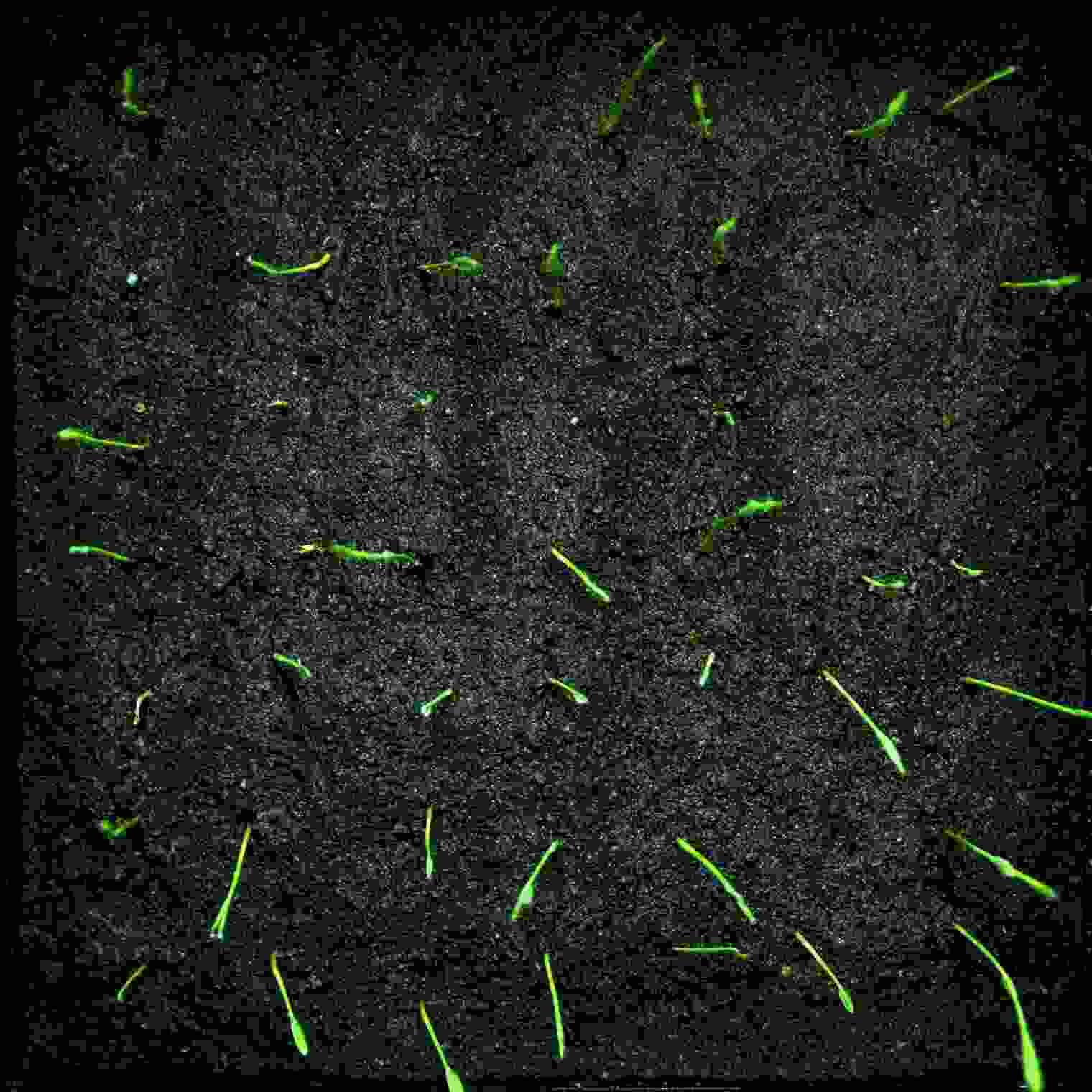

Supplement: Supplementary file 3 [file DataSheet3.zip › train1/500-2024-3-20-11-45-43.JPG]

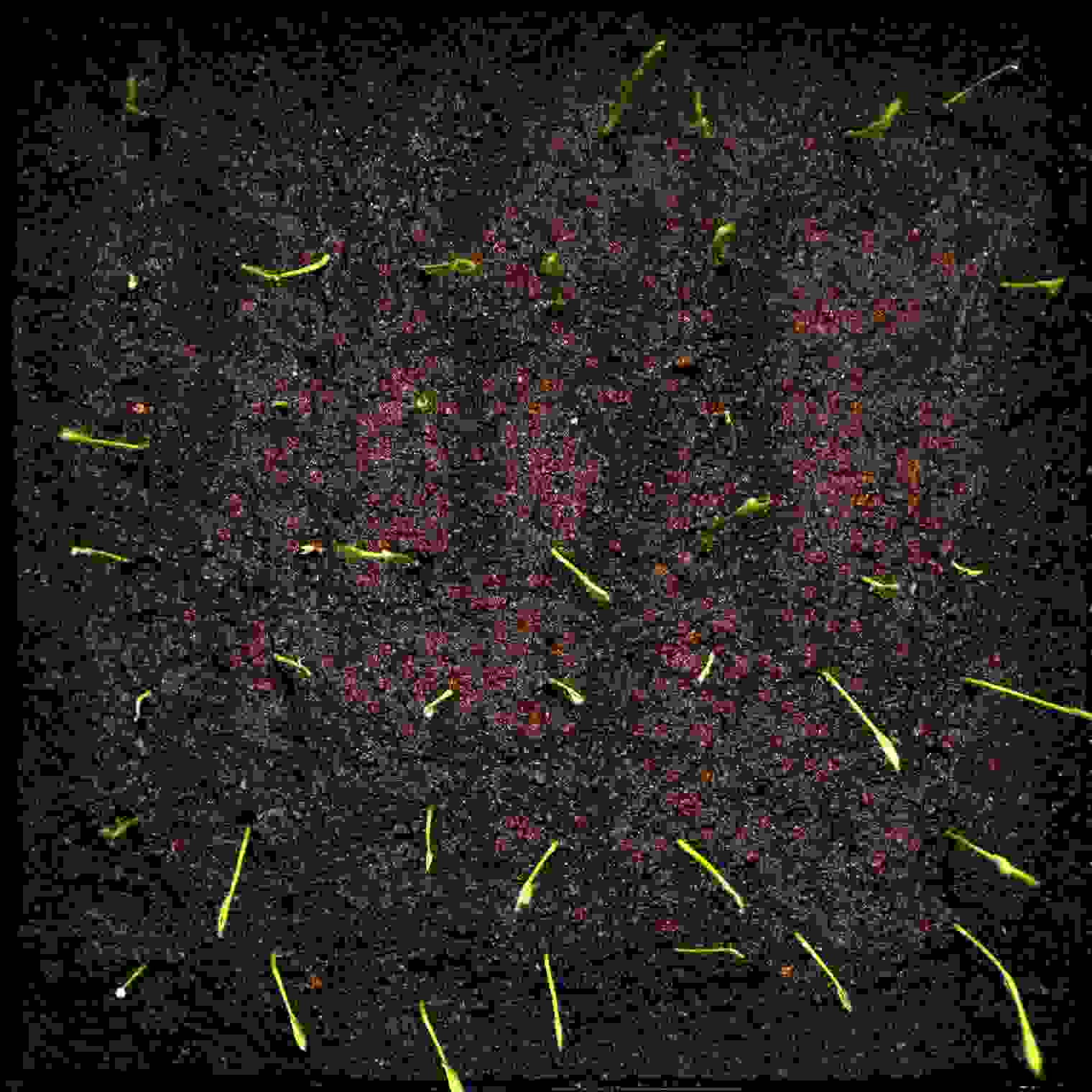

Supplement: Supplementary file 3 [file DataSheet3.zip › train1/500-2024-3-20-6-10-41.JPG]

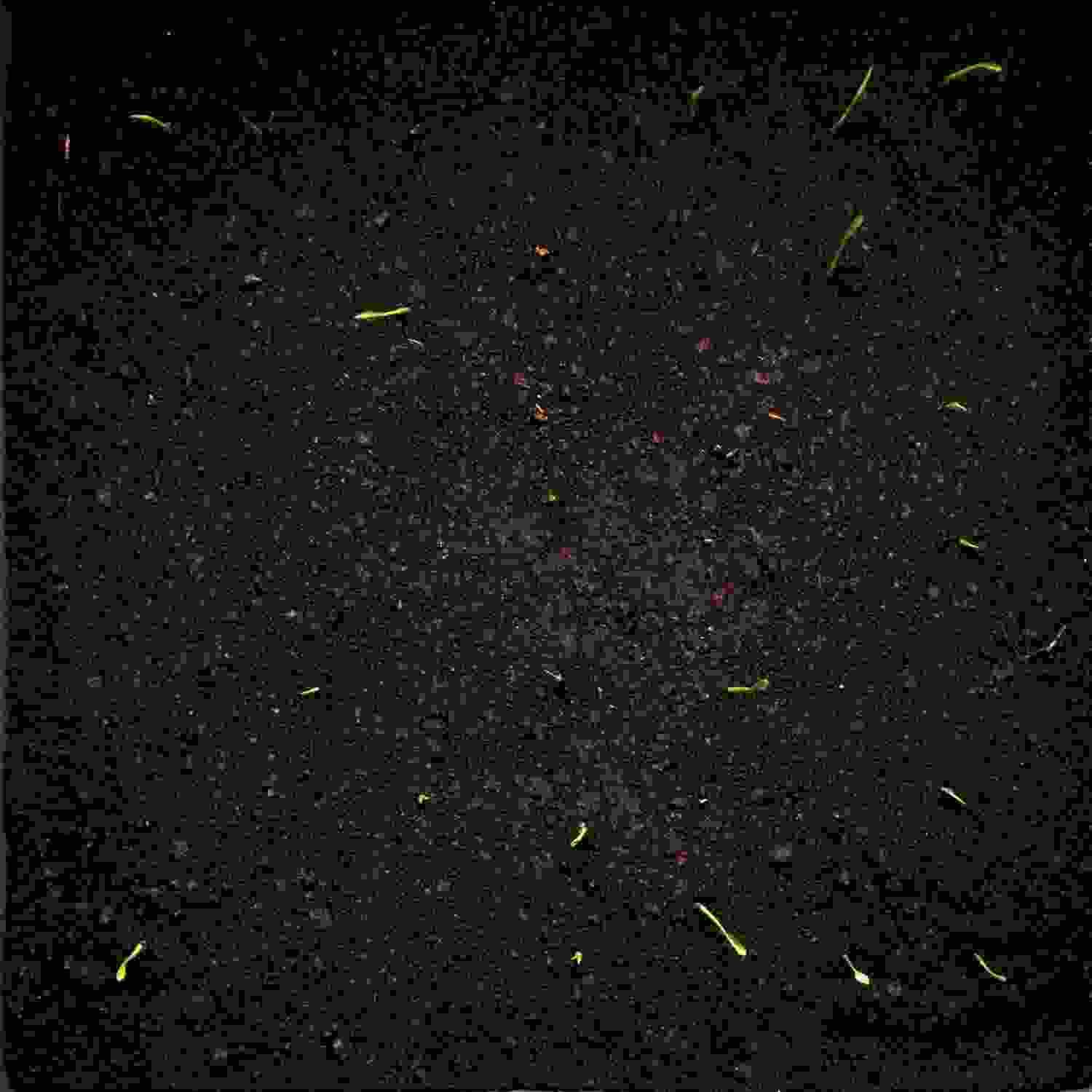

Supplement: Supplementary file 3 [file DataSheet3.zip › train1/5030-2024-3-18-20-20-45.JPG]

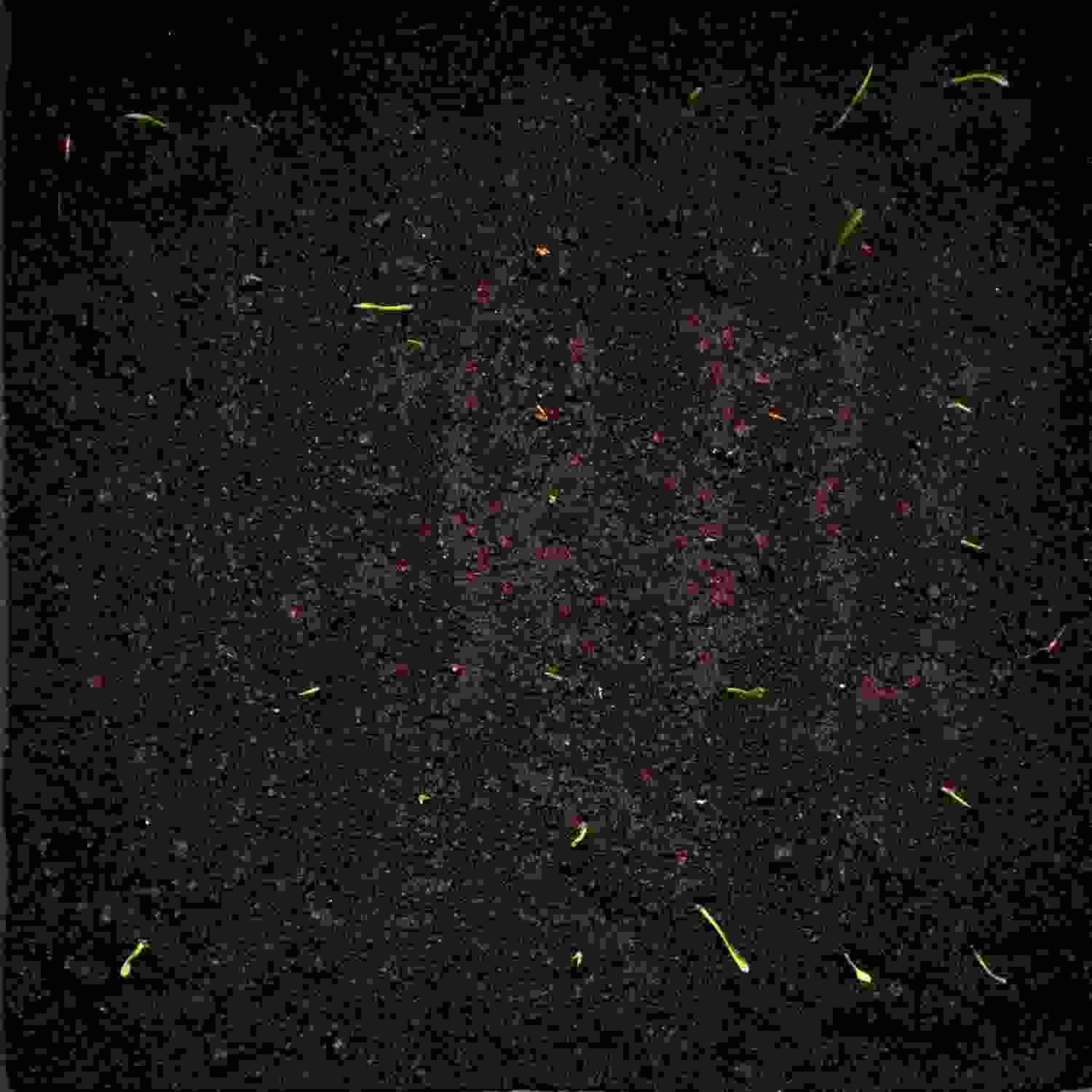

Supplement: Supplementary file 3 [file DataSheet3.zip › train1/5030-2024-3-18-23-8-54.JPG]

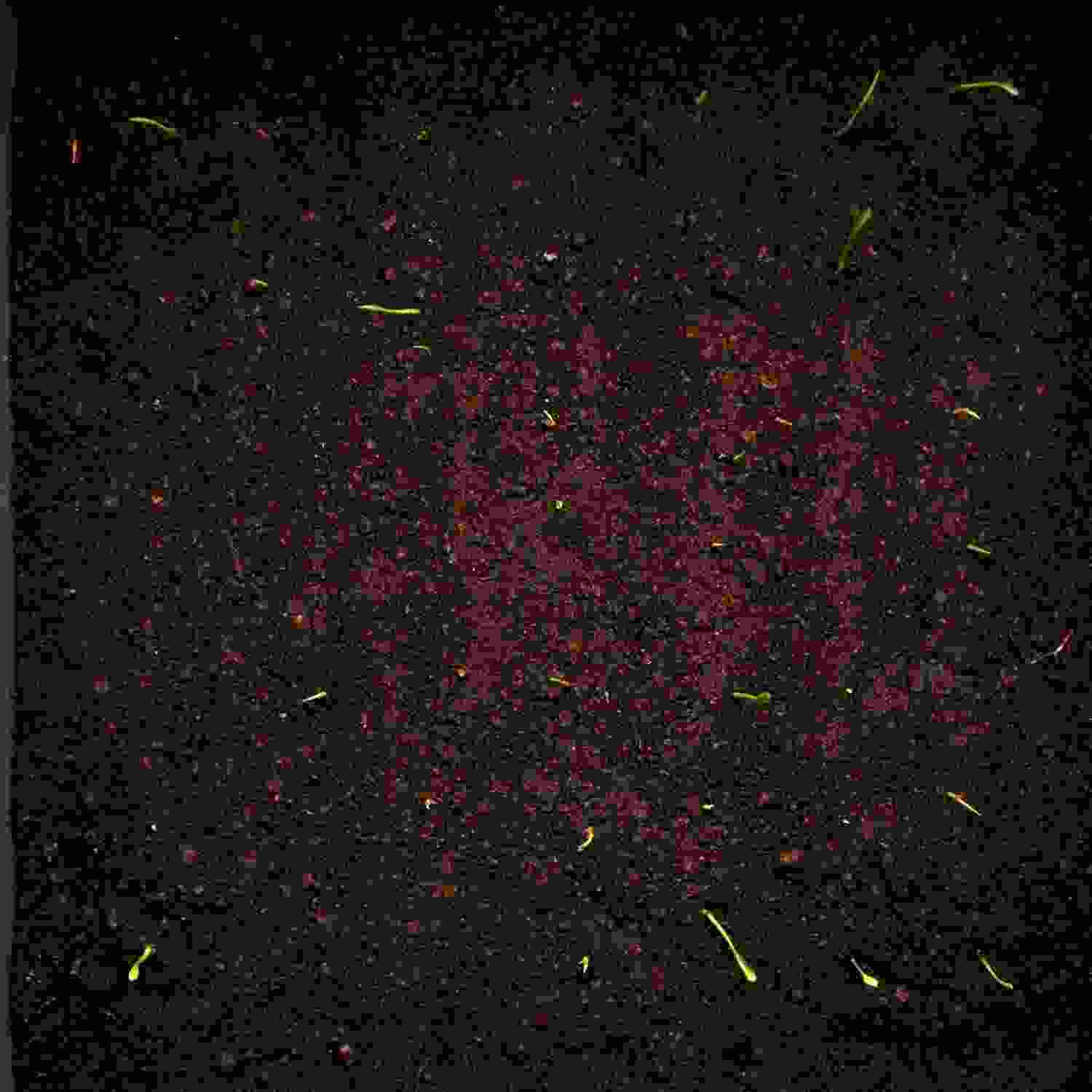

Supplement: Supplementary file 3 [file DataSheet3.zip › train1/5030-2024-3-19-1-56-12.JPG]

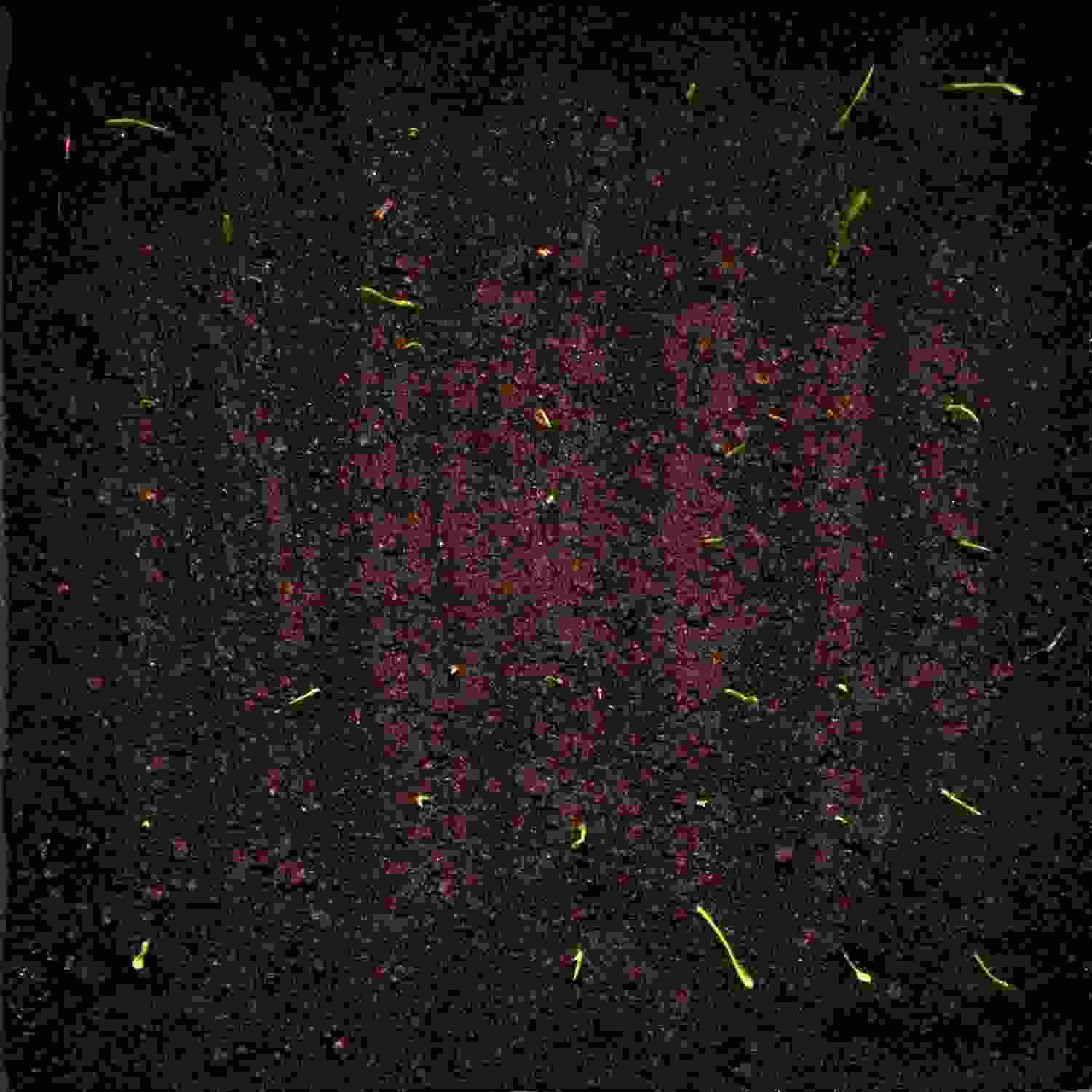

Supplement: Supplementary file 3 [file DataSheet3.zip › train1/5030-2024-3-19-10-19-23.JPG]

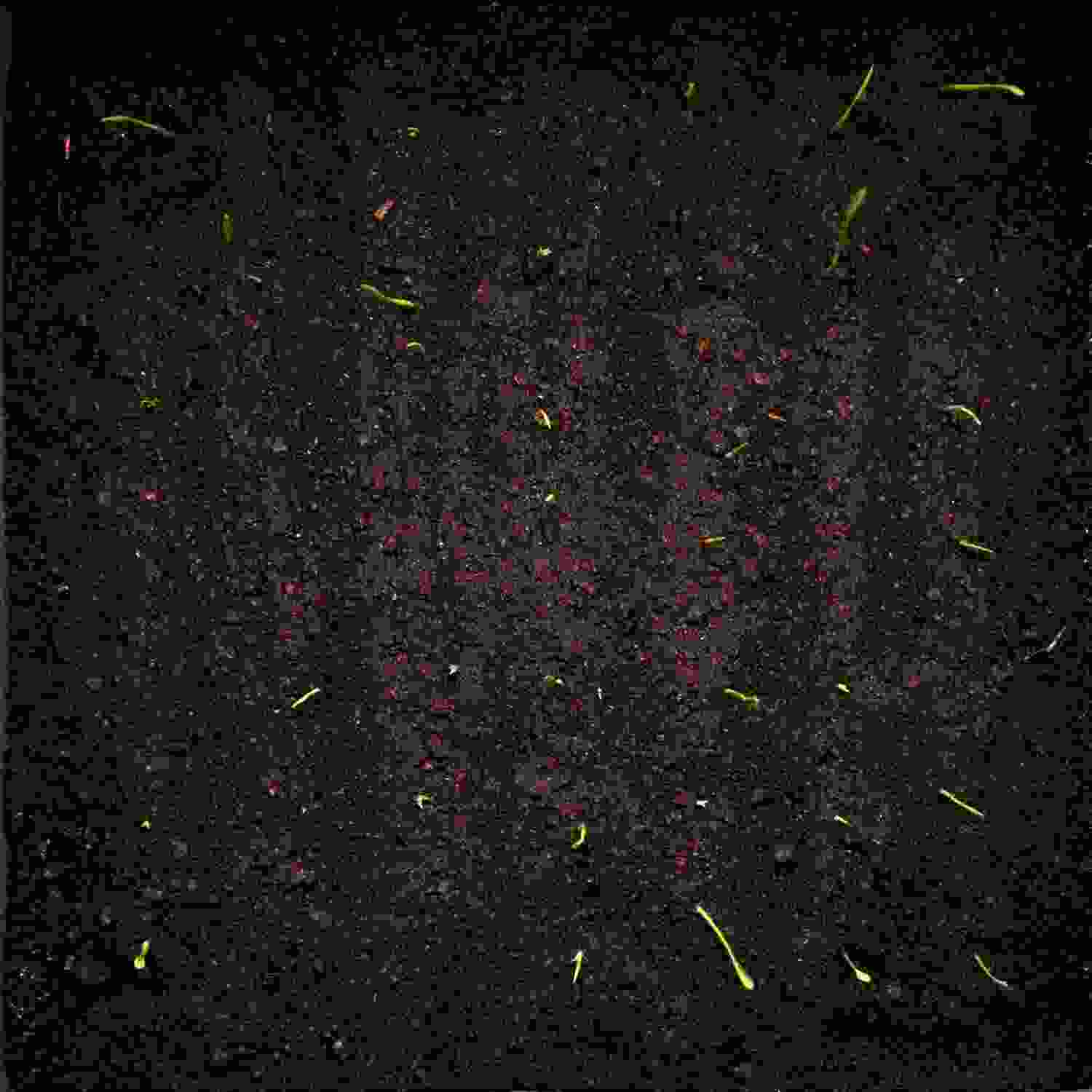

Supplement: Supplementary file 3 [file DataSheet3.zip › train1/5030-2024-3-19-13-7-4.JPG]

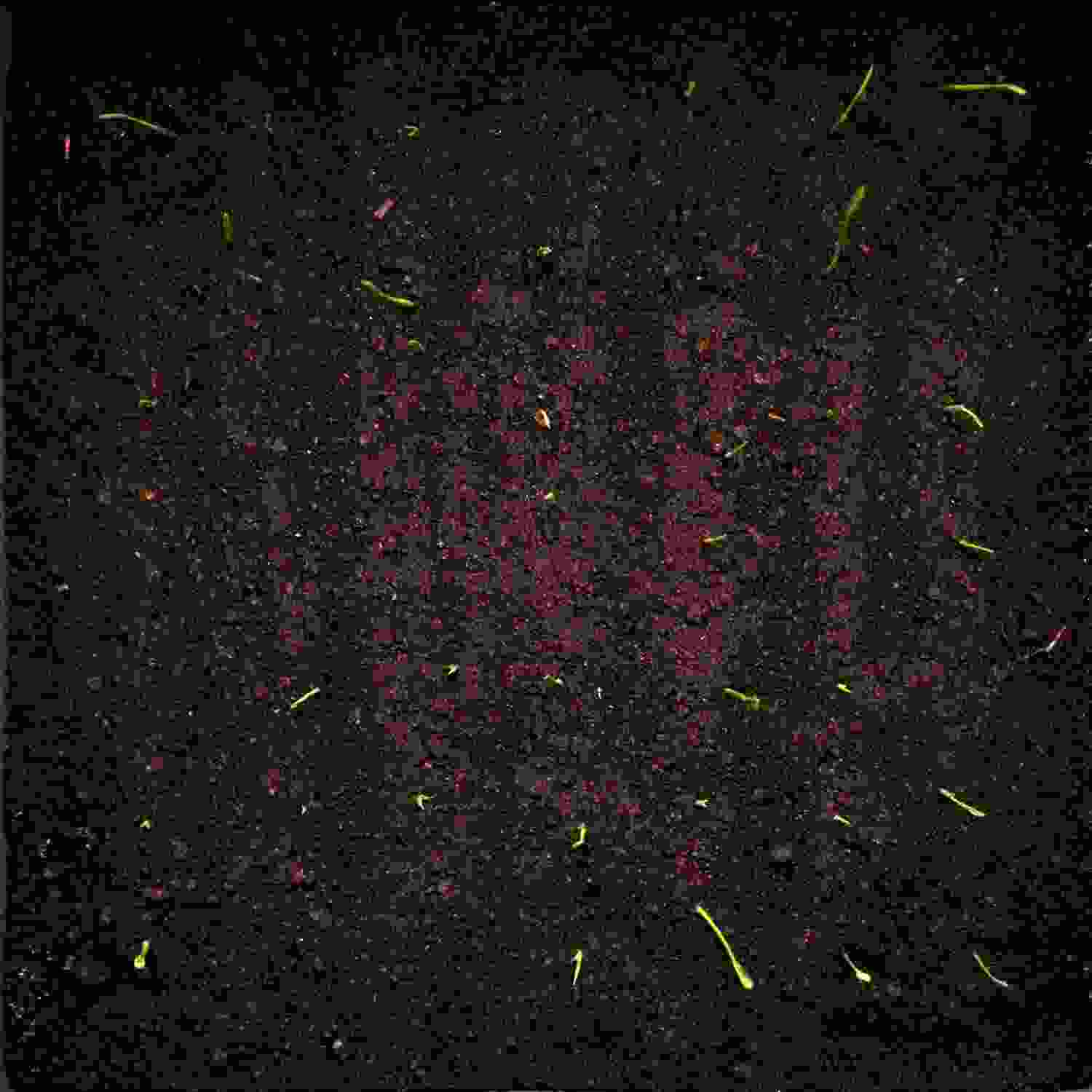

Supplement: Supplementary file 3 [file DataSheet3.zip › train1/5030-2024-3-19-15-55-1.JPG]

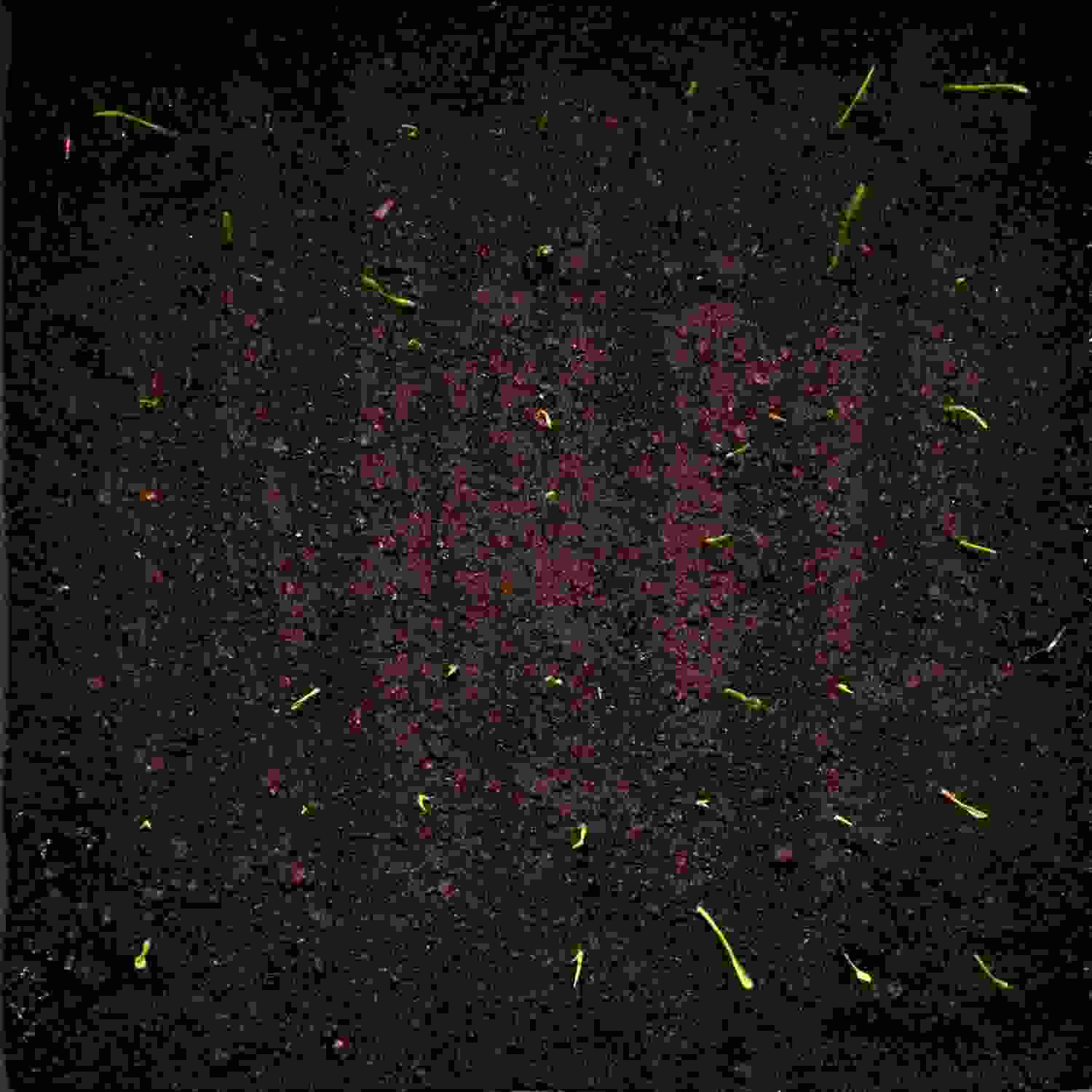

Supplement: Supplementary file 3 [file DataSheet3.zip › train1/5030-2024-3-19-18-41-55.JPG]

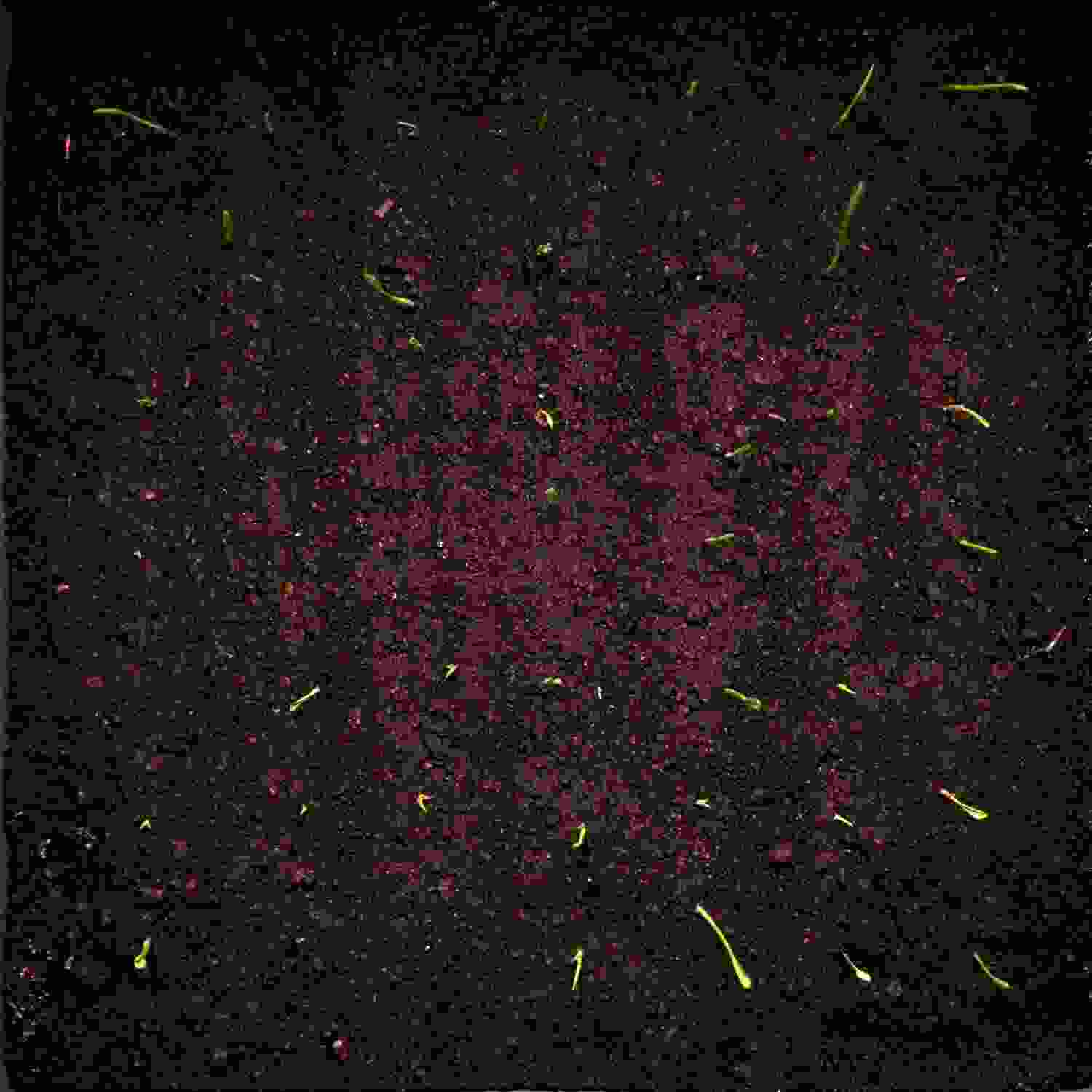

Supplement: Supplementary file 3 [file DataSheet3.zip › train1/5030-2024-3-19-21-29-49.JPG]

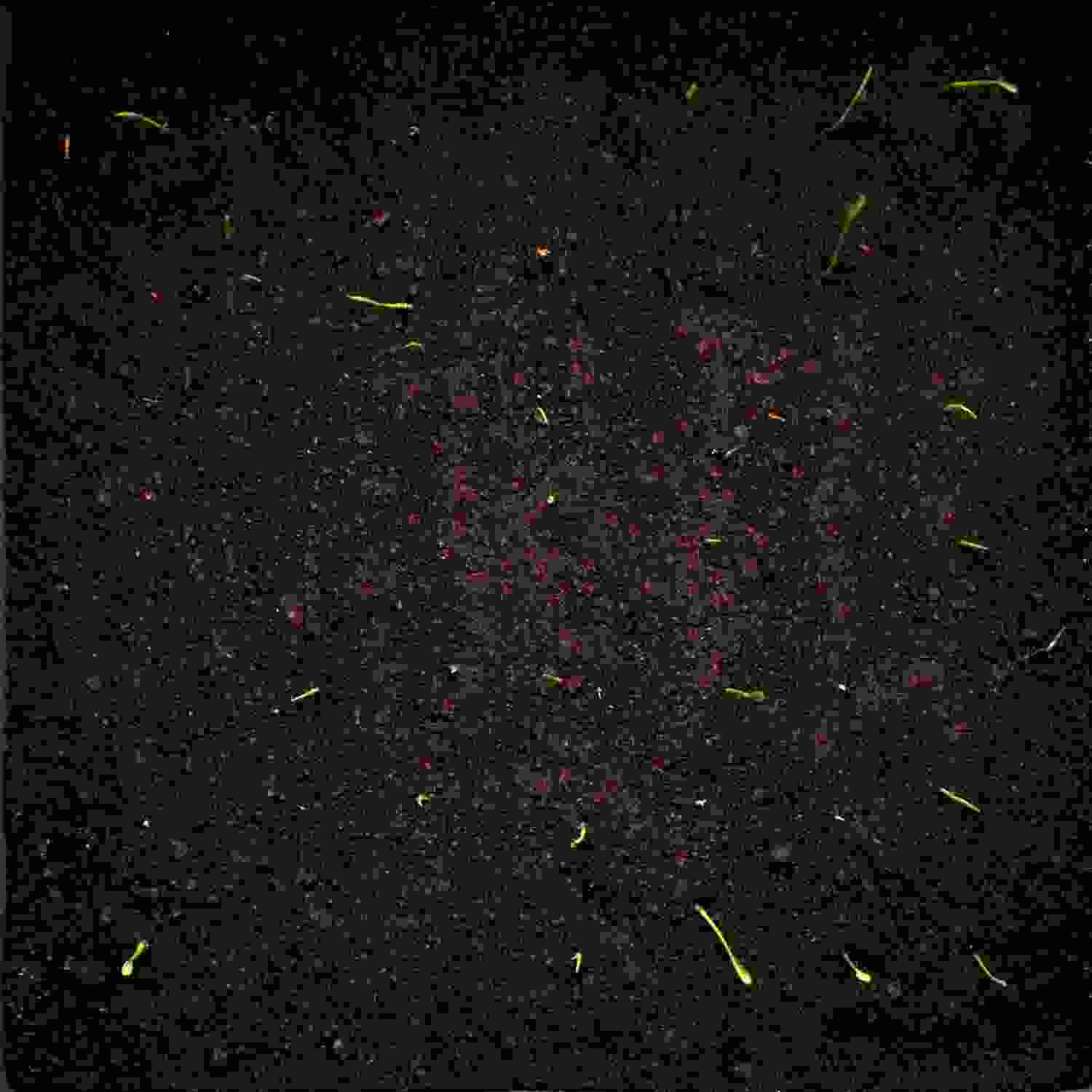

Supplement: Supplementary file 3 [file DataSheet3.zip › train1/5030-2024-3-19-7-31-22.JPG]

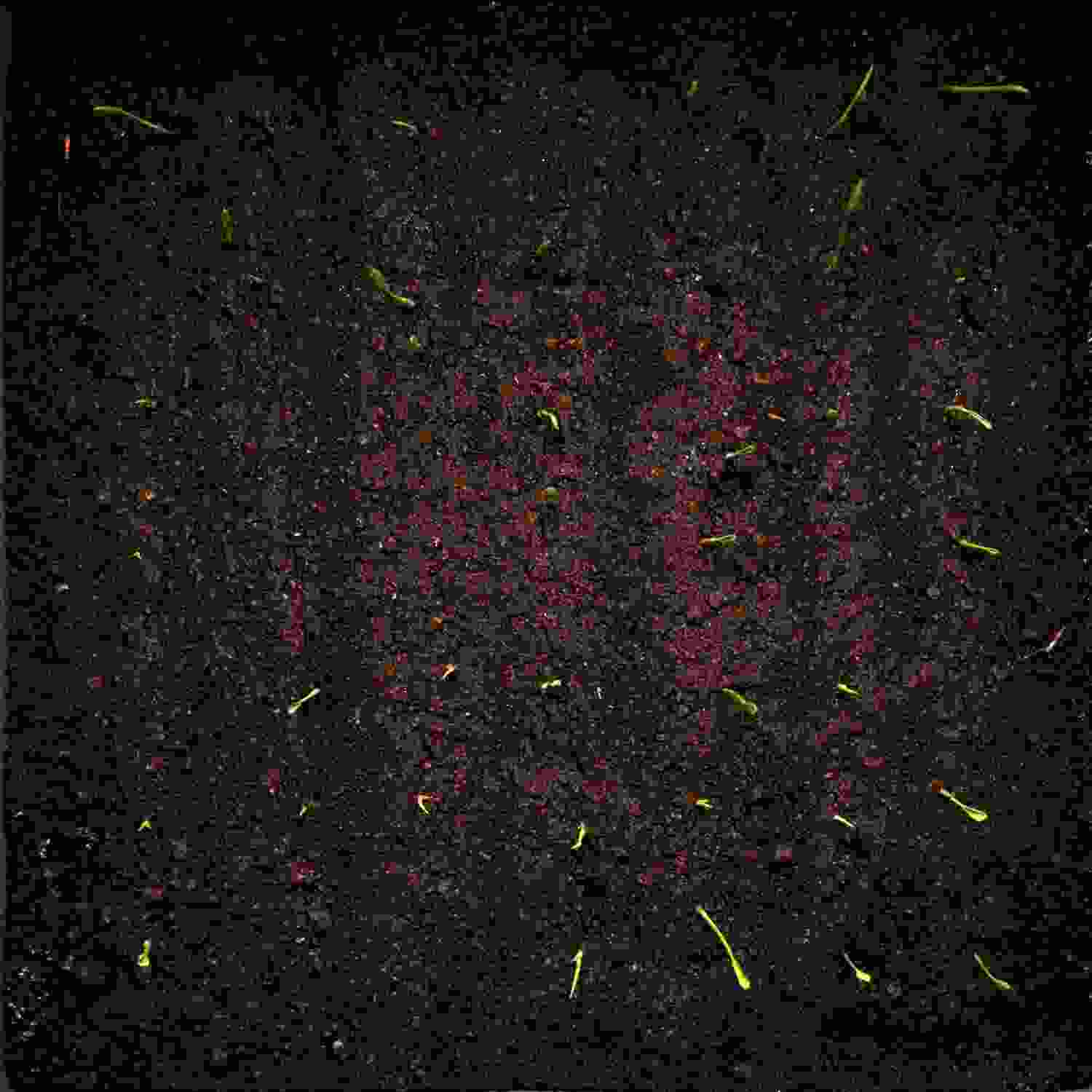

Supplement: Supplementary file 3 [file DataSheet3.zip › train1/5030-2024-3-20-0-17-38.JPG]

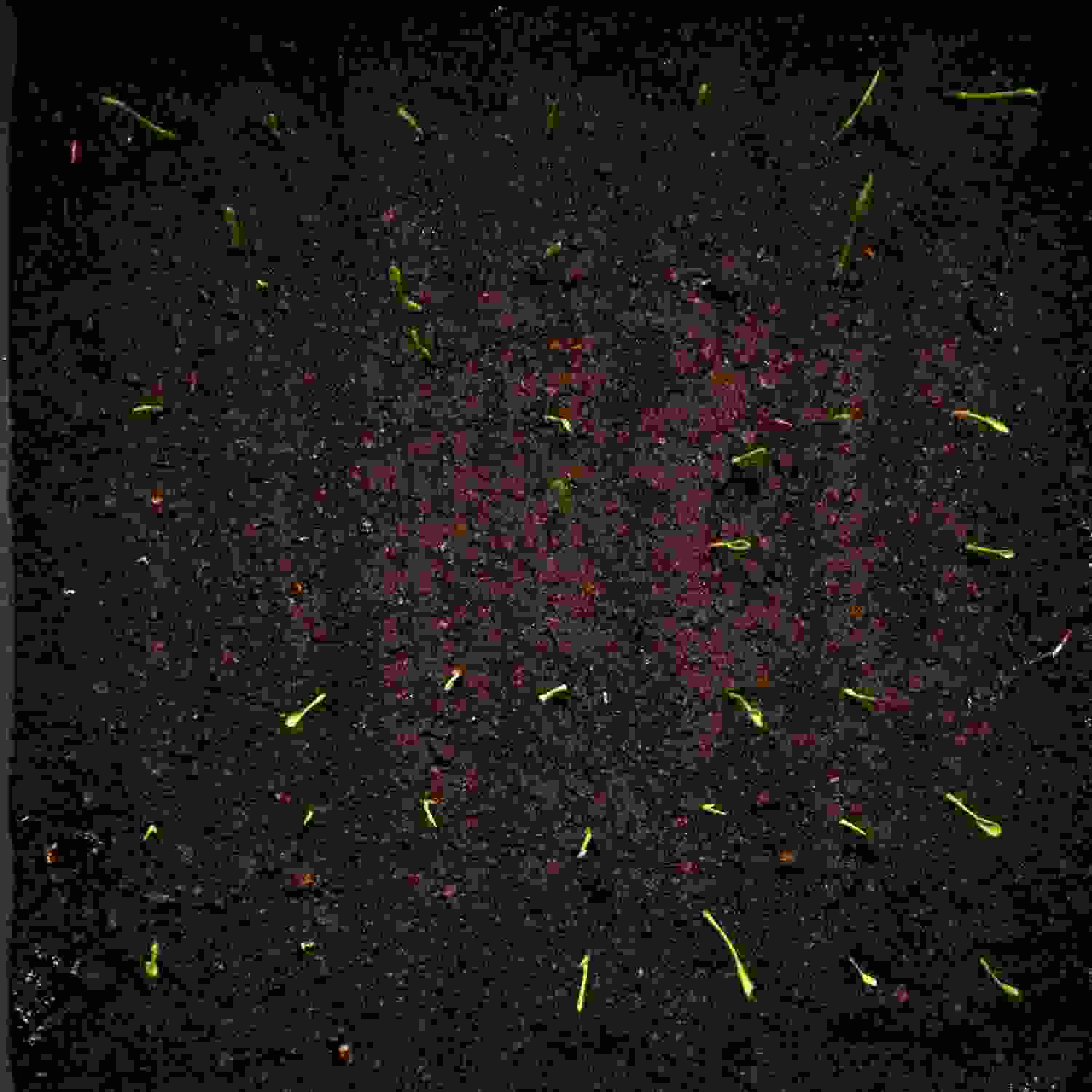

Supplement: Supplementary file 3 [file DataSheet3.zip › train1/5030-2024-3-20-11-27-40.JPG]

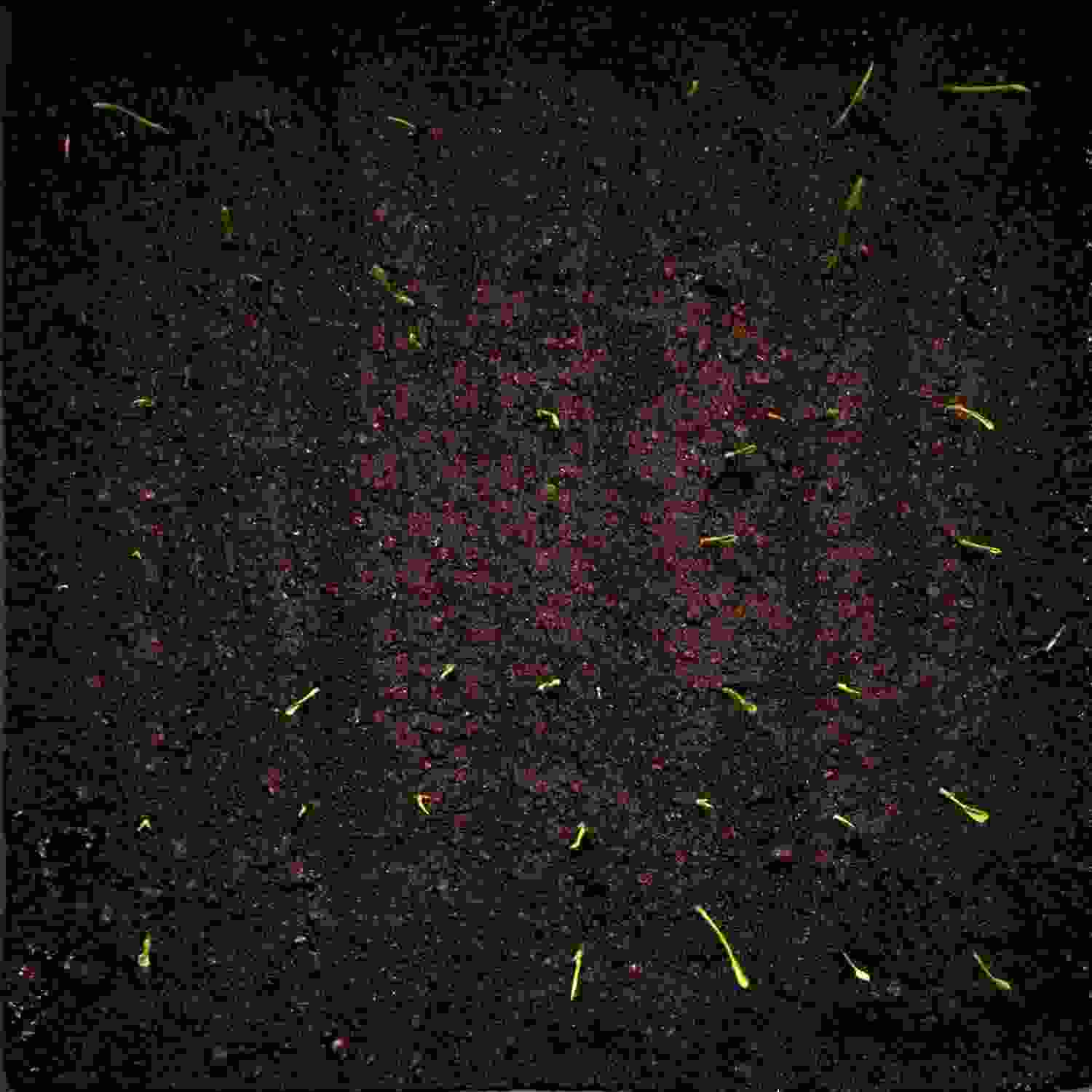

Supplement: Supplementary file 3 [file DataSheet3.zip › train1/5030-2024-3-20-3-4-46.JPG]

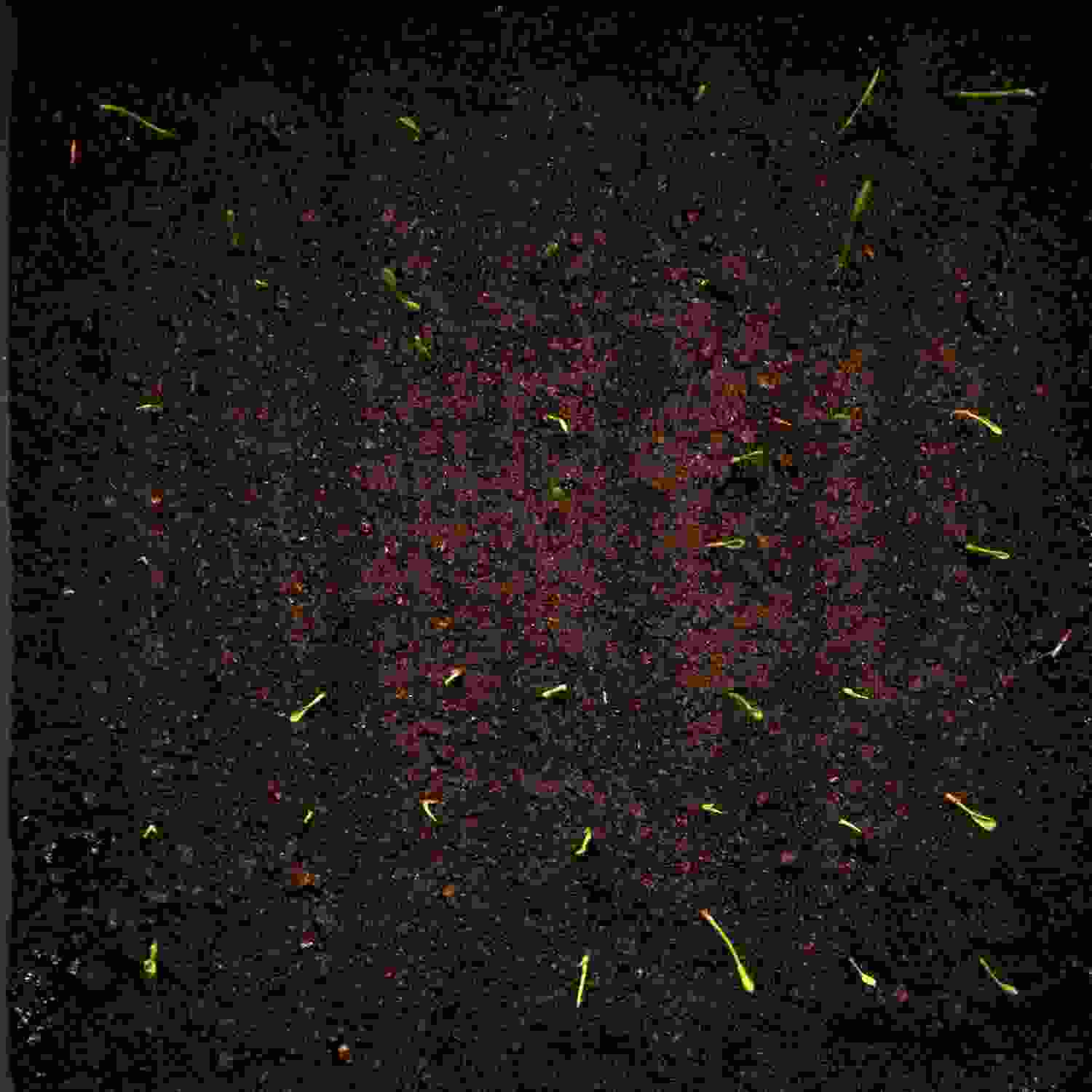

Supplement: Supplementary file 3 [file DataSheet3.zip › train1/5030-2024-3-20-5-52-29.JPG]

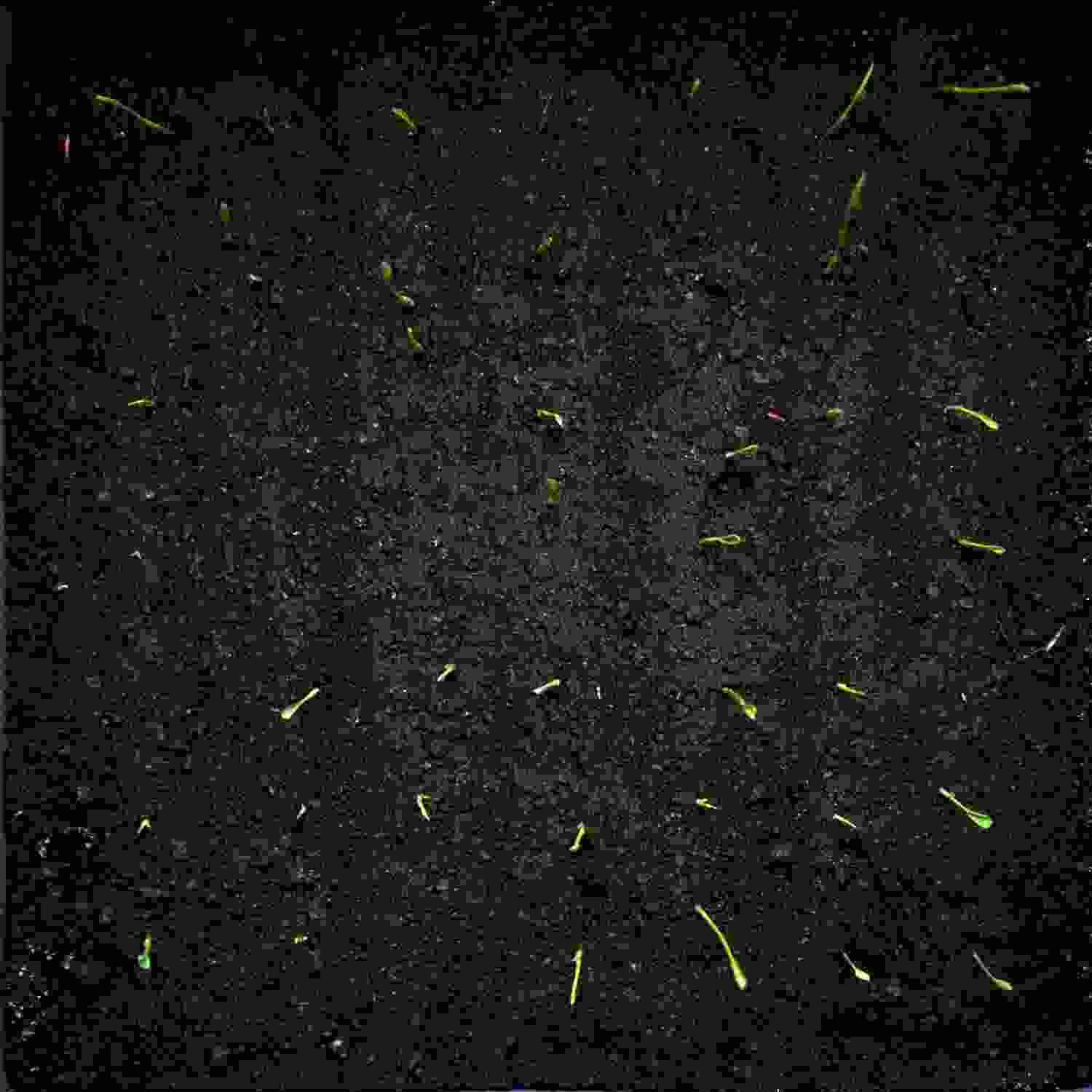

Supplement: Supplementary file 3 [file DataSheet3.zip › train1/5030-2024-3-20-8-40-29.JPG]

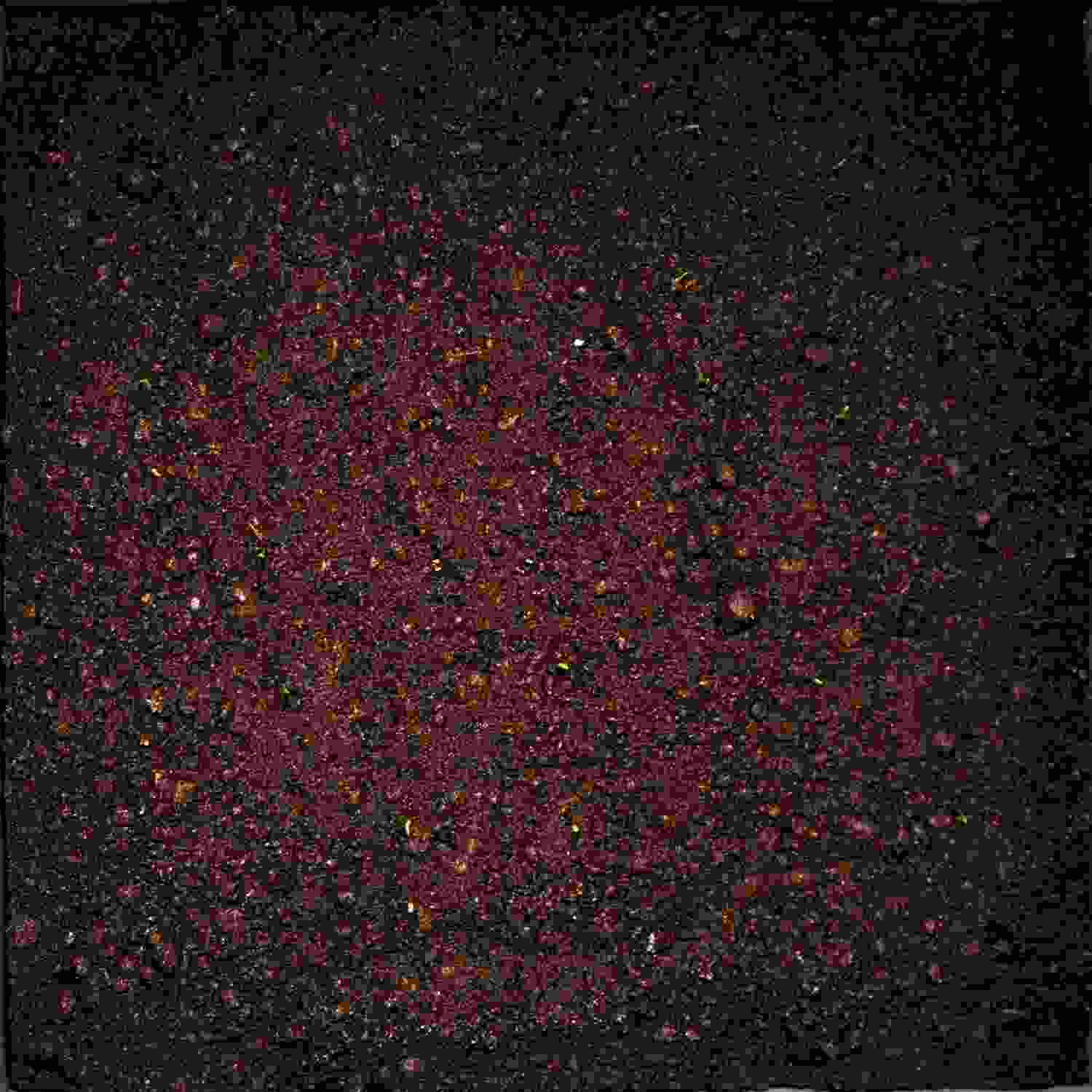

Supplement: Supplementary file 3 [file DataSheet3.zip › train1/5060-2024-3-18-15-12-43.JPG]

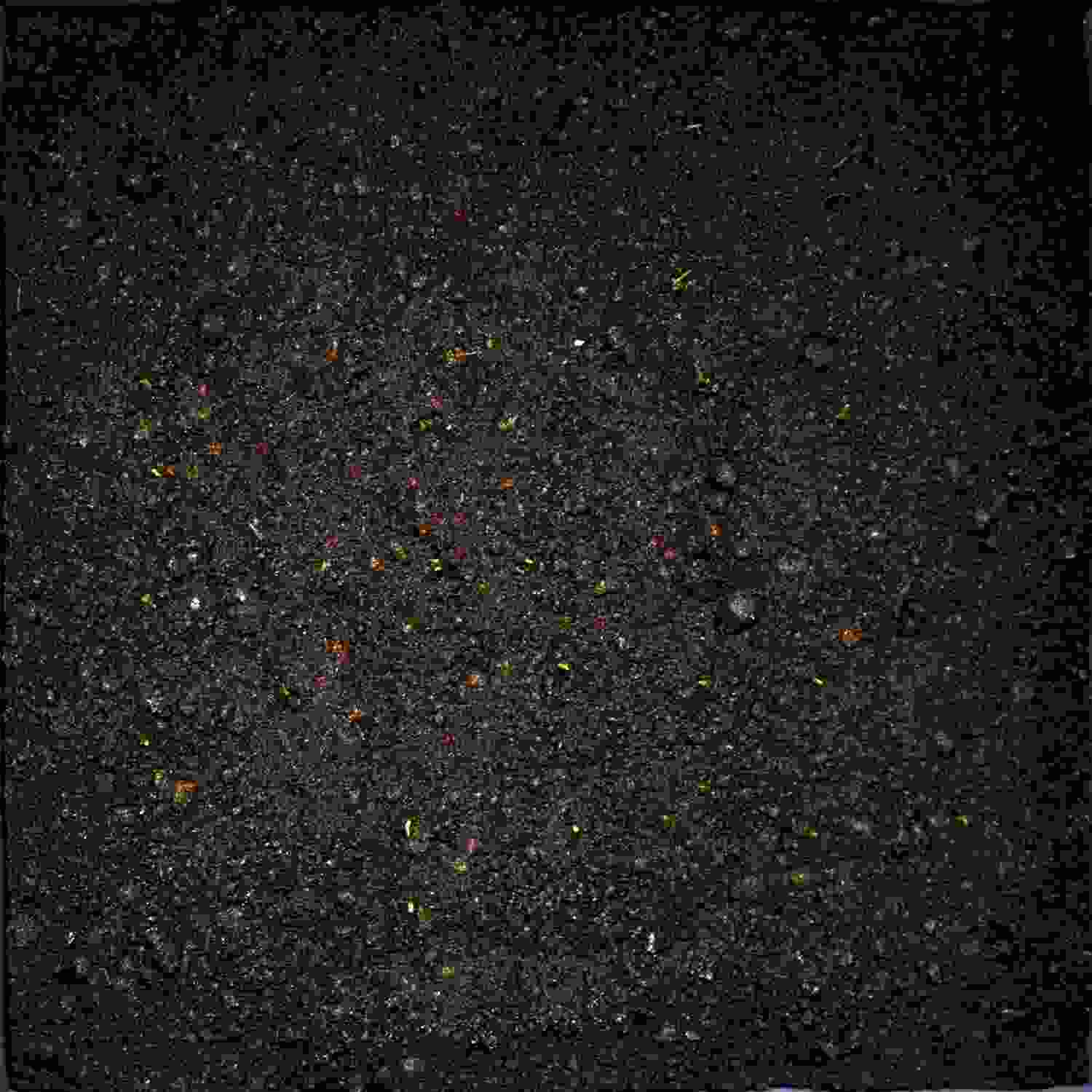

Supplement: Supplementary file 3 [file DataSheet3.zip › train1/5060-2024-3-18-17-46-12.JPG]

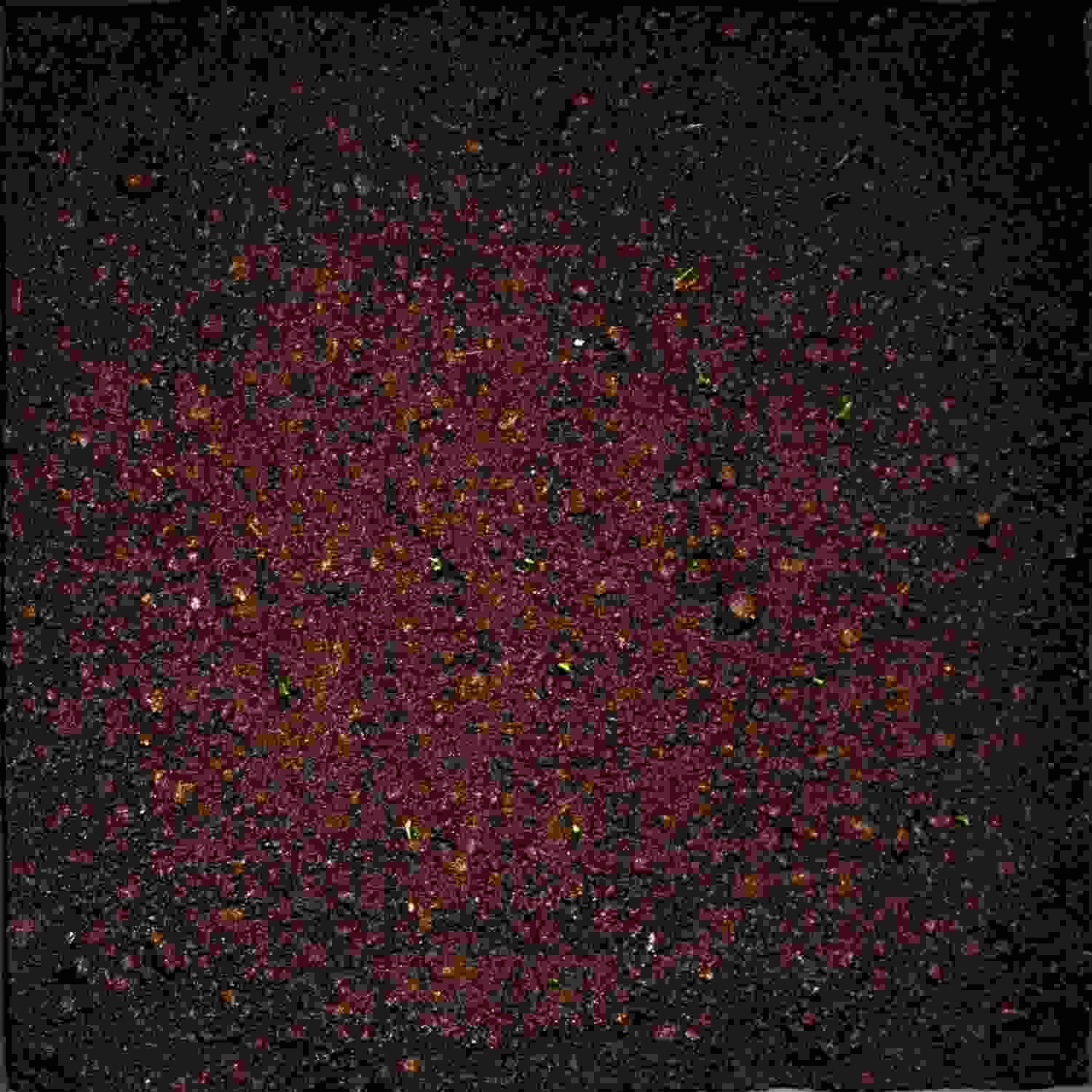

Supplement: Supplementary file 3 [file DataSheet3.zip › train1/5060-2024-3-18-20-20-36.JPG]

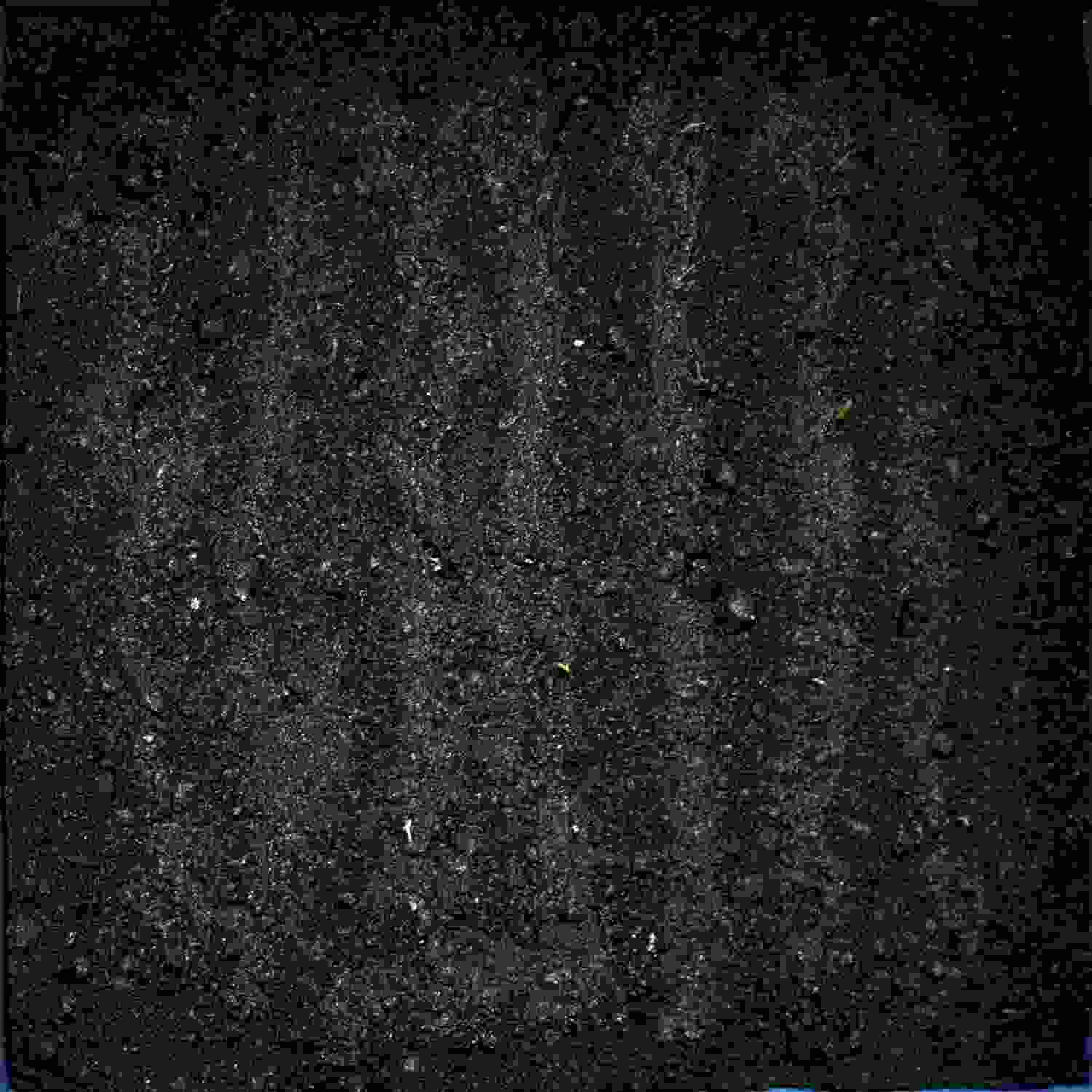

Supplement: Supplementary file 3 [file DataSheet3.zip › train1/5060-2024-3-18-22-54-16.JPG]

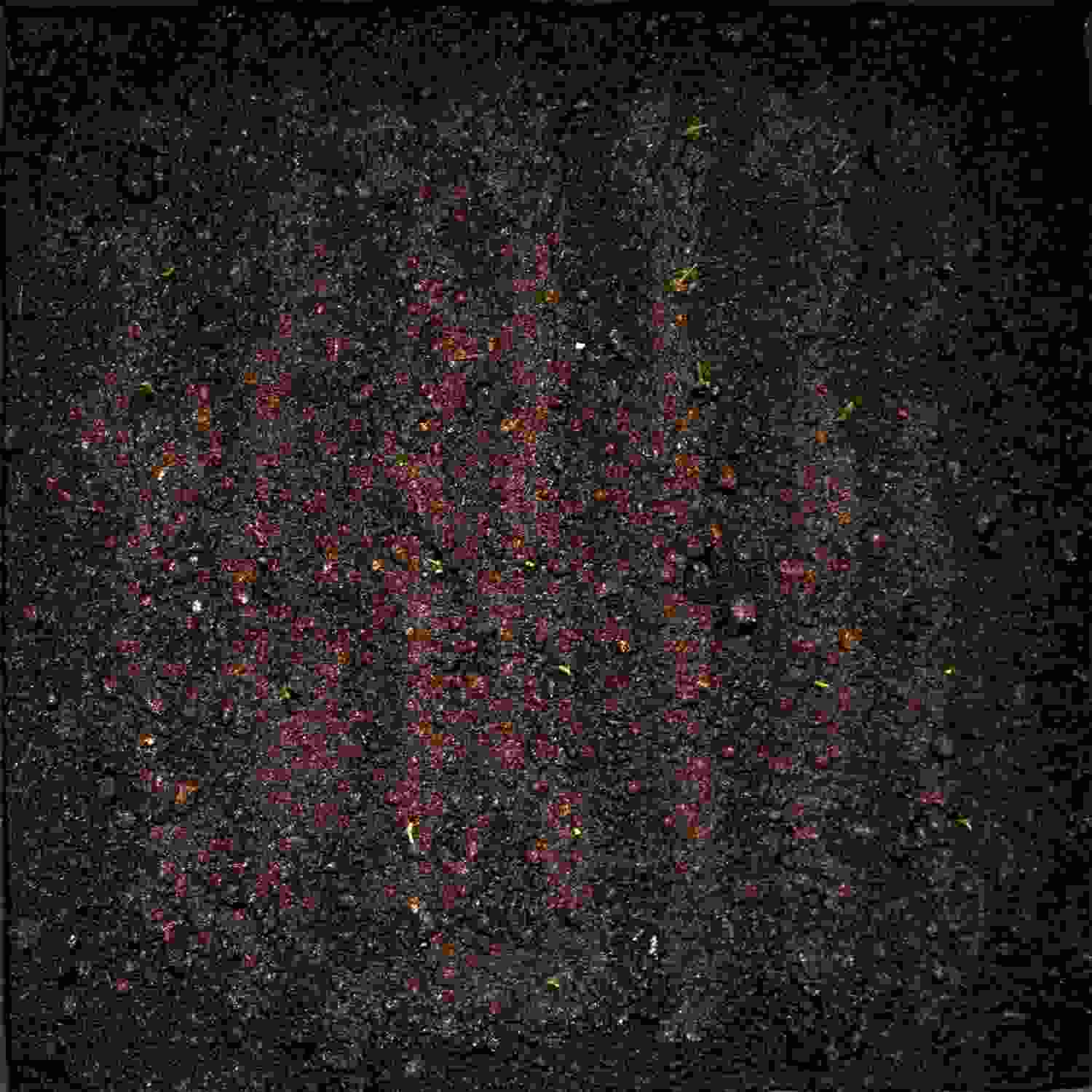

Supplement: Supplementary file 3 [file DataSheet3.zip › train1/5060-2024-3-19-1-27-29.JPG]

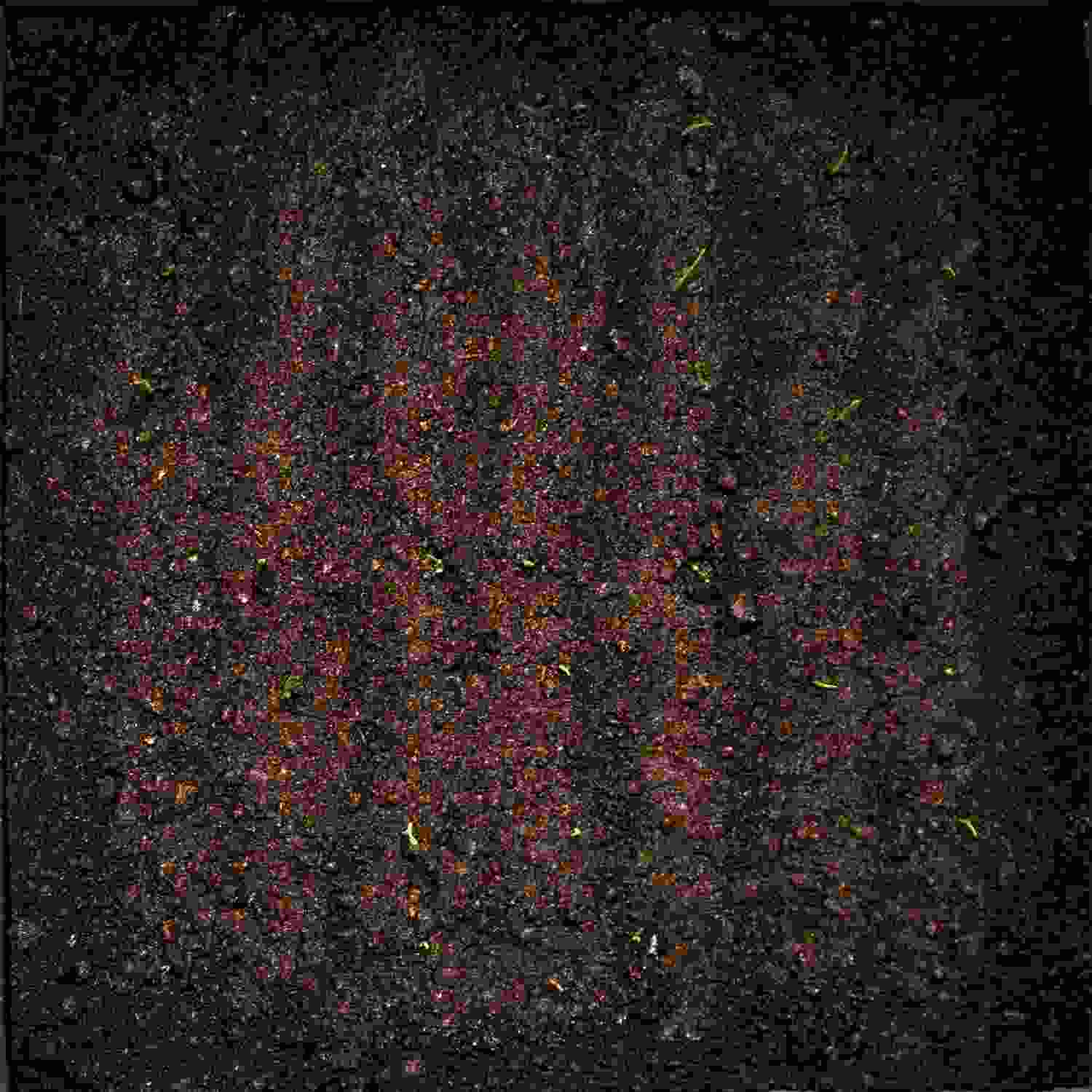

Supplement: Supplementary file 3 [file DataSheet3.zip › train1/5060-2024-3-19-11-38-12.JPG]

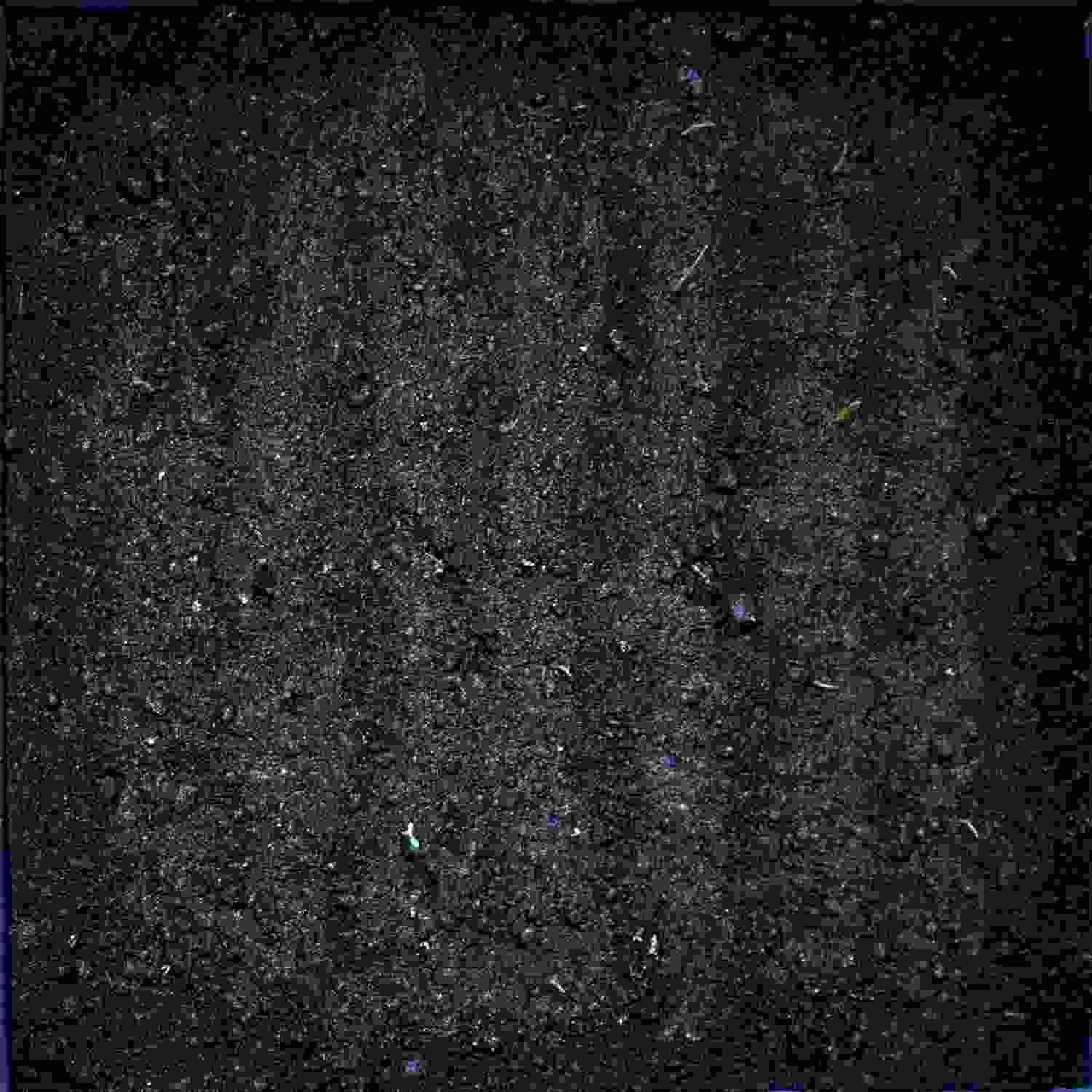

Supplement: Supplementary file 3 [file DataSheet3.zip › train1/5060-2024-3-19-14-9-54.JPG]

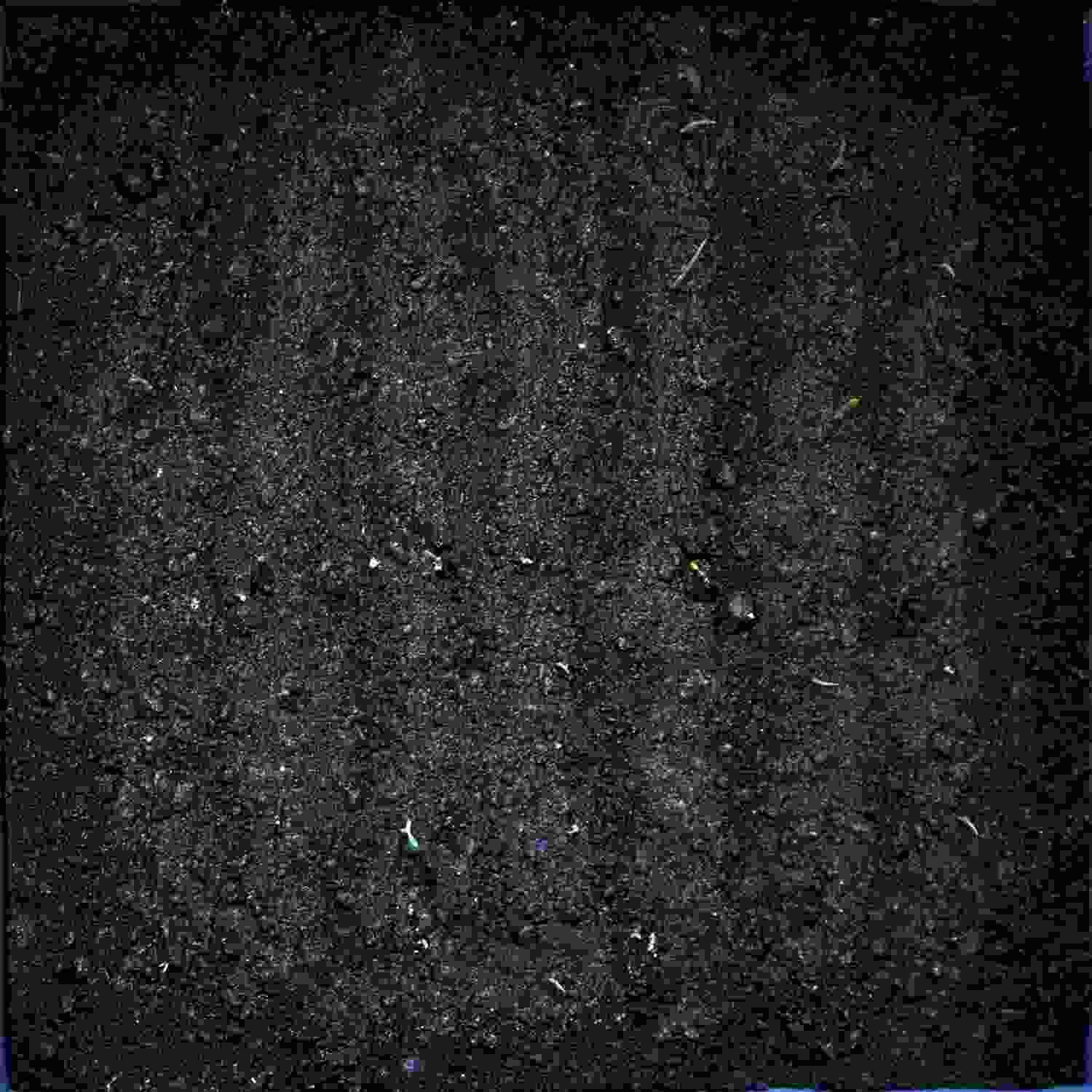

Supplement: Supplementary file 3 [file DataSheet3.zip › train1/5060-2024-3-19-16-42-2.JPG]

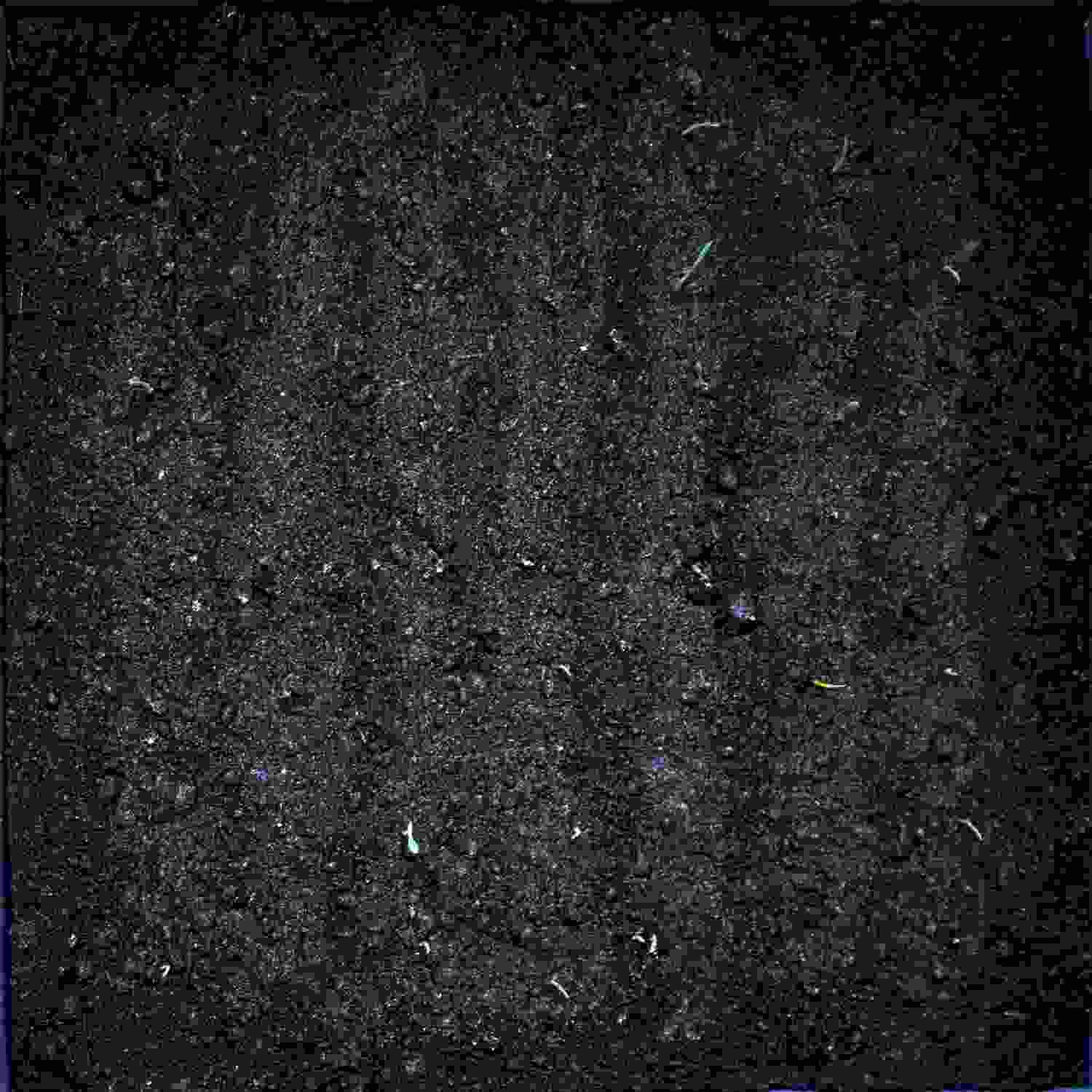

Supplement: Supplementary file 3 [file DataSheet3.zip › train1/5060-2024-3-19-21-45-47.JPG]

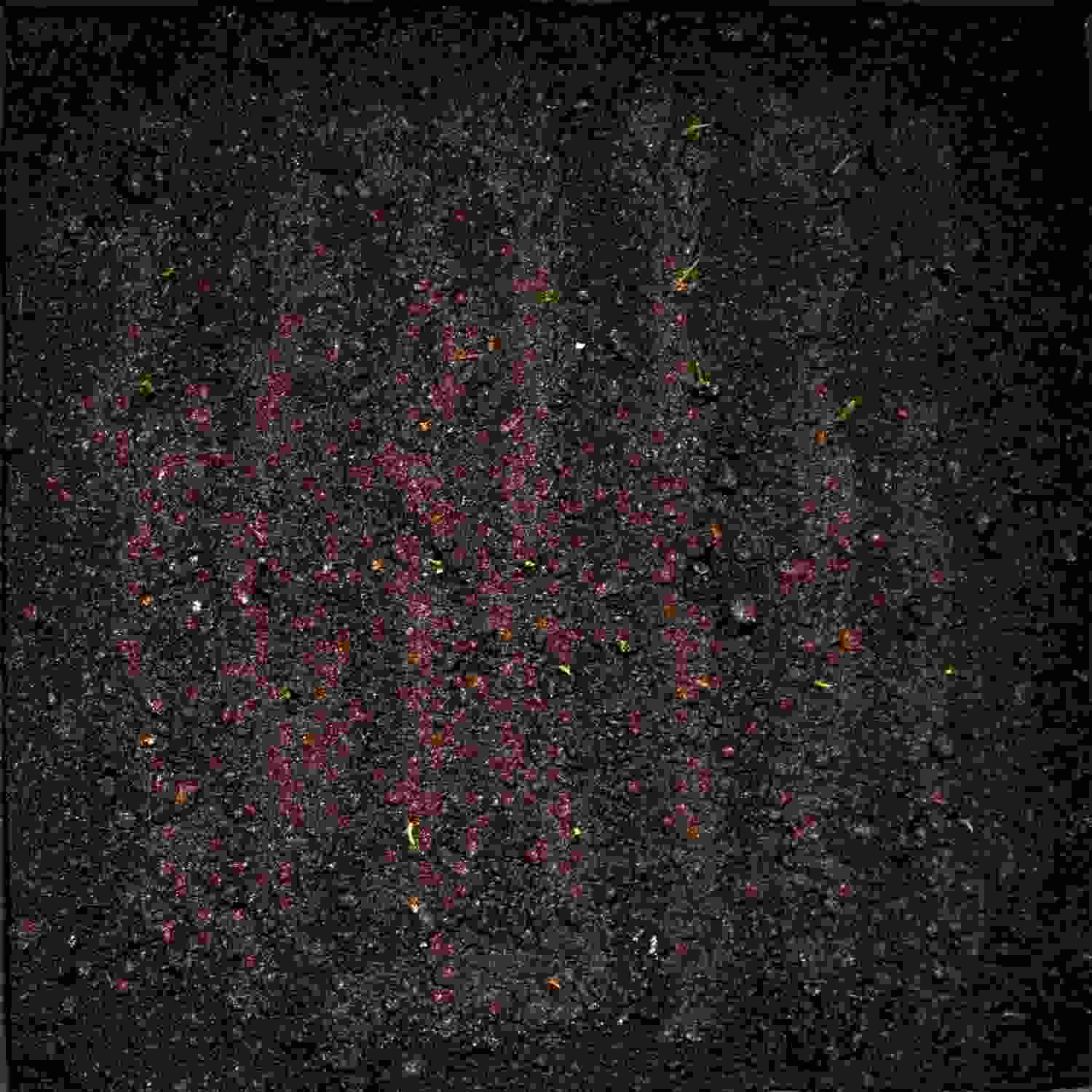

Supplement: Supplementary file 3 [file DataSheet3.zip › train1/5060-2024-3-19-3-59-57.JPG]

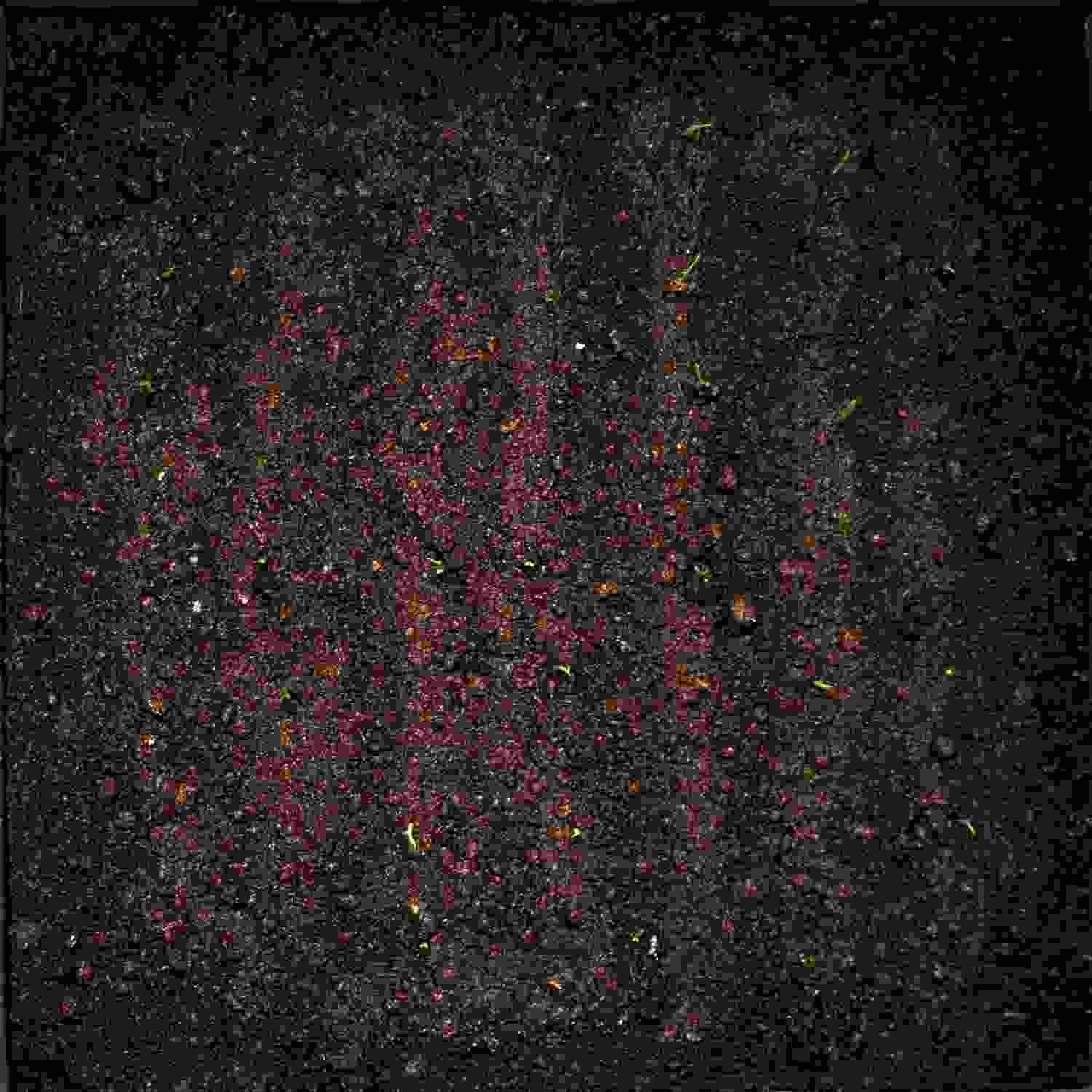

Supplement: Supplementary file 3 [file DataSheet3.zip › train1/5060-2024-3-19-6-32-44.JPG]

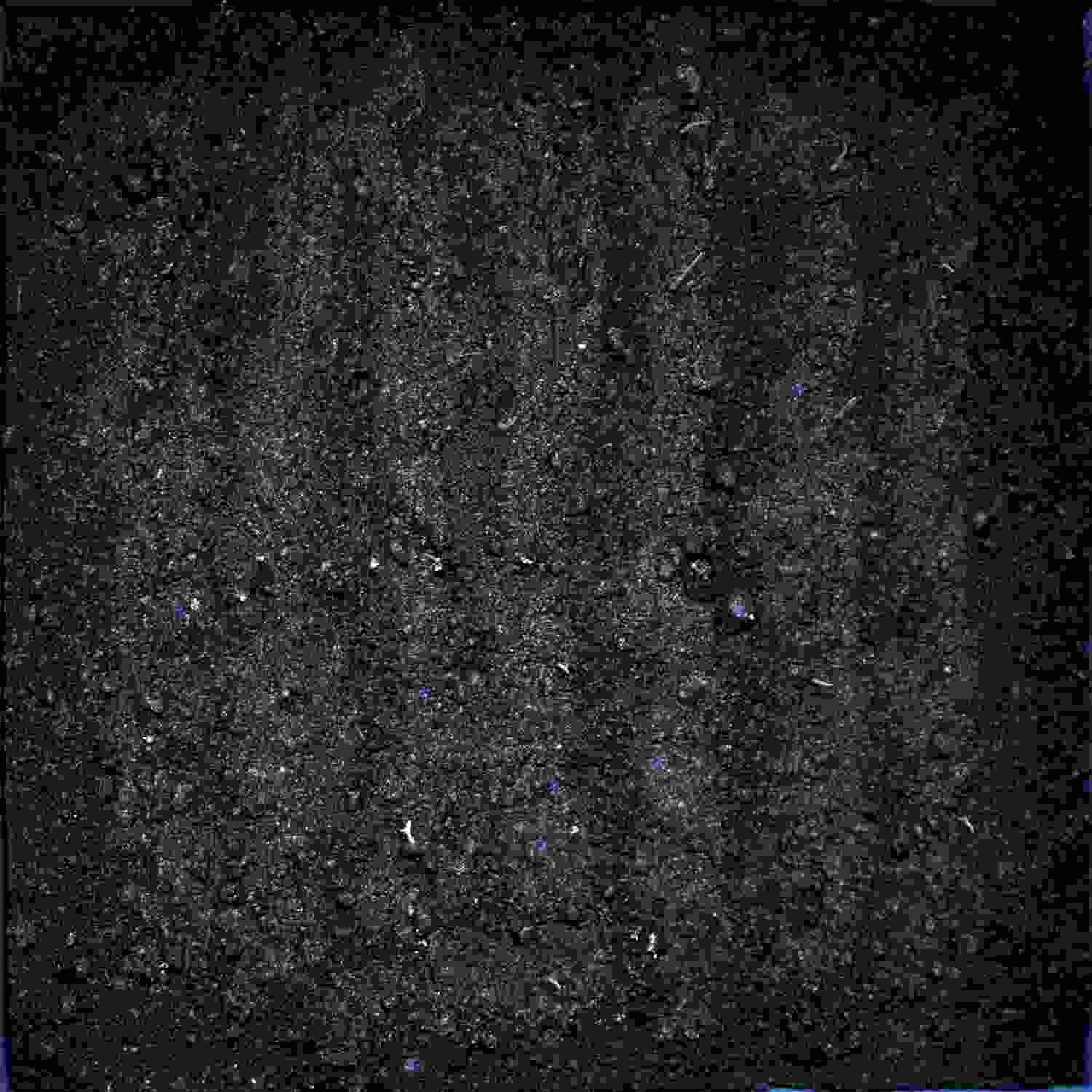

Supplement: Supplementary file 3 [file DataSheet3.zip › train1/5060-2024-3-19-9-5-22.JPG]

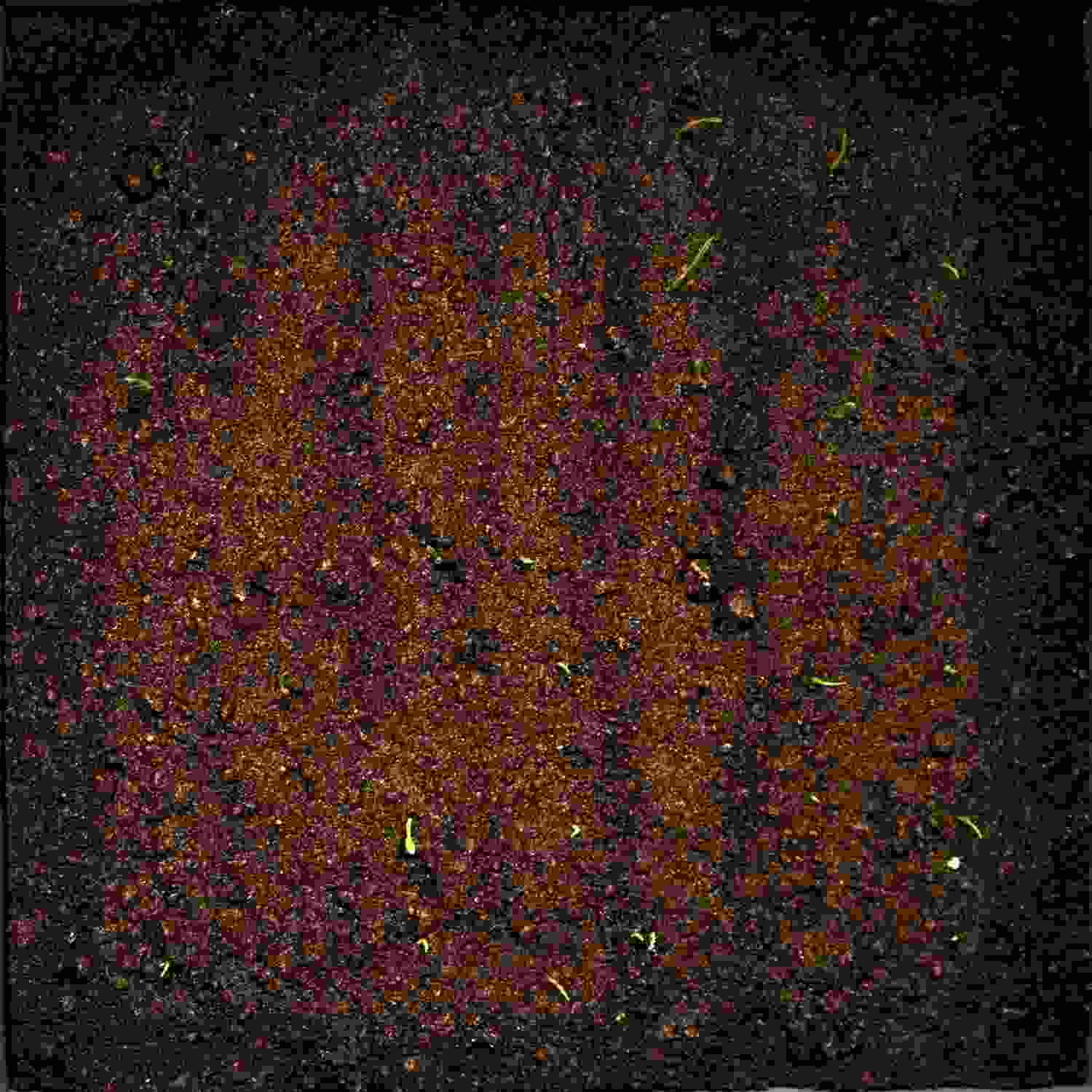

Supplement: Supplementary file 3 [file DataSheet3.zip › train1/5060-2024-3-20-0-18-53.JPG]

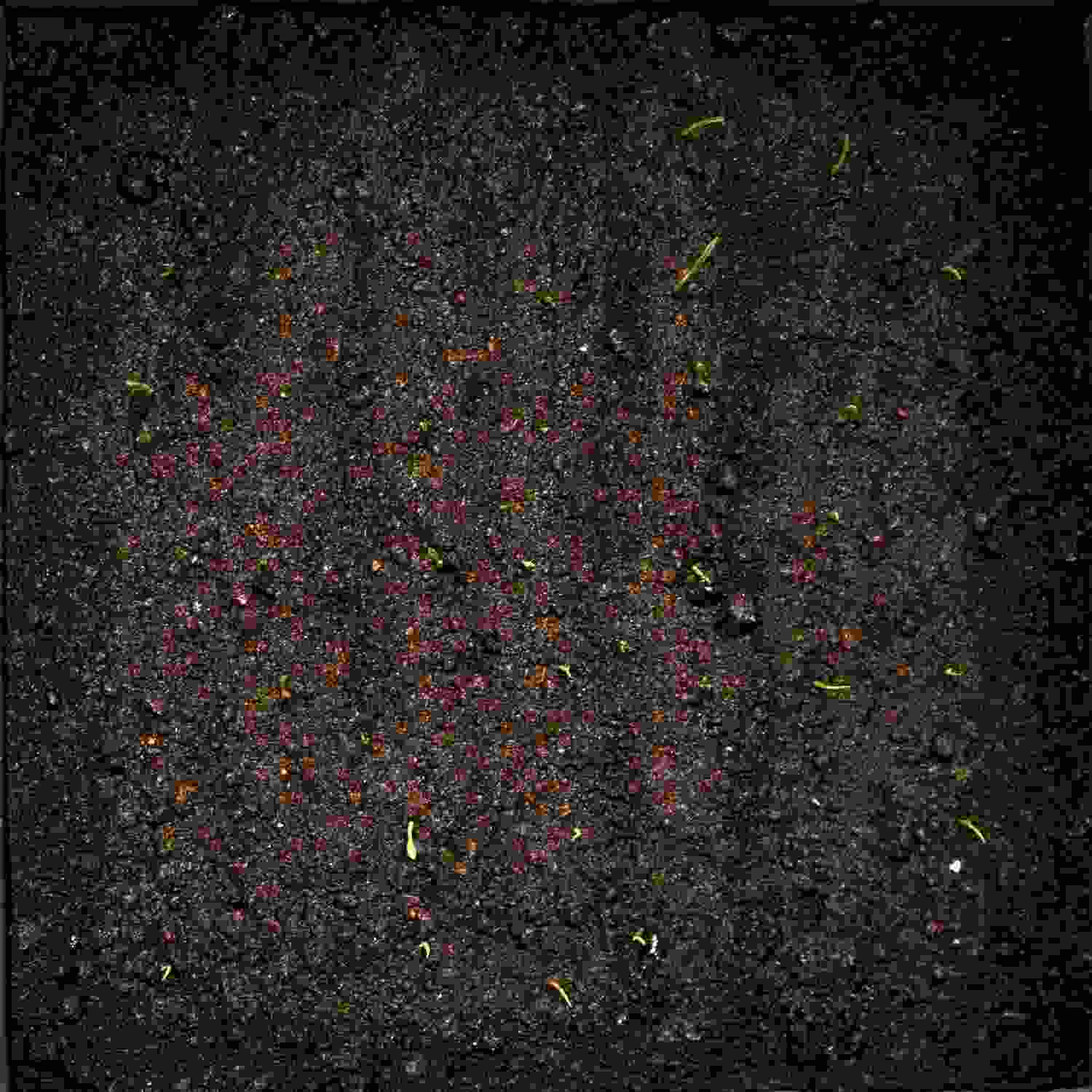

Supplement: Supplementary file 3 [file DataSheet3.zip › train1/5060-2024-3-20-2-51-0.JPG]

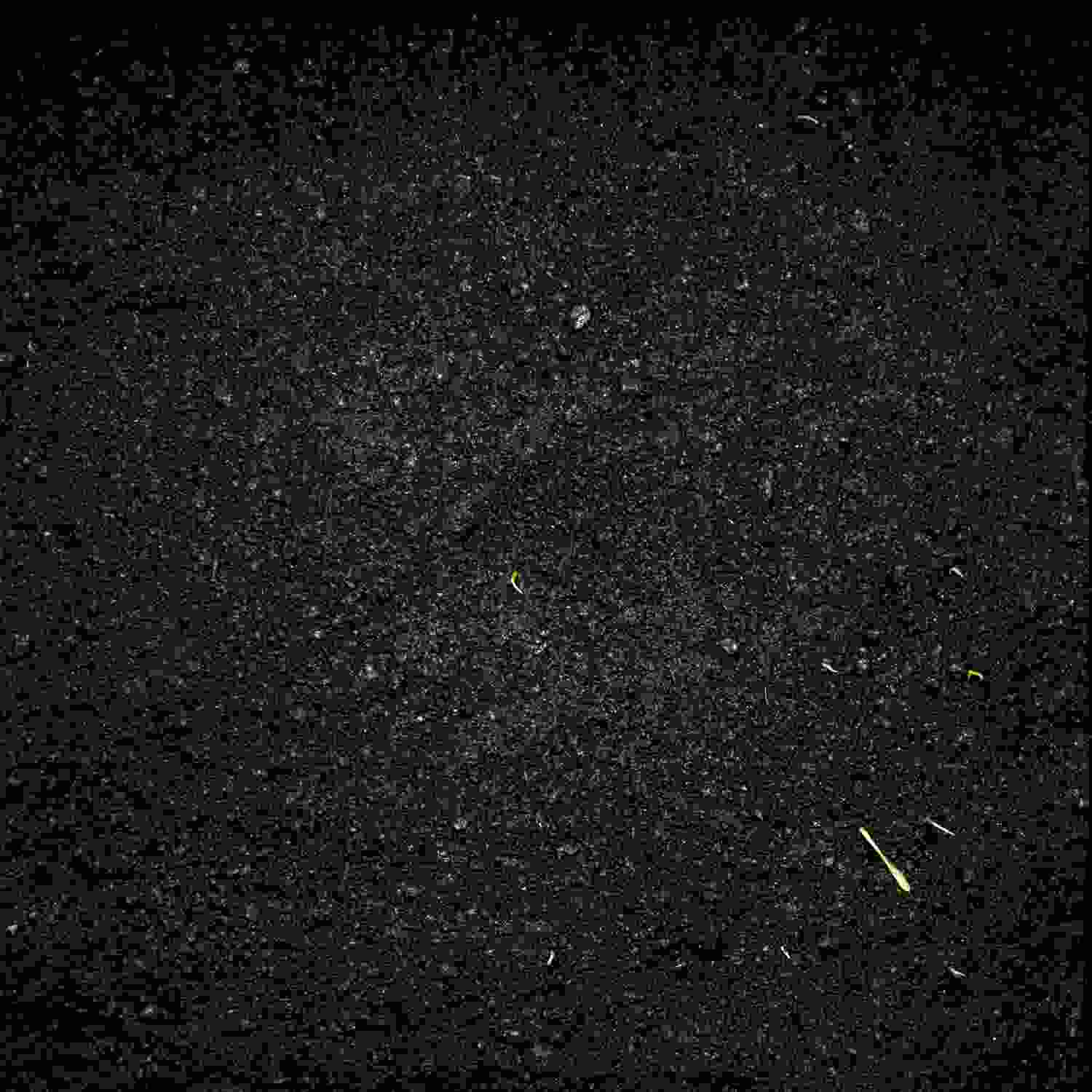

Supplement: Supplementary file 3 [file DataSheet3.zip › train1/5090-2024-3-18-14-47-17.JPG]

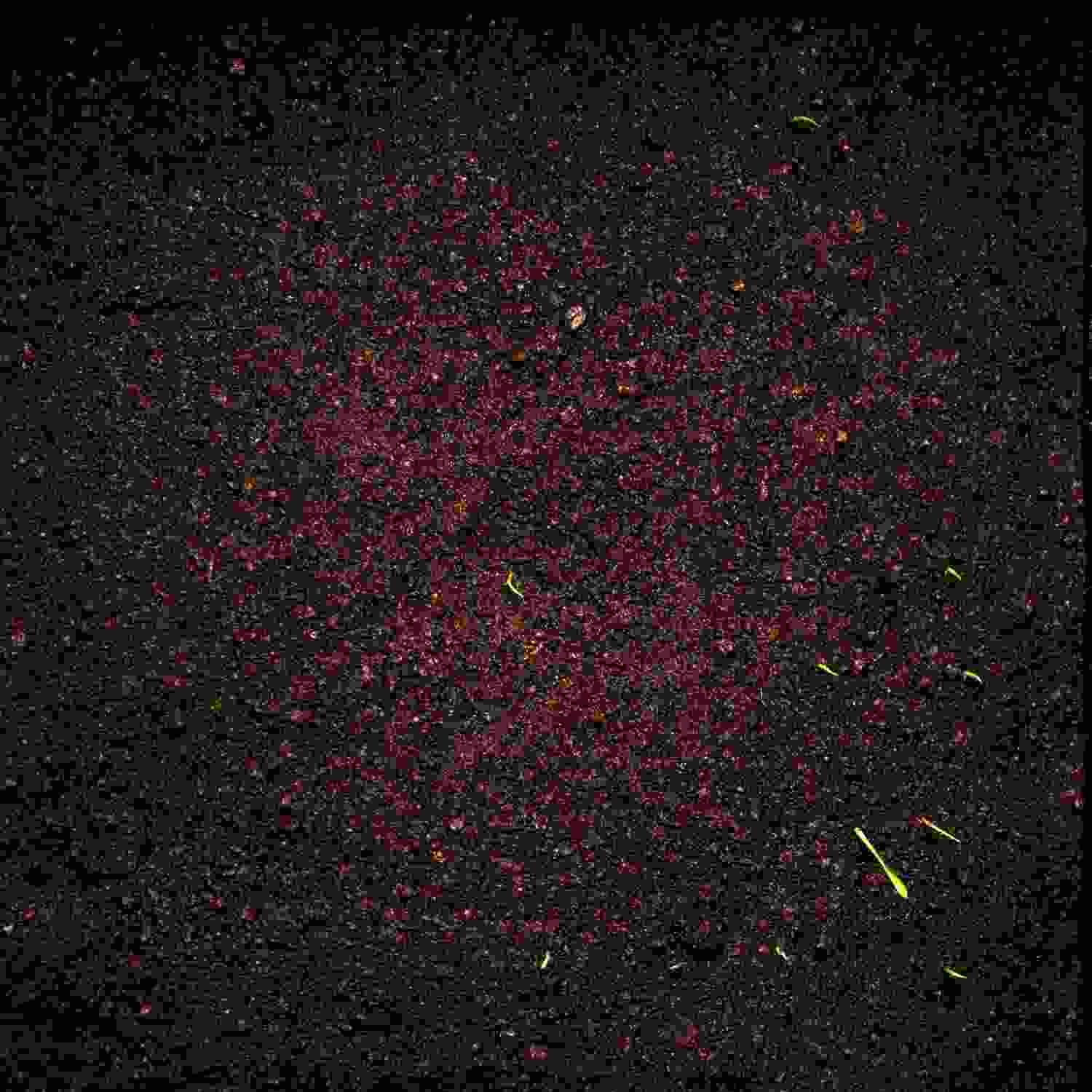

Supplement: Supplementary file 3 [file DataSheet3.zip › train1/5090-2024-3-18-19-44-38.JPG]

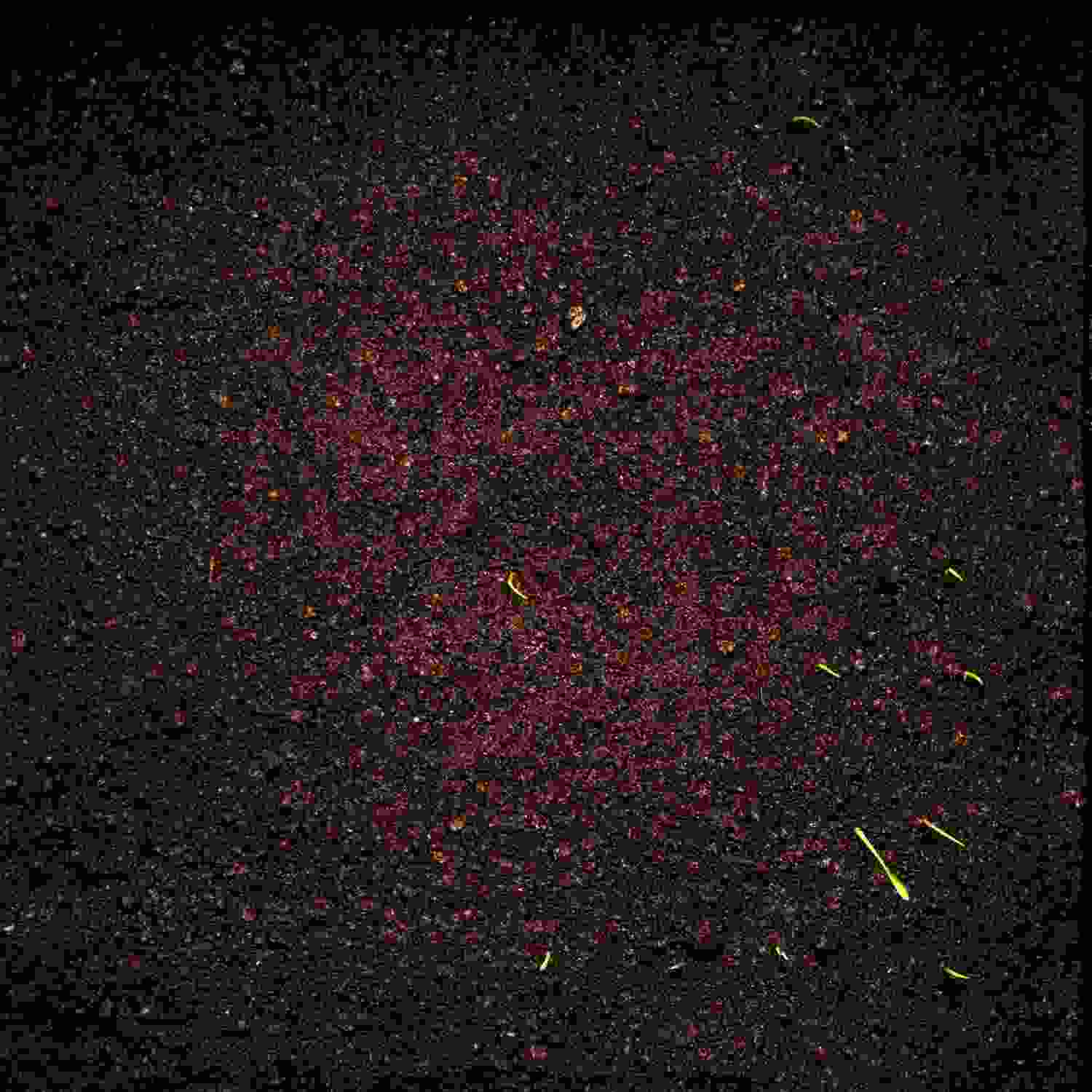

Supplement: Supplementary file 3 [file DataSheet3.zip › train1/5090-2024-3-18-22-14-15.JPG]

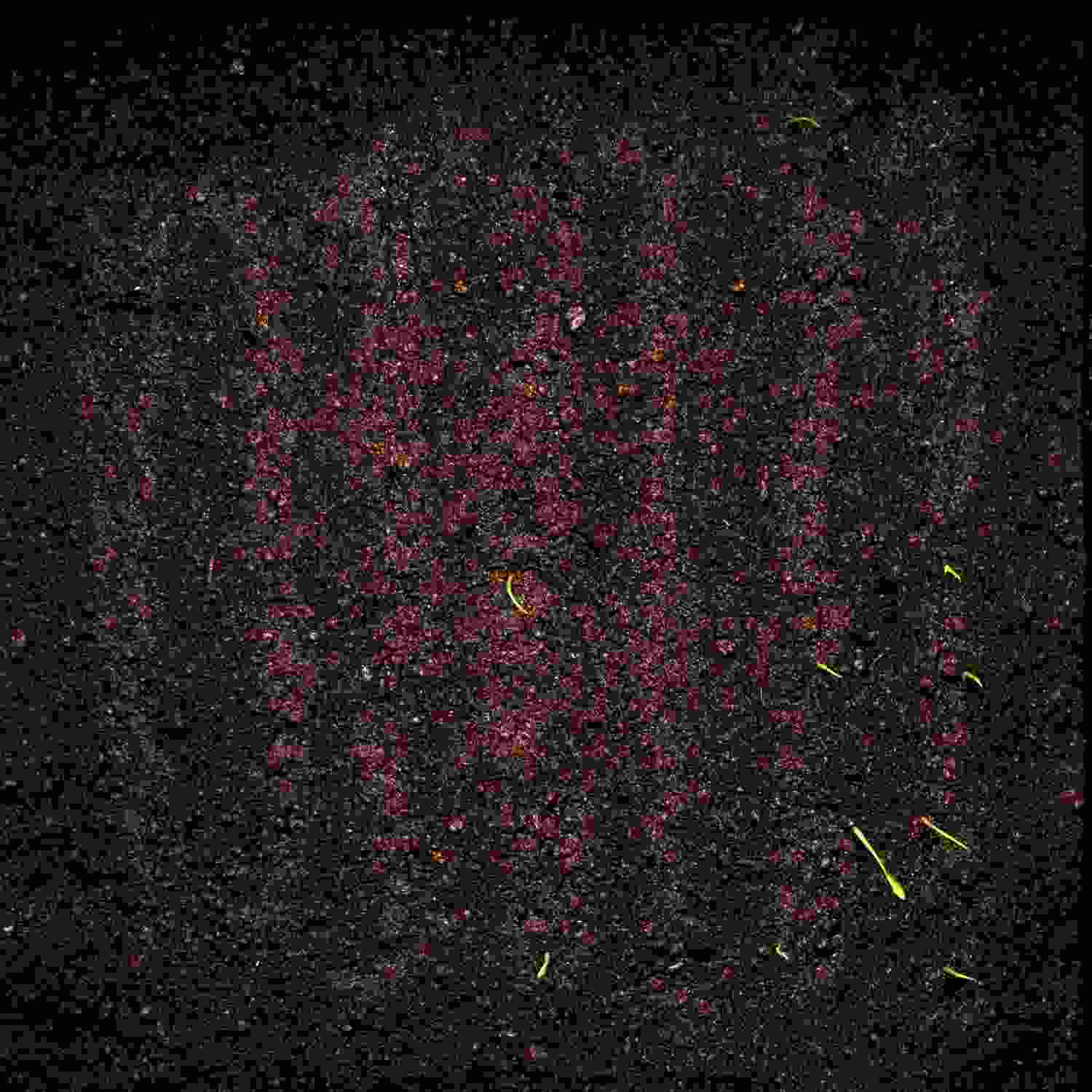

Supplement: Supplementary file 3 [file DataSheet3.zip › train1/5090-2024-3-19-0-43-8.JPG]

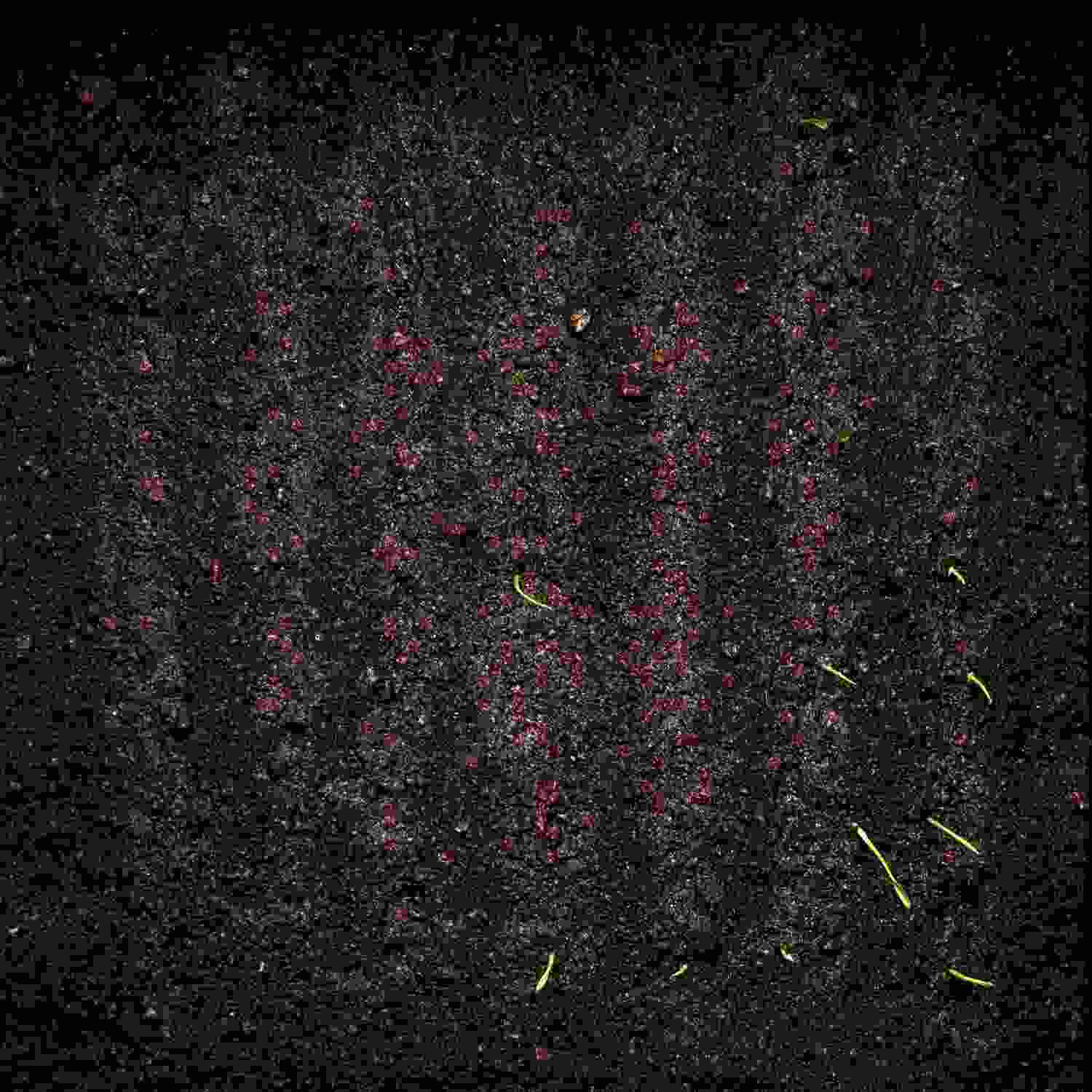

Supplement: Supplementary file 3 [file DataSheet3.zip › train1/5090-2024-3-19-10-39-7.JPG]

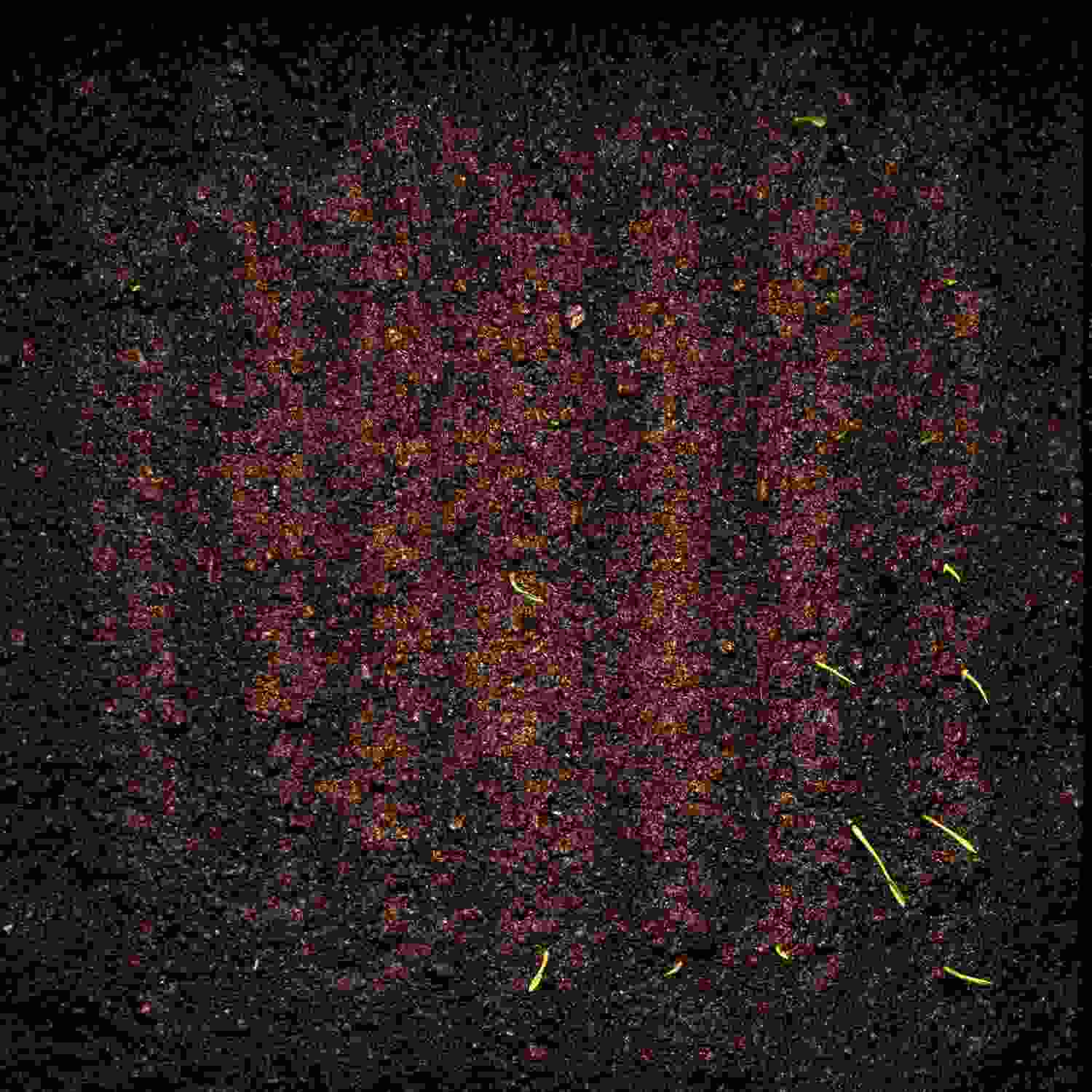

Supplement: Supplementary file 3 [file DataSheet3.zip › train1/5090-2024-3-19-13-8-21.JPG]

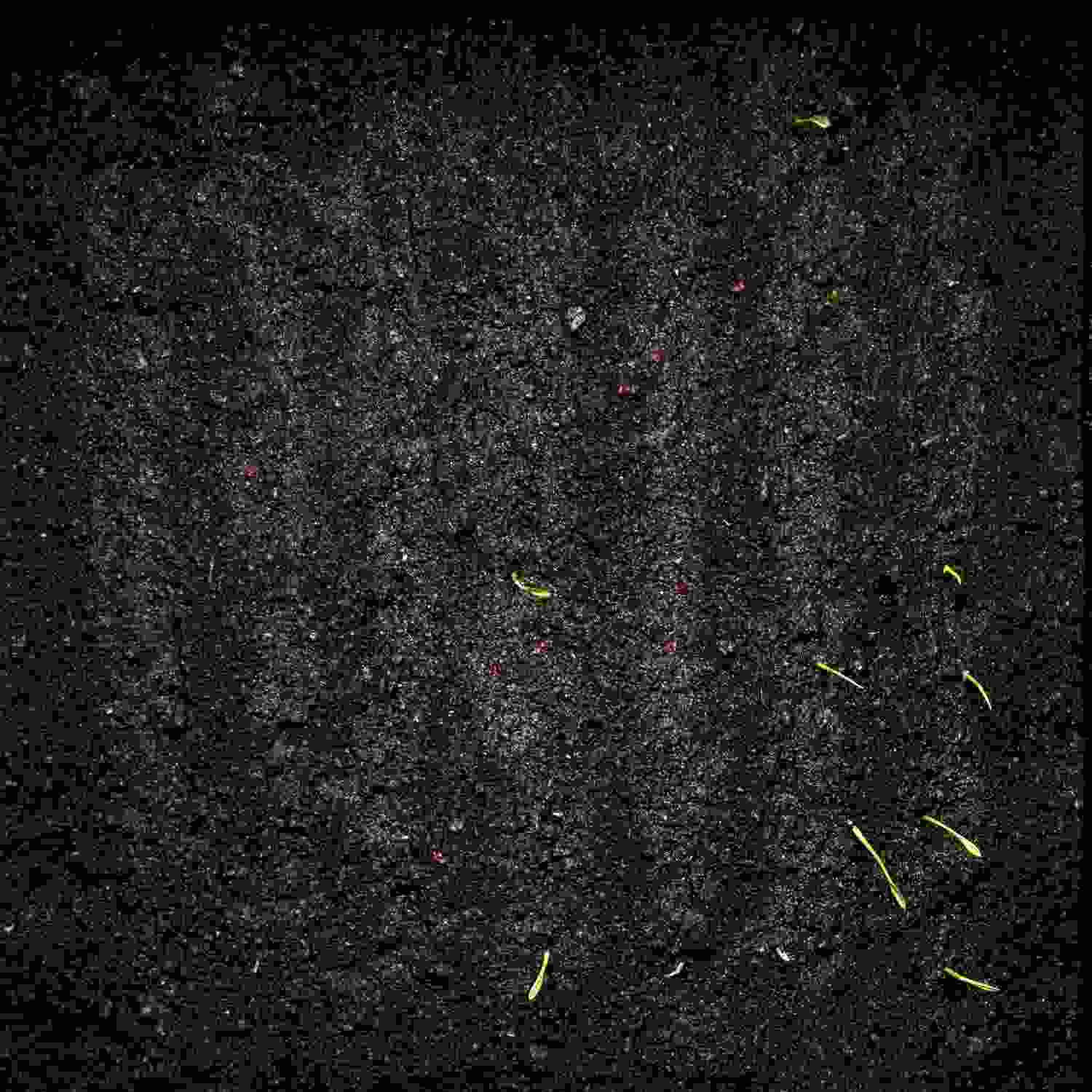

Supplement: Supplementary file 3 [file DataSheet3.zip › train1/5090-2024-3-19-18-5-51.JPG]

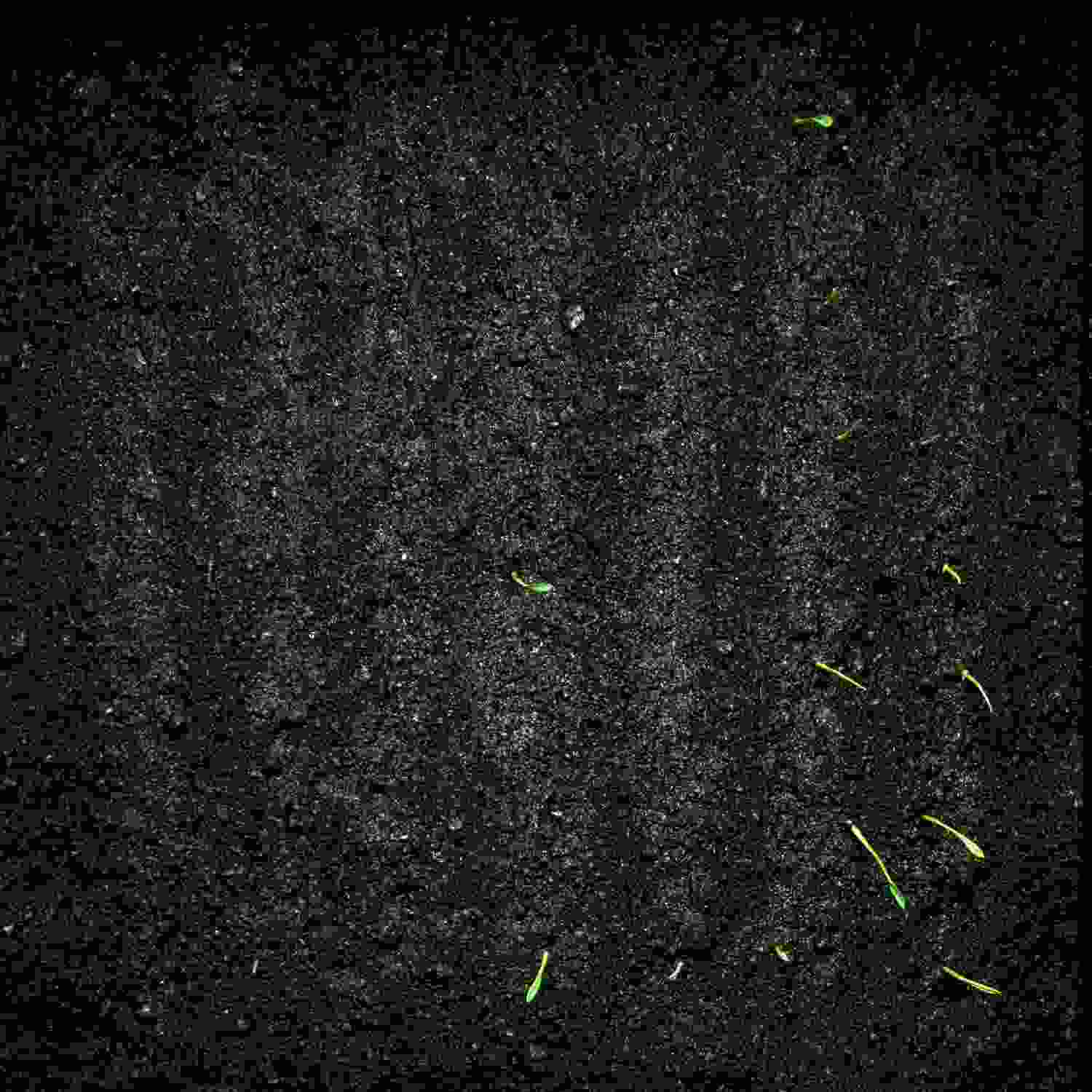

Supplement: Supplementary file 3 [file DataSheet3.zip › train1/5090-2024-3-19-20-34-53.JPG]

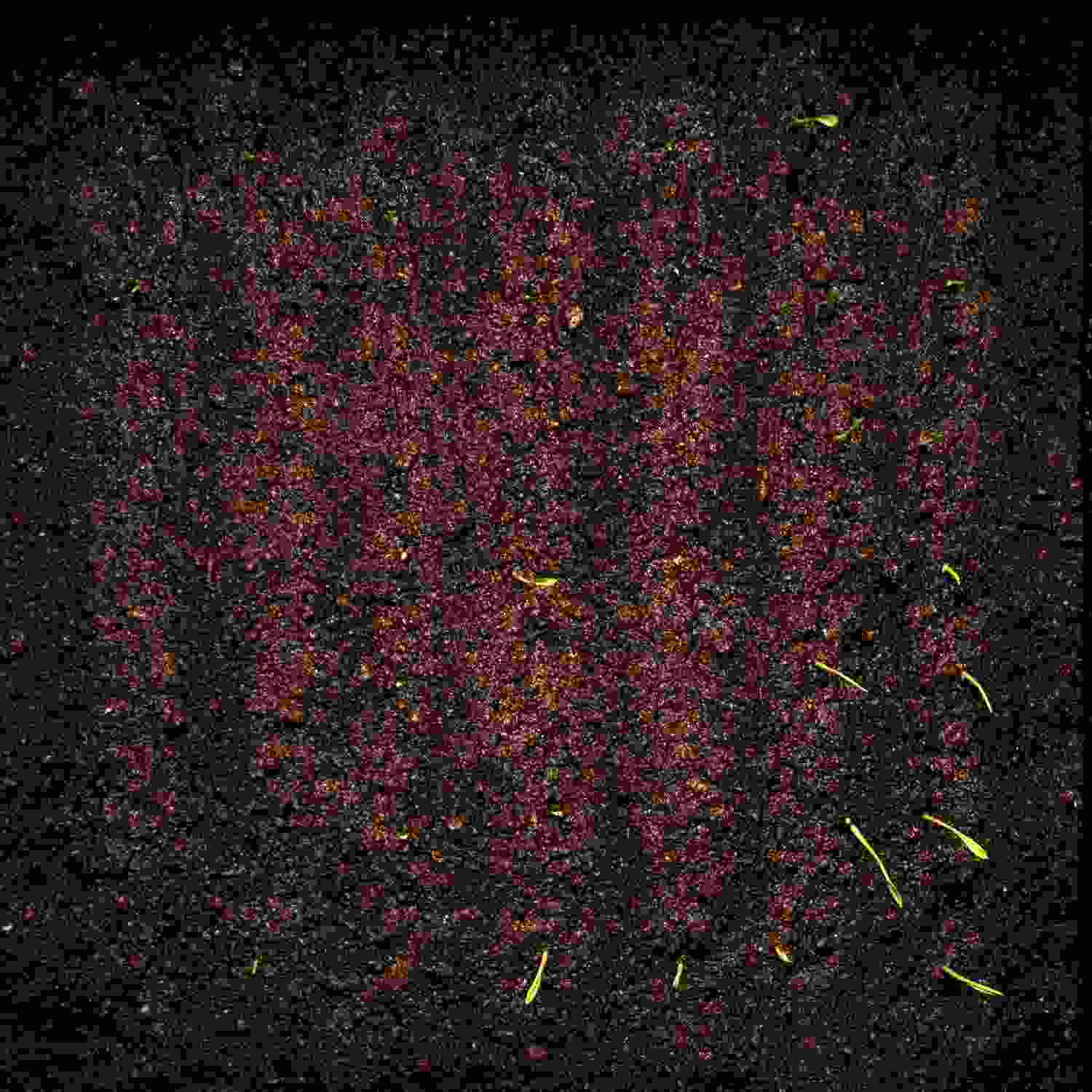

Supplement: Supplementary file 3 [file DataSheet3.zip › train1/5090-2024-3-19-23-4-14.JPG]

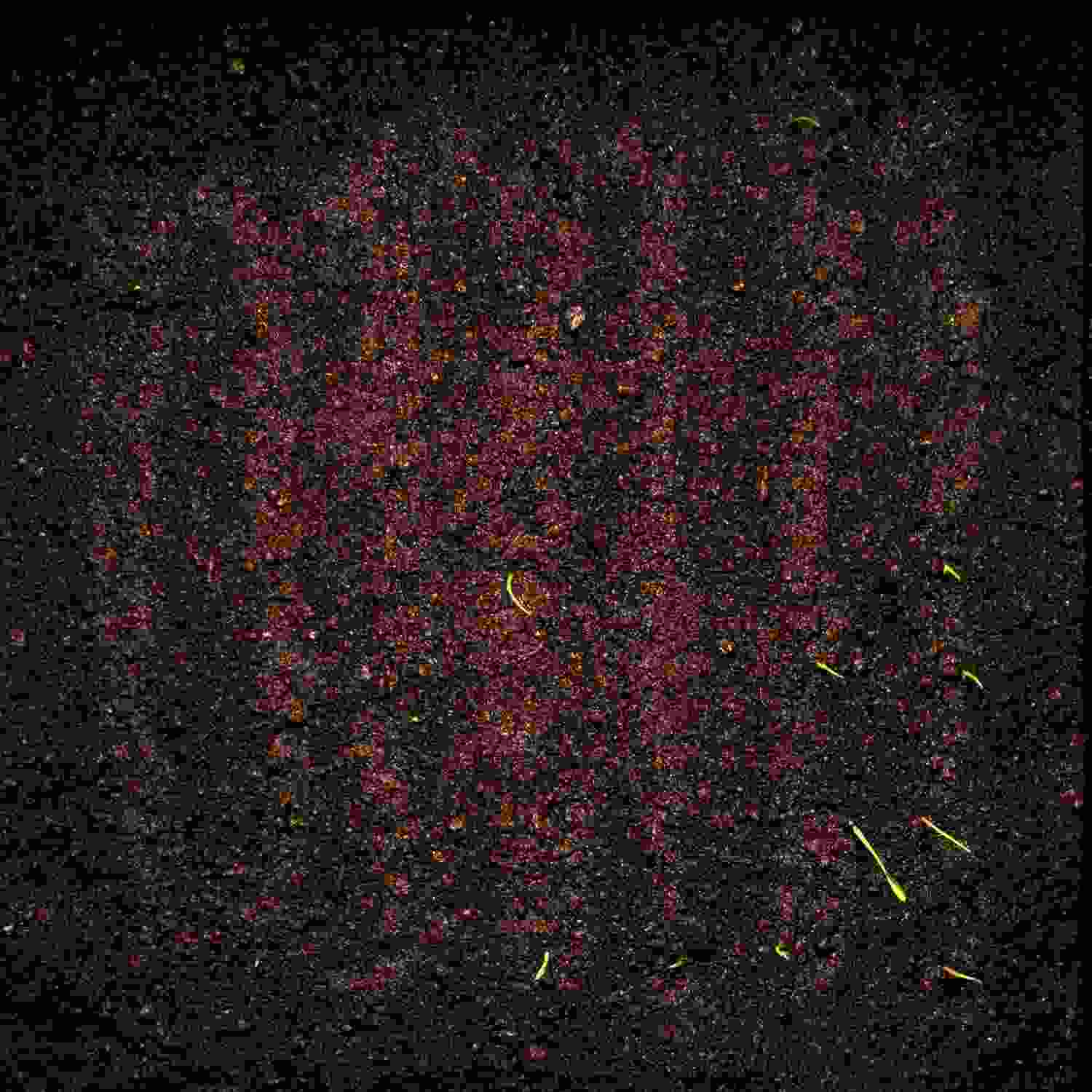

Supplement: Supplementary file 3 [file DataSheet3.zip › train1/5090-2024-3-19-3-12-4.JPG]

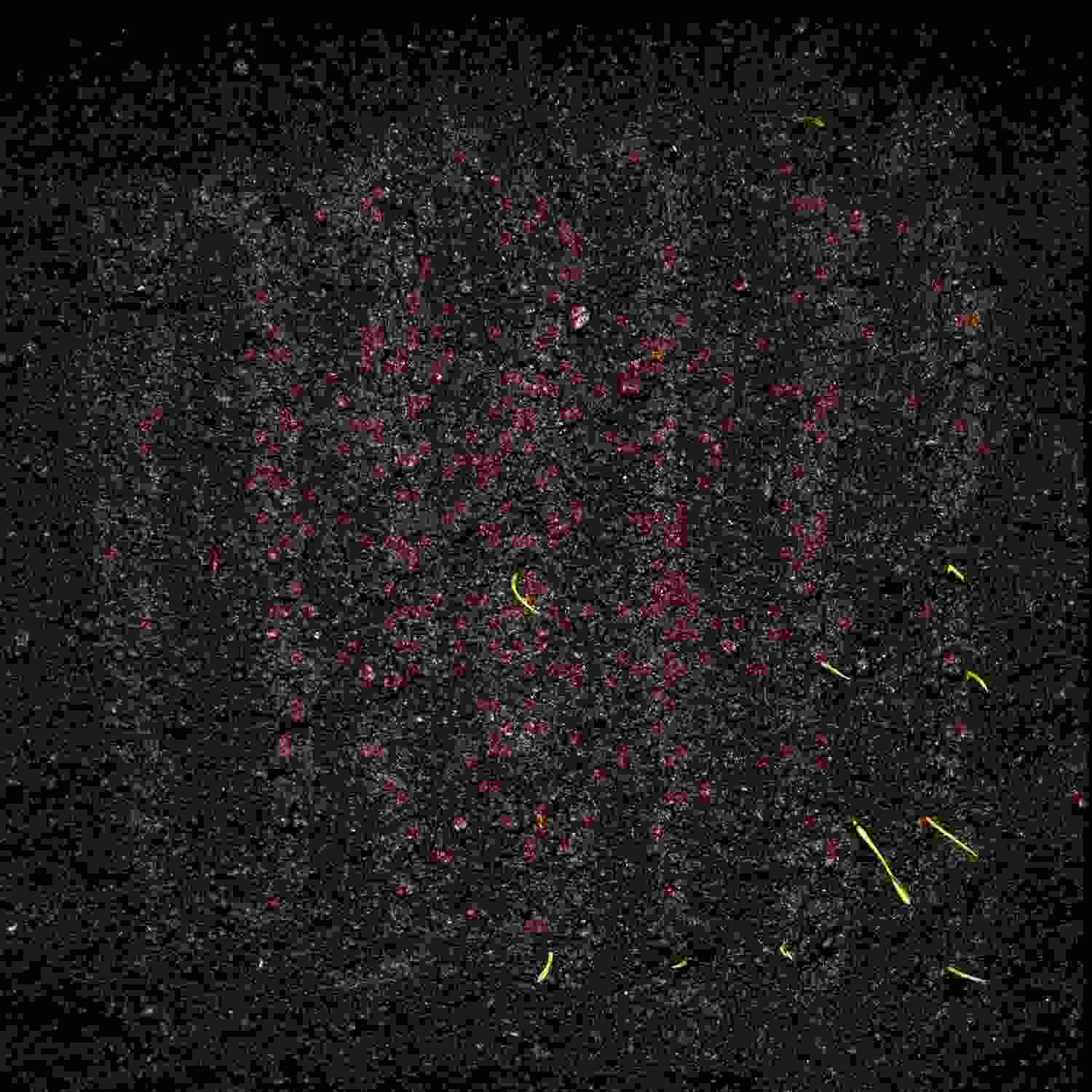

Supplement: Supplementary file 3 [file DataSheet3.zip › train1/5090-2024-3-19-5-40-27.JPG]

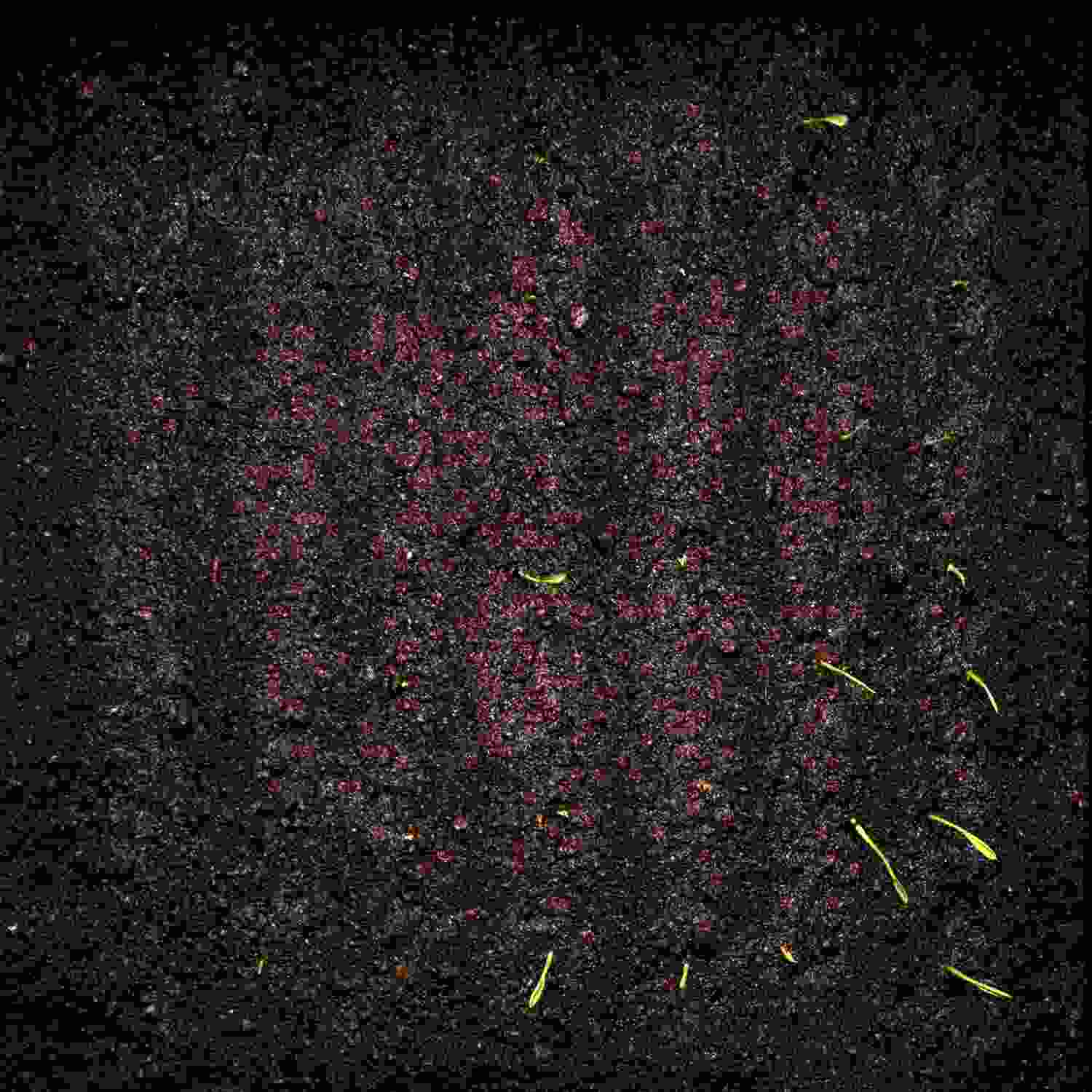

Supplement: Supplementary file 3 [file DataSheet3.zip › train1/5090-2024-3-20-1-33-17.JPG]

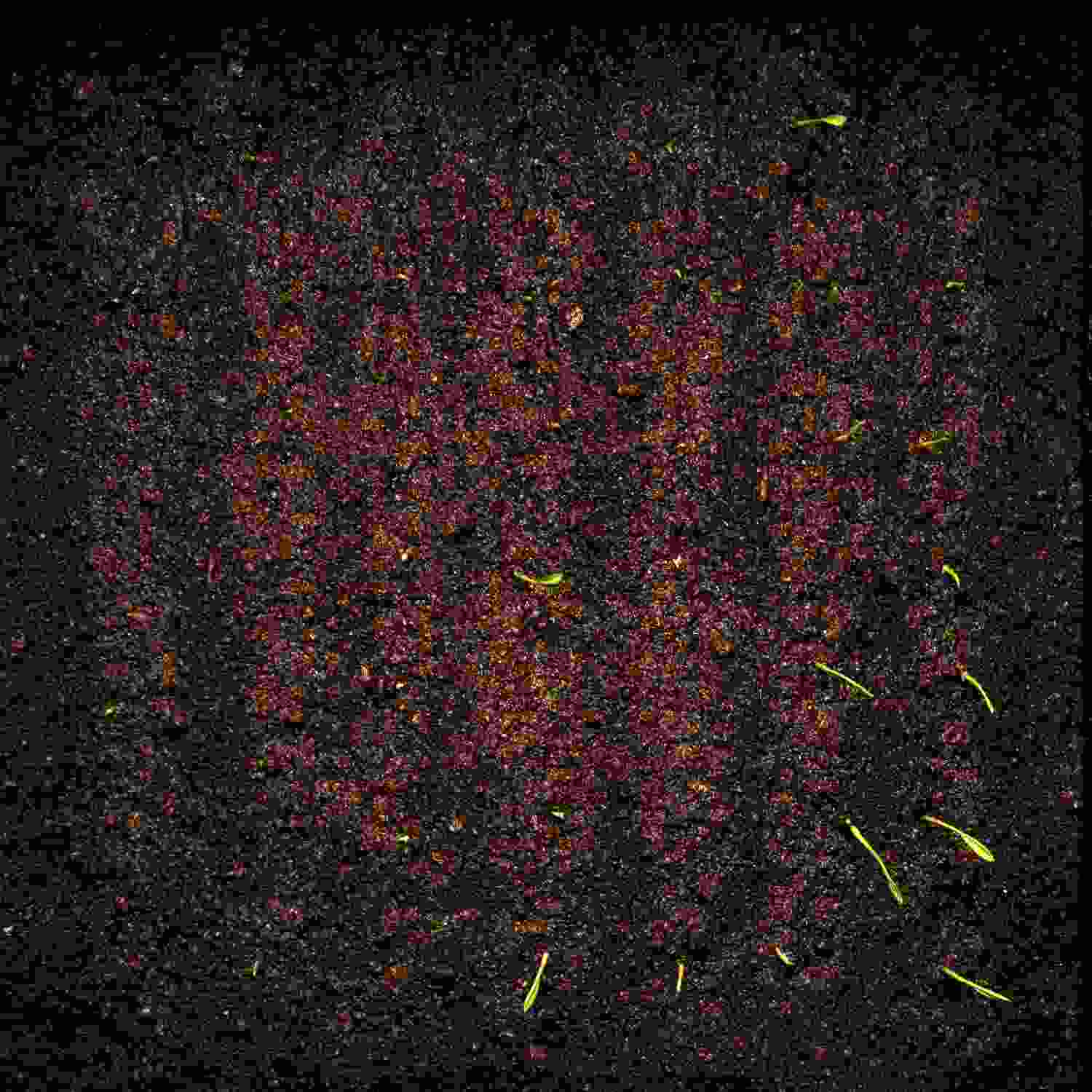

Supplement: Supplementary file 3 [file DataSheet3.zip › train1/5090-2024-3-20-4-1-41.JPG]

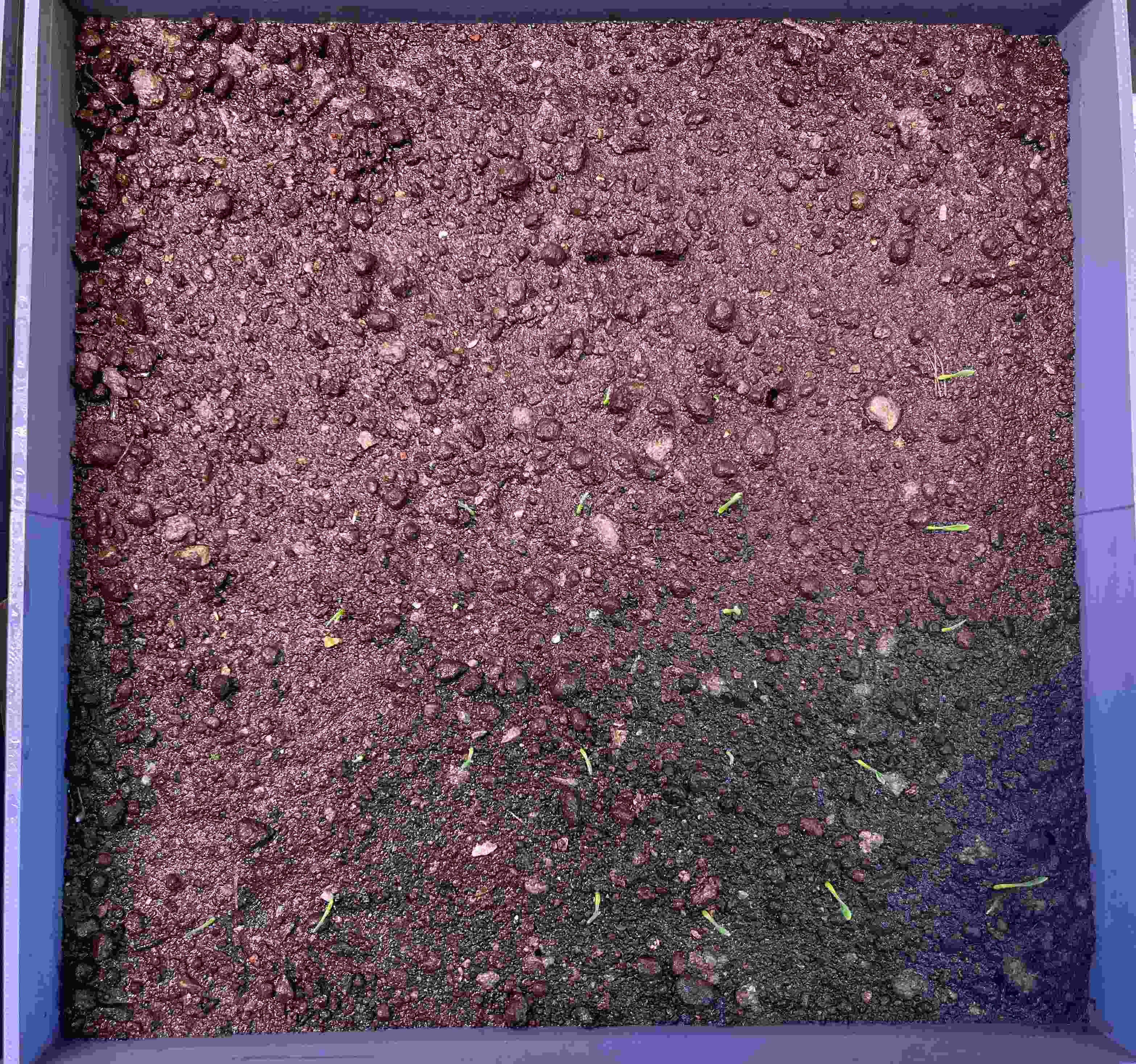

Supplement: Supplementary file 4 [file DataSheet4.zip › train/1-1.JPG]

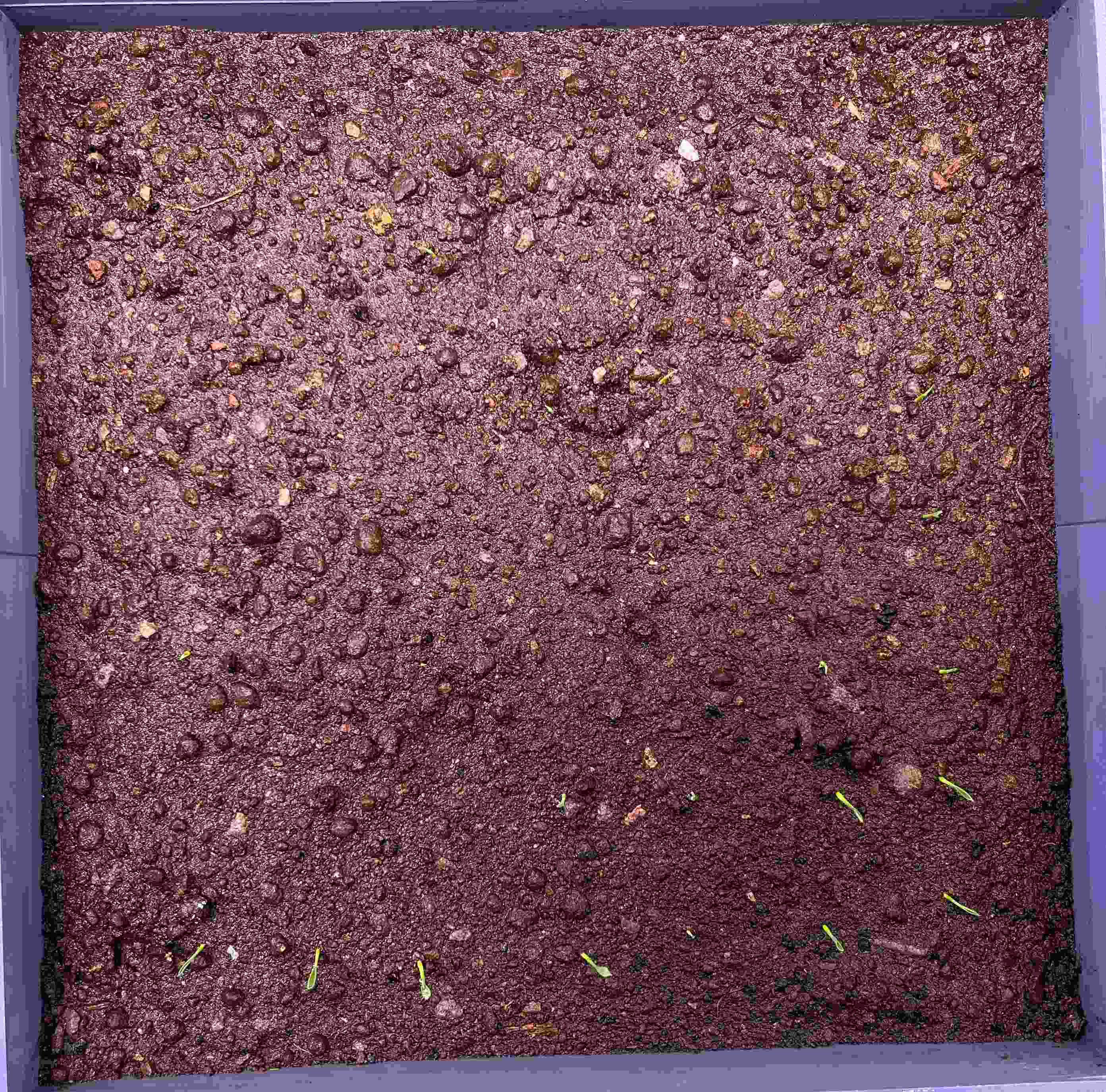

Supplement: Supplementary file 4 [file DataSheet4.zip › train/1-2.JPG]

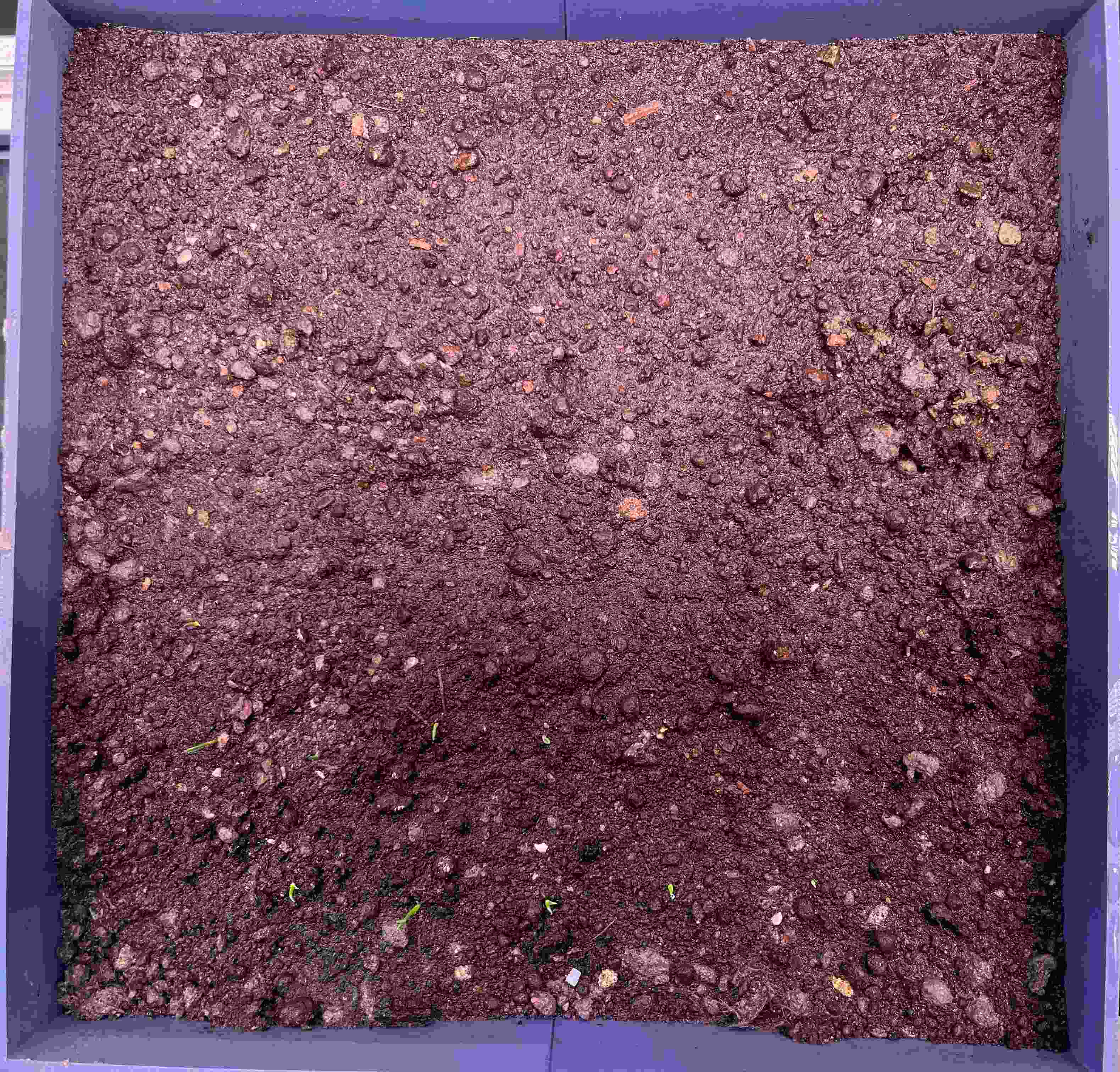

Supplement: Supplementary file 4 [file DataSheet4.zip › train/1-3.JPG]

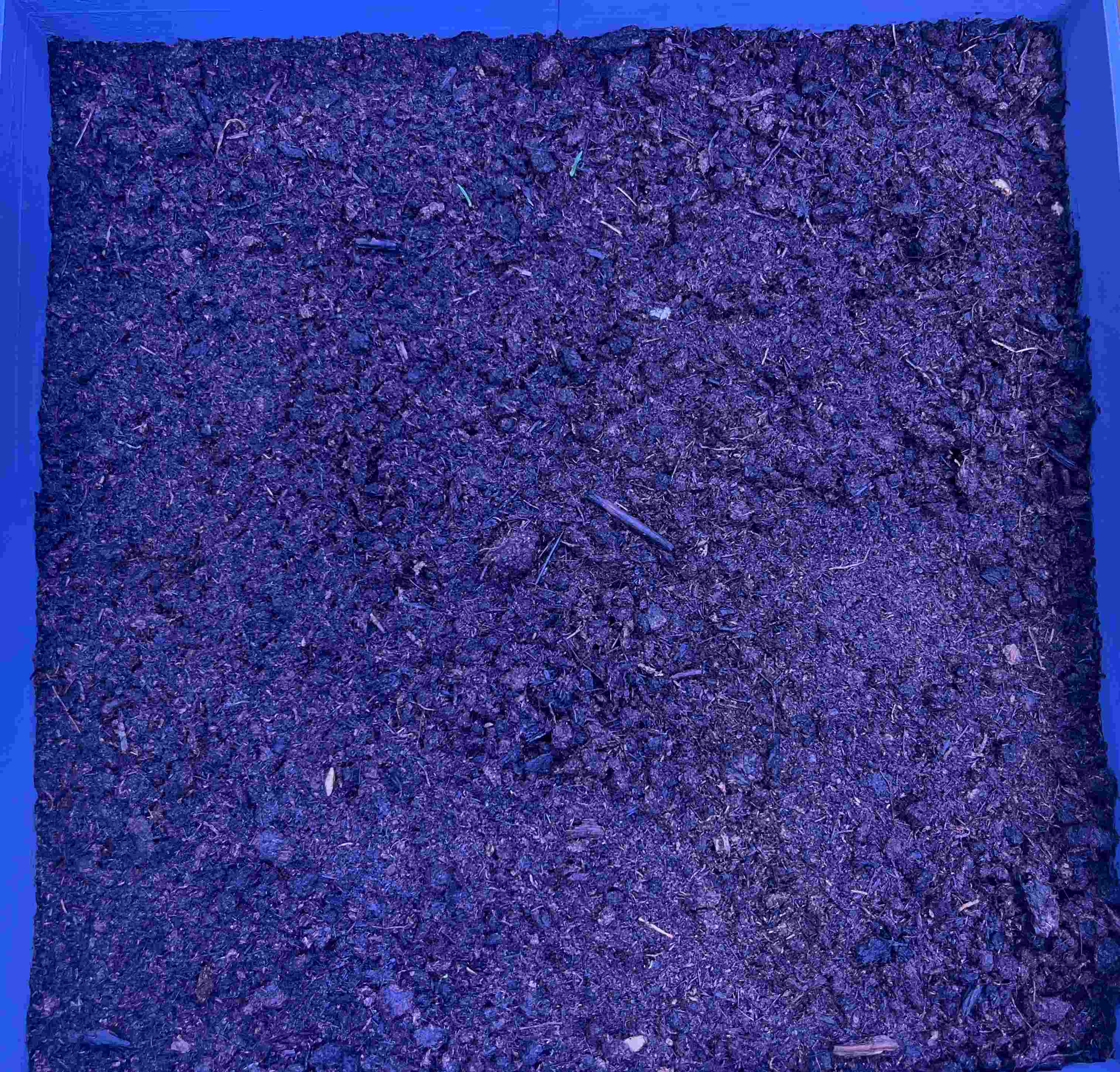

Supplement: Supplementary file 4 [file DataSheet4.zip › train/1-4.JPG]

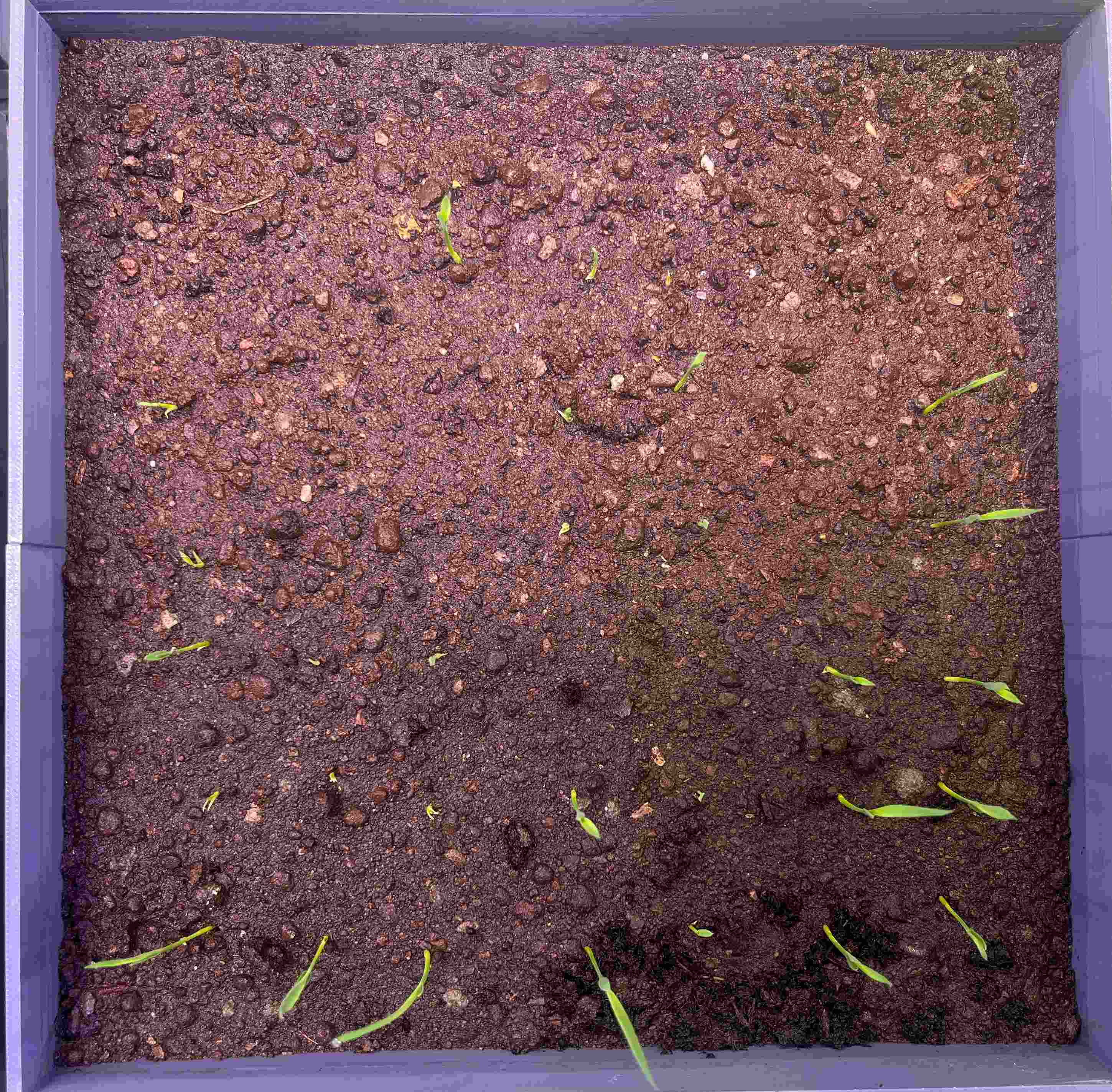

Supplement: Supplementary file 4 [file DataSheet4.zip › train/10-2.JPG]

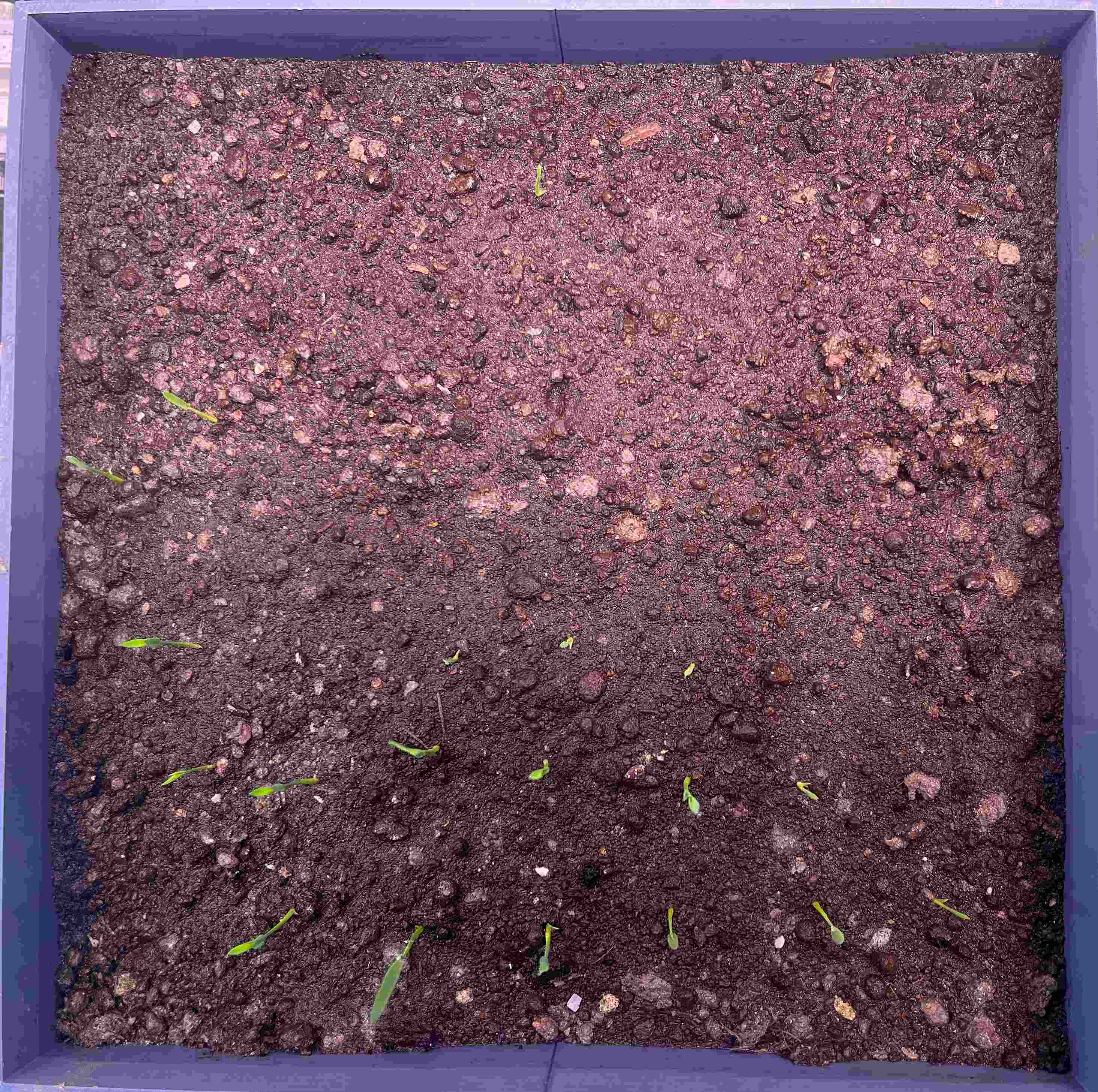

Supplement: Supplementary file 4 [file DataSheet4.zip › train/10-3.JPG]

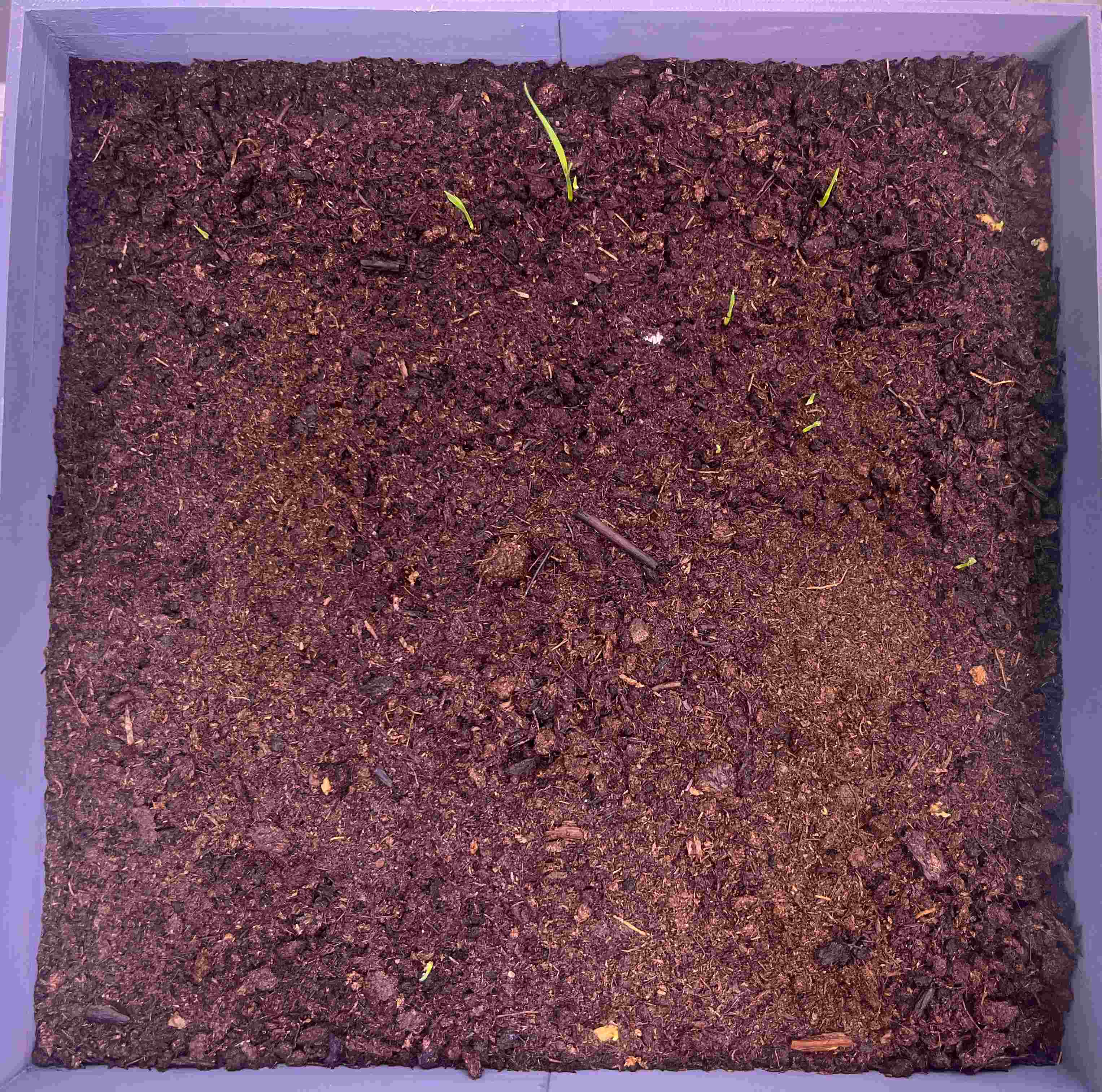

Supplement: Supplementary file 4 [file DataSheet4.zip › train/10-4.JPG]

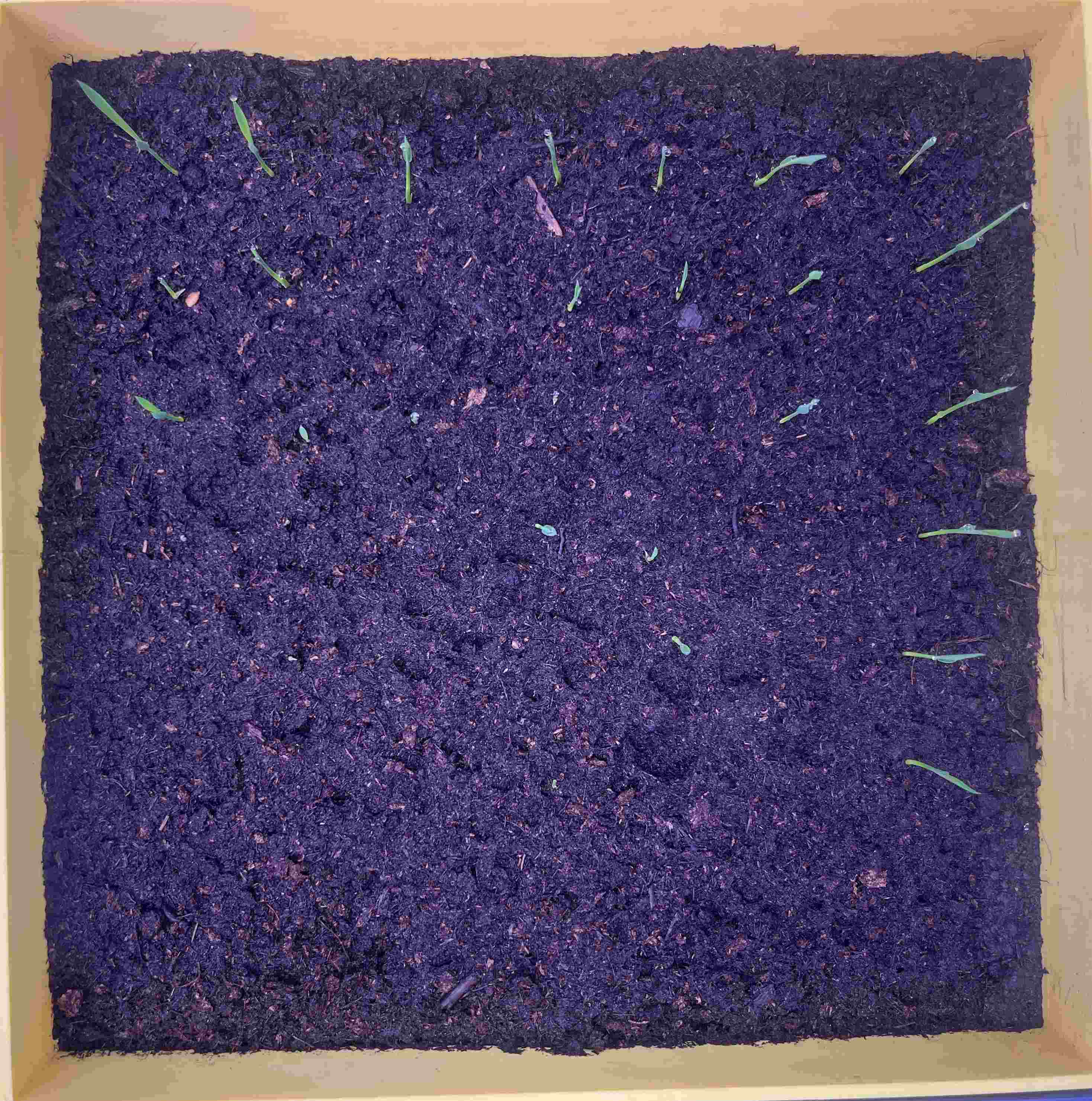

Supplement: Supplementary file 4 [file DataSheet4.zip › train/10-5.JPG]

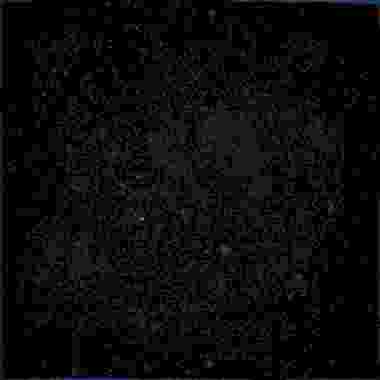

Supplement: Supplementary file 4 [file DataSheet4.zip › train/100120-2024-3-18-18-25-15.JPG]

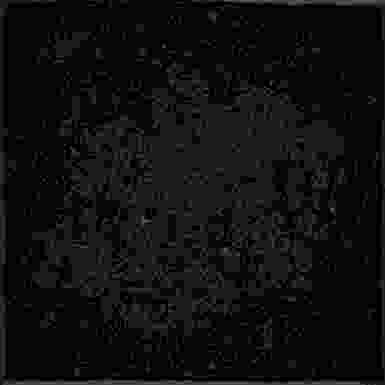

Supplement: Supplementary file 4 [file DataSheet4.zip › train/100120-2024-3-18-21-0-31.JPG]

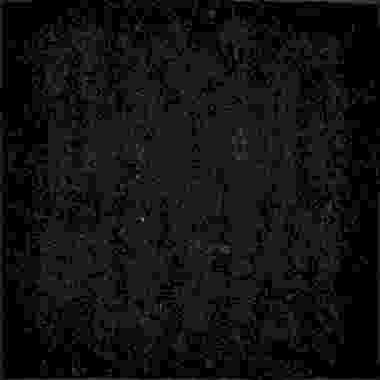

Supplement: Supplementary file 4 [file DataSheet4.zip › train/100120-2024-3-19-12-20-34.JPG]

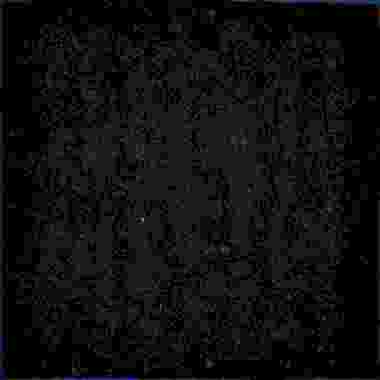

Supplement: Supplementary file 4 [file DataSheet4.zip › train/100120-2024-3-19-17-25-36.JPG]

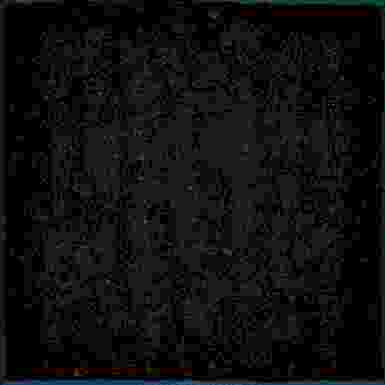

Supplement: Supplementary file 4 [file DataSheet4.zip › train/100120-2024-3-19-19-58-58.JPG]

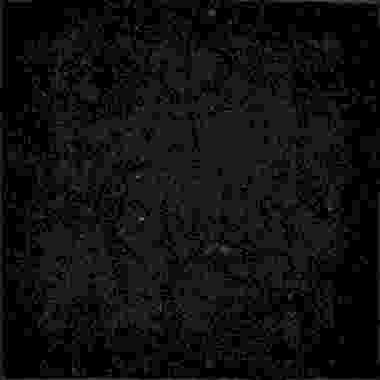

Supplement: Supplementary file 4 [file DataSheet4.zip › train/100120-2024-3-19-2-8-7.JPG]

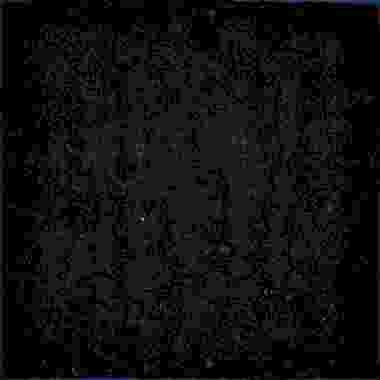

Supplement: Supplementary file 4 [file DataSheet4.zip › train/100120-2024-3-19-22-31-53.JPG]

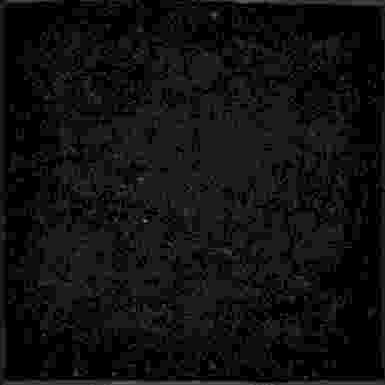

Supplement: Supplementary file 4 [file DataSheet4.zip › train/100120-2024-3-19-4-41-23.JPG]

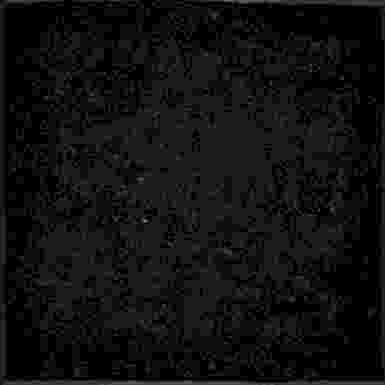

Supplement: Supplementary file 4 [file DataSheet4.zip › train/100120-2024-3-19-7-15-13.JPG]

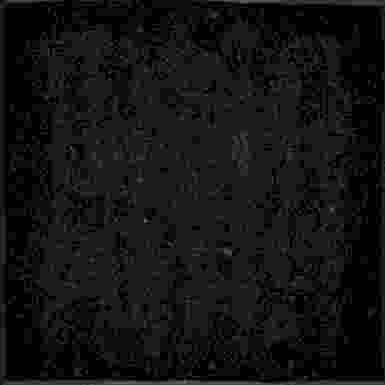

Supplement: Supplementary file 4 [file DataSheet4.zip › train/100120-2024-3-19-9-48-5.JPG]

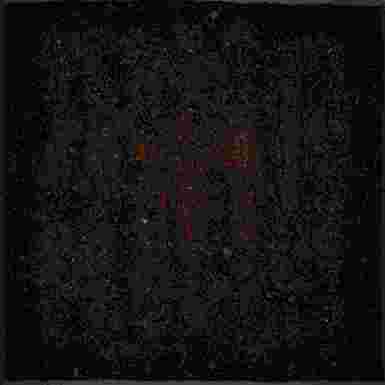

Supplement: Supplementary file 4 [file DataSheet4.zip › train/100120-2024-3-20-1-4-16.JPG]

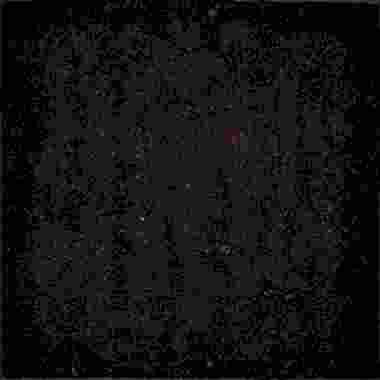

Supplement: Supplementary file 4 [file DataSheet4.zip › train/100120-2024-3-20-3-37-0.JPG]

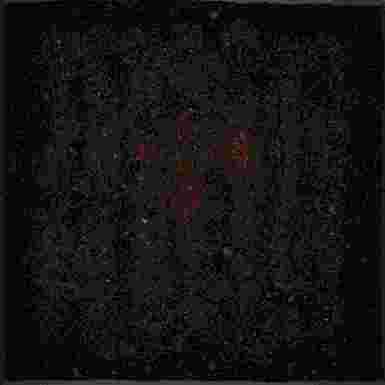

Supplement: Supplementary file 4 [file DataSheet4.zip › train/100120-2024-3-20-6-9-20.JPG]
