# Supplementary figures and images for: VanillaNet-YOLOv8 segment: detection of nano-iron oxide regulation on rice seedling growth vitality under salt stress (part 5 of 5)
Source: Front Plant Sci. 2025 Sep 17;16:1631279. doi: 10.3389/fpls.2025.1631279 (PMC12484053; doi:10.3389/fpls.2025.1631279)

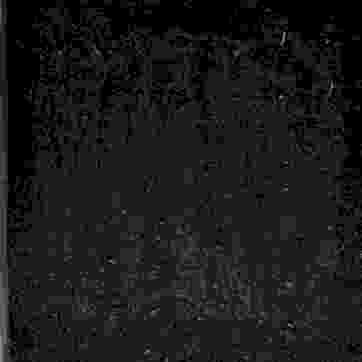

Supplement: Supplementary file 4 [file DataSheet4.zip › train/20060-2024-3-20-3-2-43.JPG]

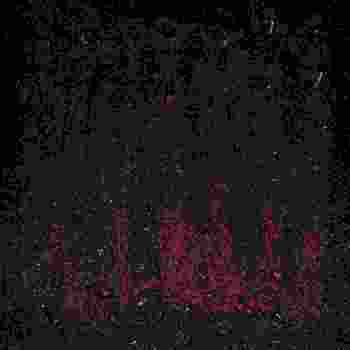

Supplement: Supplementary file 4 [file DataSheet4.zip › train/20060-2024-3-20-5-35-24.JPG]

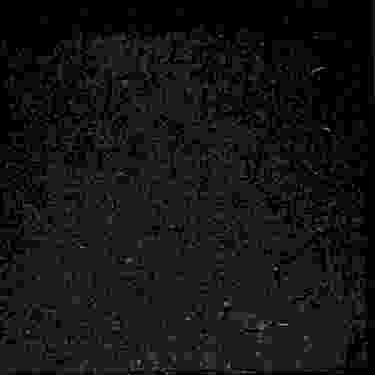

Supplement: Supplementary file 4 [file DataSheet4.zip › train/20090-2024-3-18-18-4-30.JPG]

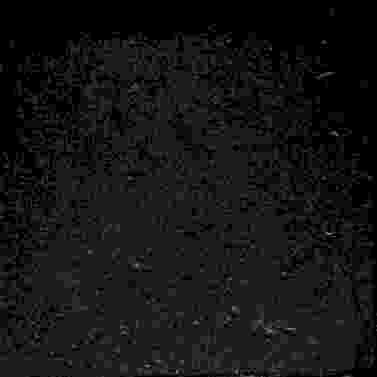

Supplement: Supplementary file 4 [file DataSheet4.zip › train/20090-2024-3-18-20-37-37.JPG]

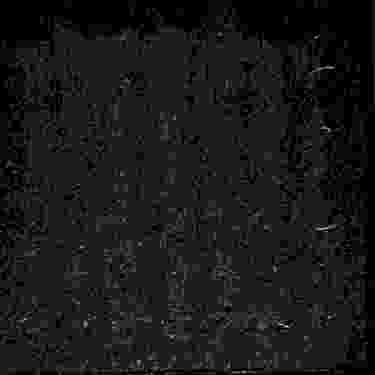

Supplement: Supplementary file 4 [file DataSheet4.zip › train/20090-2024-3-19-14-23-13.JPG]

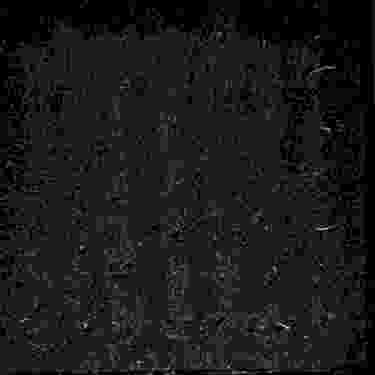

Supplement: Supplementary file 4 [file DataSheet4.zip › train/20090-2024-3-19-16-55-30.JPG]

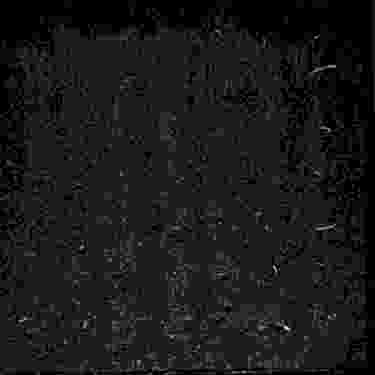

Supplement: Supplementary file 4 [file DataSheet4.zip › train/20090-2024-3-19-21-59-28.JPG]

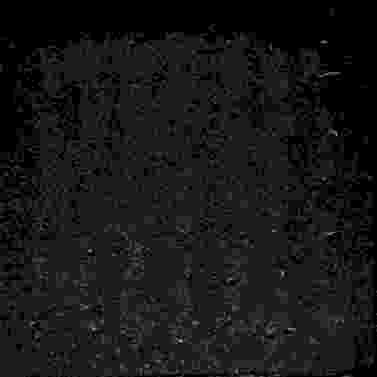

Supplement: Supplementary file 4 [file DataSheet4.zip › train/20090-2024-3-19-4-14-38.JPG]

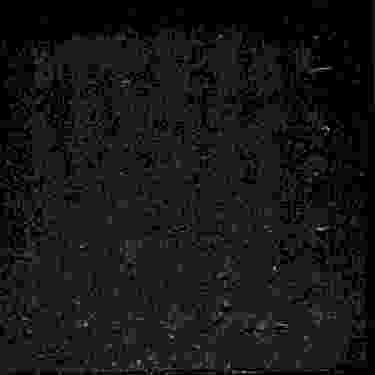

Supplement: Supplementary file 4 [file DataSheet4.zip › train/20090-2024-3-19-6-46-27.JPG]

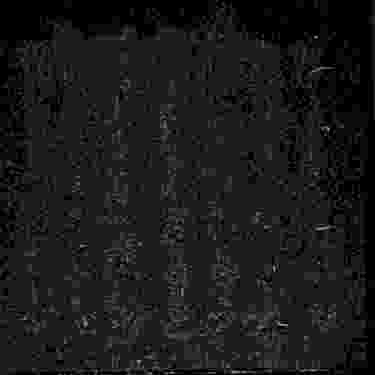

Supplement: Supplementary file 4 [file DataSheet4.zip › train/20090-2024-3-19-9-18-41.JPG]

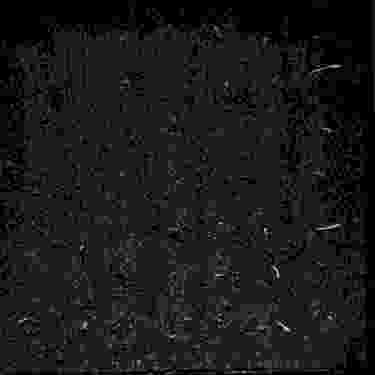

Supplement: Supplementary file 4 [file DataSheet4.zip › train/20090-2024-3-20-5-36-20.JPG]

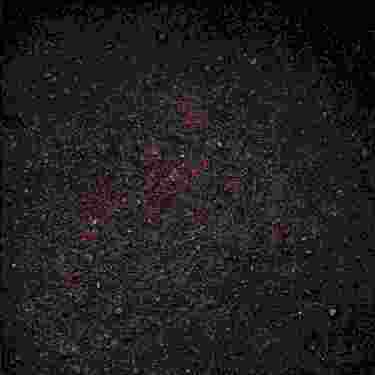

Supplement: Supplementary file 4 [file DataSheet4.zip › train/20120-2024-3-18-18-45-6.JPG]

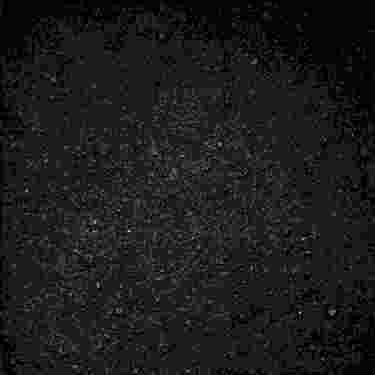

Supplement: Supplementary file 4 [file DataSheet4.zip › train/20120-2024-3-18-21-38-55.JPG]

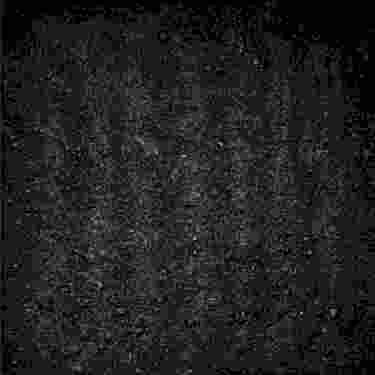

Supplement: Supplementary file 4 [file DataSheet4.zip › train/20120-2024-3-19-14-49-9.JPG]

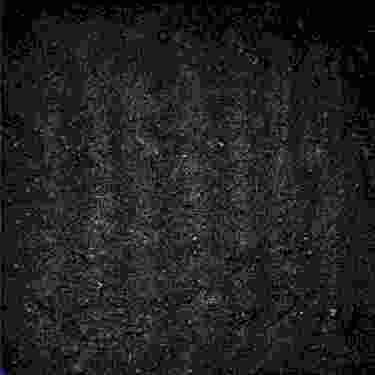

Supplement: Supplementary file 4 [file DataSheet4.zip › train/20120-2024-3-19-17-40-24.JPG]

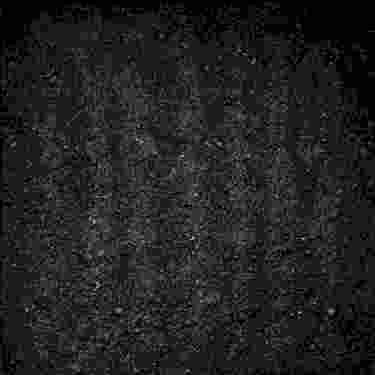

Supplement: Supplementary file 4 [file DataSheet4.zip › train/20120-2024-3-19-20-31-11.JPG]

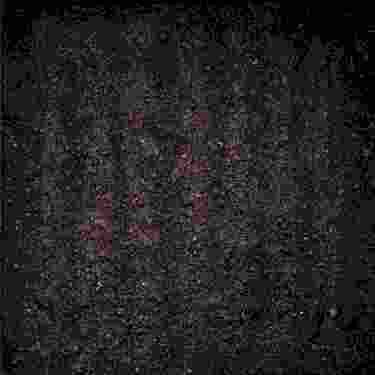

Supplement: Supplementary file 4 [file DataSheet4.zip › train/20120-2024-3-19-23-22-46.JPG]

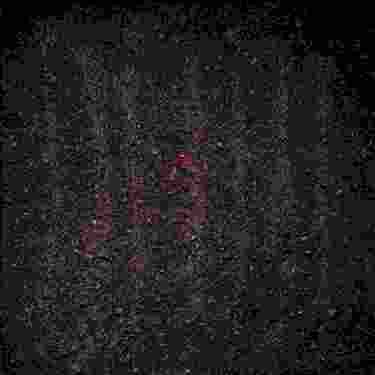

Supplement: Supplementary file 4 [file DataSheet4.zip › train/20120-2024-3-19-3-23-9.JPG]

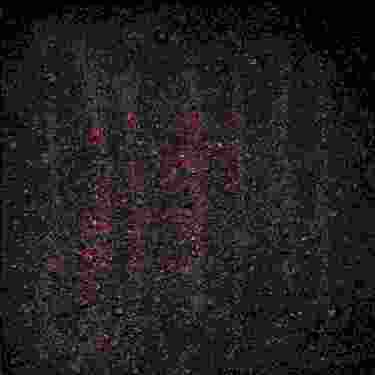

Supplement: Supplementary file 4 [file DataSheet4.zip › train/20120-2024-3-19-6-14-54.JPG]

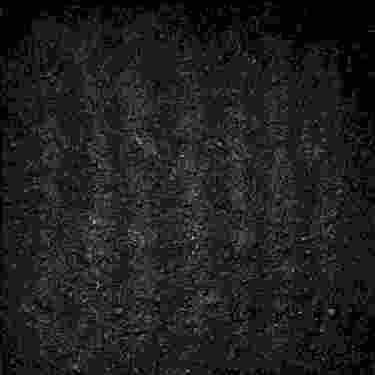

Supplement: Supplementary file 4 [file DataSheet4.zip › train/20120-2024-3-19-9-6-45.JPG]

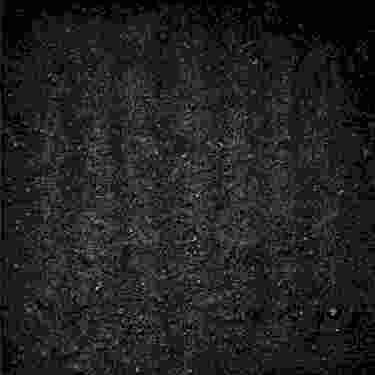

Supplement: Supplementary file 4 [file DataSheet4.zip › train/20120-2024-3-20-10-47-30.JPG]

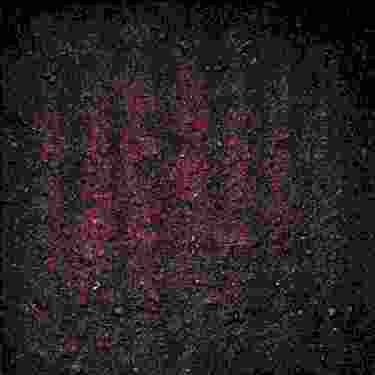

Supplement: Supplementary file 4 [file DataSheet4.zip › train/20120-2024-3-20-13-38-31.JPG]

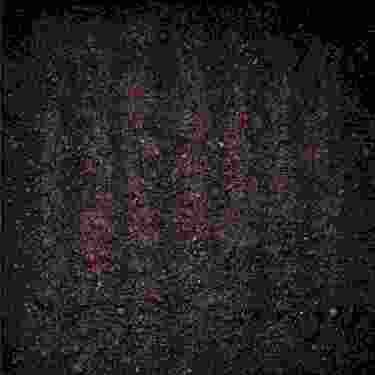

Supplement: Supplementary file 4 [file DataSheet4.zip › train/20120-2024-3-20-2-14-23.JPG]

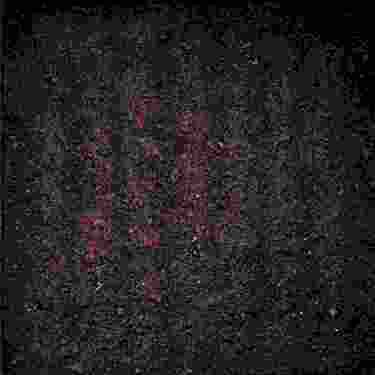

Supplement: Supplementary file 4 [file DataSheet4.zip › train/20120-2024-3-20-7-56-14.JPG]

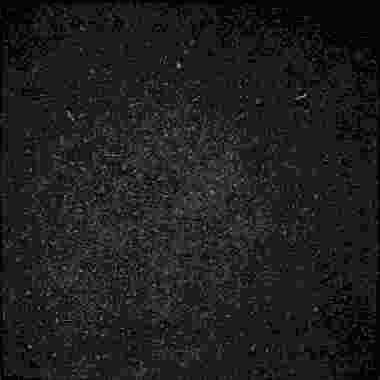

Supplement: Supplementary file 4 [file DataSheet4.zip › train/20150-2024-3-18-17-48-8.JPG]

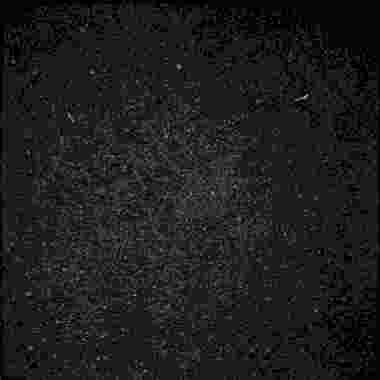

Supplement: Supplementary file 4 [file DataSheet4.zip › train/20150-2024-3-18-20-22-32.JPG]

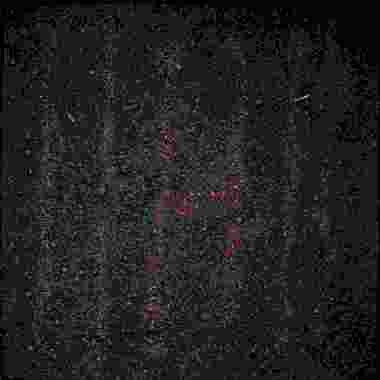

Supplement: Supplementary file 4 [file DataSheet4.zip › train/20150-2024-3-18-22-56-7.JPG]

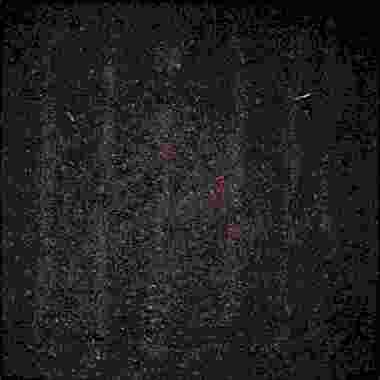

Supplement: Supplementary file 4 [file DataSheet4.zip › train/20150-2024-3-19-1-29-22.JPG]

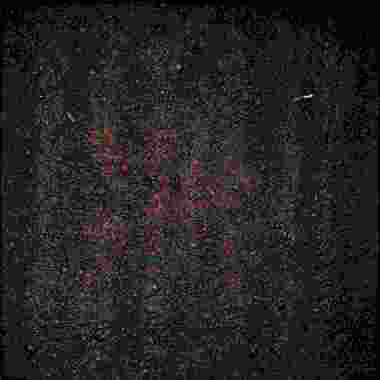

Supplement: Supplementary file 4 [file DataSheet4.zip › train/20150-2024-3-19-11-40-0.JPG]

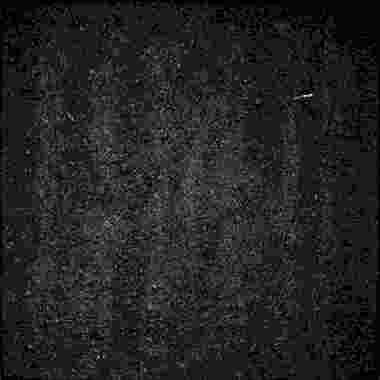

Supplement: Supplementary file 4 [file DataSheet4.zip › train/20150-2024-3-19-14-11-40.JPG]

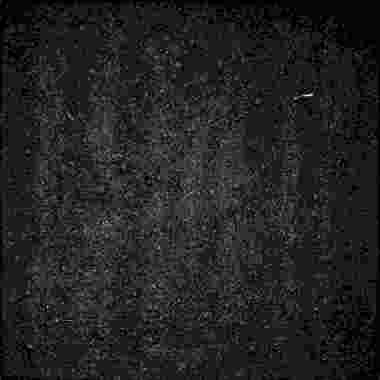

Supplement: Supplementary file 4 [file DataSheet4.zip › train/20150-2024-3-19-16-43-56.JPG]

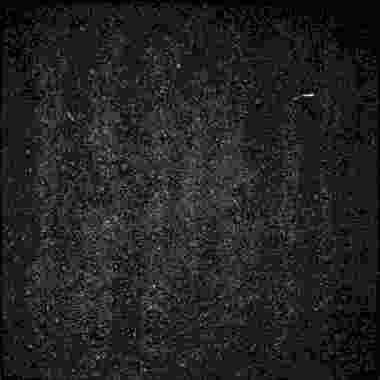

Supplement: Supplementary file 4 [file DataSheet4.zip › train/20150-2024-3-19-19-15-45.JPG]

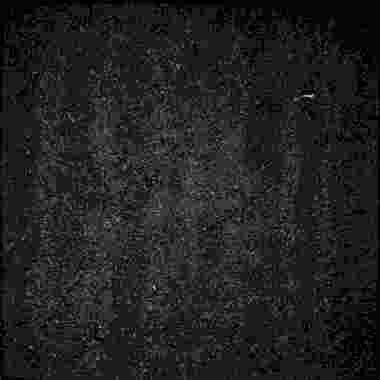

Supplement: Supplementary file 4 [file DataSheet4.zip › train/20150-2024-3-19-21-47-34.JPG]

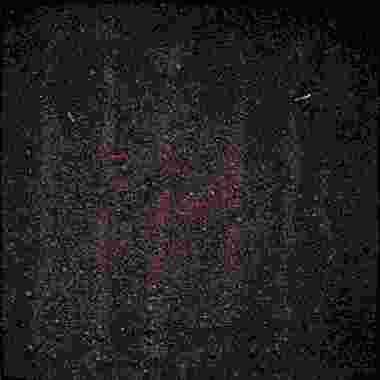

Supplement: Supplementary file 4 [file DataSheet4.zip › train/20150-2024-3-19-4-1-49.JPG]

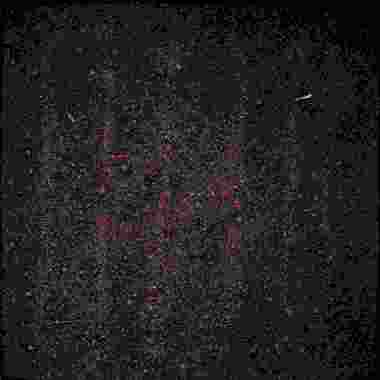

Supplement: Supplementary file 4 [file DataSheet4.zip › train/20150-2024-3-19-6-34-32.JPG]

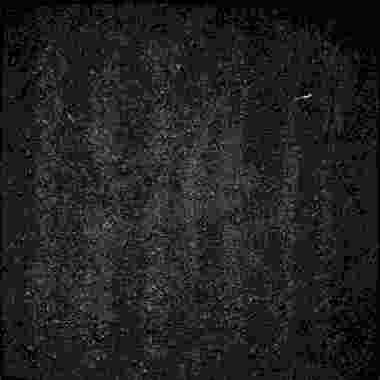

Supplement: Supplementary file 4 [file DataSheet4.zip › train/20150-2024-3-19-9-7-15.JPG]

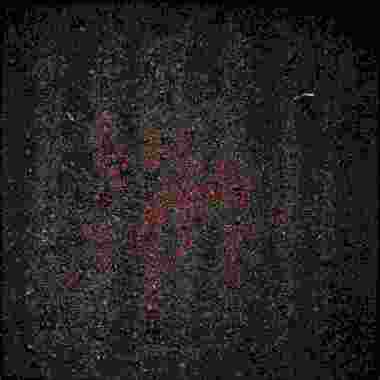

Supplement: Supplementary file 4 [file DataSheet4.zip › train/20150-2024-3-20-0-20-45.JPG]

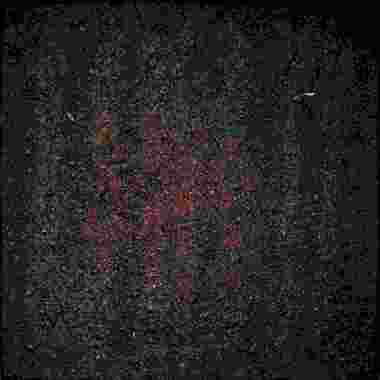

Supplement: Supplementary file 4 [file DataSheet4.zip › train/20150-2024-3-20-2-52-47.JPG]

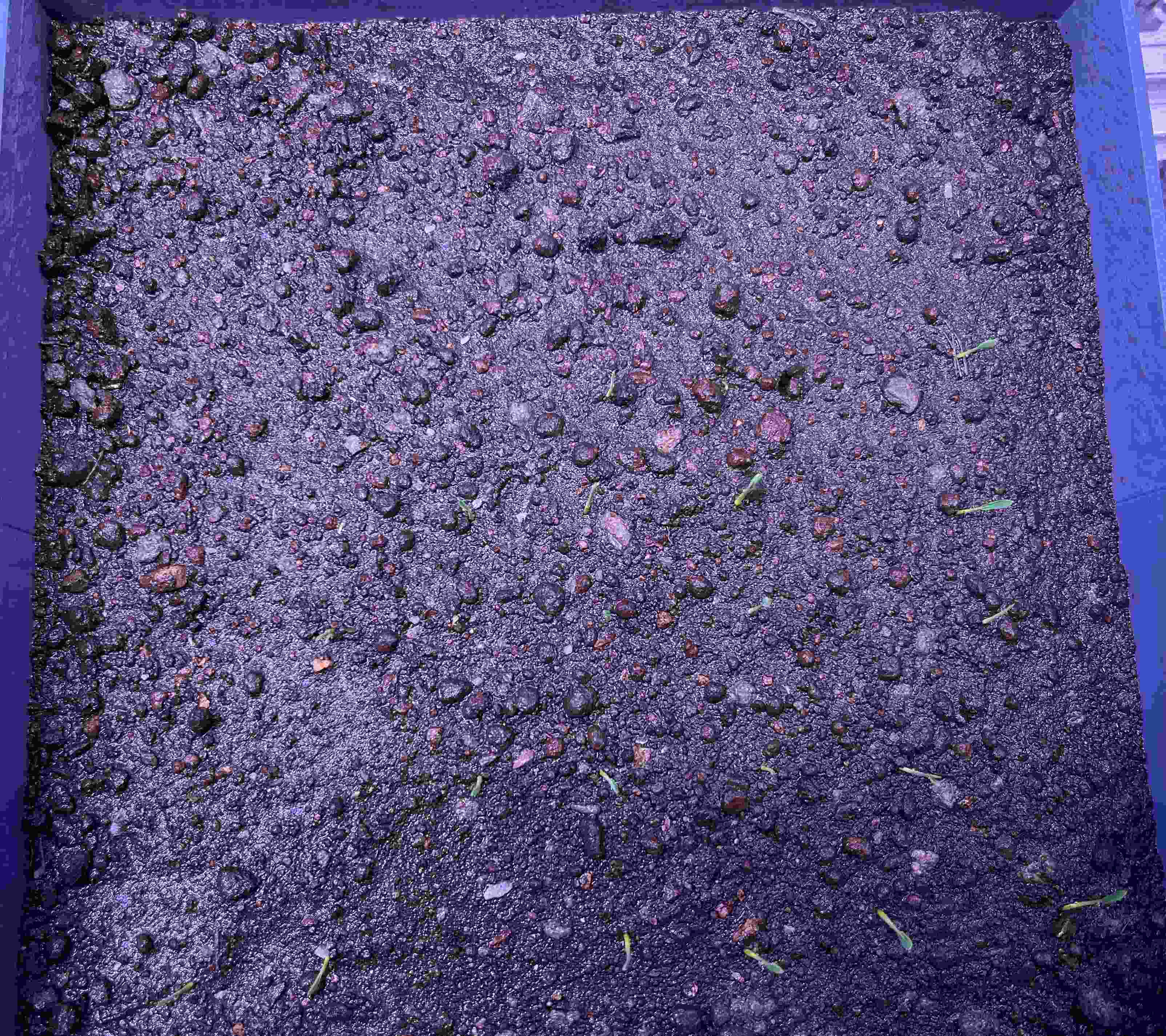

Supplement: Supplementary file 4 [file DataSheet4.zip › train/3-1.JPG]

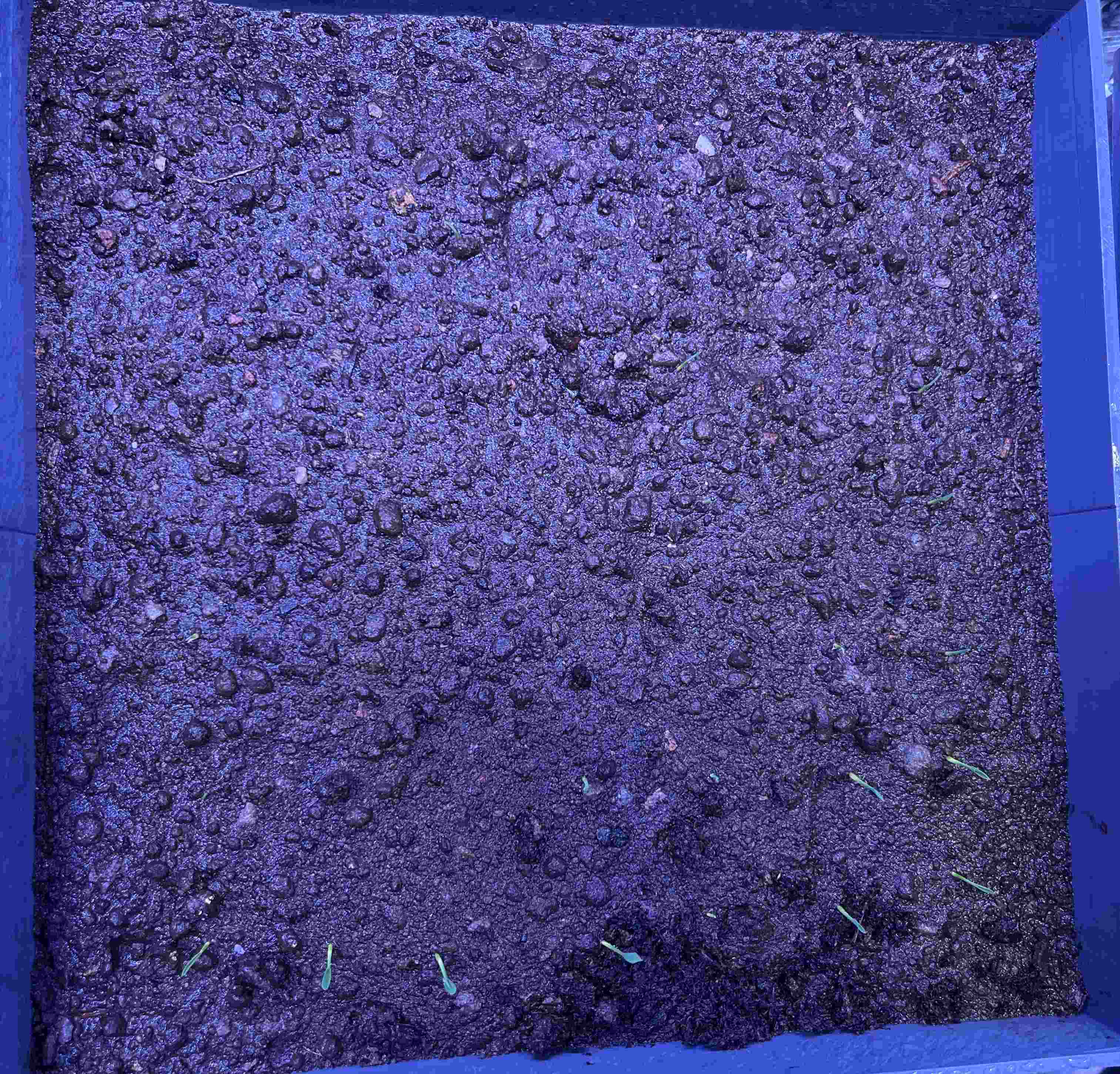

Supplement: Supplementary file 4 [file DataSheet4.zip › train/3-2.JPG]

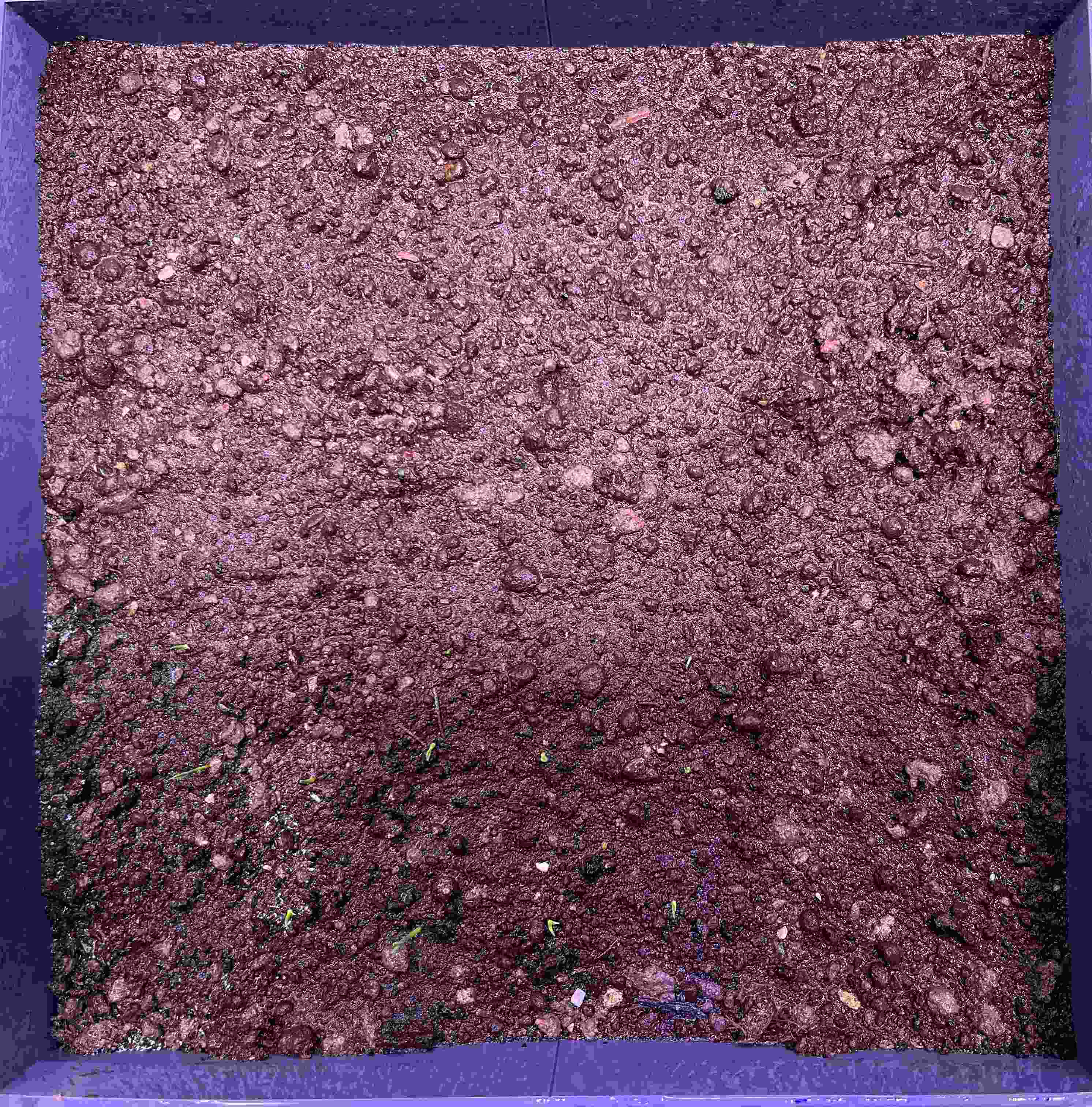

Supplement: Supplementary file 4 [file DataSheet4.zip › train/3-3.JPG]

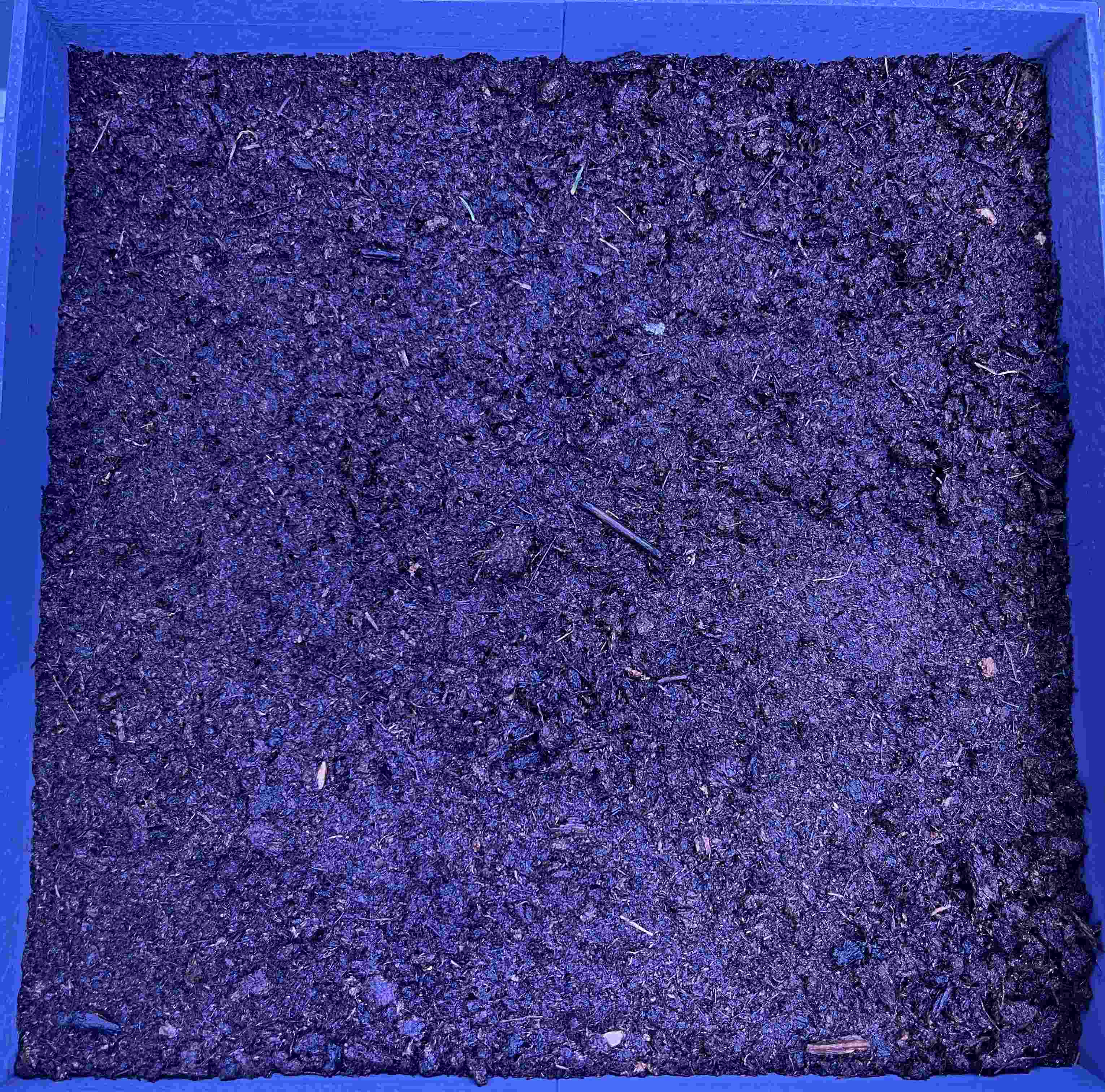

Supplement: Supplementary file 4 [file DataSheet4.zip › train/3-4.JPG]

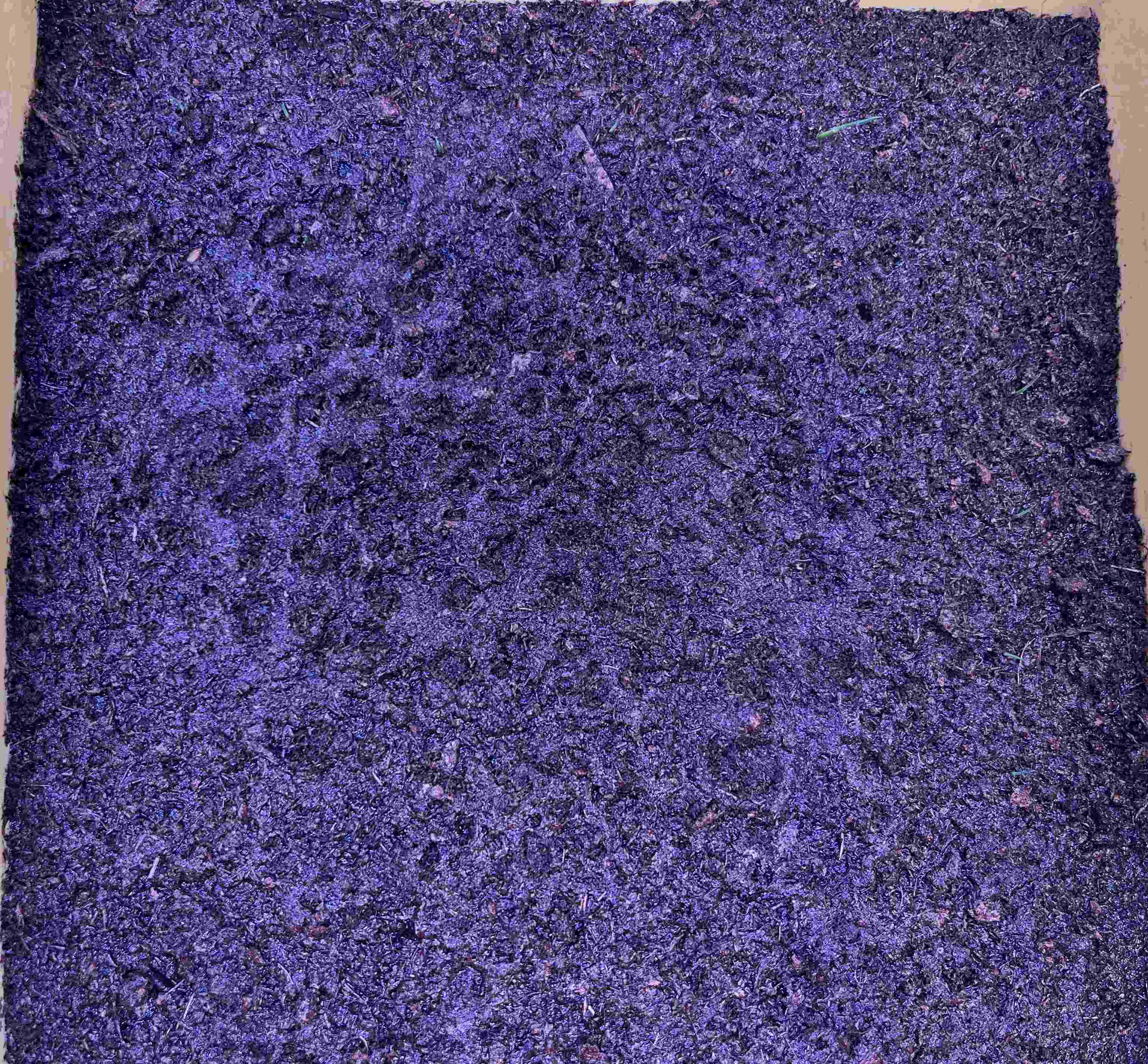

Supplement: Supplementary file 4 [file DataSheet4.zip › train/3-5.JPG]

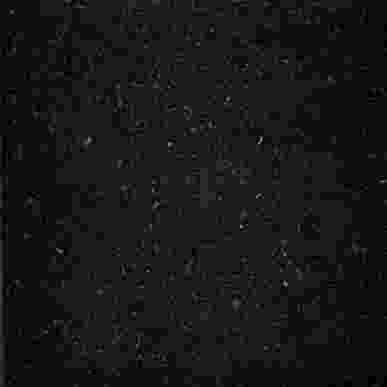

Supplement: Supplementary file 4 [file DataSheet4.zip › train/300120-2024-4-1-21-56-21.JPG]

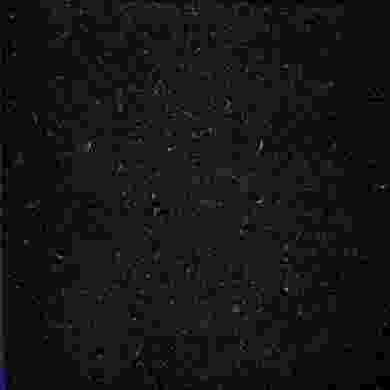

Supplement: Supplementary file 4 [file DataSheet4.zip › train/300120-2024-4-2-0-20-29.JPG]

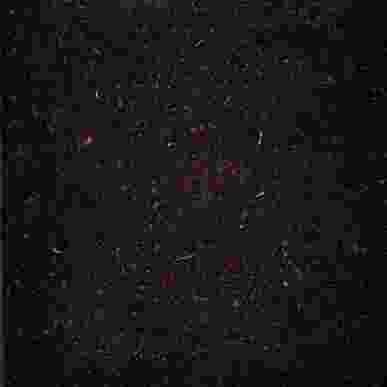

Supplement: Supplementary file 4 [file DataSheet4.zip › train/300120-2024-4-2-12-21-18.JPG]

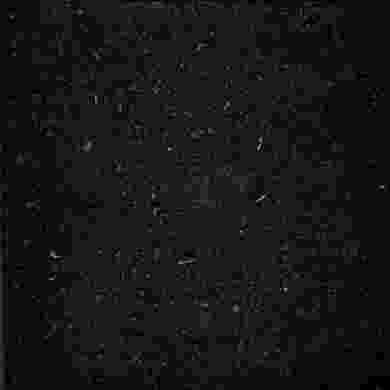

Supplement: Supplementary file 4 [file DataSheet4.zip › train/300120-2024-4-2-14-45-38.JPG]

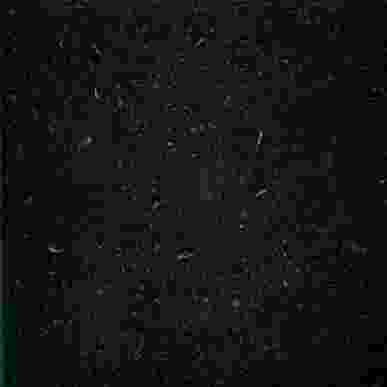

Supplement: Supplementary file 4 [file DataSheet4.zip › train/300120-2024-4-2-17-10-10.JPG]

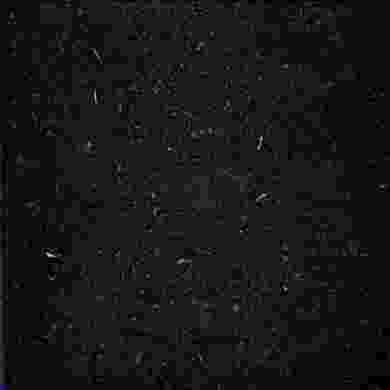

Supplement: Supplementary file 4 [file DataSheet4.zip › train/300120-2024-4-2-19-34-34.JPG]

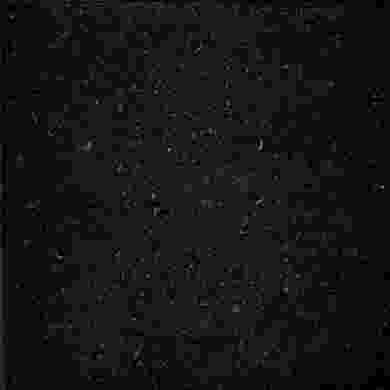

Supplement: Supplementary file 4 [file DataSheet4.zip › train/300120-2024-4-2-2-44-38.JPG]

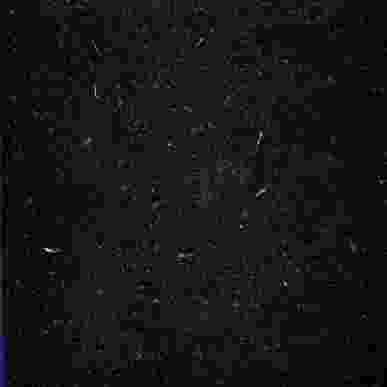

Supplement: Supplementary file 4 [file DataSheet4.zip › train/300120-2024-4-2-21-58-27.JPG]

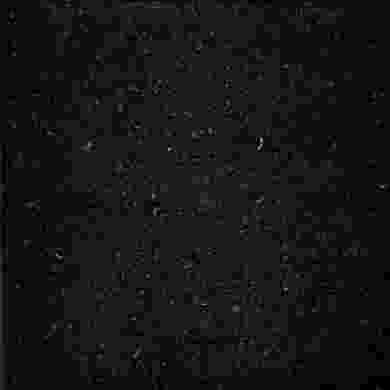

Supplement: Supplementary file 4 [file DataSheet4.zip › train/300120-2024-4-2-5-8-44.JPG]

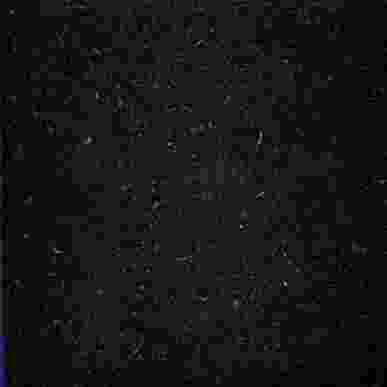

Supplement: Supplementary file 4 [file DataSheet4.zip › train/300120-2024-4-2-9-57-0.JPG]

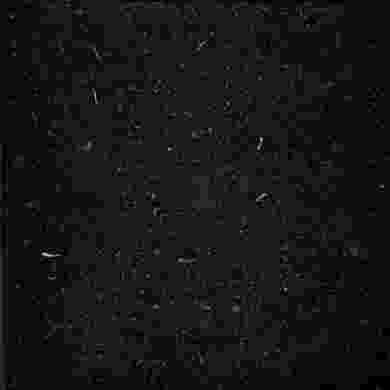

Supplement: Supplementary file 4 [file DataSheet4.zip › train/300120-2024-4-3-0-23-6.JPG]

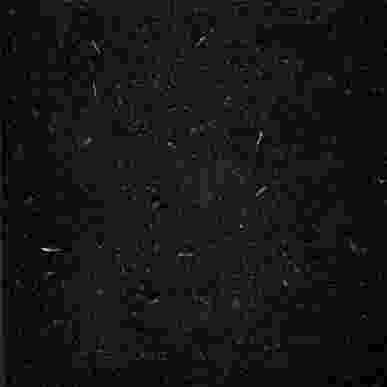

Supplement: Supplementary file 4 [file DataSheet4.zip › train/300120-2024-4-3-2-47-0.JPG]

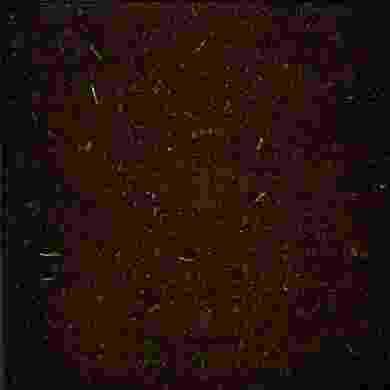

Supplement: Supplementary file 4 [file DataSheet4.zip › train/300120-2024-4-3-9-59-31.JPG]

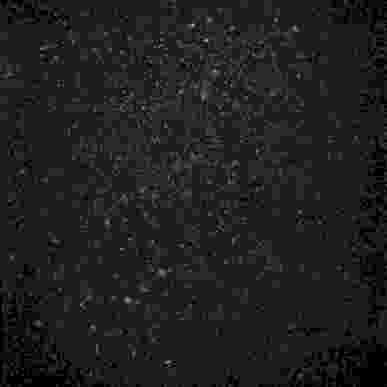

Supplement: Supplementary file 4 [file DataSheet4.zip › train/300150-2024-4-1-22-14-47.JPG]

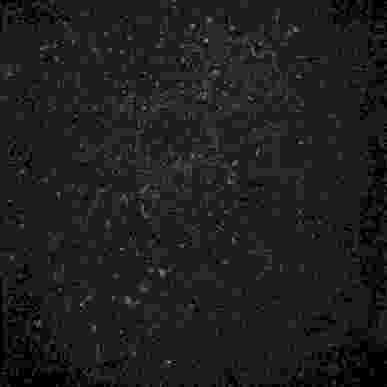

Supplement: Supplementary file 4 [file DataSheet4.zip › train/300150-2024-4-2-10-15-33.JPG]

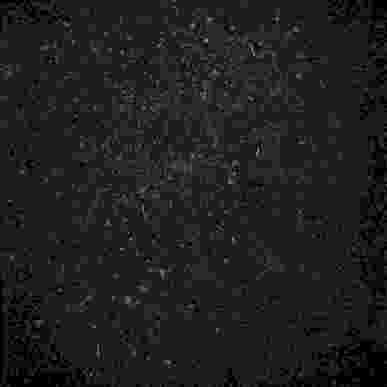

Supplement: Supplementary file 4 [file DataSheet4.zip › train/300150-2024-4-2-12-39-46.JPG]

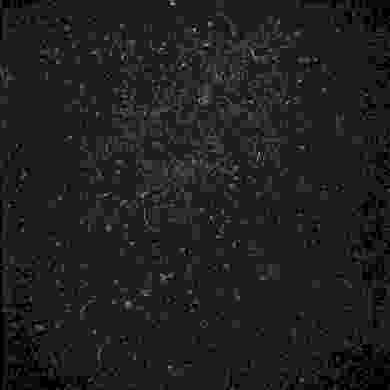

Supplement: Supplementary file 4 [file DataSheet4.zip › train/300150-2024-4-2-15-3-56.JPG]

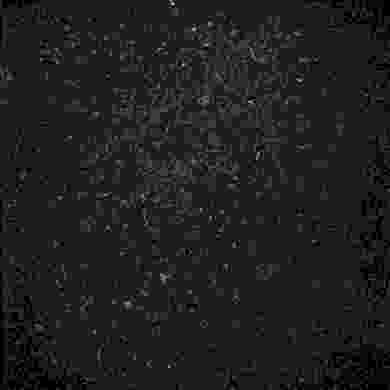

Supplement: Supplementary file 4 [file DataSheet4.zip › train/300150-2024-4-2-17-28-49.JPG]

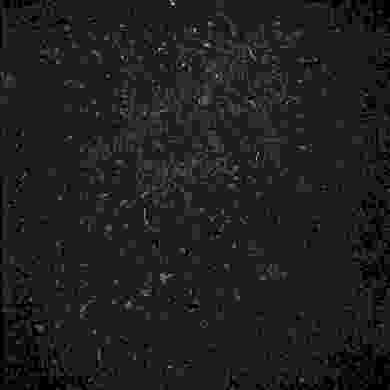

Supplement: Supplementary file 4 [file DataSheet4.zip › train/300150-2024-4-2-19-52-57.JPG]

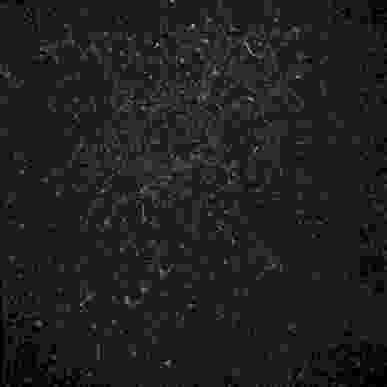

Supplement: Supplementary file 4 [file DataSheet4.zip › train/300150-2024-4-2-22-17-16.JPG]

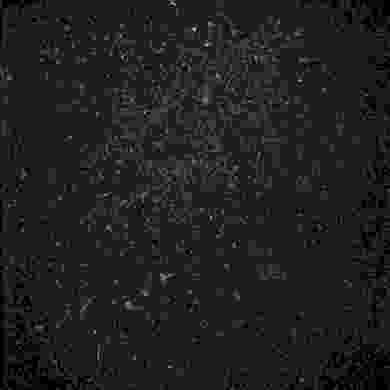

Supplement: Supplementary file 4 [file DataSheet4.zip › train/300150-2024-4-2-3-2-57.JPG]

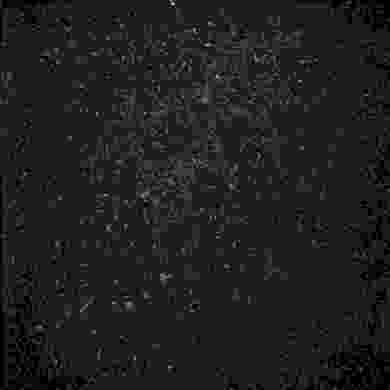

Supplement: Supplementary file 4 [file DataSheet4.zip › train/300150-2024-4-2-5-27-1.JPG]

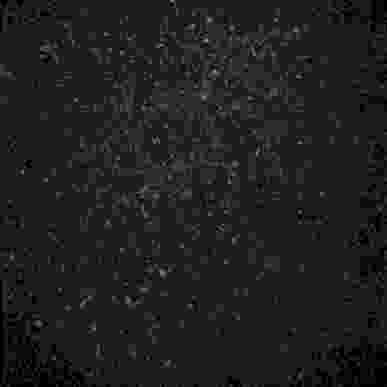

Supplement: Supplementary file 4 [file DataSheet4.zip › train/300150-2024-4-2-7-51-32.JPG]

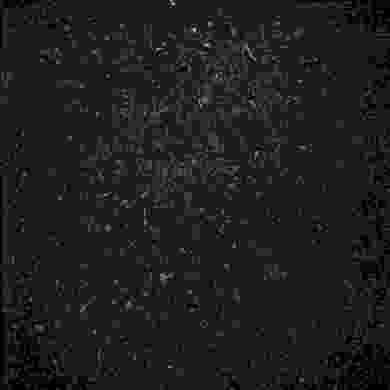

Supplement: Supplementary file 4 [file DataSheet4.zip › train/300150-2024-4-3-0-41-22.JPG]

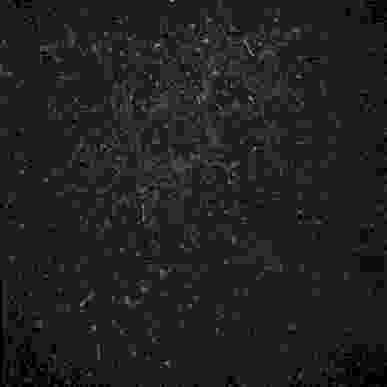

Supplement: Supplementary file 4 [file DataSheet4.zip › train/300150-2024-4-3-3-5-28.JPG]

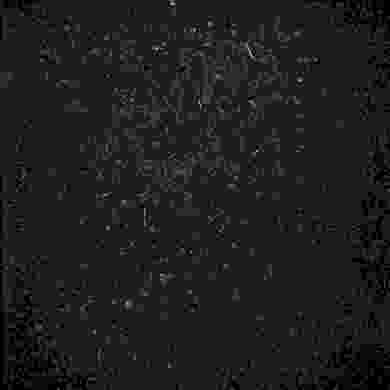

Supplement: Supplementary file 4 [file DataSheet4.zip › train/300150-2024-4-3-5-29-54.JPG]

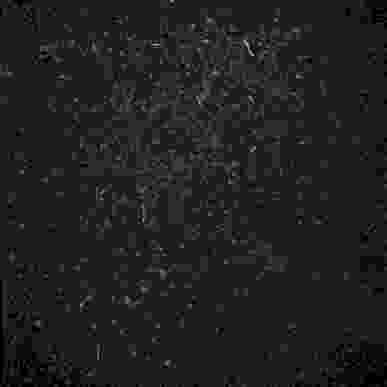

Supplement: Supplementary file 4 [file DataSheet4.zip › train/300150-2024-4-3-7-54-7.JPG]

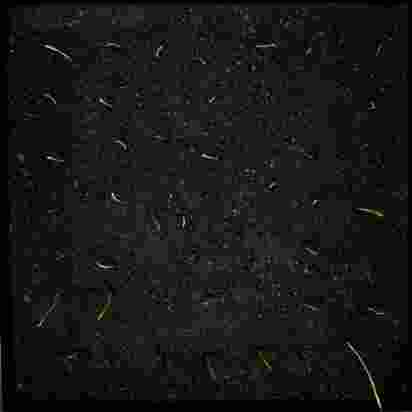

Supplement: Supplementary file 4 [file DataSheet4.zip › train/30030-2024-4-2-0-1-5.JPG]

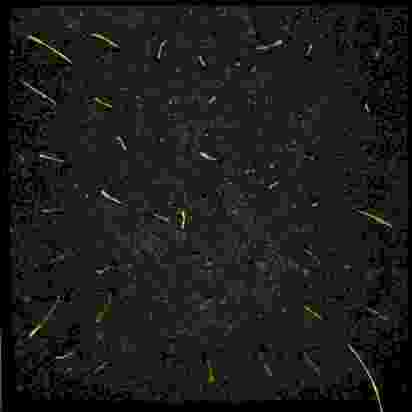

Supplement: Supplementary file 4 [file DataSheet4.zip › train/30030-2024-4-2-16-50-43.JPG]

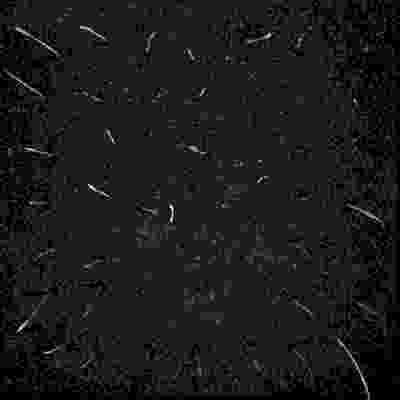

Supplement: Supplementary file 4 [file DataSheet4.zip › train/30030-2024-4-2-19-15-9.JPG]

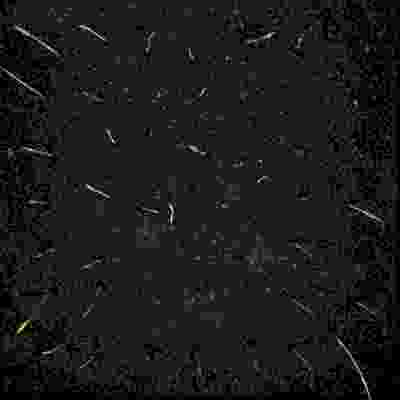

Supplement: Supplementary file 4 [file DataSheet4.zip › train/30030-2024-4-2-21-39-1.JPG]

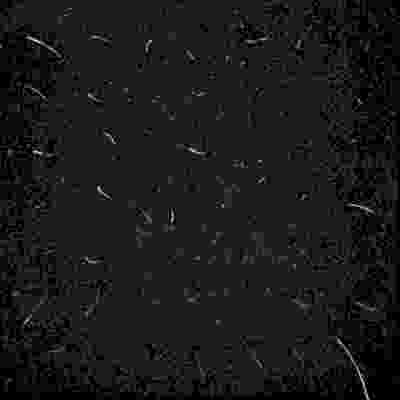

Supplement: Supplementary file 4 [file DataSheet4.zip › train/30030-2024-4-2-4-49-30.JPG]

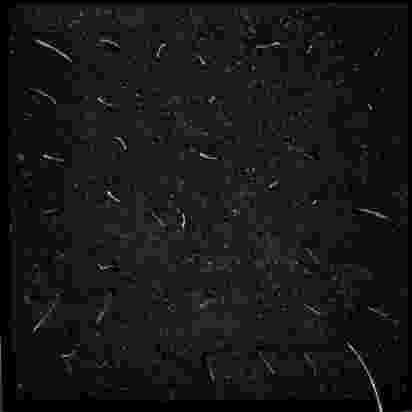

Supplement: Supplementary file 4 [file DataSheet4.zip › train/30030-2024-4-2-7-13-46.JPG]

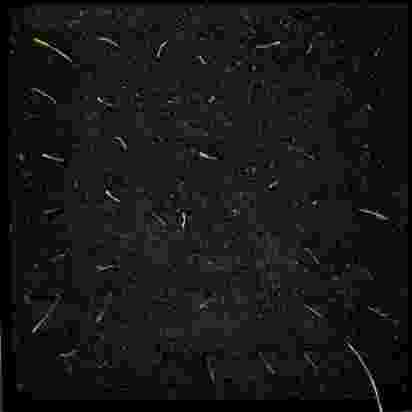

Supplement: Supplementary file 4 [file DataSheet4.zip › train/30030-2024-4-2-9-37-31.JPG]

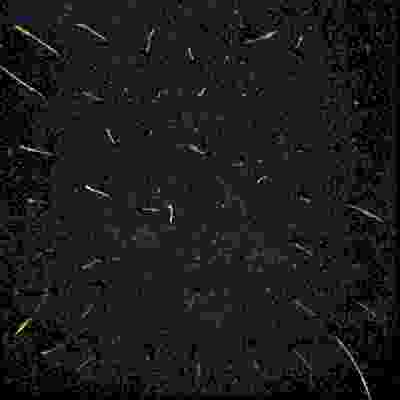

Supplement: Supplementary file 4 [file DataSheet4.zip › train/30030-2024-4-3-0-3-54.JPG]

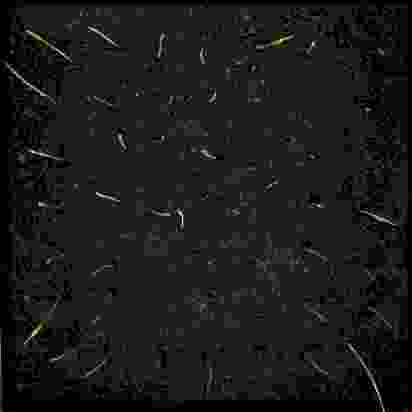

Supplement: Supplementary file 4 [file DataSheet4.zip › train/30030-2024-4-3-4-52-14.JPG]
