# Supplementary figures and images for: VanillaNet-YOLOv8 segment: detection of nano-iron oxide regulation on rice seedling growth vitality under salt stress (part 4 of 5)
Source: Front Plant Sci. 2025 Sep 17;16:1631279. doi: 10.3389/fpls.2025.1631279 (PMC12484053; doi:10.3389/fpls.2025.1631279)

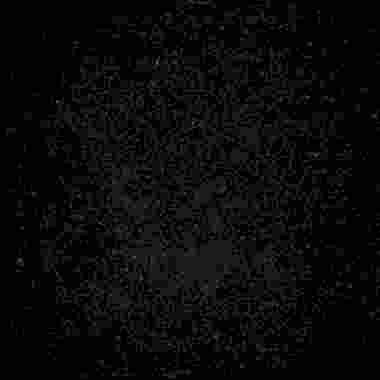

Supplement: Supplementary file 4 [file DataSheet4.zip › train/100150-2024-3-18-16-10-52.JPG]

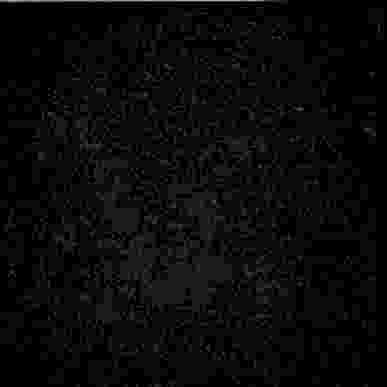

Supplement: Supplementary file 4 [file DataSheet4.zip › train/100150-2024-3-18-21-20-24.JPG]

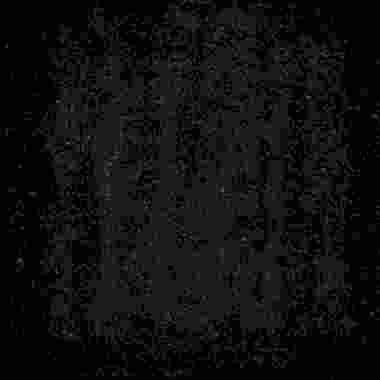

Supplement: Supplementary file 4 [file DataSheet4.zip › train/100150-2024-3-19-10-7-49.JPG]

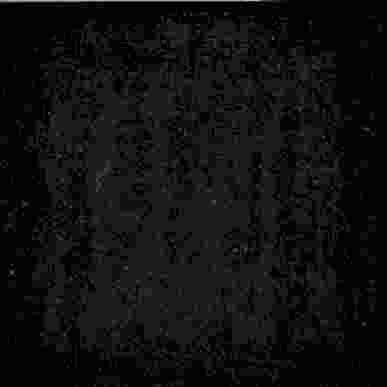

Supplement: Supplementary file 4 [file DataSheet4.zip › train/100150-2024-3-19-15-12-30.JPG]

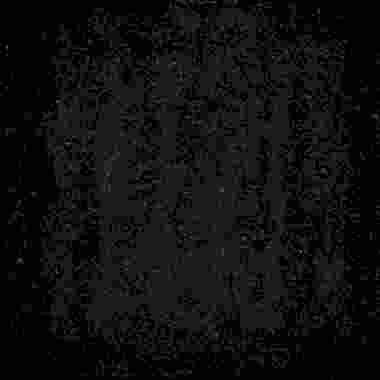

Supplement: Supplementary file 4 [file DataSheet4.zip › train/100150-2024-3-19-17-45-7.JPG]

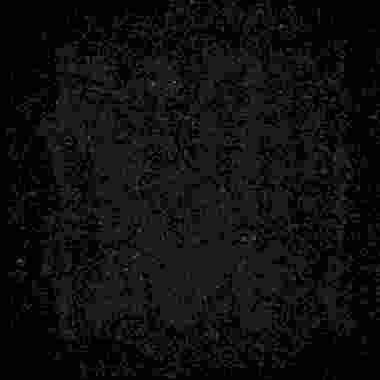

Supplement: Supplementary file 4 [file DataSheet4.zip › train/100150-2024-3-19-2-27-41.JPG]

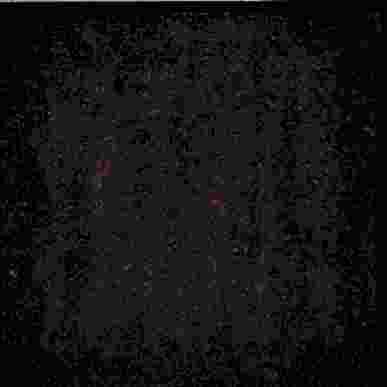

Supplement: Supplementary file 4 [file DataSheet4.zip › train/100150-2024-3-19-20-18-41.JPG]

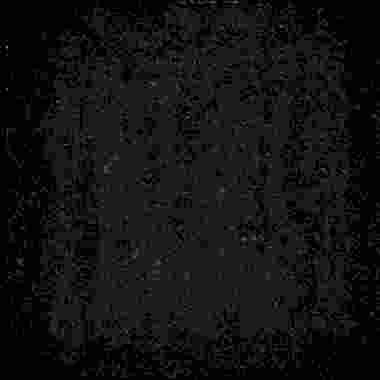

Supplement: Supplementary file 4 [file DataSheet4.zip › train/100150-2024-3-19-22-51-24.JPG]

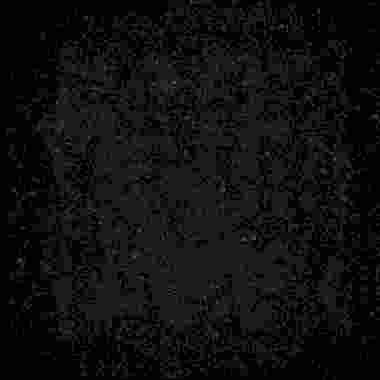

Supplement: Supplementary file 4 [file DataSheet4.zip › train/100150-2024-3-19-5-1-8.JPG]

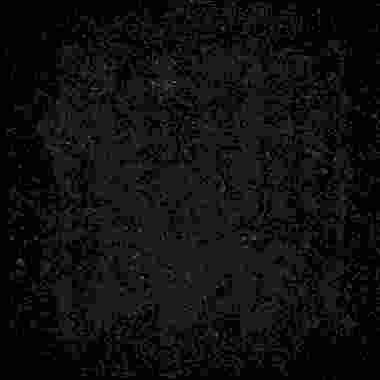

Supplement: Supplementary file 4 [file DataSheet4.zip › train/100150-2024-3-19-7-34-51.JPG]

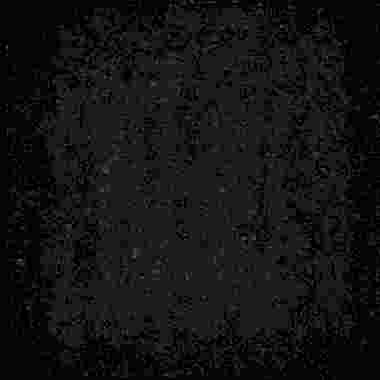

Supplement: Supplementary file 4 [file DataSheet4.zip › train/100150-2024-3-20-1-23-47.JPG]

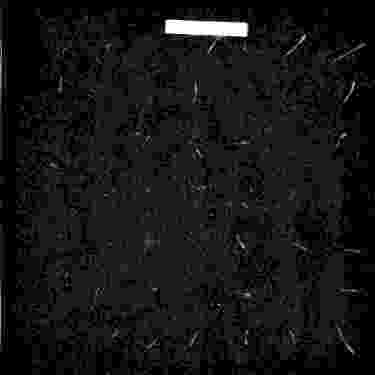

Supplement: Supplementary file 4 [file DataSheet4.zip › train/10030-2024-3-19-14-51-3.JPG]

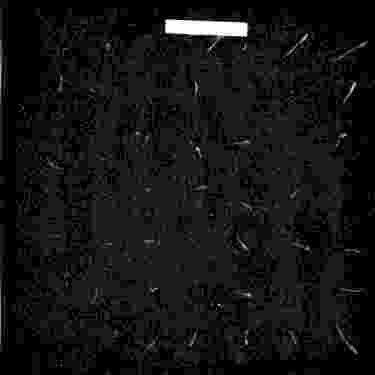

Supplement: Supplementary file 4 [file DataSheet4.zip › train/10030-2024-3-19-19-57-5.JPG]

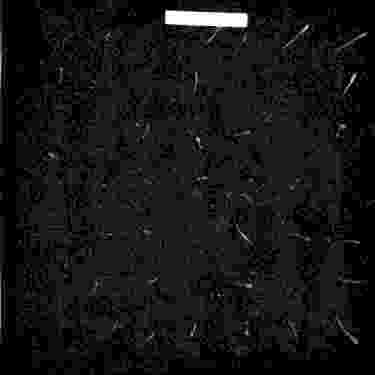

Supplement: Supplementary file 4 [file DataSheet4.zip › train/10030-2024-3-19-22-30-5.JPG]

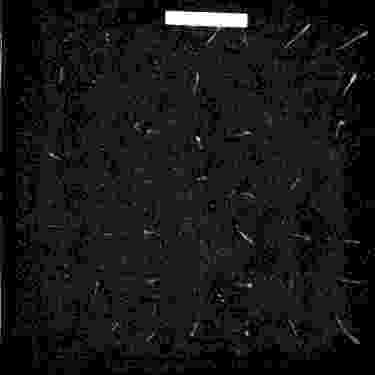

Supplement: Supplementary file 4 [file DataSheet4.zip › train/10030-2024-3-20-1-2-26.JPG]

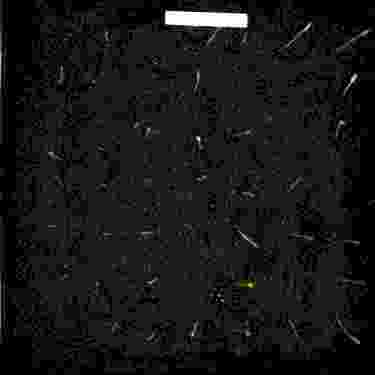

Supplement: Supplementary file 4 [file DataSheet4.zip › train/10030-2024-3-20-6-7-32.JPG]

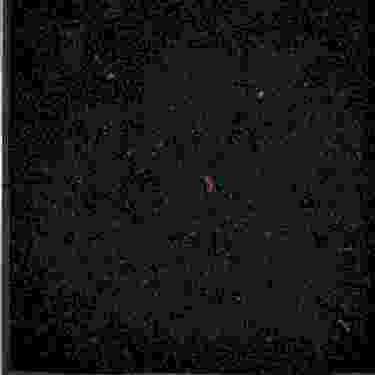

Supplement: Supplementary file 4 [file DataSheet4.zip › train/10060-2024-3-18-16-8-51.JPG]

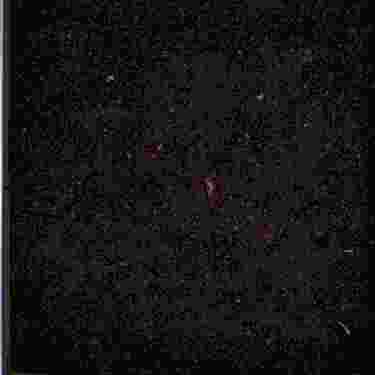

Supplement: Supplementary file 4 [file DataSheet4.zip › train/10060-2024-3-18-18-43-5.JPG]

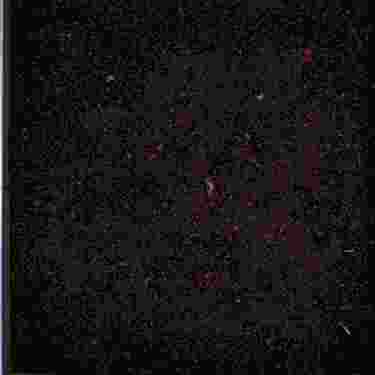

Supplement: Supplementary file 4 [file DataSheet4.zip › train/10060-2024-3-18-21-18-30.JPG]

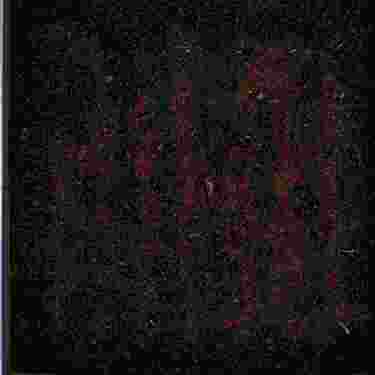

Supplement: Supplementary file 4 [file DataSheet4.zip › train/10060-2024-3-18-23-52-32.JPG]

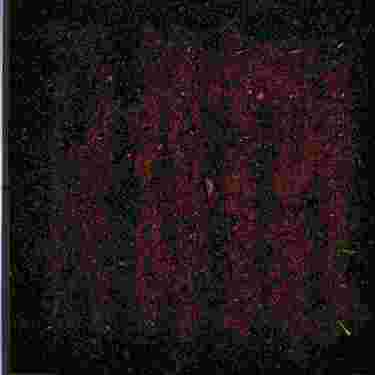

Supplement: Supplementary file 4 [file DataSheet4.zip › train/10060-2024-3-19-10-5-55.JPG]

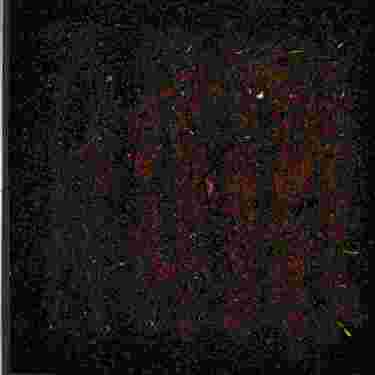

Supplement: Supplementary file 4 [file DataSheet4.zip › train/10060-2024-3-19-17-43-18.JPG]

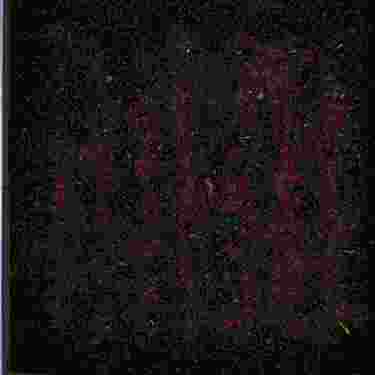

Supplement: Supplementary file 4 [file DataSheet4.zip › train/10060-2024-3-19-2-25-49.JPG]

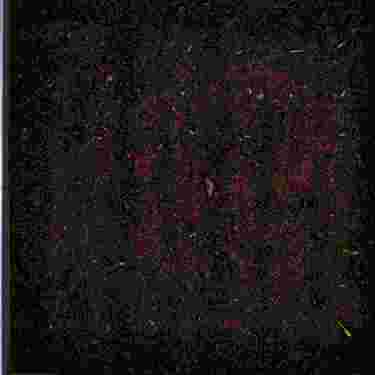

Supplement: Supplementary file 4 [file DataSheet4.zip › train/10060-2024-3-19-20-16-49.JPG]

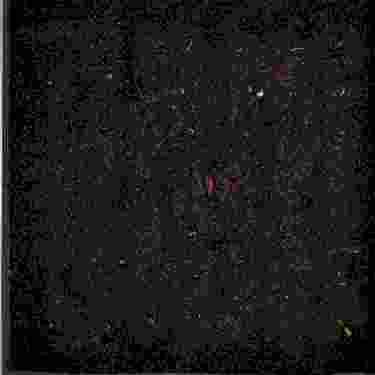

Supplement: Supplementary file 4 [file DataSheet4.zip › train/10060-2024-3-19-22-49-35.JPG]

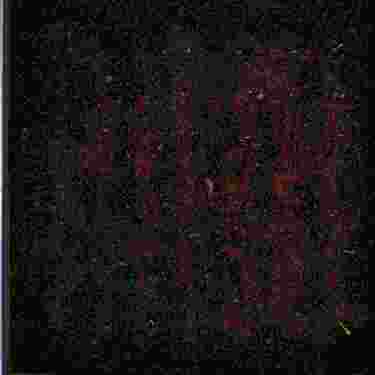

Supplement: Supplementary file 4 [file DataSheet4.zip › train/10060-2024-3-19-4-59-15.JPG]

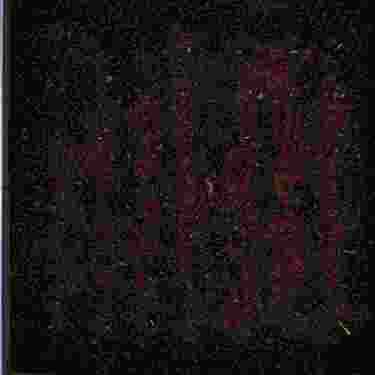

Supplement: Supplementary file 4 [file DataSheet4.zip › train/10060-2024-3-19-7-32-57.JPG]

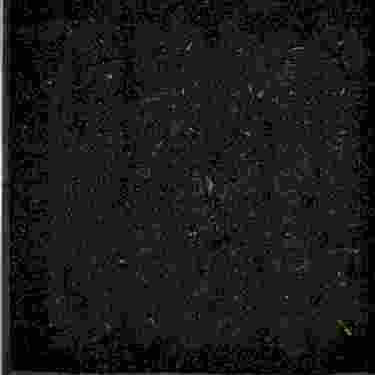

Supplement: Supplementary file 4 [file DataSheet4.zip › train/10060-2024-3-20-1-21-58.JPG]

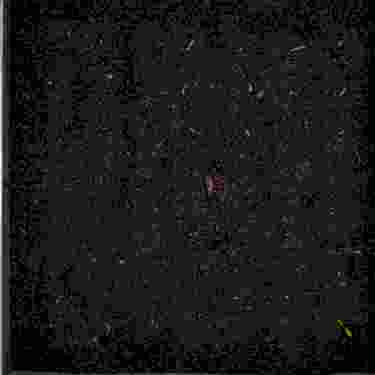

Supplement: Supplementary file 4 [file DataSheet4.zip › train/10060-2024-3-20-6-27-3.JPG]

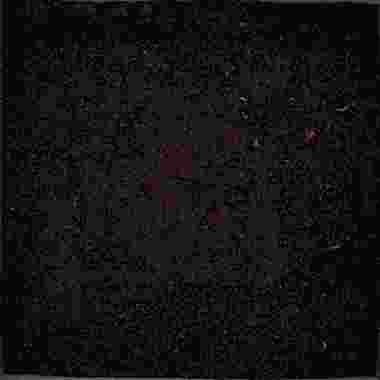

Supplement: Supplementary file 4 [file DataSheet4.zip › train/10090-2024-3-18-15-50-33.JPG]

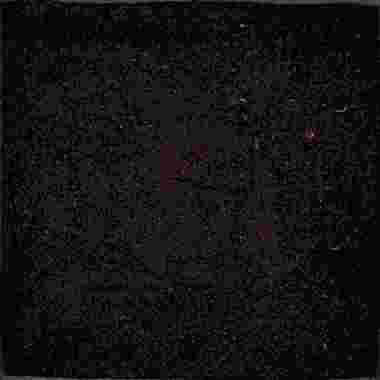

Supplement: Supplementary file 4 [file DataSheet4.zip › train/10090-2024-3-18-18-24-43.JPG]

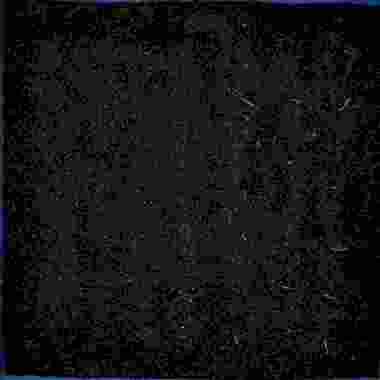

Supplement: Supplementary file 4 [file DataSheet4.zip › train/10090-2024-3-19-14-52-23.JPG]

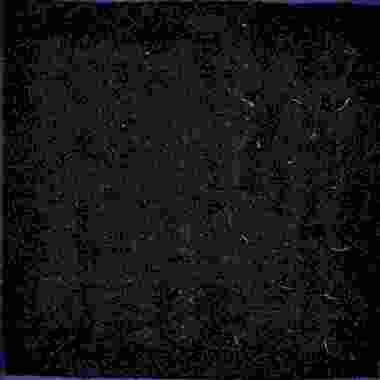

Supplement: Supplementary file 4 [file DataSheet4.zip › train/10090-2024-3-19-17-25-7.JPG]

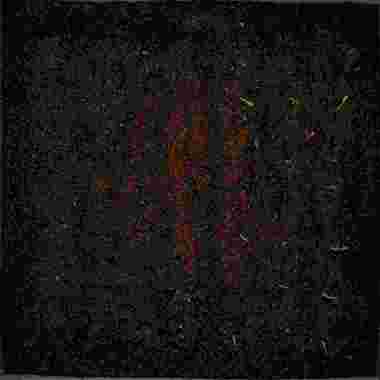

Supplement: Supplementary file 4 [file DataSheet4.zip › train/10090-2024-3-19-19-58-27.JPG]

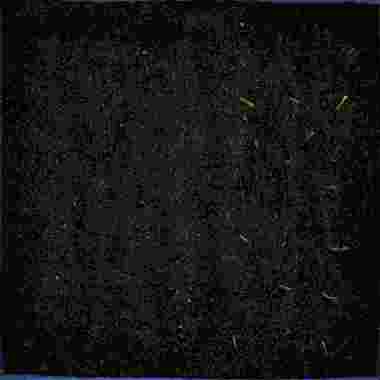

Supplement: Supplementary file 4 [file DataSheet4.zip › train/10090-2024-3-19-22-31-25.JPG]

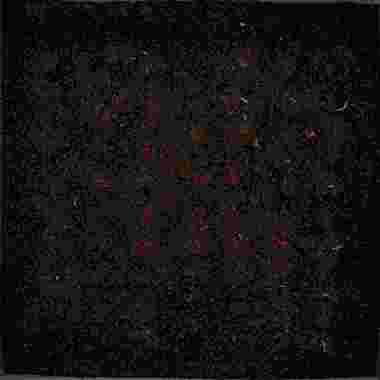

Supplement: Supplementary file 4 [file DataSheet4.zip › train/10090-2024-3-19-4-40-53.JPG]

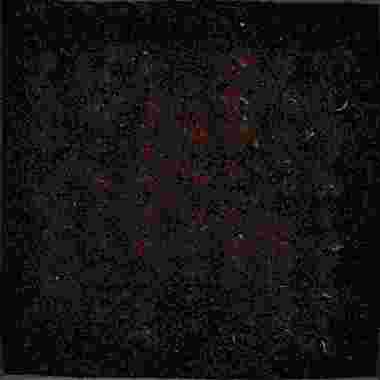

Supplement: Supplementary file 4 [file DataSheet4.zip › train/10090-2024-3-19-7-14-42.JPG]

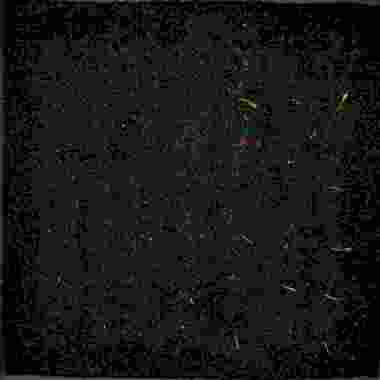

Supplement: Supplementary file 4 [file DataSheet4.zip › train/10090-2024-3-20-3-36-31.JPG]

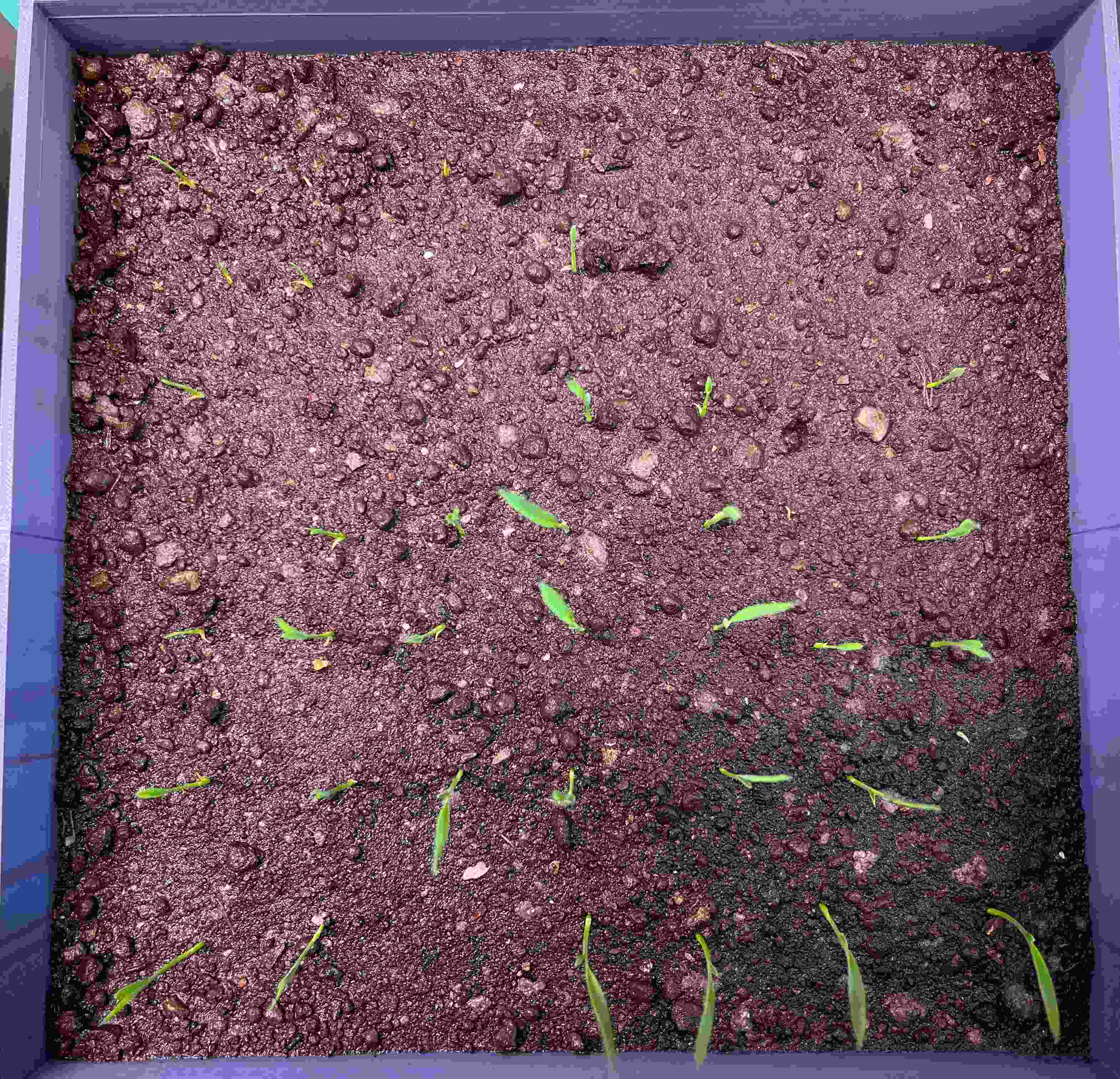

Supplement: Supplementary file 4 [file DataSheet4.zip › train/11-1.JPG]

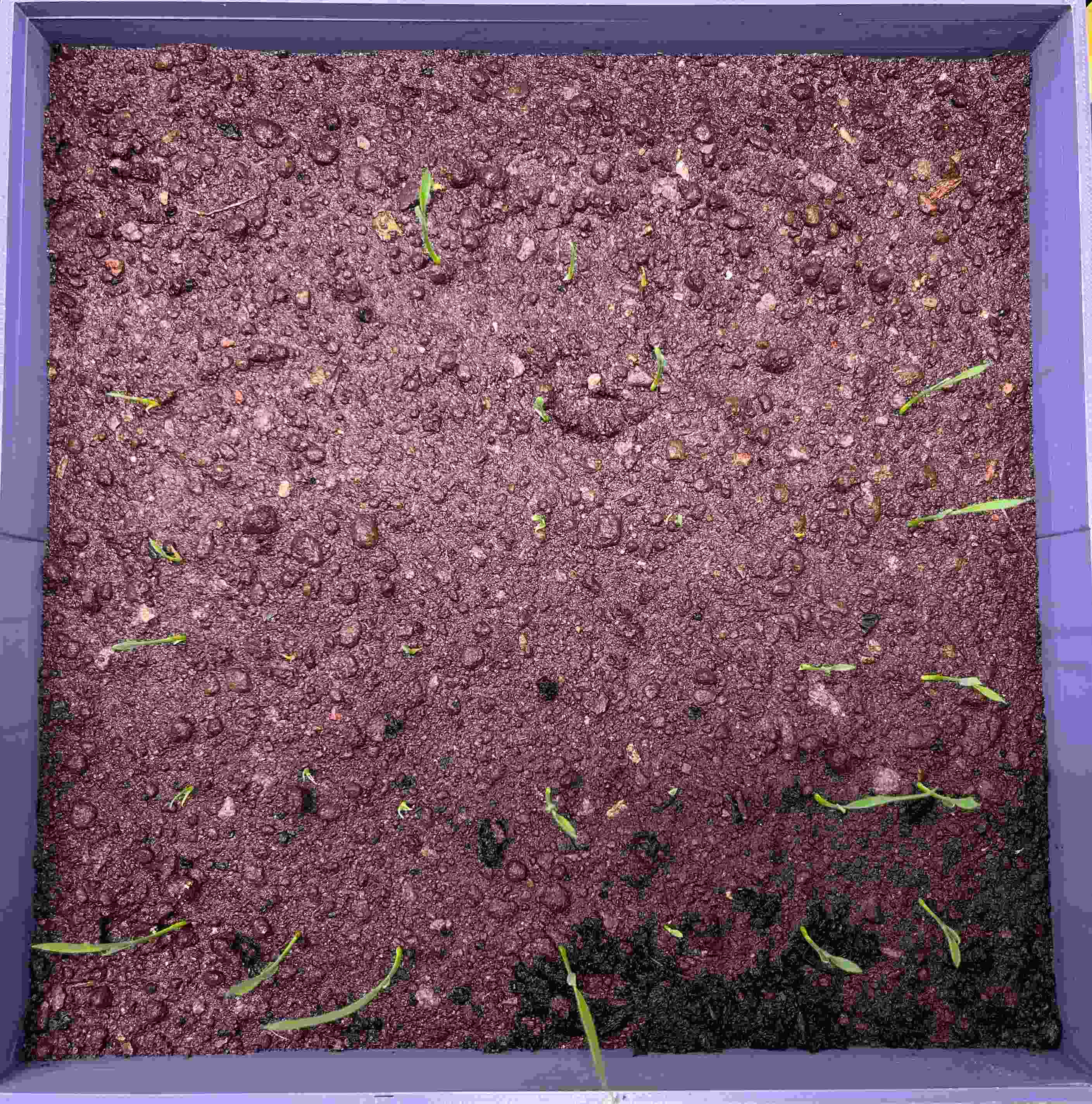

Supplement: Supplementary file 4 [file DataSheet4.zip › train/11-2.JPG]

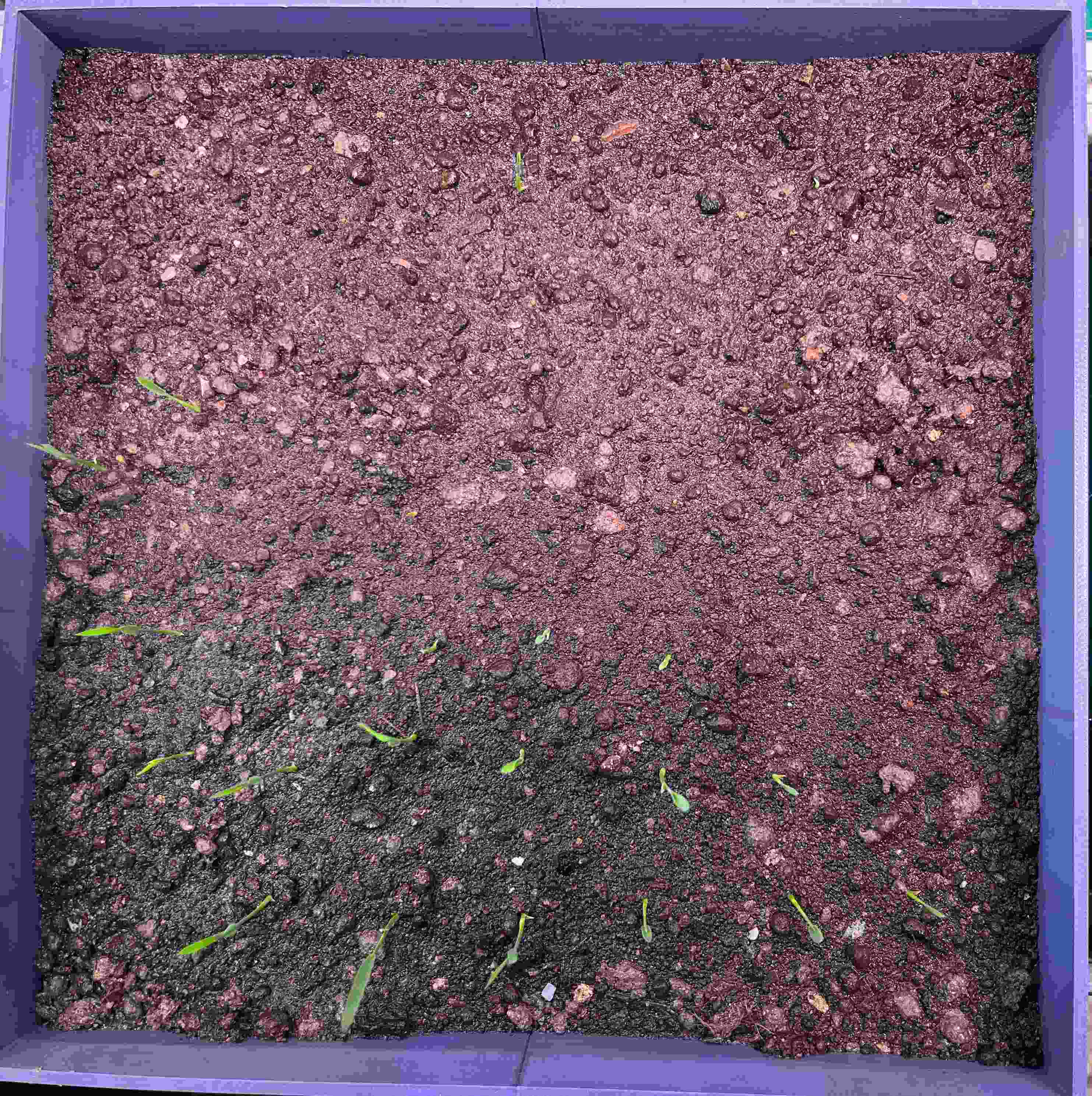

Supplement: Supplementary file 4 [file DataSheet4.zip › train/11-3.JPG]

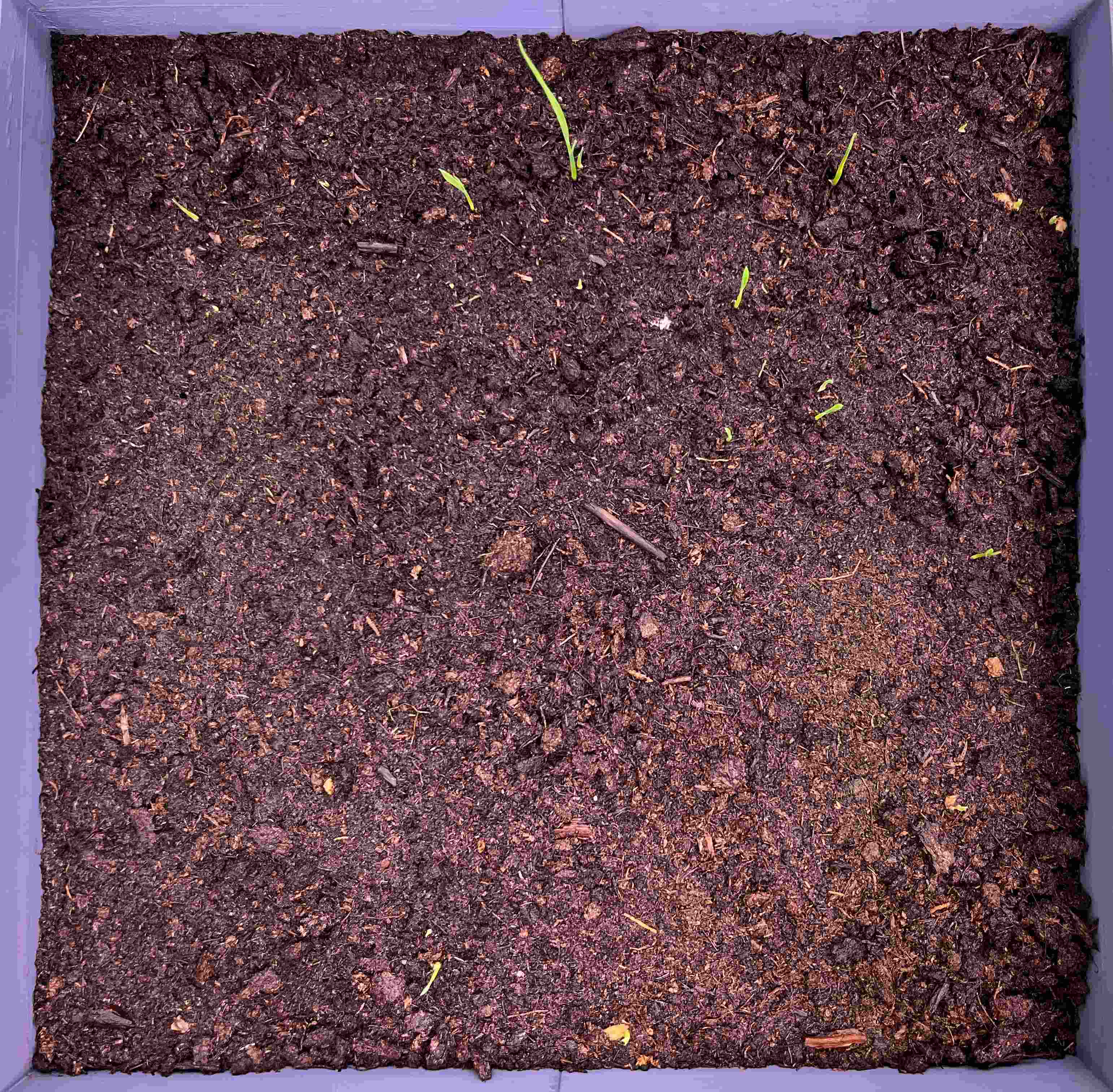

Supplement: Supplementary file 4 [file DataSheet4.zip › train/11-4.JPG]

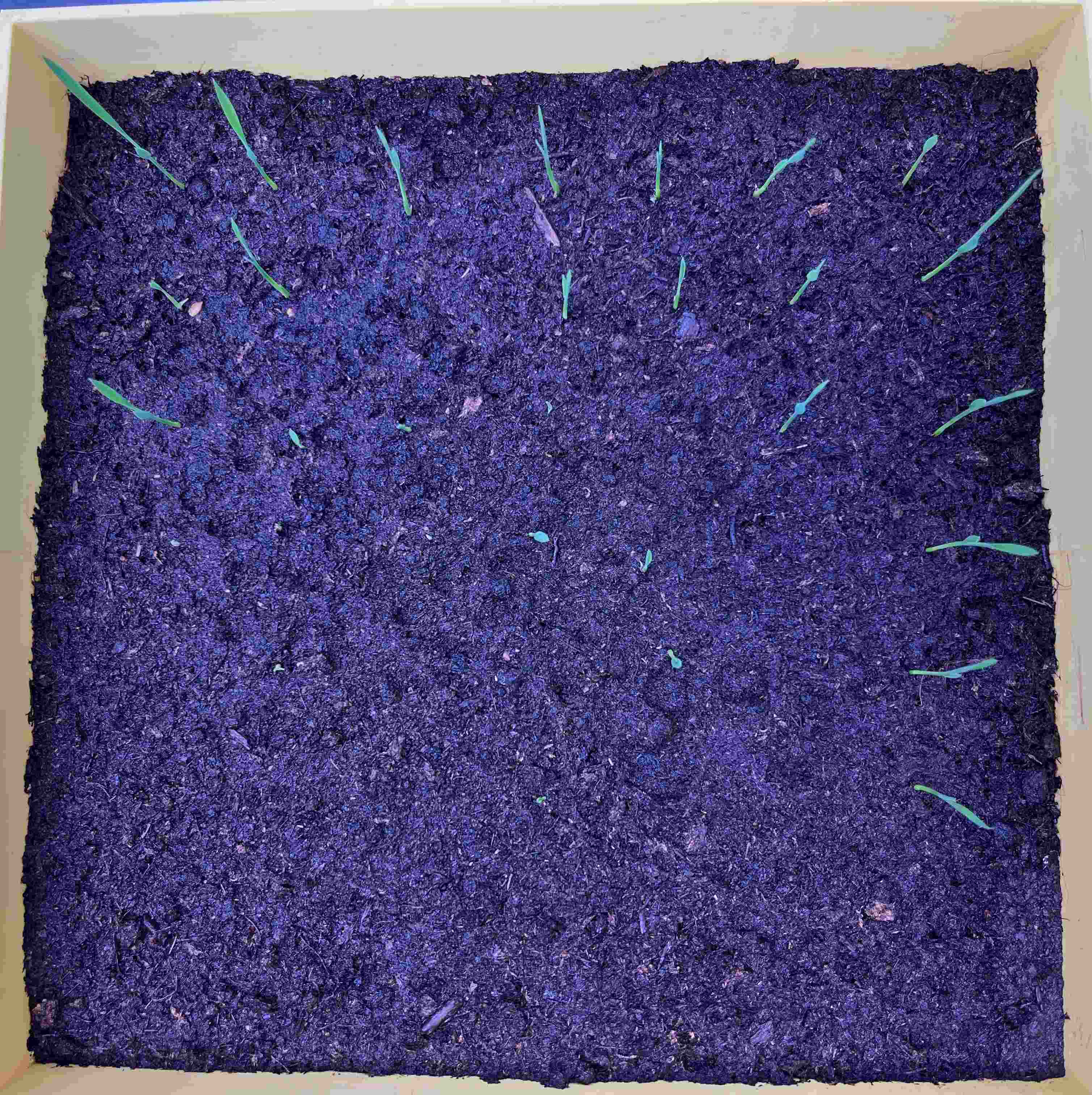

Supplement: Supplementary file 4 [file DataSheet4.zip › train/11-5.JPG]

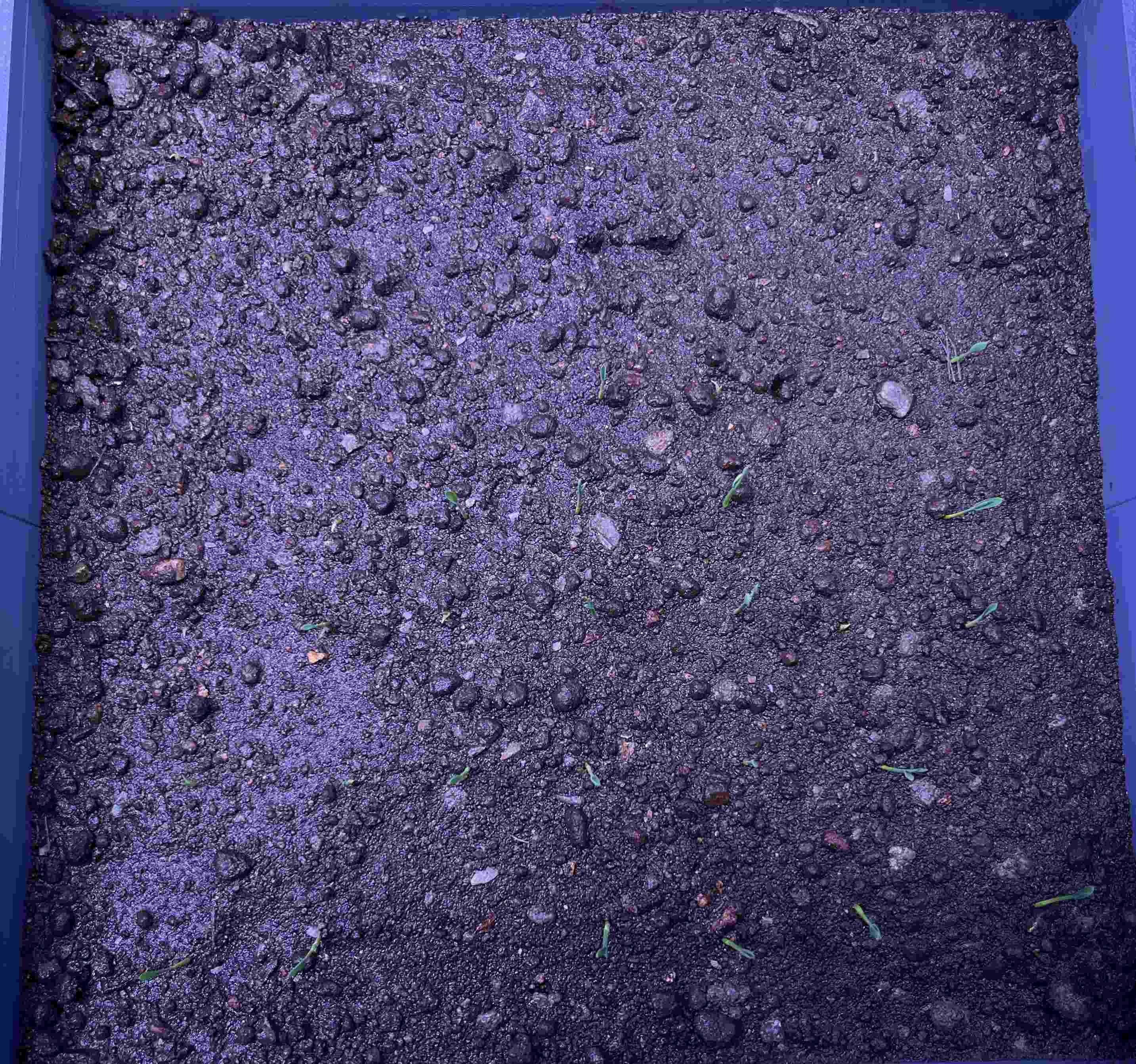

Supplement: Supplementary file 4 [file DataSheet4.zip › train/2-1.JPG]

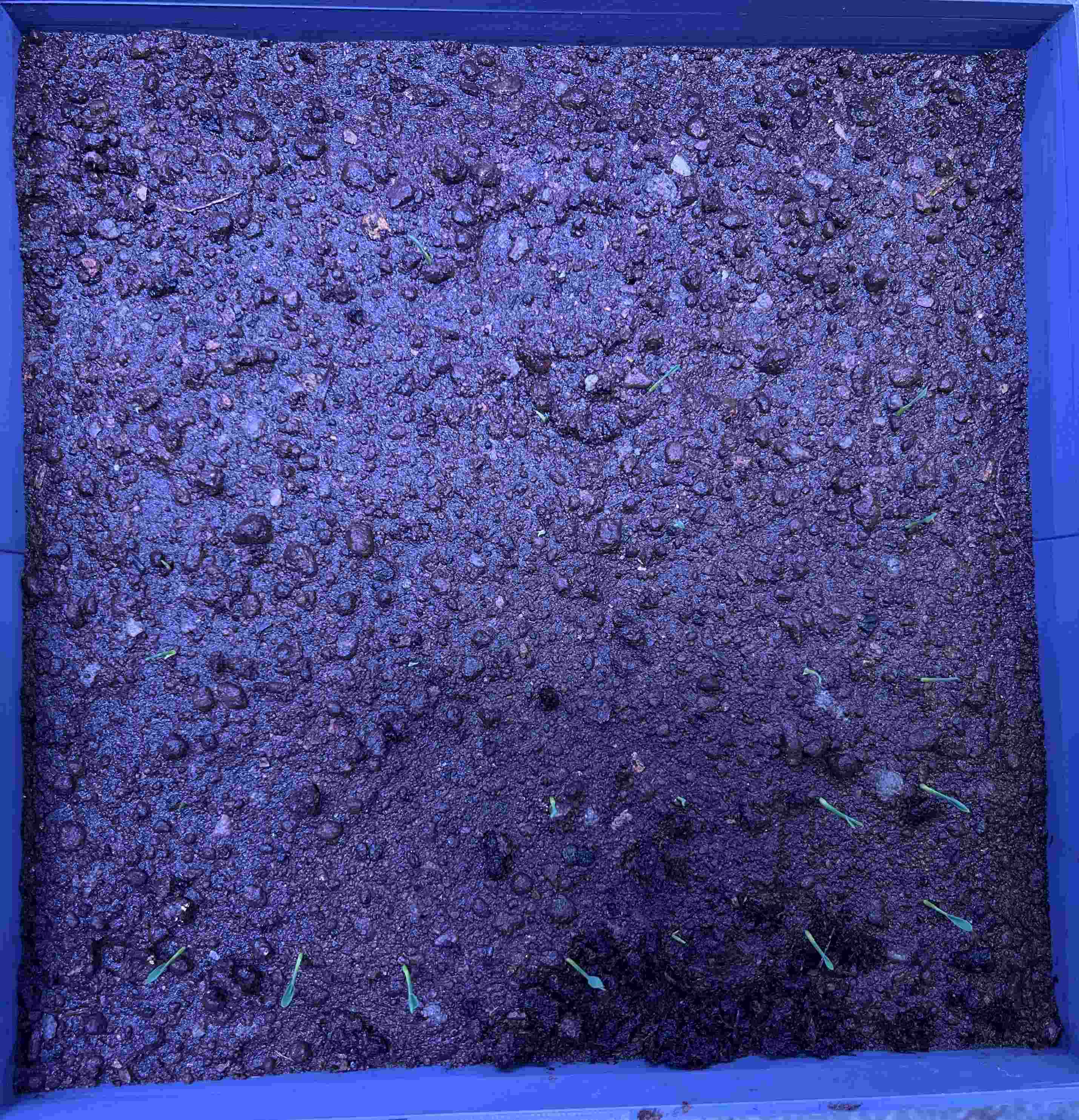

Supplement: Supplementary file 4 [file DataSheet4.zip › train/2-2.JPG]

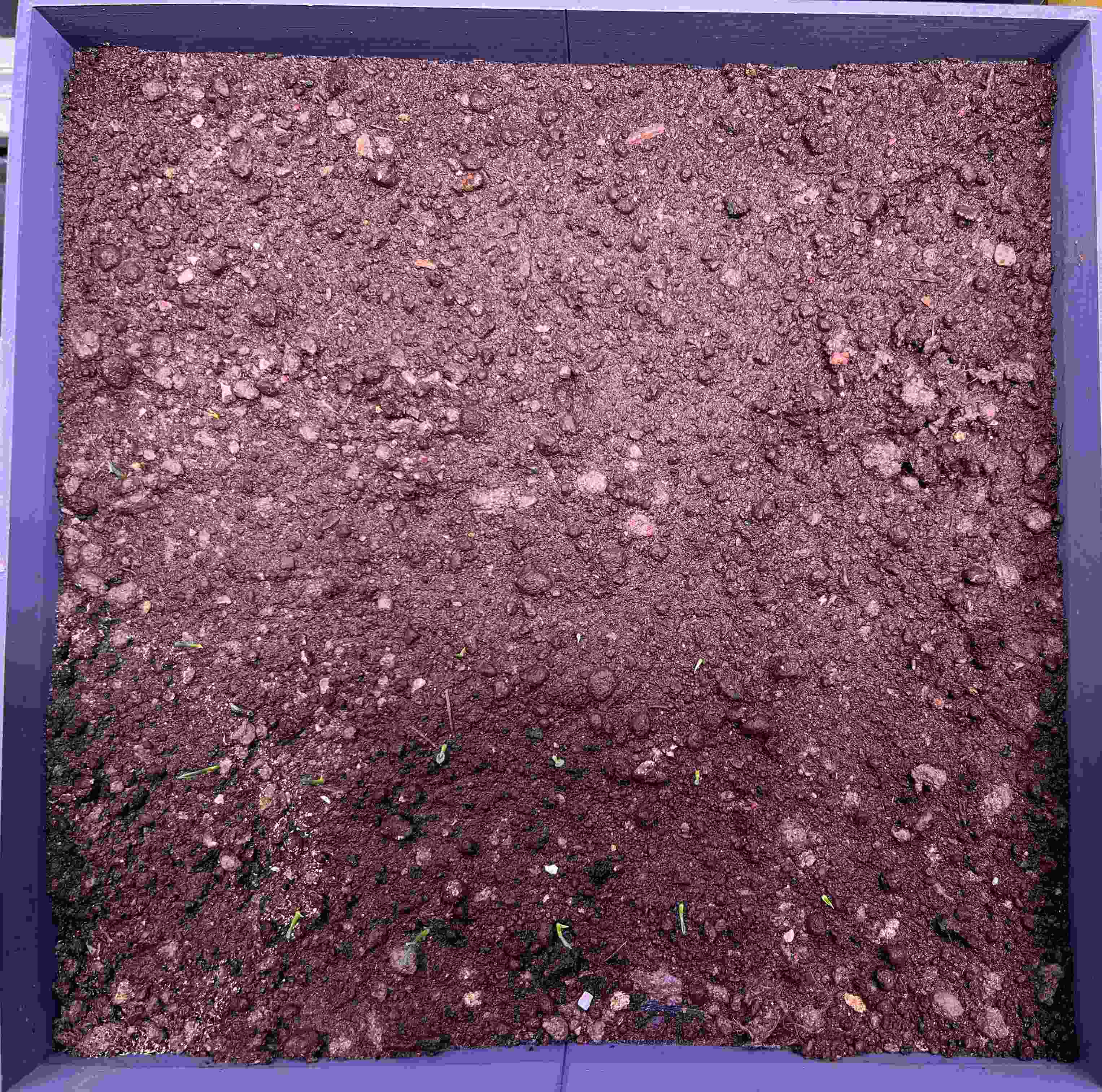

Supplement: Supplementary file 4 [file DataSheet4.zip › train/2-3.JPG]

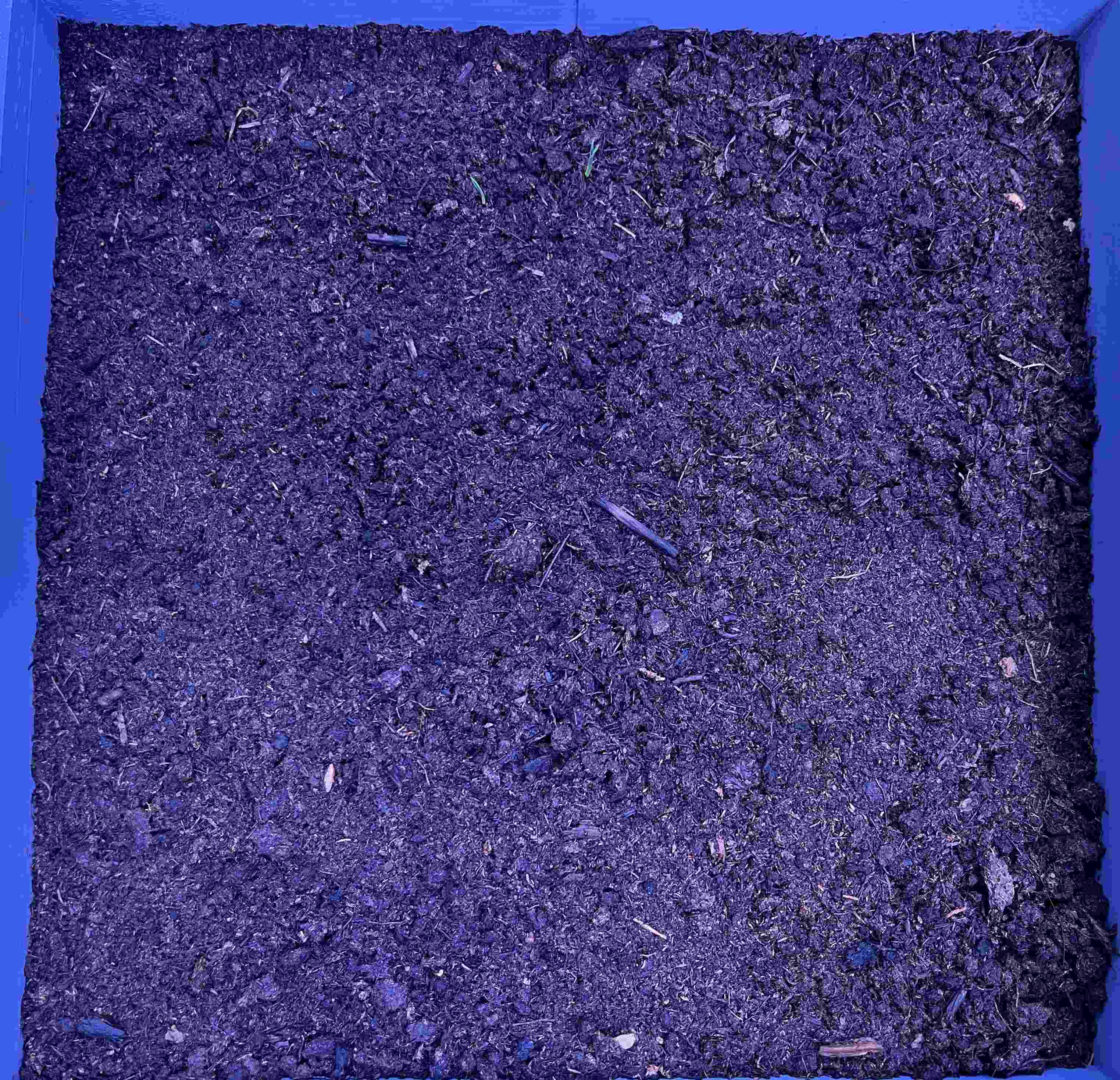

Supplement: Supplementary file 4 [file DataSheet4.zip › train/2-4.JPG]

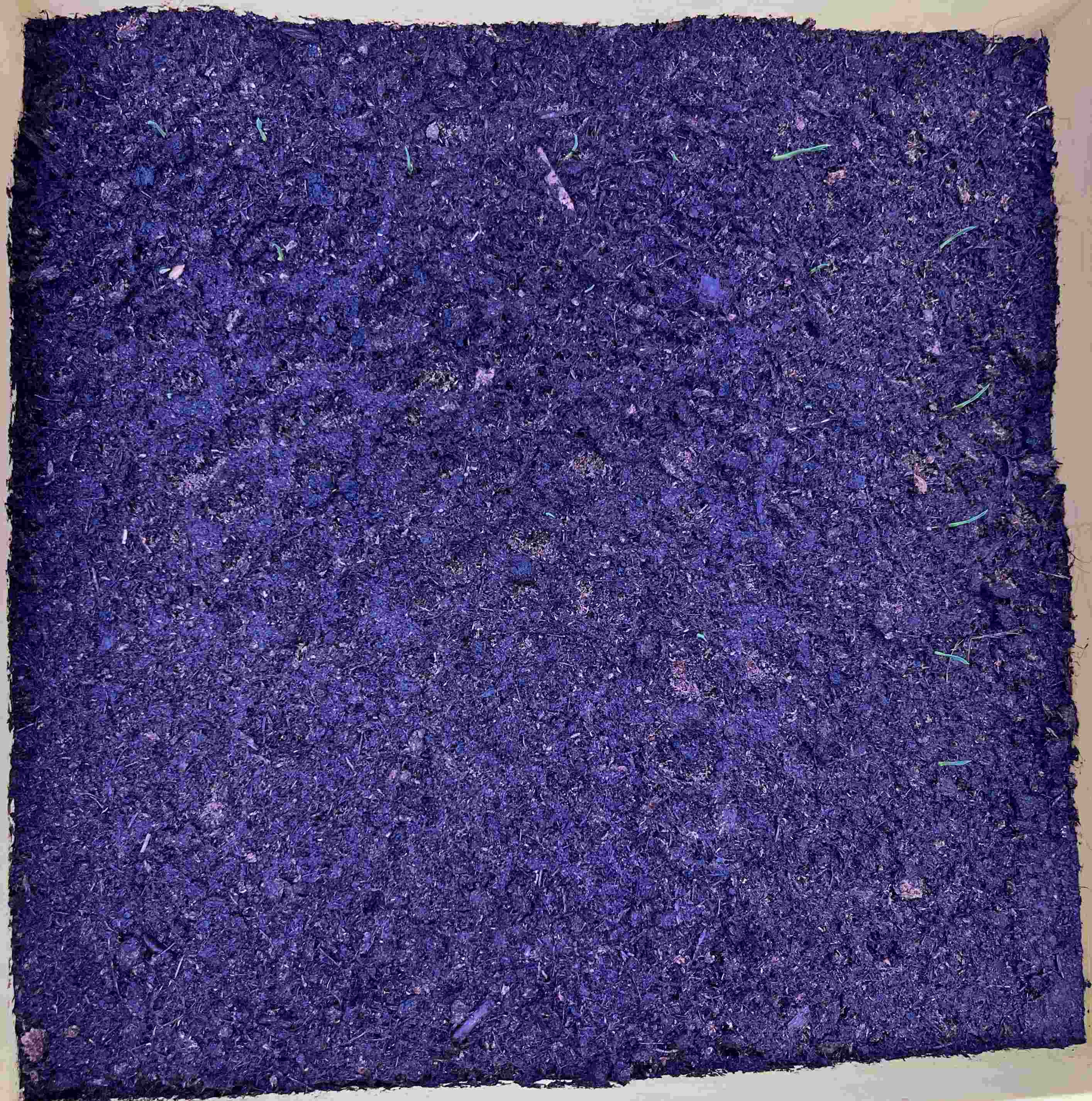

Supplement: Supplementary file 4 [file DataSheet4.zip › train/2-5.JPG]

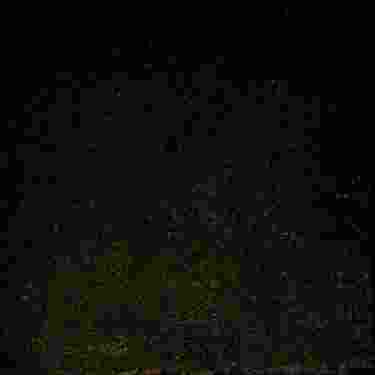

Supplement: Supplementary file 4 [file DataSheet4.zip › train/200120-2024-3-18-15-50-14.JPG]

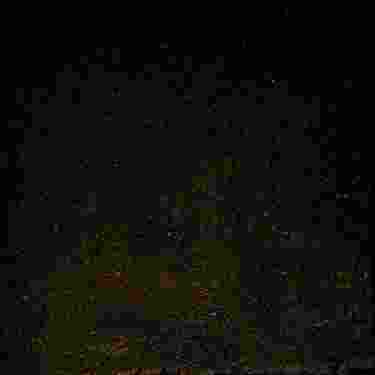

Supplement: Supplementary file 4 [file DataSheet4.zip › train/200120-2024-3-18-18-24-9.JPG]

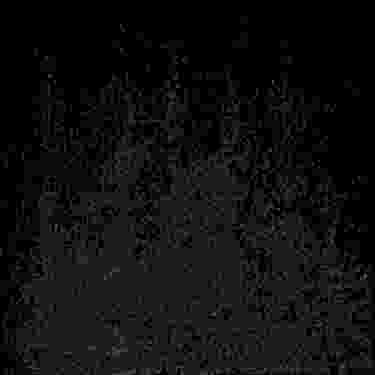

Supplement: Supplementary file 4 [file DataSheet4.zip › train/200120-2024-3-18-23-29-45.JPG]

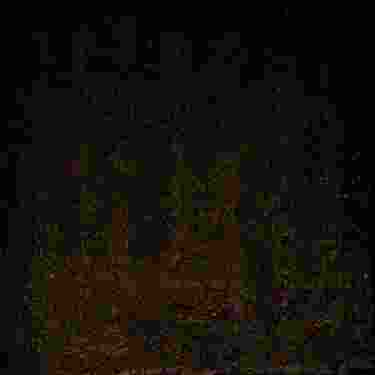

Supplement: Supplementary file 4 [file DataSheet4.zip › train/200120-2024-3-19-12-9-50.JPG]

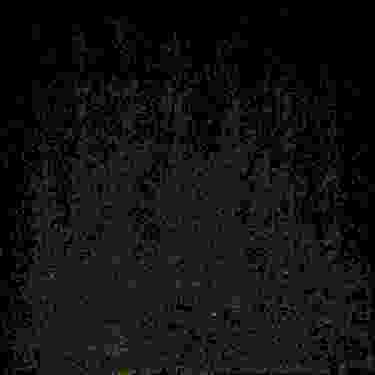

Supplement: Supplementary file 4 [file DataSheet4.zip › train/200120-2024-3-19-14-42-51.JPG]

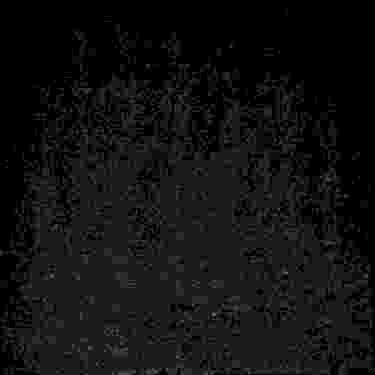

Supplement: Supplementary file 4 [file DataSheet4.zip › train/200120-2024-3-19-19-47-10.JPG]

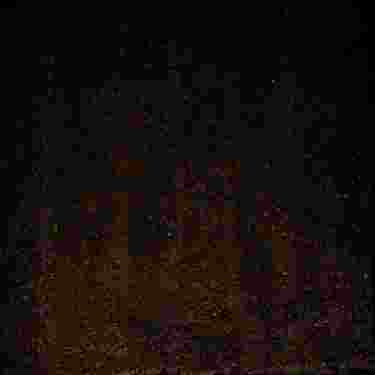

Supplement: Supplementary file 4 [file DataSheet4.zip › train/200120-2024-3-19-2-2-21.JPG]

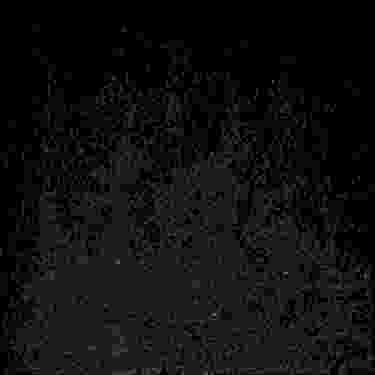

Supplement: Supplementary file 4 [file DataSheet4.zip › train/200120-2024-3-19-7-6-5.JPG]

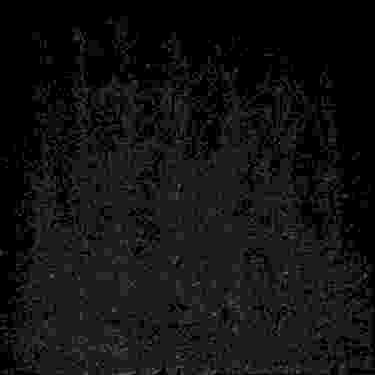

Supplement: Supplementary file 4 [file DataSheet4.zip › train/200120-2024-3-19-9-38-8.JPG]

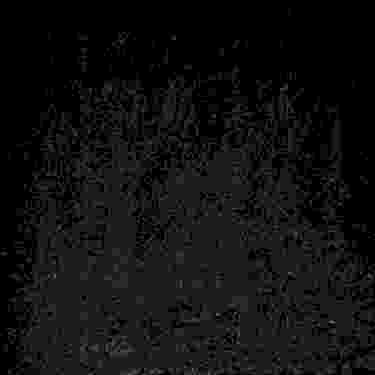

Supplement: Supplementary file 4 [file DataSheet4.zip › train/200120-2024-3-20-0-51-9.JPG]

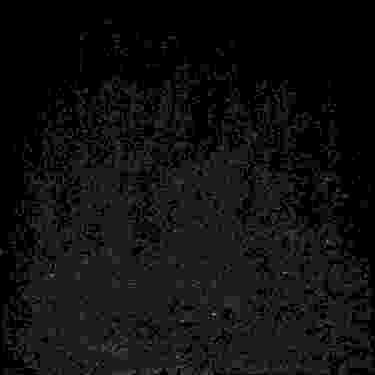

Supplement: Supplementary file 4 [file DataSheet4.zip › train/200120-2024-3-20-3-23-0.JPG]

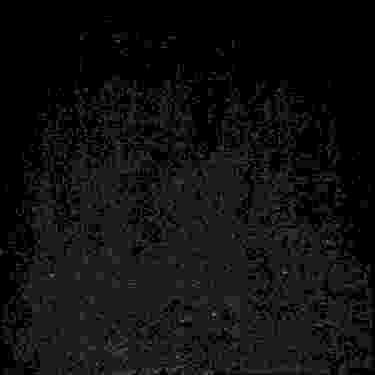

Supplement: Supplementary file 4 [file DataSheet4.zip › train/200120-2024-3-20-5-56-2.JPG]

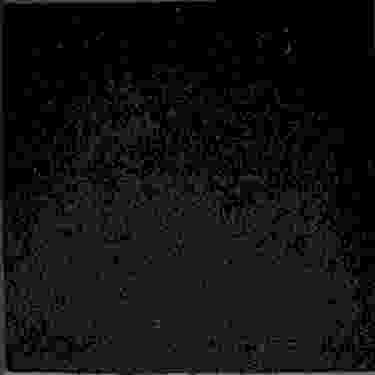

Supplement: Supplementary file 4 [file DataSheet4.zip › train/200150-2024-3-18-15-50-45.JPG]

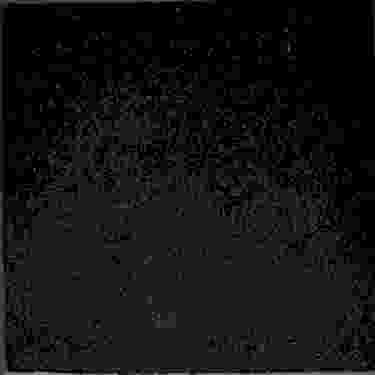

Supplement: Supplementary file 4 [file DataSheet4.zip › train/200150-2024-3-18-18-24-40.JPG]

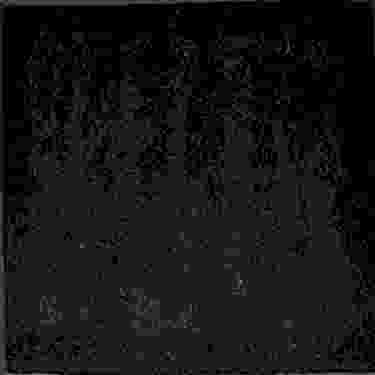

Supplement: Supplementary file 4 [file DataSheet4.zip › train/200150-2024-3-18-23-30-16.JPG]

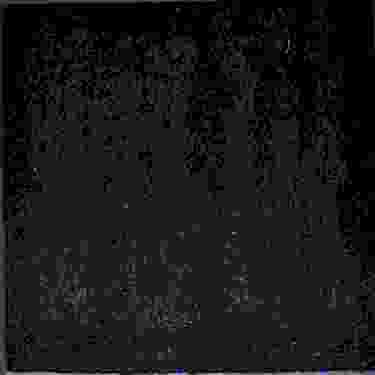

Supplement: Supplementary file 4 [file DataSheet4.zip › train/200150-2024-3-19-12-10-20.JPG]

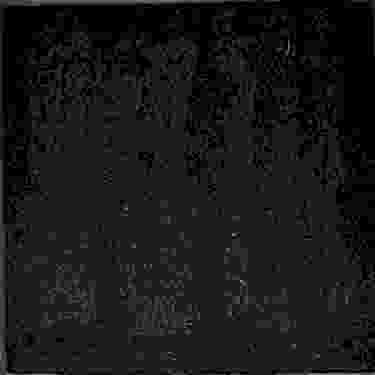

Supplement: Supplementary file 4 [file DataSheet4.zip › train/200150-2024-3-19-14-43-21.JPG]

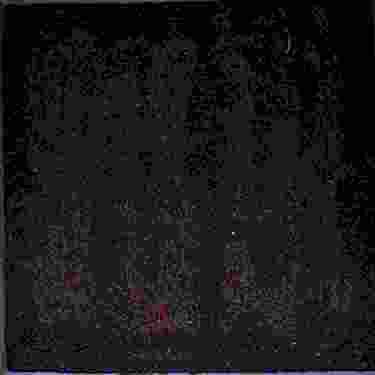

Supplement: Supplementary file 4 [file DataSheet4.zip › train/200150-2024-3-19-17-15-26.JPG]

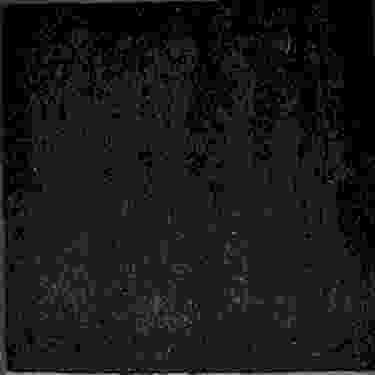

Supplement: Supplementary file 4 [file DataSheet4.zip › train/200150-2024-3-19-19-47-38.JPG]

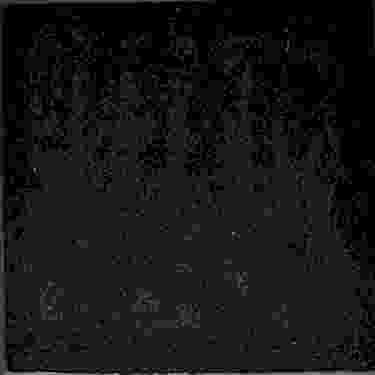

Supplement: Supplementary file 4 [file DataSheet4.zip › train/200150-2024-3-19-2-2-50.JPG]

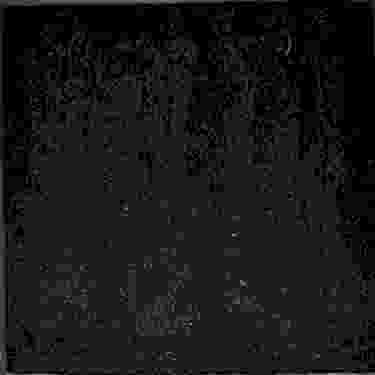

Supplement: Supplementary file 4 [file DataSheet4.zip › train/200150-2024-3-19-22-19-22.JPG]

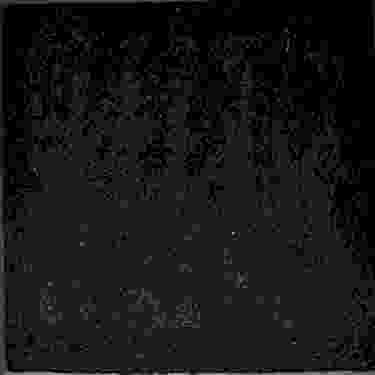

Supplement: Supplementary file 4 [file DataSheet4.zip › train/200150-2024-3-19-4-34-34.JPG]

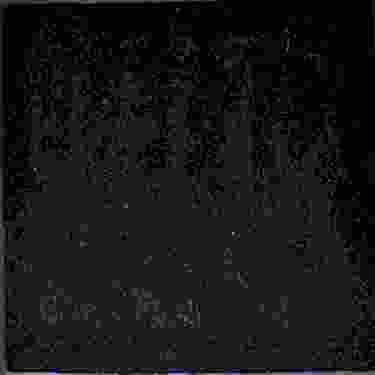

Supplement: Supplementary file 4 [file DataSheet4.zip › train/200150-2024-3-19-7-6-36.JPG]

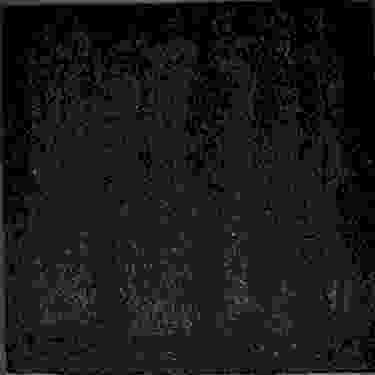

Supplement: Supplementary file 4 [file DataSheet4.zip › train/200150-2024-3-19-9-38-37.JPG]

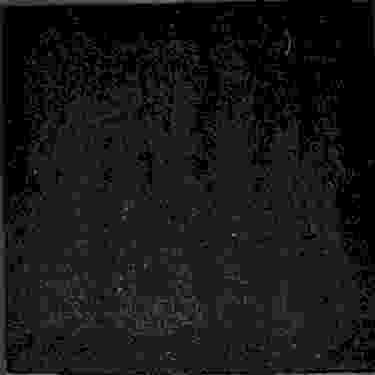

Supplement: Supplementary file 4 [file DataSheet4.zip › train/200150-2024-3-20-0-51-38.JPG]

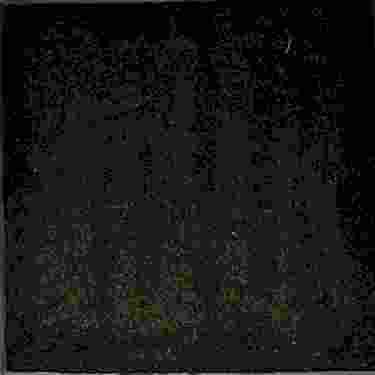

Supplement: Supplementary file 4 [file DataSheet4.zip › train/200150-2024-3-20-3-23-30.JPG]

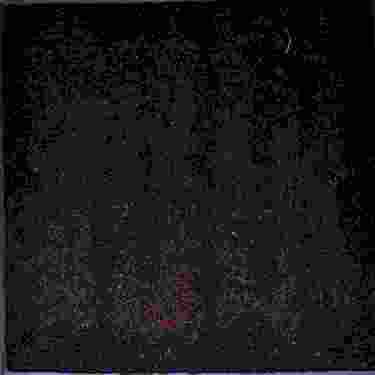

Supplement: Supplementary file 4 [file DataSheet4.zip › train/200150-2024-3-20-5-56-33.JPG]

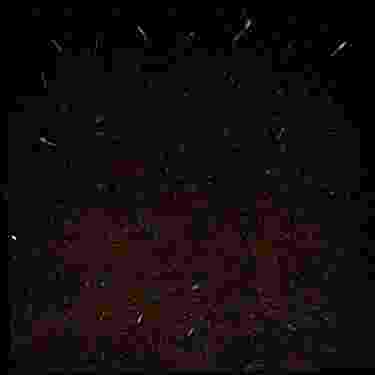

Supplement: Supplementary file 4 [file DataSheet4.zip › train/20030-2024-3-18-15-48-23.JPG]

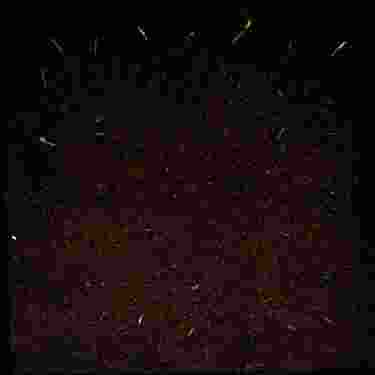

Supplement: Supplementary file 4 [file DataSheet4.zip › train/20030-2024-3-18-18-22-19.JPG]

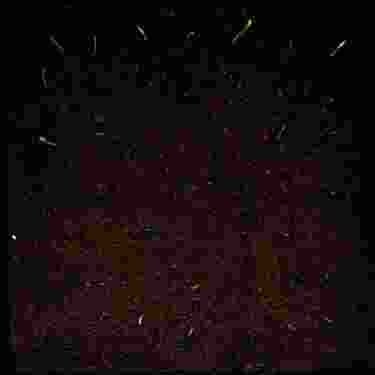

Supplement: Supplementary file 4 [file DataSheet4.zip › train/20030-2024-3-18-20-55-18.JPG]

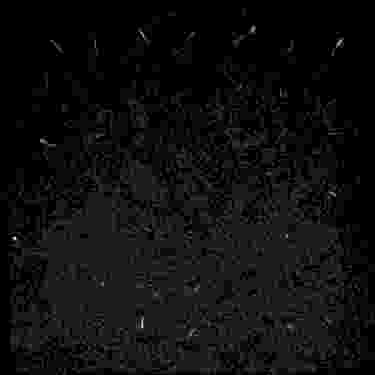

Supplement: Supplementary file 4 [file DataSheet4.zip › train/20030-2024-3-18-23-27-52.JPG]

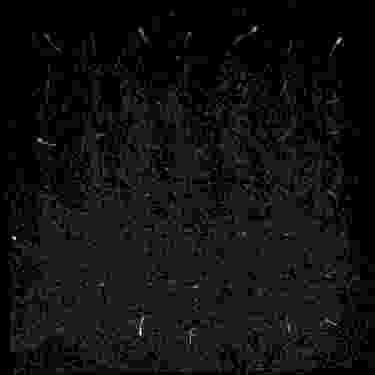

Supplement: Supplementary file 4 [file DataSheet4.zip › train/20030-2024-3-19-12-8-3.JPG]

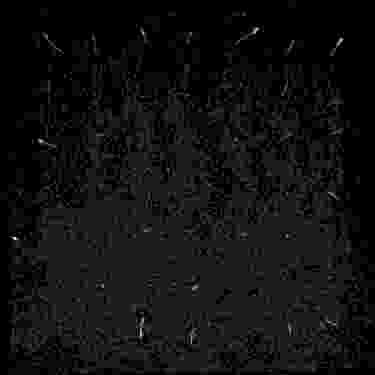

Supplement: Supplementary file 4 [file DataSheet4.zip › train/20030-2024-3-19-14-41-0.JPG]

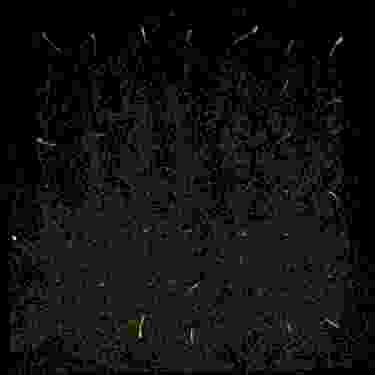

Supplement: Supplementary file 4 [file DataSheet4.zip › train/20030-2024-3-19-17-13-9.JPG]

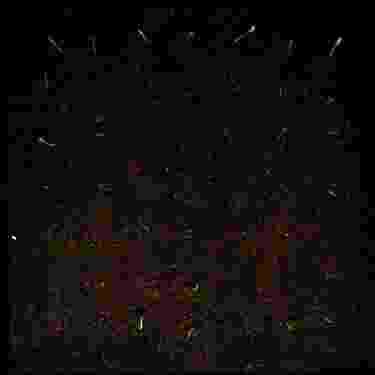

Supplement: Supplementary file 4 [file DataSheet4.zip › train/20030-2024-3-19-2-0-34.JPG]

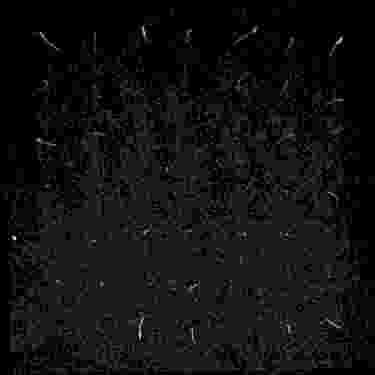

Supplement: Supplementary file 4 [file DataSheet4.zip › train/20030-2024-3-19-22-17-6.JPG]

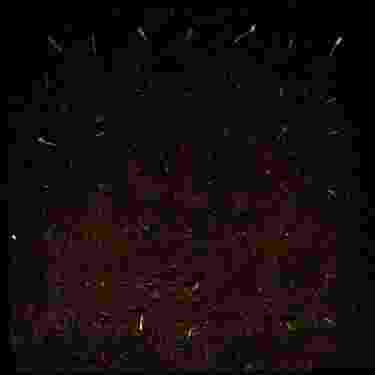

Supplement: Supplementary file 4 [file DataSheet4.zip › train/20030-2024-3-19-4-32-18.JPG]

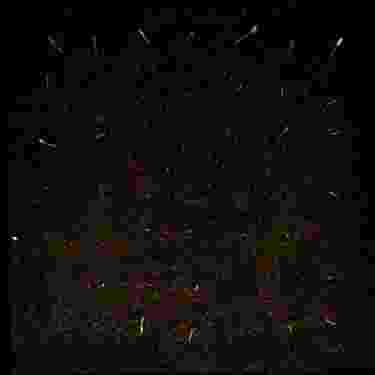

Supplement: Supplementary file 4 [file DataSheet4.zip › train/20030-2024-3-19-7-4-13.jpg]

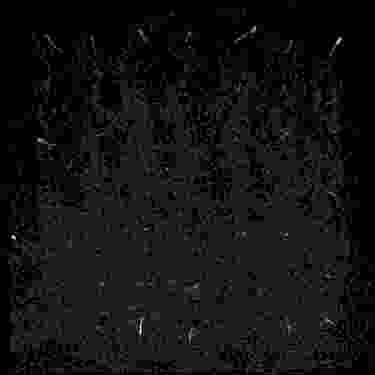

Supplement: Supplementary file 4 [file DataSheet4.zip › train/20030-2024-3-19-9-36-20.JPG]

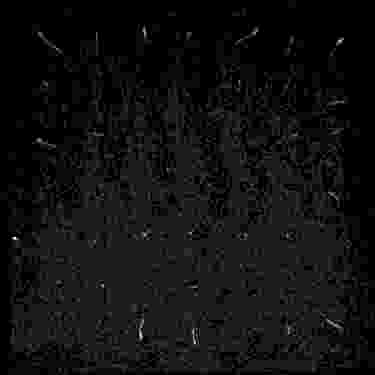

Supplement: Supplementary file 4 [file DataSheet4.zip › train/20030-2024-3-20-0-49-15.JPG]

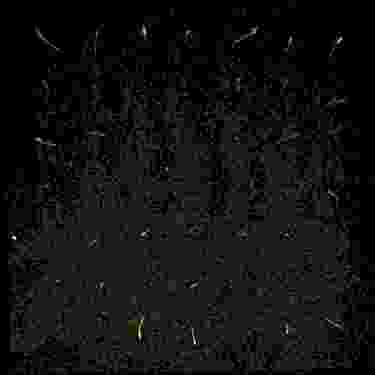

Supplement: Supplementary file 4 [file DataSheet4.zip › train/20030-2024-3-20-3-21-14.JPG]

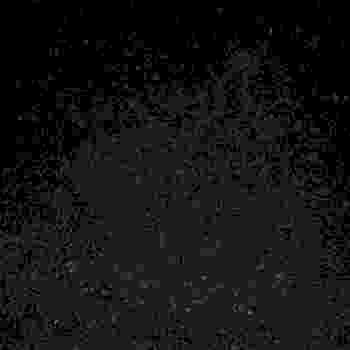

Supplement: Supplementary file 4 [file DataSheet4.zip › train/20060-2024-3-18-15-29-47.JPG]

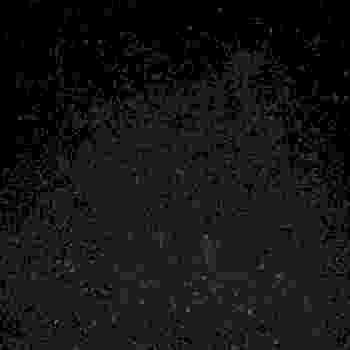

Supplement: Supplementary file 4 [file DataSheet4.zip › train/20060-2024-3-18-18-3-33.JPG]

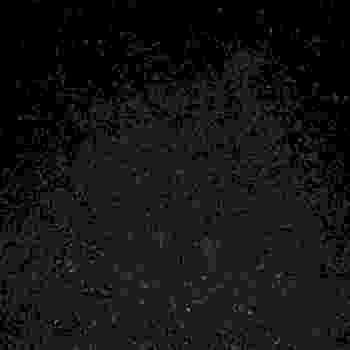

Supplement: Supplementary file 4 [file DataSheet4.zip › train/20060-2024-3-18-20-36-46.JPG]

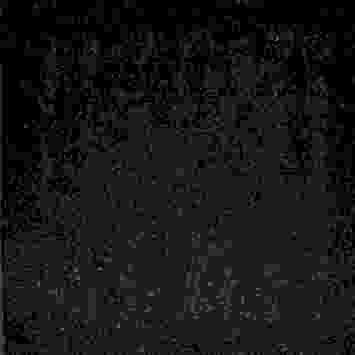

Supplement: Supplementary file 4 [file DataSheet4.zip › train/20060-2024-3-18-23-9-11.JPG]

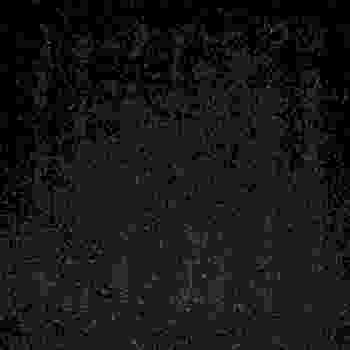

Supplement: Supplementary file 4 [file DataSheet4.zip › train/20060-2024-3-19-1-42-4.JPG]

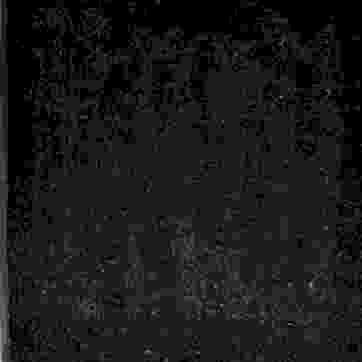

Supplement: Supplementary file 4 [file DataSheet4.zip › train/20060-2024-3-19-11-49-34.JPG]

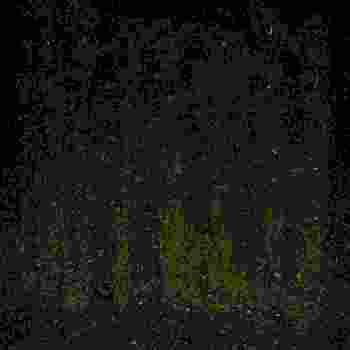

Supplement: Supplementary file 4 [file DataSheet4.zip › train/20060-2024-3-19-14-22-20.JPG]

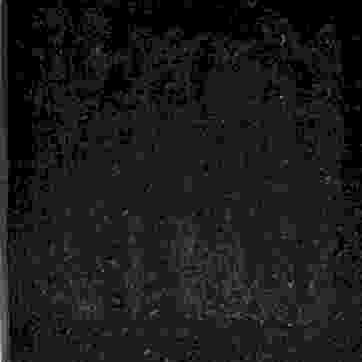

Supplement: Supplementary file 4 [file DataSheet4.zip › train/20060-2024-3-19-16-54-39.JPG]

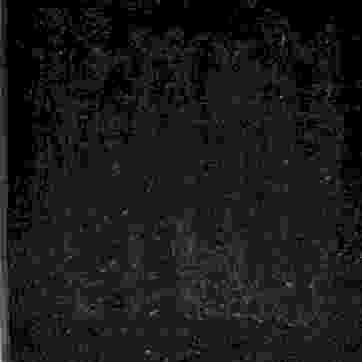

Supplement: Supplementary file 4 [file DataSheet4.zip › train/20060-2024-3-19-19-26-52.JPG]

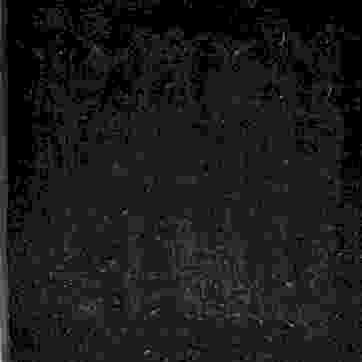

Supplement: Supplementary file 4 [file DataSheet4.zip › train/20060-2024-3-19-21-58-37.JPG]

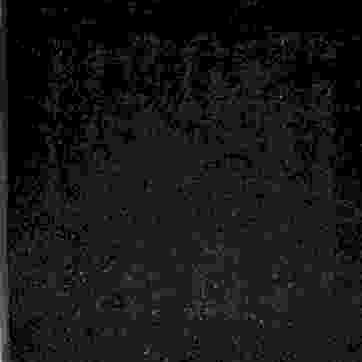

Supplement: Supplementary file 4 [file DataSheet4.zip › train/20060-2024-3-19-6-45-36.JPG]
